# High CD44 expression and enhanced E-selectin binding identified as biomarkers of chemoresistant leukemic cells in human T-ALL

**DOI:** 10.1038/s41375-024-02473-7

**Published:** 2024-11-24

**Authors:** Julien Calvo, Irina Naguibneva, Anthony Kypraios, Florian Gilain, Benjamin Uzan, Baptiste Gaillard, Lea Bellenger, Laurent Renou, Christophe Antoniewski, Helene Lapillonne, Arnaud Petit, Paola Ballerini, Stéphane JC. Mancini, Tony Marchand, Jean-François Peyron, Françoise Pflumio

**Affiliations:** 1 Université Paris Cité, Inserm, CEA, Stabilité Génétique Cellules Souches et Radiations, iRCM/SGCSR/Laboratoire des cellules Souches Hématopoïétiques et des Leucémies (LSHL), F-92260 Fontenay-aux-Roses, France; 2 Université Paris-Saclay, Inserm, CEA, Stabilité Génétique Cellules Souches et Radiations, iRCM/SGCSR/Laboratoire des cellules Souches Hématopoïétiques et des Leucémies (LSHL), F-92260 Fontenay-aux-Roses, France; 3 Laboratoire des cellules Souches Hématopoïétiques et des Leucémies, Equipe Niche et Cancer dans l’Hématopoïèse, équipe labellisée Ligue Nationale Contre le Cancer, Unité Mixte de Recherche (UMR) 1274-E008, Inserm, CEA, 92265 Fontenay-aux Roses, France; 4 OPALE Carnot Institute, The Organization for Partnerships in Leukemia, Paris, France; 5 Université Côte d’Azur, Centre Méditerranéen de Médecine Moléculaire (C3M), INSERM U1065, 06204 Nice, France; 6 ARTbio Bioinformatics Analysis Facility, IBPS, CNRS, Sorbonne Université, Institut Français de Bioinformatique, 75005 Paris, France; 7 Sorbonne University, AP-HP, Laboratory of Hematology, Armand-Trousseau Hospital, 75012 Paris, France; 8 Sorbonne Université, Centre de Recherche Saint-Antoine UMR_S938, Pediatric Hematology Oncology Unit, AP-HP, Armand-Trousseau Hospital, 75012 Paris, France; 9 Université Rennes, EFS, Inserm, MOBIDIC-UMR_S 1236, F-35000 Rennes, France; 10 Service d’hématologie Clinique, Centre Hospitalier Universitaire de Rennes, 35003 Rennes, France

**Keywords:** Acute lymphocytic leukaemia, Cancer microenvironment

## Abstract

T-cell acute lymphoblastic leukemia (T-ALL) is a hematopoietic malignancy characterized by increased proliferation and incomplete maturation of T-cell progenitors, for which relapse is often of poor prognosis. To improve patient outcomes, it is critical to understand the chemoresistance mechanisms arising from cell plasticity induced by the bone marrow (BM) microenvironment. Single-cell RNA sequencing of human T-ALL cells from adipocyte-rich and adipocyte-poor BM revealed a distinct leukemic cell population defined by quiescence and high CD44 expression (Ki67^neg/low^CD44^high^). During in vivo treatment, these cells evaded chemotherapy, and were further called Chemotherapy-resistant Leukemic Cells (CLCs). Patient sample analysis revealed Ki67^neg/low^CD44^high^ CLCs at diagnosis and during relapse, with each displaying a specific transcriptomic signature. Interestingly, CD44^high^ expression in T-ALL Ki67^neg/low^ CLCs was associated with E-selectin binding. Analysis of 39 human T-ALL samples revealed significantly enhanced E-selectin binding activity in relapse/refractory samples compared with drug-sensitive samples. These characteristics of chemoresistant T-ALL CLCs provide key insights for prognostic stratification and novel therapeutic options.

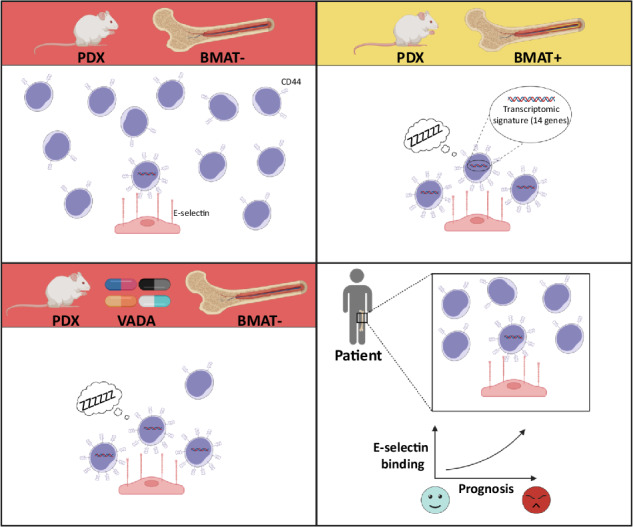

## Introduction

T-cell acute lymphoblastic leukemia (T-ALL) is characterized by the accumulation of genetic lesions that induce differentiation arrest, survival and aberrant proliferation of immature T-cell progenitors [1, 2]. T-ALL accounts for 15% of pediatric ALL cases and 25% of adult ALL cases. Although T-ALL prognosis has significantly improved due to intensive chemotherapy, relapses still occur in 20% of pediatric patients and 50% of adult patients, often with a dismal outcome [3]. To accurately classify patients based on risk of relapse, it is imperative to identify biomarkers indicative of relapse. These biomarkers can then be leveraged to identify novel drug targets and develop alternative therapies to treat patients with chemoresistant T-ALL.

Leukemic cells infiltrate various tissues including the bone marrow (BM), which provides an important microenvironment for T-ALL expansion. In the BM, leukemic cells take advantage of several BM-secreted factors that support the initiation or spread of acute myeloid leukemia or acute lymphoid leukemia, such as CXCL12 [4–7], Interleukins 7 and 18, Insulin Growth Factor 1, and Notch-ligands [8]. In addition to these soluble factors, multiple cells within the BM niches, such as hematopoietic cells, fibroblasts, osteoblasts/osteoclasts and neurons, also interact with leukemic cells [9, 10]. Adipocytes, which constitute BM adipose tissue (BMAT), are rare in young bones but markedly increase during the aging process or radio/chemotherapy [11–14]. Adipocytes have been shown to promote survival and/or proliferation of leukemic cells via 1) fatty acid assimilation [15–17], 2) intense synthesis of L-asparagine and glutamine [18], and 3) modulation of ROS production [19]. Due to these direct effects, adipocytes disturb the BM microenvironment. Indeed, adipocytes are negative regulators of the vasculature [11], which are involved in generating hypoxic areas that protect leukemic cells against chemotherapy [20, 21]. Understanding the interactions between adipocytes and leukemic cells may thus provide insight for improved patient care. Indeed, our group and others have previously shown that ALL cells infiltrate BMAT-rich sites, in which they display decreased metabolism and translational processes, accumulation in the G0 cell cycle phase, and a chemoresistance phenotype [14, 22]. This resistant cell state is plastic; when BMAT-seeded leukemic cells are relocated in regular hematopoietic BM niches, they transition back into a proliferative/activated state [22]. These characteristics may be leveraged to develop effective therapeutic alternatives to overcome BMAT-induced chemoresistance.

Given the role that the BM microenvironment plays in T-ALL chemoresistance, we aimed to fully characterize the chemoresistant quiescent T-ALL cells from BMAT sites. Using single-cell RNA sequencing (scRNAseq) analysis, we identified a minor but distinct quiescent (Ki67^low/neg^) cell population that exhibits high chemoresistance potential (called T-ALL Chemotherapy-resistant Leukemic Cells (CLCs)). The transcriptomic signature of the CLC population is highly similar to resting B-ALL cells, as previously described [23]. Furthermore, the CLC population expressed increased levels of both the standard form of CD44 and a tetrasaccharide carbohydrate termed the sialylated Lewis X (sLe^X^) motif, thus enabling interaction with E-selectin [24]. ScRNAseq analysis of primary T-ALL samples demonstrated the presence of a Ki67^low/neg^CD44^high^ CLC population both at diagnosis and during relapse, thereby underscoring the physiological relevance. Furthermore, CD44/E-selectin binding was enhanced in patients with poor prognosis, thus indicating that this attribute may be utilized to potentially stratify patients based on prognosis.

## Results

### Detection of a quiescent T-ALL cell cluster in adipocyte-poor and adipocyte-rich BMs

**Fig. 1:**
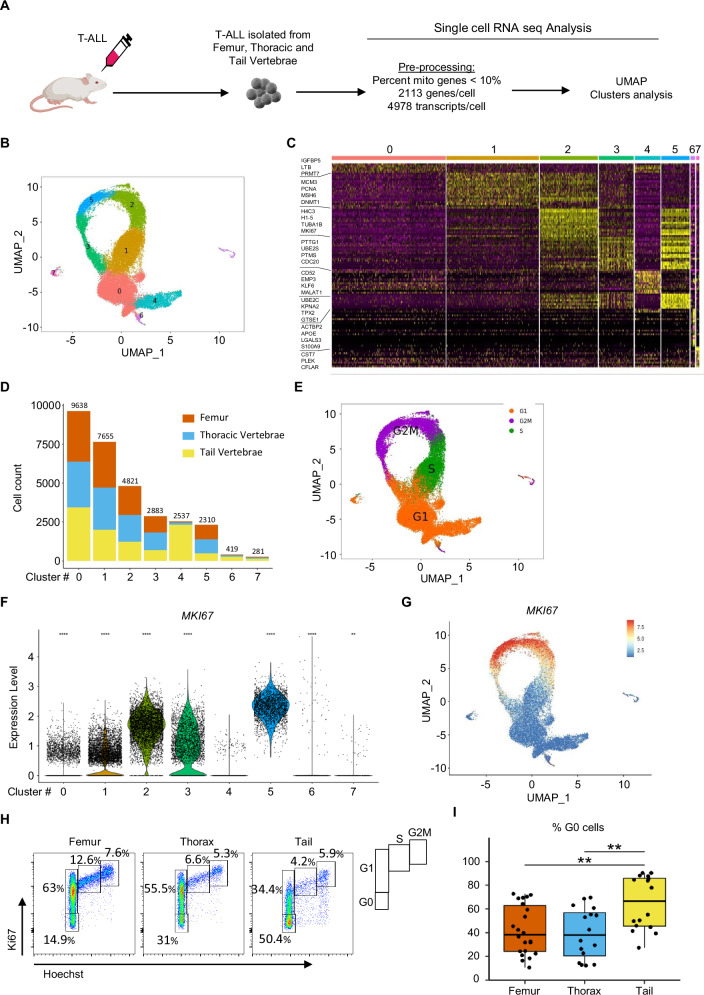
Characterization of leukemic cell diversity in adipocyte-rich/poor bone marrow. **A** Schematic overview of the study. **B** Uniform Manifold Approximation and Projection (UMAP) visualization of color-coded clustering of 30,544 huT-ALL (M18) cells from the femur, thorax and tail vertebrae (4 mice). **C** Expression of the 20 top differentially expressed genes (rows) across the cells (columns) in each cluster using the *DoHeatmap* function. Colored bars correspond to the color-coded clustering (**B**). **D** Number of cells from the femur (orange), thorax (blue) and tail vertebrae (yellow) in each cluster. The data are presented in cumulative bar plots. **E** UMAP visualization of cell cycle progression using the *CellCycleScoring* function (detailed in the Methods). *MKI67* expression analysis for each cluster represented by Violin Plot in which the points denote the values for each cell (**F**) and visualized on UMAP (**G**). Statistical significance was assessed using a Wilcoxon test for each cluster to the reference Cluster 4 (****p < 0.0001; **p < 0.01). **H**, **I** Cell cycle analysis by flow cytometry. Representative Ki67/Hoechst staining on human CD45^+^/CD7^+^ T-ALL M106 cells from the femur, thorax and tail (**H**). Frequency of quiescent (G0) huT-ALL cells from the femur (orange), thorax (blue) and tail vertebrae (yellow). The data are shown as box-and-whisker plots. Boxes indicate the 25^th^ and 75^th^ percentiles, whiskers display the range, and horizontal lines in each row represent the median. Four PDX models of huT-ALL were tested in 16–22 mice (**I**). Statistical significance was assessed by Kruskal-Wallis test followed by Dunn’s multiple comparisons test (**p < 0.01). The huT-ALL samples are described in Supplementary Table 1.

To characterize the leukemic cell diversity in different BMAT-poor/rich sites [14, 22], we isolated cells from BMAT-poor BM (also referred as red-BM) (femur, thoracic vertebrae) and BMAT-rich BM (also referred as yellow-BM) (tail vertebrae) (Supplementary Fig. 1A), then purified human leukemic huCD45^+^ cells for scRNAseq analysis (patient details in Supplementary Table 1). After quality filtering, we obtained 30 544 cells (see Materials and methods, Supplementary Fig. 1B) displaying a median of 2 113 genes/cell and 4 978 transcripts/cell (Fig. 1A). Unsupervised clustering using the principal component analysis (PCA) dimensional space enabled the visualization of eight different clusters of leukemic cells pooled from the femur, thorax and tail vertebrae via UMAP (Uniform Manifold Approximation and Projection) (Fig. 1B). The top 20 differentially expressed genes per cluster were characterized (Fig. 1C, Supplementary Table 2). While all clusters exhibited an equal distribution of cells across BM regions, Cluster 4 comprised 91.2% of cells from BMAT-rich sites (Fig. 1D, Supplementary Fig. 1C, D). Further analysis, comparing Cluster 4 to all other clusters of cells, revealed 383 upregulated genes and 569 downregulated genes with a log2 fold change >0.25 and an adjusted *p*-value < 0.05 (Supplementary Table 3, Supplementary Fig. 1E). Analysis of the cell cycle status in the scRNAseq dataset showed that cells from Clusters 0 and 4 were predominantly in the G1 phase, whereas cells from the other clusters were in the S/G2M phases (Fig. 1E). Low mRNA counts used to distinguish between the G0 and G1 phases as well as the absence of *MKI67* expression indicated that Cluster 4 contained cells primarily in G0 phase (Fig. 1F, G, Supplementary Fig. 1F). To understand whether Cluster 4 was only characterized by cell cycle progression genes, a second clustering analysis was performed by excluding the principal components associated with cell cycle-related genes. Cluster 4 remained distinct from the other clusters, indicating that this cluster does not only rely on cell cycle genes (Supplementary Fig. 1G). Using flow cytometry analysis, we found that leukemic cells from the femur and thorax/BMAT-poor BM were activated and cycling cells, while cells from tail vertebrae/BMAT-rich BM were mostly in a G0/quiescent state (Fig. 1H, I). Collectively, these findings underscore significant leukemic cell diversity in both red and yellow BM sites. The single-cell transcriptomic analysis further indicated that most leukemic cells isolated from the BMAT-rich/yellow BM sites constitute a homogeneous cluster associated with quiescence. Interestingly, a low (8.8%), but significant, proportion of leukemic cells from BMAT-poor sites displayed expression profiles similar to those from BMAT-rich/red sites (Supplementary Fig. 1C).

### Quiescent T-ALL subpopulations are associated with high-CD44 expression

**Fig. 2:**
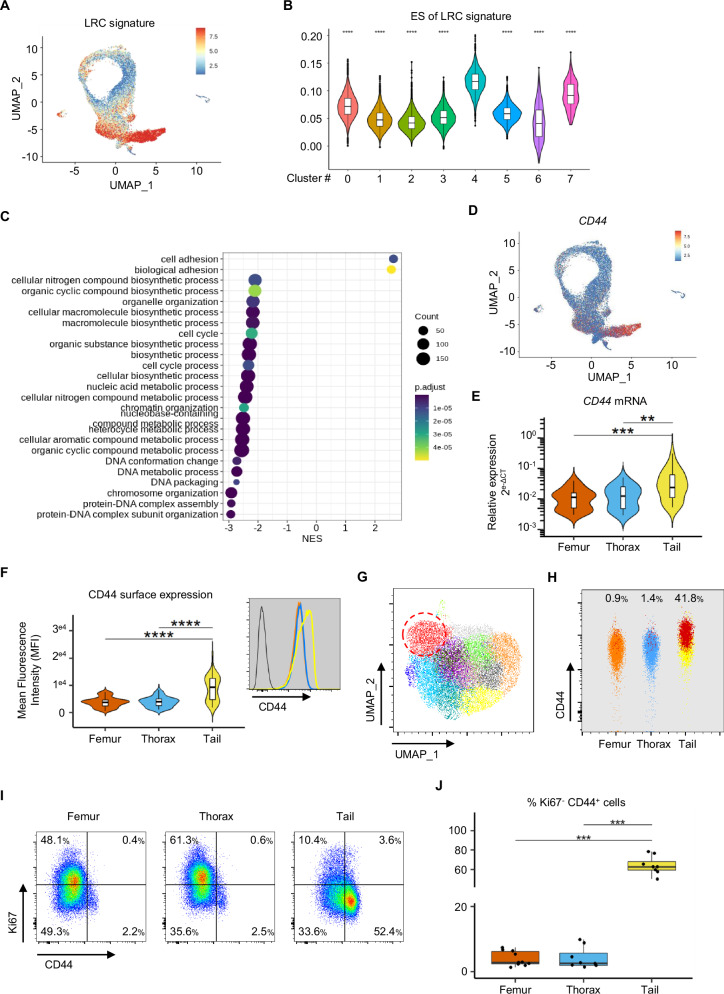
High expression of CD44 indicates quiescent/dormant T-ALL cells. Enrichment analysis (detailed in the Methods) of the LRC signature published by Ebinger et al. in huT-ALL cells from adipocyte-rich/poor BM visualized on UMAP (**A**) and represented for each cluster (**B**). **C** Gene Set Enrichment Analysis (GSEA) performed with significantly upregulated genes from Cluster 4 involved in “Biological Process” (Gene Ontology annotation). The data are shown as a normalized enrichment score (NES), and significant processes were defined by false discovery rate <0.05 (colors indicate the p.adjust level). **D** *CD44* expression analysis visualized on UMAP. **E** *CD44* relative expression analysis performed by RT-qPCR with huT-ALL cells from femur (orange), thorax (blue) and tail vertebrae (yellow). PDX models of 15 huT-ALL samples (37 mice). **F** Mean fluorescence intensity (MFI) of CD44 expression assessed by flow cytometry on huT-ALL cells from femur (orange), thorax (blue) and tail vertebrae (yellow). PDX of 19 huT-ALL samples (67 mice). Flow cytometry analysis example obtained with M18-PDX, huT-ALL cells unstained (black line), femur (orange line), thorax (blue line) and tail (yellow line) (inset). **G** UMAP visualization of 21,600 huT-ALL (M18) cells from femur, thorax and tail vertebrae; the color-coded clustering according to FSC, SSC, CD7, CD45, CD4, CD8, CXCR4, CD44 and CD34 surface expression as assessed via flow cytometry. A red dashed line indicates the “red cluster”, which displays the highest CD44 expression levels. **H** The frequency of “red clusters” in each region. **I**, **J** Flow cytometry analysis of CD44 and Ki67 expression. Representative CD44/Ki67 staining of huT-ALL M106 cells from each region (**I**). Frequency of Ki67^neg/low^CD44^high^ huT-ALL cells from each region (**J**). **E**–**J** The data are shown as violin and/or box-and-whisker plots. Boxes indicate the 25^th^ and 75^th^ percentiles, whiskers indicate the range, and horizontal lines in each row represent the median. **E**–**J** Statistical significance was assessed by Kruskal–Wallis test followed by Dunn’s multiple comparisons test (**p < 0.01; ***p < 0.005). The huT-ALL samples are described in Supplementary Table 1.

The transcriptomic signature of Cluster 4 was further compared with the label-retaining cell (LRC) transcriptomic signature that defines quiescent B-ALL cells and is enriched in B-ALL minimal residual disease (MRD) [23]. We observed a significant enrichment of the LRC signature in cells from Cluster 4 (Fig. 2A, B), which underscores a shared transcriptomic signature in quiescent and chemoresistant T/B-ALL cells. Over 18% of upregulated genes from the Cluster 4 signature were also upregulated in the LRC signature (Supplementary Fig. 2A, Supplementary Table 4). To define the biological mechanisms that are associated with quiescent/resistant leukemic cells, we performed a Gene Set Enrichment Analysis (GSEA) of the upregulated genes from Cluster 4. Consistent with previous observations [14, 22], most of the basal cellular processes such as “protein synthesis, mRNA catabolic processes and metabolism” were downregulated in leukemic cells from Cluster 4 (Fig. 2C and Supplementary Fig. 2B, C). The only significantly upregulated Gene Ontology (GO) biological processes highlighted in these resting leukemic cells were “Cell Adhesion” and “Biological Adhesion”, with normalized enrichment scores of 2.54 and 2.50, respectively (Fig. 2C). Accordingly, cellular component analysis revealed that upregulated genes encode proteins related to the “Localized to the cell membrane” GO term (Supplementary Fig. 2B). We analyzed the mRNA and protein expression of the chemokine receptor CXCR4, which plays a pivotal role in T-ALL migration/homing and niche adhesion [6, 7]. CXCR4 expression was not higher in cells from the adipocyte-rich BM (Supplementary Figure 3). The top five enriched genes involved in “Cell Adhesion” were S100A10, LGALS1, PTPRC, RIPOR2 and CD44 (Fig. 2D, Supplementary Fig. 4A–E). CD44 is a ubiquitous cell surface glycoprotein implicated in solid cancer and leukemia migration/adhesion [25–28]. Several CD44 variants, which are generated by alternative splicing, have been shown to be involved in cancer metastasis [29]. We determined which CD44 isoform is expressed in T-ALL cells using specific primers that target the different variants of *CD44* exons. We tested 12 patient-derived xenografts (PDX) obtained from four human T-ALL samples and found that leukemic cells carry the *CD44* standard isoform (Supplementary Fig. 5A), which is expressed at higher levels in cells from BMAT-rich/tail vertebrae compared with BMAT-poor BM (Fig. 2E). Furthermore, surface CD44 protein expression was also higher in leukemic cells from BMAT-rich/tail vertebrae (Fig. 2F), as confirmed by western blot analysis (Supplementary Fig. 5B). We also observed that protein and mRNA levels were significantly correlated (r = 0.75 with *p*-value = 1.509 ×10^−14^, Supplementary Fig. 5C), thus indicating that CD44 mRNA expression can be applied as a reliable surrogate for protein levels in our T-ALL cell conditions.

### High CD44 expression is not related to NOTCH1 activation and TAL1 levels, and CD44 is present in B-ALL

Activation of NOTCH1 signaling, a crucial regulator of T-ALL development/expansion [30], drives the expression of critical genes such as *Myc*, *Hes1*, *pTalpha*, *Deltex1* and *CD44* [31–33]. To investigate whether high CD44 expression in cells from BMAT-rich sites was due to NOTCH1 activation, we analyzed the mRNA expression of several NOTCH1 target genes using scRNAseq data and RT-qPCR [30]. The data revealed no change in NOTCH1 pathway activation in leukemic cells from BMAT-poor sites compared with BMAT-rich sites (Supplementary Fig. 6A–C). TAL1 transcription factor is recognized as a repressor of CD44 expression in chronic myeloid leukemia (CML) cells [28]. We found that CD44 expression was high in the tail vertebrae of mice, although they were transplanted with TAL1-positive T-ALL samples (Supplementary Fig. 6C–E). TAL1 mRNA expression levels were similar regardless of the BM region analyzed, implying that TAL1 expression did not affect CD44 expression in our conditions (Supplementary Fig. 6F). To assess whether the high CD44 expression observed in BMAT-rich sites was exclusive to T-ALL or may also occur in B-ALL, we analyzed CD44 expression in three B-ALL samples from different oncogenic groups (Supplementary Table 1). Consistent with the recent findings that B-ALL may recapitulate the T-ALL phenotype in yellow BM [14], we found that CD44 expression was also high in yellow BM compared with BMAT-poor sites using RT-qPCR and flow cytometry (Supplementary Fig. 7A, B). These findings consistently associate the CD44^high^ state with BMAT-rich/yellow BM for both T-ALL and B-ALL cells.

### CD44^high^ leukemic cells are present in all BM sites, regardless of BMAT richness

In parallel to scRNAseq analysis, we also performed flow cytometry to further characterize the leukemic cells. Using multiparametric flow cytometry and unbiased UMAP clustering integrating nine parameters, including CD44, we identified 12 clusters. Among these 12 clusters, a red-colored cluster (Fig. 2G) consisted primarily of tail vertebrae cells (95%, Supplementary Fig. 8A) and displayed the highest CD44 levels (Supplementary Fig. 8B). This CD44^high^ cell cluster (depicted as red dots in Fig. 2H) represented 0.9%, 1.4% and 41.8% of the leukemic cells isolated from the femur, thorax and tail vertebrae, respectively. These findings indicate that BMAT-poor BM also harbors a minor leukemic cell population, which resembles the major population recovered from BMAT-rich BM.

As with CD44^high^ leukemic cells from BMAT-rich sites (Fig. 1H, I), we noted that CD44^high^ leukemic cells found in all BM regions are quiescent (Ki67^neg^) (Fig. 2I, J). These findings are in line with the proportion of leukemic cells from BMAT-poor BM found in Cluster 4 (Supplementary Fig. 1C). Overall, our findings strongly suggest that high CD44 expression is associated with leukemic quiescence.

### In vivo chemotherapy efficacy is markedly attenuated in BMAT-rich BM compared with BMAT-poor BM, and CD44^high^ T-ALL cells exhibit inherent in vivo chemoresistance properties

**Fig. 3:**
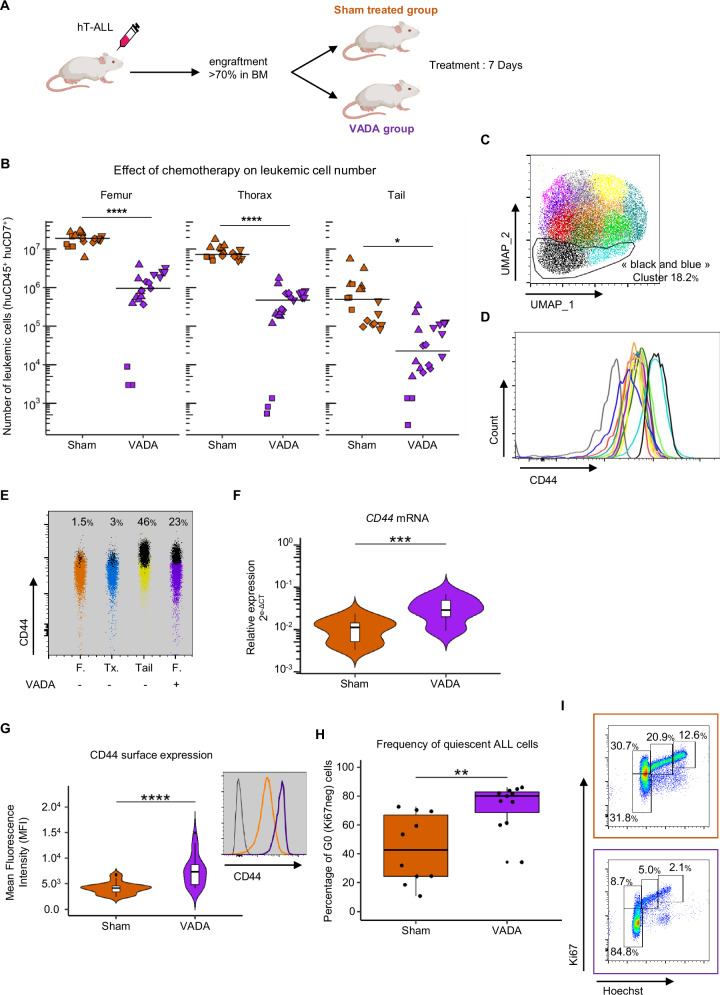
In vivo chemotherapy model to characterize minimal Residual Disease (MRD). **A** Schematic overview of the in vivo chemotherapy model. **B** Number of human CD45^+^/CD7^+^ T-ALL cells in each region after sham or VADA treatment. PDX of four huT-ALL samples (18 sham and 21 VADA mice). Square, rhombus, triangle and inverted triangle symbols represent M103, M69, M106 and M18 samples, respectively. **C**, **D** Cell heterogeneity evaluated by flow cytometry. UMAP visualization of 21,600 huT-ALL (M18) cells equally derived from femur, thorax and tail vertebrae of sham-treated mice mixed with 7,200 cells from the femurs of VADA-treated mice; PhenoGraph clustering is color-coded according to FSC, SSC, CD7, CD45, CD4, CD8, CXCR4, CD44 and CD34 surface expression (**C**). MFI of CD44 expression in huT-ALL (M18) from each cluster (**D**). **E** Frequency of “black & blue clusters” represented by black dots in the femur (orange), thorax (blue), tail (yellow) of sham-treated mice and the femur of VADA-treated mice (purple). **F** Relative expression of *CD44* mRNA analyzed via RT-qPCR with purified hCD45^+^ huT-ALL cells from the femurs of sham-treated mice (orange) and VADA-treated mice (purple). PDX of four huT-ALL samples (19 sham and 12 VADA mice). **G** MFI of CD44 expression analyzed by flow cytometry on human CD45^+^/CD7^+^ T-ALL cells from the femurs of sham-treated mice (orange) and VADA-treated mice (purple). PDX from four huT-ALL samples (35 sham mice and 23 VADA mice). A flow cytometry example of M106-PDX, huT-ALL cells unstained (black), from the femur of sham-treated mice (orange) and VADA-treated mice (purple) (inset). **H**, **I** Quiescent analysis. Frequency of quiescent huT-ALL cells from the femurs of sham-treated mice (orange) and VADA-treated mice (purple). **I** Representative Ki67/Hoechst staining of huT-ALL (M69) cells from the femurs of sham-treated mice (orange) and VADA-treated mice (purple). PDX from two huT-ALL samples (10 sham mice and 12 VADA mice) (**H**). **F**–**H** Data are shown as violin and/or box-and-whisker plots. Boxes indicate the 25^th^ and 75^th^ percentiles, whiskers indicate the range, and horizontal lines in each row represent the median. **D**–**F** Statistical significance was assessed via Mann & Whitney test (**p < 0.01; ***p < 0.005; ****p < 0.001). The huT-ALL samples are described in Supplementary Table 1.

To investigate whether the quiescent CD44^high^ T-ALL cells are chemoresistant, we administered a chemotherapy treatment in non-obese diabetic/severe combined immunodeficiency/interleukin-2Rγ null (NSG) mice transplanted with human T-ALL cells [34–37]. Mice with substantial leukemia infiltration were treated with either vehicle or a combination of Vincristine, Cytarabine, Dexamethasone and L-Asparaginase (VADA treatment, Fig. 3A and Supplementary Fig. 9A, B). One week of treatment induced a drastic reduction in tumor burden in the spleen and peripheral blood (Supplementary Fig. 9C, D). While the impact of VADA treatment was significant across all BM sites, the reduction in T-ALL cell burden was more pronounced in BMAT-poor BM than in BMAT-rich BM (Fig. 3B and Supplementary Fig. 9E). This observation indicates that drug treatment efficiency varies across BM sites, as previously observed in vitro [14, 22].

We next characterized the residual BM T-ALL cells that resisted VADA chemotherapy using comparative multi-parametric flow cytometry analysis of leukemic cells from the femur, thorax and tail vertebrae of sham-treated mice. An unbiased clustering approach using the UMAP algorithm coupled with PhenoGraph analysis revealed 12 clusters (Fig. 3C, D), in which CD44^high^ leukemic cells were predominantly localized in two clusters (“black and blue” clusters, Fig. 3D). As shown in Fig. 2H for the untreated PDX samples, the proportion of “black and blue” clusters was approximatively 50% in BMAT-rich sites and <3% in BMAT-poor sites for sham-treated mice (Fig. 3E). However, post-VADA treatment, leukemic cells from BMAT-poor sites exhibited at least a seven-fold increase compared with those observed in sham-treated mice (23% vs. <3% respectively; Fig. 3E). These findings show that human T-ALL CLC sub-populations, similar to those observed at high and low levels respectively in BMAT-poor and BMAT-rich sites, are enriched after chemotherapy. This enrichment may result either from the selection of CLC or from a CD44 upregulation, induced by chemotherapy agents.

To further characterize the selected leukemic cells post-VADA treatment, we analyzed CD44 expression in leukemic cells from treated or untreated femurs from mice xenografted with human T-ALL. Leukemic cells from VADA-treated femurs displayed elevated CD44 transcript and protein expression levels compared with sham-treated controls (Fig. 3F, G). As expected from the cell cycle-targeting drugs (e.g., Vincristine and Cytarabine), leukemic cells from the VADA-treated femurs were primarily quiescent (Fig. 3H, I).

Overall, our data show the presence of leukemic cells in BMAT-poor sites, phenotypically similar to resistant leukemic cells present in BMAT-rich sites and capable of evading chemotherapy. Furthermore, CD44^high^ expression correlates with a quiescent state and is a reliable marker to track chemoresistance.

### *CD44*^*high*^ and *Ki67*^*neg/low*^ biomarkers characterize a cell population present in T-ALL patients

**Fig. 4:**
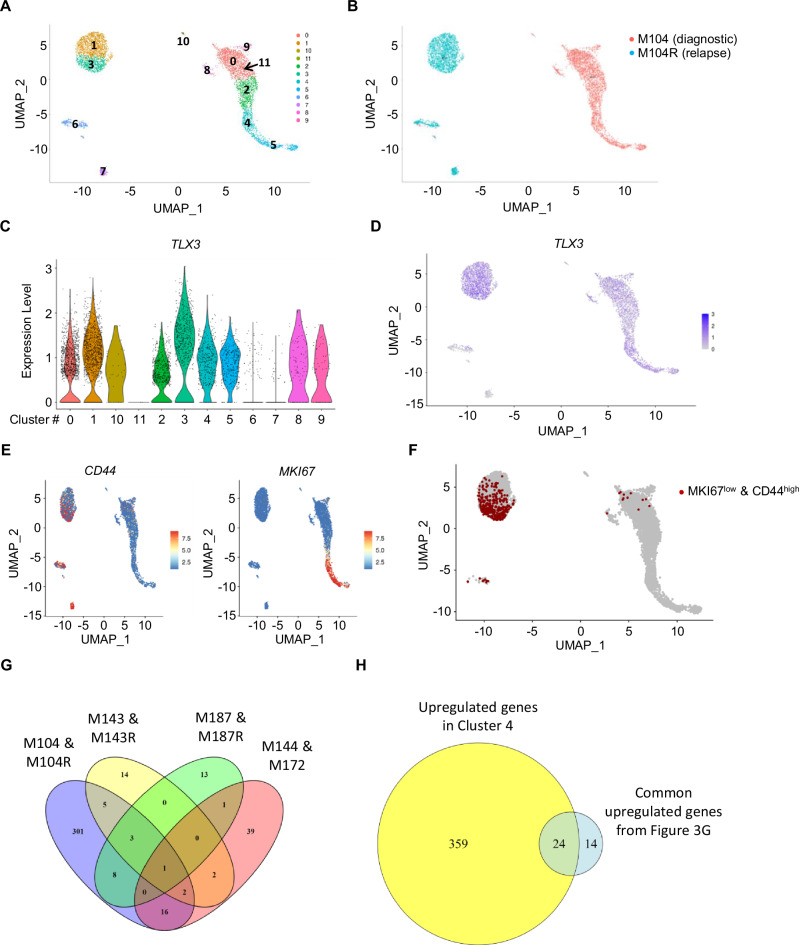
The *Ki67*^*neg/low*^*CD44*^*high*^ population is present in diagnosis and relapse huT-ALL samples. **A**–**F** Single-cell RNA sequencing of paired diagnosis (M104) and relapse (M104R) huT-ALL. UMAP color-coded clustering (**A**) and according to disease stage (diagnosis or relapse) (**B**). *TLX3* expression levels (**C**) overlaid onto a UMAP (**D**). Expression levels of *MKI67* and *CD44* overlaid onto a UMAP (**E**). **F** *MKI67*^*neg/low*^*CD44*^*high*^ population (red dots) overlaid onto a UMAP of huT-ALL cells. **G** Venn diagram of significantly upregulated genes identified in *MKI67*^*neg/low*^*CD44*^*high*^ huT-ALL cell populations from four libraries (M104 & M104R; M143 & M143R; M187 & M187R; M144 & M172). Values indicate the number of genes. **H** Venn diagram of common upregulated genes identified in *MKI67*^*neg/low*^*CD44*^*high*^ in at least two libraries (38 genes) and significantly upregulated genes identified in Cluster 4 (Fig. 1). The hu T-ALL samples are described in Supplementary Table 1.

To ensure the physiological relevance of the above described results, we performed scRNAseq analysis to assess the presence of *CD44*^*high*^ *Ki67*^*neg/low*^ leukemic cells in three T-ALL patient samples and compared their signature to the one of similar *CD44*^*high*^ *Ki67*^*neg/low*^ cells uncovered from the PDX models. The three leukemic patients were chosen according to the number of live cells from the paired Diagnosis and Relapse (Blood or BM, Supplementary Table 1) samples for scRNAseq analysis (Fig. 4 and Supplementary Fig. 10). In a fourth library, we added two patients at diagnosis that did not have relapse match as the patients responded well to treatment (Supplementary Fig. 11). The results of the UMAP and clustering analyses of pooled cells from each patient indicated several clusters, although diagnosis and relapse samples had specifically distinct gene profiles (Fig. 4A, B and Supplementary Fig. 10A, B, F, G). Analysis of the two additional T-ALL patients at diagnosis yielded the same observations, e.g. these samples had specific and distinct clustering in the UMAP (Supplementary Fig. 11A, B). To detect the leukemic cell population of interest, we excluded normal contaminating cells by retrieving cells 1) expressing oncogenic fusions (TLX3 expression in Figs. 4C, D and 2) specific lineage genes of non-T cells (B cells: *CD79A*, *CD79B*, *CD19*, *MS4A1*; *NKT* cells: *NKG7*, *GNLY*; and monocytes: *FCGR3A*, *S100A4*, *LYZ*, *CST3*, *CD33*, *CD14*) (Supplementary Figs. 10C, 10H and 11C). Having done that, we observed that *CD44* and *Ki67* were detected in all samples and their expression was mutually exclusive (Fig. 4E, Supplementary Figs. 10D, 10I and 11D). Interestingly *Ki67*^*neg/low*^*CD44*^*high*^ leukemic cells were only scarcely present in all samples, with the exception of one relapse sample (Fig. 4F, Supplementary Figs. 10E, 10J and 11E) as observed in leukemic cells from BMAT-poor sites in T-ALL models (Fig. 2H, I). To determine whether the *Ki67*^*neg/low*^*CD44*^*high*^ leukemic cells found in the different (diagnosis and relapse) patient samples share a gene signature with leukemic cells from BMAT-poor sites, we first crossed the transcriptomic profiles of the human *Ki67*^*neg/low*^*CD44*^*high*^ T-ALL patient cells (Supplementary Tables 5–8). We next selected 38 common significantly upregulated genes obtained from at least two libraries (Fig. 4G, Supplementary Table 9). By comparing with the expression pattern of Cluster 4, we found 24 (63%) upregulated shared genes (Figs. 1C and 4H, Supplementary Table 10). To determine whether this transcriptomic signature was specific to leukemic cells, we extended our analysis to *Ki67*^*neg/low*^*CD44*^*high*^ normal cells found in all libraries (Supplementary Fig. 12A–D, Supplementary Tables 11–14) and we compared the transcriptomic signature of *Ki67*^*neg/low*^*CD44*^*high*^ leukemic cells with the significantly upregulated genes found in *Ki67*^*neg/low*^*CD44*^*high*^ normal cells from the four libraries (Supplementary Fig. 12E, Supplementary Table 15). We found that 14/24 patient/cluster4 shared genes were specific to the leukemic cells, thereby suggesting that these 14 genes may constitute a transcriptomic signature of leukemic resistant cells (Supplementary Fig. 12F, Supplementary Table 16).

These observations demonstrate the presence of rare quiescent CD44^high^ leukemic cells in patient samples with an expression signature similar to human T-ALL from chemoresistant and BMAT-poor/rich sites from PDX models. To explore the benefit of high *CD44* expression as a prognosis biomarker, we evaluated *CD44* expression levels in a Children’s Oncology Group-Therapeutically Applicable Research to Generate Effective Treatments (COG-TARGET) cohort, which enabled the analysis of the expression profiles of 264 diagnostic human T-ALL samples [38]. Strikingly, the results revealed that patients with high *CD44* mRNA expression were significantly associated with HOXA, LMO2/LYL1 oncogenic subgroups, which are enriched in early T-cell precursors ALL (ETP-ALL) (Supplementary Figures 13A, B) [38–40] and present a poor prognosis after chemotherapy [41]. Together, these results show that the quiescent *CD44*^*high*^ cells described in human T-ALL PDX models are also detected in T-ALL patient samples, these cells have a specific gene signature, and *CD44* expression levels are enriched in Immature T-ALL subgroups that are associated with a poor prognosis, maybe in relation with an immaturity/progenitor cell state.

### CD44^high^ cells display an increase in E-selectin binding capacity

**Fig. 5:**
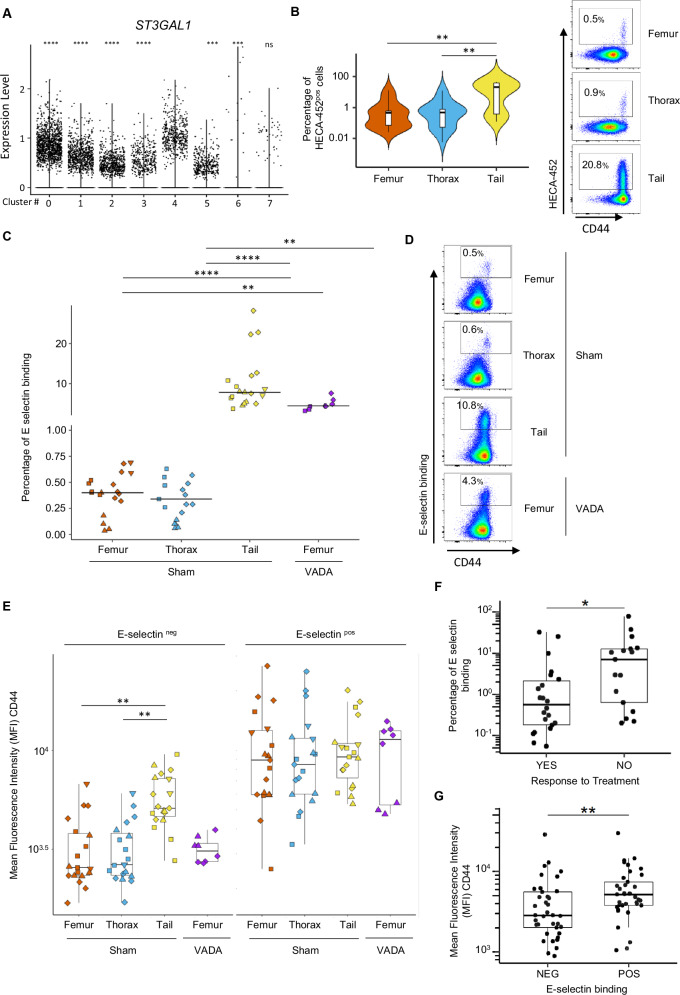
E-selectin binding is enhanced in CD44^high^ populations. **A** *ST3GAL1* expression level per cell in each cluster from scRNAseq analysis. Data are shown as points denoting values for each cell. **B** The percentage of HECA-452-positive cells in human CD45^+^/CD7^+^ T-ALL cells from the femur (orange), thorax (blue) and tail vertebrae (yellow) assessed via flow cytometry. PDX of five huT-ALL samples (17 mice). Flow cytometry analysis of M106-PDX, huT-ALL cells unstained (black line), femur (orange line), thorax (blue line) and tail (yellow line) (inset). **C** The frequency of huT-ALL cells from the femur (orange), thorax (blue), tail (yellow) of sham-treated mice and the femur of VADA-treated mice (purple). **D** Representative E-selectin binding/CD44 staining of huT-ALL (M106) cells from the femur, thorax and tail of sham-treated mice and the femur of VADA-treated mice as analyzed via flow cytometry. **E** MFI of CD44 in huT-ALL cells from the femur (orange), thorax (blue) and tail (yellow) of sham-treated mice and the femur of VADA-treated mice (purple) with in vitro E-selectin binding and non-binding controls. PDX of four huT-ALL samples (27 mice) **F** The proportion of E-selectin-bound huT-ALL cells according to treatment response (“NO” vs “YES”). **G** MFI of CD44 expression in huT-ALL cells according to E-selectin binding status (“NEG” vs “POS”). **E**–**G** The data are shown as box-and-whisker plots. The boxes indicate the 25^th^ and 75^th^ percentiles, whiskers indicate the range, and horizontal lines in each row represent the median. **B**–**D** Statistical significance was assessed via Kruskal–Wallis test followed by Dunn’s multiple comparisons test (**p < 0.01; ****p < 0.001). **F**, **G** Statistical significance was assessed via Mann & Whitney test (**p < 0.01). The huT-ALL samples are described in Supplementary Table 1.

CD44 is known to interact with a wide variety of ligands, such as hyaluronic acid and E-selectin [42]. At steady state, endothelial cells within the BM naturally express E-selectin [43]. The expression of E-selectin is further increased by inflammatory agents such as TNF-α or IL-1β-mediated activation [44], both of which are secreted by adipocytes in the BM [45]. To demonstrate E-selectin binding activity, CD44 as well as several other ligands, such as P-selectin glycoprotein ligand-1 (PSGL-1/CD162) and Leukosialin (CD43), must bear multiple sLe^X^ motifs. This motif results from a succession of post-translational modifications, such as the addition of a Fucose at α1-3 to the residue of an N-Acetylglucosamine, itself linked at β1-4 to galactose associated at α2-3 with a sialic acid group. The addition of this motif is therefore the result of modifications made by various enzymes: Fucosyltransferases (FUT3/4/6/7/9), O-linked N-Acetylglucosaminetransferases and Sialyltransferases (ST3GAL1/3/4/6). We found that ST3GAL1 is significantly overexpressed in Cluster 4 and FUT7 is only expressed by some leukemic cells of Cluster4 (Fig. 5A; Supplementary Fig. 14A). These observations were validated by qRT-PCR performed with leukemic cells isolated from BMAT-poor and –rich sites (Supplementary Fig. 14B, C). Upon analysis of the sLe^x^ motif on the surface of leukemic cells using HECA-452 staining [46], we found that leukemic cells expressing high levels of CD44 from BMAT-rich and BMAT-poor sites harbored sLe^x^ motifs (Fig. 5B). Subsequently, we evaluated the attachment efficacy of a chimeric soluble human E-selectin-IgG1 fusion protein to leukemic cells recovered from various BM regions. As anticipated, a greater proportion of leukemic cells from the BMAT-rich site bound E-selectin compared with the BMAT-poor sites (respective median: 7.8% and 0.4% E-selectin^+^ cells) (Fig. 5C). Interestingly, T-ALL cells from the VADA-treated BMAT-poor sites also exhibited higher E-selectin-binding levels (median: 4.5% E-selectin^+^ cells) compared with untreated mice (Fig. 5C). Using flow cytometry analysis, we found that regardless of the BM site analyzed, binding to E-selectin was detected in cells with the highest levels of CD44 expression (Fig. 5D, E). These observations further support the earlier findings obtained with HECA-452 staining.

E-selectin binding was also measured in leukemic cells from 46 patient samples. Using the clinical information available for 39/46 patients, we classified the samples into two groups: sensitive and aggressive cases, the latter including refractory and relapsed patients. Assessing information regarding patient treatment response and E-selectin binding levels revealed that relapse/refractory patients (the “NO” response group) had 10-fold higher levels of E-selectin binding compared with chemotherapy-sensitive patients (the “YES” response group) (median: 7.0% vs. 0.6% (Fig. 5F)). Interestingly, regardless of the patient samples analyzed, the T-ALL cells more prone to E-selectin binding were CD44^high^ (Fig. 5G, Supplementary Fig. 14D). Notably, E-selectin binding potential was not related to EGIL grade classification for the 29/39 patients for which information was available (Supplementary Fig. 14E). We analyzed samples from a patient (called M225) collected during treatment (e.g. Minimal Residual Disease, MRD). We detected 2.7% of residual leukemic cells, consistent with the molecular MRD data obtained by the clinic. At diagnosis, CD44 expression level on leukemic cells was medium, with 3.6% being CD44^high^/HECA-452^+^ (Supplementary Fig. 14F). During treatment, flow cytometry identified leukemic cells, all of them were CD44^high^ with 41% being HECA-452^+^ (Supplementary Fig. 14F). To further characterize the CD44^high^ cells, we compared sorted CD44^high^/E-selectin^+^ and CD44^low/-^/E-selectin^-^ cells for their in vivo leukemia development ability after transplantation at limiting dilution (Supplementary Fig. 15A). We observed that both cell fractions were able to engraft mice at all cell doses (5.10^4^, 1.10^4^, 1.10^3^) (Supplementary Fig. 15B). Differences in cell infiltration are observed at the 10^3^ cells/mouse with lower levels of leukemic cells in CD44^high^/E-selectin^+^ engrafted mice compared to CD44^low/-^/E-selectin^-^ injected mice (Supplementary 15b). Albeit we cannot exclude that the CD44^high^/E-selectin^+^ cells are less invasive than the CD44^low/-^/E-selectin^-^ cells thus containing lower leukemia initiating cells, this result could be related to a slower growth ability, as we previously observed for the total BMAT-cell population [22]. Importantly, *FUT7* and *ST3GAL1* expression levels were also tested in the sorted CD44^high^/E-selectin^+^ vs CD44^low/-^/E-selectin^-^ cells. As expected, CD44^high^/E-selectin^+^ cells have enhanced *FUT7* and *ST3GAL1* as compared with CD44^low/-^/E-selectin^-^ cells (Supplementary Fig. 15C).

Overall, we found that T-ALL cells capable of E-selectin binding are CD44^high^ both in T-ALL models and human patients. From a clinical perspective, our results suggest that high CD44 and E-selectin binding potential can be leveraged to stratify relapse/refractory patients.

## Discussion

Although cases of childhood ALL in high-income countries have cure rates of approximately 90%, the prognosis among ALL adults remains poor, with <45% of patients expected to survive disease-free for the long term. Furthermore, patients with relapse/refractory T-ALL experience a poor prognosis with overall survival rates at <10% for adults and <25% for children [3]. In this context, few innovative treatments are available. Nelarabine is effective in the adult and pediatric populations and is the only FDA-approved therapy. Progress has been made to apply chimeric antigen receptor-T-cell therapy (which is effective to treat B-ALL) as a second-line treatment for T-ALL after therapeutic failure [47–49]. However, further research is necessary to develop novel therapeutic strategies. Interestingly, our group and others have found that ALL cells are able to downregulate their surface marker expression in relation to decreased translational activity, which may contribute to potential chimeric antigen receptor-T-cell escape observed in B-ALL [14, 22]. In the present study, we observed multiple clusters of leukemic cells in the BM, with distinct subpopulations residing in adipocyte-rich (BMAT-rich) and adipocyte–poor (BMAT-poor) sites. We identified a quiescent cluster (Cluster 4) predominantly consisting of leukemic cells from BMAT-rich sites, which displayed additional cell adhesion properties, thus implicating CD44 involvement. The correlation between high CD44 expression and a quiescent state suggests that these characteristics may serve as a potential marker to track chemoresistant cells, which is supported by the observation that CD44 is associated with chemoresistance in various hematological diseases [25, 26, 28]. We show that transcriptional and translational upregulation of CD44 is associated with sLe^x^ motif creation and facilitates E-selectin binding. In T-ALL cells, we found that CLCs (which are more numerous in BMAT-rich BM and less numerous in BMAT-poor BM) are able to bind E-selectin in experimental models, similar to that observed in cells detected in patients. Pro-inflammatory cytokines (TNF-α and IL-1β) have been shown to enhance E-selectin expression in inflamed BM endothelium [43, 50]. We speculate that E-selectin expressed by endothelial cells, may attach to Ki67^neg/low^CD44^high^ T-ALL cells and thus constitute a chemoresistant BM niche. Uproleselan (developed by Glycomimetics), which disrupts E-selectin receptor/ligand interactions, was evaluated in a phase 3 trial to treat relapsed/refractory AML patients [51, 52]. Even though the trial results did not reach significant improvement in overall survival (NCT03616470), this antagonist may still represent an interesting therapeutic molecule to target quiescent chemoresistant T-ALL cells. Nevertheless, CD44 has several other ligands such as hyaluronic acid and osteopontin, which could be of interest in explaining the quiescence state [53].

Although recent results have revealed that chemoresistant T-ALL cells are not localized in a specific niche, they are consistent with observations that the quiescent state is a specific behavior associated with leukemic cell chemoresistance [37, 54]. Our results obtained with in vivo VADA treatment support this conclusion. Moreover, we demonstrate that the quiescent state observed after treatment in BMAT-poor sites resembles the quiescent state observed in BMAT-rich sites, with high CD44 expression and increased E-selectin binding. These observations support the idea that the Ki67^neg/low^CD44^high^ leukemic cells with E-selectin binding activity represent a chemoresistant leukemic cell population. Interestingly, a LDA experiment showed that CD44^high^/E-selectin^+^ chemoresistant leukemic cells could propagate leukemia rather less efficiently than CD44^low/-^/E-selectin^-^ cells, maybe in relation with their lower proliferative rate, thus uncoupling chemoresistance from leukemia propagation abilities.

The high CD44 expression observed in ETP-ALL may not be strictly related to the quiescent state but may be consistent with the CD44 expression levels observed during early T-cell differentiation [55]. Our data actually fit with the findings of Hoofd and colleagues, which suggested that subpopulations of CD44-expressing cells exhibit differential resistance to chemotherapy, or chemotherapy itself upregulates CD44 [56]. Additional experiments are necessary to investigate whether cluster 4 CD44^high^ cells have enhanced drug efflux pump activity that would give a rational for their chemoresistance.

Relapse remains the major cause of death for ALL patients. Ebinger et al. have described a chemoresistance model based on the persistence of dormant/non-dividing BCP-ALL cells (also referred to as LRCs) located close to BM endosteal regions [23]. LRCs display a transcriptomic signature that is enriched in patients with MRD compared with patients at the time of diagnosis [23]. In our study, the scRNAseq Cluster 4 in which we identified Ki67^neg/low^CD44^high^ cells, is highly enriched for the LRC signature, thus indicating common chemoresistance phenotypes. Moreover, scRNAseq analysis of a combination of leukemic cells from patients and experimental models displayed that Ki67^neg/low^CD44^high^ leukemic cells harbor a specific 14 gene-transcriptomic signature. In AML, a 17- or 6-gene score has been defined based on the gene expression of CLCs with properties such as dormancy and provides key prognostic insight [57, 58]. Similarly, a 9-gene score has been proposed to facilitate risk stratification in B-ALL based on the LRC transcriptomic signature [59]. Some of the 14 transcripts in our Ki67^neg/low^CD44^high^ T-ALL cell signature encode for markers implicated in cancer cell migration (MALAT1, Metastasis-associated lung adenocarcinoma transcript 1 [60]), immune checkpoints / tolerance (HLA-B/C/E, Human Leukocyte Antigen [61]), protein synthesis through the inhibition of rRNA processing (PNRC1, Proline-rich Nuclear Receptor Coactivator 1 [62]) and drug resistance (ARL4C, ADP ribosylation like factor 4 C [63]). Interestingly, MALAT1 was significantly upregulated in MRD+ compared with MRD- patients and highly expressed in relapse patients compared with new cases of pediatric T- and B-ALL [64]. Our 14-gene signature may represent a new MRD signature specific to T-ALL, as nine of the genes are also present in the B-ALL LRC signature [23]. While the NOTCH1 pathway is widely described as being responsible form the transcriptional activation of CD44 via BRD4, our data showing low NOTCH1 and MYC expression in Cluster 4 do not support this hypothesis [31–33]. In contrast, we observed in the transcriptomic signature of Cluster 4 the presence of transcription factors (FOS, JUND, JUN and ETS1), which are described in the literature as transcriptional regulators of CD44 [65, 66]. Additional ChIP-Seq experiments are necessary to uncover which factors participate in the upregulation of CD44 in cluster 4 cells.

In summary, this study provides comprehensive insights into the heterogeneity of T-ALL cells in the BM microenvironment and highlights a specific transcriptomic signature of T-ALL chemoresistance. The expression of the sLe^x^ motif, which enables binding to E-selectin, associated with high CD44 expression indicates its potential as a prognostic biomarker of chemoresistance. Our results further demonstrate that E-selectin binding is a relevant marker to stratify relapse/refractory patients.

## Materials and methods

### Human T-ALL

Blood and/or bone marrow (BM) samples from patients with human (hu)T-ALL were collected at Hôpital R. Debré, Hôpital A. Trousseau (Paris, France) and Hôpitaux Civils de Lyon (Lyon, France) and processed as previously described [22]. Some newly diagnosed leukemic cell samples were used directly after isolation for in vitro and in vivo experiments, while the rest were frozen in fetal calf serum (FCS) containing 10% dimethyl sulfoxide (DMSO) (D8418, Sigma-Aldrich). The huT-ALL samples are described in Supplementary Table 1.

### Human T-ALL xenografts

Patient-derived xenografts (PDX) were established from huT-ALL biopsy samples in non-obese diabetic/severe combined immunodeficiency/interleukin-2Rγ null mice (NSG, The Jackson Laboratory, Bar Harbor, ME, USA), which were bred in pathogen-free animal facilities (Commissariat à l’Energie Atomique et aux Energies Alternatives [CEA], Fontenay-aux-Roses, France). Leukemic cells were injected intravenously into non-irradiated mice. BM samples were extracted after anesthesia (isoflurane) and analgesia (3 µg/mL Buprenorphine) to monitor huT-ALL expansion in vivo. Leukemic cell infiltration was evaluated using immuno-labeling with anti-huCD45 and anti-huCD7 antibodies (see Supplementary Table 17) using a FACS-Canto II flow cytometer (Becton Dickinson, BD). Cells from the femur, thorax or tail vertebrae were isolated [22]. For RT-PCR and scRNAseq analysis, huT-ALL cells were purified after labeling with anti-huCD45 phycoerythrin (PE) conjugated antibody followed by immuno-magnetic selection with anti-PE microbeads (130-048-801, Miltenyi Biotec).

### Flow cytometry

Phenotype, cell cycle progression and absolute number of huT-ALL cells, were assessed by flow cytometry using FACS Canto II and LSR II systems (BD). Data analysis was carried out using FlowJo software. The cells were stained with fluorescein isothiocyanate (FITC)-, PE-, Peridinin chlorophyl protein-Cyanine 5.5 (PerCP Cy5.5)-, PE-cyanin7 (PC7)-, allophycocyanin (APC)-, allophycocyanin eFluor780 (APC-eFluor780)-, allophycocyanin Vio770 (APC-Vio770)-, Brilliant Violet 421 (BV421)-conjugated mouse monoclonal antibodies specific for human markers. All antibodies were purchased from BD Pharmingen, Miltenyi Biotec or e-Bioscience (Supplementary Table 17). Absolute number of cells was quantified by determining the number of huCD45^+^/huCD7^+^ cells in the BM samples. For cell cycle analysis, cells were stained with anti-huCD45 and anti-huCD7 antibodies and then permeabilized using Cytofix/Cytoperm (554722, BD Biosciences) for 15 min at 4 °C, washed with Perm/Wash Buffer (554723, BD Biosciences) and then labeled with anti-Ki67 antibodies (556027 or 556026, BD Biosciences) at 4 °C for 45 min. Hoechst 33342 (H3570, Life Technologies) was added at 20 µg/mL 10 min before the end of the incubation period. The cells were then washed with Perm/Wash Buffer and resuspended in PBS.

### 10x genomics single-cell package and library preparation

After purification, the cells were quantified with trypan blue and then suspended at 1 ×10^6^ cells/mL in PBS 0.04% BSA. Next, 20,000 cells per condition were loaded in Chromium Next GEM Chip G. Single-cell libraries were generated using Chromium Next GEM Single Cell 3’ Reagent Kits (v3.1): GEM, Library and Gel Bead Kit v3.1 (PN-1000128), Chip G Single-Cell kit (PN-1000127) and Dual Index kit TT Set A (PN-1000215) (10x Genomics) according to the Chromium Next GEM Single Cell 3’ Reagent Kits (v3.1) User Guide (manual part no. CG0000204 Rev D). Reverse transcription and library preparation were performed using a Veriti 96-Well Thermal Cycler (Thermo Fisher Scientific). Amplified cDNA and libraries were verified using a Bioanalyzer 2100 (Agilent Technologies) with a High Sensitivity DNA kit (Agilent Technologies). The libraries were sequenced (Read 1: 28 bp, Index 7: 10 bp, Index 5: 10 bp and Read 2: 90 bp) on a NovaSeq 6000 Sequencing System (Illumina).

### Pre-processing of scRNA-seq data

Sequencing results were demultiplexed and then converted to FASTQ format. Galaxy was used for raw data processing, demultiplexing, barcode processing, and aligning with the GRCh38/hg38 reference genome. The EmptyDrops method was used to remove the barcodes associated with a Unique Molecular Identifier (UMI) below 50. After this process, we obtained 11,334, 10,914 and 11,356 cells from femur, thorax and tail vertebrae samples, respectively.

Quality filtering, normalization, dimensionality reduction, unsupervised clustering and identification of the differentially expressed genes were performed using the *Seurat v4* R package [67]. Filtration/pre-processing was carried out to remove low quality cells. Indeed, cells with >10% of mitochondrial transcripts were removed (in which there were <100, <95 and <85 mRNA in femur, thorax and tail vertebrae samples, respectively (Supplementary Fig. 1B). After pre-processing, the median number of detected genes and transcripts per cell were 2113 and 4978, respectively (Fig. 1A). The data was normalized by the overall median mRNA count and log-transformed.

### Dimensionality reduction

For each dataset, we identified a subset of 2000 variable genes with the highest dispersion using the *FindVariableFeatures* function in the *Seurat v4* package [67]. Dimensionality reduction was performed using these variable genes. To remove the batch effect, we used the RCPA (Reciprocal PCA) method. First, we found a set of anchors between the three seurat objects (femur, thorax and tail vertebrae) using the *FindIntegrationAnchors* function, and we then integrated the objects using the *IntegrateData* function. This process generates a batch-corrected expression matrix used as an input for the principal component analysis (PCA). We identified the first 35 principal components as relevant based on the Jack Straw method (*JackStraw* and *ScoreJackStraw* functions) by applying a *p*-value drop off for the principal components observed on the *Jack Straw Plot*.

### Visualization and clustering

To visualize the data, we reduced the dimensionality to project cells in 2D space using the *RunUMAP (Uniform Manifold Approximation and Projection)* function [68]. Clusters were identified using the *FindClusters* function following the *FindNeighbors* function with resolution of 0.4. Regardless of the resolution used, Cluster 4 retained its identity up to a resolution of 1.2 (data not shown).

### Differential expression of gene signatures

To identify differentially expressed genes per cluster we performed the Wilcoxon Rank Sum test using the *FindAllMarkers* function. The significance threshold was *p*-value-adjusted (Bonferroni correction) <0.05 and log2 fold change >0.25.

### In vivo chemotherapy

Conventional drug treatment was adapted from Samuels et al. [36]. When huT-ALL in BM represented at least 70%, mice were randomized for chemotherapy treatment. We injected a one-week schedule of Vincristine (0.25 mg/kg, i.v., Monday), Cytarabine (Ara-C, 2.5 mg/kg, i.v., Monday), Dexamethasone (5 mg/kg, i.p., every day Monday-Friday) and L-Asparaginase (1000U/kg, i.p., every day Monday-Friday) (referred to as VADA treatment). During the treatment, the mice were closely monitored for signs of drug-related toxicity (weight loss [Supplementary Fig. 7B], lethargy, shaggy fur) and euthanized at the first sign of morbidity. At the end of treatment (3 days after the final injection), the mice were euthanized and BM infiltration was evaluated.

### RNA extraction and real-time PCR

Total RNA was extracted from purified huT-ALL cells using RNeasy Plus Mini or Micro Kit (Qiagen). cDNA was synthesized using SuperScript® VILO^TM^ cDNA Synthesis Kit (Thermo Fisher Scientific). Real-time PCR reactions were carried out using *Power* SYBR® Green PCR Master Mix (Thermo Fisher Scientific) with a StepOne Real-time PCR system (Applied Biosystems). The forward and reverse primer sequences are described in Supplementary Table 18. The data were normalized using the *GAPDH* Ct values.

### Western blot

Protein was extracted with lysis buffer containing 100 mM Tris pH8, 100 mM NaCl, 1 mM EDTA, 1 mM EGTA, 1% NP40, 0.5% DOC, 50% Glycerol, 0.1% SDS and cocktail of protease inhibitors. The protein was then separated by 4–12% SDS PAGE, transferred onto a nitrocellulose membrane (Schleicher & Schuell) and immunoblotted in standard conditions. The primary antibodies used were mouse anti-human CD44s (pan-specific) and rabbit anti-b-actin. The secondary antibodies used were goat anti-mouse and goat anti-rabbit. All antibodies were purchased from Bio-Techne, Sigma-Aldrich or Abcam (Supplementary Table 18).

### E-selectin binding

To analyze E-selectin binding potential, recombinant E-selectin–human-IgG1 fusion protein (724 ES 100, R&D Systems) was pre-complexed at 5 µg/mL with PE-conjugated goat anti-human IgG diluted at 1/100^e^ (Supplementary Table 2) for 2 h at ambient temperature in PBS CaCl_2_ MgCl_2_ (14040133, Thermo Fisher Scientific). A total of 200,000 huT-ALL cells pre-stained with cell surface markers were incubated with the complex. After 30 min at ambient temperature, the cells were washed in PBS and analyzed by flow cytometry. For each experiment, a negative (non-binding) control was included (an identical parallel stain in the presence of 15 mM EDTA (E-selectin binding is strictly Ca^2+^-dependent)).

### Statistical analyses

Statistical analysis was performed using R software. The values are presented as the mean ± standard error of mean (SEM) or median through boxplot. Differences between the means of two experimental conditions with non-normal distribution were analyzed using non-parametric Wilcoxon–Mann–Whitney test. For multiple-group comparisons, when *n* < 30 with non-normal distribution, a Kruskal–Wallis test with Dunn’s post-test for pairwise multiple comparisons was applied. False discovery rates were corrected by the Benjamini–Hochberg stepwise adjustment [69]. Differences with *p* < 0.05 (*), *p* < 0.01 (**), *p* < 0.001 (***) or *p* < 0.0001 (****) were considered statistically significant.

### Study approval

All animal experiments were conducted after approval by the local ethical committee and authorization from the French Ministère de l’Enseignement Supérieur et de la Recherche (animal facility agreement number: C9203202) for the care and use of laboratory animals. Experimental procedures were specifically approved by the local Ethical Committee (CEEA 26: A21_021, APAFIS#9458-2017033110277117 v2). Informed consent of patients or relatives were obtained in accordance with the Declaration of Helsinki and the Ethics regulations. The research project was approved by the Inserm ethics evaluation committee (IORG0003254, FWA00005831). No compensation was provided to patients.

## Supplementary information

Supplemental Figures

Supplemental Table 1

Supplemental Table 2

Supplemental Table 3

Supplemental Table 4

Supplemental Table 5

Supplemental Table 6

Supplemental Table 7

Supplemental Table 8

Supplemental Table 9

Supplemental Table 10

Supplemental Table 11

Supplemental Table 12

Supplemental Table 13

Supplemental Table 14

Supplemental Table 15

Supplemental Table 16

Supplemental Table 17

Supplemental Table 18

## Data Availability

The RNA-seq data reported in this study are available at the Gene Expression Omnibus repository under the accession number GSE233600 and GSE262271 [70]. For original data, please contact julien.calvo@cea.fr.

## References

[1] Belver L, Ferrando A. The genetics and mechanisms of T cell acute lymphoblastic leukaemia. Nat Rev Cancer. 2016;16:494–507. 27451956 10.1038/nrc.2016.63

[2] Girardi T, Vicente C, Cools J, De Keersmaecker K. The genetics and molecular biology of T-ALL. Blood. 2017;129:1113–23. 28115373 10.1182/blood-2016-10-706465 PMC5363819

[3] Vadillo E, Dorantes-Acosta E, Pelayo R, Schnoor M. T cell acute lymphoblastic leukemia (T-ALL): New insights into the cellular origins and infiltration mechanisms common and unique among hematologic malignancies. Blood Rev. 2018;32:36–51. 28830639 10.1016/j.blre.2017.08.006

[4] Agarwal P, Isringhausen S, Li H, Paterson AJ, He J, Gomariz Á, et al. Mesenchymal Niche-Specific Expression of Cxcl12 Controls Quiescence of Treatment-Resistant Leukemia Stem Cells. Cell Stem Cell. 2020;26:123. 31901250 10.1016/j.stem.2019.11.013 PMC6951806

[5] Schelker RC, Iberl S, Müller G, Hart C, Herr W, Grassinger J. TGF-β1 and CXCL12 modulate proliferation and chemotherapy sensitivity of acute myeloid leukemia cells co-cultured with multipotent mesenchymal stromal cells. Hematology. 2018;23:337–45. 29140182 10.1080/10245332.2017.1402455

[6] Passaro D, Irigoyen M, Catherinet C, Gachet S, Da Costa De Jesus C, Lasgi C, et al. CXCR4 Is Required for Leukemia-Initiating Cell Activity in T Cell Acute Lymphoblastic Leukemia. Cancer Cell. 2015;27:769–79. 26058076 10.1016/j.ccell.2015.05.003

[7] Pitt LA, Tikhonova AN, Hu H, Trimarchi T, King B, Gong Y, et al. CXCL12-Producing Vascular Endothelial Niches Control Acute T Cell Leukemia Maintenance. Cancer Cell. 2015;27:755–68. 26058075 10.1016/j.ccell.2015.05.002 PMC4461838

[8] Calvo J, Fahy L, Uzan B, Pflumio F. Desperately seeking a home marrow niche for T-cell acute lymphoblastic leukaemia. Adv Biol Regul. 2019;74:100640. 31378700 10.1016/j.jbior.2019.100640

[9] Hoggatt J, Kfoury Y, Scadden DT. Hematopoietic Stem Cell Niche in Health and Disease. Annu Rev Pathol Mechanisms Dis. 2016;11:555–81. 10.1146/annurev-pathol-012615-044414 27193455

[10] Schepers K, Campbell TB, Passegué E. Normal and Leukemic Stem Cell Niches: Insights and Therapeutic Opportunities. Cell Stem Cell. 2015;16:254–67. 25748932 10.1016/j.stem.2015.02.014 PMC4391962

[11] Zhou BO, Yu H, Yue R, Zhao Z, Rios JJ, Naveiras O, et al. Bone marrow adipocytes promote the regeneration of stem cells and haematopoiesis by secreting SCF. Nat Cell Biol. 2017;19:891–903. 28714970 10.1038/ncb3570 PMC5536858

[12] Li Z, Hardij J, Bagchi DP, Scheller EL, MacDougald OA. Development, regulation, metabolism and function of bone marrow adipose tissues. Bone. 2018;110:134–40. 29343445 10.1016/j.bone.2018.01.008 PMC6277028

[13] Tikhonova AN, Dolgalev I, Hu H, Sivaraj KK, Hoxha E, Cuesta-Domínguez Á, et al. The bone marrow microenvironment at single-cell resolution. Nature. 2019;569:222–8. 30971824 10.1038/s41586-019-1104-8 PMC6607432

[14] Heydt Q, Xintaropoulou C, Clear A, Austin M, Pislariu I, Miraki-Moud F, et al. Adipocytes disrupt the translational programme of acute lymphoblastic leukaemia to favour tumour survival and persistence. Nat Commun. 2021;12:5507. 34535653 10.1038/s41467-021-25540-4 PMC8448863

[15] Rozovski U, Harris DM, Li P, Liu Z, Jain P, Ferrajoli A, et al. STAT3-activated CD36 facilitates fatty acid uptake in chronic lymphocytic leukemia cells. Oncotarget. 2018;9:21268–80. 29765537 10.18632/oncotarget.25066 PMC5940394

[16] Ye H, Adane B, Khan N, Sullivan T, Minhajuddin M, Gasparetto M, et al. Leukemic Stem Cells Evade Chemotherapy by Metabolic Adaptation to an Adipose Tissue Niche. Cell Stem Cell. 2016;19:23–37. 27374788 10.1016/j.stem.2016.06.001 PMC4938766

[17] Tabe Y, Yamamoto S, Saitoh K, Sekihara K, Monma N, Ikeo K, et al. Bone Marrow Adipocytes Facilitate Fatty Acid Oxidation Activating AMPK and a Transcriptional Network Supporting Survival of Acute Monocytic Leukemia Cells. Cancer Res. 2017;77:1453–64. 28108519 10.1158/0008-5472.CAN-16-1645 PMC5354955

[18] Ehsanipour EA, Sheng X, Behan JW, Wang X, Butturini A, Avramis VI, et al. Adipocytes Cause Leukemia Cell Resistance to L-Asparaginase via Release of Glutamine. Cancer Res. 2013;73:2998–3006. 23585457 10.1158/0008-5472.CAN-12-4402 PMC3684066

[19] Sheng X, Tucci J, Parmentier JH, Ji L, Behan JW, Heisterkamp N, et al. Adipocytes cause leukemia cell resistance to daunorubicin via oxidative stress response. Oncotarget. 2016;7:73147–59. 27705905 10.18632/oncotarget.12246 PMC5341969

[20] Bruno S, Mancini M, De Santis S, Monaldi C, Cavo M, Soverini S. The Role of Hypoxic Bone Marrow Microenvironment in Acute Myeloid Leukemia and Future Therapeutic Opportunities. Int J Mol Sci. 2021;22:6857. 34202238 10.3390/ijms22136857 PMC8269413

[21] Fahy L, Calvo J, Chabi S, Renou L, Le Maout C, Poglio S, et al. Hypoxia favors chemoresistance in T-ALL through an HIF1α-mediated mTORC1 inhibition loop. Blood Adv. 2021;5:513–26. 33496749 10.1182/bloodadvances.2020002832 PMC7839374

[22] Cahu X, Calvo J, Poglio S, Prade N, Colsch B, Arcangeli ML, et al. Bone marrow sites differently imprint dormancy and chemoresistance to T-cell acute lymphoblastic leukemia. Blood Adv. 2017;1:1760–72. 29296822 10.1182/bloodadvances.2017004960 PMC5728329

[23] Ebinger S, Özdemir EZ, Ziegenhain C, Tiedt S, Castro Alves C, Grunert M, et al. Characterization of Rare, Dormant, and Therapy-Resistant Cells in Acute Lymphoblastic Leukemia. Cancer Cell. 2016;30:849–62. 27916615 10.1016/j.ccell.2016.11.002 PMC5156313

[24] Merzaban JS, Burdick MM, Gadhoum SZ, Dagia NM, Chu JT, Fuhlbrigge RC, et al. Analysis of glycoprotein E-selectin ligands on human and mouse marrow cells enriched for hematopoietic stem/progenitor cells. Blood. 2011;118:1774–83. 21659548 10.1182/blood-2010-11-320705 PMC3158712

[25] Krause DS, Lazarides K, von Andrian UH, Van Etten RA. Requirement for CD44 in homing and engraftment of BCR-ABL–expressing leukemic stem cells. Nat Med. 2006;12:1175–80. 16998483 10.1038/nm1489

[26] Jin L, Hope KJ, Zhai Q, Smadja-Joffe F, Dick JE. Targeting of CD44 eradicates human acute myeloid leukemic stem cells. Nat Med. 2006;12:1167–74. 16998484 10.1038/nm1483

[27] Yu X, Munoz-Sagredo L, Streule K, Muschong P, Bayer E, Walter RJ, et al. CD44 loss of function sensitizes AML cells to the BCL-2 inhibitor venetoclax by decreasing CXCL12-driven survival cues. Blood. 2021;138:1067–80. 34115113 10.1182/blood.2020006343

[28] Godavarthy PS, Kumar R, Herkt SC, Pereira RS, Hayduk N, Weissenberger ES, et al. The vascular bone marrow niche influences outcome in chronic myeloid leukemia via the E-selectin - SCL/TAL1 - CD44 axis. Haematologica. 2020;105:136–47. 31018977 10.3324/haematol.2018.212365 PMC6939533

[29] Yaghobi Z, Movassaghpour A, Talebi M, Abdoli Shadbad M, Hajiasgharzadeh K, Pourvahdani S, et al. The role of CD44 in cancer chemoresistance: A concise review. Eur J Pharmacol. 2021;903:174147. 33961871 10.1016/j.ejphar.2021.174147

[30] Armstrong F, de la Grange PB, Gerby B, Rouyez MC, Calvo J, Fontenay M, et al. NOTCH is a key regulator of human T-cell acute leukemia initiating cell activity. Blood. 2009;113:1730–40. 18984862 10.1182/blood-2008-02-138172

[31] Sanchez-Martin M, Ferrando A. The NOTCH1-MYC highway toward T-cell acute lymphoblastic leukemia. Blood. 2017;129:1124–33. 28115368 10.1182/blood-2016-09-692582

[32] García-Peydró M, Fuentes P, Mosquera M, García-León MJ, Alcain J, Rodríguez A, et al. The NOTCH1/CD44 axis drives pathogenesis in a T cell acute lymphoblastic leukemia model. J Clin Invest. 2018;128:2802–18. 29781813 10.1172/JCI92981 PMC6025994

[33] Piya S, Yang Y, Bhattacharya S, Sharma P, Ma H, Mu H, et al. Targeting the NOTCH1-MYC-CD44 axis in leukemia-initiating cells in T-ALL. Leukemia. 2022;36:1261–73. 35173274 10.1038/s41375-022-01516-1 PMC9061299

[34] Kang MH, Kang YH, Szymanska B, Wilczynska-Kalak U, Sheard MA, Harned TM, et al. Activity of vincristine, L-ASP, and dexamethasone against acute lymphoblastic leukemia is enhanced by the BH3-mimetic ABT-737 in vitro and in vivo. Blood. 2007;110:2057–66. 17536015 10.1182/blood-2007-03-080325

[35] Szymanska B, Wilczynska-Kalak U, Kang MH, Liem NLM, Carol H, Boehm I, et al. Pharmacokinetic Modeling of an Induction Regimen for In Vivo Combined Testing of Novel Drugs against Pediatric Acute Lymphoblastic Leukemia Xenografts. PLOS ONE. 2012;7:e33894. 22479469 10.1371/journal.pone.0033894 PMC3315513

[36] Samuels AL, Beesley AH, Yadav BD, Papa RA, Sutton R, Anderson D, et al. A pre-clinical model of resistance to induction therapy in pediatric acute lymphoblastic leukemia. Blood Cancer J. 2014;4:e232. 25083816 10.1038/bcj.2014.52 PMC4219466

[37] Hawkins ED, Duarte D, Akinduro O, Khorshed RA, Passaro D, Nowicka M, et al. T-cell acute leukaemia exhibits dynamic interactions with bone marrow microenvironments. Nature. 2016;538:518–22. 27750279 10.1038/nature19801 PMC5164929

[38] Liu Y, Easton J, Shao Y, Maciaszek J, Wang Z, Wilkinson MR, et al. The genomic landscape of pediatric and young adult T-lineage acute lymphoblastic leukemia. Nat Genet. 2017;49:1211–8. 28671688 10.1038/ng.3909 PMC5535770

[39] Ferrando AA, Neuberg DS, Staunton J, Loh ML, Huard C, Raimondi SC, et al. Gene expression signatures define novel oncogenic pathways in T cell acute lymphoblastic leukemia. Cancer Cell. 2002;1:75–87. 12086890 10.1016/s1535-6108(02)00018-1

[40] Soulier J, Clappier E, Cayuela JM, Regnault A, García-Peydró M, Dombret H, et al. HOXA genes are included in genetic and biologic networks defining human acute T-cell leukemia (T-ALL). Blood. 2005;106:274–86. 15774621 10.1182/blood-2004-10-3900

[41] Coustan-Smith E, Mullighan CG, Onciu M, Behm FG, Raimondi SC, Pei D, et al. Early T-cell precursor leukaemia: a subtype of very high-risk acute lymphoblastic leukaemia. Lancet Oncol. 2009;10:147–56. 19147408 10.1016/S1470-2045(08)70314-0 PMC2840241

[42] Zöller M. CD44, Hyaluronan, the Hematopoietic Stem Cell, and Leukemia-Initiating Cells. Front Immunol. 2015;6. Available from: https://www.frontiersin.org/journals/immunology/articles/10.3389/fimmu.2015.00235/full. 10.3389/fimmu.2015.00235 PMC4443741 26074915

[43] Xu C, Gao X, Wei Q, Nakahara F, Zimmerman SE, Mar J, et al. Stem cell factor is selectively secreted by arterial endothelial cells in bone marrow. Nat Commun. 2018;9:2449. 29934585 10.1038/s41467-018-04726-3 PMC6015052

[44] Yao L, Setiadi H, Xia L, Laszik Z, Taylor FB, McEver RP. Divergent Inducible Expression of P-Selectin and E-Selectin in Mice and Primates. Blood. 1999;94:3820–8. 10572097

[45] Mattiucci D, Maurizi G, Izzi V, Cenci L, Ciarlantini M, Mancini S, et al. Bone marrow adipocytes support hematopoietic stem cell survival. J Cell Physiol. 2018;233:1500–11. 28574591 10.1002/jcp.26037

[46] Dimitroff CJ, Lee JY, Fuhlbrigge RC, Sackstein R. A distinct glycoform of CD44 is an L-selectin ligand on human hematopoietic cells. Proc Natl Acad Sci. 2000;97:13841–6. 11095749 10.1073/pnas.250484797 PMC17663

[47] Maciocia PM, Wawrzyniecka PA, Maciocia NC, Burley A, Karpanasamy T, Devereaux S, et al. Anti-CCR9 chimeric antigen receptor T cells for T-cell acute lymphoblastic leukemia. Blood. 2022;140:25–37. 35507686 10.1182/blood.2021013648

[48] Gomes-Silva D, Srinivasan M, Sharma S, Lee CM, Wagner DL, Davis TH, et al. CD7-edited T cells expressing a CD7-specific CAR for the therapy of T-cell malignancies. Blood. 2017;130:285–96. 28539325 10.1182/blood-2017-01-761320 PMC5520470

[49] Sánchez-Martínez D, Baroni ML, Gutierrez-Agüera F, Roca-Ho H, Blanch-Lombarte O, González-García S, et al. Fratricide-resistant CD1a-specific CAR T cells for the treatment of cortical T-cell acute lymphoblastic leukemia. Blood. 2019;133:2291–304. 30796021 10.1182/blood-2018-10-882944 PMC6554538

[50] Fodil S, Arnaud M, Vaganay C, Puissant A, Lengline E, Mooney N, et al. Endothelial cells: major players in acute myeloid leukaemia. Blood Rev. 2022;54:100932. 35148910 10.1016/j.blre.2022.100932

[51] Barbier V, Erbani J, Fiveash C, Davies JM, Tay J, Tallack MR, et al. Endothelial E-selectin inhibition improves acute myeloid leukaemia therapy by disrupting vascular niche-mediated chemoresistance. Nat Commun. 2020;11:2042. 32341362 10.1038/s41467-020-15817-5 PMC7184728

[52] DeAngelo DJ, Jonas BA, Liesveld JL, Bixby DL, Advani AS, Marlton P, et al. Phase 1/2 study of uproleselan added to chemotherapy in patients with relapsed or refractory acute myeloid leukemia. Blood. 2022;139:1135–46. 34543383 10.1182/blood.2021010721 PMC11017789

[53] O’Reilly E, Zeinabad HA, Szegezdi E. Hematopoietic versus leukemic stem cell quiescence: Challenges and therapeutic opportunities. Blood Rev. 2021;50:100850. 34049731 10.1016/j.blre.2021.100850

[54] Barz MJ, Behrmann L, Capron D, Zuchtriegel G, Steffen FD, Kunz L, et al. B- and T-cell acute lymphoblastic leukemias evade chemotherapy at distinct sites in the bone marrow. Haematologica. 2023;108:1244–58. 36325888 10.3324/haematol.2021.280451 PMC10153534

[55] Canté-Barrett K, Mendes RD, Li Y, Vroegindeweij E, Pike-Overzet K, Wabeke T, et al. Loss of CD44dim Expression from Early Progenitor Cells Marks T-Cell Lineage Commitment in the Human Thymus. Front Immunol. 2017;8. Available from: https://www.frontiersin.org/journals/immunology/articles/10.3389/fimmu.2017.00032/full. 10.3389/fimmu.2017.00032 PMC5247458 28163708

[56] Hoofd C, Wang X, Lam S, Jenkins C, Wood B, Giambra V, et al. CD44 promotes chemoresistance in T-ALL by increased drug efflux. Exp Hematol. 2016;44:166–71.e17. 26708679 10.1016/j.exphem.2015.12.001

[57] Ng SWK, Mitchell A, Kennedy JA, Chen WC, McLeod J, Ibrahimova N, et al. A 17-gene stemness score for rapid determination of risk in acute leukaemia. Nature. 2016;540:433–7. 27926740 10.1038/nature20598

[58] Elsayed AH, Rafiee R, Cao X, Raimondi S, Downing JR, Ribeiro R, et al. A six-gene leukemic stem cell score identifies high risk pediatric acute myeloid leukemia. Leukemia. 2020;34:735–45. 31645648 10.1038/s41375-019-0604-8 PMC7135934

[59] Yan F, Wong NC, Powell DR, Curtis DJ. A 9-gene score for risk stratification in B-cell acute lymphoblastic leukemia. Leukemia. 2020;34:3070–4. 32488116 10.1038/s41375-020-0888-8

[60] Liu SJ, Dang HX, Lim DA, Feng FY, Maher CA. Long noncoding RNAs in cancer metastasis. Nat Rev Cancer. 2021;21:446–60. 33953369 10.1038/s41568-021-00353-1 PMC8288800

[61] Borst L, van der Burg SH, van Hall T. The NKG2A–HLA-E Axis as a Novel Checkpoint in the Tumor Microenvironment. Clin Cancer Res. 2020;26:5549–56. 32409305 10.1158/1078-0432.CCR-19-2095

[62] Gaviraghi M, Vivori C, Pareja Sanchez Y, Invernizzi F, Cattaneo A, Santoliquido BM, et al. Tumor suppressor PNRC1 blocks rRNA maturation by recruiting the decapping complex to the nucleolus. EMBO J. 2018;37:e99179. 30373810 10.15252/embj.201899179 PMC6276881

[63] Yang J, Peng S, Zhang K. ARL4C depletion suppresses the resistance of ovarian cancer to carboplatin by disrupting cholesterol transport and autophagy via notch-RBP-Jκ-H3K4Me3-OSBPL5. Hum Exp Toxicol. 2022;41:09603271221135064. 10.1177/09603271221135064 36366750

[64] Pouyanrad S, Rahgozar S, Ghodousi ES. Dysregulation of miR-335-3p, targeted by NEAT1 and MALAT1 long non-coding RNAs, is associated with poor prognosis in childhood acute lymphoblastic leukemia. Gene. 2019;692:35–43. 30639603 10.1016/j.gene.2019.01.003

[65] Zhang W, Zhao J, Lee JF, Gartung A, Jawadi H, Lambiv WL, et al. ETS-1-mediated Transcriptional Up-regulation of CD44 Is Required for Sphingosine-1-phosphate Receptor Subtype 3-stimulated Chemotaxis. J Biol Chem. 2013;288:32126–37. 24064218 10.1074/jbc.M113.495218 PMC3820853

[66] Smith SM, Cai L. Cell Specific CD44 Expression in Breast Cancer Requires the Interaction of AP-1 and NFκB with a Novel cis-Element. PLoS One. 2012;7:e50867. 23226410 10.1371/journal.pone.0050867 PMC3511339

[67] Hao Y, Hao S, Andersen-Nissen E, Mauck WM, Zheng S, Butler A, et al. Integrated analysis of multimodal single-cell data. Cell. 2021;184:3573–87.e29. 34062119 10.1016/j.cell.2021.04.048 PMC8238499

[68] Becht E, McInnes L, Healy J, Dutertre CA, Kwok IWH, Ng LG, et al. Dimensionality reduction for visualizing single-cell data using UMAP. Nat Biotechnol. 2019;37:38–44. 10.1038/nbt.4314 30531897

[69] Benjamini Y, Hochberg Y. Controlling the False Discovery Rate: A Practical and Powerful Approach to Multiple Testing. J R Stat Soc Ser B. 1995;57:289–300.

[70] Kypraios A, Bennour J, Imbert V, David L, Calvo J, Pflumio F, et al. Identifying Candidate Gene Drivers Associated with Relapse in Pediatric T-Cell Acute Lymphoblastic Leukemia Using a Gene Co-Expression Network Approach. Cancers. 2024;16:1667. 38730619 10.3390/cancers16091667 PMC11083586

