# Supplementary material for: High CD44 expression and enhanced E-selectin binding identified as biomarkers of chemoresistant leukemic cells in human T-ALL
Source: Leukemia. 2024 Nov 24;39(2):323–36. doi: 10.1038/s41375-024-02473-7 (PMC11794132; doi:10.1038/s41375-024-02473-7)
Supplement: Supplementary file 1 — Supplemental Figures [file 41375_2024_2473_MOESM1_ESM.pptx]

## Slide 1
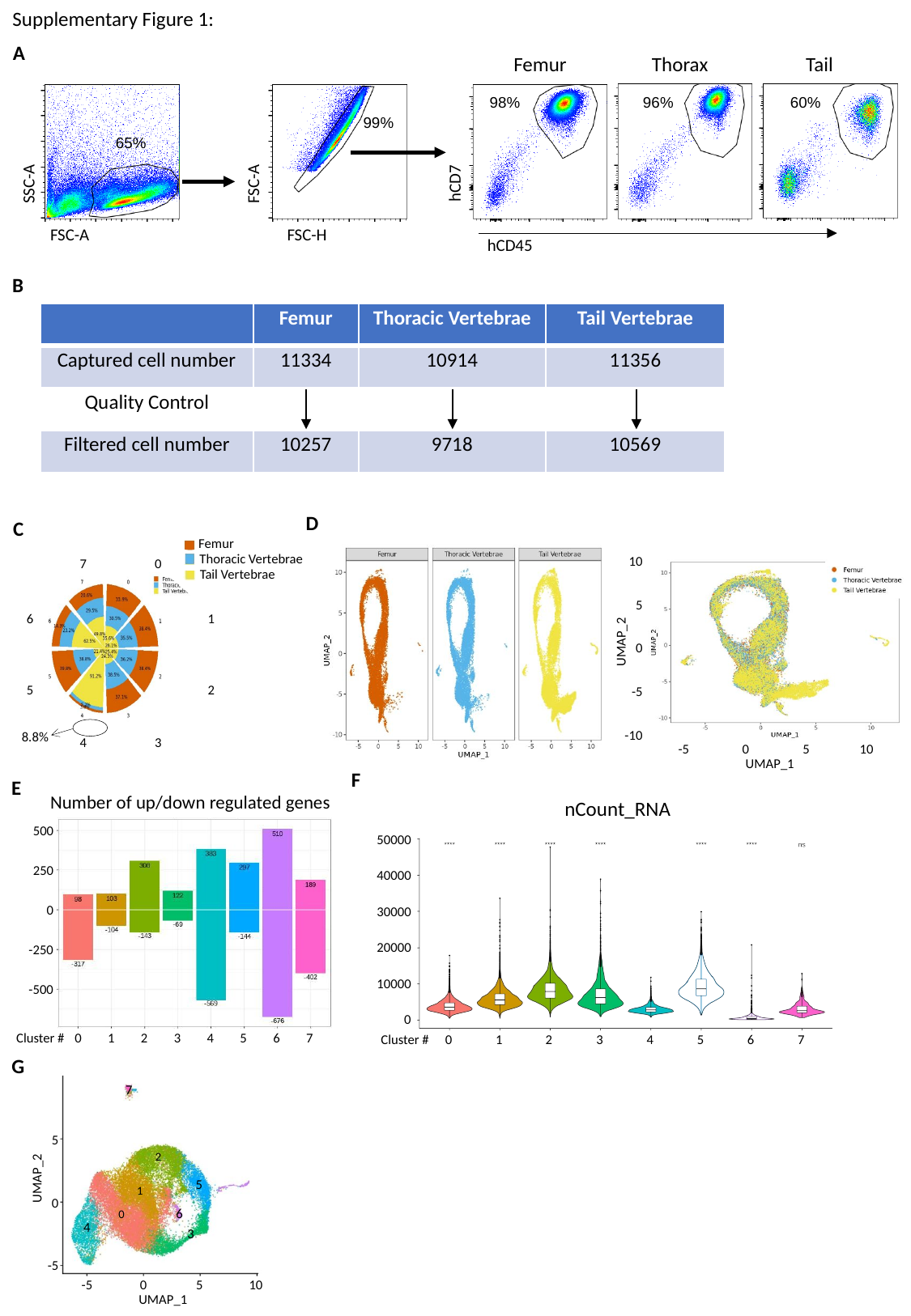

Supplementary Figure 1:
A
Femur
Thorax
Tail
98%
96%
60%
99%
65%
hCD7
FSC-A
SSC-A
FSC-A
FSC-H
hCD45
B
| | Femur | Thoracic Vertebrae | Tail Vertebrae |
| --- | --- | --- | --- |
| Captured cell number | 11334 | 10914 | 11356 |
| Quality Control | | | |
| Filtered cell number | 10257 | 9718 | 10569 |
D
C
7
0
6
1
5
2
8.8%
4
3
Femur
Thoracic Vertebrae
Tail Vertebrae
10
5
UMAP_2
0
-5
-10
-5
0
5
10
UMAP_1
F
E
Cluster #
0
1
2
3
4
5
6
7
Number of up/down regulated genes
500
250
0
-250
-500
nCount_RNA
50000
40000
30000
20000
10000
0
0
1
2
3
4
5
6
7
Cluster #
G
5
UMAP_2
0
-5
0
5
10
-5
UMAP_1
7
2
5
1
6
0
4
3

## Slide 2
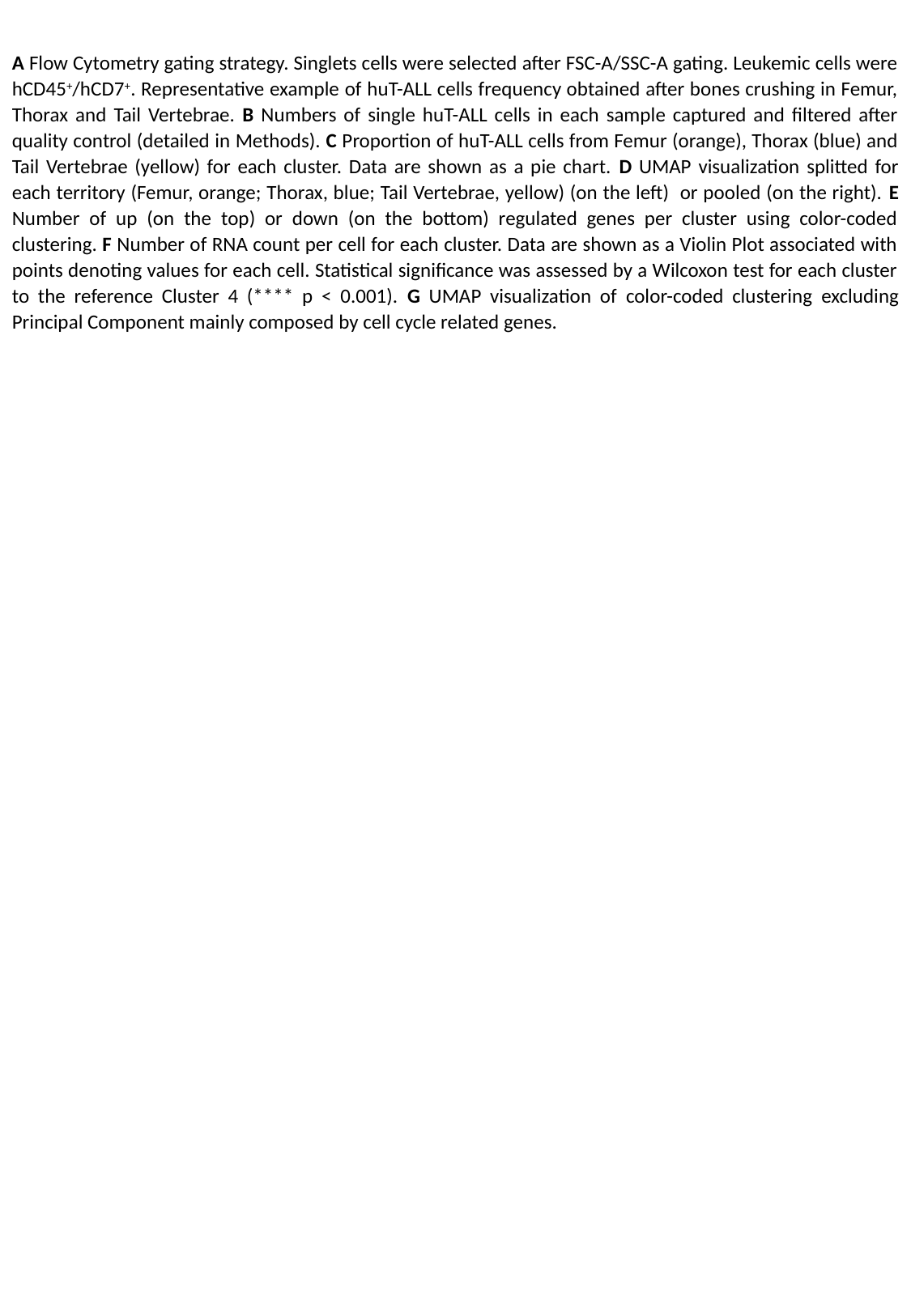

A Flow Cytometry gating strategy. Singlets cells were selected after FSC-A/SSC-A gating. Leukemic cells were hCD45+/hCD7+. Representative example of huT-ALL cells frequency obtained after bones crushing in Femur, Thorax and Tail Vertebrae. B Numbers of single huT-ALL cells in each sample captured and filtered after quality control (detailed in Methods). C Proportion of huT-ALL cells from Femur (orange), Thorax (blue) and Tail Vertebrae (yellow) for each cluster. Data are shown as a pie chart. D UMAP visualization splitted for each territory (Femur, orange; Thorax, blue; Tail Vertebrae, yellow) (on the left) or pooled (on the right). E Number of up (on the top) or down (on the bottom) regulated genes per cluster using color-coded clustering. F Number of RNA count per cell for each cluster. Data are shown as a Violin Plot associated with points denoting values for each cell. Statistical significance was assessed by a Wilcoxon test for each cluster to the reference Cluster 4 (**** p < 0.001). G UMAP visualization of color-coded clustering excluding Principal Component mainly composed by cell cycle related genes.

## Slide 3
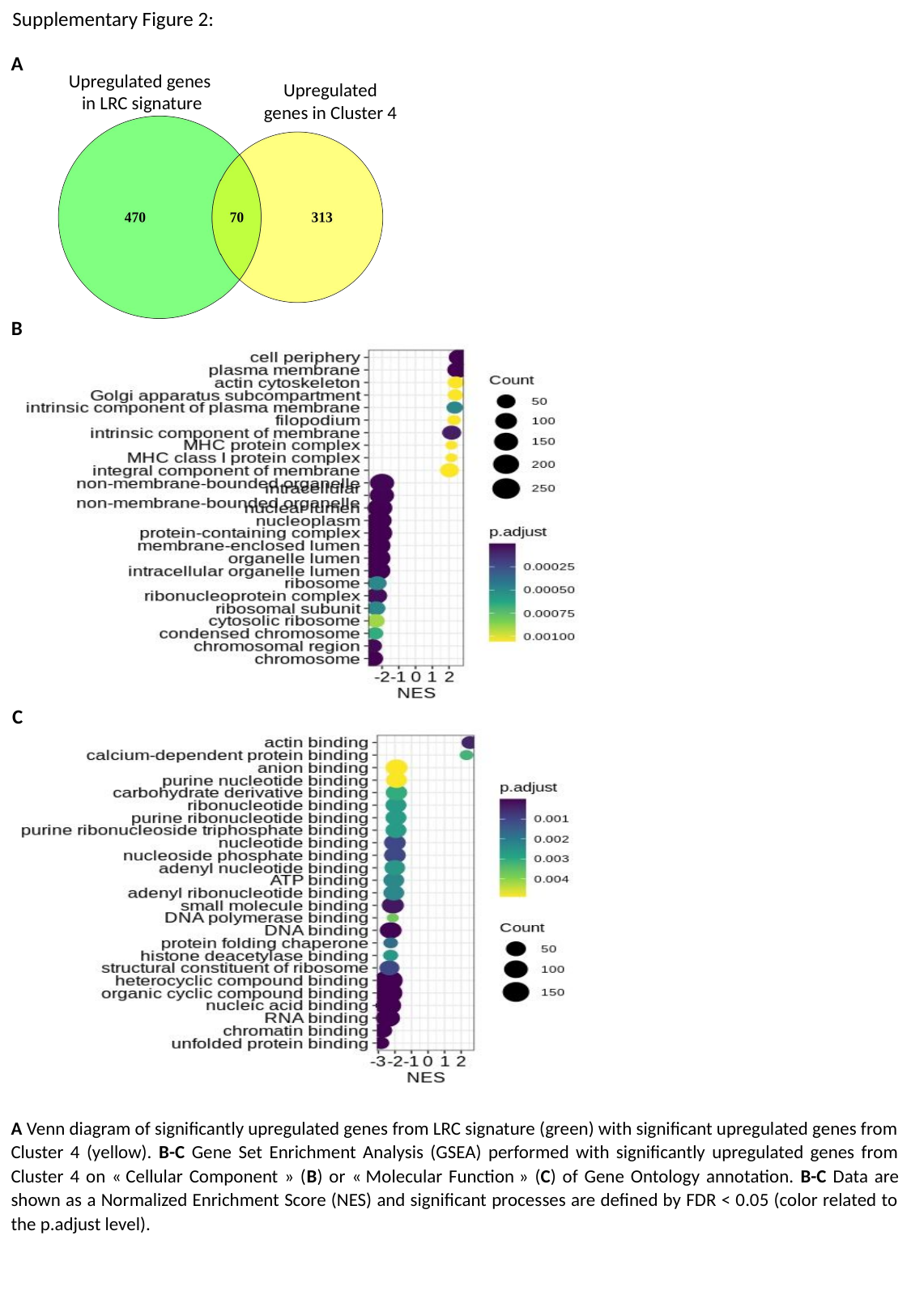

Supplementary Figure 2:
A
Upregulated genes
in LRC signature
Upregulated genes in Cluster 4
B
C
A Venn diagram of significantly upregulated genes from LRC signature (green) with significant upregulated genes from Cluster 4 (yellow). B-C Gene Set Enrichment Analysis (GSEA) performed with significantly upregulated genes from Cluster 4 on « Cellular Component » (B) or « Molecular Function » (C) of Gene Ontology annotation. B-C Data are shown as a Normalized Enrichment Score (NES) and significant processes are defined by FDR < 0.05 (color related to the p.adjust level).

## Slide 4
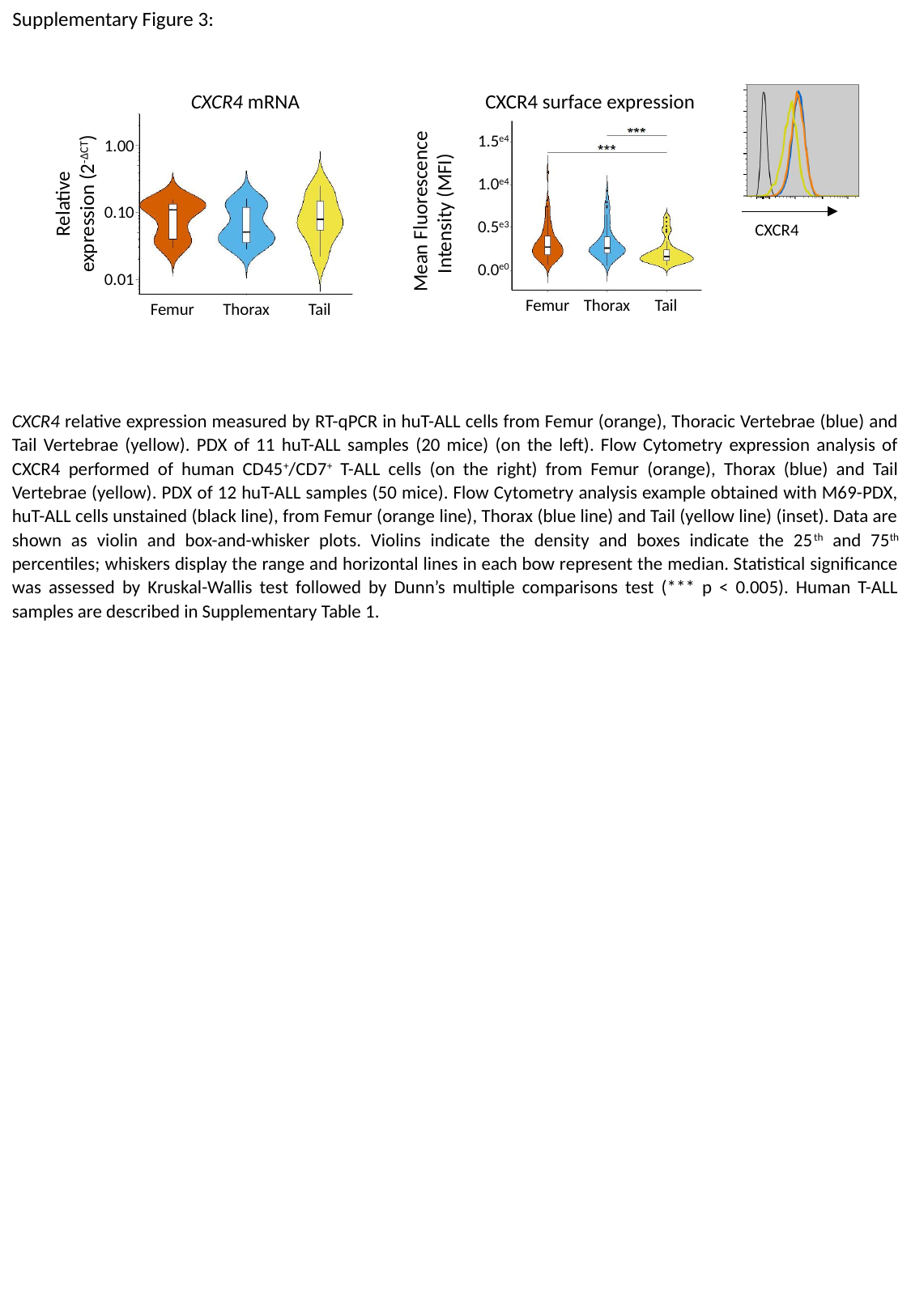

Supplementary Figure 3:
CXCR4 surface expression
Mean Fluorescence Intensity (MFI)
CXCR4
1.5e4
1.0e4
0.5e3
0.0e0
Femur
Thorax
Tail
CXCR4 mRNA
Relative expression (2-ΔCT)
1.00
0.10
0.01
Femur
Thorax
Tail
CXCR4 relative expression measured by RT-qPCR in huT-ALL cells from Femur (orange), Thoracic Vertebrae (blue) and Tail Vertebrae (yellow). PDX of 11 huT-ALL samples (20 mice) (on the left). Flow Cytometry expression analysis of CXCR4 performed of human CD45+/CD7+ T-ALL cells (on the right) from Femur (orange), Thorax (blue) and Tail Vertebrae (yellow). PDX of 12 huT-ALL samples (50 mice). Flow Cytometry analysis example obtained with M69-PDX, huT-ALL cells unstained (black line), from Femur (orange line), Thorax (blue line) and Tail (yellow line) (inset). Data are shown as violin and box-and-whisker plots. Violins indicate the density and boxes indicate the 25th and 75th percentiles; whiskers display the range and horizontal lines in each bow represent the median. Statistical significance was assessed by Kruskal-Wallis test followed by Dunn’s multiple comparisons test (*** p < 0.005). Human T-ALL samples are described in Supplementary Table 1.

## Slide 5
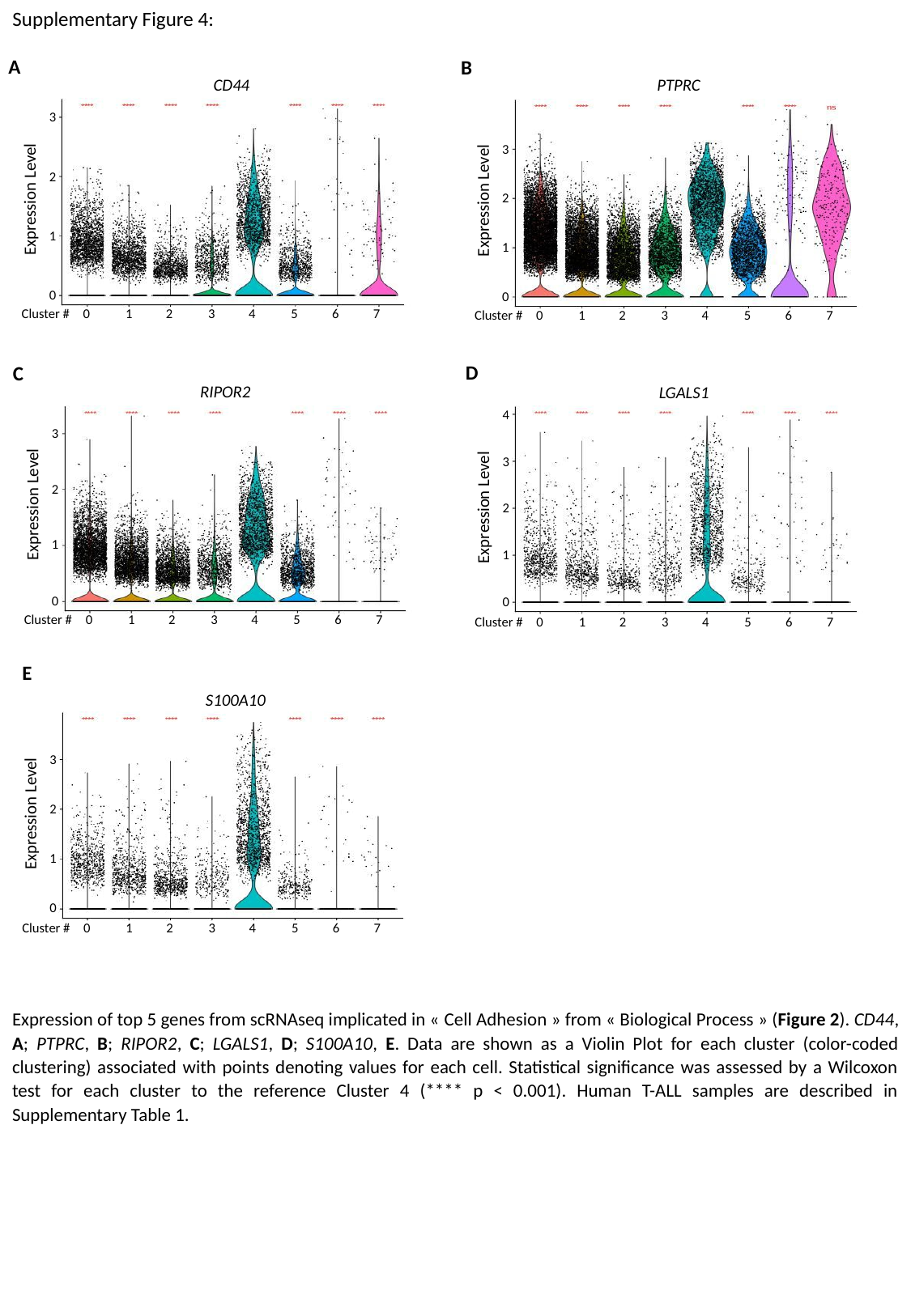

Supplementary Figure 4:
A
CD44
3
2
Expression Level
1
0
Cluster #
1
4
6
7
0
2
3
5
B
PTPRC
3
2
Expression Level
1
0
Cluster #
1
4
6
7
0
2
3
5
D
LGALS1
4
3
Expression Level
2
1
0
Cluster #
1
4
6
7
0
2
3
5
C
RIPOR2
3
2
Expression Level
1
0
Cluster #
1
4
6
7
0
2
3
5
E
S100A10
3
2
Expression Level
1
0
Cluster #
1
4
6
7
0
2
3
5
Expression of top 5 genes from scRNAseq implicated in « Cell Adhesion » from « Biological Process » (Figure 2). CD44, A; PTPRC, B; RIPOR2, C; LGALS1, D; S100A10, E. Data are shown as a Violin Plot for each cluster (color-coded clustering) associated with points denoting values for each cell. Statistical significance was assessed by a Wilcoxon test for each cluster to the reference Cluster 4 (**** p < 0.001). Human T-ALL samples are described in Supplementary Table 1.

## Slide 6
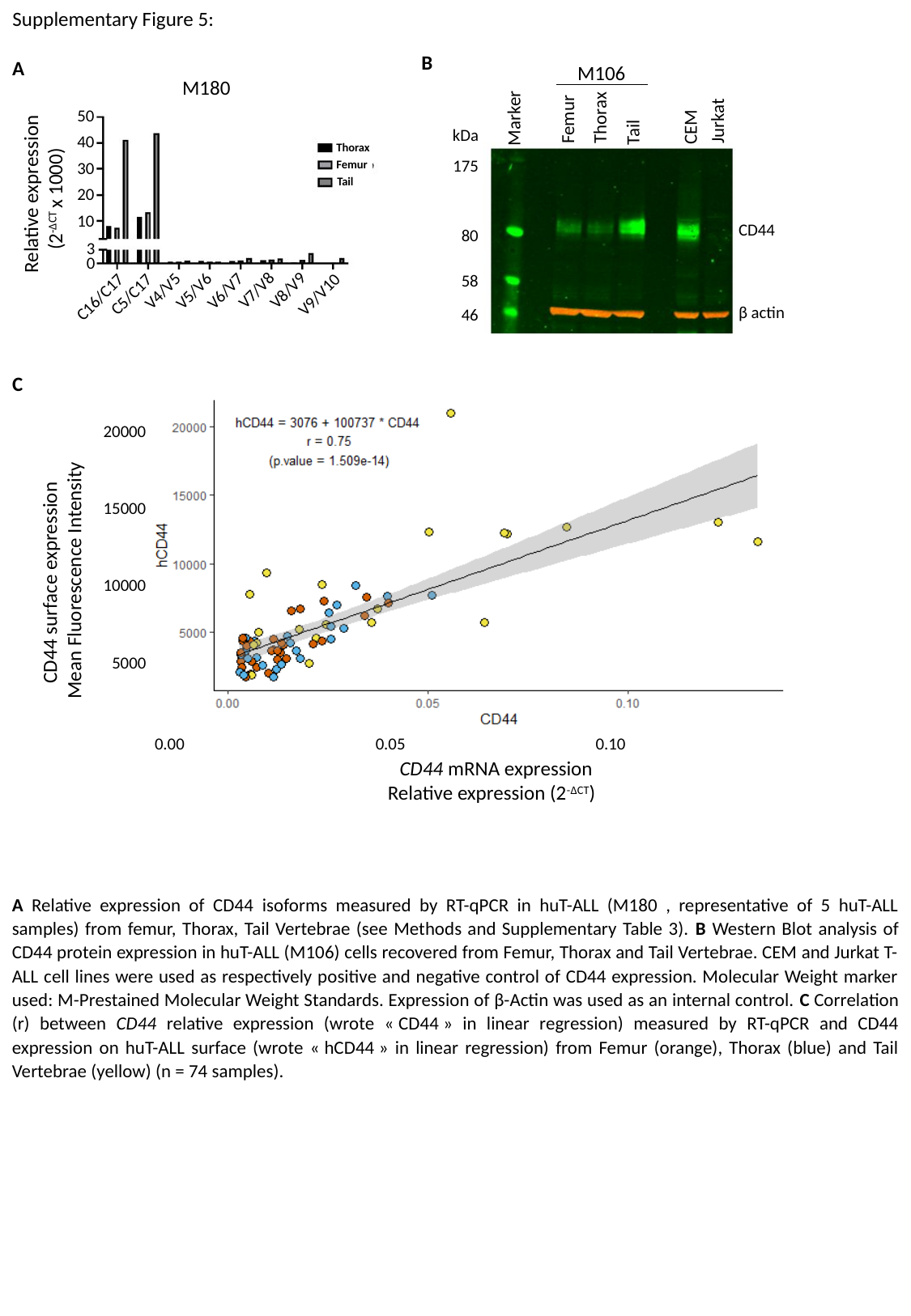

Supplementary Figure 5:
B
Thorax
Marker
Femur
Jurkat
CEM
Tail
kDa
175
80
58
46
CD44
β actin
M106
A
M180
50
40
30
Relative expression (2-ΔCT x 1000)
20
10
3
0
V7/V8
V8/V9
V4/V5
V5/V6
V6/V7
C5/C17
V9/V10
C16/C17
Thorax
Femur
Tail
C
CD44 surface expression
Mean Fluorescence Intensity
CD44 mRNA expression
Relative expression (2-ΔCT)
0.00
0.05
0.10
20000
15000
10000
5000
A Relative expression of CD44 isoforms measured by RT-qPCR in huT-ALL (M180 , representative of 5 huT-ALL samples) from femur, Thorax, Tail Vertebrae (see Methods and Supplementary Table 3). B Western Blot analysis of CD44 protein expression in huT-ALL (M106) cells recovered from Femur, Thorax and Tail Vertebrae. CEM and Jurkat T-ALL cell lines were used as respectively positive and negative control of CD44 expression. Molecular Weight marker used: M-Prestained Molecular Weight Standards. Expression of β-Actin was used as an internal control. C Correlation (r) between CD44 relative expression (wrote « CD44 » in linear regression) measured by RT-qPCR and CD44 expression on huT-ALL surface (wrote « hCD44 » in linear regression) from Femur (orange), Thorax (blue) and Tail Vertebrae (yellow) (n = 74 samples).

## Slide 7
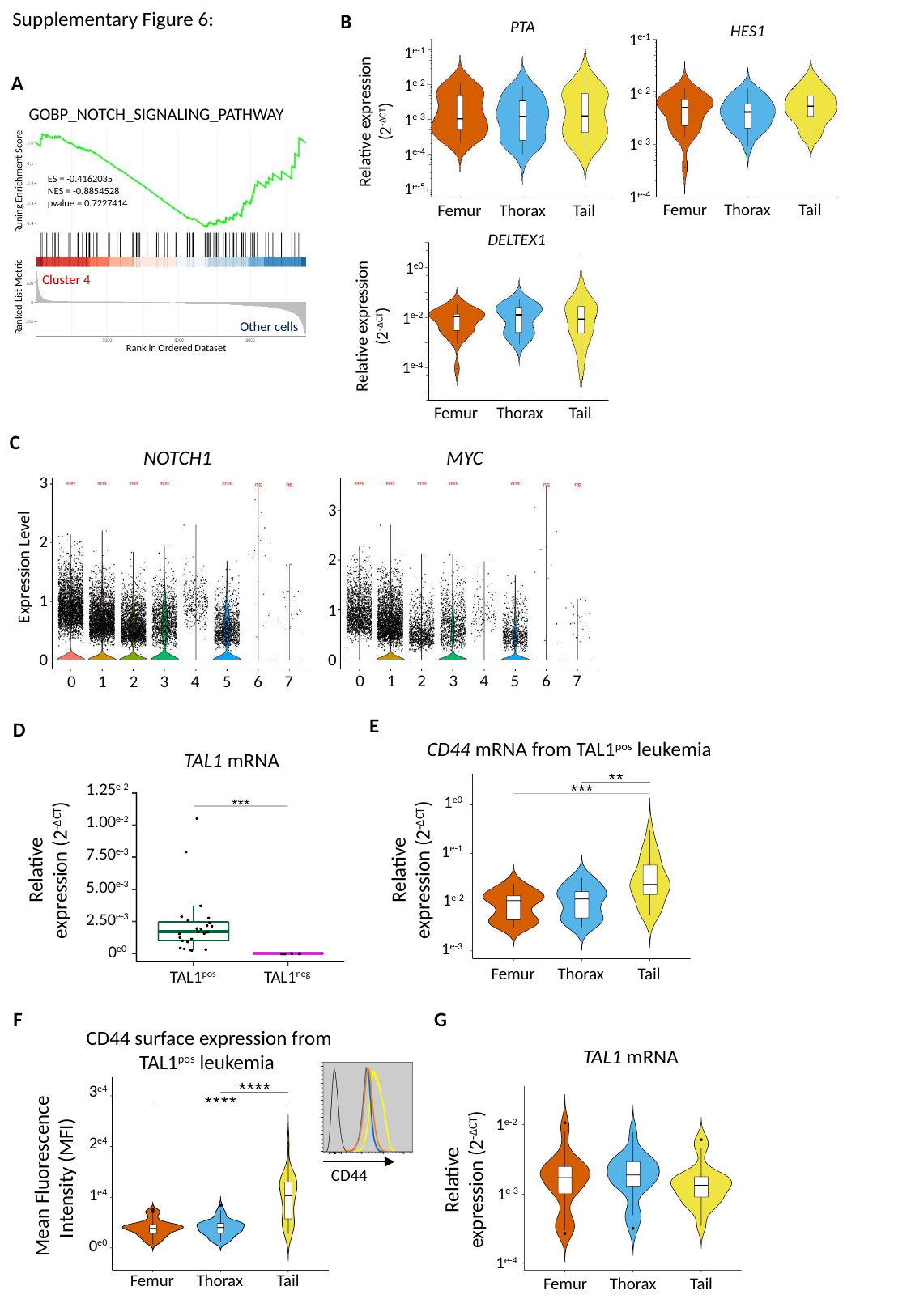

Supplementary Figure 6:
B
PTA
1e-1
1e-2
Relative expression (2-ΔCT)
1e-3
1e-4
1e-5
Femur
Thorax
Tail
HES1
1e-1
1e-2
1e-3
1e-4
Femur
Thorax
Tail
A
GOBP_NOTCH_SIGNALING_PATHWAY
Runing Enrichment Score
Ranked List Metric
Rank in Ordered Dataset
ES = -0.4162035
NES = -0.8854528
pvalue = 0.7227414
Cluster 4
Other cells
DELTEX1
1e0
1e-2
1e-4
Femur
Thorax
Tail
Relative expression (2-ΔCT)
C
NOTCH1
3
2
Expression Level
1
0
0
1
2
3
4
5
6
7
MYC
3
2
1
0
0
1
2
3
4
5
6
7
E
Femur
Thorax
Tail
1e0
1e-1
1e-2
1e-3
Relative expression (2-ΔCT)
D
TAL1 mRNA
Relative expression (2-ΔCT)
1.25e-2
1.00e-2
7.50e-3
5.00e-3
2.50e-3
0e0
TAL1pos
TAL1neg
CD44 mRNA from TAL1pos leukemia
G
1e-2
1e-3
1e-4
Femur
Thorax
Tail
Relative expression (2-ΔCT)
TAL1 mRNA
F
CD44
3e4
2e4
1e4
0e0
Femur
Thorax
Tail
Mean Fluorescence Intensity (MFI)
CD44 surface expression from TAL1pos leukemia

## Slide 8
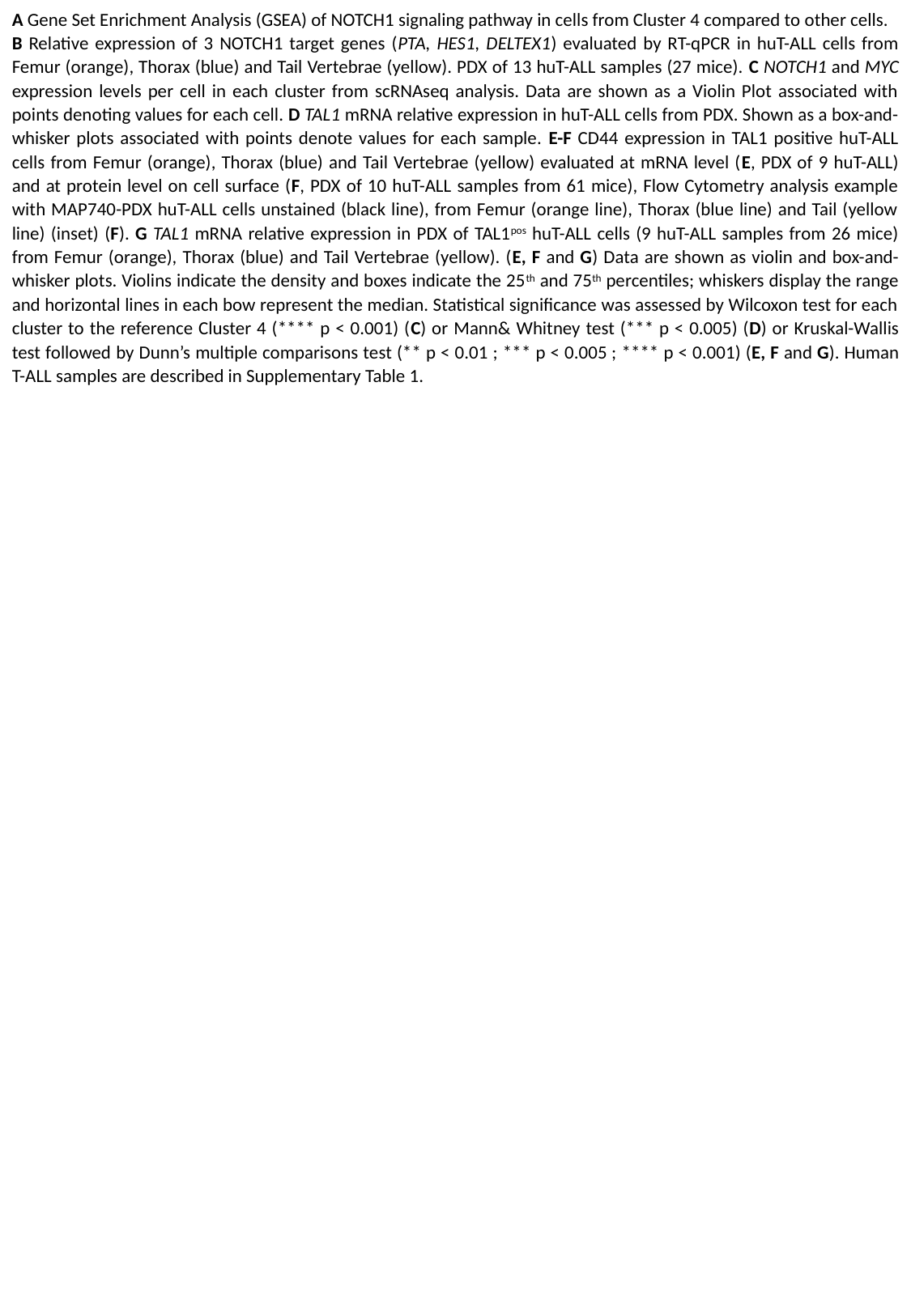

A Gene Set Enrichment Analysis (GSEA) of NOTCH1 signaling pathway in cells from Cluster 4 compared to other cells.
B Relative expression of 3 NOTCH1 target genes (PTA, HES1, DELTEX1) evaluated by RT-qPCR in huT-ALL cells from Femur (orange), Thorax (blue) and Tail Vertebrae (yellow). PDX of 13 huT-ALL samples (27 mice). C NOTCH1 and MYC expression levels per cell in each cluster from scRNAseq analysis. Data are shown as a Violin Plot associated with points denoting values for each cell. D TAL1 mRNA relative expression in huT-ALL cells from PDX. Shown as a box-and-whisker plots associated with points denote values for each sample. E-F CD44 expression in TAL1 positive huT-ALL cells from Femur (orange), Thorax (blue) and Tail Vertebrae (yellow) evaluated at mRNA level (E, PDX of 9 huT-ALL) and at protein level on cell surface (F, PDX of 10 huT-ALL samples from 61 mice), Flow Cytometry analysis example with MAP740-PDX huT-ALL cells unstained (black line), from Femur (orange line), Thorax (blue line) and Tail (yellow line) (inset) (F). G TAL1 mRNA relative expression in PDX of TAL1pos huT-ALL cells (9 huT-ALL samples from 26 mice) from Femur (orange), Thorax (blue) and Tail Vertebrae (yellow). (E, F and G) Data are shown as violin and box-and-whisker plots. Violins indicate the density and boxes indicate the 25th and 75th percentiles; whiskers display the range and horizontal lines in each bow represent the median. Statistical significance was assessed by Wilcoxon test for each cluster to the reference Cluster 4 (**** p < 0.001) (C) or Mann& Whitney test (*** p < 0.005) (D) or Kruskal-Wallis test followed by Dunn’s multiple comparisons test (** p < 0.01 ; *** p < 0.005 ; **** p < 0.001) (E, F and G). Human T-ALL samples are described in Supplementary Table 1.

## Slide 9
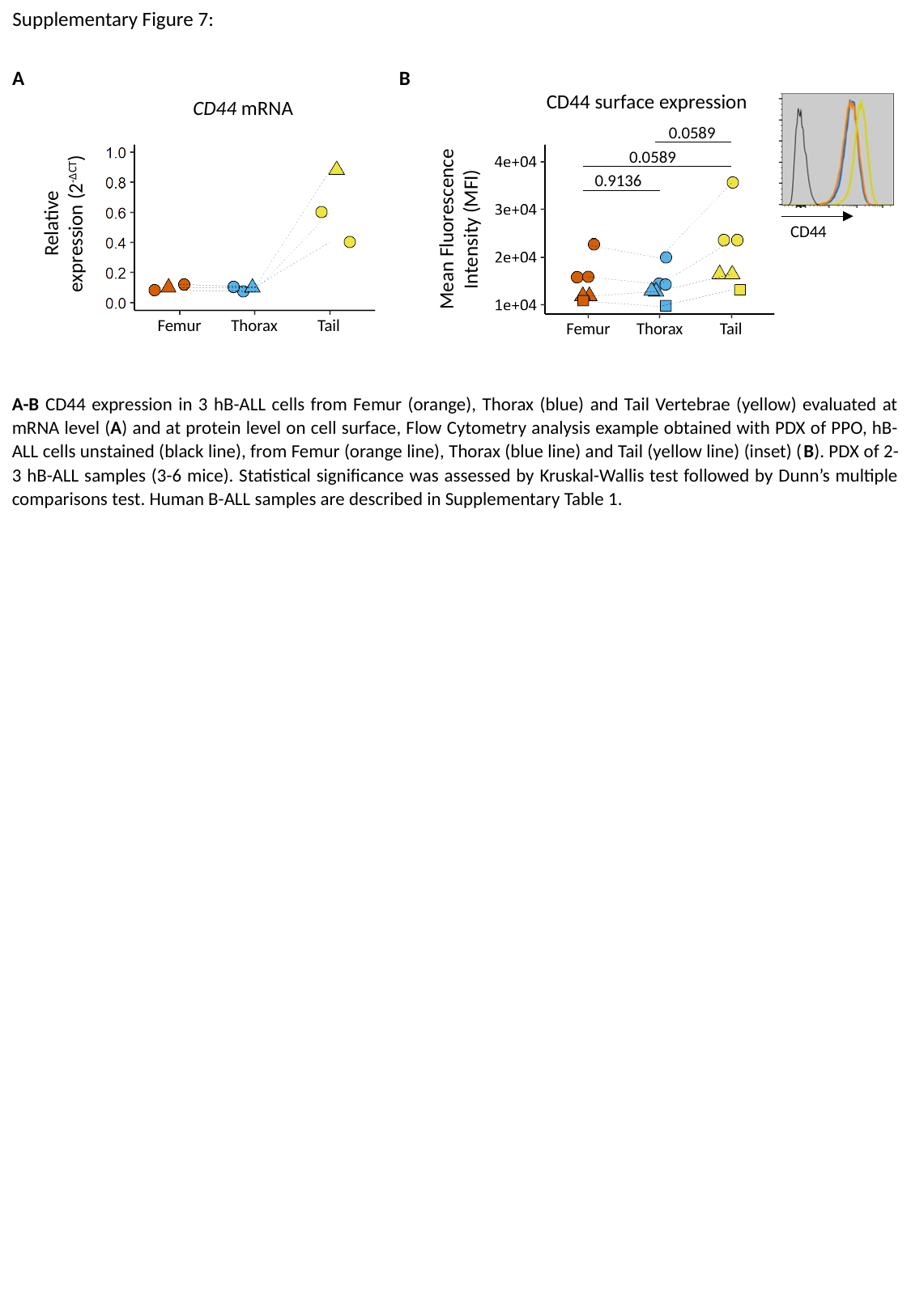

Supplementary Figure 7:
A
B
CD44 surface expression
0.0589
0.0589
0.9136
Mean Fluorescence
Intensity (MFI)
Femur
Thorax
Tail
CD44
CD44 mRNA
Relative expression (2-ΔCT)
Femur
Thorax
Tail
A-B CD44 expression in 3 hB-ALL cells from Femur (orange), Thorax (blue) and Tail Vertebrae (yellow) evaluated at mRNA level (A) and at protein level on cell surface, Flow Cytometry analysis example obtained with PDX of PPO, hB-ALL cells unstained (black line), from Femur (orange line), Thorax (blue line) and Tail (yellow line) (inset) (B). PDX of 2-3 hB-ALL samples (3-6 mice). Statistical significance was assessed by Kruskal-Wallis test followed by Dunn’s multiple comparisons test. Human B-ALL samples are described in Supplementary Table 1.

## Slide 10
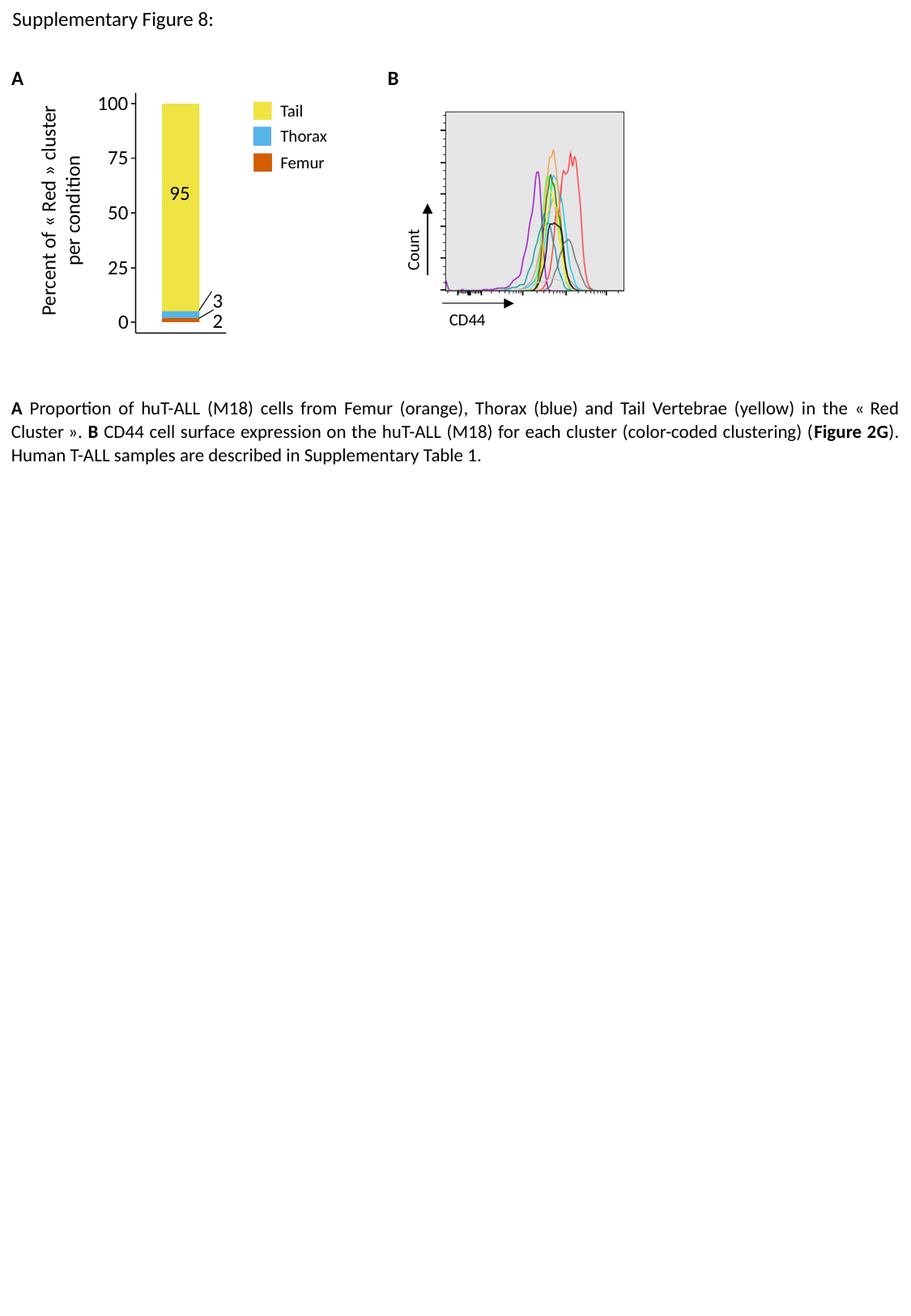

Supplementary Figure 8:
A
B
100
75
50
25
0
Tail
Thorax
Femur
95
Percent of « Red » cluster per condition
3
2
Count
CD44
A Proportion of huT-ALL (M18) cells from Femur (orange), Thorax (blue) and Tail Vertebrae (yellow) in the « Red Cluster ». B CD44 cell surface expression on the huT-ALL (M18) for each cluster (color-coded clustering) (Figure 2G). Human T-ALL samples are described in Supplementary Table 1.

## Slide 11
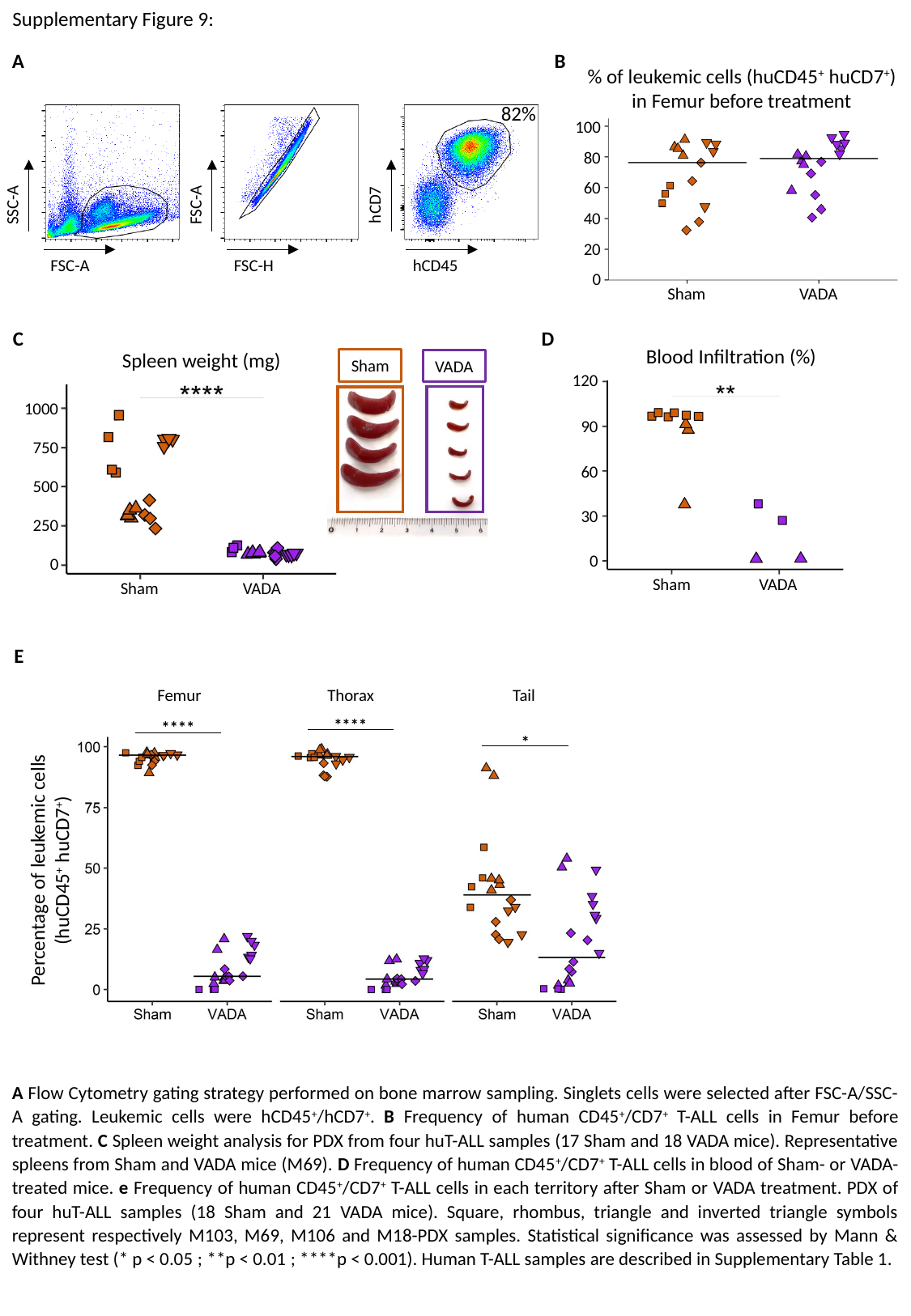

Supplementary Figure 9:
A
B
% of leukemic cells (huCD45+ huCD7+) in Femur before treatment
100
80
60
40
20
0
Sham
VADA
82%
SSC-A
FSC-A
FSC-A
FSC-H
hCD7
hCD45
C
Spleen weight (mg)
Sham
VADA
1000
750
500
250
0
Sham
VADA
D
Blood Infiltration (%)
120
90
60
30
0
Sham
VADA
E
Femur
Thorax
Tail
****
****
*
Percentage of leukemic cells (huCD45+ huCD7+)
A Flow Cytometry gating strategy performed on bone marrow sampling. Singlets cells were selected after FSC-A/SSC-A gating. Leukemic cells were hCD45+/hCD7+. B Frequency of human CD45+/CD7+ T-ALL cells in Femur before treatment. C Spleen weight analysis for PDX from four huT-ALL samples (17 Sham and 18 VADA mice). Representative spleens from Sham and VADA mice (M69). D Frequency of human CD45+/CD7+ T-ALL cells in blood of Sham- or VADA-treated mice. e Frequency of human CD45+/CD7+ T-ALL cells in each territory after Sham or VADA treatment. PDX of four huT-ALL samples (18 Sham and 21 VADA mice). Square, rhombus, triangle and inverted triangle symbols represent respectively M103, M69, M106 and M18-PDX samples. Statistical significance was assessed by Mann & Withney test (* p < 0.05 ; **p < 0.01 ; ****p < 0.001). Human T-ALL samples are described in Supplementary Table 1.

## Slide 12
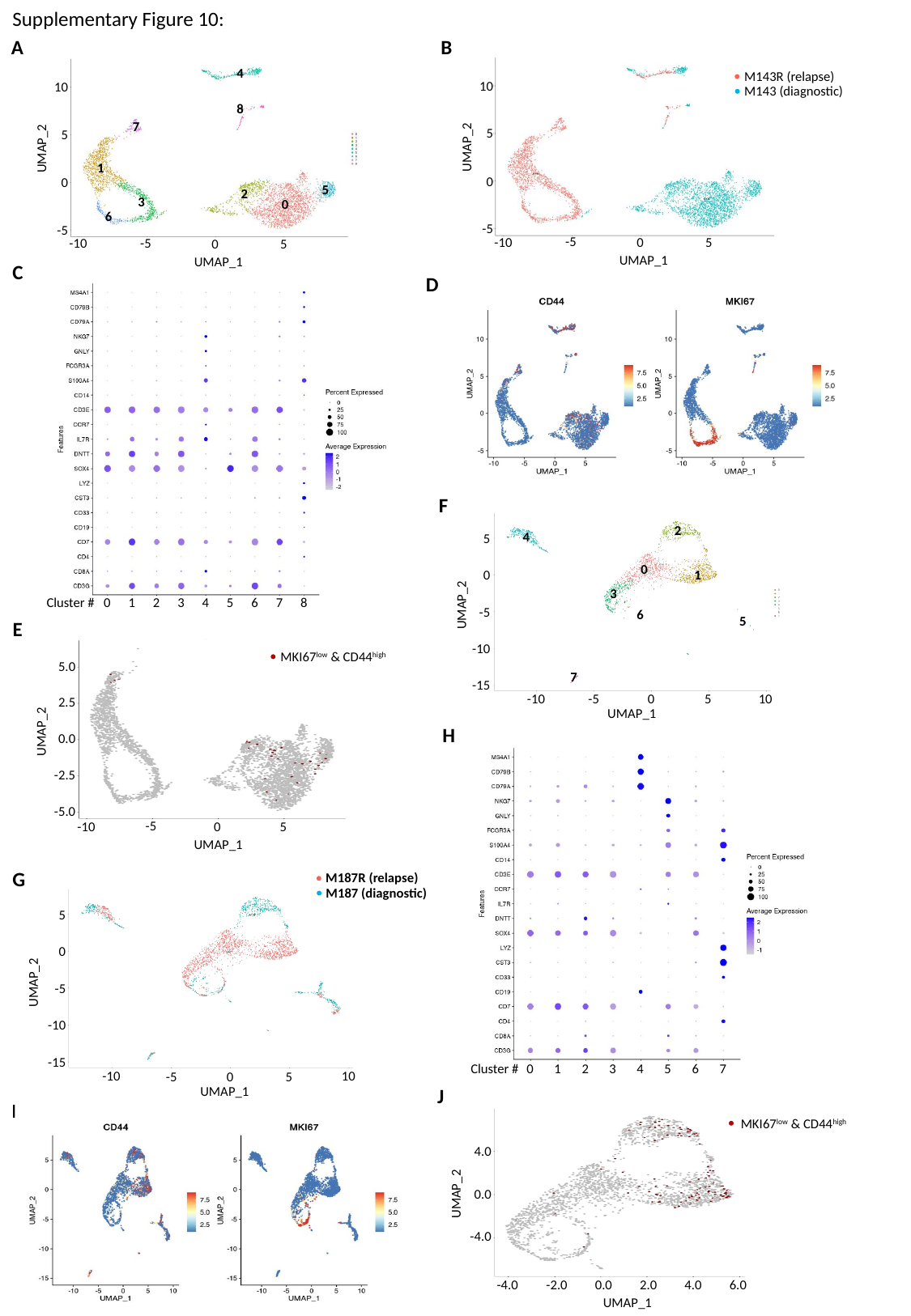

Supplementary Figure 10:
A
B
10
5
UMAP_2
0
-5
-5
-10
0
5
UMAP_1
M143R (relapse)
M143 (diagnostic)
10
5
UMAP_2
0
-5
-5
-10
0
5
UMAP_1
4
8
7
1
5
2
3
0
6
C
Cluster #
0
1
2
3
4
5
6
7
8
D
F
5
0
UMAP_2
-5
-10
-15
-10
-5
0
5
10
UMAP_1
2
4
0
1
3
6
5
E
5.0
2.5
UMAP_2
0.0
-2.5
-5.0
-5
-10
0
5
UMAP_1
MKI67low & CD44high
7
H
Cluster #
0
1
2
3
4
5
6
7
G
M187R (relapse)
M187 (diagnostic)
5
0
UMAP_2
-5
-10
-15
-10
10
-5
0
5
UMAP_1
J
4.0
0.0
UMAP_2
-4.0
-4.0
0.0
4.0
UMAP_1
-2.0
2.0
6.0
MKI67low & CD44high
I

## Slide 13
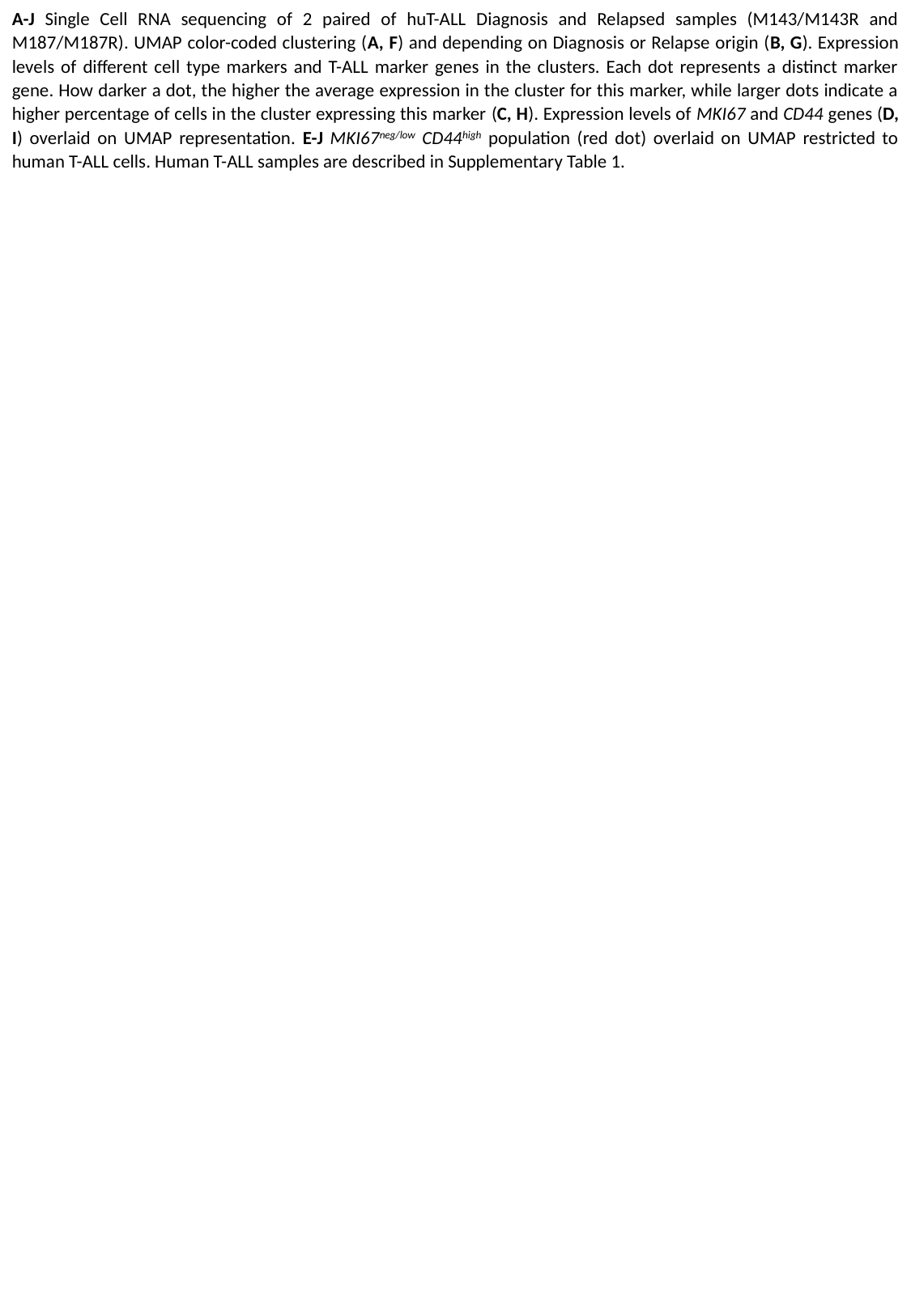

A-J Single Cell RNA sequencing of 2 paired of huT-ALL Diagnosis and Relapsed samples (M143/M143R and M187/M187R). UMAP color-coded clustering (A, F) and depending on Diagnosis or Relapse origin (B, G). Expression levels of different cell type markers and T-ALL marker genes in the clusters. Each dot represents a distinct marker gene. How darker a dot, the higher the average expression in the cluster for this marker, while larger dots indicate a higher percentage of cells in the cluster expressing this marker (C, H). Expression levels of MKI67 and CD44 genes (D, I) overlaid on UMAP representation. E-J MKI67neg/low CD44high population (red dot) overlaid on UMAP restricted to human T-ALL cells. Human T-ALL samples are described in Supplementary Table 1.

## Slide 14
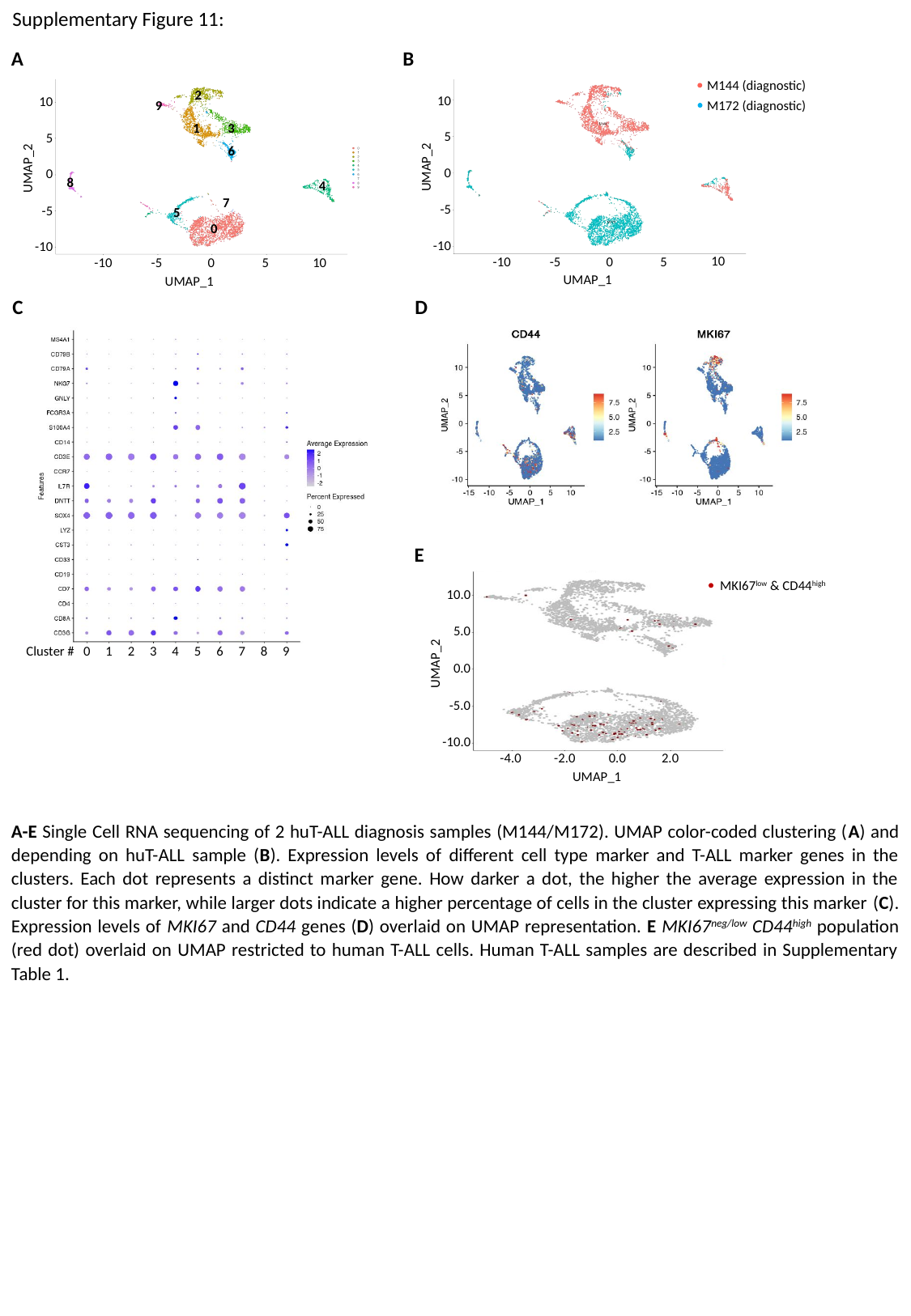

Supplementary Figure 11:
A
B
M144 (diagnostic)
M172 (diagnostic)
10
5
UMAP_2
0
-5
-10
-10
-5
0
5
UMAP_1
10
10
5
UMAP_2
0
-5
-10
-10
-5
0
5
UMAP_1
10
2
9
1
3
6
8
4
7
5
0
C
D
Cluster #
0
1
2
3
4
5
6
7
8
9
E
10.0
5.0
UMAP_2
0.0
-5.0
-10.0
-4.0
-2.0
0.0
2.0
UMAP_1
MKI67low & CD44high
A-E Single Cell RNA sequencing of 2 huT-ALL diagnosis samples (M144/M172). UMAP color-coded clustering (A) and depending on huT-ALL sample (B). Expression levels of different cell type marker and T-ALL marker genes in the clusters. Each dot represents a distinct marker gene. How darker a dot, the higher the average expression in the cluster for this marker, while larger dots indicate a higher percentage of cells in the cluster expressing this marker (C). Expression levels of MKI67 and CD44 genes (D) overlaid on UMAP representation. E MKI67neg/low CD44high population (red dot) overlaid on UMAP restricted to human T-ALL cells. Human T-ALL samples are described in Supplementary Table 1.

## Slide 15
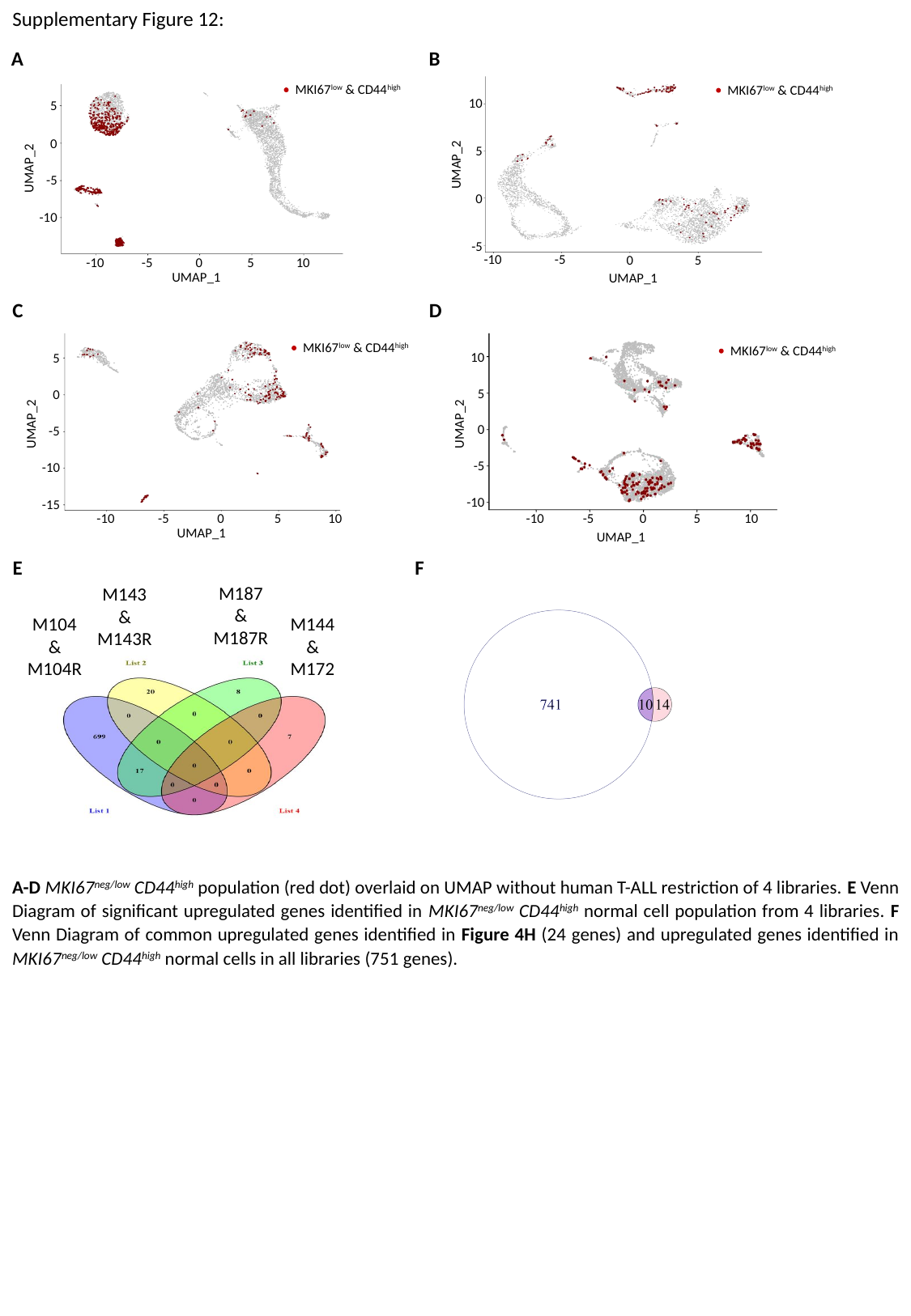

Supplementary Figure 12:
A
B
MKI67low & CD44high
5
0
UMAP_2
-5
-10
-10
-5
0
5
10
UMAP_1
MKI67low & CD44high
10
5
UMAP_2
0
-5
-5
-10
0
5
UMAP_1
C
D
MKI67low & CD44high
10
5
UMAP_2
0
-5
-10
-10
-5
0
5
UMAP_1
10
MKI67low & CD44high
5
0
UMAP_2
-5
-10
-15
-10
-5
0
5
10
UMAP_1
E
F
M187 & M187R
M143 & M143R
M144 & M172
M104 & M104R
A-D MKI67neg/low CD44high population (red dot) overlaid on UMAP without human T-ALL restriction of 4 libraries. E Venn Diagram of significant upregulated genes identified in MKI67neg/low CD44high normal cell population from 4 libraries. F Venn Diagram of common upregulated genes identified in Figure 4H (24 genes) and upregulated genes identified in MKI67neg/low CD44high normal cells in all libraries (751 genes).

## Slide 16
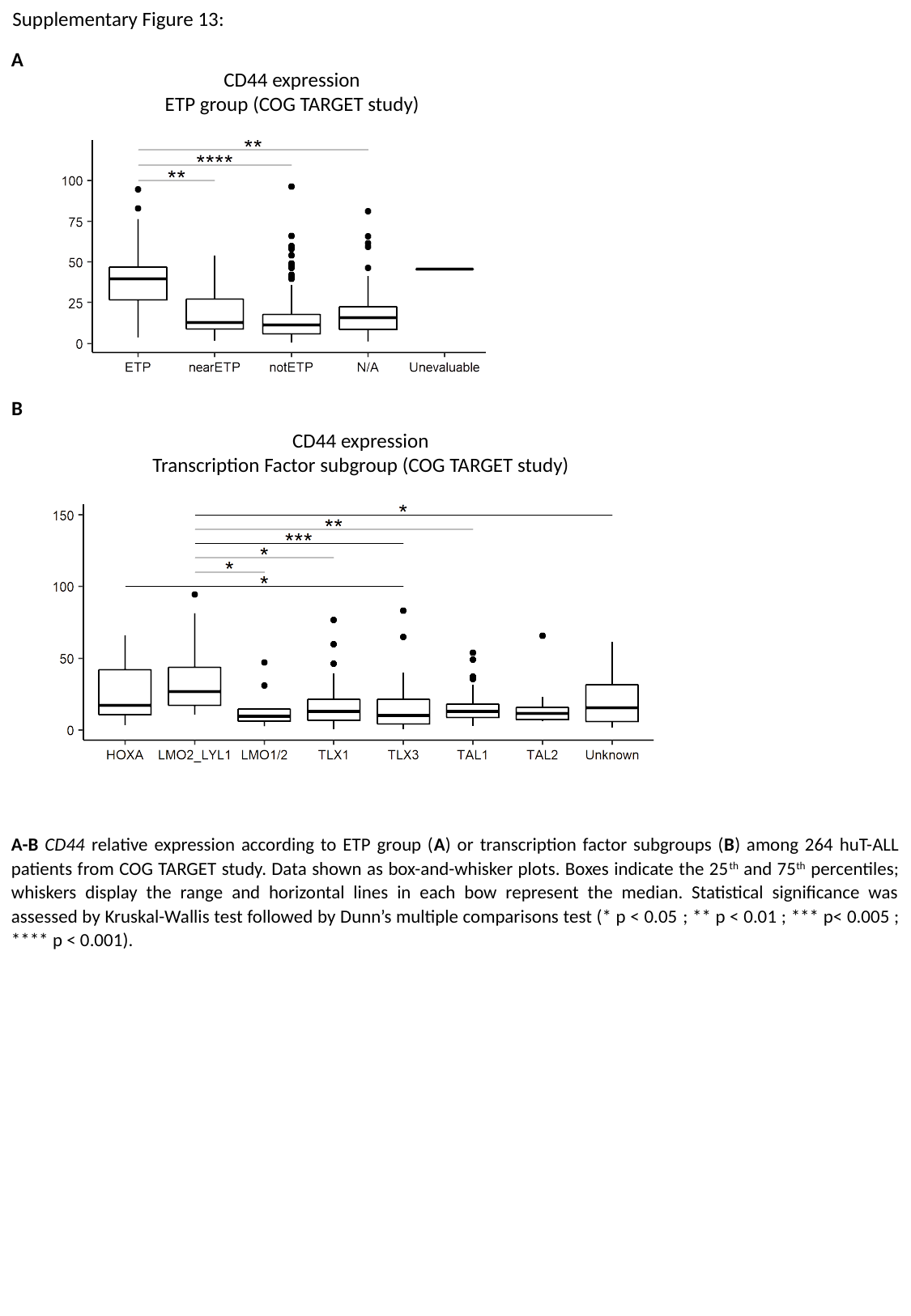

Supplementary Figure 13:
A
CD44 expression
ETP group (COG TARGET study)
B
CD44 expression
Transcription Factor subgroup (COG TARGET study)
A-B CD44 relative expression according to ETP group (A) or transcription factor subgroups (B) among 264 huT-ALL patients from COG TARGET study. Data shown as box-and-whisker plots. Boxes indicate the 25th and 75th percentiles; whiskers display the range and horizontal lines in each bow represent the median. Statistical significance was assessed by Kruskal-Wallis test followed by Dunn’s multiple comparisons test (* p < 0.05 ; ** p < 0.01 ; *** p< 0.005 ; **** p < 0.001).

## Slide 17
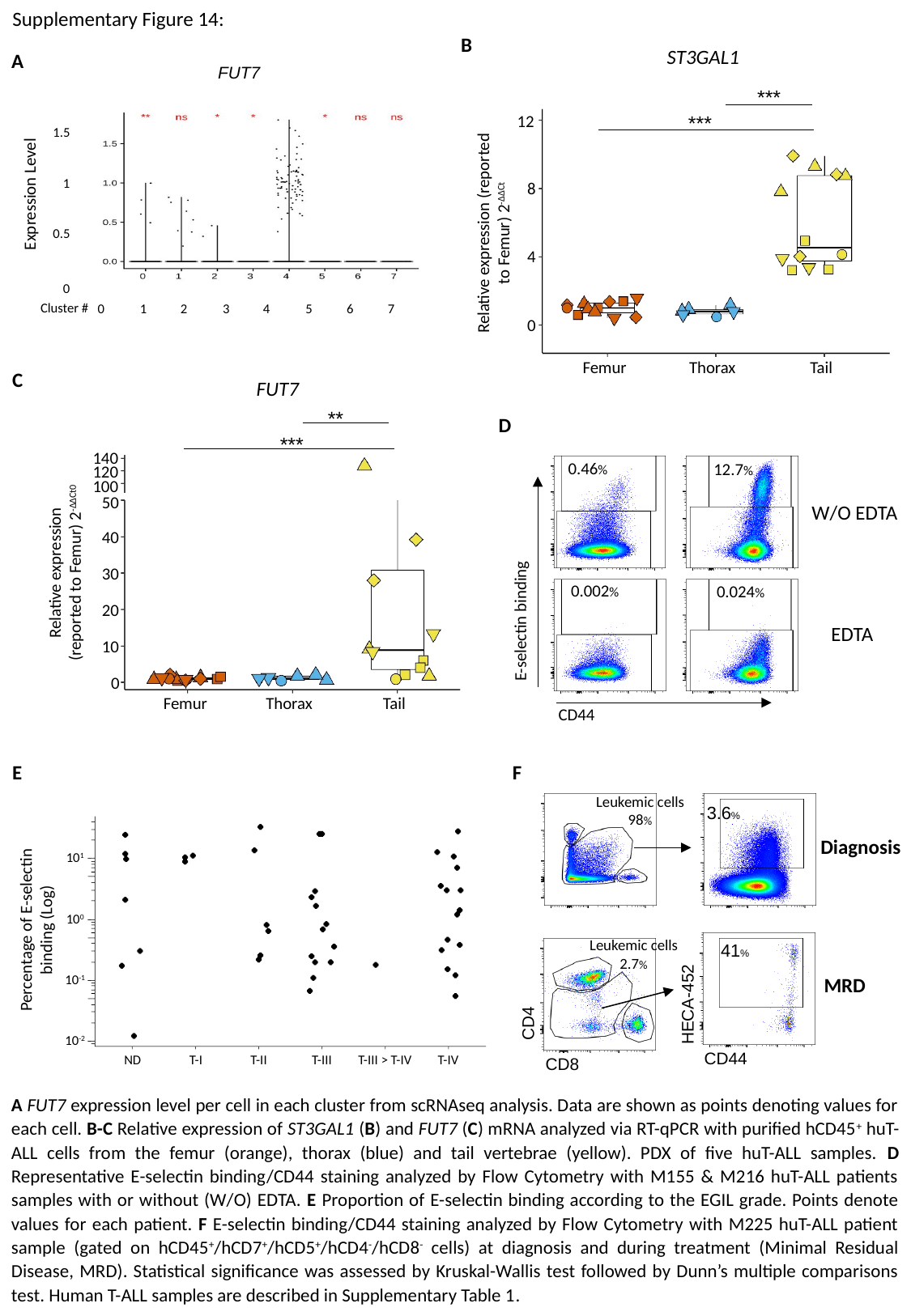

Supplementary Figure 14:
B
ST3GAL1
***
***
Femur
Thorax
Tail
12
8
4
0
Relative expression (reported to Femur) 2-ΔΔCt
A
FUT7
1.5
1
Expression Level
0
0
1
2
3
4
5
6
7
0.5
Cluster #
C
FUT7
**
***
Femur
Thorax
Tail
140
120
100
50
40
30
20
10
0
Relative expression (reported to Femur) 2-ΔΔCt0
D
0.46%
12.7%
W/O EDTA
0.002%
0.024%
E-selectin binding
EDTA
CD44
E
F
Leukemic cells
98%
101
Percentage of E-selectin binding (Log)
100
10-1
10-2
ND
T-I
T-II
T-III
T-III > T-IV
T-IV
3.6%
41%
HECA-452
CD44
Diagnosis
Leukemic cells
2.7%
MRD
CD4
CD8
A FUT7 expression level per cell in each cluster from scRNAseq analysis. Data are shown as points denoting values for each cell. B-C Relative expression of ST3GAL1 (B) and FUT7 (C) mRNA analyzed via RT-qPCR with purified hCD45+ huT-ALL cells from the femur (orange), thorax (blue) and tail vertebrae (yellow). PDX of five huT-ALL samples. D Representative E-selectin binding/CD44 staining analyzed by Flow Cytometry with M155 & M216 huT-ALL patients samples with or without (W/O) EDTA. E Proportion of E-selectin binding according to the EGIL grade. Points denote values for each patient. F E-selectin binding/CD44 staining analyzed by Flow Cytometry with M225 huT-ALL patient sample (gated on hCD45+/hCD7+/hCD5+/hCD4-/hCD8- cells) at diagnosis and during treatment (Minimal Residual Disease, MRD). Statistical significance was assessed by Kruskal-Wallis test followed by Dunn’s multiple comparisons test. Human T-ALL samples are described in Supplementary Table 1.

## Slide 18
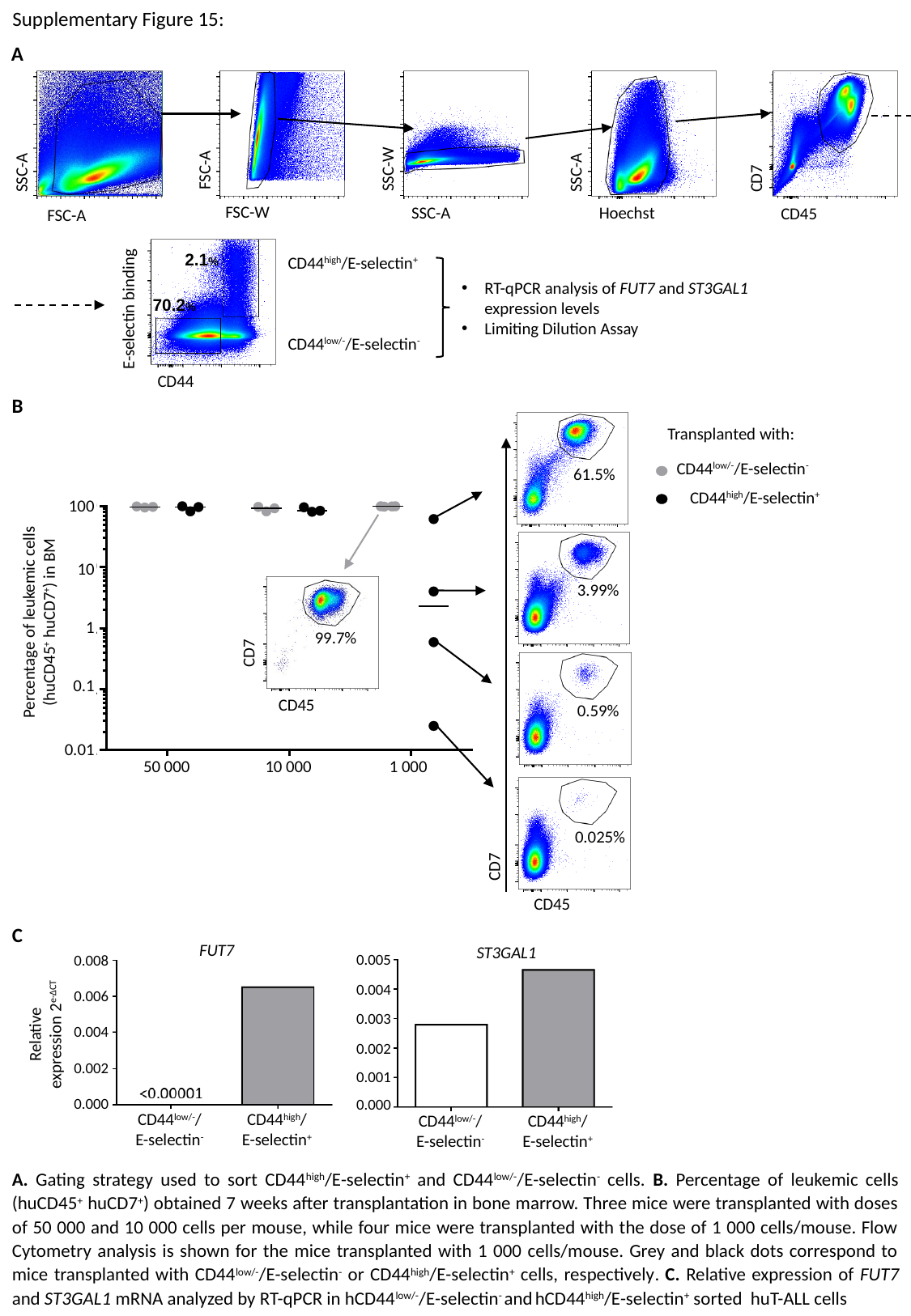

Supplementary Figure 15:
A
SSC-W
FSC-A
SSC-A
SSC-A
CD7
FSC-W
SSC-A
Hoechst
CD45
FSC-A
2.1%
70.2%
E-selectin binding
CD44
CD44high/E-selectin+
RT-qPCR analysis of FUT7 and ST3GAL1 expression levels
Limiting Dilution Assay
CD44low/-/E-selectin-
B
61.5%
3.99%
Percentage of leukemic cells (huCD45+ huCD7+) in BM
99.7%
CD7
CD45
0.59%
CD7
CD45
0.025%
Transplanted with:
CD44low/-/E-selectin-
CD44high/E-selectin+
100
10
1
0.1
0.01
50 000
10 000
1 000
C
FUT7
0.008
0.006
Relative expression 2e-ΔCT
0.004
0.002
0.000
CD44low/-/
E-selectin-
CD44high/
E-selectin+
ST3GAL1
0.005
0.004
0.003
0.002
0.001
0.000
CD44low/-/
E-selectin-
CD44high/
E-selectin+
A. Gating strategy used to sort CD44high/E-selectin+ and CD44low/-/E-selectin- cells. B. Percentage of leukemic cells (huCD45+ huCD7+) obtained 7 weeks after transplantation in bone marrow. Three mice were transplanted with doses of 50 000 and 10 000 cells per mouse, while four mice were transplanted with the dose of 1 000 cells/mouse. Flow Cytometry analysis is shown for the mice transplanted with 1 000 cells/mouse. Grey and black dots correspond to mice transplanted with CD44low/-/E-selectin- or CD44high/E-selectin+ cells, respectively. C. Relative expression of FUT7 and ST3GAL1 mRNA analyzed by RT-qPCR in hCD44low/-/E-selectin- and hCD44high/E-selectin+ sorted huT-ALL cells
