# Supplementary material for: High CD44 expression and enhanced E-selectin binding identified as biomarkers of chemoresistant leukemic cells in human T-ALL
Source: Leukemia. 2024 Nov 24;39(2):323–36. doi: 10.1038/s41375-024-02473-7 (PMC11794132; doi:10.1038/s41375-024-02473-7)
Supplement: Supplementary file 3 — Supplemental Table 2 [file 41375_2024_2473_MOESM3_ESM.pdf]

gene	p_val	avg_log2FC	pct.1	pct.2	p_val_adj	cluster
**IGFBP5**	1.32E-148	0.574863284	0.559	0.489	8.01E-144	0
**SNHG14**	0	0.530586526	0.691	0.623	0	0
**CASC15**	0	0.48220875	0.813	0.783	0	0
**LTB**	0	0.481652748	0.895	0.825	0	0
**GABPB1-AS1**	1.59E-287	0.478144085	0.741	0.688	9.67E-283	0
**KDM5B**	0	0.475081991	0.653	0.563	0	0
**GAS5**	0	0.453035568	0.835	0.829	0	0
**PCAT18**	8.83E-234	0.442366541	0.642	0.598	5.36E-229	0
**DNTT**	2.08E-245	0.440083944	0.823	0.791	1.26E-240	0
**PRMT7**	2.53E-137	0.438941496	0.625	0.603	1.54E-132	0
**CD7**	0	0.430155935	0.929	0.927	0	0
**MZB1**	0	0.425914522	0.94	0.91	0	0
**MXD4**	5.09E-201	0.42482854	0.47	0.375	3.08E-196	0
**MDM4**	2.76E-177	0.4172862	0.629	0.604	1.67E-172	0
**GIHCG**	5.37E-241	0.415325503	0.772	0.754	3.26E-236	0
**ARPP21**	5.72E-110	0.407065403	0.485	0.448	3.47E-105	0
**PNISR**	0	0.403672057	0.882	0.883	0	0
**RCBTB2**	1.22E-295	0.402481618	0.905	0.901	7.39E-291	0
**PNRC1**	1.71E-227	0.393975525	0.616	0.547	1.04E-222	0
**TRGC2**	1.49E-112	0.374767929	0.788	0.803	9.06E-108	0
**MCM4**	0	0.733777698	0.705	0.274	0	1
**MCM3**	0	0.7295957	0.775	0.361	0	1
**HELLS**	0	0.720785492	0.783	0.373	0	1
**GINS2**	0	0.682534769	0.637	0.209	0	1
**DUT**	0	0.674791821	0.905	0.632	0	1
**PCNA**	0	0.664595154	0.79	0.387	0	1
**MCM5**	0	0.66147187	0.685	0.271	0	1
**NASP**	0	0.650226492	0.932	0.631	0	1
**MCM6**	0	0.636927018	0.663	0.263	0	1
**MSH6**	0	0.578295148	0.681	0.329	0	1
**MCM7**	0	0.564749859	0.769	0.423	0	1
**DNMT1**	0	0.547685365	0.832	0.506	0	1
**UHRF1**	0	0.539191102	0.636	0.317	0	1
**CDCA7**	0	0.538458211	0.691	0.37	0	1
**TYMS**	0	0.498312182	0.877	0.491	0	1
**CLSPN**	0	0.48721343	0.625	0.303	0	1
**PAICS**	0	0.483799349	0.712	0.413	0	1
**UNG**	0	0.483348313	0.465	0.124	0	1
**HSP90AB1**	0	0.475976201	0.988	0.883	0	1
**HSPD1**	0	0.475725392	0.869	0.641	0	1
**H4C3**	0	2.370371465	0.999	0.687	0	2
**H1-5**	0	1.991481149	0.98	0.187	0	2
**TUBA1B**	0	1.632143468	0.999	0.753	0	2
**TUBB**	0	1.503033862	0.999	0.834	0	2
**RRM2**	0	1.416356145	0.96	0.212	0	2
**H1-3**	0	1.400235802	0.998	0.749	0	2

**H1-2**	0	1.33845476	0.978	0.519	0	2
**TYMS**	0	1.336255419	0.992	0.512	0	2
**MKI67**	0	1.204883697	0.983	0.385	0	2
**H2AZ1**	0	1.199992282	1	0.785	0	2
**H2AC20**	0	1.187966815	0.893	0.27	0	2
**PCLAF**	0	1.158309927	0.982	0.477	0	2
**HMGB2**	0	1.126510631	0.999	0.578	0	2
**DUT**	0	1.039328707	0.986	0.647	0	2
**H2AX**	0	0.958382166	0.941	0.323	0	2
**TOP2A**	0	0.92866172	0.942	0.261	0	2
**ATAD2**	0	0.916607393	0.905	0.332	0	2
**HMGN2**	0	0.892491622	1	0.844	0	2
**HMGB1**	0	0.882526275	1	0.965	0	2
**PCNA**	0	0.873353001	0.923	0.406	0	2
**HMGN2**	0	0.912023729	0.996	0.855	0	3
**PTTG1**	0	0.889057261	0.754	0.299	0	3
**NUCKS1**	0	0.793420703	0.991	0.856	0	3
**HDGF**	0	0.674996103	0.851	0.544	0	3
**CENPF**	0	0.669740223	0.784	0.4	0	3
**UBE2S**	0	0.659303837	0.8	0.517	0	3
**ARL6IP1**	4.04E-242	0.651684854	0.599	0.336	2.45E-237	3
**HMGB1**	0	0.647696151	1	0.967	0	3
**HNRNPM**	0	0.64338802	0.954	0.738	0	3
**HNRNPA2B1**	0	0.638529348	0.995	0.946	0	3
**CCNB1**	0	0.634233191	0.515	0.178	0	3
**HMGB2**	1.09E-263	0.630731274	0.884	0.62	6.63E-259	3
**ASPM**	3.40E-265	0.615143299	0.588	0.294	2.06E-260	3
**TMSB4X**	0	0.606126328	1	0.998	0	3
**PTMS**	0	0.59931261	0.51	0.135	0	3
**PRR11**	0	0.598515679	0.651	0.27	0	3
**HMMR**	0	0.558436105	0.472	0.161	0	3
**BIRC5**	0	0.54961589	0.621	0.256	0	3
**CDC20**	0	0.532797657	0.493	0.157	0	3
**LDHA**	5.60E-255	0.528149461	0.967	0.84	3.40E-250	3
**LTB**	0	1.953117948	0.982	0.835	0	4
**CD52**	0	1.934309577	0.87	0.449	0	4
**S100A4**	0	1.920559655	0.533	0.096	0	4
**KLF2**	0	1.847348569	0.657	0.052	0	4
**S100A10**	0	1.810572783	0.546	0.094	0	4
**EMP3**	0	1.803552583	0.865	0.368	0	4
**LGALS1**	0	1.711056746	0.431	0.086	0	4
**SH3BGRL3**	0	1.618161238	0.978	0.896	0	4
**TXNIP**	0	1.503755719	0.814	0.443	0	4
**SH3BP5**	0	1.426446171	0.681	0.296	0	4
**PTPRC**	0	1.304066295	0.933	0.752	0	4
**KLF6**	0	1.302693468	0.625	0.362	0	4
**MALAT1**	0	1.275141549	1	0.998	0	4

**ARL4C**	0	1.268672507	0.942	0.784	0	4
**TMSB10**	0	1.206173349	0.997	0.983	0	4
**KLRB1**	0	1.200760661	0.328	0.018	0	4
**RIPOR2**	0	1.191882457	0.708	0.388	0	4
**CD44**	0	1.18424078	0.613	0.233	0	4
**HCST**	0	1.119118603	0.781	0.536	0	4
**TRG-AS1**	0	1.10969395	0.801	0.593	0	4
**TOP2A**	0	2.517064627	0.999	0.317	0	5
**CENPF**	0	2.43924992	0.998	0.39	0	5
**UBE2C**	0	2.220233355	0.993	0.215	0	5
**MKI67**	0	2.181015086	1	0.437	0	5
**ASPM**	0	2.058492335	0.992	0.267	0	5
**HMGB2**	0	2.0086564	1	0.616	0	5
**H4C3**	0	1.883094835	0.993	0.716	0	5
**TUBB4B**	0	1.820009843	0.991	0.345	0	5
**KPNA2**	0	1.746204986	0.97	0.293	0	5
**UBE2S**	0	1.717298356	0.994	0.507	0	5
**ARL6IP1**	0	1.709938285	0.968	0.311	0	5
**TPX2**	0	1.674870064	0.989	0.345	0	5
**TUBA1B**	0	1.620528097	1	0.775	0	5
**PTTG1**	0	1.608264286	0.981	0.289	0	5
**TUBA1C**	0	1.556553059	0.953	0.295	0	5
**CCNB1**	0	1.545017151	0.935	0.151	0	5
**GTSE1**	0	1.544963635	0.982	0.266	0	5
**TUBB**	0	1.459421288	1	0.849	0	5
**CENPE**	0	1.451325877	0.941	0.151	0	5
**DLGAP5**	0	1.425444896	0.935	0.138	0	5
**S100A6**	1.89E-87	5.935506253	0.315	0.075	1.15E-82	6
**FOS**	1.35E-38	3.965906675	0.375	0.189	8.19E-34	6
**ACTBP2**	0	3.700275231	0.368	0.001	0	6
**RPS27AP16**	2.87E-104	3.572853625	0.382	0.097	1.74E-99	6
**FAUP1**	0	3.534155295	0.368	0.013	0	6
**H2AW**	8.56E-24	3.381578219	0.315	0.179	5.19E-19	6
**APOE**	8.03E-268	3.190634883	0.11	0.002	4.87E-263	6
**AC016739.2**	7.74E-96	2.569830859	0.236	0.04	4.70E-91	6
**RPL17P34**	0	2.533669243	0.239	0.003	0	6
**HPR**	0	2.38829077	0.167	0	0	6
**RPS3AP26**	0	2.370214916	0.246	0.003	0	6
**JUNB**	1.36E-08	2.256818653	0.181	0.111	0.0008258	6
**RP3-486I3.4**	0	2.180710102	0.215	0.005	0	6
**S100A11**	4.80E-10	2.164417938	0.181	0.104	2.91E-05	6
**LGALS3**	0	2.099234164	0.169	0.001	0	6
**RPL27AP5**	0	1.962198006	0.212	0.001	0	6
**RPSAP19**	0	1.950733236	0.198	0.001	0	6
**FTH1P2**	0	1.921311535	0.196	0.001	0	6
**RP1-278E11.3**	0	1.892712533	0.193	0.007	0	6
**RPS24P19**	4.46E-87	1.83527312	0.193	0.029	2.71E-82	6

**S100A9**	0	3.457173782	0.477	0.004	0	7
**CEBPE**	0	2.626256988	0.879	0.044	0	7
**GLUL**	1.25E-94	1.81445479	0.893	0.645	7.57E-90	7
**RAP1B**	5.82E-117	1.805787606	0.943	0.702	3.53E-112	7
**MAFB**	0	1.768847144	0.562	0.034	0	7
**SERPINB1**	2.48E-52	1.6895328	0.815	0.729	1.51E-47	7
**RAB27A**	7.13E-190	1.559520525	0.63	0.115	4.32E-185	7
**CST7**	1.20E-168	1.494111373	0.456	0.063	7.30E-164	7
**PLIN2**	1.44E-70	1.493909448	0.765	0.458	8.76E-66	7
**FTH1**	1.38E-119	1.39652086	0.996	0.991	8.34E-115	7
**PLEK**	0	1.395178927	0.552	0.018	0	7
**LINC02384**	4.55E-107	1.349995248	0.637	0.196	2.76E-102	7
**MIR503HG**	8.86E-110	1.287565052	0.552	0.141	5.37E-105	7
**TRDC**	9.52E-75	1.276073068	0.925	0.781	5.78E-70	7
**RFLNB**	6.72E-63	1.263566845	0.776	0.521	4.07E-58	7
**PTPRC**	5.13E-69	1.208210561	0.932	0.766	3.11E-64	7
**CREG1**	1.93E-191	1.194262283	0.477	0.062	1.17E-186	7
**C14orf119**	2.54E-54	1.16511959	0.52	0.217	1.54E-49	7
**VAT1**	6.38E-57	1.158638624	0.619	0.303	3.87E-52	7
**CFLAR**	7.43E-41	1.130601571	0.587	0.334	4.51E-36	7
