# Supplementary material for: High CD44 expression and enhanced E-selectin binding identified as biomarkers of chemoresistant leukemic cells in human T-ALL
Source: Leukemia. 2024 Nov 24;39(2):323–36. doi: 10.1038/s41375-024-02473-7 (PMC11794132; doi:10.1038/s41375-024-02473-7)
Supplement: Supplementary file 4 — Supplemental Table 3 [file 41375_2024_2473_MOESM4_ESM.pdf]

gene	p_val	avg_log2FC	pct.1	pct.2	p_val_adj	cluster
LTB	0.00E+00	1.953117948	0.982	0.835	0.00E+00	4
CD52	0.00E+00	1.934309577	0.87	0.449	0.00E+00	4
S100A4	0.00E+00	1.920559655	0.533	0.096	0.00E+00	4
KLF2	0.00E+00	1.847348569	0.657	0.052	0.00E+00	4
S100A10	0.00E+00	1.810572783	0.546	0.094	0.00E+00	4
EMP3	0.00E+00	1.803552583	0.865	0.368	0.00E+00	4
LGALS1	0.00E+00	1.711056746	0.431	0.086	0.00E+00	4
SH3BGRL3	0.00E+00	1.618161238	0.978	0.896	0.00E+00	4
TXNIP	0.00E+00	1.503755719	0.814	0.443	0.00E+00	4
SH3BP5	0.00E+00	1.426446171	0.681	0.296	0.00E+00	4
PTPRC	0.00E+00	1.304066295	0.933	0.752	0.00E+00	4
KLF6	0.00E+00	1.302693468	0.625	0.362	0.00E+00	4
MALAT1	0.00E+00	1.275141549	1	0.998	0.00E+00	4
ARL4C	0.00E+00	1.268672507	0.942	0.784	0.00E+00	4
TMSB10	0.00E+00	1.206173349	0.997	0.983	0.00E+00	4
KLRB1	0.00E+00	1.200760661	0.328	0.018	0.00E+00	4
RIPOR2	0.00E+00	1.191882457	0.708	0.388	0.00E+00	4
CD44	0.00E+00	1.18424078	0.613	0.233	0.00E+00	4
HCST	0.00E+00	1.119118603	0.781	0.536	0.00E+00	4
TRG-AS1	0.00E+00	1.10969395	0.801	0.593	0.00E+00	4
FOS	0.00E+00	1.057225141	0.492	0.165	0.00E+00	4
MIAT	0.00E+00	1.055253614	0.449	0.075	0.00E+00	4
HLA-E	0.00E+00	1.029195823	0.836	0.671	0.00E+00	4
NEAT1	3.62E-298	0.963525583	0.86	0.792	2.20E-293	4
PI16	0.00E+00	0.954822803	0.412	0.047	0.00E+00	4
CALM1	1.56E-265	0.938235327	0.697	0.573	9.47E-261	4
TPST2	0.00E+00	0.931447578	0.492	0.196	0.00E+00	4
B2M	0.00E+00	0.89699686	1	0.991	0.00E+00	4
FLNA	6.34E-240	0.895542109	0.58	0.401	3.85E-235	4
STK4	3.06E-283	0.89296564	0.618	0.414	1.86E-278	4
CRIP1	0.00E+00	0.891132899	0.31	0.062	0.00E+00	4
CIRBP	0.00E+00	0.854006664	0.865	0.763	0.00E+00	4
DOK2	0.00E+00	0.834103622	0.463	0.191	0.00E+00	4
ACAP1	0.00E+00	0.819216065	0.812	0.713	0.00E+00	4
VIM	1.93E-270	0.808154679	0.956	0.946	1.17E-265	4
GZMM	7.47E-240	0.807457188	0.638	0.465	4.53E-235	4
PCSK7	7.27E-204	0.804756759	0.637	0.501	4.41E-199	4
AHNAK	0.00E+00	0.797587456	0.266	0.01	0.00E+00	4
PPP1R15A	7.61E-209	0.792834592	0.462	0.255	4.62E-204	4
ANXA1	7.35E-164	0.789279283	0.564	0.424	4.46E-159	4
PCDH10	1.41E-195	0.782848263	0.558	0.382	8.53E-191	4
MBP	1.22E-204	0.773500434	0.583	0.418	7.39E-200	4
ATM	6.40E-217	0.772902386	0.701	0.593	3.88E-212	4
PNRC1	4.19E-252	0.771112893	0.709	0.556	2.54E-247	4
UCP2	2.86E-168	0.749316125	0.616	0.517	1.73E-163	4
LIMD2	3.11E-282	0.735201927	0.834	0.767	1.88E-277	4
HLA-B	1.30E-284	0.725983098	0.93	0.891	7.86E-280	4
CD27	0.00E+00	0.721669608	0.357	0.091	0.00E+00	4
ETS1	6.18E-174	0.712737521	0.622	0.517	3.75E-169	4
MTURN	3.79E-214	0.700819965	0.451	0.234	2.30E-209	4
GPSM3	3.21E-185	0.688090631	0.642	0.536	1.95E-180	4
CNN2	5.73E-148	0.687827927	0.547	0.427	3.47E-143	4
TLE5	8.30E-255	0.68597908	0.849	0.826	5.03E-250	4
N4BP2L2	4.24E-219	0.685255462	0.784	0.727	2.57E-214	4

**PGM2L1**	6.27E-135	0.682821809	0.467	0.316	3.80E-130	4
**FOSB**	1.39E-212	0.679468622	0.276	0.092	8.45E-208	4
**STK17B**	1.26E-154	0.677159133	0.669	0.604	7.67E-150	4
**TSC22D3**	0.00E+00	0.674278175	0.294	0.051	0.00E+00	4
**IL32**	7.56E-172	0.669595751	0.905	0.899	4.59E-167	4
**CDKN2D**	1.86E-180	0.668846518	0.644	0.503	1.13E-175	4
**HLA-A**	7.97E-163	0.660261729	0.754	0.713	4.83E-158	4
**GABPB1-AS1**	3.71E-143	0.652612546	0.749	0.701	2.25E-138	4
**AHI1**	2.91E-115	0.644069548	0.595	0.53	1.77E-110	4
**TRDC**	2.52E-178	0.637726546	0.825	0.779	1.53E-173	4
**TAGLN2**	3.27E-97	0.634426751	0.643	0.628	1.98E-92	4
**CDC42**	1.72E-186	0.625137554	0.759	0.735	1.05E-181	4
**JUND**	4.63E-171	0.623960003	0.83	0.817	2.81E-166	4
**KLF13**	4.01E-145	0.622873407	0.515	0.374	2.43E-140	4
**RSRP1**	1.13E-135	0.615588918	0.566	0.459	6.88E-131	4
**RCBTB2**	3.72E-239	0.609546382	0.93	0.9	2.25E-234	4
**MYO1G**	5.63E-152	0.608026804	0.706	0.653	3.41E-147	4
**TUBA1A**	5.63E-117	0.602912809	0.739	0.674	3.42E-112	4
**DUSP1**	7.97E-166	0.600826316	0.287	0.118	4.83E-161	4
**LAPTM5**	1.02E-169	0.596220283	0.777	0.752	6.21E-165	4
**LST1**	3.30E-109	0.591971371	0.69	0.687	2.00E-104	4
**CASP8**	4.77E-124	0.588686929	0.41	0.264	2.90E-119	4
**CLEC2D**	2.61E-127	0.586830231	0.392	0.237	1.59E-122	4
**TRAF3IP3**	7.97E-128	0.583423061	0.452	0.303	4.83E-123	4
**CD37**	1.21E-135	0.581895848	0.619	0.545	7.33E-131	4
**SCN3A**	2.10E-111	0.579199854	0.423	0.282	1.27E-106	4
**PRKCB**	5.42E-110	0.577390038	0.588	0.527	3.28E-105	4
**RORB**	3.42E-127	0.576871069	0.285	0.133	2.07E-122	4
**FMNL1**	1.37E-91	0.574254812	0.492	0.411	8.32E-87	4
**SF1**	7.63E-173	0.570598142	0.803	0.788	4.63E-168	4
**MED13L**	2.35E-123	0.570354787	0.692	0.657	1.42E-118	4
**RAP1B**	3.57E-163	0.562581322	0.739	0.701	2.16E-158	4
**FNBP1**	3.86E-141	0.557813164	0.738	0.728	2.34E-136	4
**MSN**	4.49E-166	0.557152175	0.827	0.83	2.72E-161	4
**GLG1**	2.35E-89	0.550664907	0.478	0.387	1.43E-84	4
**MDM4**	3.81E-111	0.546276612	0.655	0.608	2.31E-106	4
**ISG20**	2.05E-170	0.546144611	0.291	0.12	1.25E-165	4
**SKAP1**	5.77E-90	0.539385486	0.409	0.295	3.50E-85	4
**ARAP2**	1.16E-112	0.53848213	0.324	0.181	7.04E-108	4
**EVL**	7.31E-173	0.535410253	0.869	0.853	4.43E-168	4
**ANTKMT**	2.73E-75	0.532722901	0.589	0.538	1.66E-70	4
**YWHAZ**	1.29E-174	0.532370203	0.846	0.859	7.82E-170	4
**RAP2B**	1.02E-80	0.528107453	0.361	0.253	6.21E-76	4
**EZR**	1.93E-56	0.52432929	0.593	0.616	1.17E-51	4
**CD84**	1.23E-112	0.523106948	0.744	0.733	7.47E-108	4
**RAB37**	5.76E-92	0.523063933	0.443	0.328	3.49E-87	4
**PLAC8**	6.48E-279	0.522761299	0.191	0.037	3.93E-274	4
**TIMP1**	0.00E+00	0.521760102	0.218	0.024	0.00E+00	4
**ITM2B**	1.11E-156	0.519158153	0.844	0.856	6.74E-152	4
**SARAF**	4.88E-104	0.518630611	0.585	0.527	2.96E-99	4
**RGCC**	8.17E-86	0.516965741	0.356	0.236	4.96E-81	4
**ANXA2**	1.98E-50	0.516953087	0.389	0.331	1.20E-45	4
**MYL12A**	1.52E-135	0.51389287	0.798	0.792	9.24E-131	4
**HLA-C**	4.83E-114	0.511836109	0.787	0.783	2.93E-109	4
**IKZF1**	1.33E-102	0.509213265	0.683	0.67	8.05E-98	4

**GUK1**	1.66E-153	0.50813735	0.87	0.867	1.01E-148	4
**FGR**	2.95E-253	0.50695104	0.266	0.074	1.79E-248	4
**PNISR**	1.75E-159	0.50371067	0.882	0.883	1.06E-154	4
**FTX**	1.35E-80	0.500894349	0.496	0.413	8.17E-76	4
**SBK1**	6.93E-103	0.500593693	0.334	0.199	4.20E-98	4
**IFITM2**	2.31E-68	0.489030958	0.345	0.244	1.40E-63	4
**RP5-1171I10.!**	9.39E-60	0.486094899	0.435	0.357	5.70E-55	4
**HDAC7**	8.49E-65	0.485161566	0.457	0.392	5.15E-60	4
**CD48**	1.05E-66	0.483466629	0.513	0.471	6.37E-62	4
**ELOVL4**	7.47E-89	0.47935068	0.28	0.155	4.53E-84	4
**TRAC**	9.57E-69	0.478888005	0.585	0.549	5.80E-64	4
**MYADM**	1.10E-184	0.478601097	0.214	0.064	6.66E-180	4
**ANKRD44**	1.77E-66	0.475965859	0.356	0.259	1.07E-61	4
**AAK1**	7.64E-67	0.47380011	0.351	0.253	4.63E-62	4
**BTG1**	1.86E-134	0.470030239	0.797	0.796	1.13E-129	4
**DMBT1**	8.68E-90	0.465998646	0.25	0.128	5.26E-85	4
**KDM5B**	7.92E-71	0.464671681	0.615	0.589	4.80E-66	4
**BIN1**	3.68E-109	0.464211138	0.285	0.147	2.23E-104	4
**BTG2**	5.95E-99	0.463853654	0.439	0.304	3.61E-94	4
**DDX17**	4.96E-129	0.463826246	0.851	0.865	3.01E-124	4
**TRIB2**	9.13E-127	0.461260298	0.253	0.11	5.54E-122	4
**LPAR6**	3.01E-58	0.456966593	0.525	0.482	1.83E-53	4
**AKAP13**	5.39E-61	0.455539438	0.434	0.365	3.27E-56	4
**ABLIM1**	5.33E-53	0.45479132	0.357	0.273	3.23E-48	4
**CCDC18-AS1**	5.71E-169	0.454396536	0.253	0.09	3.46E-164	4
**ZBTB7A**	5.65E-45	0.452973398	0.406	0.359	3.43E-40	4
**LRRFIP1**	1.97E-72	0.452271025	0.732	0.768	1.19E-67	4
**CORO1A**	7.41E-97	0.451723899	0.75	0.754	4.49E-92	4
**ZNF683**	0.00E+00	0.45138075	0.156	0.006	0.00E+00	4
**UBC**	4.56E-93	0.448944069	0.857	0.871	2.76E-88	4
**YPEL5**	7.13E-64	0.44863083	0.339	0.241	4.32E-59	4
**EIF1**	2.99E-243	0.446855822	0.989	0.985	1.82E-238	4
**TBC1D10C**	7.18E-50	0.443811473	0.487	0.467	4.35E-45	4
**PTGER4**	1.32E-53	0.441477844	0.362	0.283	8.01E-49	4
**KAT6B**	2.00E-46	0.438830622	0.492	0.471	1.22E-41	4
**TNRC6B**	3.70E-52	0.435988937	0.57	0.575	2.24E-47	4
**GCC2**	1.72E-35	0.433428116	0.463	0.456	1.04E-30	4
**ICAM3**	3.25E-60	0.428577569	0.598	0.621	1.97E-55	4
**SP110**	7.66E-36	0.421797463	0.38	0.343	4.64E-31	4
**RHOH**	5.47E-68	0.420743099	0.641	0.641	3.32E-63	4
**ORMDL1**	9.59E-45	0.420155425	0.466	0.446	5.82E-40	4
**MT-CO1**	4.44E-213	0.415654359	1	0.991	2.70E-208	4
**MHENCR**	2.31E-93	0.415121513	0.237	0.115	1.40E-88	4
**ADGRG1**	1.40E-56	0.413600136	0.298	0.205	8.51E-52	4
**CD53**	5.11E-45	0.412111001	0.461	0.432	3.10E-40	4
**ADD3**	0.00E+00	0.409715209	0.161	0.022	0.00E+00	4
**ZFP36L2**	8.32E-34	0.409062798	0.636	0.691	5.05E-29	4
**NINJ1**	1.73E-84	0.40397793	0.251	0.133	1.05E-79	4
**TCF7L2**	2.96E-32	0.401236936	0.38	0.339	1.79E-27	4
**S1PR1**	1.14E-51	0.400757004	0.289	0.199	6.90E-47	4
**ANKRD12**	2.45E-26	0.398265093	0.435	0.436	1.48E-21	4
**TNRC6C**	5.33E-34	0.398008752	0.511	0.525	3.23E-29	4
**PGGHG**	3.64E-56	0.39750589	0.315	0.222	2.21E-51	4
**PSMA3-AS1**	4.38E-44	0.397414619	0.487	0.47	2.66E-39	4
**ETFDH**	2.99E-34	0.396274918	0.324	0.267	1.82E-29	4

**MYH9**	5.57E-38	0.394573434	0.465	0.457	3.38E-33	4
**PLEKHF1**	1.96E-41	0.392741529	0.322	0.255	1.19E-36	4
**PLEC**	2.41E-91	0.392631393	0.253	0.128	1.46E-86	4
**ATP2B1-AS1**	8.75E-106	0.391076343	0.202	0.083	5.31E-101	4
**NHLRC3**	7.11E-36	0.390855186	0.337	0.279	4.31E-31	4
**AKNA**	1.30E-29	0.390437116	0.356	0.32	7.86E-25	4
**VAMP2**	6.64E-41	0.389662791	0.427	0.39	4.03E-36	4
**C16orf54**	1.08E-67	0.389581214	0.252	0.147	6.57E-63	4
**RNF125**	3.42E-157	0.389030071	0.195	0.06	2.08E-152	4
**PRR7**	4.56E-39	0.388011252	0.288	0.218	2.76E-34	4
**CCNL1**	4.02E-43	0.386798264	0.568	0.593	2.44E-38	4
**STK10**	9.73E-53	0.38671684	0.289	0.2	5.90E-48	4
**ARHGEF1**	1.53E-35	0.385968482	0.316	0.259	9.29E-31	4
**PTPN7**	3.72E-42	0.384625316	0.622	0.664	2.26E-37	4
**LINC-PINT**	5.59E-189	0.382705213	0.196	0.053	3.39E-184	4
**PRMT7**	2.53E-26	0.381192337	0.574	0.613	1.54E-21	4
**CST7**	4.47E-157	0.379982931	0.188	0.056	2.71E-152	4
**VCL**	9.79E-207	0.379840712	0.165	0.036	5.94E-202	4
**MECP2**	3.05E-25	0.379477471	0.346	0.319	1.85E-20	4
**LAT**	2.05E-23	0.376012349	0.385	0.372	1.24E-18	4
**POU2F2**	1.14E-93	0.375443049	0.197	0.083	6.92E-89	4
**MIR181A1HG**	2.37E-38	0.375177264	0.587	0.606	1.43E-33	4
**ARHGAP30**	7.14E-31	0.374942644	0.468	0.476	4.33E-26	4
**SPTBN1**	2.84E-46	0.372943775	0.73	0.784	1.72E-41	4
**BIN2**	1.09E-34	0.371931749	0.553	0.587	6.62E-30	4
**CHASERR**	3.49E-40	0.370621845	0.542	0.569	2.11E-35	4
**C9orf16**	1.44E-76	0.370605195	0.807	0.848	8.71E-72	4
**CFLAR**	3.95E-34	0.369874145	0.377	0.333	2.40E-29	4
**LCP2**	3.69E-28	0.369737594	0.476	0.495	2.24E-23	4
**ANXA6**	3.75E-24	0.369535851	0.436	0.449	2.27E-19	4
**YIPF4**	9.57E-23	0.368943578	0.405	0.407	5.80E-18	4
**CD3E**	4.09E-104	0.368320887	0.922	0.939	2.48E-99	4
**SYTL1**	1.86E-36	0.367761567	0.373	0.323	1.13E-31	4
**CAST**	1.81E-85	0.367481009	0.198	0.09	1.10E-80	4
**NKTR**	2.57E-53	0.36734496	0.728	0.767	1.56E-48	4
**RP11-463O12**	1.02E-26	0.36696163	0.374	0.348	6.18E-22	4
**RNF213**	1.13E-32	0.366672738	0.492	0.504	6.87E-28	4
**ANKRD13D**	1.32E-32	0.366271988	0.33	0.281	8.00E-28	4
**TIAM1**	8.43E-37	0.363629693	0.229	0.159	5.11E-32	4
**MT-ATP6**	4.56E-172	0.36340168	1	0.991	2.77E-167	4
**CRBN**	1.98E-25	0.363022027	0.387	0.374	1.20E-20	4
**DAZAP2**	2.89E-34	0.362858944	0.513	0.539	1.75E-29	4
**NFATC3**	2.15E-26	0.362387592	0.281	0.234	1.31E-21	4
**IER2**	5.02E-30	0.361894704	0.568	0.581	3.05E-25	4
**SCAI**	1.94E-29	0.361723556	0.474	0.484	1.18E-24	4
**CTSC**	1.80E-29	0.359244001	0.549	0.605	1.09E-24	4
**CD79A**	7.13E-66	0.359242638	0.194	0.097	4.32E-61	4
**TMC6**	8.81E-43	0.359133036	0.272	0.194	5.34E-38	4
**JAK1**	4.24E-26	0.359066002	0.448	0.459	2.57E-21	4
**NKG7**	0.00E+00	0.358858874	0.145	0.017	0.00E+00	4
**SLC2A3**	8.78E-23	0.357701624	0.308	0.273	5.33E-18	4
**DDX6**	2.80E-35	0.357209285	0.555	0.589	1.70E-30	4
**TPM3**	1.64E-56	0.356438205	0.851	0.887	9.95E-52	4
**TTC14**	2.54E-25	0.356334366	0.371	0.343	1.54E-20	4
**CREBRF**	1.55E-51	0.355778131	0.26	0.168	9.42E-47	4

**RASGRP2**	1.21E-17	0.355686776	0.335	0.325	7.34E-13	4
**GRK6**	9.57E-23	0.353593836	0.408	0.416	5.81E-18	4
**FOXP1**	1.29E-17	0.352745838	0.408	0.423	7.84E-13	4
**IQGAP1**	1.07E-27	0.352564677	0.495	0.52	6.52E-23	4
**KLHL24**	5.25E-40	0.35233973	0.302	0.227	3.19E-35	4
**ARGLU1**	4.55E-47	0.351779363	0.685	0.749	2.76E-42	4
**CCDC57**	1.08E-26	0.351719246	0.346	0.31	6.54E-22	4
**ARRB2**	1.20E-23	0.351590822	0.289	0.25	7.31E-19	4
**SYF2**	1.65E-26	0.350795747	0.525	0.575	1.00E-21	4
**OPTN**	1.07E-82	0.350479244	0.188	0.084	6.51E-78	4
**CELF2**	1.02E-35	0.350343222	0.606	0.655	6.16E-31	4
**ELF1**	5.55E-27	0.349872272	0.502	0.531	3.37E-22	4
**BAZ2B**	5.40E-30	0.34842995	0.366	0.325	3.27E-25	4
**TLE4**	8.16E-19	0.347390548	0.402	0.415	4.95E-14	4
**ORAI2**	5.67E-26	0.346904794	0.517	0.55	3.44E-21	4
**ZBTB20**	1.88E-36	0.343797764	0.285	0.211	1.14E-31	4
**R3HDM4**	1.07E-35	0.343441919	0.256	0.188	6.49E-31	4
**DUSP2**	2.72E-22	0.343334141	0.3	0.258	1.65E-17	4
**PAXX**	5.00E-13	0.343158283	0.42	0.466	3.03E-08	4
**SNHG14**	1.97E-36	0.342699392	0.622	0.647	1.19E-31	4
**CHURC1**	1.08E-23	0.341400298	0.467	0.493	6.52E-19	4
**TTC24**	7.76E-36	0.340822041	0.231	0.16	4.71E-31	4
**ITGB2-AS1**	4.13E-91	0.339351279	0.181	0.074	2.50E-86	4
**NCOA7**	1.46E-20	0.337766986	0.266	0.228	8.84E-16	4
**TRGC2**	7.19E-51	0.335381943	0.807	0.798	4.36E-46	4
**EPC1**	1.61E-25	0.33402415	0.497	0.529	9.76E-21	4
**PRMT2**	7.31E-16	0.33379148	0.344	0.341	4.43E-11	4
**DYRK2**	1.57E-51	0.332230128	0.194	0.109	9.55E-47	4
**CASC15**	7.18E-45	0.331702364	0.734	0.798	4.35E-40	4
**SEPTIN9**	3.32E-36	0.331272034	0.59	0.645	2.01E-31	4
**POLR2J3.1**	3.83E-24	0.330729916	0.503	0.54	2.32E-19	4
**TNFAIP8**	1.67E-20	0.330520919	0.507	0.558	1.01E-15	4
**BIRC3**	2.26E-64	0.329259477	0.171	0.08	1.37E-59	4
**RCSD1**	1.65E-35	0.32842374	0.62	0.666	9.99E-31	4
**TGOLN2**	3.19E-24	0.328423026	0.511	0.55	1.94E-19	4
**ACAP2**	1.81E-17	0.328041377	0.341	0.333	1.10E-12	4
**CDC42SE2**	1.69E-18	0.327908289	0.392	0.398	1.02E-13	4
**MT-CO3**	9.23E-180	0.326729985	1	0.993	5.60E-175	4
**RFLNB**	1.90E-17	0.325630507	0.482	0.527	1.15E-12	4
**RNPEPL1**	2.57E-31	0.323475681	0.238	0.176	1.56E-26	4
**ARHGAP25**	2.33E-17	0.322849679	0.288	0.26	1.42E-12	4
**KMT2C**	2.67E-21	0.322752127	0.486	0.522	1.62E-16	4
**TAPBP**	1.72E-14	0.321961381	0.422	0.459	1.04E-09	4
**THUMPD3-AS**	9.57E-19	0.321865888	0.366	0.355	5.80E-14	4
**REL**	6.99E-19	0.321555103	0.31	0.287	4.24E-14	4
**CDK13**	4.65E-15	0.319906149	0.399	0.42	2.82E-10	4
**ARHGAP45**	5.46E-16	0.318475562	0.311	0.297	3.31E-11	4
**LINC00173**	3.75E-96	0.317867015	0.163	0.06	2.27E-91	4
**FMNL1-DT**	2.00E-22	0.317804351	0.259	0.213	1.21E-17	4
**SP100**	1.05E-16	0.316536321	0.297	0.273	6.38E-12	4
**STAG3**	6.74E-23	0.316420121	0.216	0.163	4.09E-18	4
**ODF2L**	6.61E-15	0.315751582	0.477	0.535	4.01E-10	4
**PTGDR2**	9.06E-157	0.315324332	0.149	0.036	5.49E-152	4
**KRT1**	7.42E-10	0.314852629	0.326	0.328	4.50E-05	4
**CD2**	2.63E-39	0.31435183	0.758	0.832	1.59E-34	4

**FXYD5**	4.66E-19	0.314350081	0.497	0.555	2.83E-14	4
**RASSF5**	3.96E-16	0.313964717	0.272	0.244	2.40E-11	4
**SYNRG**	3.09E-14	0.313800811	0.394	0.415	1.87E-09	4
**TMBIM4**	3.67E-15	0.313221768	0.291	0.272	2.22E-10	4
**TMC8**	2.32E-16	0.312024052	0.332	0.321	1.41E-11	4
**SNX14**	4.42E-31	0.311914337	0.246	0.179	2.68E-26	4
**THEMIS**	1.22E-75	0.310861074	0.166	0.071	7.41E-71	4
**SIGIRR**	2.19E-15	0.310271677	0.351	0.355	1.33E-10	4
**MLLT6**	1.74E-13	0.308853695	0.389	0.412	1.06E-08	4
**DSTN**	2.52E-14	0.307638975	0.479	0.548	1.53E-09	4
**JUN**	4.72E-08	0.307322796	0.203	0.18	2.87E-03	4
**DRAP1**	2.27E-23	0.306539883	0.583	0.658	1.38E-18	4
**OGA**	2.86E-18	0.305474348	0.385	0.383	1.74E-13	4
**CCM2**	6.94E-14	0.30511094	0.397	0.423	4.21E-09	4
**GMFG**	1.98E-56	0.304813791	0.869	0.919	1.20E-51	4
**MXD4**	1.26E-22	0.303601291	0.415	0.404	7.65E-18	4
**CDC42SE1**	7.40E-17	0.302455803	0.242	0.207	4.49E-12	4
**RCAN3**	2.38E-61	0.3019055	0.174	0.085	1.44E-56	4
**JAK3**	9.69E-25	0.30186881	0.227	0.174	5.87E-20	4
**RBMS1**	2.22E-09	0.300982974	0.367	0.397	1.35E-04	4
**MT-CO2**	6.34E-132	0.300921379	1	0.991	3.85E-127	4
**APOBEC3G**	2.08E-22	0.299143093	0.218	0.169	1.26E-17	4
**DIAPH1**	3.42E-42	0.298507437	0.785	0.841	2.07E-37	4
**RP5-1028K7.2**	1.34E-32	0.297168047	0.189	0.122	8.12E-28	4
**PBXIP1**	5.88E-25	0.297114833	0.308	0.26	3.57E-20	4
**RAC2**	1.19E-41	0.297096375	0.755	0.807	7.21E-37	4
**SELL**	5.14E-07	0.296232969	0.35	0.384	3.12E-02	4
**LAMTOR4**	1.72E-13	0.296159542	0.402	0.435	1.05E-08	4
**TRIM38**	9.59E-12	0.295271542	0.274	0.26	5.82E-07	4
**MT-ND3**	1.83E-60	0.293688507	0.966	0.965	1.11E-55	4
**ANXA11**	8.50E-11	0.293573418	0.4	0.443	5.16E-06	4
**PPP2R5C**	3.54E-07	0.292913379	0.359	0.397	2.15E-02	4
**GABPB1-IT1**	6.43E-13	0.292307386	0.352	0.36	3.90E-08	4
**MBD5**	4.08E-14	0.292022909	0.276	0.252	2.48E-09	4
**KMT2E**	3.28E-17	0.290960004	0.509	0.573	1.99E-12	4
**MED10**	5.61E-12	0.290015154	0.362	0.381	3.40E-07	4
**YPEL1**	3.32E-13	0.289936698	0.305	0.294	2.01E-08	4
**KCNAB2**	3.24E-11	0.28872715	0.451	0.512	1.96E-06	4
**EID1**	1.12E-48	0.288609784	0.794	0.857	6.80E-44	4
**TSPAN2**	1.07E-106	0.288565531	0.138	0.042	6.50E-102	4
**BACH2**	3.19E-54	0.287854146	0.166	0.083	1.93E-49	4
**SCFD1**	1.21E-11	0.287063673	0.419	0.461	7.33E-07	4
**ZC3HAV1**	8.12E-09	0.286178208	0.396	0.44	4.93E-04	4
**PLP2**	8.30E-12	0.285528788	0.236	0.214	5.03E-07	4
**SELPLG**	6.02E-44	0.285359872	0.189	0.11	3.65E-39	4
**RICTOR**	3.60E-12	0.285296278	0.307	0.299	2.19E-07	4
**PIK3C2B**	3.93E-14	0.284937495	0.296	0.28	2.38E-09	4
**NT5C**	7.58E-11	0.284771181	0.361	0.382	4.60E-06	4
**IL16**	3.83E-34	0.284437333	0.186	0.117	2.32E-29	4
**RBM4**	3.68E-13	0.284128572	0.403	0.432	2.23E-08	4
**ST3GAL1**	1.65E-33	0.283702484	0.185	0.117	1.00E-28	4
**MTATP6P1**	1.37E-38	0.283407336	0.833	0.885	8.29E-34	4
**NME3**	1.75E-24	0.283306453	0.225	0.17	1.06E-19	4
**ATP2B4**	4.05E-13	0.28290477	0.432	0.471	2.45E-08	4
**BCL11B**	2.18E-44	0.282478287	0.849	0.9	1.32E-39	4

**FCGRT**	1.22E-17	0.281780815	0.22	0.179	7.39E-13	4
**CHD2**	1.12E-13	0.28154936	0.382	0.394	6.80E-09	4
**MAPRE2**	1.10E-10	0.281243812	0.296	0.293	6.67E-06	4
**CTSW**	3.25E-99	0.281025169	0.119	0.035	1.97E-94	4
**RASAL3**	5.37E-13	0.27889664	0.288	0.271	3.26E-08	4
**RP3-395M20.**	8.51E-17	0.277968797	0.218	0.177	5.16E-12	4
**PTPN22**	3.97E-22	0.27750891	0.218	0.167	2.41E-17	4
**NCKAP1L**	5.13E-09	0.276555711	0.337	0.354	3.11E-04	4
**SP140L**	1.60E-09	0.273642574	0.25	0.238	9.71E-05	4
**AQP3**	1.46E-39	0.273584574	0.164	0.093	8.87E-35	4
**MMP24OS**	1.96E-12	0.273461072	0.263	0.244	1.19E-07	4
**TCF25**	8.38E-09	0.272136575	0.428	0.487	5.08E-04	4
**LCK**	6.60E-38	0.271908876	0.808	0.876	4.00E-33	4
**SPOCK2**	1.14E-241	0.271380328	0.114	0.014	6.91E-237	4
**TOB1**	1.29E-18	0.271243826	0.196	0.151	7.80E-14	4
**NDST3**	7.31E-10	0.270122275	0.34	0.341	4.43E-05	4
**ACTR2**	2.08E-21	0.270091285	0.6	0.683	1.26E-16	4
**POMT1**	2.88E-20	0.269844488	0.206	0.157	1.75E-15	4
**FAM89B**	1.11E-10	0.269808075	0.426	0.476	6.71E-06	4
**PDE3B**	3.28E-07	0.269261049	0.245	0.238	1.99E-02	4
**WASF2**	3.60E-16	0.267924929	0.55	0.637	2.18E-11	4
**GCSAML**	3.46E-21	0.265429924	0.214	0.162	2.10E-16	4
**ATP6V1G1**	1.17E-38	0.263874352	0.696	0.768	7.12E-34	4
**MT-CYB**	1.04E-69	0.263519465	0.997	0.988	6.29E-65	4
**FBRS**	1.92E-12	0.263412007	0.254	0.232	1.16E-07	4
**TRBC2**	1.70E-79	0.263338386	0.988	0.982	1.03E-74	4
**CD226**	3.86E-238	0.263259377	0.108	0.012	2.34E-233	4
**ZFP36**	6.60E-65	0.263109068	0.174	0.083	4.00E-60	4
**IDS**	2.41E-23	0.262435536	0.175	0.121	1.46E-18	4
**SYNJ2**	1.22E-08	0.262331053	0.393	0.436	7.41E-04	4
**ITGAL**	4.14E-09	0.26197572	0.432	0.487	2.51E-04	4
**PTEN**	2.53E-16	0.26189692	0.584	0.669	1.54E-11	4
**PRKCQ-AS1**	2.60E-08	0.261782189	0.451	0.52	1.58E-03	4
**KRT2**	5.29E-98	0.26119519	0.132	0.042	3.21E-93	4
**CYLD**	4.51E-14	0.261060785	0.25	0.222	2.74E-09	4
**PDLIM2**	1.61E-09	0.261056089	0.23	0.214	9.78E-05	4
**RP11-108K14.**	1.75E-09	0.260628462	0.291	0.289	1.06E-04	4
**RP11-284N8.3**	8.35E-25	0.260569798	0.171	0.116	5.06E-20	4
**ARL6IP5**	7.70E-07	0.258790616	0.393	0.448	4.67E-02	4
**BAZ2A**	5.80E-10	0.25834655	0.294	0.29	3.52E-05	4
**RP11-198M19**	2.40E-29	0.258035584	0.197	0.133	1.45E-24	4
**BCL9L**	2.18E-28	0.255980524	0.163	0.104	1.32E-23	4
**DGKA**	3.40E-08	0.255658717	0.32	0.331	2.06E-03	4
**LINC02384**	2.25E-16	0.255230147	0.238	0.196	1.36E-11	4
**SLFN5**	6.55E-15	0.255014736	0.58	0.639	3.97E-10	4
**WDR26**	1.41E-13	0.254798917	0.257	0.231	8.57E-09	4
**GSTK1**	1.12E-09	0.254662638	0.281	0.276	6.80E-05	4
**ADCY7**	1.62E-60	0.254502706	0.149	0.067	9.83E-56	4
**LRP10**	2.85E-24	0.254380909	0.177	0.122	1.73E-19	4
**SPN**	9.62E-18	0.253188566	0.64	0.728	5.84E-13	4
**ERICH1**	4.13E-07	0.253154497	0.286	0.292	2.50E-02	4
**TCF7**	1.55E-30	0.25166936	0.794	0.853	9.38E-26	4
**CITED2**	2.58E-40	0.250851378	0.146	0.077	1.57E-35	4
**INPP4A**	8.23E-08	0.250388457	0.218	0.205	4.99E-03	4
