# Supplementary material for: High CD44 expression and enhanced E-selectin binding identified as biomarkers of chemoresistant leukemic cells in human T-ALL
Source: Leukemia. 2024 Nov 24;39(2):323–36. doi: 10.1038/s41375-024-02473-7 (PMC11794132; doi:10.1038/s41375-024-02473-7)
Supplement: Supplementary file 5 — Supplemental Table 4 [file 41375_2024_2473_MOESM5_ESM.pdf]

Common upregulated genes between Cluster 4 & LRC signature

GENENAME
KLF2
S100A10
EMP3
LGALS1
TXNIP
MALAT1
ARL4C
RIPOR2
CD44
HCST
FOS
HLA-E
TPST2
B2M
TSC22D3
TIMP1
NKG7
NEAT1
HLA-B
PNRC1
EIF1
SPOCK2
RCBTB2
ATM
PPP1R15A
LINC-PINT
CDKN2D
LAPTM5
DUSP1
HLA-A
CST7
ITM2B
KLF13
CD37
BTG1
TRAF3IP3
HLA-C
ARAP2
SARAF
UBC
RAB37
FMNL1
LRRFIP1
TRAC
C16orf54
AAK1
ZFP36
ICAM3
LPAR6
PGGHG
PTGER4
NKTR
CD53
CASC15
PSMA3-AS1
KLHL24
TIAM1
ZBTB20
BIN2
IER2
BAZ2B
ANXA6
FCGRT
RFLNB
RASSF5
CCM2
PLP2
TRIM38
RBMS1
SELL
