# Supplementary material for: High CD44 expression and enhanced E-selectin binding identified as biomarkers of chemoresistant leukemic cells in human T-ALL
Source: Leukemia. 2024 Nov 24;39(2):323–36. doi: 10.1038/s41375-024-02473-7 (PMC11794132; doi:10.1038/s41375-024-02473-7)
Supplement: Supplementary file 6 — Supplemental Table 5 [file 41375_2024_2473_MOESM6_ESM.pdf]

upregulated genes in Ki67neg CD44high leukemic cells from Library 1 (M104 & M104R) (Figure 4f)

	p_val	avg_log2FC	pct.1	pct.2	p_val_adj	cluster	gene
CD44	0	1.886539144	1	0.153	0	CD44>1 & MKI67<1 LEUK	CD44
EIF1	1.99E-98	1.227397396	0.971	0.973	7.28E-94	CD44>1 & MKI67<1 LEUK	EIF1
HLA-B	3.41E-91	1.530037419	0.786	0.336	1.25E-86	CD44>1 & MKI67<1 LEUK	HLA-B
EMP3	6.58E-83	1.29270205	0.883	0.661	2.41E-78	CD44>1 & MKI67<1 LEUK	EMP3
MALAT1	6.50E-80	0.851685794	1	1	2.38E-75	CD44>1 & MKI67<1 LEUK	MALAT1
YWHAZ	4.62E-71	1.039794419	0.927	0.869	1.69E-66	CD44>1 & MKI67<1 LEUK	YWHAZ
TRBV5-4	8.31E-66	0.989217825	0.9	0.371	3.04E-61	CD44>1 & MKI67<1 LEUK	TRBV5-4
STK17B	1.76E-65	1.166198682	0.842	0.604	6.44E-61	CD44>1 & MKI67<1 LEUK	STK17B
IFITM1	8.77E-65	0.905217969	0.985	0.975	3.21E-60	CD44>1 & MKI67<1 LEUK	IFITM1
CDKN2D	5.83E-58	1.039018516	0.701	0.371	2.13E-53	CD44>1 & MKI67<1 LEUK	CDKN2D
KHDRBS3	3.80E-57	1.024864084	0.396	0.114	1.39E-52	CD44>1 & MKI67<1 LEUK	KHDRBS3
FTH1	5.31E-55	0.975797231	0.994	0.996	1.95E-50	CD44>1 & MKI67<1 LEUK	FTH1
ANKRD12	2.93E-53	1.104970062	0.654	0.361	1.07E-48	CD44>1 & MKI67<1 LEUK	ANKRD12
PRKX	3.03E-49	0.867056892	0.455	0.168	1.11E-44	CD44>1 & MKI67<1 LEUK	PRKX
RPL30	1.53E-47	0.493509016	0.997	0.999	5.59E-43	CD44>1 & MKI67<1 LEUK	RPL30
GNA15	1.88E-47	0.963900978	0.812	0.683	6.88E-43	CD44>1 & MKI67<1 LEUK	GNA15
UBC	3.86E-47	0.756222082	0.971	0.97	1.41E-42	CD44>1 & MKI67<1 LEUK	UBC
RGS16	3.87E-46	0.744110364	0.226	0.044	1.42E-41	CD44>1 & MKI67<1 LEUK	RGS16
LIMD2	8.99E-45	1.016653036	0.642	0.383	3.29E-40	CD44>1 & MKI67<1 LEUK	LIMD2
RBM3	9.73E-44	0.915456231	0.815	0.723	3.56E-39	CD44>1 & MKI67<1 LEUK	RBM3
METRNL	6.62E-43	0.715696882	0.349	0.107	2.42E-38	CD44>1 & MKI67<1 LEUK	METRNL
EZR	2.87E-40	1.043198727	0.622	0.407	1.05E-35	CD44>1 & MKI67<1 LEUK	EZR
FXYD5	3.28E-40	0.92244138	0.789	0.748	1.20E-35	CD44>1 & MKI67<1 LEUK	FXYD5
RBM17	3.54E-40	1.019300883	0.68	0.476	1.30E-35	CD44>1 & MKI67<1 LEUK	RBM17
RELB	8.38E-40	0.736321381	0.323	0.099	3.07E-35	CD44>1 & MKI67<1 LEUK	RELB
TPT1	2.82E-39	0.427226552	0.991	0.998	1.03E-34	CD44>1 & MKI67<1 LEUK	TPT1
JUND	3.65E-38	0.812188019	0.848	0.852	1.34E-33	CD44>1 & MKI67<1 LEUK	JUND
B2M	7.71E-37	0.624082947	0.979	0.981	2.82E-32	CD44>1 & MKI67<1 LEUK	B2M
HLA-C	2.90E-34	0.857630145	0.651	0.456	1.06E-29	CD44>1 & MKI67<1 LEUK	HLA-C
IRF9	3.78E-34	0.692905662	0.399	0.164	1.38E-29	CD44>1 & MKI67<1 LEUK	IRF9
BCL3	1.44E-32	0.624825553	0.29	0.092	5.26E-28	CD44>1 & MKI67<1 LEUK	BCL3
HLA-E	3.35E-32	0.904294664	0.563	0.349	1.23E-27	CD44>1 & MKI67<1 LEUK	HLA-E
IER2	4.94E-32	0.767230454	0.619	0.415	1.81E-27	CD44>1 & MKI67<1 LEUK	IER2
BHLHE40	8.03E-32	0.819518313	0.46	0.229	2.94E-27	CD44>1 & MKI67<1 LEUK	BHLHE40
TPM4	2.54E-29	0.920509204	0.616	0.459	9.30E-25	CD44>1 & MKI67<1 LEUK	TPM4
EEF1D	3.70E-29	0.503251745	0.927	0.972	1.35E-24	CD44>1 & MKI67<1 LEUK	EEF1D
ARID5A	1.18E-28	0.740558302	0.472	0.252	4.32E-24	CD44>1 & MKI67<1 LEUK	ARID5A
NFKB1	1.54E-28	0.560081381	0.264	0.088	5.64E-24	CD44>1 & MKI67<1 LEUK	NFKB1
MX1	2.43E-28	0.763221115	0.449	0.229	8.90E-24	CD44>1 & MKI67<1 LEUK	MX1
KLF6	3.20E-28	0.706727099	0.95	0.94	1.17E-23	CD44>1 & MKI67<1 LEUK	KLF6
H3F3B	4.15E-28	0.480702324	0.991	0.996	1.52E-23	CD44>1 & MKI67<1 LEUK	H3F3B
RNF125	9.76E-28	0.653149295	0.358	0.157	3.57E-23	CD44>1 & MKI67<1 LEUK	RNF125
PNRC1	1.03E-26	0.71569578	0.836	0.839	3.78E-22	CD44>1 & MKI67<1 LEUK	PNRC1
FTL	3.17E-26	0.48081883	0.935	0.963	1.16E-21	CD44>1 & MKI67<1 LEUK	FTL
LRRFIP1	6.47E-26	0.856621352	0.619	0.51	2.37E-21	CD44>1 & MKI67<1 LEUK	LRRFIP1
SH3BGRL3	4.67E-25	0.66206708	0.833	0.852	1.71E-20	CD44>1 & MKI67<1 LEUK	SH3BGRL3
ARAP2	6.77E-25	0.640267231	0.32	0.14	2.48E-20	CD44>1 & MKI67<1 LEUK	ARAP2
FLT1	3.47E-24	0.445495057	0.223	0.072	1.27E-19	CD44>1 & MKI67<1 LEUK	FLT1
S100A10	3.77E-24	0.697263882	0.352	0.159	1.38E-19	CD44>1 & MKI67<1 LEUK	S100A10
PGK1	9.73E-24	0.712515251	0.698	0.682	3.56E-19	CD44>1 & MKI67<1 LEUK	PGK1
TLX3	1.45E-23	0.630607165	0.754	0.737	5.30E-19	CD44>1 & MKI67<1 LEUK	TLX3

REL	2.01E-23	0.633927912	0.372	0.188	7.36E-19	CD44>1 & MKI67<1 LEUK	REL
S1PR1	3.04E-23	0.652245689	0.334	0.155	1.11E-18	CD44>1 & MKI67<1 LEUK	S1PR1
PRNP	3.44E-23	0.722889243	0.425	0.243	1.26E-18	CD44>1 & MKI67<1 LEUK	PRNP
EML4	1.11E-22	0.724727409	0.384	0.208	4.05E-18	CD44>1 & MKI67<1 LEUK	EML4
TWIST1	1.33E-22	0.437007556	0.144	0.035	4.89E-18	CD44>1 & MKI67<1 LEUK	TWIST1
ECE1	3.33E-22	0.504105694	0.223	0.079	1.22E-17	CD44>1 & MKI67<1 LEUK	ECE1
NEU4	3.47E-22	0.400593505	0.199	0.064	1.27E-17	CD44>1 & MKI67<1 LEUK	NEU4
CXCL8	4.62E-22	0.725637099	0.261	0.102	1.69E-17	CD44>1 & MKI67<1 LEUK	CXCL8
SKIL	6.52E-22	0.443891671	0.208	0.07	2.39E-17	CD44>1 & MKI67<1 LEUK	SKIL
TRAF4	6.67E-22	0.596879603	0.317	0.144	2.44E-17	CD44>1 & MKI67<1 LEUK	TRAF4
PHLDA1	1.63E-21	0.571512283	0.246	0.094	5.95E-17	CD44>1 & MKI67<1 LEUK	PHLDA1
MAP3K8	1.68E-21	0.670199265	0.349	0.175	6.16E-17	CD44>1 & MKI67<1 LEUK	MAP3K8
LINC01578	3.55E-21	0.653126311	0.701	0.664	1.30E-16	CD44>1 & MKI67<1 LEUK	LINC01578
UPP1	5.66E-21	0.369427371	0.185	0.058	2.07E-16	CD44>1 & MKI67<1 LEUK	UPP1
AREG	2.49E-20	0.450168568	0.12	0.028	9.10E-16	CD44>1 & MKI67<1 LEUK	AREG
RAB22A	4.22E-20	0.603634254	0.328	0.166	1.54E-15	CD44>1 & MKI67<1 LEUK	RAB22A
IER3	4.85E-20	0.648549018	0.296	0.134	1.78E-15	CD44>1 & MKI67<1 LEUK	IER3
PDP1	1.83E-19	0.391712835	0.185	0.062	6.70E-15	CD44>1 & MKI67<1 LEUK	PDP1
CLIC1	1.10E-18	0.536784768	0.804	0.869	4.02E-14	CD44>1 & MKI67<1 LEUK	CLIC1
VIM	1.28E-18	0.467569863	0.974	0.971	4.67E-14	CD44>1 & MKI67<1 LEUK	VIM
KLF10	3.70E-18	0.563868455	0.299	0.143	1.36E-13	CD44>1 & MKI67<1 LEUK	KLF10
ETS2	4.16E-18	0.742215566	0.607	0.544	1.52E-13	CD44>1 & MKI67<1 LEUK	ETS2
SH3BP5	4.21E-18	0.708261789	0.581	0.484	1.54E-13	CD44>1 & MKI67<1 LEUK	SH3BP5
ADARB2	4.91E-18	0.328379759	0.129	0.035	1.80E-13	CD44>1 & MKI67<1 LEUK	ADARB2
IRF1	9.90E-18	0.595601618	0.337	0.176	3.62E-13	CD44>1 & MKI67<1 LEUK	IRF1
FAM177A1	1.18E-17	0.573676353	0.328	0.175	4.34E-13	CD44>1 & MKI67<1 LEUK	FAM177A1
JUNB	1.67E-17	0.593356364	0.299	0.149	6.12E-13	CD44>1 & MKI67<1 LEUK	JUNB
IL2RA	4.26E-17	0.351364135	0.158	0.051	1.56E-12	CD44>1 & MKI67<1 LEUK	IL2RA
TMSB10	6.18E-17	0.423959524	0.988	0.998	2.26E-12	CD44>1 & MKI67<1 LEUK	TMSB10
PABPC1	6.22E-17	0.500094357	0.786	0.815	2.28E-12	CD44>1 & MKI67<1 LEUK	PABPC1
OASL	2.69E-16	0.453862239	0.211	0.085	9.86E-12	CD44>1 & MKI67<1 LEUK	OASL
TPM3	3.40E-16	0.588245585	0.754	0.817	1.25E-11	CD44>1 & MKI67<1 LEUK	TPM3
RHOU	5.54E-16	0.652099817	0.434	0.28	2.03E-11	CD44>1 & MKI67<1 LEUK	RHOU
RBM38	6.00E-16	0.345686289	0.15	0.049	2.20E-11	CD44>1 & MKI67<1 LEUK	RBM38
DDX3X	6.97E-16	0.692233468	0.472	0.348	2.55E-11	CD44>1 & MKI67<1 LEUK	DDX3X
S100A4	8.63E-16	0.685055356	0.616	0.547	3.16E-11	CD44>1 & MKI67<1 LEUK	S100A4
EIF4G2	9.17E-16	0.639536275	0.595	0.542	3.36E-11	CD44>1 & MKI67<1 LEUK	EIF4G2
SYAP1	9.50E-16	0.605290978	0.422	0.278	3.48E-11	CD44>1 & MKI67<1 LEUK	SYAP1
LMBR1	1.03E-15	0.545788164	0.364	0.223	3.77E-11	CD44>1 & MKI67<1 LEUK	LMBR1
MIR4435-2HG	2.59E-15	0.697137202	0.496	0.394	9.48E-11	CD44>1 & MKI67<1 LEUK	MIR4435-2HG
HIF1A	4.02E-15	0.627835264	0.384	0.252	1.47E-10	CD44>1 & MKI67<1 LEUK	HIF1A
SAT1	5.14E-15	0.699030715	0.478	0.363	1.88E-10	CD44>1 & MKI67<1 LEUK	SAT1
RBM39	5.58E-15	0.535292122	0.639	0.635	2.04E-10	CD44>1 & MKI67<1 LEUK	RBM39
PIK3R1	6.30E-15	0.635094799	0.548	0.463	2.30E-10	CD44>1 & MKI67<1 LEUK	PIK3R1
CTSW	6.65E-15	0.677938544	0.378	0.251	2.43E-10	CD44>1 & MKI67<1 LEUK	CTSW
CDC42	1.10E-14	0.564595304	0.669	0.691	4.01E-10	CD44>1 & MKI67<1 LEUK	CDC42
PLAU	1.76E-14	0.521897931	0.232	0.109	6.43E-10	CD44>1 & MKI67<1 LEUK	PLAU
S100A11	4.26E-14	0.638986248	0.452	0.338	1.56E-09	CD44>1 & MKI67<1 LEUK	S100A11
IL4R	5.83E-14	0.368447821	0.167	0.064	2.13E-09	CD44>1 & MKI67<1 LEUK	IL4R
MAL	2.23E-13	0.641780366	0.804	0.868	8.16E-09	CD44>1 & MKI67<1 LEUK	MAL
MAFB	2.30E-13	0.36055508	0.161	0.061	8.42E-09	CD44>1 & MKI67<1 LEUK	MAFB
CDV3	2.93E-13	0.605549423	0.46	0.369	1.07E-08	CD44>1 & MKI67<1 LEUK	CDV3
PELI1	3.12E-13	0.610573502	0.284	0.162	1.14E-08	CD44>1 & MKI67<1 LEUK	PELI1
CYTOR	5.25E-13	0.55993561	0.584	0.54	1.92E-08	CD44>1 & MKI67<1 LEUK	CYTOR

IDS	6.94E-13	0.505347476	0.311	0.191	2.54E-08	CD44>1 & MKI67<1 LEUK	IDS
GYPC	1.24E-12	0.501832132	0.449	0.317	4.53E-08	CD44>1 & MKI67<1 LEUK	GYPC
IFIT3	1.95E-12	0.482285542	0.249	0.126	7.15E-08	CD44>1 & MKI67<1 LEUK	IFIT3
CD69	3.26E-12	0.518441933	0.205	0.097	1.19E-07	CD44>1 & MKI67<1 LEUK	CD69
DENND3	8.31E-12	0.292842823	0.132	0.049	3.04E-07	CD44>1 & MKI67<1 LEUK	DENND3
PPP1R16B	9.12E-12	0.355859138	0.173	0.074	3.34E-07	CD44>1 & MKI67<1 LEUK	PPP1R16B
CD7	1.01E-11	0.525828966	0.783	0.867	3.69E-07	CD44>1 & MKI67<1 LEUK	CD7
STT3B	1.21E-11	0.669664679	0.481	0.409	4.41E-07	CD44>1 & MKI67<1 LEUK	STT3B
ARL4C	1.28E-11	0.588149021	0.695	0.755	4.70E-07	CD44>1 & MKI67<1 LEUK	ARL4C
BZW1	2.32E-11	0.586964614	0.49	0.429	8.47E-07	CD44>1 & MKI67<1 LEUK	BZW1
CRYBG1	2.74E-11	0.448372485	0.214	0.109	1.00E-06	CD44>1 & MKI67<1 LEUK	CRYBG1
ZNFX1	3.36E-11	0.312938402	0.173	0.078	1.23E-06	CD44>1 & MKI67<1 LEUK	ZNFX1
CLEC2B	4.24E-11	0.38624372	0.164	0.072	1.55E-06	CD44>1 & MKI67<1 LEUK	CLEC2B
CMPK2	4.45E-11	0.3952223	0.173	0.079	1.63E-06	CD44>1 & MKI67<1 LEUK	CMPK2
YBX3	8.70E-11	0.550513177	0.642	0.693	3.19E-06	CD44>1 & MKI67<1 LEUK	YBX3
HLA-A	8.90E-11	0.558402411	0.657	0.675	3.26E-06	CD44>1 & MKI67<1 LEUK	HLA-A
CTDP1	1.73E-10	0.37651597	0.243	0.137	6.33E-06	CD44>1 & MKI67<1 LEUK	CTDP1
TRBV18	2.14E-10	0.276896605	0.167	0.075	7.83E-06	CD44>1 & MKI67<1 LEUK	TRBV18
STK17A	2.18E-10	0.539267771	0.393	0.303	7.99E-06	CD44>1 & MKI67<1 LEUK	STK17A
CSF3R	2.36E-10	0.274262536	0.152	0.066	8.65E-06	CD44>1 & MKI67<1 LEUK	CSF3R
CALM1	3.03E-10	0.541858309	0.648	0.708	1.11E-05	CD44>1 & MKI67<1 LEUK	CALM1
GUK1	3.27E-10	0.490937815	0.589	0.627	1.20E-05	CD44>1 & MKI67<1 LEUK	GUK1
NINJ1	3.56E-10	0.476354118	0.264	0.16	1.30E-05	CD44>1 & MKI67<1 LEUK	NINJ1
NAMPT	4.54E-10	0.358424056	0.188	0.092	1.66E-05	CD44>1 & MKI67<1 LEUK	NAMPT
EIF5	4.66E-10	0.552093293	0.525	0.505	1.71E-05	CD44>1 & MKI67<1 LEUK	EIF5
SBDS	4.97E-10	0.528529061	0.358	0.259	1.82E-05	CD44>1 & MKI67<1 LEUK	SBDS
IRF7	6.88E-10	0.396944043	0.211	0.112	2.52E-05	CD44>1 & MKI67<1 LEUK	IRF7
OTULINL	8.18E-10	0.195954723	0.111	0.041	2.99E-05	CD44>1 & MKI67<1 LEUK	OTULINL
RAP2B	8.20E-10	0.483882963	0.273	0.17	3.00E-05	CD44>1 & MKI67<1 LEUK	RAP2B
ARHGDIA	1.34E-09	0.56462376	0.534	0.523	4.91E-05	CD44>1 & MKI67<1 LEUK	ARHGDIA
SARAF	1.62E-09	0.549553889	0.619	0.669	5.94E-05	CD44>1 & MKI67<1 LEUK	SARAF
ARPC5L	2.54E-09	0.504525147	0.49	0.447	9.31E-05	CD44>1 & MKI67<1 LEUK	ARPC5L
ATP2B1	2.62E-09	0.415348114	0.199	0.109	9.58E-05	CD44>1 & MKI67<1 LEUK	ATP2B1
TRIM24	2.73E-09	0.41864799	0.282	0.181	9.99E-05	CD44>1 & MKI67<1 LEUK	TRIM24
DNAJB6	3.44E-09	0.544003565	0.501	0.477	0.00012607	CD44>1 & MKI67<1 LEUK	DNAJB6
GLS	4.80E-09	0.516371946	0.326	0.237	0.000175569	CD44>1 & MKI67<1 LEUK	GLS
RALA	5.64E-09	0.439566607	0.276	0.181	0.000206439	CD44>1 & MKI67<1 LEUK	RALA
TLE3	5.67E-09	0.443114448	0.238	0.142	0.000207601	CD44>1 & MKI67<1 LEUK	TLE3
GNA13	6.75E-09	0.428711337	0.261	0.165	0.000247205	CD44>1 & MKI67<1 LEUK	GNA13
LY6E	8.31E-09	0.534939857	0.56	0.572	0.000304153	CD44>1 & MKI67<1 LEUK	LY6E
PLEKHB2	9.21E-09	0.409606228	0.22	0.128	0.000336996	CD44>1 & MKI67<1 LEUK	PLEKHB2
KMT2E	1.17E-08	0.555750113	0.484	0.459	0.000429584	CD44>1 & MKI67<1 LEUK	KMT2E
HBEGF	1.53E-08	0.331374342	0.103	0.04	0.000561492	CD44>1 & MKI67<1 LEUK	HBEGF
LAPTM4A	2.21E-08	0.55436007	0.384	0.318	0.00080926	CD44>1 & MKI67<1 LEUK	LAPTM4A
LCP2	2.23E-08	0.543003347	0.34	0.257	0.000815132	CD44>1 & MKI67<1 LEUK	LCP2
ACTB	2.63E-08	0.345785934	0.991	0.994	0.000962808	CD44>1 & MKI67<1 LEUK	ACTB
TRIB2	3.03E-08	0.340714373	0.155	0.077	0.00110844	CD44>1 & MKI67<1 LEUK	TRIB2
OSGIN2	3.28E-08	0.284101799	0.138	0.065	0.00119932	CD44>1 & MKI67<1 LEUK	OSGIN2
PPP1R15A	3.65E-08	0.568089535	0.496	0.474	0.001335142	CD44>1 & MKI67<1 LEUK	PPP1R15A
RASGRP1	3.67E-08	0.270743809	0.144	0.069	0.001343101	CD44>1 & MKI67<1 LEUK	RASGRP1
TNFAIP3	5.27E-08	0.385258479	0.208	0.121	0.001927447	CD44>1 & MKI67<1 LEUK	TNFAIP3
HCST	5.44E-08	0.505180331	0.431	0.374	0.001989293	CD44>1 & MKI67<1 LEUK	HCST
KLF2	6.16E-08	0.3122191	0.152	0.074	0.002253536	CD44>1 & MKI67<1 LEUK	KLF2
ARF6	6.58E-08	0.514222723	0.472	0.455	0.002409799	CD44>1 & MKI67<1 LEUK	ARF6

FLT3LG	7.73E-08	0.527535612	0.305	0.222	0.002827786	CD44>1 & MKI67<1 LEUK	FLT3LG
MYADM	8.11E-08	0.499169505	0.235	0.148	0.002969946	CD44>1 & MKI67<1 LEUK	MYADM
ITM2B	8.32E-08	0.447985751	0.686	0.795	0.003046249	CD44>1 & MKI67<1 LEUK	ITM2B
RNF145	9.00E-08	0.422324784	0.273	0.183	0.003293908	CD44>1 & MKI67<1 LEUK	RNF145
FOSL2	9.72E-08	0.225692245	0.111	0.048	0.003558526	CD44>1 & MKI67<1 LEUK	FOSL2
N4BP1	1.00E-07	0.39554735	0.217	0.132	0.003665245	CD44>1 & MKI67<1 LEUK	N4BP1
ADAM19	1.01E-07	0.247538213	0.1	0.041	0.003703925	CD44>1 & MKI67<1 LEUK	ADAM19
LUZP1	1.23E-07	0.338363973	0.179	0.1	0.004516604	CD44>1 & MKI67<1 LEUK	LUZP1
IL15RA	1.30E-07	0.267010316	0.126	0.058	0.00474468	CD44>1 & MKI67<1 LEUK	IL15RA
STAT3	1.47E-07	0.418968876	0.27	0.186	0.005373456	CD44>1 & MKI67<1 LEUK	STAT3
ZFAND5	1.61E-07	0.441430735	0.252	0.169	0.005900407	CD44>1 & MKI67<1 LEUK	ZFAND5
LTB	1.78E-07	0.557682099	0.44	0.386	0.006507808	CD44>1 & MKI67<1 LEUK	LTB
SACS	2.13E-07	0.330738743	0.179	0.101	0.007805102	CD44>1 & MKI67<1 LEUK	SACS
PIM3	3.50E-07	0.270356323	0.135	0.067	0.012811448	CD44>1 & MKI67<1 LEUK	PIM3
ZC3HAV1	4.15E-07	0.475077872	0.34	0.272	0.01518667	CD44>1 & MKI67<1 LEUK	ZC3HAV1
HSH2D	4.30E-07	0.303233394	0.158	0.085	0.0157455	CD44>1 & MKI67<1 LEUK	HSH2D
NACA	5.26E-07	0.186783938	0.953	0.989	0.019259106	CD44>1 & MKI67<1 LEUK	NACA
JARID2	7.07E-07	0.298102947	0.161	0.089	0.025886899	CD44>1 & MKI67<1 LEUK	JARID2
NR4A2	7.75E-07	0.21894964	0.12	0.057	0.028351449	CD44>1 & MKI67<1 LEUK	NR4A2
ELF1	7.97E-07	0.508894109	0.416	0.38	0.029186124	CD44>1 & MKI67<1 LEUK	ELF1
USP12	1.29E-06	0.342521082	0.196	0.122	0.047157568	CD44>1 & MKI67<1 LEUK	USP12
RB1CC1	1.31E-06	0.439730836	0.323	0.249	0.047951124	CD44>1 & MKI67<1 LEUK	RB1CC1
DDX5	1.43E-06	0.324512424	0.727	0.817	0.052281552	CD44>1 & MKI67<1 LEUK	DDX5
CDK13	1.61E-06	0.36003975	0.261	0.186	0.058895596	CD44>1 & MKI67<1 LEUK	CDK13
CIRBP	1.71E-06	0.345998276	0.695	0.803	0.062699359	CD44>1 & MKI67<1 LEUK	CIRBP
ABCG1	2.17E-06	0.275089237	0.135	0.072	0.079455229	CD44>1 & MKI67<1 LEUK	ABCG1
SPSB1	2.21E-06	0.254956292	0.114	0.056	0.081012042	CD44>1 & MKI67<1 LEUK	SPSB1
MT2A	2.34E-06	1.086659125	0.396	0.358	0.085528109	CD44>1 & MKI67<1 LEUK	MT2A
SF1	2.47E-06	0.410391024	0.566	0.624	0.090379879	CD44>1 & MKI67<1 LEUK	SF1
SOD2	2.83E-06	0.542389674	0.381	0.34	0.103606849	CD44>1 & MKI67<1 LEUK	SOD2
USP15	3.17E-06	0.479349003	0.393	0.358	0.116107464	CD44>1 & MKI67<1 LEUK	USP15
EFHD2	5.01E-06	0.480096636	0.29	0.229	0.183282991	CD44>1 & MKI67<1 LEUK	EFHD2
OSM	5.59E-06	0.323440101	0.106	0.051	0.204583543	CD44>1 & MKI67<1 LEUK	OSM
EPHB6	7.61E-06	0.390142621	0.381	0.332	0.278361795	CD44>1 & MKI67<1 LEUK	EPHB6
UBALD2	7.62E-06	0.57559098	0.501	0.527	0.278856437	CD44>1 & MKI67<1 LEUK	UBALD2
KLF13	7.85E-06	0.449769366	0.449	0.437	0.287298024	CD44>1 & MKI67<1 LEUK	KLF13
RPL36AL	8.21E-06	0.26250171	0.669	0.765	0.300623875	CD44>1 & MKI67<1 LEUK	RPL36AL
RAB8B	9.35E-06	0.305865296	0.167	0.102	0.342180273	CD44>1 & MKI67<1 LEUK	RAB8B
KDSR	9.87E-06	0.302330301	0.185	0.119	0.361256381	CD44>1 & MKI67<1 LEUK	KDSR
SH2D1A	1.02E-05	0.420535027	0.314	0.251	0.374042197	CD44>1 & MKI67<1 LEUK	SH2D1A
RPL21	1.13E-05	0.175204744	0.933	0.98	0.412841686	CD44>1 & MKI67<1 LEUK	RPL21
STRAP	1.23E-05	0.440362442	0.452	0.441	0.449410518	CD44>1 & MKI67<1 LEUK	STRAP
CMTM3	1.34E-05	0.489310949	0.405	0.376	0.490640769	CD44>1 & MKI67<1 LEUK	CMTM3
ATP6V0C	1.36E-05	0.418769239	0.499	0.538	0.496245766	CD44>1 & MKI67<1 LEUK	ATP6V0C
ITM2A	1.38E-05	0.570468117	0.405	0.377	0.505104701	CD44>1 & MKI67<1 LEUK	ITM2A
NR1H2	1.54E-05	0.394258992	0.264	0.202	0.564973768	CD44>1 & MKI67<1 LEUK	NR1H2
MYL12A	1.72E-05	0.400657919	0.666	0.779	0.628386419	CD44>1 & MKI67<1 LEUK	MYL12A
UBE2D3	1.80E-05	0.462064214	0.513	0.562	0.659775128	CD44>1 & MKI67<1 LEUK	UBE2D3
ARID4B	1.92E-05	0.471716028	0.361	0.33	0.701311441	CD44>1 & MKI67<1 LEUK	ARID4B
KDM5B	1.93E-05	0.338995026	0.217	0.149	0.70791638	CD44>1 & MKI67<1 LEUK	KDM5B
GNAS	2.11E-05	0.301365543	0.783	0.884	0.77210805	CD44>1 & MKI67<1 LEUK	GNAS
FNBP1	2.30E-05	0.501919765	0.413	0.404	0.841846262	CD44>1 & MKI67<1 LEUK	FNBP1
ITPRID2	2.89E-05	0.4236029	0.24	0.179	1	CD44>1 & MKI67<1 LEUK	ITPRID2
SOCS1	2.89E-05	0.533988138	0.352	0.31	1	CD44>1 & MKI67<1 LEUK	SOCS1

MOB3A	3.20E-05	0.278704443	0.152	0.093	1	CD44>1 & MKI67<1 LEUK	MOB3A
RNMT	3.38E-05	0.389391242	0.258	0.199	1	CD44>1 & MKI67<1 LEUK	RNMT
IRF2BP2	3.39E-05	0.374329511	0.232	0.169	1	CD44>1 & MKI67<1 LEUK	IRF2BP2
NDUFV2	3.54E-05	0.590436044	0.431	0.437	1	CD44>1 & MKI67<1 LEUK	NDUFV2
SLC7A5	4.32E-05	0.290114858	0.152	0.094	1	CD44>1 & MKI67<1 LEUK	SLC7A5
SMURF2	4.75E-05	0.245829774	0.152	0.094	1	CD44>1 & MKI67<1 LEUK	SMURF2
STAT1	4.89E-05	0.325248122	0.191	0.128	1	CD44>1 & MKI67<1 LEUK	STAT1
SUGT1	4.93E-05	0.504344552	0.326	0.282	1	CD44>1 & MKI67<1 LEUK	SUGT1
SAMD9	5.11E-05	0.25640711	0.167	0.106	1	CD44>1 & MKI67<1 LEUK	SAMD9
RNF168	5.30E-05	0.395177937	0.211	0.154	1	CD44>1 & MKI67<1 LEUK	RNF168
TFE3	5.99E-05	0.200953739	0.109	0.058	1	CD44>1 & MKI67<1 LEUK	TFE3
CCNL1	6.34E-05	0.446042068	0.507	0.554	1	CD44>1 & MKI67<1 LEUK	CCNL1
SH3GL1	7.32E-05	0.309594752	0.188	0.129	1	CD44>1 & MKI67<1 LEUK	SH3GL1
CSRNP1	7.49E-05	0.457937507	0.235	0.178	1	CD44>1 & MKI67<1 LEUK	CSRNP1
NOP53	7.64E-05	0.278928025	0.754	0.898	1	CD44>1 & MKI67<1 LEUK	NOP53
RSAD2	8.36E-05	0.220583893	0.103	0.054	1	CD44>1 & MKI67<1 LEUK	RSAD2
RPS27	8.45E-05	0.139954813	0.977	0.996	1	CD44>1 & MKI67<1 LEUK	RPS27
RUNX3	0.000100997	0.264724313	0.135	0.081	1	CD44>1 & MKI67<1 LEUK	RUNX3
BACH1	0.000114132	0.292393509	0.155	0.1	1	CD44>1 & MKI67<1 LEUK	BACH1
LAP3	0.000115053	0.330456688	0.229	0.173	1	CD44>1 & MKI67<1 LEUK	LAP3
RYBP	0.000115754	0.231973604	0.109	0.061	1	CD44>1 & MKI67<1 LEUK	RYBP
EPC1	0.000132335	0.408290453	0.455	0.469	1	CD44>1 & MKI67<1 LEUK	EPC1
RASA2	0.000134573	0.240988424	0.152	0.097	1	CD44>1 & MKI67<1 LEUK	RASA2
MIDN	0.0001514	0.246188426	0.185	0.125	1	CD44>1 & MKI67<1 LEUK	MIDN
TRAM1	0.00015773	0.484409724	0.44	0.47	1	CD44>1 & MKI67<1 LEUK	TRAM1
FCGRT	0.000165517	0.365574379	0.311	0.272	1	CD44>1 & MKI67<1 LEUK	FCGRT
OAS3	0.00016579	0.286810965	0.135	0.083	1	CD44>1 & MKI67<1 LEUK	OAS3
TNFSF10	0.000176018	0.306038092	0.15	0.095	1	CD44>1 & MKI67<1 LEUK	TNFSF10
TOX	0.00017602	0.331658396	0.287	0.233	1	CD44>1 & MKI67<1 LEUK	TOX
IFIT5	0.000179695	0.287623469	0.152	0.098	1	CD44>1 & MKI67<1 LEUK	IFIT5
SLC39A8	0.000206799	0.280454632	0.188	0.132	1	CD44>1 & MKI67<1 LEUK	SLC39A8
FAM133B	0.000241849	0.390246767	0.273	0.225	1	CD44>1 & MKI67<1 LEUK	FAM133B
GADD45B	0.000262681	0.325126695	0.129	0.08	1	CD44>1 & MKI67<1 LEUK	GADD45B
CHST11	0.000273254	0.28107384	0.182	0.127	1	CD44>1 & MKI67<1 LEUK	CHST11
BCL2L11	0.000278267	0.190242918	0.1	0.055	1	CD44>1 & MKI67<1 LEUK	BCL2L11
LEPROTL1	0.00031148	0.334400994	0.217	0.166	1	CD44>1 & MKI67<1 LEUK	LEPROTL1
ARID3B	0.000325522	0.224277349	0.109	0.063	1	CD44>1 & MKI67<1 LEUK	ARID3B
EPC2	0.00037935	0.264268025	0.144	0.094	1	CD44>1 & MKI67<1 LEUK	EPC2
SUCO	0.000390699	0.287286319	0.152	0.103	1	CD44>1 & MKI67<1 LEUK	SUCO
HEBP2	0.000390815	0.406096602	0.372	0.361	1	CD44>1 & MKI67<1 LEUK	HEBP2
OSER1	0.000423944	0.338125359	0.196	0.147	1	CD44>1 & MKI67<1 LEUK	OSER1
IFIT2	0.000436418	0.205561427	0.1	0.056	1	CD44>1 & MKI67<1 LEUK	IFIT2
UXS1	0.000510041	0.219329185	0.12	0.075	1	CD44>1 & MKI67<1 LEUK	UXS1
RPS26	0.000514521	0.120565885	0.982	0.999	1	CD44>1 & MKI67<1 LEUK	RPS26
ZCCHC2	0.000537089	0.248050725	0.12	0.074	1	CD44>1 & MKI67<1 LEUK	ZCCHC2
C9orf16	0.000571651	0.303886376	0.548	0.649	1	CD44>1 & MKI67<1 LEUK	C9orf16
PIM1	0.000574918	0.482087466	0.308	0.277	1	CD44>1 & MKI67<1 LEUK	PIM1
USP16	0.000727964	0.375486497	0.273	0.232	1	CD44>1 & MKI67<1 LEUK	USP16
EIF1B	0.000737202	0.33184206	0.361	0.347	1	CD44>1 & MKI67<1 LEUK	EIF1B
SLC1A5	0.000773727	0.284358611	0.173	0.125	1	CD44>1 & MKI67<1 LEUK	SLC1A5
XBP1	0.000809948	0.433736406	0.372	0.361	1	CD44>1 & MKI67<1 LEUK	XBP1
TBCB	0.000933469	0.100384494	0.235	0.391	1	CD44>1 & MKI67<1 LEUK	TBCB
GNG2	0.000937981	0.368900541	0.243	0.202	1	CD44>1 & MKI67<1 LEUK	GNG2
KHDRBS1	0.001018844	0.292219782	0.592	0.696	1	CD44>1 & MKI67<1 LEUK	KHDRBS1

SMAP2	0.001080002	0.425138877	0.449	0.488	1	CD44>1 & MKI67<1 LEUK	SMAP2
ZFP36	0.00112118	0.396747873	0.249	0.206	1	CD44>1 & MKI67<1 LEUK	ZFP36
ST3GAL1	0.001210836	0.29899633	0.194	0.148	1	CD44>1 & MKI67<1 LEUK	ST3GAL1
CHCHD7	0.001213015	0.295803137	0.191	0.145	1	CD44>1 & MKI67<1 LEUK	CHCHD7
COX4I1	0.00124258	0.172475075	0.804	0.909	1	CD44>1 & MKI67<1 LEUK	COX4I1
SELENOK	0.001263938	0.275789987	0.132	0.088	1	CD44>1 & MKI67<1 LEUK	SELENOK
UBL3	0.001264891	0.22246894	0.109	0.068	1	CD44>1 & MKI67<1 LEUK	UBL3
GATA3	0.001332693	0.408081385	0.188	0.144	1	CD44>1 & MKI67<1 LEUK	GATA3
GALNT6	0.001360689	0.402124582	0.249	0.208	1	CD44>1 & MKI67<1 LEUK	GALNT6
CISH	0.001419284	0.347738696	0.185	0.138	1	CD44>1 & MKI67<1 LEUK	CISH
RHOF	0.001461984	0.195632464	0.135	0.09	1	CD44>1 & MKI67<1 LEUK	RHOF
VPS37B	0.001529714	0.329427048	0.229	0.188	1	CD44>1 & MKI67<1 LEUK	VPS37B
FOS	0.001642107	0.212833341	0.152	0.106	1	CD44>1 & MKI67<1 LEUK	FOS
MTRNR2L12	0.001671309	0.242878016	0.716	0.832	1	CD44>1 & MKI67<1 LEUK	MTRNR2L12
PARP8	0.001676825	0.239627477	0.135	0.091	1	CD44>1 & MKI67<1 LEUK	PARP8
HIST1H1E	0.001688665	0.489657286	0.487	0.523	1	CD44>1 & MKI67<1 LEUK	HIST1H1E
OPTN	0.001823067	0.271195166	0.126	0.085	1	CD44>1 & MKI67<1 LEUK	OPTN
SLC15A4	0.002199692	0.280273017	0.144	0.102	1	CD44>1 & MKI67<1 LEUK	SLC15A4
CYTH1	0.002304623	0.192926226	0.123	0.082	1	CD44>1 & MKI67<1 LEUK	CYTH1
SNHG6	0.00230815	0.193661834	0.742	0.863	1	CD44>1 & MKI67<1 LEUK	SNHG6
TRIM25	0.002328161	0.177369007	0.111	0.072	1	CD44>1 & MKI67<1 LEUK	TRIM25
SP100	0.002421197	0.391649899	0.255	0.226	1	CD44>1 & MKI67<1 LEUK	SP100
SLC7A1	0.002435537	0.190029455	0.106	0.067	1	CD44>1 & MKI67<1 LEUK	SLC7A1
PLEC	0.002506043	0.159349296	0.106	0.067	1	CD44>1 & MKI67<1 LEUK	PLEC
ZBTB10	0.002668328	0.297188454	0.17	0.129	1	CD44>1 & MKI67<1 LEUK	ZBTB10
SHFL	0.002736441	0.387302579	0.279	0.249	1	CD44>1 & MKI67<1 LEUK	SHFL
SPRY1	0.002775795	0.350071858	0.211	0.171	1	CD44>1 & MKI67<1 LEUK	SPRY1
UBE2D2	0.002782468	0.330169313	0.534	0.65	1	CD44>1 & MKI67<1 LEUK	UBE2D2
ADNP2	0.002893822	0.250661335	0.141	0.1	1	CD44>1 & MKI67<1 LEUK	ADNP2
B4GALT1	0.002918987	0.29230298	0.208	0.169	1	CD44>1 & MKI67<1 LEUK	B4GALT1
CHCHD2	0.002953909	0.232550769	0.783	0.916	1	CD44>1 & MKI67<1 LEUK	CHCHD2
KRT10	0.002967426	0.392760457	0.405	0.42	1	CD44>1 & MKI67<1 LEUK	KRT10
CMTM6	0.003171328	0.312541129	0.235	0.201	1	CD44>1 & MKI67<1 LEUK	CMTM6
CDS2	0.003186906	0.365540301	0.205	0.168	1	CD44>1 & MKI67<1 LEUK	CDS2
AL135905.2	0.003330317	0.174950741	0.138	0.096	1	CD44>1 & MKI67<1 LEUK	AL135905.2
PCGF1	0.0035264	0.154841335	0.114	0.076	1	CD44>1 & MKI67<1 LEUK	PCGF1
DDAH2	0.003586755	0.344231048	0.24	0.21	1	CD44>1 & MKI67<1 LEUK	DDAH2
CDK17	0.00370715	0.29500536	0.191	0.152	1	CD44>1 & MKI67<1 LEUK	CDK17
MEX3C	0.003779832	0.264119287	0.194	0.153	1	CD44>1 & MKI67<1 LEUK	MEX3C
PPP4R3A	0.003936758	0.104668155	0.1	0.176	1	CD44>1 & MKI67<1 LEUK	PPP4R3A
IKZF1	0.003984576	0.405821627	0.381	0.393	1	CD44>1 & MKI67<1 LEUK	IKZF1
BCL11B	0.003991547	0.355098918	0.302	0.285	1	CD44>1 & MKI67<1 LEUK	BCL11B
ANXA1	0.00401197	0.208036199	0.276	0.425	1	CD44>1 & MKI67<1 LEUK	ANXA1
KRAS	0.004053594	0.341401462	0.328	0.311	1	CD44>1 & MKI67<1 LEUK	KRAS
SFPQ	0.004381186	0.290023754	0.625	0.742	1	CD44>1 & MKI67<1 LEUK	SFPQ
SIK3	0.004511449	0.219541173	0.126	0.088	1	CD44>1 & MKI67<1 LEUK	SIK3
SYNJ2	0.00503518	0.287499542	0.194	0.155	1	CD44>1 & MKI67<1 LEUK	SYNJ2
PLP2	0.005085258	0.410931165	0.44	0.478	1	CD44>1 & MKI67<1 LEUK	PLP2
FAU	0.005086502	0.155568819	0.982	0.992	1	CD44>1 & MKI67<1 LEUK	FAU
C12orf75	0.005116287	0.341129726	0.229	0.198	1	CD44>1 & MKI67<1 LEUK	C12orf75
UBR5	0.005150733	0.306471137	0.208	0.174	1	CD44>1 & MKI67<1 LEUK	UBR5
CCDC69	0.00527371	0.212153382	0.106	0.07	1	CD44>1 & MKI67<1 LEUK	CCDC69
IRF2BPL	0.005337557	0.303227678	0.22	0.186	1	CD44>1 & MKI67<1 LEUK	IRF2BPL
JAK3	0.005438127	0.202450987	0.114	0.078	1	CD44>1 & MKI67<1 LEUK	JAK3

MT-ND2	0.005538921	0.214110088	0.795	0.917	1	CD44>1 & MKI67<1 LEUK	MT-ND2
YWHAB	0.006225446	0.308117407	0.581	0.704	1	CD44>1 & MKI67<1 LEUK	YWHAB
NIPBL	0.006307553	0.10048323	0.158	0.252	1	CD44>1 & MKI67<1 LEUK	NIPBL
SLC3A2	0.006371594	0.348323927	0.475	0.548	1	CD44>1 & MKI67<1 LEUK	SLC3A2
CD47	0.006515569	0.129227014	0.29	0.452	1	CD44>1 & MKI67<1 LEUK	CD47
TWISTNB	0.006959002	0.330396356	0.191	0.158	1	CD44>1 & MKI67<1 LEUK	TWISTNB
PLSCR1	0.006989092	0.308885113	0.205	0.17	1	CD44>1 & MKI67<1 LEUK	PLSCR1
NR3C1	0.007151765	0.123586149	0.328	0.498	1	CD44>1 & MKI67<1 LEUK	NR3C1
ASB8	0.007694236	0.262915042	0.167	0.131	1	CD44>1 & MKI67<1 LEUK	ASB8
HNRNPA0	0.008318389	0.273699491	0.595	0.734	1	CD44>1 & MKI67<1 LEUK	HNRNPA0
MACF1	0.008478843	0.117210831	0.109	0.179	1	CD44>1 & MKI67<1 LEUK	MACF1
SPTBN1	0.008666224	0.410244889	0.352	0.37	1	CD44>1 & MKI67<1 LEUK	SPTBN1
ARFGEF1	0.008931113	0.269433933	0.217	0.188	1	CD44>1 & MKI67<1 LEUK	ARFGEF1
GTPBP4	0.00904185	0.311400862	0.196	0.165	1	CD44>1 & MKI67<1 LEUK	GTPBP4
NEAT1	0.009461802	0.100957732	0.296	0.457	1	CD44>1 & MKI67<1 LEUK	NEAT1
RHOH	0.009724494	0.368281055	0.449	0.51	1	CD44>1 & MKI67<1 LEUK	RHOH
STMN1	1.24E-88	1.768054572	0.834	0.352	4.55E-84	NO SIGNATURE LEUK	STMN1
GSTP1	1.17E-80	1.295747621	0.862	0.431	4.28E-76	NO SIGNATURE LEUK	GSTP1
RPL26	1.07E-73	0.808853771	0.986	0.915	3.90E-69	NO SIGNATURE LEUK	RPL26
RPSA	8.19E-71	0.788752803	0.975	0.824	3.00E-66	NO SIGNATURE LEUK	RPSA
RPLP0	1.82E-64	0.692473749	0.995	0.968	6.67E-60	NO SIGNATURE LEUK	RPLP0
TSC22D3	2.21E-63	1.375441138	0.727	0.24	8.08E-59	NO SIGNATURE LEUK	TSC22D3
SNHG29	5.83E-63	0.912543166	0.924	0.622	2.13E-58	NO SIGNATURE LEUK	SNHG29
DNTT	7.04E-63	0.959718014	0.635	0.109	2.58E-58	NO SIGNATURE LEUK	DNTT
RPS5	3.62E-60	0.678368625	0.986	0.88	1.33E-55	NO SIGNATURE LEUK	RPS5
IMPDH2	1.81E-58	0.810751478	0.627	0.114	6.63E-54	NO SIGNATURE LEUK	IMPDH2
PPIA	7.54E-58	0.756224943	0.971	0.806	2.76E-53	NO SIGNATURE LEUK	PPIA
RPS8	2.39E-57	0.538463435	0.998	0.982	8.74E-53	NO SIGNATURE LEUK	RPS8
RPL41	1.95E-56	0.484564539	1	0.982	7.15E-52	NO SIGNATURE LEUK	RPL41
SELL	4.01E-55	0.834227587	0.553	0.07	1.47E-50	NO SIGNATURE LEUK	SELL
GAPDH	1.98E-53	0.689514117	0.984	0.921	7.24E-49	NO SIGNATURE LEUK	GAPDH
MZB1	6.05E-51	0.786998929	0.762	0.282	2.21E-46	NO SIGNATURE LEUK	MZB1
PFN1	3.43E-50	0.826239482	0.914	0.651	1.26E-45	NO SIGNATURE LEUK	PFN1
TUBA1B	1.82E-46	1.283837533	0.85	0.531	6.67E-42	NO SIGNATURE LEUK	TUBA1B
RAC2	4.71E-43	0.674807696	0.669	0.205	1.72E-38	NO SIGNATURE LEUK	RAC2
TPI1	5.13E-42	0.646548617	0.589	0.147	1.88E-37	NO SIGNATURE LEUK	TPI1
TUBB	2.69E-40	1.202249343	0.787	0.425	9.83E-36	NO SIGNATURE LEUK	TUBB
RPL28	3.57E-40	0.388393089	1	0.991	1.31E-35	NO SIGNATURE LEUK	RPL28
RPS3	6.95E-40	0.398891425	0.999	0.985	2.55E-35	NO SIGNATURE LEUK	RPS3
HSPB1	1.69E-39	0.664136649	0.783	0.355	6.19E-35	NO SIGNATURE LEUK	HSPB1
RACK1	2.24E-39	0.587990012	0.961	0.765	8.20E-35	NO SIGNATURE LEUK	RACK1
PTMS	1.67E-38	0.725337961	0.405	0.035	6.12E-34	NO SIGNATURE LEUK	PTMS
RPL7A	1.65E-37	0.401973647	0.995	0.971	6.05E-33	NO SIGNATURE LEUK	RPL7A
PTCRA	2.99E-37	0.626224434	0.52	0.12	1.09E-32	NO SIGNATURE LEUK	PTCRA
RPS2	9.61E-37	0.465322529	0.995	0.959	3.52E-32	NO SIGNATURE LEUK	RPS2
GABARAP	1.01E-36	0.552488583	0.565	0.15	3.68E-32	NO SIGNATURE LEUK	GABARAP
TRBV7-9	1.90E-36	0.535392922	0.372	0.021	6.96E-32	NO SIGNATURE LEUK	TRBV7-9
SOCS2	1.24E-35	0.516976846	0.559	0.144	4.53E-31	NO SIGNATURE LEUK	SOCS2
SLC29A1	1.33E-35	0.489393064	0.384	0.032	4.87E-31	NO SIGNATURE LEUK	SLC29A1
AP2M1	2.07E-35	0.456067478	0.506	0.111	7.57E-31	NO SIGNATURE LEUK	AP2M1
RPS18	3.18E-35	0.419035619	0.998	0.985	1.17E-30	NO SIGNATURE LEUK	RPS18
PEBP1	2.10E-34	0.546399153	0.656	0.232	7.68E-30	NO SIGNATURE LEUK	PEBP1
OAZ1	3.69E-34	0.570107638	0.766	0.343	1.35E-29	NO SIGNATURE LEUK	OAZ1
ANP32B	8.36E-34	0.595713486	0.725	0.317	3.06E-29	NO SIGNATURE LEUK	ANP32B

LSM4	8.51E-34	0.511469311	0.45	0.088	3.11E-29	NO SIGNATURE LEUK	LSM4
CRIP1	9.64E-34	0.86687392	0.599	0.235	3.53E-29	NO SIGNATURE LEUK	CRIP1
DEK	9.96E-34	0.505114913	0.516	0.132	3.65E-29	NO SIGNATURE LEUK	DEK
DUT	1.90E-33	0.593301454	0.501	0.123	6.95E-29	NO SIGNATURE LEUK	DUT
HMGN2	2.29E-33	0.930530114	0.841	0.522	8.39E-29	NO SIGNATURE LEUK	HMGN2
HMGB2	9.58E-33	1.135202213	0.656	0.296	3.51E-28	NO SIGNATURE LEUK	HMGB2
TAGLN2	1.40E-32	0.599889712	0.807	0.469	5.12E-28	NO SIGNATURE LEUK	TAGLN2
MIF	2.78E-32	0.590362516	0.932	0.713	1.02E-27	NO SIGNATURE LEUK	MIF
RPLP1	1.04E-31	0.375099014	0.999	0.991	3.81E-27	NO SIGNATURE LEUK	RPLP1
H1F0	1.52E-31	0.66175012	0.411	0.07	5.57E-27	NO SIGNATURE LEUK	H1F0
NASP	2.23E-31	0.507196645	0.483	0.123	8.15E-27	NO SIGNATURE LEUK	NASP
CCT3	7.23E-31	0.44027826	0.494	0.123	2.65E-26	NO SIGNATURE LEUK	CCT3
NME2	1.76E-30	0.52714118	0.763	0.349	6.43E-26	NO SIGNATURE LEUK	NME2
POLR2E	2.19E-30	0.354604624	0.39	0.056	8.02E-26	NO SIGNATURE LEUK	POLR2E
NPM1	2.23E-30	0.541623623	0.828	0.46	8.15E-26	NO SIGNATURE LEUK	NPM1
TALDO1	2.25E-30	0.393932142	0.423	0.076	8.23E-26	NO SIGNATURE LEUK	TALDO1
TYMS	1.10E-29	0.727623702	0.435	0.109	4.02E-25	NO SIGNATURE LEUK	TYMS
BANF1	3.52E-29	0.452963842	0.554	0.188	1.29E-24	NO SIGNATURE LEUK	BANF1
SLC25A5	3.65E-29	0.519575087	0.814	0.437	1.34E-24	NO SIGNATURE LEUK	SLC25A5
SNRPA	6.30E-29	0.383665177	0.394	0.067	2.31E-24	NO SIGNATURE LEUK	SNRPA
MEST	9.72E-29	0.467028467	0.363	0.05	3.56E-24	NO SIGNATURE LEUK	MEST
PCBP2	1.16E-28	0.490182352	0.716	0.314	4.25E-24	NO SIGNATURE LEUK	PCBP2
ATP5F1D	1.96E-28	0.403520817	0.594	0.196	7.17E-24	NO SIGNATURE LEUK	ATP5F1D
UQCR10	2.33E-28	0.426980956	0.547	0.176	8.54E-24	NO SIGNATURE LEUK	UQCR10
C1QBP	3.23E-28	0.389346701	0.478	0.123	1.18E-23	NO SIGNATURE LEUK	C1QBP
RPL37A	4.29E-28	0.426958601	0.98	0.842	1.57E-23	NO SIGNATURE LEUK	RPL37A
CCT7	4.46E-28	0.368207635	0.491	0.132	1.63E-23	NO SIGNATURE LEUK	CCT7
FKBP1A	7.88E-28	0.384203822	0.543	0.167	2.89E-23	NO SIGNATURE LEUK	FKBP1A
UHRF1	1.02E-27	0.395274943	0.295	0.015	3.74E-23	NO SIGNATURE LEUK	UHRF1
BRK1	1.16E-27	0.37834539	0.456	0.111	4.24E-23	NO SIGNATURE LEUK	BRK1
ATP5MC2	1.31E-27	0.484040278	0.896	0.589	4.78E-23	NO SIGNATURE LEUK	ATP5MC2
RNASEH2B	3.23E-27	0.389760482	0.472	0.123	1.18E-22	NO SIGNATURE LEUK	RNASEH2B
SELENOW	3.75E-27	0.486890891	0.71	0.328	1.37E-22	NO SIGNATURE LEUK	SELENOW
ZFP36L2	4.77E-27	0.572728484	0.828	0.501	1.75E-22	NO SIGNATURE LEUK	ZFP36L2
TRAPPC1	5.15E-27	0.341199957	0.453	0.111	1.88E-22	NO SIGNATURE LEUK	TRAPPC1
ECH1	5.49E-27	0.327104155	0.335	0.038	2.01E-22	NO SIGNATURE LEUK	ECH1
MCM7	5.61E-27	0.420504919	0.35	0.053	2.05E-22	NO SIGNATURE LEUK	MCM7
NEDD8	5.83E-27	0.366712005	0.477	0.126	2.13E-22	NO SIGNATURE LEUK	NEDD8
PPP1CA	7.84E-27	0.372501581	0.58	0.199	2.87E-22	NO SIGNATURE LEUK	PPP1CA
RPS23	8.15E-27	0.314881444	0.999	0.991	2.98E-22	NO SIGNATURE LEUK	RPS23
XRCC5	8.22E-27	0.366522694	0.453	0.111	3.01E-22	NO SIGNATURE LEUK	XRCC5
RPL15	1.43E-26	0.40492444	0.984	0.871	5.23E-22	NO SIGNATURE LEUK	RPL15
FDFT1	1.64E-26	0.408472643	0.368	0.067	6.01E-22	NO SIGNATURE LEUK	FDFT1
CAT	1.71E-26	0.353317527	0.34	0.047	6.26E-22	NO SIGNATURE LEUK	CAT
DDIT4	1.91E-26	0.524417925	0.651	0.276	7.00E-22	NO SIGNATURE LEUK	DDIT4
SSRP1	5.36E-26	0.339031816	0.323	0.038	1.96E-21	NO SIGNATURE LEUK	SSRP1
MPST	6.52E-26	0.331187465	0.351	0.053	2.39E-21	NO SIGNATURE LEUK	MPST
CLNS1A	7.09E-26	0.336004062	0.347	0.053	2.59E-21	NO SIGNATURE LEUK	CLNS1A
CFTR	8.08E-26	0.371068927	0.353	0.056	2.96E-21	NO SIGNATURE LEUK	CFTR
PHB2	9.34E-26	0.363396744	0.524	0.164	3.42E-21	NO SIGNATURE LEUK	PHB2
MEA1	9.42E-26	0.324415483	0.3	0.026	3.45E-21	NO SIGNATURE LEUK	MEA1
RPA3	1.03E-25	0.335295228	0.285	0.018	3.78E-21	NO SIGNATURE LEUK	RPA3
MCM3	1.33E-25	0.387197622	0.297	0.026	4.87E-21	NO SIGNATURE LEUK	MCM3
SERF2	2.16E-25	0.464189765	0.905	0.613	7.89E-21	NO SIGNATURE LEUK	SERF2

XG	4.21E-25	0.384846611	0.333	0.053	1.54E-20	NO SIGNATURE LEUK	XG
NDUFS8	4.55E-25	0.352116689	0.435	0.111	1.66E-20	NO SIGNATURE LEUK	NDUFS8
CDK2AP2	4.55E-25	0.329792495	0.276	0.015	1.67E-20	NO SIGNATURE LEUK	CDK2AP2
PARP1	5.32E-25	0.339490832	0.341	0.056	1.95E-20	NO SIGNATURE LEUK	PARP1
RUVBL2	1.00E-24	0.305258449	0.285	0.023	3.67E-20	NO SIGNATURE LEUK	RUVBL2
IL2RG	1.44E-24	0.340949078	0.631	0.246	5.28E-20	NO SIGNATURE LEUK	IL2RG
PCLAF	1.47E-24	0.507092884	0.258	0.009	5.38E-20	NO SIGNATURE LEUK	PCLAF
VAMP8	1.83E-24	0.363964773	0.459	0.132	6.69E-20	NO SIGNATURE LEUK	VAMP8
DBI	2.46E-24	0.341220071	0.437	0.114	9.02E-20	NO SIGNATURE LEUK	DBI
ENO1	2.47E-24	0.496076074	0.8	0.46	9.04E-20	NO SIGNATURE LEUK	ENO1
UCP2	2.71E-24	0.349774178	0.36	0.07	9.90E-20	NO SIGNATURE LEUK	UCP2
NUCKS1	3.40E-24	0.426671931	0.486	0.158	1.25E-19	NO SIGNATURE LEUK	NUCKS1
FKBP5	4.10E-24	0.370296409	0.343	0.062	1.50E-19	NO SIGNATURE LEUK	FKBP5
ATP5PO	5.33E-24	0.363664831	0.581	0.22	1.95E-19	NO SIGNATURE LEUK	ATP5PO
HSPD1	5.46E-24	0.425208826	0.532	0.191	2.00E-19	NO SIGNATURE LEUK	HSPD1
TAF15	5.74E-24	0.313862826	0.353	0.067	2.10E-19	NO SIGNATURE LEUK	TAF15
AP2S1	6.91E-24	0.331356411	0.393	0.091	2.53E-19	NO SIGNATURE LEUK	AP2S1
APEX1	7.28E-24	0.349500449	0.485	0.152	2.66E-19	NO SIGNATURE LEUK	APEX1
TKT	7.55E-24	0.382327338	0.657	0.273	2.76E-19	NO SIGNATURE LEUK	TKT
PHB	7.75E-24	0.299482402	0.312	0.041	2.84E-19	NO SIGNATURE LEUK	PHB
RPL36A	9.56E-24	0.408239917	0.975	0.818	3.50E-19	NO SIGNATURE LEUK	RPL36A
COX7C	1.35E-23	0.415018089	0.766	0.378	4.95E-19	NO SIGNATURE LEUK	COX7C
TSTA3	1.89E-23	0.310294754	0.304	0.038	6.93E-19	NO SIGNATURE LEUK	TSTA3
CDK4	2.54E-23	0.305575509	0.275	0.023	9.31E-19	NO SIGNATURE LEUK	CDK4
HCLS1	2.56E-23	0.315962269	0.392	0.094	9.36E-19	NO SIGNATURE LEUK	HCLS1
SAP30	2.90E-23	0.385421557	0.298	0.038	1.06E-18	NO SIGNATURE LEUK	SAP30
PSMD4	2.93E-23	0.291151152	0.314	0.047	1.07E-18	NO SIGNATURE LEUK	PSMD4
TMEM14C	3.72E-23	0.302350666	0.368	0.079	1.36E-18	NO SIGNATURE LEUK	TMEM14C
CORO1A	3.86E-23	0.453745961	0.711	0.361	1.41E-18	NO SIGNATURE LEUK	CORO1A
RPL10A	4.43E-23	0.367729634	0.993	0.9	1.62E-18	NO SIGNATURE LEUK	RPL10A
TUFM	5.44E-23	0.306942234	0.456	0.138	1.99E-18	NO SIGNATURE LEUK	TUFM
PTOV1	5.69E-23	0.268533741	0.33	0.056	2.08E-18	NO SIGNATURE LEUK	PTOV1
TRIM28	6.31E-23	0.326719062	0.481	0.155	2.31E-18	NO SIGNATURE LEUK	TRIM28
LAGE3	8.64E-23	0.293310631	0.281	0.029	3.16E-18	NO SIGNATURE LEUK	LAGE3
TSC22D4	9.51E-23	0.296054723	0.279	0.029	3.48E-18	NO SIGNATURE LEUK	TSC22D4
DYNLL1	9.88E-23	0.340795935	0.466	0.147	3.62E-18	NO SIGNATURE LEUK	DYNLL1
RPS14	1.10E-22	0.305167768	0.993	0.962	4.03E-18	NO SIGNATURE LEUK	RPS14
FADS3	1.11E-22	0.295795679	0.282	0.029	4.05E-18	NO SIGNATURE LEUK	FADS3
TLN1	1.11E-22	0.274555386	0.345	0.065	4.06E-18	NO SIGNATURE LEUK	TLN1
DDX17	1.18E-22	0.373861476	0.543	0.199	4.33E-18	NO SIGNATURE LEUK	DDX17
AHCY	1.26E-22	0.301551201	0.33	0.059	4.63E-18	NO SIGNATURE LEUK	AHCY
POLD2	1.28E-22	0.287697754	0.331	0.062	4.69E-18	NO SIGNATURE LEUK	POLD2
MCM5	1.80E-22	0.364817649	0.317	0.056	6.57E-18	NO SIGNATURE LEUK	MCM5
RPL18A	2.01E-22	0.335895036	0.997	0.974	7.34E-18	NO SIGNATURE LEUK	RPL18A
NDUFB11	2.05E-22	0.299799495	0.602	0.24	7.51E-18	NO SIGNATURE LEUK	NDUFB11
HINT2	2.18E-22	0.279426949	0.279	0.029	7.97E-18	NO SIGNATURE LEUK	HINT2
HP1BP3	2.26E-22	0.359923698	0.56	0.217	8.26E-18	NO SIGNATURE LEUK	HP1BP3
RAD23A	2.70E-22	0.270978053	0.398	0.1	9.87E-18	NO SIGNATURE LEUK	RAD23A
RPL31	2.70E-22	0.415278069	0.958	0.748	9.89E-18	NO SIGNATURE LEUK	RPL31
ANXA6	3.31E-22	0.2806905	0.349	0.07	1.21E-17	NO SIGNATURE LEUK	ANXA6
APBB1IP	3.41E-22	0.2866058	0.35	0.07	1.25E-17	NO SIGNATURE LEUK	APBB1IP
PSMB6	3.52E-22	0.270663706	0.409	0.106	1.29E-17	NO SIGNATURE LEUK	PSMB6
TRMT112	3.53E-22	0.289642247	0.466	0.147	1.29E-17	NO SIGNATURE LEUK	TRMT112
PPM1G	3.79E-22	0.280754927	0.358	0.079	1.39E-17	NO SIGNATURE LEUK	PPM1G

PSME1	4.15E-22	0.344123826	0.691	0.305	1.52E-17	NO SIGNATURE LEUK	PSME1
COPS6	4.53E-22	0.266463484	0.285	0.035	1.66E-17	NO SIGNATURE LEUK	COPS6
PRDX3	4.60E-22	0.271694651	0.279	0.032	1.68E-17	NO SIGNATURE LEUK	PRDX3
GAS2	4.64E-22	0.31594836	0.232	0.006	1.70E-17	NO SIGNATURE LEUK	GAS2
FDPS	4.73E-22	0.284739539	0.316	0.053	1.73E-17	NO SIGNATURE LEUK	FDPS
RPS15	1.17E-21	0.323294515	0.995	0.953	4.30E-17	NO SIGNATURE LEUK	RPS15
UQCRC1	1.24E-21	0.256124095	0.335	0.065	4.53E-17	NO SIGNATURE LEUK	UQCRC1
DNMT1	1.40E-21	0.277637402	0.295	0.044	5.14E-17	NO SIGNATURE LEUK	DNMT1
RPL4	1.54E-21	0.405754921	0.95	0.762	5.65E-17	NO SIGNATURE LEUK	RPL4
MDH1	1.78E-21	0.282998912	0.402	0.111	6.52E-17	NO SIGNATURE LEUK	MDH1
CENPU	1.80E-21	0.337419557	0.33	0.067	6.59E-17	NO SIGNATURE LEUK	CENPU
RCSD1	1.83E-21	0.333387518	0.445	0.138	6.69E-17	NO SIGNATURE LEUK	RCSD1
RPL10	1.92E-21	0.278266023	1	0.985	7.02E-17	NO SIGNATURE LEUK	RPL10
METTL26	2.21E-21	0.292506005	0.415	0.117	8.07E-17	NO SIGNATURE LEUK	METTL26
CSNK2B	2.27E-21	0.337132605	0.599	0.24	8.31E-17	NO SIGNATURE LEUK	CSNK2B
IDH2	2.80E-21	0.277725491	0.392	0.103	1.02E-16	NO SIGNATURE LEUK	IDH2
ATP5PD	3.31E-21	0.269503476	0.349	0.076	1.21E-16	NO SIGNATURE LEUK	ATP5PD
SNRPD2	4.11E-21	0.409198764	0.805	0.452	1.50E-16	NO SIGNATURE LEUK	SNRPD2
NAA38	4.11E-21	0.251144905	0.278	0.035	1.51E-16	NO SIGNATURE LEUK	NAA38
NDUFA11	4.96E-21	0.311662551	0.563	0.223	1.82E-16	NO SIGNATURE LEUK	NDUFA11
PSMA5	5.67E-21	0.245336657	0.301	0.05	2.08E-16	NO SIGNATURE LEUK	PSMA5
RPL13	5.79E-21	0.269292645	0.999	0.991	2.12E-16	NO SIGNATURE LEUK	RPL13
ARPC1B	7.01E-21	0.292105097	0.404	0.114	2.57E-16	NO SIGNATURE LEUK	ARPC1B
PHPT1	7.32E-21	0.323743712	0.342	0.079	2.68E-16	NO SIGNATURE LEUK	PHPT1
PYCR2	1.08E-20	0.263141871	0.242	0.018	3.95E-16	NO SIGNATURE LEUK	PYCR2
ADA	1.61E-20	0.36650104	0.661	0.314	5.88E-16	NO SIGNATURE LEUK	ADA
HSP90AB1	1.90E-20	0.407897681	0.933	0.754	6.94E-16	NO SIGNATURE LEUK	HSP90AB1
CDT1	2.19E-20	0.299857149	0.298	0.053	8.03E-16	NO SIGNATURE LEUK	CDT1
COMT	2.26E-20	0.227450199	0.329	0.067	8.29E-16	NO SIGNATURE LEUK	COMT
SNHG7	2.37E-20	0.281690732	0.349	0.082	8.66E-16	NO SIGNATURE LEUK	SNHG7
FBL	2.90E-20	0.268896186	0.486	0.17	1.06E-15	NO SIGNATURE LEUK	FBL
SOD1	3.41E-20	0.350170224	0.585	0.249	1.25E-15	NO SIGNATURE LEUK	SOD1
RPL3	3.59E-20	0.301989972	0.997	0.977	1.31E-15	NO SIGNATURE LEUK	RPL3
PSIP1	3.61E-20	0.295229251	0.491	0.179	1.32E-15	NO SIGNATURE LEUK	PSIP1
TMEM258	3.78E-20	0.224247019	0.431	0.126	1.38E-15	NO SIGNATURE LEUK	TMEM258
RPL14	3.85E-20	0.309664814	0.988	0.935	1.41E-15	NO SIGNATURE LEUK	RPL14
MPP1	5.32E-20	0.23153698	0.331	0.07	1.95E-15	NO SIGNATURE LEUK	MPP1
NCF4	5.58E-20	0.254435069	0.245	0.023	2.04E-15	NO SIGNATURE LEUK	NCF4
NME4	5.72E-20	0.278217732	0.367	0.097	2.09E-15	NO SIGNATURE LEUK	NME4
ETFA	5.91E-20	0.251192837	0.298	0.056	2.16E-15	NO SIGNATURE LEUK	ETFA
DDX46	5.98E-20	0.235923505	0.339	0.076	2.19E-15	NO SIGNATURE LEUK	DDX46
CNPY3	7.73E-20	0.258172869	0.319	0.067	2.83E-15	NO SIGNATURE LEUK	CNPY3
AKR7A2	9.62E-20	0.245976692	0.24	0.021	3.52E-15	NO SIGNATURE LEUK	AKR7A2
ARPP21	1.31E-19	0.278685721	0.429	0.138	4.79E-15	NO SIGNATURE LEUK	ARPP21
ANP32A	1.36E-19	0.288291107	0.476	0.167	4.99E-15	NO SIGNATURE LEUK	ANP32A
EIF4A1	1.40E-19	0.383196216	0.74	0.405	5.11E-15	NO SIGNATURE LEUK	EIF4A1
LUC7L3	1.67E-19	0.26789399	0.447	0.147	6.10E-15	NO SIGNATURE LEUK	LUC7L3
ANAPC16	1.85E-19	0.251258364	0.449	0.15	6.77E-15	NO SIGNATURE LEUK	ANAPC16
ECHS1	1.97E-19	0.234112061	0.248	0.026	7.22E-15	NO SIGNATURE LEUK	ECHS1
PRKDC	2.12E-19	0.263857634	0.297	0.056	7.75E-15	NO SIGNATURE LEUK	PRKDC
EDF1	2.27E-19	0.279085482	0.561	0.226	8.30E-15	NO SIGNATURE LEUK	EDF1
EEF1G	2.45E-19	0.334891326	0.96	0.833	8.98E-15	NO SIGNATURE LEUK	EEF1G
EVL	3.13E-19	0.330531452	0.611	0.27	1.15E-14	NO SIGNATURE LEUK	EVL
EEF1B2	3.20E-19	0.349861909	0.972	0.865	1.17E-14	NO SIGNATURE LEUK	EEF1B2

RPS4X	3.43E-19	0.247721726	0.999	1	1.26E-14	NO SIGNATURE LEUK	RPS4X
RMI2	3.43E-19	0.242677587	0.211	0.009	1.26E-14	NO SIGNATURE LEUK	RMI2
FKBP3	3.44E-19	0.22603172	0.257	0.032	1.26E-14	NO SIGNATURE LEUK	FKBP3
HES4	3.89E-19	0.314127272	0.456	0.167	1.42E-14	NO SIGNATURE LEUK	HES4
ATP5IF1	3.97E-19	0.26130882	0.474	0.17	1.45E-14	NO SIGNATURE LEUK	ATP5IF1
SELPLG	4.08E-19	0.267512779	0.264	0.038	1.49E-14	NO SIGNATURE LEUK	SELPLG
DARS	4.08E-19	0.223590066	0.34	0.079	1.49E-14	NO SIGNATURE LEUK	DARS
ACADVL	4.12E-19	0.216337926	0.303	0.059	1.51E-14	NO SIGNATURE LEUK	ACADVL
PGLS	4.18E-19	0.262438996	0.346	0.088	1.53E-14	NO SIGNATURE LEUK	PGLS
MRPS26	4.39E-19	0.239374572	0.278	0.047	1.61E-14	NO SIGNATURE LEUK	MRPS26
TBCA	4.41E-19	0.244978822	0.389	0.111	1.61E-14	NO SIGNATURE LEUK	TBCA
PSME2	4.49E-19	0.252004677	0.416	0.135	1.64E-14	NO SIGNATURE LEUK	PSME2
PSMB2	4.55E-19	0.232589664	0.317	0.067	1.66E-14	NO SIGNATURE LEUK	PSMB2
PSMA2	4.69E-19	0.261151325	0.44	0.147	1.72E-14	NO SIGNATURE LEUK	PSMA2
CCDC138	4.71E-19	0.275696892	0.312	0.067	1.72E-14	NO SIGNATURE LEUK	CCDC138
KLHDC3	4.82E-19	0.224576396	0.244	0.026	1.76E-14	NO SIGNATURE LEUK	KLHDC3
ATP5MF	5.16E-19	0.280636077	0.516	0.196	1.89E-14	NO SIGNATURE LEUK	ATP5MF
DMAC1	5.25E-19	0.244492148	0.257	0.035	1.92E-14	NO SIGNATURE LEUK	DMAC1
TMEM131L	5.59E-19	0.237117805	0.331	0.076	2.05E-14	NO SIGNATURE LEUK	TMEM131L
TMUB1	5.90E-19	0.250982688	0.308	0.065	2.16E-14	NO SIGNATURE LEUK	TMUB1
MRPS34	5.98E-19	0.255699597	0.334	0.082	2.19E-14	NO SIGNATURE LEUK	MRPS34
ST13	6.02E-19	0.237105453	0.475	0.164	2.20E-14	NO SIGNATURE LEUK	ST13
LDHB	6.28E-19	0.376806201	0.812	0.496	2.30E-14	NO SIGNATURE LEUK	LDHB
HNRNPR	6.91E-19	0.273440382	0.528	0.202	2.53E-14	NO SIGNATURE LEUK	HNRNPR
NUCB2	7.71E-19	0.350822309	0.767	0.411	2.82E-14	NO SIGNATURE LEUK	NUCB2
CCND2	7.78E-19	0.306494832	0.544	0.217	2.85E-14	NO SIGNATURE LEUK	CCND2
CSTB	7.93E-19	0.290992887	0.581	0.246	2.90E-14	NO SIGNATURE LEUK	CSTB
CAPZB	8.46E-19	0.223416255	0.406	0.123	3.10E-14	NO SIGNATURE LEUK	CAPZB
SNRPE	8.84E-19	0.227819324	0.439	0.147	3.24E-14	NO SIGNATURE LEUK	SNRPE
HADH	8.99E-19	0.22092437	0.22	0.015	3.29E-14	NO SIGNATURE LEUK	HADH
SHMT2	9.29E-19	0.236000712	0.23	0.021	3.40E-14	NO SIGNATURE LEUK	SHMT2
NSG1	9.42E-19	0.248726589	0.291	0.056	3.45E-14	NO SIGNATURE LEUK	NSG1
TECR	1.07E-18	0.231256868	0.335	0.082	3.92E-14	NO SIGNATURE LEUK	TECR
SMARCB1	1.20E-18	0.224792537	0.325	0.076	4.39E-14	NO SIGNATURE LEUK	SMARCB1
POLR2L	1.20E-18	0.266260041	0.433	0.147	4.40E-14	NO SIGNATURE LEUK	POLR2L
ANKRD1	1.27E-18	0.245906592	0.207	0.009	4.65E-14	NO SIGNATURE LEUK	ANKRD1
CLTA	1.30E-18	0.191189976	0.381	0.106	4.76E-14	NO SIGNATURE LEUK	CLTA
BUB3	1.37E-18	0.265087397	0.32	0.073	5.02E-14	NO SIGNATURE LEUK	BUB3
PRDX6	1.42E-18	0.232615257	0.578	0.24	5.21E-14	NO SIGNATURE LEUK	PRDX6
PRMT1	1.55E-18	0.258997499	0.388	0.114	5.67E-14	NO SIGNATURE LEUK	PRMT1
UQCRQ	1.57E-18	0.241153157	0.49	0.182	5.75E-14	NO SIGNATURE LEUK	UQCRQ
TFDP2	1.72E-18	0.230419531	0.386	0.114	6.30E-14	NO SIGNATURE LEUK	TFDP2
THYN1	1.82E-18	0.228741909	0.259	0.038	6.68E-14	NO SIGNATURE LEUK	THYN1
ITPR2	1.91E-18	0.30860166	0.499	0.194	6.99E-14	NO SIGNATURE LEUK	ITPR2
RNASEK	2.00E-18	0.262456637	0.449	0.155	7.30E-14	NO SIGNATURE LEUK	RNASEK
OSTC	2.07E-18	0.244923916	0.409	0.129	7.59E-14	NO SIGNATURE LEUK	OSTC
TSPO	2.18E-18	0.26766165	0.401	0.126	7.99E-14	NO SIGNATURE LEUK	TSPO
PNKD	2.20E-18	0.238882873	0.242	0.029	8.04E-14	NO SIGNATURE LEUK	PNKD
TSC22D1	2.70E-18	0.2543475	0.529	0.211	9.87E-14	NO SIGNATURE LEUK	TSC22D1
AKR1A1	2.72E-18	0.249494448	0.323	0.079	9.96E-14	NO SIGNATURE LEUK	AKR1A1
PRR13	2.75E-18	0.203562881	0.507	0.185	1.01E-13	NO SIGNATURE LEUK	PRR13
NDUFV1	3.28E-18	0.221897094	0.387	0.117	1.20E-13	NO SIGNATURE LEUK	NDUFV1
MRPS24	3.49E-18	0.209072002	0.314	0.07	1.28E-13	NO SIGNATURE LEUK	MRPS24
RPL29	3.73E-18	0.290239073	0.994	0.956	1.36E-13	NO SIGNATURE LEUK	RPL29

PSMB10	4.23E-18	0.226094883	0.328	0.082	1.55E-13	NO SIGNATURE LEUK	PSMB10
CBX5	4.39E-18	0.20089479	0.272	0.047	1.61E-13	NO SIGNATURE LEUK	CBX5
MKI67	4.53E-18	0.467113996	0.185	0	1.66E-13	NO SIGNATURE LEUK	MKI67
PIN1	4.65E-18	0.2256494	0.383	0.111	1.70E-13	NO SIGNATURE LEUK	PIN1
KDELR1	4.91E-18	0.233437255	0.395	0.123	1.80E-13	NO SIGNATURE LEUK	KDELR1
MYL6B	4.91E-18	0.234245055	0.356	0.097	1.80E-13	NO SIGNATURE LEUK	MYL6B
WDR54	4.99E-18	0.228284498	0.237	0.029	1.83E-13	NO SIGNATURE LEUK	WDR54
PDCD5	5.08E-18	0.229914367	0.29	0.059	1.86E-13	NO SIGNATURE LEUK	PDCD5
WDR34	5.30E-18	0.236694178	0.199	0.009	1.94E-13	NO SIGNATURE LEUK	WDR34
ITGAE	5.33E-18	0.209036338	0.398	0.123	1.95E-13	NO SIGNATURE LEUK	ITGAE
LCK	5.44E-18	0.246780153	0.492	0.185	1.99E-13	NO SIGNATURE LEUK	LCK
PSMA6	5.57E-18	0.347187586	0.692	0.34	2.04E-13	NO SIGNATURE LEUK	PSMA6
PAXX	6.41E-18	0.255770999	0.324	0.082	2.34E-13	NO SIGNATURE LEUK	PAXX
FERMT3	6.62E-18	0.222893936	0.237	0.029	2.42E-13	NO SIGNATURE LEUK	FERMT3
CENPX	8.25E-18	0.223240923	0.203	0.012	3.02E-13	NO SIGNATURE LEUK	CENPX
GSDMD	8.26E-18	0.216277171	0.248	0.035	3.02E-13	NO SIGNATURE LEUK	GSDMD
CKS2	8.70E-18	0.376929474	0.247	0.038	3.19E-13	NO SIGNATURE LEUK	CKS2
SSBP4	9.73E-18	0.238728694	0.355	0.1	3.56E-13	NO SIGNATURE LEUK	SSBP4
NDUFB10	1.12E-17	0.232867158	0.421	0.141	4.09E-13	NO SIGNATURE LEUK	NDUFB10
ITM2C	1.15E-17	0.297257418	0.609	0.279	4.21E-13	NO SIGNATURE LEUK	ITM2C
YWHAE	1.19E-17	0.248596375	0.38	0.117	4.34E-13	NO SIGNATURE LEUK	YWHAE
ATP5MC1	1.29E-17	0.232188657	0.348	0.097	4.72E-13	NO SIGNATURE LEUK	ATP5MC1
MAP1A	1.36E-17	0.233729063	0.331	0.085	4.96E-13	NO SIGNATURE LEUK	MAP1A
ZWINT	1.37E-17	0.219796917	0.197	0.009	5.02E-13	NO SIGNATURE LEUK	ZWINT
SNX17	1.38E-17	0.216268909	0.25	0.038	5.06E-13	NO SIGNATURE LEUK	SNX17
DAD1	1.41E-17	0.220285297	0.286	0.059	5.15E-13	NO SIGNATURE LEUK	DAD1
CPNE1	1.43E-17	0.221288836	0.341	0.091	5.25E-13	NO SIGNATURE LEUK	CPNE1
RPL22L1	1.62E-17	0.244032903	0.415	0.141	5.91E-13	NO SIGNATURE LEUK	RPL22L1
CCT4	1.65E-17	0.201610024	0.455	0.161	6.03E-13	NO SIGNATURE LEUK	CCT4
EIF2S3	1.68E-17	0.250737004	0.519	0.205	6.17E-13	NO SIGNATURE LEUK	EIF2S3
TMPO	1.71E-17	0.261629432	0.338	0.094	6.27E-13	NO SIGNATURE LEUK	TMPO
AC109466.1	1.73E-17	0.261868404	0.19	0.006	6.35E-13	NO SIGNATURE LEUK	AC109466.1
DBNDD2	1.82E-17	0.214502439	0.185	0.003	6.68E-13	NO SIGNATURE LEUK	DBNDD2
PLIN3	1.94E-17	0.245458531	0.282	0.059	7.10E-13	NO SIGNATURE LEUK	PLIN3
ATP5MC3	2.05E-17	0.297278669	0.748	0.387	7.49E-13	NO SIGNATURE LEUK	ATP5MC3
PPA1	2.26E-17	0.234810502	0.417	0.141	8.26E-13	NO SIGNATURE LEUK	PPA1
MRPL11	2.26E-17	0.20573326	0.3	0.07	8.26E-13	NO SIGNATURE LEUK	MRPL11
RBMX	2.94E-17	0.235405469	0.425	0.147	1.08E-12	NO SIGNATURE LEUK	RBMX
TXNDC17	2.99E-17	0.228991546	0.241	0.035	1.09E-12	NO SIGNATURE LEUK	TXNDC17
MRPS18B	3.11E-17	0.218526982	0.21	0.018	1.14E-12	NO SIGNATURE LEUK	MRPS18B
SDHC	3.18E-17	0.191287075	0.34	0.091	1.16E-12	NO SIGNATURE LEUK	SDHC
MGST3	3.37E-17	0.214933535	0.279	0.059	1.23E-12	NO SIGNATURE LEUK	MGST3
EIF4A2	3.47E-17	0.194889043	0.463	0.17	1.27E-12	NO SIGNATURE LEUK	EIF4A2
PSMB3	3.63E-17	0.228966956	0.456	0.167	1.33E-12	NO SIGNATURE LEUK	PSMB3
SPCS2	4.12E-17	0.195670436	0.279	0.056	1.51E-12	NO SIGNATURE LEUK	SPCS2
RPL35	4.44E-17	0.298937267	0.983	0.88	1.63E-12	NO SIGNATURE LEUK	RPL35
GRHPR	4.81E-17	0.216292187	0.242	0.038	1.76E-12	NO SIGNATURE LEUK	GRHPR
SMC1A	5.07E-17	0.240513776	0.289	0.065	1.85E-12	NO SIGNATURE LEUK	SMC1A
RPS6	5.17E-17	0.309841171	0.996	0.962	1.89E-12	NO SIGNATURE LEUK	RPS6
SNHG1	5.41E-17	0.22915268	0.352	0.103	1.98E-12	NO SIGNATURE LEUK	SNHG1
CCND3	5.67E-17	0.278511557	0.717	0.349	2.08E-12	NO SIGNATURE LEUK	CCND3
RNF5	7.28E-17	0.198638191	0.212	0.021	2.67E-12	NO SIGNATURE LEUK	RNF5
PABPN1	8.20E-17	0.236705157	0.587	0.261	3.00E-12	NO SIGNATURE LEUK	PABPN1
YIF1B	8.30E-17	0.199536476	0.196	0.012	3.04E-12	NO SIGNATURE LEUK	YIF1B

SAE1	8.35E-17	0.200124036	0.184	0.006	3.06E-12	NO SIGNATURE LEUK	SAE1
SHQ1	9.40E-17	0.212998181	0.189	0.009	3.44E-12	NO SIGNATURE LEUK	SHQ1
PARK7	9.55E-17	0.236879479	0.548	0.229	3.49E-12	NO SIGNATURE LEUK	PARK7
NDUFA7	9.71E-17	0.192477928	0.326	0.085	3.55E-12	NO SIGNATURE LEUK	NDUFA7
PAFAH1B3	9.86E-17	0.198908095	0.275	0.059	3.61E-12	NO SIGNATURE LEUK	PAFAH1B3
TMEM106C	1.01E-16	0.221798959	0.194	0.012	3.71E-12	NO SIGNATURE LEUK	TMEM106C
NUSAP1	1.03E-16	0.372461996	0.172	0	3.76E-12	NO SIGNATURE LEUK	NUSAP1
RPS12	1.10E-16	0.288724709	0.998	0.988	4.03E-12	NO SIGNATURE LEUK	RPS12
PIH1D1	1.17E-16	0.191965574	0.241	0.038	4.28E-12	NO SIGNATURE LEUK	PIH1D1
TMEM256	1.29E-16	0.198708767	0.21	0.021	4.72E-12	NO SIGNATURE LEUK	TMEM256
RPS3A	1.37E-16	0.248512263	0.999	0.988	5.01E-12	NO SIGNATURE LEUK	RPS3A
DCTPP1	1.55E-16	0.217280912	0.237	0.038	5.68E-12	NO SIGNATURE LEUK	DCTPP1
CKS1B	1.65E-16	0.262169181	0.217	0.026	6.03E-12	NO SIGNATURE LEUK	CKS1B
NME1	1.65E-16	0.223174278	0.371	0.12	6.04E-12	NO SIGNATURE LEUK	NME1
PXMP2	1.66E-16	0.206943311	0.223	0.029	6.09E-12	NO SIGNATURE LEUK	PXMP2
DDX39B	1.67E-16	0.242926536	0.447	0.164	6.11E-12	NO SIGNATURE LEUK	DDX39B
SMOX	1.71E-16	0.236826621	0.288	0.067	6.26E-12	NO SIGNATURE LEUK	SMOX
MRPL38	1.73E-16	0.196145629	0.242	0.038	6.33E-12	NO SIGNATURE LEUK	MRPL38
PDAP1	1.86E-16	0.199300575	0.268	0.056	6.81E-12	NO SIGNATURE LEUK	PDAP1
AF064858.1	1.88E-16	0.239046551	0.239	0.038	6.87E-12	NO SIGNATURE LEUK	AF064858.1
CFH	1.88E-16	0.215651738	0.175	0.003	6.88E-12	NO SIGNATURE LEUK	CFH
SEPTIN6	2.28E-16	0.324304237	0.82	0.499	8.34E-12	NO SIGNATURE LEUK	SEPTIN6
TM7SF3	2.44E-16	0.224930609	0.268	0.056	8.92E-12	NO SIGNATURE LEUK	TM7SF3
PSMC5	2.53E-16	0.205888052	0.325	0.091	9.25E-12	NO SIGNATURE LEUK	PSMC5
SND1	2.56E-16	0.212906588	0.248	0.044	9.35E-12	NO SIGNATURE LEUK	SND1
RAN	2.65E-16	0.339073991	0.754	0.434	9.71E-12	NO SIGNATURE LEUK	RAN
ETFB	2.79E-16	0.216481287	0.356	0.114	1.02E-11	NO SIGNATURE LEUK	ETFB
ATIC	2.79E-16	0.215195038	0.247	0.044	1.02E-11	NO SIGNATURE LEUK	ATIC
CUEDC2	3.06E-16	0.185146459	0.306	0.076	1.12E-11	NO SIGNATURE LEUK	CUEDC2
DCXR	3.08E-16	0.17862083	0.244	0.041	1.13E-11	NO SIGNATURE LEUK	DCXR
SCCPDH	3.22E-16	0.19779854	0.255	0.05	1.18E-11	NO SIGNATURE LEUK	SCCPDH
ARRB2	3.61E-16	0.204565712	0.266	0.056	1.32E-11	NO SIGNATURE LEUK	ARRB2
RPN2	4.11E-16	0.213333815	0.384	0.132	1.50E-11	NO SIGNATURE LEUK	RPN2
TCF7	4.18E-16	0.275231243	0.56	0.255	1.53E-11	NO SIGNATURE LEUK	TCF7
TRAF3IP3	4.47E-16	0.216568561	0.262	0.053	1.64E-11	NO SIGNATURE LEUK	TRAF3IP3
XRCC6	5.03E-16	0.216891701	0.529	0.22	1.84E-11	NO SIGNATURE LEUK	XRCC6
RPS25	5.26E-16	0.299506969	0.936	0.68	1.92E-11	NO SIGNATURE LEUK	RPS25
MZT2A	5.48E-16	0.203911818	0.465	0.176	2.00E-11	NO SIGNATURE LEUK	MZT2A
ATRX	5.53E-16	0.215748858	0.301	0.079	2.02E-11	NO SIGNATURE LEUK	ATRX
GADD45GIP1	5.65E-16	0.195392054	0.306	0.079	2.07E-11	NO SIGNATURE LEUK	GADD45GIP1
SCP2	6.07E-16	0.212960789	0.361	0.111	2.22E-11	NO SIGNATURE LEUK	SCP2
MRPL57	6.29E-16	0.219569151	0.431	0.161	2.30E-11	NO SIGNATURE LEUK	MRPL57
PRDX1	6.89E-16	0.2098262	0.407	0.147	2.52E-11	NO SIGNATURE LEUK	PRDX1
WAS	6.93E-16	0.176267927	0.272	0.059	2.54E-11	NO SIGNATURE LEUK	WAS
NUDT1	7.09E-16	0.184692365	0.322	0.091	2.60E-11	NO SIGNATURE LEUK	NUDT1
INO80E	7.18E-16	0.199555542	0.213	0.026	2.63E-11	NO SIGNATURE LEUK	INO80E
POLR3GL	7.35E-16	0.21243982	0.201	0.021	2.69E-11	NO SIGNATURE LEUK	POLR3GL
LAMTOR2	7.52E-16	0.201734912	0.207	0.023	2.75E-11	NO SIGNATURE LEUK	LAMTOR2
OCIAD1	7.57E-16	0.190104026	0.39	0.132	2.77E-11	NO SIGNATURE LEUK	OCIAD1
COX5B	7.81E-16	0.218168188	0.554	0.238	2.86E-11	NO SIGNATURE LEUK	COX5B
MLLT11	8.09E-16	0.200470845	0.174	0.006	2.96E-11	NO SIGNATURE LEUK	MLLT11
NONO	1.01E-15	0.184853707	0.417	0.147	3.71E-11	NO SIGNATURE LEUK	NONO
HPRT1	1.05E-15	0.167155635	0.278	0.065	3.86E-11	NO SIGNATURE LEUK	HPRT1
MRPL34	1.06E-15	0.190071254	0.211	0.026	3.86E-11	NO SIGNATURE LEUK	MRPL34

FEN1	1.15E-15	0.214553489	0.195	0.018	4.21E-11	NO SIGNATURE LEUK	FEN1
SEC13	1.18E-15	0.208520888	0.334	0.1	4.32E-11	NO SIGNATURE LEUK	SEC13
UROD	1.18E-15	0.179726529	0.207	0.023	4.34E-11	NO SIGNATURE LEUK	UROD
SF3B2	1.25E-15	0.162308572	0.53	0.217	4.58E-11	NO SIGNATURE LEUK	SF3B2
ACAP1	1.30E-15	0.165662556	0.361	0.114	4.74E-11	NO SIGNATURE LEUK	ACAP1
DNAJC8	1.33E-15	0.168870946	0.366	0.114	4.87E-11	NO SIGNATURE LEUK	DNAJC8
MRPL14	1.41E-15	0.19439398	0.236	0.041	5.15E-11	NO SIGNATURE LEUK	MRPL14
COMMD4	1.41E-15	0.197450588	0.202	0.023	5.17E-11	NO SIGNATURE LEUK	COMMD4
MAP2K2	1.44E-15	0.20462412	0.361	0.117	5.27E-11	NO SIGNATURE LEUK	MAP2K2
AK2	1.56E-15	0.177738014	0.205	0.023	5.72E-11	NO SIGNATURE LEUK	AK2
SMC3	1.60E-15	0.178862227	0.308	0.085	5.85E-11	NO SIGNATURE LEUK	SMC3
MRPS21	1.63E-15	0.186719732	0.398	0.138	5.95E-11	NO SIGNATURE LEUK	MRPS21
SNRNP70	1.65E-15	0.209331564	0.423	0.158	6.03E-11	NO SIGNATURE LEUK	SNRNP70
UBXN1	1.68E-15	0.189667587	0.42	0.15	6.16E-11	NO SIGNATURE LEUK	UBXN1
HMGN3	1.72E-15	0.208915421	0.546	0.24	6.28E-11	NO SIGNATURE LEUK	HMGN3
PSMB8	1.81E-15	0.178521699	0.369	0.12	6.62E-11	NO SIGNATURE LEUK	PSMB8
NUDC	1.83E-15	0.153332976	0.406	0.144	6.71E-11	NO SIGNATURE LEUK	NUDC
LMNB1	1.94E-15	0.187837767	0.244	0.047	7.09E-11	NO SIGNATURE LEUK	LMNB1
PRPS2	1.94E-15	0.211301705	0.222	0.035	7.11E-11	NO SIGNATURE LEUK	PRPS2
STOML2	2.12E-15	0.199723363	0.351	0.111	7.77E-11	NO SIGNATURE LEUK	STOML2
PYCARD	2.20E-15	0.209117602	0.239	0.044	8.06E-11	NO SIGNATURE LEUK	PYCARD
UQCC2	2.31E-15	0.185731495	0.169	0.006	8.47E-11	NO SIGNATURE LEUK	UQCC2
TCF25	2.33E-15	0.201549029	0.17	0.006	8.51E-11	NO SIGNATURE LEUK	TCF25
SMPD3	2.49E-15	0.22561777	0.305	0.088	9.12E-11	NO SIGNATURE LEUK	SMPD3
NAP1L4	2.53E-15	0.17042326	0.341	0.103	9.25E-11	NO SIGNATURE LEUK	NAP1L4
SRP9	2.58E-15	0.24792969	0.544	0.243	9.44E-11	NO SIGNATURE LEUK	SRP9
NDUFB2	2.60E-15	0.187313799	0.455	0.182	9.52E-11	NO SIGNATURE LEUK	NDUFB2
ATP6V0E2	2.62E-15	0.185692709	0.308	0.085	9.61E-11	NO SIGNATURE LEUK	ATP6V0E2
SMC2	2.79E-15	0.187407032	0.175	0.009	1.02E-10	NO SIGNATURE LEUK	SMC2
CDCA7	2.85E-15	0.224618239	0.3	0.082	1.04E-10	NO SIGNATURE LEUK	CDCA7
H2AFZ	2.88E-15	0.508488231	0.762	0.493	1.05E-10	NO SIGNATURE LEUK	H2AFZ
AKR1B1	3.48E-15	0.171674413	0.247	0.05	1.27E-10	NO SIGNATURE LEUK	AKR1B1
GDI2	3.85E-15	0.180729558	0.307	0.085	1.41E-10	NO SIGNATURE LEUK	GDI2
HSPA8	3.85E-15	0.35201119	0.712	0.396	1.41E-10	NO SIGNATURE LEUK	HSPA8
POLR2G	3.94E-15	0.188841473	0.372	0.126	1.44E-10	NO SIGNATURE LEUK	POLR2G
RBBP4	3.99E-15	0.168500439	0.365	0.123	1.46E-10	NO SIGNATURE LEUK	RBBP4
CUTA	4.03E-15	0.154042314	0.511	0.211	1.48E-10	NO SIGNATURE LEUK	CUTA
GSS	4.33E-15	0.182399432	0.173	0.009	1.59E-10	NO SIGNATURE LEUK	GSS
POLD4	4.54E-15	0.191339021	0.231	0.041	1.66E-10	NO SIGNATURE LEUK	POLD4
NRDC	4.89E-15	0.204540049	0.255	0.056	1.79E-10	NO SIGNATURE LEUK	NRDC
ARL2	4.90E-15	0.180042783	0.171	0.009	1.79E-10	NO SIGNATURE LEUK	ARL2
CENPF	4.93E-15	0.454057843	0.18	0.015	1.81E-10	NO SIGNATURE LEUK	CENPF
MGMT	5.01E-15	0.166713891	0.282	0.07	1.83E-10	NO SIGNATURE LEUK	MGMT
TK1	5.34E-15	0.274488156	0.155	0	1.95E-10	NO SIGNATURE LEUK	TK1
THRAP3	5.54E-15	0.194624857	0.387	0.135	2.03E-10	NO SIGNATURE LEUK	THRAP3
COX6B1	5.61E-15	0.246621313	0.693	0.346	2.05E-10	NO SIGNATURE LEUK	COX6B1
UBA1	5.75E-15	0.194771724	0.181	0.015	2.11E-10	NO SIGNATURE LEUK	UBA1
MCM2	5.86E-15	0.18647857	0.194	0.021	2.14E-10	NO SIGNATURE LEUK	MCM2
SORBS3	6.61E-15	0.186172934	0.159	0.003	2.42E-10	NO SIGNATURE LEUK	SORBS3
UFC1	7.95E-15	0.1969693	0.428	0.161	2.91E-10	NO SIGNATURE LEUK	UFC1
QARS	8.09E-15	0.227221154	0.347	0.114	2.96E-10	NO SIGNATURE LEUK	QARS
NDUFB8	8.99E-15	0.234523471	0.527	0.235	3.29E-10	NO SIGNATURE LEUK	NDUFB8
CTBP1	9.23E-15	0.174332463	0.333	0.103	3.38E-10	NO SIGNATURE LEUK	CTBP1
ACADM	1.05E-14	0.179835006	0.217	0.035	3.86E-10	NO SIGNATURE LEUK	ACADM

MRPL52	1.09E-14	0.198368749	0.214	0.035	3.99E-10	NO SIGNATURE LEUK	MRPL52
ACYP1	1.16E-14	0.175239387	0.157	0.003	4.24E-10	NO SIGNATURE LEUK	ACYP1
NPPC	1.19E-14	0.277598954	0.178	0.015	4.35E-10	NO SIGNATURE LEUK	NPPC
NDUFB4	1.22E-14	0.173819711	0.379	0.132	4.46E-10	NO SIGNATURE LEUK	NDUFB4
BAX	1.27E-14	0.200645499	0.466	0.191	4.65E-10	NO SIGNATURE LEUK	BAX
UBL7	1.36E-14	0.153151027	0.294	0.082	4.98E-10	NO SIGNATURE LEUK	UBL7
GABARAPL2	1.40E-14	0.168937809	0.271	0.065	5.11E-10	NO SIGNATURE LEUK	GABARAPL2
GXYLT2	1.40E-14	0.199091671	0.385	0.138	5.14E-10	NO SIGNATURE LEUK	GXYLT2
SMIM3	1.47E-14	0.192730581	0.395	0.144	5.38E-10	NO SIGNATURE LEUK	SMIM3
PGD	1.49E-14	0.157947666	0.326	0.1	5.45E-10	NO SIGNATURE LEUK	PGD
CLPP	1.56E-14	0.180100863	0.445	0.176	5.70E-10	NO SIGNATURE LEUK	CLPP
PPP2R1A	1.96E-14	0.168748628	0.361	0.123	7.17E-10	NO SIGNATURE LEUK	PPP2R1A
PFKL	2.12E-14	0.174628002	0.183	0.018	7.74E-10	NO SIGNATURE LEUK	PFKL
HNRNPF	2.16E-14	0.199551132	0.426	0.167	7.92E-10	NO SIGNATURE LEUK	HNRNPF
CHCHD3	2.23E-14	0.146773304	0.216	0.035	8.17E-10	NO SIGNATURE LEUK	CHCHD3
UBE2L6	2.29E-14	0.171282522	0.331	0.103	8.40E-10	NO SIGNATURE LEUK	UBE2L6
MPG	2.31E-14	0.174752858	0.255	0.059	8.46E-10	NO SIGNATURE LEUK	MPG
EIF4EBP1	2.34E-14	0.17673124	0.159	0.006	8.55E-10	NO SIGNATURE LEUK	EIF4EBP1
RNF167	2.35E-14	0.180118958	0.224	0.041	8.62E-10	NO SIGNATURE LEUK	RNF167
CDCA7L	2.35E-14	0.175381638	0.177	0.015	8.62E-10	NO SIGNATURE LEUK	CDCA7L
BABAM1	2.44E-14	0.140373887	0.187	0.021	8.93E-10	NO SIGNATURE LEUK	BABAM1
SRM	2.55E-14	0.214944663	0.326	0.106	9.34E-10	NO SIGNATURE LEUK	SRM
DCAF7	2.57E-14	0.169147157	0.199	0.026	9.42E-10	NO SIGNATURE LEUK	DCAF7
CALR	2.87E-14	0.217600975	0.53	0.243	1.05E-09	NO SIGNATURE LEUK	CALR
ARPC3	3.03E-14	0.248104353	0.66	0.343	1.11E-09	NO SIGNATURE LEUK	ARPC3
PSMA4	3.05E-14	0.159326381	0.298	0.085	1.12E-09	NO SIGNATURE LEUK	PSMA4
ESD	3.05E-14	0.163424164	0.385	0.138	1.12E-09	NO SIGNATURE LEUK	ESD
NDUFA13	3.20E-14	0.232140991	0.706	0.37	1.17E-09	NO SIGNATURE LEUK	NDUFA13
EIF4B	3.20E-14	0.206979156	0.516	0.223	1.17E-09	NO SIGNATURE LEUK	EIF4B
RBBP7	3.24E-14	0.176633872	0.338	0.111	1.19E-09	NO SIGNATURE LEUK	RBBP7
BEX3	3.25E-14	0.18171788	0.446	0.182	1.19E-09	NO SIGNATURE LEUK	BEX3
CENPW	3.28E-14	0.177174831	0.157	0.006	1.20E-09	NO SIGNATURE LEUK	CENPW
GPI	3.45E-14	0.182656442	0.26	0.065	1.26E-09	NO SIGNATURE LEUK	GPI
AL138899.1	3.52E-14	0.195561072	0.191	0.023	1.29E-09	NO SIGNATURE LEUK	AL138899.1
CR2	3.53E-14	0.18802188	0.175	0.015	1.29E-09	NO SIGNATURE LEUK	CR2
EPRS	3.55E-14	0.147482117	0.212	0.035	1.30E-09	NO SIGNATURE LEUK	EPRS
TMEM230	3.80E-14	0.166051736	0.297	0.085	1.39E-09	NO SIGNATURE LEUK	TMEM230
ITGA4	4.04E-14	0.212411363	0.552	0.252	1.48E-09	NO SIGNATURE LEUK	ITGA4
NDUFB3	4.09E-14	0.163606532	0.208	0.032	1.50E-09	NO SIGNATURE LEUK	NDUFB3
ERP29	4.23E-14	0.148385004	0.434	0.17	1.55E-09	NO SIGNATURE LEUK	ERP29
MDH2	4.26E-14	0.188124616	0.557	0.249	1.56E-09	NO SIGNATURE LEUK	MDH2
CMTM7	4.46E-14	0.189414441	0.279	0.076	1.63E-09	NO SIGNATURE LEUK	CMTM7
MAD2L1	4.54E-14	0.163106367	0.163	0.009	1.66E-09	NO SIGNATURE LEUK	MAD2L1
CAPG	4.62E-14	0.180667839	0.24	0.053	1.69E-09	NO SIGNATURE LEUK	CAPG
RTRAF	4.67E-14	0.191621269	0.546	0.246	1.71E-09	NO SIGNATURE LEUK	RTRAF
ARPC4	4.80E-14	0.15771199	0.298	0.085	1.76E-09	NO SIGNATURE LEUK	ARPC4
RPL19	4.89E-14	0.249004685	0.997	0.971	1.79E-09	NO SIGNATURE LEUK	RPL19
DDT	5.28E-14	0.177318759	0.205	0.032	1.93E-09	NO SIGNATURE LEUK	DDT
RPN1	5.36E-14	0.146627202	0.231	0.047	1.96E-09	NO SIGNATURE LEUK	RPN1
ATP5F1A	5.63E-14	0.21094553	0.632	0.311	2.06E-09	NO SIGNATURE LEUK	ATP5F1A
PDCD6	5.67E-14	0.145556293	0.344	0.114	2.08E-09	NO SIGNATURE LEUK	PDCD6
AP1S1	5.69E-14	0.161073533	0.185	0.021	2.08E-09	NO SIGNATURE LEUK	AP1S1
GALNT2	5.93E-14	0.209603999	0.45	0.188	2.17E-09	NO SIGNATURE LEUK	GALNT2
FKBP8	7.03E-14	0.18223899	0.506	0.214	2.57E-09	NO SIGNATURE LEUK	FKBP8

RPL23A	7.05E-14	0.267756565	0.974	0.842	2.58E-09	NO SIGNATURE LEUK	RPL23A
CCDC167	7.33E-14	0.178875687	0.187	0.023	2.68E-09	NO SIGNATURE LEUK	CCDC167
HDAC1	7.51E-14	0.17345899	0.296	0.088	2.75E-09	NO SIGNATURE LEUK	HDAC1
WASHC3	8.28E-14	0.173798897	0.225	0.044	3.03E-09	NO SIGNATURE LEUK	WASHC3
BCKDHA	8.66E-14	0.175831507	0.186	0.023	3.17E-09	NO SIGNATURE LEUK	BCKDHA
VAMP2	9.03E-14	0.207789693	0.488	0.214	3.30E-09	NO SIGNATURE LEUK	VAMP2
SEPTIN11	9.05E-14	0.169158029	0.195	0.029	3.31E-09	NO SIGNATURE LEUK	SEPTIN11
CIAO2A	9.35E-14	0.142643692	0.236	0.05	3.42E-09	NO SIGNATURE LEUK	CIAO2A
MRPL16	9.83E-14	0.150344746	0.219	0.041	3.60E-09	NO SIGNATURE LEUK	MRPL16
PSMB4	1.01E-13	0.180309345	0.256	0.065	3.70E-09	NO SIGNATURE LEUK	PSMB4
RAD50	1.02E-13	0.181170516	0.18	0.021	3.72E-09	NO SIGNATURE LEUK	RAD50
C19orf48	1.11E-13	0.160281885	0.283	0.082	4.06E-09	NO SIGNATURE LEUK	C19orf48
PSMD8	1.12E-13	0.166533798	0.424	0.167	4.12E-09	NO SIGNATURE LEUK	PSMD8
PPP2R3B	1.18E-13	0.156692968	0.181	0.021	4.31E-09	NO SIGNATURE LEUK	PPP2R3B
POLR2I	1.19E-13	0.172647992	0.319	0.103	4.34E-09	NO SIGNATURE LEUK	POLR2I
HES1	1.24E-13	0.229868649	0.327	0.109	4.55E-09	NO SIGNATURE LEUK	HES1
ACBD6	1.38E-13	0.154161903	0.244	0.056	5.04E-09	NO SIGNATURE LEUK	ACBD6
COA3	1.51E-13	0.161675132	0.168	0.015	5.54E-09	NO SIGNATURE LEUK	COA3
CARHSP1	1.52E-13	0.149951618	0.321	0.106	5.56E-09	NO SIGNATURE LEUK	CARHSP1
DHFR	1.53E-13	0.176973166	0.169	0.015	5.60E-09	NO SIGNATURE LEUK	DHFR
CNPY2	1.65E-13	0.161091898	0.191	0.026	6.03E-09	NO SIGNATURE LEUK	CNPY2
HDGF	1.75E-13	0.192893197	0.332	0.114	6.39E-09	NO SIGNATURE LEUK	HDGF
CBX1	1.77E-13	0.153422491	0.162	0.012	6.49E-09	NO SIGNATURE LEUK	CBX1
RPS21	1.81E-13	0.251481309	0.982	0.894	6.61E-09	NO SIGNATURE LEUK	RPS21
PLEKHJ1	1.97E-13	0.151610047	0.247	0.059	7.20E-09	NO SIGNATURE LEUK	PLEKHJ1
PAXIP1-AS1	1.98E-13	0.176649705	0.238	0.056	7.23E-09	NO SIGNATURE LEUK	PAXIP1-AS1
PPP1R14B	2.06E-13	0.231241814	0.571	0.279	7.53E-09	NO SIGNATURE LEUK	PPP1R14B
HNRNPK	2.11E-13	0.215757023	0.694	0.358	7.72E-09	NO SIGNATURE LEUK	HNRNPK
CD1B	2.11E-13	0.194449072	0.232	0.053	7.72E-09	NO SIGNATURE LEUK	CD1B
LSM7	2.30E-13	0.221847132	0.549	0.261	8.42E-09	NO SIGNATURE LEUK	LSM7
TPGS2	2.34E-13	0.159602655	0.251	0.062	8.55E-09	NO SIGNATURE LEUK	TPGS2
LTA4H	2.37E-13	0.153668139	0.255	0.065	8.68E-09	NO SIGNATURE LEUK	LTA4H
RAB13	2.42E-13	0.170642518	0.155	0.009	8.86E-09	NO SIGNATURE LEUK	RAB13
UNG	2.45E-13	0.187640439	0.148	0.006	8.95E-09	NO SIGNATURE LEUK	UNG
CNTLN	2.57E-13	0.170318691	0.155	0.009	9.40E-09	NO SIGNATURE LEUK	CNTLN
BIN2	2.59E-13	0.136244074	0.312	0.1	9.49E-09	NO SIGNATURE LEUK	BIN2
UBE2C	2.70E-13	0.452369156	0.148	0.006	9.88E-09	NO SIGNATURE LEUK	UBE2C
COX5A	2.71E-13	0.190311972	0.543	0.246	9.91E-09	NO SIGNATURE LEUK	COX5A
PSMD3	2.79E-13	0.136593601	0.21	0.038	1.02E-08	NO SIGNATURE LEUK	PSMD3
DHRS4	2.88E-13	0.143608164	0.203	0.035	1.06E-08	NO SIGNATURE LEUK	DHRS4
NSD3	3.21E-13	0.149409282	0.267	0.073	1.18E-08	NO SIGNATURE LEUK	NSD3
QTRT1	3.23E-13	0.16327611	0.185	0.026	1.18E-08	NO SIGNATURE LEUK	QTRT1
SDHA	3.57E-13	0.136159551	0.24	0.056	1.31E-08	NO SIGNATURE LEUK	SDHA
EIF3G	3.59E-13	0.145332861	0.548	0.246	1.31E-08	NO SIGNATURE LEUK	EIF3G
UQCR11	3.68E-13	0.150125154	0.529	0.238	1.35E-08	NO SIGNATURE LEUK	UQCR11
TOMM7	3.68E-13	0.188178081	0.563	0.267	1.35E-08	NO SIGNATURE LEUK	TOMM7
PHGDH	3.79E-13	0.155607461	0.229	0.053	1.39E-08	NO SIGNATURE LEUK	PHGDH
IK	3.89E-13	0.153739136	0.316	0.103	1.43E-08	NO SIGNATURE LEUK	IK
ATP5PF	3.92E-13	0.180100165	0.563	0.261	1.43E-08	NO SIGNATURE LEUK	ATP5PF
NUDT8	3.93E-13	0.166149006	0.152	0.009	1.44E-08	NO SIGNATURE LEUK	NUDT8
DTYMK	3.95E-13	0.153473038	0.201	0.035	1.44E-08	NO SIGNATURE LEUK	DTYMK
PCM1	4.17E-13	0.166823872	0.339	0.12	1.53E-08	NO SIGNATURE LEUK	PCM1
FAM111A	4.23E-13	0.136348423	0.165	0.015	1.55E-08	NO SIGNATURE LEUK	FAM111A
ANAPC5	4.30E-13	0.155470646	0.326	0.111	1.57E-08	NO SIGNATURE LEUK	ANAPC5

POLR2H	4.32E-13	0.168925403	0.204	0.038	1.58E-08	NO SIGNATURE LEUK	POLR2H
UBA2	4.46E-13	0.1720857	0.249	0.065	1.63E-08	NO SIGNATURE LEUK	UBA2
PSMC3	4.50E-13	0.128916294	0.347	0.123	1.65E-08	NO SIGNATURE LEUK	PSMC3
STMP1	4.77E-13	0.134803418	0.317	0.103	1.75E-08	NO SIGNATURE LEUK	STMP1
AC011893.1	4.82E-13	0.163179439	0.157	0.012	1.76E-08	NO SIGNATURE LEUK	AC011893.1
PSMA1	5.23E-13	0.166220643	0.503	0.223	1.91E-08	NO SIGNATURE LEUK	PSMA1
RABL6	5.50E-13	0.149330841	0.205	0.038	2.01E-08	NO SIGNATURE LEUK	RABL6
IL7R	5.56E-13	0.203182433	0.366	0.141	2.03E-08	NO SIGNATURE LEUK	IL7R
RRM1	5.67E-13	0.145148982	0.158	0.012	2.07E-08	NO SIGNATURE LEUK	RRM1
SH3BGRL	5.82E-13	0.133592175	0.392	0.15	2.13E-08	NO SIGNATURE LEUK	SH3BGRL
SNRPF	5.84E-13	0.173501482	0.5	0.223	2.14E-08	NO SIGNATURE LEUK	SNRPF
TRIM47	6.23E-13	0.146287826	0.156	0.012	2.28E-08	NO SIGNATURE LEUK	TRIM47
HSPE1	6.29E-13	0.171922195	0.369	0.141	2.30E-08	NO SIGNATURE LEUK	HSPE1
NUBP2	6.36E-13	0.155894655	0.198	0.035	2.33E-08	NO SIGNATURE LEUK	NUBP2
VPS51	6.64E-13	0.149445241	0.285	0.088	2.43E-08	NO SIGNATURE LEUK	VPS51
SMDT1	6.67E-13	0.178102923	0.505	0.229	2.44E-08	NO SIGNATURE LEUK	SMDT1
KXD1	6.70E-13	0.128282609	0.263	0.073	2.45E-08	NO SIGNATURE LEUK	KXD1
RGS10	6.73E-13	0.179825577	0.397	0.158	2.46E-08	NO SIGNATURE LEUK	RGS10
HDGFL2	6.85E-13	0.150792432	0.162	0.015	2.51E-08	NO SIGNATURE LEUK	HDGFL2
ADH5	7.04E-13	0.133601853	0.308	0.1	2.58E-08	NO SIGNATURE LEUK	ADH5
SUMF2	7.35E-13	0.158461019	0.223	0.05	2.69E-08	NO SIGNATURE LEUK	SUMF2
TPX2	7.45E-13	0.281634433	0.175	0.023	2.73E-08	NO SIGNATURE LEUK	TPX2
CENPM	7.52E-13	0.171388163	0.133	0	2.75E-08	NO SIGNATURE LEUK	CENPM
OLA1	7.53E-13	0.132059199	0.389	0.147	2.76E-08	NO SIGNATURE LEUK	OLA1
CD320	7.73E-13	0.12713537	0.218	0.047	2.83E-08	NO SIGNATURE LEUK	CD320
TRBC2	7.74E-13	0.217963354	0.586	0.279	2.83E-08	NO SIGNATURE LEUK	TRBC2
SMS	8.15E-13	0.153514009	0.272	0.079	2.98E-08	NO SIGNATURE LEUK	SMS
RNASEH2A	9.56E-13	0.144311032	0.132	0	3.50E-08	NO SIGNATURE LEUK	RNASEH2A
STUB1	9.61E-13	0.123350358	0.29	0.088	3.52E-08	NO SIGNATURE LEUK	STUB1
COMMD1	9.65E-13	0.154460658	0.298	0.097	3.53E-08	NO SIGNATURE LEUK	COMMD1
DECR1	9.95E-13	0.136531875	0.193	0.032	3.64E-08	NO SIGNATURE LEUK	DECR1
BIRC5	1.05E-12	0.252189849	0.153	0.012	3.85E-08	NO SIGNATURE LEUK	BIRC5
GPSM3	1.08E-12	0.168548155	0.503	0.226	3.97E-08	NO SIGNATURE LEUK	GPSM3
COX14	1.13E-12	0.155593816	0.226	0.053	4.13E-08	NO SIGNATURE LEUK	COX14
GGCT	1.18E-12	0.145763377	0.216	0.047	4.32E-08	NO SIGNATURE LEUK	GGCT
RPA1	1.20E-12	0.142651	0.148	0.009	4.38E-08	NO SIGNATURE LEUK	RPA1
COX8A	1.23E-12	0.234777005	0.659	0.37	4.49E-08	NO SIGNATURE LEUK	COX8A
RPL18	1.24E-12	0.216555781	0.997	0.959	4.52E-08	NO SIGNATURE LEUK	RPL18
GMPS	1.29E-12	0.158944037	0.21	0.044	4.71E-08	NO SIGNATURE LEUK	GMPS
SEPTIN1	1.32E-12	0.13869432	0.406	0.164	4.84E-08	NO SIGNATURE LEUK	SEPTIN1
PCMT1	1.33E-12	0.14302994	0.232	0.056	4.87E-08	NO SIGNATURE LEUK	PCMT1
TCEA2	1.33E-12	0.168458685	0.199	0.038	4.87E-08	NO SIGNATURE LEUK	TCEA2
TBXAS1	1.42E-12	0.159191036	0.185	0.029	5.19E-08	NO SIGNATURE LEUK	TBXAS1
CYSLTR2	1.44E-12	0.161531889	0.175	0.023	5.28E-08	NO SIGNATURE LEUK	CYSLTR2
SELENOH	1.47E-12	0.165789482	0.52	0.238	5.39E-08	NO SIGNATURE LEUK	SELENOH
PCNA	1.49E-12	0.187311162	0.229	0.056	5.46E-08	NO SIGNATURE LEUK	PCNA
PLAC8	1.53E-12	0.138892615	0.257	0.07	5.59E-08	NO SIGNATURE LEUK	PLAC8
RPL22	1.61E-12	0.268812415	0.961	0.777	5.88E-08	NO SIGNATURE LEUK	RPL22
CZIB	1.75E-12	0.142218575	0.179	0.026	6.39E-08	NO SIGNATURE LEUK	CZIB
AP1G2	1.79E-12	0.151980245	0.163	0.018	6.54E-08	NO SIGNATURE LEUK	AP1G2
HMGB1	1.80E-12	0.450046198	0.927	0.771	6.57E-08	NO SIGNATURE LEUK	HMGB1
NUTF2	1.84E-12	0.122967632	0.389	0.15	6.72E-08	NO SIGNATURE LEUK	NUTF2
KIF22	1.94E-12	0.157516316	0.263	0.076	7.11E-08	NO SIGNATURE LEUK	KIF22
TBCD	1.95E-12	0.161979038	0.287	0.091	7.12E-08	NO SIGNATURE LEUK	TBCD

MRPS15	1.96E-12	0.138692492	0.289	0.091	7.17E-08	NO SIGNATURE LEUK	MRPS15
SUCLG1	2.00E-12	0.131096719	0.235	0.059	7.31E-08	NO SIGNATURE LEUK	SUCLG1
HSD17B11	2.02E-12	0.143202188	0.299	0.097	7.38E-08	NO SIGNATURE LEUK	HSD17B11
HIVEP3	2.03E-12	0.143437474	0.238	0.062	7.43E-08	NO SIGNATURE LEUK	HIVEP3
AIP	2.05E-12	0.152864126	0.307	0.103	7.51E-08	NO SIGNATURE LEUK	AIP
NRM	2.20E-12	0.147845199	0.167	0.021	8.04E-08	NO SIGNATURE LEUK	NRM
RPL27A	2.47E-12	0.285085308	0.907	0.689	9.04E-08	NO SIGNATURE LEUK	RPL27A
RPL6	2.53E-12	0.25445782	0.986	0.906	9.27E-08	NO SIGNATURE LEUK	RPL6
ARHGEF1	2.73E-12	0.103355862	0.308	0.103	9.97E-08	NO SIGNATURE LEUK	ARHGEF1
FPGS	2.74E-12	0.139926376	0.273	0.085	1.00E-07	NO SIGNATURE LEUK	FPGS
TACC3	2.83E-12	0.142383036	0.145	0.009	1.04E-07	NO SIGNATURE LEUK	TACC3
CAMK4	3.12E-12	0.138079279	0.307	0.106	1.14E-07	NO SIGNATURE LEUK	CAMK4
EIF3D	3.25E-12	0.148105365	0.535	0.249	1.19E-07	NO SIGNATURE LEUK	EIF3D
AP005482.1	3.41E-12	0.118833749	0.198	0.038	1.25E-07	NO SIGNATURE LEUK	AP005482.1
SOCS2-AS1	3.72E-12	0.165609949	0.131	0.003	1.36E-07	NO SIGNATURE LEUK	SOCS2-AS1
GSTK1	3.77E-12	0.131813333	0.408	0.17	1.38E-07	NO SIGNATURE LEUK	GSTK1
TCEAL4	3.97E-12	0.132726519	0.267	0.079	1.45E-07	NO SIGNATURE LEUK	TCEAL4
PAICS	3.98E-12	0.127521362	0.226	0.056	1.46E-07	NO SIGNATURE LEUK	PAICS
ELP5	4.00E-12	0.144291499	0.184	0.032	1.47E-07	NO SIGNATURE LEUK	ELP5
HELLS	4.00E-12	0.148615477	0.197	0.038	1.47E-07	NO SIGNATURE LEUK	HELLS
VAT1	4.03E-12	0.188304058	0.34	0.129	1.47E-07	NO SIGNATURE LEUK	VAT1
FARSA	4.06E-12	0.151357003	0.163	0.021	1.49E-07	NO SIGNATURE LEUK	FARSA
BAZ1B	4.08E-12	0.126959416	0.235	0.062	1.49E-07	NO SIGNATURE LEUK	BAZ1B
ZNHIT1	4.24E-12	0.144259877	0.333	0.123	1.55E-07	NO SIGNATURE LEUK	ZNHIT1
CACYBP	4.30E-12	0.131000992	0.323	0.117	1.58E-07	NO SIGNATURE LEUK	CACYBP
FIS1	4.37E-12	0.133495397	0.317	0.111	1.60E-07	NO SIGNATURE LEUK	FIS1
WDR61	4.37E-12	0.129579937	0.185	0.032	1.60E-07	NO SIGNATURE LEUK	WDR61
LINC02458	4.40E-12	0.164823971	0.125	0	1.61E-07	NO SIGNATURE LEUK	LINC02458
TPR	4.51E-12	0.176177493	0.459	0.205	1.65E-07	NO SIGNATURE LEUK	TPR
NMRAL1	4.59E-12	0.158192389	0.162	0.021	1.68E-07	NO SIGNATURE LEUK	NMRAL1
ABHD17A	4.66E-12	0.136489923	0.234	0.062	1.70E-07	NO SIGNATURE LEUK	ABHD17A
VPS28	4.72E-12	0.149059887	0.407	0.167	1.73E-07	NO SIGNATURE LEUK	VPS28
STK25	4.90E-12	0.144695043	0.141	0.009	1.80E-07	NO SIGNATURE LEUK	STK25
HAUS4	5.07E-12	0.136098508	0.124	0	1.85E-07	NO SIGNATURE LEUK	HAUS4
ARL6IP5	5.16E-12	0.133180677	0.315	0.111	1.89E-07	NO SIGNATURE LEUK	ARL6IP5
SNX3	5.18E-12	0.186171549	0.468	0.214	1.90E-07	NO SIGNATURE LEUK	SNX3
TTC3	5.26E-12	0.12147718	0.297	0.103	1.92E-07	NO SIGNATURE LEUK	TTC3
PNN	5.30E-12	0.19749913	0.584	0.299	1.94E-07	NO SIGNATURE LEUK	PNN
TWF2	5.45E-12	0.140861671	0.308	0.109	2.00E-07	NO SIGNATURE LEUK	TWF2
PA2G4	5.52E-12	0.195080572	0.513	0.246	2.02E-07	NO SIGNATURE LEUK	PA2G4
GINS2	5.75E-12	0.156919228	0.152	0.015	2.11E-07	NO SIGNATURE LEUK	GINS2
GOT2	5.83E-12	0.129607067	0.177	0.029	2.13E-07	NO SIGNATURE LEUK	GOT2
PNISR	5.97E-12	0.138097262	0.648	0.326	2.19E-07	NO SIGNATURE LEUK	PNISR
DPM2	6.08E-12	0.136707715	0.163	0.021	2.23E-07	NO SIGNATURE LEUK	DPM2
REX1BD	6.13E-12	0.16694898	0.288	0.097	2.24E-07	NO SIGNATURE LEUK	REX1BD
POLE4	6.48E-12	0.136444852	0.188	0.035	2.37E-07	NO SIGNATURE LEUK	POLE4
HNRNPA2B1	6.82E-12	0.316669453	0.902	0.713	2.50E-07	NO SIGNATURE LEUK	HNRNPA2B1
SLC25A11	7.11E-12	0.136661854	0.14	0.009	2.60E-07	NO SIGNATURE LEUK	SLC25A11
DNAJC9	7.28E-12	0.115637928	0.391	0.158	2.67E-07	NO SIGNATURE LEUK	DNAJC9
KIFC1	7.40E-12	0.152937454	0.123	0	2.71E-07	NO SIGNATURE LEUK	KIFC1
CD3G	7.70E-12	0.145344994	0.412	0.173	2.82E-07	NO SIGNATURE LEUK	CD3G
EEF1A1	7.71E-12	0.160075337	1	1	2.82E-07	NO SIGNATURE LEUK	EEF1A1
H2AFY	7.99E-12	0.1866051	0.713	0.419	2.93E-07	NO SIGNATURE LEUK	H2AFY
COX11	8.08E-12	0.155168382	0.185	0.035	2.96E-07	NO SIGNATURE LEUK	COX11

HADHA	8.29E-12	0.135410069	0.339	0.126	3.04E-07	NO SIGNATURE LEUK	HADHA
ATP6V1F	8.61E-12	0.153974806	0.329	0.123	3.15E-07	NO SIGNATURE LEUK	ATP6V1F
TSPAN7	8.72E-12	0.149293968	0.213	0.05	3.19E-07	NO SIGNATURE LEUK	TSPAN7
FHIT	8.75E-12	0.135842844	0.247	0.07	3.20E-07	NO SIGNATURE LEUK	FHIT
NDUFA8	8.76E-12	0.149251895	0.148	0.015	3.21E-07	NO SIGNATURE LEUK	NDUFA8
COMMD3	8.83E-12	0.151117875	0.177	0.029	3.23E-07	NO SIGNATURE LEUK	COMMD3
GNPDA1	8.94E-12	0.148044353	0.122	0	3.27E-07	NO SIGNATURE LEUK	GNPDA1
DHRS4L2	9.25E-12	0.143326778	0.15	0.015	3.39E-07	NO SIGNATURE LEUK	DHRS4L2
HAUS1	9.39E-12	0.14289018	0.161	0.021	3.44E-07	NO SIGNATURE LEUK	HAUS1
CCT8	9.44E-12	0.120959718	0.359	0.141	3.45E-07	NO SIGNATURE LEUK	CCT8
RFXANK	9.64E-12	0.144150398	0.19	0.038	3.53E-07	NO SIGNATURE LEUK	RFXANK
C11orf49	9.66E-12	0.136415685	0.171	0.026	3.54E-07	NO SIGNATURE LEUK	C11orf49
SIT1	9.95E-12	0.135099217	0.247	0.07	3.64E-07	NO SIGNATURE LEUK	SIT1
BOP1	1.07E-11	0.13955157	0.159	0.021	3.92E-07	NO SIGNATURE LEUK	BOP1
PGP	1.08E-11	0.15696859	0.168	0.026	3.95E-07	NO SIGNATURE LEUK	PGP
TSPAN3	1.12E-11	0.113251732	0.349	0.132	4.10E-07	NO SIGNATURE LEUK	TSPAN3
EXOSC5	1.12E-11	0.144528118	0.154	0.018	4.10E-07	NO SIGNATURE LEUK	EXOSC5
G6PD	1.16E-11	0.157355295	0.153	0.018	4.26E-07	NO SIGNATURE LEUK	G6PD
MICOS10	1.17E-11	0.120538902	0.313	0.111	4.27E-07	NO SIGNATURE LEUK	MICOS10
CAPN1	1.21E-11	0.125041224	0.197	0.041	4.44E-07	NO SIGNATURE LEUK	CAPN1
PRPSAP1	1.22E-11	0.136405665	0.126	0.003	4.47E-07	NO SIGNATURE LEUK	PRPSAP1
JTB	1.27E-11	0.139121923	0.418	0.182	4.63E-07	NO SIGNATURE LEUK	JTB
PSMF1	1.36E-11	0.128097911	0.379	0.152	4.96E-07	NO SIGNATURE LEUK	PSMF1
LARS	1.36E-11	0.151292651	0.189	0.038	4.98E-07	NO SIGNATURE LEUK	LARS
CHMP4A	1.39E-11	0.134565128	0.154	0.018	5.09E-07	NO SIGNATURE LEUK	CHMP4A
ETHE1	1.41E-11	0.149923665	0.217	0.056	5.16E-07	NO SIGNATURE LEUK	ETHE1
VKORC1	1.41E-11	0.135244685	0.2	0.044	5.17E-07	NO SIGNATURE LEUK	VKORC1
THOC3	1.45E-11	0.122916292	0.175	0.029	5.29E-07	NO SIGNATURE LEUK	THOC3
RUVBL1	1.46E-11	0.136173123	0.131	0.006	5.35E-07	NO SIGNATURE LEUK	RUVBL1
ANAPC11	1.48E-11	0.168872254	0.444	0.202	5.41E-07	NO SIGNATURE LEUK	ANAPC11
AP1M1	1.51E-11	0.115007762	0.227	0.059	5.52E-07	NO SIGNATURE LEUK	AP1M1
GUSB	1.52E-11	0.156495361	0.167	0.026	5.55E-07	NO SIGNATURE LEUK	GUSB
CPNE3	1.58E-11	0.142754774	0.198	0.044	5.79E-07	NO SIGNATURE LEUK	CPNE3
IMMT	1.64E-11	0.123711104	0.235	0.065	6.01E-07	NO SIGNATURE LEUK	IMMT
LAMTOR1	1.70E-11	0.115575933	0.229	0.062	6.21E-07	NO SIGNATURE LEUK	LAMTOR1
EZH2	1.70E-11	0.146545786	0.184	0.035	6.21E-07	NO SIGNATURE LEUK	EZH2
RRM2	1.72E-11	0.191870878	0.13	0.006	6.31E-07	NO SIGNATURE LEUK	RRM2
HAX1	1.74E-11	0.130117603	0.163	0.023	6.36E-07	NO SIGNATURE LEUK	HAX1
TSTD1	1.74E-11	0.145166223	0.369	0.147	6.38E-07	NO SIGNATURE LEUK	TSTD1
DAP3	1.77E-11	0.146288372	0.23	0.065	6.46E-07	NO SIGNATURE LEUK	DAP3
GLO1	1.79E-11	0.132721436	0.228	0.062	6.55E-07	NO SIGNATURE LEUK	GLO1
ILK	1.80E-11	0.114853017	0.179	0.032	6.61E-07	NO SIGNATURE LEUK	ILK
SNHG3	1.90E-11	0.135179435	0.318	0.117	6.96E-07	NO SIGNATURE LEUK	SNHG3
NUDCD2	1.92E-11	0.125516328	0.188	0.038	7.03E-07	NO SIGNATURE LEUK	NUDCD2
TIMM13	1.96E-11	0.143318305	0.366	0.15	7.16E-07	NO SIGNATURE LEUK	TIMM13
POP5	2.17E-11	0.12729269	0.146	0.015	7.94E-07	NO SIGNATURE LEUK	POP5
TRBC1	2.22E-11	0.130237422	0.234	0.065	8.14E-07	NO SIGNATURE LEUK	TRBC1
NENF	2.23E-11	0.140555968	0.293	0.103	8.15E-07	NO SIGNATURE LEUK	NENF
CHURC1	2.26E-11	0.103052136	0.299	0.106	8.26E-07	NO SIGNATURE LEUK	CHURC1
PKMYT1	2.29E-11	0.148344345	0.117	0	8.37E-07	NO SIGNATURE LEUK	PKMYT1
CPSF6	2.34E-11	0.109672114	0.234	0.065	8.58E-07	NO SIGNATURE LEUK	CPSF6
SIVA1	2.39E-11	0.15289824	0.568	0.284	8.75E-07	NO SIGNATURE LEUK	SIVA1
PYURF	2.50E-11	0.107518847	0.388	0.158	9.14E-07	NO SIGNATURE LEUK	PYURF
THOC6	2.53E-11	0.122957279	0.151	0.018	9.26E-07	NO SIGNATURE LEUK	THOC6

SYK	2.61E-11	0.135983701	0.129	0.006	9.55E-07	NO SIGNATURE LEUK	SYK
DNPH1	2.82E-11	0.148854547	0.147	0.018	1.03E-06	NO SIGNATURE LEUK	DNPH1
VDAC3	2.86E-11	0.131521562	0.264	0.085	1.05E-06	NO SIGNATURE LEUK	VDAC3
MPLKIP	3.04E-11	0.129702233	0.21	0.053	1.11E-06	NO SIGNATURE LEUK	MPLKIP
PTTG1	3.14E-11	0.279226222	0.172	0.032	1.15E-06	NO SIGNATURE LEUK	PTTG1
FBXW5	3.14E-11	0.134212472	0.185	0.038	1.15E-06	NO SIGNATURE LEUK	FBXW5
COPZ1	3.22E-11	0.124598429	0.218	0.059	1.18E-06	NO SIGNATURE LEUK	COPZ1
PSMB9	3.22E-11	0.124253153	0.18	0.035	1.18E-06	NO SIGNATURE LEUK	PSMB9
TXN2	3.25E-11	0.121381764	0.246	0.073	1.19E-06	NO SIGNATURE LEUK	TXN2
SAC3D1	3.25E-11	0.133645838	0.143	0.015	1.19E-06	NO SIGNATURE LEUK	SAC3D1
ARGLU1	3.38E-11	0.144931954	0.626	0.32	1.24E-06	NO SIGNATURE LEUK	ARGLU1
HSPB11	3.42E-11	0.142114371	0.194	0.044	1.25E-06	NO SIGNATURE LEUK	HSPB11
ACAT2	3.43E-11	0.123604784	0.144	0.015	1.26E-06	NO SIGNATURE LEUK	ACAT2
FUZ	3.44E-11	0.131882671	0.138	0.012	1.26E-06	NO SIGNATURE LEUK	FUZ
NDUFS5	3.44E-11	0.115623414	0.616	0.311	1.26E-06	NO SIGNATURE LEUK	NDUFS5
MRPL18	3.50E-11	0.11140364	0.172	0.029	1.28E-06	NO SIGNATURE LEUK	MRPL18
RPS9	3.58E-11	0.204682109	0.995	0.974	1.31E-06	NO SIGNATURE LEUK	RPS9
ARPC5	3.59E-11	0.132243689	0.436	0.196	1.31E-06	NO SIGNATURE LEUK	ARPC5
MYO7B	3.70E-11	0.147179143	0.159	0.023	1.35E-06	NO SIGNATURE LEUK	MYO7B
NIPSNAP1	3.85E-11	0.143405684	0.158	0.023	1.41E-06	NO SIGNATURE LEUK	NIPSNAP1
PSMD13	3.87E-11	0.108205675	0.256	0.079	1.42E-06	NO SIGNATURE LEUK	PSMD13
ATP5MD	4.01E-11	0.127218311	0.479	0.22	1.47E-06	NO SIGNATURE LEUK	ATP5MD
IDH3G	4.03E-11	0.132601165	0.215	0.056	1.48E-06	NO SIGNATURE LEUK	IDH3G
CCT6A	4.03E-11	0.162370241	0.441	0.202	1.48E-06	NO SIGNATURE LEUK	CCT6A
HSD17B10	4.15E-11	0.117175949	0.204	0.05	1.52E-06	NO SIGNATURE LEUK	HSD17B10
DPM3	4.37E-11	0.137594257	0.192	0.044	1.60E-06	NO SIGNATURE LEUK	DPM3
HIRIP3	4.47E-11	0.129030646	0.126	0.006	1.64E-06	NO SIGNATURE LEUK	HIRIP3
TASP1	4.55E-11	0.212149587	0.675	0.372	1.67E-06	NO SIGNATURE LEUK	TASP1
THAP4	4.64E-11	0.145647326	0.162	0.026	1.70E-06	NO SIGNATURE LEUK	THAP4
HNRNPUL2	4.74E-11	0.126750665	0.268	0.088	1.73E-06	NO SIGNATURE LEUK	HNRNPUL2
MRPL37	4.78E-11	0.111264207	0.191	0.041	1.75E-06	NO SIGNATURE LEUK	MRPL37
HYI	4.97E-11	0.125393612	0.137	0.012	1.82E-06	NO SIGNATURE LEUK	HYI
ASF1B	5.12E-11	0.148886659	0.131	0.009	1.87E-06	NO SIGNATURE LEUK	ASF1B
MIS18BP1	5.12E-11	0.103475652	0.248	0.076	1.87E-06	NO SIGNATURE LEUK	MIS18BP1
GTF2I	5.13E-11	0.113673114	0.286	0.1	1.88E-06	NO SIGNATURE LEUK	GTF2I
NDUFAF3	5.19E-11	0.13053319	0.13	0.009	1.90E-06	NO SIGNATURE LEUK	NDUFAF3
STMN3	5.20E-11	0.119662123	0.352	0.141	1.90E-06	NO SIGNATURE LEUK	STMN3
CCDC124	5.26E-11	0.134996239	0.258	0.082	1.92E-06	NO SIGNATURE LEUK	CCDC124
RPAIN	5.34E-11	0.136705787	0.201	0.05	1.95E-06	NO SIGNATURE LEUK	RPAIN
BCAP31	5.34E-11	0.103651494	0.379	0.161	1.95E-06	NO SIGNATURE LEUK	BCAP31
PDIA6	5.37E-11	0.112678811	0.305	0.111	1.97E-06	NO SIGNATURE LEUK	PDIA6
CNTRL	5.45E-11	0.114973895	0.215	0.056	2.00E-06	NO SIGNATURE LEUK	CNTRL
CDC20	5.51E-11	0.272627453	0.124	0.006	2.02E-06	NO SIGNATURE LEUK	CDC20
WDR18	5.53E-11	0.110300293	0.174	0.032	2.03E-06	NO SIGNATURE LEUK	WDR18
NDUFS6	5.85E-11	0.106714643	0.362	0.147	2.14E-06	NO SIGNATURE LEUK	NDUFS6
ANXA11	5.86E-11	0.129636185	0.432	0.196	2.15E-06	NO SIGNATURE LEUK	ANXA11
HIST1H1B	5.89E-11	0.283630929	0.165	0.029	2.15E-06	NO SIGNATURE LEUK	HIST1H1B
CENPK	5.98E-11	0.11850568	0.163	0.026	2.19E-06	NO SIGNATURE LEUK	CENPK
DDB1	6.18E-11	0.126140041	0.173	0.032	2.26E-06	NO SIGNATURE LEUK	DDB1
XRCC1	6.22E-11	0.123637898	0.146	0.018	2.28E-06	NO SIGNATURE LEUK	XRCC1
RANGRF	6.49E-11	0.124690127	0.152	0.021	2.37E-06	NO SIGNATURE LEUK	RANGRF
ATP2A3	6.51E-11	0.127390813	0.229	0.065	2.38E-06	NO SIGNATURE LEUK	ATP2A3
TMIGD2	6.58E-11	0.129018623	0.237	0.07	2.41E-06	NO SIGNATURE LEUK	TMIGD2
ATPAF1	6.82E-11	0.129824613	0.14	0.015	2.50E-06	NO SIGNATURE LEUK	ATPAF1

SPSB3	6.96E-11	0.118709527	0.31	0.114	2.55E-06	NO SIGNATURE LEUK	SPSB3
TMEM14B	7.21E-11	0.112203441	0.426	0.191	2.64E-06	NO SIGNATURE LEUK	TMEM14B
EXOSC8	7.56E-11	0.127658661	0.15	0.021	2.77E-06	NO SIGNATURE LEUK	EXOSC8
MSRB2	7.65E-11	0.117301884	0.145	0.018	2.80E-06	NO SIGNATURE LEUK	MSRB2
ATXN10	7.70E-11	0.132489724	0.189	0.044	2.82E-06	NO SIGNATURE LEUK	ATXN10
GTF2A2	7.87E-11	0.112664852	0.257	0.082	2.88E-06	NO SIGNATURE LEUK	GTF2A2
DGUOK	7.93E-11	0.12633701	0.255	0.082	2.90E-06	NO SIGNATURE LEUK	DGUOK
AURKB	8.04E-11	0.144240945	0.111	0	2.94E-06	NO SIGNATURE LEUK	AURKB
ATRAID	8.18E-11	0.127438139	0.249	0.079	2.99E-06	NO SIGNATURE LEUK	ATRAID
DENND2D	8.19E-11	0.130296884	0.221	0.062	3.00E-06	NO SIGNATURE LEUK	DENND2D
CHCHD1	8.21E-11	0.107702021	0.15	0.021	3.01E-06	NO SIGNATURE LEUK	CHCHD1
CXCR4	8.60E-11	0.190066074	0.734	0.446	3.15E-06	NO SIGNATURE LEUK	CXCR4
TOMM6	8.65E-11	0.129943096	0.463	0.211	3.17E-06	NO SIGNATURE LEUK	TOMM6
N4BP2L2	9.02E-11	0.102105192	0.281	0.1	3.30E-06	NO SIGNATURE LEUK	N4BP2L2
SNRNP25	9.11E-11	0.11458646	0.14	0.015	3.33E-06	NO SIGNATURE LEUK	SNRNP25
MAPKAPK5-AS1	9.31E-11	0.123719482	0.205	0.053	3.41E-06	NO SIGNATURE LEUK	MAPKAPK5-AS1
CCNB2	9.69E-11	0.189719896	0.116	0.003	3.55E-06	NO SIGNATURE LEUK	CCNB2
SNW1	9.70E-11	0.100351348	0.231	0.067	3.55E-06	NO SIGNATURE LEUK	SNW1
INTS11	9.80E-11	0.117352559	0.229	0.067	3.59E-06	NO SIGNATURE LEUK	INTS11
HDLBP	1.01E-10	0.112159816	0.144	0.018	3.68E-06	NO SIGNATURE LEUK	HDLBP
ERI3	1.06E-10	0.112534399	0.251	0.079	3.88E-06	NO SIGNATURE LEUK	ERI3
SSNA1	1.21E-10	0.110570913	0.336	0.135	4.42E-06	NO SIGNATURE LEUK	SSNA1
STX10	1.22E-10	0.135022768	0.188	0.044	4.46E-06	NO SIGNATURE LEUK	STX10
MRPL51	1.24E-10	0.115627361	0.126	0.009	4.53E-06	NO SIGNATURE LEUK	MRPL51
EIF2AK1	1.28E-10	0.13282479	0.231	0.07	4.69E-06	NO SIGNATURE LEUK	EIF2AK1
RPL12	1.28E-10	0.255301553	0.995	0.956	4.69E-06	NO SIGNATURE LEUK	RPL12
CDIPT	1.31E-10	0.127678695	0.164	0.029	4.79E-06	NO SIGNATURE LEUK	CDIPT
BAD	1.45E-10	0.110501594	0.204	0.053	5.32E-06	NO SIGNATURE LEUK	BAD
CTNNBL1	1.47E-10	0.1102884	0.138	0.015	5.37E-06	NO SIGNATURE LEUK	CTNNBL1
MT-CO3	1.50E-10	0.182547987	0.995	0.997	5.47E-06	NO SIGNATURE LEUK	MT-CO3
HSBP1	1.51E-10	0.108566402	0.256	0.085	5.53E-06	NO SIGNATURE LEUK	HSBP1
MAD2L2	1.53E-10	0.12646516	0.419	0.188	5.59E-06	NO SIGNATURE LEUK	MAD2L2
TTC19	1.54E-10	0.111190503	0.233	0.07	5.63E-06	NO SIGNATURE LEUK	TTC19
CHD4	1.59E-10	0.114041321	0.285	0.103	5.81E-06	NO SIGNATURE LEUK	CHD4
NAP1L1	1.61E-10	0.198572409	0.721	0.422	5.90E-06	NO SIGNATURE LEUK	NAP1L1
DPP7	1.61E-10	0.119143424	0.232	0.07	5.90E-06	NO SIGNATURE LEUK	DPP7
MCM6	1.66E-10	0.143108924	0.226	0.067	6.09E-06	NO SIGNATURE LEUK	MCM6
CD53	1.77E-10	0.10445768	0.268	0.091	6.49E-06	NO SIGNATURE LEUK	CD53
PPP1R7	1.81E-10	0.112748397	0.207	0.056	6.63E-06	NO SIGNATURE LEUK	PPP1R7
PKN1	1.85E-10	0.113428943	0.28	0.1	6.77E-06	NO SIGNATURE LEUK	PKN1
MRPS16	1.88E-10	0.128262189	0.171	0.035	6.89E-06	NO SIGNATURE LEUK	MRPS16
CDCA8	1.91E-10	0.147272697	0.113	0.003	6.98E-06	NO SIGNATURE LEUK	CDCA8
SCN2A	1.93E-10	0.149339046	0.135	0.015	7.07E-06	NO SIGNATURE LEUK	SCN2A
NDUFS2	1.98E-10	0.117637201	0.212	0.059	7.26E-06	NO SIGNATURE LEUK	NDUFS2
STN1	2.07E-10	0.121582053	0.136	0.015	7.59E-06	NO SIGNATURE LEUK	STN1
PIN4	2.13E-10	0.123797584	0.141	0.018	7.80E-06	NO SIGNATURE LEUK	PIN4
SERBP1	2.17E-10	0.167498777	0.584	0.308	7.93E-06	NO SIGNATURE LEUK	SERBP1
BRD8	2.17E-10	0.130353407	0.119	0.006	7.94E-06	NO SIGNATURE LEUK	BRD8
TUBB4B	2.20E-10	0.433778307	0.372	0.182	8.05E-06	NO SIGNATURE LEUK	TUBB4B
RUFY3	2.25E-10	0.139715096	0.181	0.041	8.23E-06	NO SIGNATURE LEUK	RUFY3
SKP1	2.32E-10	0.134444753	0.6	0.311	8.49E-06	NO SIGNATURE LEUK	SKP1
RHOC	2.32E-10	0.139381157	0.242	0.079	8.51E-06	NO SIGNATURE LEUK	RHOC
TMEM42	2.33E-10	0.130589191	0.107	0	8.52E-06	NO SIGNATURE LEUK	TMEM42
RNF187	2.34E-10	0.123230013	0.234	0.073	8.56E-06	NO SIGNATURE LEUK	RNF187

PLXND1	2.41E-10	0.139899143	0.16	0.029	8.81E-06	NO SIGNATURE LEUK	PLXND1
DGCR6L	2.48E-10	0.108905717	0.175	0.038	9.06E-06	NO SIGNATURE LEUK	DGCR6L
METTL17	2.50E-10	0.13173577	0.17	0.035	9.15E-06	NO SIGNATURE LEUK	METTL17
AC123912.4	2.52E-10	0.13892546	0.112	0.003	9.21E-06	NO SIGNATURE LEUK	AC123912.4
FKBP4	2.55E-10	0.119491923	0.106	0	9.34E-06	NO SIGNATURE LEUK	FKBP4
ZNRD1	2.57E-10	0.116066593	0.349	0.144	9.41E-06	NO SIGNATURE LEUK	ZNRD1
LSM5	2.74E-10	0.101570827	0.355	0.147	1.00E-05	NO SIGNATURE LEUK	LSM5
DHPS	2.74E-10	0.101303509	0.171	0.035	1.00E-05	NO SIGNATURE LEUK	DHPS
BOLA3	2.75E-10	0.119099225	0.138	0.018	1.01E-05	NO SIGNATURE LEUK	BOLA3
MSH6	2.77E-10	0.131004826	0.16	0.029	1.01E-05	NO SIGNATURE LEUK	MSH6
CALM3	2.79E-10	0.127932409	0.411	0.188	1.02E-05	NO SIGNATURE LEUK	CALM3
ASPM	2.88E-10	0.192066375	0.111	0.003	1.05E-05	NO SIGNATURE LEUK	ASPM
IVD	3.06E-10	0.125759216	0.134	0.015	1.12E-05	NO SIGNATURE LEUK	IVD
BCL7C	3.12E-10	0.127791585	0.217	0.065	1.14E-05	NO SIGNATURE LEUK	BCL7C
APIP	3.22E-10	0.12745497	0.133	0.015	1.18E-05	NO SIGNATURE LEUK	APIP
THOP1	3.27E-10	0.116852178	0.117	0.006	1.20E-05	NO SIGNATURE LEUK	THOP1
HMGA1	3.29E-10	0.154507531	0.595	0.32	1.20E-05	NO SIGNATURE LEUK	HMGA1
YEATS4	3.50E-10	0.121918114	0.167	0.035	1.28E-05	NO SIGNATURE LEUK	YEATS4
EIF3I	3.64E-10	0.12941448	0.482	0.235	1.33E-05	NO SIGNATURE LEUK	EIF3I
CDKN1B	3.79E-10	0.134456211	0.126	0.012	1.39E-05	NO SIGNATURE LEUK	CDKN1B
PAGR1	3.80E-10	0.126875887	0.132	0.015	1.39E-05	NO SIGNATURE LEUK	PAGR1
HNRNPM	3.87E-10	0.110025575	0.487	0.238	1.42E-05	NO SIGNATURE LEUK	HNRNPM
CCDC26	3.93E-10	0.132717371	0.174	0.038	1.44E-05	NO SIGNATURE LEUK	CCDC26
HDDC2	3.96E-10	0.110833895	0.165	0.032	1.45E-05	NO SIGNATURE LEUK	HDDC2
SPI1	4.04E-10	0.120960414	0.153	0.026	1.48E-05	NO SIGNATURE LEUK	SPI1
AAAS	4.14E-10	0.103762362	0.143	0.021	1.51E-05	NO SIGNATURE LEUK	AAAS
PDLIM2	4.14E-10	0.126430989	0.11	0.003	1.52E-05	NO SIGNATURE LEUK	PDLIM2
MRPL12	4.22E-10	0.12615913	0.151	0.026	1.55E-05	NO SIGNATURE LEUK	MRPL12
AK3	4.29E-10	0.132104412	0.168	0.035	1.57E-05	NO SIGNATURE LEUK	AK3
BDH1	4.41E-10	0.123187745	0.109	0.003	1.62E-05	NO SIGNATURE LEUK	BDH1
HNRNPAB	4.49E-10	0.105950596	0.288	0.109	1.64E-05	NO SIGNATURE LEUK	HNRNPAB
UGP2	4.50E-10	0.130247388	0.358	0.155	1.65E-05	NO SIGNATURE LEUK	UGP2
LSM2	4.60E-10	0.107056578	0.368	0.158	1.69E-05	NO SIGNATURE LEUK	LSM2
CCT5	4.62E-10	0.109967402	0.362	0.155	1.69E-05	NO SIGNATURE LEUK	CCT5
CD4	4.70E-10	0.12138404	0.142	0.021	1.72E-05	NO SIGNATURE LEUK	CD4
TCTEX1D2	4.73E-10	0.119495597	0.203	0.056	1.73E-05	NO SIGNATURE LEUK	TCTEX1D2
TST	4.82E-10	0.109358999	0.12	0.009	1.76E-05	NO SIGNATURE LEUK	TST
FLOT2	4.82E-10	0.120107753	0.115	0.006	1.76E-05	NO SIGNATURE LEUK	FLOT2
NHP2	4.94E-10	0.101980674	0.293	0.111	1.81E-05	NO SIGNATURE LEUK	NHP2
USP1	4.95E-10	0.111455549	0.331	0.138	1.81E-05	NO SIGNATURE LEUK	USP1
MRPL24	5.00E-10	0.116034154	0.115	0.006	1.83E-05	NO SIGNATURE LEUK	MRPL24
ACAA1	5.04E-10	0.11113245	0.245	0.082	1.84E-05	NO SIGNATURE LEUK	ACAA1
CLSPN	5.23E-10	0.116215421	0.114	0.006	1.91E-05	NO SIGNATURE LEUK	CLSPN
HPF1	5.24E-10	0.114801582	0.115	0.006	1.92E-05	NO SIGNATURE LEUK	HPF1
NDUFS7	5.27E-10	0.104729209	0.314	0.126	1.93E-05	NO SIGNATURE LEUK	NDUFS7
JOSD2	5.37E-10	0.118046626	0.12	0.009	1.96E-05	NO SIGNATURE LEUK	JOSD2
APRT	5.53E-10	0.185384981	0.584	0.314	2.03E-05	NO SIGNATURE LEUK	APRT
COASY	5.76E-10	0.10624175	0.142	0.021	2.11E-05	NO SIGNATURE LEUK	COASY
REPIN1	5.79E-10	0.108302218	0.426	0.196	2.12E-05	NO SIGNATURE LEUK	REPIN1
PDE6D	5.82E-10	0.116716049	0.114	0.006	2.13E-05	NO SIGNATURE LEUK	PDE6D
SCRN2	5.87E-10	0.124494757	0.125	0.012	2.15E-05	NO SIGNATURE LEUK	SCRN2
RNASEH2C	5.89E-10	0.127574761	0.135	0.018	2.16E-05	NO SIGNATURE LEUK	RNASEH2C
SPRY2	5.93E-10	0.153590373	0.141	0.021	2.17E-05	NO SIGNATURE LEUK	SPRY2
NSD2	6.09E-10	0.108592618	0.178	0.041	2.23E-05	NO SIGNATURE LEUK	NSD2

PRDX5	6.10E-10	0.151817048	0.574	0.293	2.23E-05	NO SIGNATURE LEUK	PRDX5
NDUFB5	6.10E-10	0.105674936	0.141	0.021	2.23E-05	NO SIGNATURE LEUK	NDUFB5
ATP6AP1	6.13E-10	0.112794179	0.151	0.026	2.24E-05	NO SIGNATURE LEUK	ATP6AP1
RPUSD3	6.37E-10	0.115658652	0.13	0.015	2.33E-05	NO SIGNATURE LEUK	RPUSD3
MRPS12	6.43E-10	0.102990283	0.188	0.047	2.35E-05	NO SIGNATURE LEUK	MRPS12
TOP2A	6.49E-10	0.209488074	0.145	0.023	2.37E-05	NO SIGNATURE LEUK	TOP2A
CKAP5	6.64E-10	0.120878467	0.135	0.018	2.43E-05	NO SIGNATURE LEUK	CKAP5
METTL5	6.69E-10	0.118391959	0.167	0.035	2.45E-05	NO SIGNATURE LEUK	METTL5
MAD1L1	6.77E-10	0.113474984	0.19	0.05	2.48E-05	NO SIGNATURE LEUK	MAD1L1
IAH1	6.94E-10	0.122295865	0.191	0.05	2.54E-05	NO SIGNATURE LEUK	IAH1
NUDT5	7.00E-10	0.106662337	0.156	0.029	2.56E-05	NO SIGNATURE LEUK	NUDT5
MFNG	7.28E-10	0.115049145	0.161	0.032	2.66E-05	NO SIGNATURE LEUK	MFNG
HPCAL1	7.35E-10	0.115303928	0.166	0.035	2.69E-05	NO SIGNATURE LEUK	HPCAL1
APOBEC3C	7.40E-10	0.11416707	0.124	0.012	2.71E-05	NO SIGNATURE LEUK	APOBEC3C
EXOC4	7.51E-10	0.109797846	0.15	0.026	2.75E-05	NO SIGNATURE LEUK	EXOC4
ITPA	7.80E-10	0.10362389	0.196	0.053	2.85E-05	NO SIGNATURE LEUK	ITPA
ATP5PB	8.48E-10	0.112927746	0.475	0.232	3.10E-05	NO SIGNATURE LEUK	ATP5PB
ACOT7	8.59E-10	0.100420993	0.217	0.067	3.15E-05	NO SIGNATURE LEUK	ACOT7
GPS2	8.80E-10	0.100706841	0.258	0.091	3.22E-05	NO SIGNATURE LEUK	GPS2
SMYD2	8.84E-10	0.116399258	0.129	0.015	3.23E-05	NO SIGNATURE LEUK	SMYD2
COPB2	9.00E-10	0.113327524	0.133	0.018	3.29E-05	NO SIGNATURE LEUK	COPB2
IRF2	9.45E-10	0.116572514	0.106	0.003	3.46E-05	NO SIGNATURE LEUK	IRF2
NEIL3	9.70E-10	0.106821731	0.117	0.009	3.55E-05	NO SIGNATURE LEUK	NEIL3
CCDC91	1.02E-09	0.127431652	0.154	0.029	3.73E-05	NO SIGNATURE LEUK	CCDC91
MTHFD1	1.06E-09	0.107797832	0.122	0.012	3.88E-05	NO SIGNATURE LEUK	MTHFD1
RPL5	1.10E-09	0.209863918	0.992	0.938	4.02E-05	NO SIGNATURE LEUK	RPL5
HIST1H3D	1.11E-09	0.159951948	0.193	0.056	4.06E-05	NO SIGNATURE LEUK	HIST1H3D
VBP1	1.12E-09	0.107618519	0.169	0.038	4.09E-05	NO SIGNATURE LEUK	VBP1
NCBP3	1.12E-09	0.113565595	0.143	0.023	4.10E-05	NO SIGNATURE LEUK	NCBP3
PCBD1	1.13E-09	0.114270779	0.116	0.009	4.15E-05	NO SIGNATURE LEUK	PCBD1
GALK1	1.16E-09	0.105561931	0.11	0.006	4.26E-05	NO SIGNATURE LEUK	GALK1
UQCRH	1.20E-09	0.160741597	0.623	0.334	4.37E-05	NO SIGNATURE LEUK	UQCRH
ALOX5	1.21E-09	0.118794133	0.122	0.012	4.42E-05	NO SIGNATURE LEUK	ALOX5
EEF2	1.21E-09	0.265350138	0.96	0.798	4.44E-05	NO SIGNATURE LEUK	EEF2
ATP5F1B	1.22E-09	0.191497075	0.758	0.46	4.45E-05	NO SIGNATURE LEUK	ATP5F1B
VRK3	1.22E-09	0.105188132	0.164	0.035	4.45E-05	NO SIGNATURE LEUK	VRK3
HMG20B	1.24E-09	0.114935007	0.188	0.05	4.53E-05	NO SIGNATURE LEUK	HMG20B
ICAM2	1.25E-09	0.104681057	0.198	0.056	4.57E-05	NO SIGNATURE LEUK	ICAM2
CCS	1.28E-09	0.119473162	0.138	0.021	4.69E-05	NO SIGNATURE LEUK	CCS
USP48	1.31E-09	0.117356511	0.163	0.035	4.81E-05	NO SIGNATURE LEUK	USP48
HMGN4	1.33E-09	0.108304578	0.218	0.067	4.85E-05	NO SIGNATURE LEUK	HMGN4
PPDPF	1.39E-09	0.165080126	0.631	0.364	5.10E-05	NO SIGNATURE LEUK	PPDPF
BSPRY	1.42E-09	0.111650387	0.11	0.006	5.18E-05	NO SIGNATURE LEUK	BSPRY
LANCL1	1.43E-09	0.124258814	0.115	0.009	5.23E-05	NO SIGNATURE LEUK	LANCL1
RCC1	1.47E-09	0.102907759	0.11	0.006	5.38E-05	NO SIGNATURE LEUK	RCC1
CALHM6	1.50E-09	0.119898404	0.109	0.006	5.47E-05	NO SIGNATURE LEUK	CALHM6
TRAPPC6A	1.52E-09	0.117341676	0.214	0.067	5.58E-05	NO SIGNATURE LEUK	TRAPPC6A
LSM10	1.60E-09	0.117384573	0.114	0.009	5.86E-05	NO SIGNATURE LEUK	LSM10
RANBP1	1.65E-09	0.121875246	0.433	0.211	6.03E-05	NO SIGNATURE LEUK	RANBP1
DALRD3	1.68E-09	0.127687506	0.12	0.012	6.14E-05	NO SIGNATURE LEUK	DALRD3
SSBP1	1.70E-09	0.101063064	0.413	0.191	6.24E-05	NO SIGNATURE LEUK	SSBP1
ERH	1.74E-09	0.115701859	0.563	0.29	6.36E-05	NO SIGNATURE LEUK	ERH
FLYWCH2	1.75E-09	0.11793477	0.135	0.021	6.39E-05	NO SIGNATURE LEUK	FLYWCH2
COQ4	1.82E-09	0.12613284	0.149	0.029	6.67E-05	NO SIGNATURE LEUK	COQ4

CTDSP1	1.90E-09	0.108158261	0.146	0.026	6.95E-05	NO SIGNATURE LEUK	CTDSP1
C12orf10	1.90E-09	0.101179583	0.211	0.065	6.96E-05	NO SIGNATURE LEUK	C12orf10
CYC1	1.92E-09	0.10318528	0.392	0.185	7.02E-05	NO SIGNATURE LEUK	CYC1
PDCD6IP	1.92E-09	0.117150878	0.19	0.053	7.04E-05	NO SIGNATURE LEUK	PDCD6IP
NAPRT	1.93E-09	0.106801629	0.156	0.032	7.07E-05	NO SIGNATURE LEUK	NAPRT
UBE2T	1.98E-09	0.100521468	0.113	0.009	7.24E-05	NO SIGNATURE LEUK	UBE2T
MT-CO1	2.00E-09	0.175197465	0.997	0.997	7.31E-05	NO SIGNATURE LEUK	MT-CO1
NFU1	2.14E-09	0.100578959	0.145	0.026	7.84E-05	NO SIGNATURE LEUK	NFU1
CCNB1IP1	2.17E-09	0.106621079	0.226	0.076	7.96E-05	NO SIGNATURE LEUK	CCNB1IP1
RPS11	2.42E-09	0.238368158	0.895	0.633	8.85E-05	NO SIGNATURE LEUK	RPS11
SFXN4	2.54E-09	0.10580509	0.151	0.029	9.31E-05	NO SIGNATURE LEUK	SFXN4
PMVK	2.63E-09	0.108985531	0.101	0.003	9.61E-05	NO SIGNATURE LEUK	PMVK
POLD1	2.66E-09	0.103733156	0.107	0.006	9.74E-05	NO SIGNATURE LEUK	POLD1
ING4	2.74E-09	0.117355801	0.139	0.023	0.00010032	NO SIGNATURE LEUK	ING4
BLK	2.76E-09	0.113442	0.112	0.009	0.000101134	NO SIGNATURE LEUK	BLK
LIG1	2.84E-09	0.102340735	0.145	0.026	0.000103971	NO SIGNATURE LEUK	LIG1
BORCS7	2.96E-09	0.124527241	0.158	0.035	0.000108276	NO SIGNATURE LEUK	BORCS7
SNX1	3.14E-09	0.104092377	0.134	0.021	0.000115059	NO SIGNATURE LEUK	SNX1
SCG2	3.20E-09	0.126515077	0.106	0.006	0.000116993	NO SIGNATURE LEUK	SCG2
C20orf27	3.25E-09	0.112353494	0.143	0.026	0.00011884	NO SIGNATURE LEUK	C20orf27
MAN1A1	3.26E-09	0.118227081	0.122	0.015	0.000119269	NO SIGNATURE LEUK	MAN1A1
DPEP1	3.38E-09	0.108286872	0.332	0.147	0.000123887	NO SIGNATURE LEUK	DPEP1
DEF6	3.45E-09	0.122307581	0.201	0.062	0.000126177	NO SIGNATURE LEUK	DEF6
PAQR4	3.48E-09	0.10661051	0.105	0.006	0.00012729	NO SIGNATURE LEUK	PAQR4
MCTS1	3.51E-09	0.106817664	0.127	0.018	0.000128317	NO SIGNATURE LEUK	MCTS1
ARHGAP30	3.56E-09	0.102614478	0.184	0.05	0.000130331	NO SIGNATURE LEUK	ARHGAP30
MDFIC	3.86E-09	0.100736792	0.272	0.106	0.000141297	NO SIGNATURE LEUK	MDFIC
PARD3	3.92E-09	0.101762152	0.192	0.056	0.000143298	NO SIGNATURE LEUK	PARD3
TP53I13	4.05E-09	0.107482025	0.146	0.029	0.000148129	NO SIGNATURE LEUK	TP53I13
MUTYH	4.05E-09	0.104789863	0.132	0.021	0.000148344	NO SIGNATURE LEUK	MUTYH
MGST2	4.10E-09	0.111618604	0.127	0.018	0.000149887	NO SIGNATURE LEUK	MGST2
ZNF32	4.13E-09	0.110832164	0.166	0.041	0.000150998	NO SIGNATURE LEUK	ZNF32
LINC01550	4.23E-09	0.124639596	0.11	0.009	0.000154931	NO SIGNATURE LEUK	LINC01550
HPS1	4.49E-09	0.102718743	0.186	0.053	0.000164336	NO SIGNATURE LEUK	HPS1
HNRNPU	4.52E-09	0.104060507	0.572	0.317	0.000165254	NO SIGNATURE LEUK	HNRNPU
MZT2B	4.75E-09	0.175991176	0.715	0.44	0.000173994	NO SIGNATURE LEUK	MZT2B
SEPTIN9	5.07E-09	0.10834176	0.493	0.255	0.000185644	NO SIGNATURE LEUK	SEPTIN9
CHI3L2	5.19E-09	0.128054569	0.14	0.026	0.000189959	NO SIGNATURE LEUK	CHI3L2
COA6	5.40E-09	0.107408794	0.144	0.029	0.000197488	NO SIGNATURE LEUK	COA6
EIF3A	5.65E-09	0.105645475	0.53	0.279	0.000206924	NO SIGNATURE LEUK	EIF3A
CORO1B	5.91E-09	0.107923592	0.164	0.041	0.000216143	NO SIGNATURE LEUK	CORO1B
EIF3L	6.01E-09	0.156833291	0.729	0.411	0.000220091	NO SIGNATURE LEUK	EIF3L
FAM47E	6.10E-09	0.120412368	0.108	0.009	0.000223317	NO SIGNATURE LEUK	FAM47E
ZNF738	6.61E-09	0.105934304	0.114	0.012	0.000241898	NO SIGNATURE LEUK	ZNF738
TRIP11	6.68E-09	0.110283765	0.119	0.015	0.000244566	NO SIGNATURE LEUK	TRIP11
PSAP	6.79E-09	0.128598334	0.632	0.352	0.000248534	NO SIGNATURE LEUK	PSAP
BCAP29	6.86E-09	0.104270132	0.108	0.009	0.000251158	NO SIGNATURE LEUK	BCAP29
ATF3	7.33E-09	0.113649157	0.169	0.044	0.000268106	NO SIGNATURE LEUK	ATF3
SLBP	7.43E-09	0.112687912	0.227	0.082	0.000271773	NO SIGNATURE LEUK	SLBP
GIMAP2	7.46E-09	0.106061841	0.113	0.012	0.000272912	NO SIGNATURE LEUK	GIMAP2
SPATA13	7.71E-09	0.132389048	0.181	0.053	0.000282026	NO SIGNATURE LEUK	SPATA13
CMBL	7.86E-09	0.116505407	0.106	0.009	0.000287844	NO SIGNATURE LEUK	CMBL
HNRNPA3	8.15E-09	0.142442333	0.658	0.381	0.000298326	NO SIGNATURE LEUK	HNRNPA3
ZNF439	8.40E-09	0.102537376	0.139	0.026	0.000307323	NO SIGNATURE LEUK	ZNF439

SLC35A4	8.64E-09	0.102030385	0.154	0.035	0.000316215	NO SIGNATURE LEUK	SLC35A4
OXNAD1	8.90E-09	0.114337049	0.157	0.038	0.000325748	NO SIGNATURE LEUK	OXNAD1
PAM16	8.94E-09	0.107663823	0.111	0.012	0.000327214	NO SIGNATURE LEUK	PAM16
POLD3	9.27E-09	0.106320943	0.106	0.009	0.000339402	NO SIGNATURE LEUK	POLD3
CHAF1A	9.48E-09	0.102390727	0.117	0.015	0.000346796	NO SIGNATURE LEUK	CHAF1A
GCSAM	9.61E-09	0.118436519	0.1	0.006	0.000351822	NO SIGNATURE LEUK	GCSAM
LINC00665	9.91E-09	0.107821617	0.111	0.012	0.000362845	NO SIGNATURE LEUK	LINC00665
ATAD5	1.04E-08	0.101174115	0.116	0.015	0.000381974	NO SIGNATURE LEUK	ATAD5
CEP57	1.12E-08	0.106097652	0.146	0.032	0.000410622	NO SIGNATURE LEUK	CEP57
ERCC5	1.12E-08	0.108281517	0.11	0.012	0.00041076	NO SIGNATURE LEUK	ERCC5
HERC2	1.13E-08	0.102950994	0.147	0.032	0.000412258	NO SIGNATURE LEUK	HERC2
RPS24	1.13E-08	0.187573991	0.999	0.991	0.000414399	NO SIGNATURE LEUK	RPS24
ARL3	1.15E-08	0.106147165	0.105	0.009	0.000420294	NO SIGNATURE LEUK	ARL3
TRADD	1.21E-08	0.113558637	0.141	0.029	0.000441927	NO SIGNATURE LEUK	TRADD
PRMT5	1.21E-08	0.104086497	0.115	0.015	0.000442052	NO SIGNATURE LEUK	PRMT5
TXNIP	1.24E-08	0.22114115	0.747	0.478	0.000453273	NO SIGNATURE LEUK	TXNIP
BLMH	1.26E-08	0.103142628	0.121	0.018	0.000459996	NO SIGNATURE LEUK	BLMH
ABCF2	1.26E-08	0.103010863	0.109	0.012	0.000460368	NO SIGNATURE LEUK	ABCF2
CD2	1.27E-08	0.120768147	0.104	0.009	0.000464223	NO SIGNATURE LEUK	CD2
CDC37	1.39E-08	0.10213784	0.472	0.238	0.000510175	NO SIGNATURE LEUK	CDC37
RPL32	1.45E-08	0.161910744	1	0.985	0.000529107	NO SIGNATURE LEUK	RPL32
ABHD14B	1.45E-08	0.107555399	0.244	0.097	0.000529352	NO SIGNATURE LEUK	ABHD14B
C1orf21	1.48E-08	0.101207851	0.12	0.018	0.000541937	NO SIGNATURE LEUK	C1orf21
TCEAL3	1.55E-08	0.108394743	0.115	0.015	0.000568247	NO SIGNATURE LEUK	TCEAL3
MRPS33	1.58E-08	0.105102347	0.109	0.012	0.000579926	NO SIGNATURE LEUK	MRPS33
TCAF1	1.62E-08	0.100130494	0.125	0.021	0.000593987	NO SIGNATURE LEUK	TCAF1
DPP4	1.64E-08	0.10235864	0.104	0.009	0.000599711	NO SIGNATURE LEUK	DPP4
LETMD1	1.82E-08	0.100534943	0.155	0.038	0.000666676	NO SIGNATURE LEUK	LETMD1
ARMC10	1.90E-08	0.109258144	0.117	0.018	0.000695233	NO SIGNATURE LEUK	ARMC10
RPS13	2.06E-08	0.174115428	0.995	0.977	0.000755628	NO SIGNATURE LEUK	RPS13
PRR5	2.08E-08	0.130778298	0.137	0.029	0.000761853	NO SIGNATURE LEUK	PRR5
SETD7	2.12E-08	0.105573313	0.102	0.009	0.000777074	NO SIGNATURE LEUK	SETD7
VSIR	2.15E-08	0.112995219	0.118	0.018	0.000788192	NO SIGNATURE LEUK	VSIR
WDR86-AS1	2.38E-08	0.101400017	0.107	0.012	0.000871687	NO SIGNATURE LEUK	WDR86-AS1
MCUR1	2.45E-08	0.102336094	0.148	0.035	0.000898461	NO SIGNATURE LEUK	MCUR1
AHCYL1	2.46E-08	0.101892743	0.122	0.021	0.000901083	NO SIGNATURE LEUK	AHCYL1
HNRNPDL	2.51E-08	0.193027152	0.801	0.537	0.000917191	NO SIGNATURE LEUK	HNRNPDL
SNHG8	2.52E-08	0.133767915	0.682	0.396	0.0009213	NO SIGNATURE LEUK	SNHG8
COA1	2.54E-08	0.10868389	0.176	0.053	0.000929799	NO SIGNATURE LEUK	COA1
WDR86	2.69E-08	0.10661032	0.107	0.012	0.000982759	NO SIGNATURE LEUK	WDR86
HIST1H1D	2.90E-08	0.184024169	0.586	0.346	0.001061018	NO SIGNATURE LEUK	HIST1H1D
GTF3A	3.02E-08	0.135424803	0.691	0.396	0.001104826	NO SIGNATURE LEUK	GTF3A
ARL6IP4	3.09E-08	0.107564215	0.534	0.284	0.001130909	NO SIGNATURE LEUK	ARL6IP4
GIHCG	3.71E-08	0.145832984	0.726	0.437	0.001358391	NO SIGNATURE LEUK	GIHCG
KATNAL1	3.84E-08	0.104118981	0.11	0.015	0.001403807	NO SIGNATURE LEUK	KATNAL1
SLC25A6	4.09E-08	0.181752923	0.791	0.493	0.001496197	NO SIGNATURE LEUK	SLC25A6
BSCL2	4.76E-08	0.100149286	0.135	0.029	0.00174209	NO SIGNATURE LEUK	BSCL2
MFAP4	5.05E-08	0.114596802	0.108	0.015	0.001849875	NO SIGNATURE LEUK	MFAP4
SAT2	5.46E-08	0.102145342	0.124	0.023	0.001996753	NO SIGNATURE LEUK	SAT2
MRPS27	6.57E-08	0.109638277	0.116	0.021	0.002405331	NO SIGNATURE LEUK	MRPS27
STK32C	7.00E-08	0.105697361	0.106	0.015	0.002563333	NO SIGNATURE LEUK	STK32C
SRP14	7.23E-08	0.172500681	0.845	0.569	0.002645545	NO SIGNATURE LEUK	SRP14
SHARPIN	8.16E-08	0.100253334	0.183	0.062	0.002986401	NO SIGNATURE LEUK	SHARPIN
NOA1	9.18E-08	0.110581006	0.129	0.029	0.003360936	NO SIGNATURE LEUK	NOA1

HMGN1	1.28E-07	0.134463486	0.693	0.416	0.004689875	NO SIGNATURE LEUK	HMGN1
NCL	1.50E-07	0.178810562	0.796	0.545	0.005507326	NO SIGNATURE LEUK	NCL
NME3	1.65E-07	0.101683665	0.108	0.018	0.00604143	NO SIGNATURE LEUK	NME3
RPLP2	1.95E-07	0.18401083	0.989	0.912	0.007141435	NO SIGNATURE LEUK	RPLP2
UBL5	2.15E-07	0.100337978	0.596	0.34	0.007880504	NO SIGNATURE LEUK	UBL5
PTMA	2.21E-07	0.185579906	0.997	0.979	0.008073633	NO SIGNATURE LEUK	PTMA
RPL27	2.69E-07	0.181802273	0.96	0.786	0.009858175	NO SIGNATURE LEUK	RPL27
RHOA	6.87E-07	0.173792017	0.773	0.507	0.025132267	NO SIGNATURE LEUK	RHOA
UBB	8.50E-07	0.18814839	0.847	0.592	0.03109462	NO SIGNATURE LEUK	UBB
DUSP1	8.68E-07	0.106335741	0.179	0.067	0.031783351	NO SIGNATURE LEUK	DUSP1
CALM2	9.25E-07	0.182401498	0.703	0.452	0.033862159	NO SIGNATURE LEUK	CALM2
MT-CO2	1.42E-06	0.125718742	0.996	1	0.052038545	NO SIGNATURE LEUK	MT-CO2
RPS17	1.63E-06	0.176070355	0.858	0.616	0.059679968	NO SIGNATURE LEUK	RPS17
EIF3F	2.10E-06	0.154297037	0.799	0.537	0.076912929	NO SIGNATURE LEUK	EIF3F
RPL36	2.97E-06	0.157256562	0.98	0.88	0.10863094	NO SIGNATURE LEUK	RPL36
GMFG	3.49E-06	0.105589645	0.738	0.46	0.127827966	NO SIGNATURE LEUK	GMFG
RPS28	3.98E-06	0.129896507	0.997	0.988	0.145613375	NO SIGNATURE LEUK	RPS28
RPL13A	5.27E-06	0.151912841	0.987	0.915	0.192824264	NO SIGNATURE LEUK	RPL13A
TUBA1A	7.80E-06	0.175557658	0.768	0.516	0.285381059	NO SIGNATURE LEUK	TUBA1A
JUN	8.64E-06	0.171217289	0.836	0.707	0.316369838	NO SIGNATURE LEUK	JUN
RPL39	1.01E-05	0.139799923	0.995	0.953	0.368305871	NO SIGNATURE LEUK	RPL39
HIST1H4C	1.67E-05	0.877551596	0.581	0.396	0.61162118	NO SIGNATURE LEUK	HIST1H4C
GAS5	2.00E-05	0.180732931	0.82	0.563	0.730562085	NO SIGNATURE LEUK	GAS5
PFDN5	2.19E-05	0.108184174	0.867	0.575	0.800633761	NO SIGNATURE LEUK	PFDN5
RPS19	2.25E-05	0.153428472	0.999	0.985	0.825046727	NO SIGNATURE LEUK	RPS19
TLE5	4.60E-05	0.115565508	0.807	0.543	1	NO SIGNATURE LEUK	TLE5
RPS16	9.30E-05	0.109254636	0.983	0.915	1	NO SIGNATURE LEUK	RPS16
ARMH1	0.000302144	0.103308903	0.844	0.607	1	NO SIGNATURE LEUK	ARMH1
RPL17	0.000447697	0.112159345	0.992	0.921	1	NO SIGNATURE LEUK	RPL17
RPS29	0.000836677	0.113089089	0.953	0.821	1	NO SIGNATURE LEUK	RPS29
TMSB4X	0.001009964	0.105507149	0.996	0.985	1	NO SIGNATURE LEUK	TMSB4X
MT-ATP6	0.001924011	0.100562278	0.984	0.944	1	NO SIGNATURE LEUK	MT-ATP6
HSP90AA1	0.002467232	0.135887828	0.894	0.78	1	NO SIGNATURE LEUK	HSP90AA1
RPL7	0.003498253	0.101603373	0.976	0.865	1	NO SIGNATURE LEUK	RPL7
