# Supplementary material for: High CD44 expression and enhanced E-selectin binding identified as biomarkers of chemoresistant leukemic cells in human T-ALL
Source: Leukemia. 2024 Nov 24;39(2):323–36. doi: 10.1038/s41375-024-02473-7 (PMC11794132; doi:10.1038/s41375-024-02473-7)
Supplement: Supplementary file 7 — Supplemental Table 6 [file 41375_2024_2473_MOESM7_ESM.pdf]

upregulated genes in Ki67neg CD44high leukemic cells from Library 2 (M143 & M143R) (Supplementary Figure 10e)

	p_val	avg_log2FC	pct.1	pct.2	p_val_adj	cluster	gene
CD44	1.14E-96	1.682954921	1	0.104	4.19E-92	CD44 > 1 & MKI67 < 1 Leuk	CD44
MALAT1	7.89E-10	0.964820825	1	1	2.89E-05	CD44 > 1 & MKI67 < 1 Leuk	MALAT1
SOX4	2.69E-06	0.577857468	1	0.983	0.09839289	CD44 > 1 & MKI67 < 1 Leuk	SOX4
SGPP1	3.87E-05	0.190726288	0.111	0.021	1	CD44 > 1 & MKI67 < 1 Leuk	SGPP1
SF1	5.48E-05	0.503900845	0.8	0.787	1	CD44 > 1 & MKI67 < 1 Leuk	SF1
HLA-C	0.00012528	0.590708626	0.867	0.864	1	CD44 > 1 & MKI67 < 1 Leuk	HLA-C
MIAT	0.000161352	0.488379954	0.222	0.077	1	CD44 > 1 & MKI67 < 1 Leuk	MIAT
HERPUD1	0.000162477	0.442377274	0.867	0.773	1	CD44 > 1 & MKI67 < 1 Leuk	HERPUD1
SNED1	0.00022406	0.200139421	0.111	0.025	1	CD44 > 1 & MKI67 < 1 Leuk	SNED1
JUND	0.000262538	0.446775507	0.911	0.921	1	CD44 > 1 & MKI67 < 1 Leuk	JUND
COQ7	0.00054067	0.495746285	0.156	0.049	1	CD44 > 1 & MKI67 < 1 Leuk	COQ7
PGPEP1	0.000567345	0.363109821	0.156	0.049	1	CD44 > 1 & MKI67 < 1 Leuk	PGPEP1
ARL4C	0.000585151	0.718787284	0.733	0.712	1	CD44 > 1 & MKI67 < 1 Leuk	ARL4C
EMP3	0.001060224	0.757621728	0.489	0.365	1	CD44 > 1 & MKI67 < 1 Leuk	EMP3
FTX	0.001255614	0.541551883	0.222	0.095	1	CD44 > 1 & MKI67 < 1 Leuk	FTX
VAT1	0.001305058	0.466636578	0.6	0.505	1	CD44 > 1 & MKI67 < 1 Leuk	VAT1
EIF1	0.001476109	0.278310803	0.933	0.988	1	CD44 > 1 & MKI67 < 1 Leuk	EIF1
PLK3	0.001586877	0.406039796	0.222	0.093	1	CD44 > 1 & MKI67 < 1 Leuk	PLK3
TSC22D3	0.002162728	0.348743594	0.889	0.798	1	CD44 > 1 & MKI67 < 1 Leuk	TSC22D3
MT-CO3	0.002829123	0.363363437	1	0.994	1	CD44 > 1 & MKI67 < 1 Leuk	MT-CO3
SH3BGRL3	0.003351936	0.478978714	0.778	0.862	1	CD44 > 1 & MKI67 < 1 Leuk	SH3BGRL3
FTH1	0.003634486	0.243669979	0.978	0.996	1	CD44 > 1 & MKI67 < 1 Leuk	FTH1
ST3GAL1	0.003732601	0.419113932	0.156	0.059	1	CD44 > 1 & MKI67 < 1 Leuk	ST3GAL1
MT-CO1	0.004219007	0.289255587	1	0.998	1	CD44 > 1 & MKI67 < 1 Leuk	MT-CO1
BTG1	0.004667933	0.450461526	0.844	0.871	1	CD44 > 1 & MKI67 < 1 Leuk	BTG1
IL17RA	0.004829181	0.376629864	0.133	0.048	1	CD44 > 1 & MKI67 < 1 Leuk	IL17RA
KLF2	0.009313985	0.315687028	0.178	0.076	1	CD44 > 1 & MKI67 < 1 Leuk	KLF2
TUBB2A	0.009423782	0.604634203	0.267	0.149	1	CD44 > 1 & MKI67 < 1 Leuk	TUBB2A
RPLP1	2.56E-07	0.463156777	0.99	0.978	0.009364011	Leuk	RPLP1
LDHB	2.90E-07	0.657359093	0.858	0.556	0.010610459	Leuk	LDHB
GSTP1	3.56E-07	0.728622225	0.867	0.578	0.013016746	Leuk	GSTP1
HSPD1	3.62E-07	0.637643261	0.467	0.067	0.013238503	Leuk	HSPD1
RPS16	4.44E-07	0.513221759	0.962	0.867	0.01623332	Leuk	RPS16
RPL23A	4.65E-07	0.602433357	0.951	0.733	0.017006628	Leuk	RPL23A
RPL14	6.91E-07	0.517324195	0.971	0.844	0.025281553	Leuk	RPL14
RPL4	6.91E-07	0.55840061	0.951	0.778	0.025293333	Leuk	RPL4
HMGA1	1.06E-06	0.603816592	0.505	0.111	0.03896208	Leuk	HMGA1
GAPDH	1.14E-06	0.585615393	0.988	0.933	0.04181531	Leuk	GAPDH
SLC25A6	1.24E-06	0.604219481	0.924	0.667	0.045435175	Leuk	SLC25A6
RPL23	1.34E-06	0.623933714	0.791	0.356	0.04897264	Leuk	RPL23
RPS23	1.53E-06	0.461685425	0.982	0.911	0.05585708	Leuk	RPS23
NME4	1.65E-06	0.475176205	0.421	0.044	0.060491777	Leuk	NME4
RPS5	2.50E-06	0.484977039	0.979	0.933	0.091461729	Leuk	RPS5
SCP2	2.84E-06	0.416204332	0.341	0	0.103929319	Leuk	SCP2
NPM1	3.03E-06	0.557617738	0.927	0.689	0.110976108	Leuk	NPM1
PSMA6	3.05E-06	0.526840169	0.699	0.267	0.111550593	Leuk	PSMA6
MIF	3.09E-06	0.656649658	0.875	0.6	0.113200848	Leuk	MIF
RPLP2	3.72E-06	0.500203911	0.951	0.844	0.136312379	Leuk	RPLP2
ENO1	3.98E-06	0.518468814	0.666	0.244	0.145744339	Leuk	ENO1

PRMT1	6.26E-06	0.443872442	0.494	0.111	0.229196237	Leuk	PRMT1
HMGN2	7.00E-06	1.075335845	0.705	0.356	0.256240491	Leuk	HMGN2
TOMM6	7.20E-06	0.431960091	0.465	0.089	0.263350013	Leuk	TOMM6
RPS6	7.29E-06	0.388033964	0.983	0.956	0.266705263	Leuk	RPS6
BANF1	9.53E-06	0.35694139	0.434	0.067	0.348852743	Leuk	BANF1
PHGDH	1.31E-05	0.426056605	0.307	0	0.480421144	Leuk	PHGDH
ERH	1.33E-05	0.337014277	0.391	0.044	0.485302808	Leuk	ERH
ALYREF	1.47E-05	0.432182045	0.454	0.089	0.536873063	Leuk	ALYREF
EEF1G	1.60E-05	0.451522759	0.953	0.889	0.586100076	Leuk	EEF1G
EIF5A	1.64E-05	0.424352632	0.473	0.111	0.599462998	Leuk	EIF5A
RPL12	1.73E-05	0.441073529	0.975	0.867	0.632655266	Leuk	RPL12
GTF2A2	2.05E-05	0.334735602	0.297	0	0.748644193	Leuk	GTF2A2
SNHG29	2.46E-05	0.526253614	0.95	0.778	0.902211137	Leuk	SNHG29
RPL18A	2.73E-05	0.405963515	0.986	0.933	1	Leuk	RPL18A
UBE2L6	3.00E-05	0.368182649	0.39	0.067	1	Leuk	UBE2L6
PSMB2	3.26E-05	0.331479885	0.41	0.067	1	Leuk	PSMB2
PRMT7	3.55E-05	0.410179876	0.608	0.2	1	Leuk	PRMT7
ADA	3.88E-05	0.489383317	0.584	0.222	1	Leuk	ADA
BLOC1S1	3.91E-05	0.381788837	0.425	0.089	1	Leuk	BLOC1S1
COX8A	4.25E-05	0.469680381	0.581	0.2	1	Leuk	COX8A
RPS12	4.40E-05	0.376925697	0.994	0.956	1	Leuk	RPS12
EEF1B2	4.43E-05	0.420799064	0.959	0.889	1	Leuk	EEF1B2
HDAC2	4.50E-05	0.330236305	0.361	0.044	1	Leuk	HDAC2
RBMX	4.69E-05	0.381518088	0.546	0.178	1	Leuk	RBMX
DENND2D	5.10E-05	0.350477458	0.391	0.067	1	Leuk	DENND2D
SMARCE1	5.60E-05	0.370922481	0.45	0.111	1	Leuk	SMARCE1
TMPO	5.69E-05	0.330042828	0.315	0.022	1	Leuk	TMPO
CCT7	6.30E-05	0.370397084	0.495	0.133	1	Leuk	CCT7
TCF7	6.78E-05	0.297328784	0.809	0.356	1	Leuk	TCF7
OAZ1	7.31E-05	0.441965522	0.649	0.267	1	Leuk	OAZ1
TYMS	8.17E-05	0.52295875	0.331	0.044	1	Leuk	TYMS
GLRX	8.60E-05	0.316772426	0.262	0	1	Leuk	GLRX
LSM4	9.04E-05	0.395880242	0.479	0.133	1	Leuk	LSM4
CHI3L2	9.21E-05	0.586536706	0.679	0.356	1	Leuk	CHI3L2
RPL10A	9.35E-05	0.420236635	0.982	0.911	1	Leuk	RPL10A
RPL35	9.42E-05	0.29704639	0.946	0.911	1	Leuk	RPL35
PPP1R14B	9.67E-05	0.367209895	0.59	0.222	1	Leuk	PPP1R14B
RPL28	0.00010138	0.293366831	0.993	0.956	1	Leuk	RPL28
PHB2	0.000104066	0.426512699	0.586	0.244	1	Leuk	PHB2
ATP5PF	0.000108158	0.363182099	0.527	0.156	1	Leuk	ATP5PF
CNOT7	0.000112716	0.296408333	0.255	0	1	Leuk	CNOT7
EEF1A1	0.000121658	0.32182437	0.993	0.933	1	Leuk	EEF1A1
ATP5F1C	0.000127764	0.32079865	0.489	0.133	1	Leuk	ATP5F1C
NDUFA6	0.000137786	0.314746554	0.357	0.067	1	Leuk	NDUFA6
MRPL51	0.000140557	0.324447504	0.365	0.067	1	Leuk	MRPL51
H2AFX	0.000144165	0.42886439	0.249	0	1	Leuk	H2AFX
RAD23A	0.000146332	0.30919766	0.363	0.067	1	Leuk	RAD23A
ANXA11	0.000147607	0.228809854	0.373	0.067	1	Leuk	ANXA11
RPLP0	0.000170068	0.380904739	0.991	0.933	1	Leuk	RPLP0
RPL15	0.000183108	0.407267316	0.979	0.844	1	Leuk	RPL15
MCM7	0.000189068	0.262057527	0.326	0.044	1	Leuk	MCM7
ARPC5	0.000191298	0.289860234	0.357	0.067	1	Leuk	ARPC5

ATP5PO	0.000200228	0.371290547	0.677	0.333	1	Leuk	ATP5PO
SPCS2	0.000215626	0.280511578	0.317	0.044	1	Leuk	SPCS2
SLC25A5	0.000221842	0.435310723	0.825	0.6	1	Leuk	SLC25A5
RPL37A	0.000226799	0.369668007	0.953	0.889	1	Leuk	RPL37A
NDUFAB1	0.000251649	0.243514017	0.277	0.022	1	Leuk	NDUFAB1
CCT8	0.00025624	0.305759143	0.417	0.111	1	Leuk	CCT8
NME1	0.000260904	0.263589515	0.356	0.067	1	Leuk	NME1
SF3B2	0.000261387	0.336436563	0.552	0.2	1	Leuk	SF3B2
RPL7A	0.000261826	0.299168041	0.987	0.911	1	Leuk	RPL7A
RPL7	0.000265707	0.417722194	0.94	0.8	1	Leuk	RPL7
MRPS24	0.000267528	0.316787248	0.372	0.089	1	Leuk	MRPS24
STMN1	0.000268635	0.543835676	0.758	0.489	1	Leuk	STMN1
PSIP1	0.000276974	0.168369064	0.382	0.089	1	Leuk	PSIP1
RPS18	0.000277067	0.332004249	0.99	0.978	1	Leuk	RPS18
SNU13	0.000284511	0.21275858	0.502	0.178	1	Leuk	SNU13
SSRP1	0.000285886	0.284249697	0.308	0.044	1	Leuk	SSRP1
RPL9	0.000287612	0.41602735	0.965	0.822	1	Leuk	RPL9
RPL27	0.000295769	0.404550548	0.921	0.711	1	Leuk	RPL27
SELENOW	0.000296844	0.503060117	0.84	0.6	1	Leuk	SELENOW
KIF2A	0.000297799	0.267870299	0.272	0.022	1	Leuk	KIF2A
PARK7	0.000314956	0.347781516	0.507	0.178	1	Leuk	PARK7
BABAM1	0.000322496	0.246998717	0.228	0	1	Leuk	BABAM1
ARL6IP4	0.000327004	0.31062052	0.493	0.156	1	Leuk	ARL6IP4
CNPY2	0.000343224	0.255717134	0.308	0.044	1	Leuk	CNPY2
RNASEK	0.000344113	0.242658914	0.596	0.222	1	Leuk	RNASEK
RANBP1	0.000347205	0.290807849	0.348	0.067	1	Leuk	RANBP1
SET	0.000348848	0.311746275	0.685	0.333	1	Leuk	SET
ATP5F1D	0.000354675	0.242170725	0.583	0.222	1	Leuk	ATP5F1D
NHP2	0.000354851	0.286677084	0.469	0.156	1	Leuk	NHP2
ANAPC11	0.000355015	0.333508254	0.435	0.133	1	Leuk	ANAPC11
EIF3F	0.000360297	0.344831767	0.855	0.533	1	Leuk	EIF3F
BST2	0.000362853	0.270541281	0.341	0.067	1	Leuk	BST2
BEX3	0.000369473	0.313722168	0.438	0.133	1	Leuk	BEX3
PPP1CA	0.000371359	0.249313692	0.546	0.2	1	Leuk	PPP1CA
ECH1	0.0003789	0.284954501	0.372	0.089	1	Leuk	ECH1
PGLS	0.000396937	0.308988598	0.501	0.178	1	Leuk	PGLS
CD1E	0.000407961	0.325668432	0.221	0	1	Leuk	CD1E
PPIA	0.000415798	0.506327691	0.895	0.778	1	Leuk	PPIA
CCT3	0.000422764	0.266460194	0.475	0.156	1	Leuk	CCT3
HIGD2A	0.000451507	0.324596392	0.598	0.244	1	Leuk	HIGD2A
CDK4	0.000451863	0.165701383	0.262	0.022	1	Leuk	CDK4
HNRNPD	0.000452734	0.291458031	0.545	0.2	1	Leuk	HNRNPD
SERBP1	0.000460616	0.239080626	0.556	0.2	1	Leuk	SERBP1
IMPDH2	0.000462056	0.338720318	0.616	0.267	1	Leuk	IMPDH2
BRK1	0.000462907	0.316915695	0.481	0.178	1	Leuk	BRK1
FUNDC2	0.000467933	0.295275856	0.451	0.133	1	Leuk	FUNDC2
SUPT16H	0.000468551	0.24609343	0.26	0.022	1	Leuk	SUPT16H
SMARCC1	0.000475978	0.305844886	0.469	0.156	1	Leuk	SMARCC1
ANP32B	0.000487261	0.427171119	0.702	0.378	1	Leuk	ANP32B
RPL5	0.000494328	0.346421203	0.979	0.933	1	Leuk	RPL5
ANXA5	0.000499478	0.270969828	0.294	0.044	1	Leuk	ANXA5
DTYMK	0.000499511	0.238473447	0.216	0	1	Leuk	DTYMK

ATP5MC1	0.000500395	0.282225163	0.361	0.089	1	Leuk	ATP5MC1
SNRNP200	0.000514297	0.286486858	0.297	0.044	1	Leuk	SNRNP200
ADRM1	0.000533558	0.187845131	0.257	0.022	1	Leuk	ADRM1
RAN	0.000534533	0.411880425	0.619	0.311	1	Leuk	RAN
RSL24D1	0.000536435	0.272985199	0.461	0.156	1	Leuk	RSL24D1
SEM1	0.000540736	0.266776638	0.368	0.089	1	Leuk	SEM1
HTATSF1	0.00054328	0.250673496	0.253	0.022	1	Leuk	HTATSF1
DANCR	0.00054436	0.259861431	0.41	0.111	1	Leuk	DANCR
PCLAF	0.000546837	0.323389505	0.214	0	1	Leuk	PCLAF
HMGB2	0.00054749	0.779109533	0.343	0.089	1	Leuk	HMGB2
CKLF	0.000550044	0.297840542	0.519	0.2	1	Leuk	CKLF
ATP5MC2	0.000551307	0.355104414	0.898	0.689	1	Leuk	ATP5MC2
RPS21	0.000567256	0.351107256	0.963	0.8	1	Leuk	RPS21
NAPA	0.000569044	0.219976651	0.255	0.022	1	Leuk	NAPA
CD1B	0.00056922	0.304636311	0.212	0	1	Leuk	CD1B
XRN2	0.000607109	0.259359985	0.289	0.044	1	Leuk	XRN2
PCBP2	0.000652959	0.298576938	0.745	0.356	1	Leuk	PCBP2
CPSF6	0.000653834	0.263718052	0.286	0.044	1	Leuk	CPSF6
COX5A	0.000672872	0.168230127	0.519	0.2	1	Leuk	COX5A
RPSA	0.00067539	0.352207789	0.972	0.867	1	Leuk	RPSA
YBX1	0.000685138	0.439411483	0.854	0.578	1	Leuk	YBX1
EIF3A	0.000705352	0.313648287	0.475	0.156	1	Leuk	EIF3A
UBL5	0.000705786	0.277342218	0.599	0.244	1	Leuk	UBL5
SNHG16	0.000708852	0.229317292	0.249	0.022	1	Leuk	SNHG16
SCCPDH	0.000722509	0.208127516	0.248	0.022	1	Leuk	SCCPDH
PRKDC	0.000730318	0.134035738	0.29	0.044	1	Leuk	PRKDC
TIMM13	0.000740984	0.280268876	0.385	0.111	1	Leuk	TIMM13
FKBP4	0.000746224	0.216842631	0.248	0.022	1	Leuk	FKBP4
NEDD8	0.000762023	0.186729089	0.5	0.178	1	Leuk	NEDD8
MARCKSL1	0.000772176	0.437009491	0.745	0.422	1	Leuk	MARCKSL1
MGST3	0.000775413	0.228412549	0.244	0.022	1	Leuk	MGST3
NDUFA11	0.000781104	0.331501849	0.65	0.311	1	Leuk	NDUFA11
PIH1D1	0.000782949	0.227193339	0.244	0.022	1	Leuk	PIH1D1
ABRACL	0.000788989	0.220832968	0.36	0.089	1	Leuk	ABRACL
RACK1	0.000816284	0.323390436	0.983	0.911	1	Leuk	RACK1
NASP	0.000817167	0.31434213	0.391	0.111	1	Leuk	NASP
RPL27A	0.000824664	0.423762577	0.855	0.556	1	Leuk	RPL27A
VAMP8	0.000852195	0.22901284	0.279	0.044	1	Leuk	VAMP8
CAPZA1	0.000872276	0.232172259	0.201	0	1	Leuk	CAPZA1
LSM3	0.00087318	0.213132779	0.283	0.044	1	Leuk	LSM3
HNRNPK	0.000874846	0.342473699	0.696	0.333	1	Leuk	HNRNPK
HMGN1	0.000918028	0.142517983	0.663	0.333	1	Leuk	HMGN1
SHKBP1	0.000920697	0.244568187	0.238	0.022	1	Leuk	SHKBP1
GPX4	0.000951506	0.181861498	0.621	0.267	1	Leuk	GPX4
UBALD2	0.000954872	0.24986953	0.359	0.089	1	Leuk	UBALD2
MYB	0.000964759	0.273747652	0.482	0.178	1	Leuk	MYB
MAD2L2	0.000982905	0.250576507	0.352	0.089	1	Leuk	MAD2L2
RPL19	0.000997011	0.318987268	0.988	0.911	1	Leuk	RPL19
RPN1	0.001018717	0.229368691	0.319	0.067	1	Leuk	RPN1
KRTCAP2	0.001071315	0.218754844	0.454	0.156	1	Leuk	KRTCAP2
MZT1	0.001071368	0.207789125	0.195	0	1	Leuk	MZT1
RPS13	0.001103012	0.344334226	0.976	0.911	1	Leuk	RPS13

RPS8	0.001104198	0.272781904	0.992	0.933	1	Leuk	RPS8
G3BP1	0.001138724	0.234212007	0.271	0.044	1	Leuk	G3BP1
CCT5	0.001156389	0.207076447	0.306	0.067	1	Leuk	CCT5
ITGAE	0.001164159	0.109807867	0.546	0.2	1	Leuk	ITGAE
EBNA1BP2	0.001192309	0.20221163	0.192	0	1	Leuk	EBNA1BP2
CCDC88A	0.001192978	0.279991764	0.299	0.067	1	Leuk	CCDC88A
ILF2	0.001193571	0.148447485	0.639	0.267	1	Leuk	ILF2
SRM	0.001206141	0.210933756	0.355	0.089	1	Leuk	SRM
UHRF1	0.001215657	0.222477692	0.191	0	1	Leuk	UHRF1
RPL36	0.001216768	0.388193622	0.956	0.756	1	Leuk	RPL36
FKBP8	0.001241024	0.163170249	0.524	0.2	1	Leuk	FKBP8
PGAM1	0.001248253	0.2648387	0.463	0.178	1	Leuk	PGAM1
FBL	0.001267973	0.28317241	0.534	0.222	1	Leuk	FBL
RPS15	0.001268505	0.264031553	0.982	0.911	1	Leuk	RPS15
PRPF19	0.001279947	0.152750712	0.233	0.022	1	Leuk	PRPF19
RPS11	0.001289291	0.320056581	0.915	0.689	1	Leuk	RPS11
DCTPP1	0.001300867	0.196245837	0.19	0	1	Leuk	DCTPP1
DAZAP1	0.001305566	0.202527618	0.354	0.089	1	Leuk	DAZAP1
RAB7A	0.001311334	0.208458969	0.271	0.044	1	Leuk	RAB7A
GXYLT2	0.00132078	0.201839128	0.272	0.044	1	Leuk	GXYLT2
TBCD	0.00133041	0.221123999	0.229	0.022	1	Leuk	TBCD
CUEDC2	0.001399882	0.184565065	0.312	0.067	1	Leuk	CUEDC2
NREP	0.001405856	0.230464312	0.34	0.089	1	Leuk	NREP
PAFAH1B3	0.001409322	0.149111501	0.422	0.133	1	Leuk	PAFAH1B3
GYPC	0.001417588	0.23358778	0.494	0.178	1	Leuk	GYPC
PUF60	0.00142263	0.23244854	0.377	0.111	1	Leuk	PUF60
CORO1A	0.001430557	0.102411284	0.575	0.222	1	Leuk	CORO1A
ICAM2	0.001449915	0.160234325	0.41	0.133	1	Leuk	ICAM2
HNRNPA2B1	0.001460251	0.408263756	0.894	0.6	1	Leuk	HNRNPA2B1
PGD	0.001477974	0.112344544	0.392	0.111	1	Leuk	PGD
EIF3D	0.001484081	0.144931528	0.633	0.289	1	Leuk	EIF3D
LAGE3	0.00148783	0.190124845	0.265	0.044	1	Leuk	LAGE3
RBM42	0.001492615	0.183609945	0.229	0.022	1	Leuk	RBM42
COX6C	0.001492943	0.376148944	0.72	0.4	1	Leuk	COX6C
GDI2	0.001496383	0.184990754	0.377	0.111	1	Leuk	GDI2
SKP1	0.001514374	0.25223162	0.678	0.333	1	Leuk	SKP1
SMC2	0.001547012	0.223240443	0.185	0	1	Leuk	SMC2
CKS1B	0.001547012	0.232552542	0.185	0	1	Leuk	CKS1B
SNRPG	0.00154989	0.173694215	0.307	0.067	1	Leuk	SNRPG
TUBB	0.001564066	0.605502045	0.848	0.644	1	Leuk	TUBB
HSP90AB1	0.001595721	0.318495542	0.945	0.733	1	Leuk	HSP90AB1
NUDT8	0.001596414	0.14852327	0.264	0.044	1	Leuk	NUDT8
HNRNPAB	0.001607594	0.210851735	0.477	0.178	1	Leuk	HNRNPAB
UGP2	0.001627814	0.229612285	0.225	0.022	1	Leuk	UGP2
RNF181	0.001691652	0.183132145	0.262	0.044	1	Leuk	RNF181
NUDC	0.001695528	0.188086532	0.341	0.089	1	Leuk	NUDC
ZCRB1	0.00171256	0.183549885	0.265	0.044	1	Leuk	ZCRB1
SNRPA	0.001721679	0.189031377	0.44	0.156	1	Leuk	SNRPA
C1orf43	0.001727594	0.209566361	0.337	0.089	1	Leuk	C1orf43
ANP32E	0.001772445	0.235260823	0.259	0.044	1	Leuk	ANP32E
FAM32A	0.001788783	0.188155882	0.223	0.022	1	Leuk	FAM32A
SNRPB	0.001789588	0.246617091	0.597	0.267	1	Leuk	SNRPB

HADH	0.001808263	0.123936746	0.223	0.022	1	Leuk	HADH
RPS2	0.001809353	0.260244299	0.987	0.978	1	Leuk	RPS2
RPS4X	0.001817086	0.28770995	0.984	0.978	1	Leuk	RPS4X
POLR2I	0.001842803	0.176537664	0.263	0.044	1	Leuk	POLR2I
DCAF7	0.001871131	0.188533899	0.222	0.022	1	Leuk	DCAF7
ATP5MG	0.001872649	0.262357808	0.848	0.556	1	Leuk	ATP5MG
MCM6	0.001908622	0.186583693	0.179	0	1	Leuk	MCM6
DYNLL1	0.001921538	0.291012012	0.348	0.111	1	Leuk	DYNLL1
PPAN	0.001967144	0.109751554	0.297	0.067	1	Leuk	PPAN
ATP5PD	0.002013127	0.185730644	0.375	0.111	1	Leuk	ATP5PD
LSM2	0.002024852	0.159378985	0.463	0.178	1	Leuk	LSM2
AP2M1	0.002031672	0.15229897	0.48	0.178	1	Leuk	AP2M1
CHRAC1	0.002040379	0.206905484	0.216	0.022	1	Leuk	CHRAC1
MFNG	0.002042397	0.245176856	0.288	0.067	1	Leuk	MFNG
PSMB1	0.002045174	0.171379639	0.508	0.2	1	Leuk	PSMB1
H3F3A	0.002067384	0.333558352	0.951	0.844	1	Leuk	H3F3A
RFC2	0.002078755	0.181341089	0.176	0	1	Leuk	RFC2
YWHAQ	0.002083316	0.187105026	0.564	0.244	1	Leuk	YWHAQ
ANAPC5	0.002093066	0.177621012	0.338	0.089	1	Leuk	ANAPC5
SSBP1	0.002117838	0.213300906	0.405	0.133	1	Leuk	SSBP1
RPL24	0.002120839	0.325704615	0.966	0.778	1	Leuk	RPL24
AKR1C3	0.002135293	0.232328643	0.36	0.111	1	Leuk	AKR1C3
DNTT	0.002144884	0.367155302	0.747	0.444	1	Leuk	DNTT
TAGLN2	0.002146028	0.244437676	0.719	0.333	1	Leuk	TAGLN2
CCT2	0.002155485	0.243357955	0.485	0.2	1	Leuk	CCT2
UQCRB	0.002161069	0.270684546	0.673	0.333	1	Leuk	UQCRB
DBI	0.002163628	0.205091633	0.364	0.111	1	Leuk	DBI
ATP5F1A	0.002164047	0.282904316	0.641	0.289	1	Leuk	ATP5F1A
GAS5	0.002178898	0.235680628	0.88	0.556	1	Leuk	GAS5
GPS1	0.002201484	0.207191809	0.213	0.022	1	Leuk	GPS1
SMIM24	0.002210905	0.231512875	0.332	0.089	1	Leuk	SMIM24
PSMD8	0.002224165	0.112563728	0.494	0.178	1	Leuk	PSMD8
VPS28	0.002233901	0.208775421	0.444	0.156	1	Leuk	VPS28
TM7SF3	0.002234468	0.23254993	0.322	0.089	1	Leuk	TM7SF3
C19orf53	0.002254526	0.292513916	0.546	0.244	1	Leuk	C19orf53
COX14	0.002261172	0.157169687	0.258	0.044	1	Leuk	COX14
LBH	0.002265415	0.238423078	0.215	0.022	1	Leuk	LBH
ROCK1	0.002271999	0.171478092	0.399	0.133	1	Leuk	ROCK1
TFAM	0.002291204	0.117743941	0.217	0.022	1	Leuk	TFAM
PECAM1	0.002292985	0.226850438	0.252	0.044	1	Leuk	PECAM1
PHF14	0.002300973	0.128406386	0.296	0.067	1	Leuk	PHF14
GSTO1	0.00237718	0.19300661	0.287	0.067	1	Leuk	GSTO1
PSMC3	0.002379188	0.17720256	0.33	0.089	1	Leuk	PSMC3
IK	0.002433789	0.207690607	0.287	0.067	1	Leuk	IK
STOML2	0.00244928	0.182130521	0.291	0.067	1	Leuk	STOML2
C19orf48	0.002481761	0.118076201	0.336	0.089	1	Leuk	C19orf48
ERG	0.00250203	0.200894216	0.253	0.044	1	Leuk	ERG
ZNRD1	0.002516155	0.19990287	0.25	0.044	1	Leuk	ZNRD1
DHPS	0.002520201	0.19992547	0.286	0.067	1	Leuk	DHPS
CCDC138	0.002534018	0.168293209	0.17	0	1	Leuk	CCDC138
CD2	0.002548349	0.25897678	0.315	0.089	1	Leuk	CD2
WDR83OS	0.002573097	0.249846347	0.416	0.156	1	Leuk	WDR83OS

ATP5MF	0.002584942	0.217697267	0.522	0.222	1	Leuk	ATP5MF
MRPL20	0.00259832	0.207551715	0.245	0.044	1	Leuk	MRPL20
UBA52	0.002605544	0.338250968	0.925	0.8	1	Leuk	UBA52
WASF2	0.002628507	0.20064671	0.475	0.178	1	Leuk	WASF2
MRPL57	0.002631001	0.139214538	0.473	0.178	1	Leuk	MRPL57
TPGS2	0.00267002	0.20614884	0.362	0.111	1	Leuk	TPGS2
DNMT3A	0.002674668	0.118343051	0.212	0.022	1	Leuk	DNMT3A
FSCN1	0.002678418	0.150986191	0.326	0.089	1	Leuk	FSCN1
SMC4	0.002680611	0.23976903	0.249	0.044	1	Leuk	SMC4
CENPF	0.002680715	0.394929983	0.169	0	1	Leuk	CENPF
CD3E	0.002711842	0.27446072	0.942	0.756	1	Leuk	CD3E
CARHSP1	0.002719883	0.191210336	0.286	0.067	1	Leuk	CARHSP1
CLPP	0.002721657	0.164571015	0.411	0.133	1	Leuk	CLPP
SNRPE	0.002820297	0.153795092	0.434	0.156	1	Leuk	SNRPE
TMEM243	0.00284691	0.231582762	0.205	0.022	1	Leuk	TMEM243
MICOS10	0.002873898	0.187054381	0.245	0.044	1	Leuk	MICOS10
MRPS21	0.002899203	0.197993839	0.396	0.133	1	Leuk	MRPS21
BSG	0.002900476	0.163454452	0.451	0.178	1	Leuk	BSG
NCOR1	0.0029052	0.162998406	0.392	0.133	1	Leuk	NCOR1
SELENOH	0.002906267	0.169141682	0.582	0.267	1	Leuk	SELENOH
PSMD1	0.00291611	0.177557477	0.166	0	1	Leuk	PSMD1
ARRDC2	0.002921606	0.203795981	0.247	0.044	1	Leuk	ARRDC2
NDUFS8	0.002961604	0.227435726	0.46	0.178	1	Leuk	NDUFS8
ADSL	0.002962386	0.202405535	0.245	0.044	1	Leuk	ADSL
DDX54	0.002971045	0.181898073	0.166	0	1	Leuk	DDX54
BUD31	0.002985487	0.188998881	0.321	0.089	1	Leuk	BUD31
NRDC	0.003012476	0.193591004	0.35	0.111	1	Leuk	NRDC
EVL	0.003039067	0.179891313	0.699	0.356	1	Leuk	EVL
PKN1	0.003039339	0.186717899	0.284	0.067	1	Leuk	PKN1
IL7R	0.003048056	0.201237014	0.385	0.133	1	Leuk	IL7R
BAZ1B	0.003055948	0.201796434	0.242	0.044	1	Leuk	BAZ1B
CAT	0.003064847	0.18667687	0.206	0.022	1	Leuk	CAT
GPAA1	0.003065208	0.19914695	0.203	0.022	1	Leuk	GPAA1
GADD45GIP1	0.003153167	0.184579663	0.358	0.111	1	Leuk	GADD45GIP1
ZMAT2	0.003207386	0.189440667	0.204	0.022	1	Leuk	ZMAT2
NDUFB3	0.003209144	0.154229217	0.206	0.022	1	Leuk	NDUFB3
PDAP1	0.003220342	0.142526252	0.287	0.067	1	Leuk	PDAP1
CFDP1	0.003224709	0.219930628	0.242	0.044	1	Leuk	CFDP1
COA4	0.003260931	0.158106052	0.163	0	1	Leuk	COA4
RGS9	0.003291367	0.159332916	0.163	0	1	Leuk	RGS9
MAGOH	0.003307735	0.151284396	0.325	0.089	1	Leuk	MAGOH
PSME1	0.00332677	0.359782653	0.746	0.467	1	Leuk	PSME1
ATP5MD	0.003366378	0.114924615	0.398	0.133	1	Leuk	ATP5MD
MCM3	0.003419597	0.197538302	0.202	0.022	1	Leuk	MCM3
POLR2F	0.003436092	0.179587762	0.241	0.044	1	Leuk	POLR2F
METTL5	0.003479776	0.168684175	0.161	0	1	Leuk	METTL5
MCUR1	0.003512167	0.158473266	0.161	0	1	Leuk	MCUR1
TMEM109	0.003546277	0.155600501	0.203	0.022	1	Leuk	TMEM109
FAM120AOS	0.003608125	0.10121445	0.203	0.022	1	Leuk	FAM120AOS
CTNNBIP1	0.00361108	0.164138627	0.16	0	1	Leuk	CTNNBIP1
HNRNPF	0.003615498	0.170297579	0.36	0.111	1	Leuk	HNRNPF
SMARCC2	0.003622275	0.215563728	0.273	0.067	1	Leuk	SMARCC2

HDAC3	0.00363514	0.155244587	0.203	0.022	1	Leuk	HDAC3
PTCRA	0.003642948	0.335230614	0.267	0.067	1	Leuk	PTCRA
DPM3	0.003644641	0.178331444	0.16	0	1	Leuk	DPM3
TUBA1B	0.003663954	0.886436328	0.689	0.422	1	Leuk	TUBA1B
PRR13	0.003730591	0.206869193	0.492	0.222	1	Leuk	PRR13
SNRPC	0.003736214	0.19387401	0.481	0.2	1	Leuk	SNRPC
GGCT	0.003747127	0.153470668	0.159	0	1	Leuk	GGCT
DDOST	0.003749807	0.199001719	0.485	0.2	1	Leuk	DDOST
HSPE1	0.003774609	0.156759784	0.318	0.089	1	Leuk	HSPE1
DCXR	0.003801019	0.182171511	0.237	0.044	1	Leuk	DCXR
UQCRQ	0.003852325	0.180277853	0.493	0.2	1	Leuk	UQCRQ
NAXE	0.003893591	0.166932506	0.311	0.089	1	Leuk	NAXE
MMADHC	0.003910184	0.123963054	0.276	0.067	1	Leuk	MMADHC
ANAPC15	0.003950209	0.163396347	0.276	0.067	1	Leuk	ANAPC15
BBX	0.003955163	0.199259965	0.198	0.022	1	Leuk	BBX
MAD2L1	0.00396045	0.159369322	0.157	0	1	Leuk	MAD2L1
HSP90AA1	0.003975568	0.27252184	0.898	0.667	1	Leuk	HSP90AA1
PSMC1	0.003989843	0.179727929	0.196	0.022	1	Leuk	PSMC1
AAMP	0.004035717	0.174072357	0.197	0.022	1	Leuk	AAMP
LPXN	0.004043828	0.150555451	0.199	0.022	1	Leuk	LPXN
HSPB1	0.004045528	0.264693947	0.588	0.311	1	Leuk	HSPB1
ATP6V1F	0.004061603	0.195291453	0.348	0.111	1	Leuk	ATP6V1F
CENPM	0.00414707	0.172624847	0.156	0	1	Leuk	CENPM
P4HB	0.004192217	0.2950125	0.524	0.244	1	Leuk	P4HB
PHB	0.004254277	0.167699198	0.311	0.089	1	Leuk	PHB
CCT6A	0.004277699	0.20375416	0.425	0.156	1	Leuk	CCT6A
MRPL3	0.004295593	0.13419123	0.198	0.022	1	Leuk	MRPL3
LGALS3BP	0.004302945	0.203233007	0.303	0.089	1	Leuk	LGALS3BP
CISD3	0.004307141	0.139229833	0.238	0.044	1	Leuk	CISD3
GMFG	0.004314834	0.264370827	0.83	0.444	1	Leuk	GMFG
PPA1	0.004315066	0.219370273	0.439	0.178	1	Leuk	PPA1
PSMA2	0.004329771	0.188508794	0.428	0.156	1	Leuk	PSMA2
PPDPF	0.004334136	0.134504434	0.682	0.378	1	Leuk	PPDPF
DNMT1	0.00436259	0.16096187	0.237	0.044	1	Leuk	DNMT1
MRPL37	0.004368928	0.130414215	0.238	0.044	1	Leuk	MRPL37
HIST1H4C	0.004383807	0.595722202	0.611	0.333	1	Leuk	HIST1H4C
LSM12	0.004392761	0.169706832	0.197	0.022	1	Leuk	LSM12
UBE2G1	0.004463326	0.189324169	0.154	0	1	Leuk	UBE2G1
IER3IP1	0.004463326	0.147169863	0.154	0	1	Leuk	IER3IP1
GMPS	0.004481045	0.138775348	0.196	0.022	1	Leuk	GMPS
RPL31	0.00449737	0.352502365	0.906	0.622	1	Leuk	RPL31
SNRPD2	0.004508178	0.318750523	0.793	0.533	1	Leuk	SNRPD2
COX5B	0.004565433	0.226228849	0.59	0.267	1	Leuk	COX5B
CDT1	0.004567492	0.176720196	0.235	0.044	1	Leuk	CDT1
FIBP	0.004645134	0.124997978	0.316	0.089	1	Leuk	FIBP
HYI	0.004666582	0.17489585	0.193	0.022	1	Leuk	HYI
SNRPF	0.004715567	0.180223282	0.455	0.178	1	Leuk	SNRPF
SLC17A9	0.004715824	0.136720158	0.195	0.022	1	Leuk	SLC17A9
C12orf10	0.004748373	0.139877896	0.232	0.044	1	Leuk	C12orf10
STX16	0.00475892	0.207036424	0.152	0	1	Leuk	STX16
PYCR2	0.004776501	0.162929116	0.191	0.022	1	Leuk	PYCR2
BTF3	0.004819782	0.291100699	0.929	0.733	1	Leuk	BTF3

SELENOF	0.004837224	0.140213575	0.354	0.111	1	Leuk	SELENOF
RUVBL1	0.004846769	0.138232306	0.151	0	1	Leuk	RUVBL1
SNRPD1	0.004858714	0.205575051	0.412	0.156	1	Leuk	SNRPD1
CCDC28B	0.004891275	0.154954382	0.151	0	1	Leuk	CCDC28B
VBP1	0.004891275	0.140980016	0.151	0	1	Leuk	VBP1
HDAC1	0.004966912	0.141482319	0.348	0.111	1	Leuk	HDAC1
HSP90B1	0.004981646	0.189986993	0.507	0.222	1	Leuk	HSP90B1
CYB5R3	0.004982007	0.17228795	0.301	0.089	1	Leuk	CYB5R3
SF3B4	0.004984114	0.154123371	0.307	0.089	1	Leuk	SF3B4
IDH2	0.004987277	0.230853373	0.65	0.333	1	Leuk	IDH2
MAL	0.004990393	0.408579942	0.893	0.689	1	Leuk	MAL
LYPLA1	0.005009097	0.152659008	0.193	0.022	1	Leuk	LYPLA1
PRDX2	0.005030655	0.230701461	0.485	0.222	1	Leuk	PRDX2
RPS26	0.005051661	0.279822925	0.987	0.889	1	Leuk	RPS26
COMMD1	0.005052756	0.176937428	0.265	0.067	1	Leuk	COMMD1
MRPL34	0.005116561	0.14238693	0.192	0.022	1	Leuk	MRPL34
RPS25	0.005139699	0.288137589	0.975	0.867	1	Leuk	RPS25
EIF4EBP1	0.005237	0.151810616	0.191	0.022	1	Leuk	EIF4EBP1
MKI67	0.005261724	0.369377994	0.149	0	1	Leuk	MKI67
GSK3B	0.005291345	0.183267679	0.39	0.156	1	Leuk	GSK3B
SNRPA1	0.005369947	0.171367697	0.229	0.044	1	Leuk	SNRPA1
REX1BD	0.005379837	0.125167569	0.301	0.089	1	Leuk	REX1BD
PCM1	0.005409711	0.126865665	0.387	0.133	1	Leuk	PCM1
FADS2	0.00542399	0.100274102	0.191	0.022	1	Leuk	FADS2
FEN1	0.005456908	0.164940424	0.148	0	1	Leuk	FEN1
TXN2	0.005479436	0.164860866	0.262	0.067	1	Leuk	TXN2
CACYBP	0.00548154	0.144728741	0.268	0.067	1	Leuk	CACYBP
GNA15	0.005498354	0.312049991	0.78	0.467	1	Leuk	GNA15
DGCR6L	0.005501639	0.128180037	0.19	0.022	1	Leuk	DGCR6L
RBCK1	0.00554435	0.187985366	0.261	0.067	1	Leuk	RBCK1
ALDOA	0.005546902	0.231070767	0.568	0.289	1	Leuk	ALDOA
NABP2	0.005557089	0.157514896	0.147	0	1	Leuk	NABP2
AP3B1	0.005557089	0.167803268	0.147	0	1	Leuk	AP3B1
TSPAN7	0.005592799	0.130368487	0.426	0.156	1	Leuk	TSPAN7
PSMD4	0.005594772	0.125483176	0.344	0.111	1	Leuk	PSMD4
TOMM40	0.005668545	0.178861128	0.26	0.067	1	Leuk	TOMM40
RNF220	0.005761311	0.1869959	0.184	0.022	1	Leuk	RNF220
HPRT1	0.005772696	0.114734328	0.189	0.022	1	Leuk	HPRT1
SLC25A1	0.005797727	0.177162955	0.185	0.022	1	Leuk	SLC25A1
ACIN1	0.005810872	0.181915324	0.263	0.067	1	Leuk	ACIN1
CALM2	0.005821001	0.160205346	0.594	0.267	1	Leuk	CALM2
PMF1	0.005941428	0.112013237	0.302	0.089	1	Leuk	PMF1
TALDO1	0.005991857	0.255651178	0.614	0.311	1	Leuk	TALDO1
CD38	0.006008927	0.147383902	0.187	0.022	1	Leuk	CD38
EIF2S2	0.006051277	0.101999933	0.266	0.067	1	Leuk	EIF2S2
RPS17	0.006117012	0.257348467	0.778	0.467	1	Leuk	RPS17
CHD4	0.006146575	0.163313427	0.296	0.089	1	Leuk	CHD4
SEPTIN9	0.006154388	0.14674668	0.442	0.178	1	Leuk	SEPTIN9
RPS3A	0.006161445	0.26724497	0.988	0.933	1	Leuk	RPS3A
FDPS	0.006210547	0.188763984	0.37	0.133	1	Leuk	FDPS
UROS	0.006234079	0.137471128	0.187	0.022	1	Leuk	UROS
UQCR10	0.006256085	0.217078878	0.508	0.222	1	Leuk	UQCR10

CDK2AP2	0.006265117	0.204229726	0.329	0.111	1	Leuk	CDK2AP2
NAA38	0.006271145	0.116180349	0.267	0.067	1	Leuk	NAA38
XPO1	0.00629353	0.153366232	0.185	0.022	1	Leuk	XPO1
MCM5	0.006320028	0.170023346	0.221	0.044	1	Leuk	MCM5
EPRS	0.006323973	0.172547533	0.258	0.067	1	Leuk	EPRS
SMC3	0.006387229	0.1440649	0.296	0.089	1	Leuk	SMC3
EIF3E	0.006391786	0.123000455	0.487	0.2	1	Leuk	EIF3E
SSBP4	0.006416966	0.160124543	0.339	0.111	1	Leuk	SSBP4
H2AFV	0.006471131	0.276242548	0.559	0.289	1	Leuk	H2AFV
EIF4EBP2	0.006490919	0.163601753	0.182	0.022	1	Leuk	EIF4EBP2
FAHD2A	0.006541861	0.153001963	0.142	0	1	Leuk	FAHD2A
LTA4H	0.00656513	0.172543108	0.221	0.044	1	Leuk	LTA4H
TXNDC12	0.006601224	0.145075211	0.142	0	1	Leuk	TXNDC12
TPRKB	0.006611514	0.131396636	0.185	0.022	1	Leuk	TPRKB
ITGB2	0.006617722	0.112652989	0.221	0.044	1	Leuk	ITGB2
NCL	0.006628083	0.328969277	0.7	0.422	1	Leuk	NCL
EIF2AK4	0.006661105	0.158352457	0.142	0	1	Leuk	EIF2AK4
SNHG3	0.006708798	0.266364011	0.407	0.178	1	Leuk	SNHG3
UPF3A	0.006772972	0.174441357	0.254	0.067	1	Leuk	UPF3A
AFF3	0.006784906	0.177839042	0.183	0.022	1	Leuk	AFF3
HIST1H2BK	0.006804638	0.119418472	0.295	0.089	1	Leuk	HIST1H2BK
OCIAD2	0.006863785	0.187369149	0.5	0.222	1	Leuk	OCIAD2
VDAC2	0.006887751	0.148781817	0.408	0.156	1	Leuk	VDAC2
PRDX6	0.00689094	0.102214506	0.494	0.222	1	Leuk	PRDX6
NELFCD	0.006900699	0.116045754	0.259	0.067	1	Leuk	NELFCD
CIAO3	0.006903685	0.142380278	0.223	0.044	1	Leuk	CIAO3
NUTM2A-AS1	0.006905767	0.154783014	0.218	0.044	1	Leuk	NUTM2A-AS1
GABARAPL2	0.006918718	0.153327066	0.297	0.089	1	Leuk	GABARAPL2
TAF9	0.00695906	0.108128426	0.289	0.089	1	Leuk	TAF9
TK1	0.006968431	0.169110846	0.14	0	1	Leuk	TK1
FKBP1A	0.006988222	0.143638272	0.472	0.2	1	Leuk	FKBP1A
TUFM	0.007040403	0.151644087	0.547	0.244	1	Leuk	TUFM
TSEN15	0.007095142	0.149737609	0.14	0	1	Leuk	TSEN15
SOCS3	0.007100899	0.165261502	0.182	0.022	1	Leuk	SOCS3
EIF4A1	0.007142458	0.206266321	0.747	0.444	1	Leuk	EIF4A1
MTHFD1	0.007159325	0.138987469	0.14	0	1	Leuk	MTHFD1
ZC3H7A	0.007159325	0.158015336	0.14	0	1	Leuk	ZC3H7A
NDUFB2	0.007449258	0.161649153	0.404	0.156	1	Leuk	NDUFB2
PFKL	0.007488711	0.133870092	0.138	0	1	Leuk	PFKL
WDR34	0.007521486	0.163786718	0.177	0.022	1	Leuk	WDR34
ZC3H18	0.007556315	0.157281002	0.138	0	1	Leuk	ZC3H18
STAG2	0.007603976	0.159039172	0.217	0.044	1	Leuk	STAG2
TIMP2	0.007637283	0.175106215	0.216	0.044	1	Leuk	TIMP2
EIF3B	0.007673661	0.109363764	0.329	0.111	1	Leuk	EIF3B
TRIM44	0.00769329	0.15932699	0.137	0	1	Leuk	TRIM44
CHMP4A	0.007708382	0.168485024	0.213	0.044	1	Leuk	CHMP4A
USB1	0.007762671	0.141854675	0.137	0	1	Leuk	USB1
PRPF31	0.007773499	0.151054207	0.218	0.044	1	Leuk	PRPF31
CLNS1A	0.007794993	0.164203584	0.327	0.111	1	Leuk	CLNS1A
NUTF2	0.007880247	0.128207993	0.374	0.133	1	Leuk	NUTF2
SRA1	0.007903243	0.146577986	0.137	0	1	Leuk	SRA1
IL4	0.007903243	0.135887223	0.137	0	1	Leuk	IL4

SINHCAF	0.007904059	0.159936733	0.329	0.111	1	Leuk	SINHCAF
ATP6V0E1	0.007922688	0.144638735	0.178	0.022	1	Leuk	ATP6V0E1
UBE2E3	0.007991894	0.138538574	0.218	0.044	1	Leuk	UBE2E3
ETFA	0.008046265	0.127774772	0.136	0	1	Leuk	ETFA
MMP24OS	0.008048788	0.206605844	0.244	0.067	1	Leuk	MMP24OS
PFDN1	0.00805992	0.14085468	0.178	0.022	1	Leuk	PFDN1
BAD	0.008176738	0.176693204	0.211	0.044	1	Leuk	BAD
OTUD6B-AS1	0.008191776	0.144348476	0.136	0	1	Leuk	OTUD6B-AS1
SNHG30	0.008191776	0.136012429	0.136	0	1	Leuk	SNHG30
SEPTIN1	0.008319078	0.227411802	0.537	0.267	1	Leuk	SEPTIN1
UBA2	0.008396617	0.122831364	0.217	0.044	1	Leuk	UBA2
CCDC12	0.008400022	0.165906145	0.288	0.089	1	Leuk	CCDC12
CWC27	0.008411345	0.172560271	0.173	0.022	1	Leuk	CWC27
MRPL41	0.008480274	0.161386191	0.173	0.022	1	Leuk	MRPL41
MLX	0.008490437	0.149738129	0.134	0	1	Leuk	MLX
RNF187	0.008545786	0.145020855	0.289	0.089	1	Leuk	RNF187
RRP7A	0.008552819	0.14572715	0.175	0.022	1	Leuk	RRP7A
POLR2G	0.008558131	0.132996496	0.409	0.156	1	Leuk	POLR2G
APEX1	0.00855984	0.160627802	0.622	0.311	1	Leuk	APEX1
POLD2	0.0085878	0.157122019	0.321	0.111	1	Leuk	POLD2
RPS29	0.008599993	0.282634021	0.858	0.6	1	Leuk	RPS29
FAM111A	0.008624086	0.156689533	0.176	0.022	1	Leuk	FAM111A
CENPH	0.008643673	0.123420488	0.134	0	1	Leuk	CENPH
RBBP8	0.008643673	0.130974983	0.134	0	1	Leuk	RBBP8
AP005482.1	0.008643673	0.14234241	0.134	0	1	Leuk	AP005482.1
GMDS	0.008660954	0.161475814	0.174	0.022	1	Leuk	GMDS
SLIRP	0.008665528	0.113851219	0.217	0.044	1	Leuk	SLIRP
TMSB4X	0.008766773	0.260289981	0.987	0.933	1	Leuk	TMSB4X
UQCC2	0.008775562	0.119182392	0.176	0.022	1	Leuk	UQCC2
VHL	0.008799571	0.149961526	0.133	0	1	Leuk	VHL
RPL22L1	0.008815625	0.225876772	0.519	0.244	1	Leuk	RPL22L1
THOC7	0.008892734	0.15798	0.211	0.044	1	Leuk	THOC7
COX7B	0.008922547	0.143346903	0.453	0.2	1	Leuk	COX7B
CCM2	0.00898367	0.159804067	0.285	0.089	1	Leuk	CCM2
SYF2	0.009014508	0.143807881	0.426	0.178	1	Leuk	SYF2
SAE1	0.009021742	0.118442096	0.175	0.022	1	Leuk	SAE1
BLMH	0.009038508	0.131338829	0.133	0	1	Leuk	BLMH
PSMB7	0.009054308	0.181060898	0.428	0.178	1	Leuk	PSMB7
CCDC124	0.009058379	0.149790735	0.321	0.111	1	Leuk	CCDC124
AKT2	0.009122987	0.166858678	0.173	0.022	1	Leuk	AKT2
TRMT1	0.009137965	0.166536243	0.172	0.022	1	Leuk	TRMT1
RBX1	0.009151512	0.133117524	0.288	0.089	1	Leuk	RBX1
FYB1	0.009157341	0.142266645	0.328	0.111	1	Leuk	FYB1
PARL	0.009167697	0.123437737	0.175	0.022	1	Leuk	PARL
MRPL52	0.009176287	0.144118992	0.249	0.067	1	Leuk	MRPL52
ZBTB44	0.009201259	0.162462375	0.132	0	1	Leuk	ZBTB44
PTPA	0.00920126	0.144036535	0.132	0	1	Leuk	PTPA
ZNF431	0.009283691	0.183670028	0.132	0	1	Leuk	ZNF431
DNAJC15	0.009283691	0.135142618	0.132	0	1	Leuk	DNAJC15
ITM2A	0.009307398	0.190686026	0.417	0.178	1	Leuk	ITM2A
POLD4	0.009375507	0.118084712	0.29	0.089	1	Leuk	POLD4
WDR18	0.009385054	0.100356206	0.211	0.044	1	Leuk	WDR18

KPNB1	0.00940649	0.160071695	0.359	0.133	1	Leuk	KPNB1
SAC3D1	0.009450693	0.13019068	0.131	0	1	Leuk	SAC3D1
MCM4	0.009450693	0.145645137	0.131	0	1	Leuk	MCM4
STX10	0.009506759	0.146625027	0.285	0.089	1	Leuk	STX10
DEK	0.00952905	0.191189214	0.281	0.089	1	Leuk	DEK
YY1AP1	0.009535277	0.172961546	0.131	0	1	Leuk	YY1AP1
SLBP	0.009551984	0.152396086	0.172	0.022	1	Leuk	SLBP
MIF4GD	0.00962059	0.143209608	0.131	0	1	Leuk	MIF4GD
CDKN2AIPNL	0.009749955	0.141468121	0.21	0.044	1	Leuk	CDKN2AIPNL
BZW2	0.00986033	0.107569032	0.252	0.067	1	Leuk	BZW2
NDUFB4	0.009908858	0.12625292	0.504	0.222	1	Leuk	NDUFB4
WAPL	0.00992117	0.177727633	0.168	0.022	1	Leuk	WAPL
RPS20	0.009921505	0.240312117	0.815	0.556	1	Leuk	RPS20
PITHD1	0.009925736	0.148826582	0.245	0.067	1	Leuk	PITHD1
UBA6	0.009969261	0.158009821	0.13	0	1	Leuk	UBA6
