# Supplementary material for: High CD44 expression and enhanced E-selectin binding identified as biomarkers of chemoresistant leukemic cells in human T-ALL
Source: Leukemia. 2024 Nov 24;39(2):323–36. doi: 10.1038/s41375-024-02473-7 (PMC11794132; doi:10.1038/s41375-024-02473-7)
Supplement: Supplementary file 8 — Supplemental Table 7 [file 41375_2024_2473_MOESM8_ESM.pdf]

upregulated genes in Ki67neg CD44high leukemic cells from Library 3 (M187 & M187R) (Supplementary Figure 10j)

	p_val	avg_log2FC	pct.1	pct.2	p_val_adj	cluster	gene
CD44	1.36E-136	1.571953806	1	0.196	4.96E-132	CD44 > 1 & MKI67 < 1 Leuk	CD44
B2M	1.26E-19	0.619832336	1	0.999	4.61E-15	CD44 > 1 & MKI67 < 1 Leuk	B2M
LTB	1.52E-16	0.962271287	0.933	0.801	5.58E-12	CD44 > 1 & MKI67 < 1 Leuk	LTB
FCGRT	5.13E-15	0.614411463	0.45	0.185	1.88E-10	CD44 > 1 & MKI67 < 1 Leuk	FCGRT
MALAT1	1.04E-14	0.692023392	1	1	3.82E-10	CD44 > 1 & MKI67 < 1 Leuk	MALAT1
HLA-B	3.79E-13	0.565153208	0.992	0.971	1.39E-08	CD44 > 1 & MKI67 < 1 Leuk	HLA-B
CD52	4.64E-12	0.779083554	0.825	0.718	1.70E-07	CD44 > 1 & MKI67 < 1 Leuk	CD52
SH3BP5	1.31E-11	0.728910961	0.508	0.273	4.79E-07	CD44 > 1 & MKI67 < 1 Leuk	SH3BP5
CLIC3	1.21E-10	0.64227319	0.417	0.192	4.42E-06	CD44 > 1 & MKI67 < 1 Leuk	CLIC3
HLA-C	1.22E-10	0.567498722	0.917	0.93	4.47E-06	CD44 > 1 & MKI67 < 1 Leuk	HLA-C
PI16	1.05E-08	0.619135152	0.45	0.247	0.000385305	CD44 > 1 & MKI67 < 1 Leuk	PI16
CD3E	1.17E-08	0.421168956	0.925	0.94	0.000426772	CD44 > 1 & MKI67 < 1 Leuk	CD3E
KLRB1	2.02E-08	0.72517419	0.725	0.625	0.000741001	CD44 > 1 & MKI67 < 1 Leuk	KLRB1
LIMD2	2.28E-08	0.477077985	0.858	0.862	0.000835967	CD44 > 1 & MKI67 < 1 Leuk	LIMD2
SH3BGRL3	2.81E-08	0.472904702	0.967	0.975	0.001029485	CD44 > 1 & MKI67 < 1 Leuk	SH3BGRL3
EMP3	3.99E-08	0.583557501	0.758	0.7	0.001460094	CD44 > 1 & MKI67 < 1 Leuk	EMP3
TXNIP	4.05E-08	0.521317975	0.75	0.716	0.00148215	CD44 > 1 & MKI67 < 1 Leuk	TXNIP
PTPRC	2.04E-07	0.492908139	0.917	0.909	0.007450543	CD44 > 1 & MKI67 < 1 Leuk	PTPRC
THEMIS	2.47E-07	0.281760882	0.183	0.064	0.009053519	CD44 > 1 & MKI67 < 1 Leuk	THEMIS
RETN	2.53E-07	0.598084747	0.525	0.375	0.009252626	CD44 > 1 & MKI67 < 1 Leuk	RETN
IFITM2	1.26E-06	0.558143099	0.767	0.739	0.046247551	CD44 > 1 & MKI67 < 1 Leuk	IFITM2
HLA-A	1.68E-06	0.388396584	0.867	0.901	0.061314822	CD44 > 1 & MKI67 < 1 Leuk	HLA-A
SEPTIN9	3.67E-06	0.412441258	0.692	0.673	0.134368447	CD44 > 1 & MKI67 < 1 Leuk	SEPTIN9
HLA-E	3.84E-06	0.448178675	0.7	0.711	0.14048765	CD44 > 1 & MKI67 < 1 Leuk	HLA-E
TLE5	6.44E-06	0.311728105	0.867	0.885	0.23587684	CD44 > 1 & MKI67 < 1 Leuk	TLE5
FUT7	1.55E-05	0.273393965	0.192	0.082	0.565673696	CD44 > 1 & MKI67 < 1 Leuk	FUT7
TIAM1	2.63E-05	0.213308159	0.142	0.052	0.963079459	CD44 > 1 & MKI67 < 1 Leuk	TIAM1
AQP3	2.92E-05	0.431912266	0.392	0.255	1	CD44 > 1 & MKI67 < 1 Leuk	AQP3
HOMER3	4.39E-05	0.347550706	0.217	0.106	1	CD44 > 1 & MKI67 < 1 Leuk	HOMER3
CD7	5.06E-05	0.396140659	0.883	0.878	1	CD44 > 1 & MKI67 < 1 Leuk	CD7
TMSB10	5.24E-05	0.268694737	0.992	0.997	1	CD44 > 1 & MKI67 < 1 Leuk	TMSB10
CD79A	9.24E-05	0.579802076	0.367	0.246	1	CD44 > 1 & MKI67 < 1 Leuk	CD79A
HCST	0.000125165	0.53546157	0.6	0.565	1	CD44 > 1 & MKI67 < 1 Leuk	HCST
MBP	0.000131081	0.310415517	0.25	0.139	1	CD44 > 1 & MKI67 < 1 Leuk	MBP
DDIT4	0.000146025	0.477178146	0.508	0.424	1	CD44 > 1 & MKI67 < 1 Leuk	DDIT4
SELL	0.000154061	0.364506219	0.792	0.826	1	CD44 > 1 & MKI67 < 1 Leuk	SELL
IER2	0.000154219	0.581289042	0.55	0.513	1	CD44 > 1 & MKI67 < 1 Leuk	IER2
EVL	0.000165248	0.311038081	0.817	0.836	1	CD44 > 1 & MKI67 < 1 Leuk	EVL
ITGA6	0.000187095	0.356121931	0.333	0.22	1	CD44 > 1 & MKI67 < 1 Leuk	ITGA6
RIPOR2	0.00021043	0.481317311	0.417	0.316	1	CD44 > 1 & MKI67 < 1 Leuk	RIPOR2
KLF2	0.000211934	0.421528777	0.442	0.313	1	CD44 > 1 & MKI67 < 1 Leuk	KLF2
ITGB7	0.000245888	0.253130052	0.2	0.104	1	CD44 > 1 & MKI67 < 1 Leuk	ITGB7
ARL4C	0.000335234	0.475573486	0.617	0.614	1	CD44 > 1 & MKI67 < 1 Leuk	ARL4C
STK17B	0.00036059	0.414362889	0.45	0.369	1	CD44 > 1 & MKI67 < 1 Leuk	STK17B
ULK2	0.000607696	0.161742865	0.1	0.038	1	CD44 > 1 & MKI67 < 1 Leuk	ULK2
JUNB	0.000636773	0.46171332	0.308	0.207	1	CD44 > 1 & MKI67 < 1 Leuk	JUNB

PNRC1	0.000670937	0.455307172	0.692	0.73	1	CD44 > 1 & MKI67 < 1 Leuk	PNRC1
SIT1	0.000754793	0.492927205	0.458	0.402	1	CD44 > 1 & MKI67 < 1 Leuk	SIT1
S100A6	0.000816276	0.300844999	0.517	0.438	1	CD44 > 1 & MKI67 < 1 Leuk	S100A6
TBC1D10C	0.000820864	0.368395618	0.492	0.448	1	CD44 > 1 & MKI67 < 1 Leuk	TBC1D10C
TRAF3IP3	0.000866314	0.367337792	0.558	0.533	1	CD44 > 1 & MKI67 < 1 Leuk	TRAF3IP3
ETS1	0.000934933	0.508639406	0.55	0.535	1	CD44 > 1 & MKI67 < 1 Leuk	ETS1
LSP1	0.00120728	0.331680497	0.642	0.635	1	CD44 > 1 & MKI67 < 1 Leuk	LSP1
PPM1M	0.001327401	0.249038255	0.192	0.109	1	CD44 > 1 & MKI67 < 1 Leuk	PPM1M
PARP12	0.001332503	0.19817177	0.117	0.052	1	CD44 > 1 & MKI67 < 1 Leuk	PARP12
IFITM1	0.001678176	0.360152126	0.883	0.906	1	CD44 > 1 & MKI67 < 1 Leuk	IFITM1
LIME1	0.001719191	0.310277396	0.658	0.683	1	CD44 > 1 & MKI67 < 1 Leuk	LIME1
RSRP1	0.001769609	0.458584859	0.5	0.473	1	CD44 > 1 & MKI67 < 1 Leuk	RSRP1
ELOVL4	0.001934727	0.377512755	0.225	0.134	1	CD44 > 1 & MKI67 < 1 Leuk	ELOVL4
TC2N	0.001961684	0.188853785	0.15	0.076	1	CD44 > 1 & MKI67 < 1 Leuk	TC2N
COL6A2	0.002077086	0.174863528	0.1	0.042	1	CD44 > 1 & MKI67 < 1 Leuk	COL6A2
SYNRG	0.002132317	0.371992801	0.325	0.249	1	CD44 > 1 & MKI67 < 1 Leuk	SYNRG
SPOCK2	0.002736487	0.297468606	0.325	0.234	1	CD44 > 1 & MKI67 < 1 Leuk	SPOCK2
LY9	0.002741243	0.191558216	0.158	0.085	1	CD44 > 1 & MKI67 < 1 Leuk	LY9
LPGAT1	0.002885301	0.269094788	0.267	0.185	1	CD44 > 1 & MKI67 < 1 Leuk	LPGAT1
IL7R	0.003059824	0.255775483	0.15	0.078	1	CD44 > 1 & MKI67 < 1 Leuk	IL7R
CDKN2D	0.003363299	0.394044665	0.483	0.436	1	CD44 > 1 & MKI67 < 1 Leuk	CDKN2D
IL32	0.00346976	0.254157953	0.992	0.979	1	CD44 > 1 & MKI67 < 1 Leuk	IL32
TMEM63A	0.003915818	0.165976326	0.108	0.05	1	CD44 > 1 & MKI67 < 1 Leuk	TMEM63A
SGTB	0.004060796	0.126238778	0.1	0.045	1	CD44 > 1 & MKI67 < 1 Leuk	SGTB
PFDN5	0.004181182	0.242548831	0.792	0.864	1	CD44 > 1 & MKI67 < 1 Leuk	PFDN5
MYOM2	0.004321914	0.253587887	0.167	0.094	1	CD44 > 1 & MKI67 < 1 Leuk	MYOM2
EPB41	0.004322247	0.282434056	0.342	0.271	1	CD44 > 1 & MKI67 < 1 Leuk	EPB41
ATM	0.004365566	0.333306327	0.308	0.227	1	CD44 > 1 & MKI67 < 1 Leuk	ATM
NKG7	0.004458366	0.401109902	0.425	0.364	1	CD44 > 1 & MKI67 < 1 Leuk	NKG7
PARP8	0.00457129	0.190034675	0.183	0.11	1	CD44 > 1 & MKI67 < 1 Leuk	PARP8
FXYD7	0.004699278	0.151539185	0.133	0.069	1	CD44 > 1 & MKI67 < 1 Leuk	FXYD7
DDX5	0.004773875	0.280141265	0.867	0.907	1	CD44 > 1 & MKI67 < 1 Leuk	DDX5
RAC2	0.004833452	0.270759596	0.875	0.915	1	CD44 > 1 & MKI67 < 1 Leuk	RAC2
LEF1	0.005636446	0.283305741	0.508	0.477	1	CD44 > 1 & MKI67 < 1 Leuk	LEF1
RAB29	0.005647362	0.159366237	0.1	0.047	1	CD44 > 1 & MKI67 < 1 Leuk	RAB29
RAB37	0.006870862	0.30730294	0.508	0.513	1	CD44 > 1 & MKI67 < 1 Leuk	RAB37
CD2	0.007421252	0.307230184	0.558	0.564	1	CD44 > 1 & MKI67 < 1 Leuk	CD2
SMIM29	0.008931284	0.190691964	0.15	0.088	1	CD44 > 1 & MKI67 < 1 Leuk	SMIM29
ZBTB20	0.00928796	0.319672022	0.233	0.167	1	CD44 > 1 & MKI67 < 1 Leuk	ZBTB20
TCF7	0.009977348	0.248887254	0.767	0.823	1	CD44 > 1 & MKI67 < 1 Leuk	TCF7
GAPDH	6.59E-20	0.9180176	0.955	0.85	2.41E-15	Leuk	GAPDH
RPLP1	2.24E-16	0.585931407	0.991	0.983	8.18E-12	Leuk	RPLP1
RPLP0	5.01E-16	0.608184805	0.974	0.95	1.84E-11	Leuk	RPLP0
MIF	1.04E-14	0.741314181	0.859	0.65	3.80E-10	Leuk	MIF
HSP90AB1	1.23E-14	0.758008847	0.815	0.55	4.51E-10	Leuk	HSP90AB1
RPS2	2.46E-13	0.547813445	0.961	0.892	9.00E-09	Leuk	RPS2
PTMA	9.84E-13	0.480687578	0.989	0.967	3.60E-08	Leuk	PTMA
GSTP1	1.01E-12	0.652052389	0.759	0.417	3.69E-08	Leuk	GSTP1
FTH1	3.26E-12	0.452645185	0.983	0.95	1.19E-07	Leuk	FTH1

TUBA1B	4.79E-12	1.07369808	0.696	0.358	1.75E-07	Leuk	TUBA1B
RPSA	3.72E-11	0.456636639	0.949	0.875	1.36E-06	Leuk	RPSA
MT2A	4.18E-11	0.414016364	0.359	0.05	1.53E-06	Leuk	MT2A
HSPE1	6.30E-11	0.40872195	0.438	0.108	2.31E-06	Leuk	HSPE1
GLUL	9.18E-11	0.63611347	0.73	0.4	3.36E-06	Leuk	GLUL
IGLL1	9.30E-11	0.616683698	0.336	0.042	3.40E-06	Leuk	IGLL1
CALR	2.33E-10	0.535553678	0.658	0.333	8.53E-06	Leuk	CALR
PPP1R14B	3.15E-10	0.440918133	0.61	0.25	1.15E-05	Leuk	PPP1R14B
NPM1	3.70E-10	0.517305749	0.872	0.675	1.35E-05	Leuk	NPM1
TYMS	6.05E-10	0.53711778	0.365	0.083	2.21E-05	Leuk	TYMS
ACTG1	8.77E-10	0.491220031	0.976	0.908	3.21E-05	Leuk	ACTG1
ENO1	9.46E-10	0.560527409	0.765	0.483	3.46E-05	Leuk	ENO1
EEF1B2	1.25E-09	0.477350941	0.898	0.75	4.58E-05	Leuk	EEF1B2
ITM2A	1.49E-09	0.523578882	0.421	0.125	5.44E-05	Leuk	ITM2A
PCLAF	2.56E-09	0.474573545	0.31	0.05	9.38E-05	Leuk	PCLAF
RPL35	2.59E-09	0.459985526	0.921	0.733	9.48E-05	Leuk	RPL35
STMN1	2.73E-09	0.612559173	0.91	0.75	9.98E-05	Leuk	STMN1
PPA1	3.29E-09	0.309898204	0.397	0.1	0.000120368	Leuk	PPA1
HNRNPA1	3.77E-09	0.432319079	0.906	0.742	0.000138028	Leuk	HNRNPA1
HSPD1	5.34E-09	0.441062099	0.507	0.2	0.000195583	Leuk	HSPD1
TUBB	5.38E-09	0.734516922	0.75	0.483	0.000196777	Leuk	TUBB
SNRPB	6.13E-09	0.376764112	0.563	0.225	0.000224518	Leuk	SNRPB
RANBP1	6.34E-09	0.364004547	0.403	0.125	0.000232176	Leuk	RANBP1
FABP5	7.58E-09	0.355157372	0.4	0.108	0.000277438	Leuk	FABP5
ATP5MC1	7.61E-09	0.374785405	0.481	0.175	0.000278432	Leuk	ATP5MC1
CD99	7.90E-09	0.447893996	0.959	0.875	0.000289318	Leuk	CD99
PGD	9.56E-09	0.25133494	0.338	0.067	0.000349996	Leuk	PGD
RTRAF	1.16E-08	0.3722663	0.551	0.233	0.000423368	Leuk	RTRAF
RPS18	1.22E-08	0.353622191	0.984	0.967	0.000445476	Leuk	RPS18
SRSF7	1.84E-08	0.334795067	0.619	0.283	0.000672317	Leuk	SRSF7
MCM7	2.46E-08	0.314938501	0.273	0.033	0.000900577	Leuk	MCM7
NME1	2.48E-08	0.378616689	0.455	0.167	0.00090777	Leuk	NME1
LYL1	2.49E-08	0.264552356	0.225	0.008	0.000910439	Leuk	LYL1
RPA3	2.82E-08	0.234479385	0.271	0.033	0.001032475	Leuk	RPA3
RPS17	3.37E-08	0.374737633	0.709	0.4	0.00123217	Leuk	RPS17
BAZ1B	3.91E-08	0.284548797	0.333	0.075	0.001431215	Leuk	BAZ1B
HINT1	4.40E-08	0.381449864	0.877	0.633	0.001610958	Leuk	HINT1
COX5A	4.54E-08	0.33963804	0.61	0.283	0.001662108	Leuk	COX5A
RPL7A	5.66E-08	0.341295017	0.981	0.917	0.002070728	Leuk	RPL7A
ATP5F1B	6.19E-08	0.393427829	0.728	0.408	0.002264684	Leuk	ATP5F1B
GNA15	7.09E-08	0.379213326	0.505	0.208	0.002596341	Leuk	GNA15
SEM1	8.45E-08	0.25299184	0.451	0.15	0.003091564	Leuk	SEM1
PPIA	8.75E-08	0.381162147	0.945	0.875	0.00320265	Leuk	PPIA
ATP5PF	1.03E-07	0.335722352	0.602	0.283	0.003778911	Leuk	ATP5PF
MRPL51	1.04E-07	0.264596287	0.44	0.158	0.003814257	Leuk	MRPL51
MDH1	1.15E-07	0.278388985	0.44	0.15	0.0042205	Leuk	MDH1
RPL10A	1.16E-07	0.38640117	0.963	0.867	0.004246708	Leuk	RPL10A
CDK6	1.28E-07	0.303777581	0.765	0.483	0.00470039	Leuk	CDK6
H2AFZ	1.34E-07	0.629766843	0.702	0.433	0.004921871	Leuk	H2AFZ

ACTB	1.85E-07	0.339149825	0.995	1	0.006760486	Leuk	ACTB
SRM	1.91E-07	0.210397048	0.352	0.092	0.006990842	Leuk	SRM
RPS3	2.15E-07	0.294840093	0.985	0.975	0.0078643	Leuk	RPS3
RPS11	3.38E-07	0.366580989	0.863	0.608	0.012378566	Leuk	RPS11
PA2G4	3.47E-07	0.337121114	0.496	0.225	0.012710243	Leuk	PA2G4
WDR18	3.65E-07	0.212488681	0.212	0.017	0.013346648	Leuk	WDR18
DCTPP1	4.00E-07	0.238543338	0.274	0.058	0.014648336	Leuk	DCTPP1
PEBP1	4.02E-07	0.245657222	0.479	0.183	0.014710123	Leuk	PEBP1
CCT3	4.75E-07	0.262742341	0.451	0.175	0.017378452	Leuk	CCT3
FDPS	5.01E-07	0.255243523	0.355	0.108	0.018348238	Leuk	FDPS
CCT6A	5.05E-07	0.276164015	0.476	0.2	0.018489559	Leuk	CCT6A
RNF130	5.74E-07	0.226894141	0.496	0.2	0.020994104	Leuk	RNF130
HSPA5	7.43E-07	0.296306148	0.398	0.142	0.027203465	Leuk	HSPA5
MCM5	7.51E-07	0.244987468	0.255	0.05	0.027473184	Leuk	MCM5
VDAC1	8.78E-07	0.22075435	0.379	0.125	0.032136001	Leuk	VDAC1
TOMM6	9.36E-07	0.283219302	0.52	0.233	0.034243182	Leuk	TOMM6
CFL1	9.88E-07	0.316978884	0.958	0.892	0.036158279	Leuk	CFL1
NHP2	1.03E-06	0.248146483	0.467	0.192	0.037775356	Leuk	NHP2
TSPAN7	1.06E-06	0.308935376	0.569	0.283	0.038728275	Leuk	TSPAN7
RBM3	1.06E-06	0.262175338	0.576	0.267	0.03883043	Leuk	RBM3
PHB	1.09E-06	0.226995501	0.425	0.167	0.039744581	Leuk	PHB
TESC	1.16E-06	0.214598389	0.24	0.042	0.042491375	Leuk	TESC
LMNB1	1.18E-06	0.237474323	0.29	0.075	0.04309899	Leuk	LMNB1
SLC25A3	1.18E-06	0.265972759	0.661	0.35	0.043307518	Leuk	SLC25A3
SNRPG	1.20E-06	0.193803662	0.418	0.15	0.043914557	Leuk	SNRPG
GNG5	1.27E-06	0.257597812	0.644	0.317	0.046576485	Leuk	GNG5
JPT1	1.52E-06	0.379533278	0.719	0.442	0.055650656	Leuk	JPT1
EIF3B	1.62E-06	0.236801808	0.317	0.092	0.059146462	Leuk	EIF3B
TBCA	1.70E-06	0.226727748	0.488	0.208	0.062066818	Leuk	TBCA
C12orf57	1.72E-06	0.307045572	0.802	0.492	0.063122005	Leuk	C12orf57
TXN	1.94E-06	0.394578708	0.71	0.458	0.070846727	Leuk	TXN
IMPDH2	1.95E-06	0.241766818	0.388	0.142	0.071495286	Leuk	IMPDH2
DNMT1	2.03E-06	0.239868268	0.351	0.117	0.074243756	Leuk	DNMT1
MCM3	2.05E-06	0.225863675	0.263	0.058	0.075079493	Leuk	MCM3
NOP56	2.11E-06	0.166227736	0.306	0.083	0.077409595	Leuk	NOP56
NUDC	2.21E-06	0.245677309	0.41	0.158	0.080733972	Leuk	NUDC
LDHA	2.46E-06	0.304253517	0.711	0.433	0.090170019	Leuk	LDHA
HMGN2	2.47E-06	0.573859259	0.8	0.583	0.090243933	Leuk	HMGN2
CDT1	2.48E-06	0.192125546	0.203	0.025	0.090787812	Leuk	CDT1
HNRNPAB	2.55E-06	0.244246621	0.414	0.158	0.093213584	Leuk	HNRNPAB
NENF	2.56E-06	0.172375244	0.249	0.05	0.093682602	Leuk	NENF
AKR1B1	2.75E-06	0.157479852	0.291	0.075	0.100518277	Leuk	AKR1B1
AHCY	2.80E-06	0.183089104	0.244	0.05	0.102577106	Leuk	AHCY
IGFBP2	2.93E-06	0.181519951	0.173	0.008	0.107417655	Leuk	IGFBP2
TCERG1	2.95E-06	0.210301649	0.301	0.083	0.108030818	Leuk	TCERG1
ENSA	3.00E-06	0.216183503	0.43	0.167	0.109724014	Leuk	ENSA
ZNRD1	3.10E-06	0.181208547	0.402	0.15	0.113464059	Leuk	ZNRD1
POLR2E	3.10E-06	0.206099572	0.409	0.15	0.113537902	Leuk	POLR2E
PAFAH1B3	3.18E-06	0.20459729	0.377	0.133	0.11646919	Leuk	PAFAH1B3

TMPO	3.21E-06	0.251238158	0.418	0.167	0.117390354	Leuk	TMPO
PSMA4	3.22E-06	0.186862144	0.502	0.217	0.117846597	Leuk	PSMA4
PAICS	3.29E-06	0.206958176	0.296	0.083	0.120301541	Leuk	PAICS
SNRPD1	3.29E-06	0.216403641	0.367	0.133	0.120353461	Leuk	SNRPD1
GTF3C6	3.56E-06	0.197607611	0.319	0.1	0.130158461	Leuk	GTF3C6
RPL27	3.87E-06	0.297751765	0.886	0.667	0.141727736	Leuk	RPL27
RPL8	3.91E-06	0.292399526	0.974	0.883	0.143290211	Leuk	RPL8
NCBP2AS2	4.11E-06	0.183119207	0.242	0.05	0.150564555	Leuk	NCBP2AS2
SNX5	4.36E-06	0.177520096	0.196	0.025	0.159589312	Leuk	SNX5
RUVBL2	4.98E-06	0.183417212	0.267	0.067	0.182363407	Leuk	RUVBL2
CCDC26	5.29E-06	0.215556695	0.333	0.117	0.193633594	Leuk	CCDC26
RTF1	5.40E-06	0.20826254	0.31	0.1	0.1977558	Leuk	RTF1
CLNS1A	5.81E-06	0.158508015	0.373	0.133	0.212492241	Leuk	CLNS1A
CCT5	5.95E-06	0.160904865	0.391	0.15	0.217621689	Leuk	CCT5
GIHCG	5.99E-06	0.287964835	0.604	0.308	0.219344417	Leuk	GIHCG
NDUFA4	6.40E-06	0.307187686	0.71	0.433	0.234354732	Leuk	NDUFA4
ZCRB1	6.68E-06	0.175244232	0.248	0.058	0.244658156	Leuk	ZCRB1
HSBP1	6.82E-06	0.144173954	0.295	0.083	0.249625869	Leuk	HSBP1
EBNA1BP2	6.85E-06	0.172107985	0.193	0.025	0.250803752	Leuk	EBNA1BP2
BANF1	7.49E-06	0.218040922	0.549	0.258	0.274272896	Leuk	BANF1
AC000065.1	7.56E-06	0.214777272	0.219	0.042	0.27655259	Leuk	AC000065.1
PPIH	7.70E-06	0.152230524	0.206	0.033	0.281677025	Leuk	PPIH
HNRNPM	7.84E-06	0.237972162	0.542	0.258	0.286951666	Leuk	HNRNPM
TOP1	8.26E-06	0.17050102	0.444	0.183	0.302265987	Leuk	TOP1
UQCRQ	8.43E-06	0.197507342	0.506	0.225	0.308665872	Leuk	UQCRQ
MRPL3	8.71E-06	0.153619079	0.25	0.058	0.318912471	Leuk	MRPL3
CDK4	8.80E-06	0.196939751	0.27	0.075	0.322043942	Leuk	CDK4
C19orf48	8.99E-06	0.162564289	0.249	0.058	0.329104173	Leuk	C19orf48
RPL3	9.14E-06	0.276471076	0.974	0.933	0.334713908	Leuk	RPL3
WDR61	9.24E-06	0.172758161	0.204	0.033	0.338313166	Leuk	WDR61
SSRP1	9.66E-06	0.194481209	0.311	0.1	0.353515396	Leuk	SSRP1
ATP5PO	9.74E-06	0.242883724	0.619	0.35	0.356455121	Leuk	ATP5PO
MDH2	9.90E-06	0.226152607	0.504	0.242	0.362252018	Leuk	MDH2
SH3GLB1	1.03E-05	0.193325841	0.273	0.075	0.3752854	Leuk	SH3GLB1
CLEC11A	1.03E-05	0.164869209	0.432	0.175	0.376706132	Leuk	CLEC11A
EIF4E	1.14E-05	0.155960707	0.215	0.042	0.41723494	Leuk	EIF4E
HMGB1	1.15E-05	0.473989587	0.92	0.808	0.420286331	Leuk	HMGB1
TMEM14C	1.16E-05	0.138013336	0.328	0.108	0.424749794	Leuk	TMEM14C
CTHRC1	1.16E-05	0.256626727	0.431	0.192	0.42501347	Leuk	CTHRC1
SNRPF	1.16E-05	0.16985386	0.428	0.183	0.426035081	Leuk	SNRPF
RPL15	1.29E-05	0.250138622	0.958	0.925	0.471645126	Leuk	RPL15
PRDX6	1.29E-05	0.20679117	0.494	0.225	0.471803277	Leuk	PRDX6
PDIA6	1.31E-05	0.170582696	0.359	0.133	0.480027273	Leuk	PDIA6
U2AF2	1.33E-05	0.166103045	0.249	0.067	0.485346245	Leuk	U2AF2
DNAJB6	1.33E-05	0.168335062	0.361	0.133	0.487465481	Leuk	DNAJB6
SNHG6	1.37E-05	0.21123857	0.623	0.325	0.502004019	Leuk	SNHG6
SOD1	1.46E-05	0.208954947	0.594	0.308	0.535630018	Leuk	SOD1
SRI	1.47E-05	0.181618774	0.436	0.192	0.538531526	Leuk	SRI
NDUFS6	1.48E-05	0.182909946	0.468	0.217	0.540796259	Leuk	NDUFS6

PSMA6	1.48E-05	0.257250509	0.604	0.308	0.542312521	Leuk	PSMA6
CACYBP	1.50E-05	0.175139223	0.341	0.125	0.548504197	Leuk	CACYBP
SNRPA	1.51E-05	0.180922216	0.397	0.158	0.553325775	Leuk	SNRPA
EPRS	1.53E-05	0.121695927	0.293	0.092	0.559925913	Leuk	EPRS
PRKDC	1.56E-05	0.178151537	0.319	0.108	0.57160061	Leuk	PRKDC
SDHB	1.59E-05	0.166867437	0.226	0.05	0.580361694	Leuk	SDHB
RHEX	1.71E-05	0.181242411	0.135	0	0.624727067	Leuk	RHEX
AC002454.1	1.73E-05	0.197319395	0.181	0.025	0.631505853	Leuk	AC002454.1
RPL28	1.73E-05	0.203328736	0.994	0.983	0.634389067	Leuk	RPL28
WDR34	1.74E-05	0.168450439	0.164	0.017	0.637060202	Leuk	WDR34
POLR2I	1.77E-05	0.132687836	0.305	0.1	0.646718159	Leuk	POLR2I
SND1	1.84E-05	0.163674084	0.25	0.067	0.672665273	Leuk	SND1
MRPL20	1.88E-05	0.156537079	0.333	0.117	0.687748633	Leuk	MRPL20
USP1	1.90E-05	0.163359632	0.29	0.092	0.69487529	Leuk	USP1
HMGB2	1.92E-05	0.489831995	0.485	0.258	0.703034071	Leuk	HMGB2
SEC13	1.92E-05	0.17597947	0.33	0.117	0.704446688	Leuk	SEC13
MCM6	2.01E-05	0.157978902	0.195	0.033	0.736526066	Leuk	MCM6
POLDIP2	2.04E-05	0.156052508	0.165	0.017	0.747060036	Leuk	POLDIP2
VDAC3	2.08E-05	0.162899784	0.331	0.117	0.759982527	Leuk	VDAC3
RPS6	2.11E-05	0.300103266	0.946	0.842	0.773197312	Leuk	RPS6
CKS2	2.14E-05	0.171356337	0.194	0.033	0.78416447	Leuk	CKS2
SIVA1	2.15E-05	0.259262072	0.53	0.283	0.788159246	Leuk	SIVA1
NIFK	2.20E-05	0.160665478	0.298	0.1	0.806373423	Leuk	NIFK
DAZAP1	2.20E-05	0.215227451	0.36	0.15	0.80696787	Leuk	DAZAP1
NASP	2.28E-05	0.275896545	0.473	0.233	0.835684808	Leuk	NASP
CCT4	2.31E-05	0.190243915	0.425	0.192	0.846143028	Leuk	CCT4
GTF3A	2.32E-05	0.201987454	0.729	0.433	0.849880498	Leuk	GTF3A
PRDX1	2.34E-05	0.211124909	0.583	0.308	0.857779093	Leuk	PRDX1
COPS6	2.38E-05	0.153751768	0.346	0.125	0.870729266	Leuk	COPS6
MAD2L1	2.39E-05	0.143056746	0.163	0.017	0.874675173	Leuk	MAD2L1
DYNLL1	2.40E-05	0.223609264	0.635	0.358	0.877223218	Leuk	DYNLL1
AKR1C3	2.42E-05	0.175790637	0.324	0.117	0.885716121	Leuk	AKR1C3
RAN	2.50E-05	0.331314994	0.68	0.458	0.914997127	Leuk	RAN
POLE4	2.55E-05	0.128084017	0.177	0.025	0.934538099	Leuk	POLE4
PSMA3	2.58E-05	0.192965696	0.28	0.092	0.945027745	Leuk	PSMA3
TSHR	2.62E-05	0.212861415	0.326	0.117	0.959754733	Leuk	TSHR
MRPL15	2.63E-05	0.13189981	0.146	0.008	0.961728675	Leuk	MRPL15
PRELID1	2.66E-05	0.136687214	0.438	0.192	0.973538	Leuk	PRELID1
FKBP1A	2.75E-05	0.193088543	0.537	0.267	1	Leuk	FKBP1A
MRPL22	2.89E-05	0.123401003	0.192	0.033	1	Leuk	MRPL22
LSM5	2.94E-05	0.180128082	0.334	0.125	1	Leuk	LSM5
NUCKS1	2.98E-05	0.287261438	0.523	0.275	1	Leuk	NUCKS1
PXMP2	3.00E-05	0.129269233	0.176	0.025	1	Leuk	PXMP2
RPL22	3.04E-05	0.275908577	0.928	0.775	1	Leuk	RPL22
PSMB1	3.05E-05	0.161714794	0.569	0.283	1	Leuk	PSMB1
UQCC2	3.07E-05	0.139760568	0.201	0.042	1	Leuk	UQCC2
RPN1	3.10E-05	0.142111764	0.317	0.108	1	Leuk	RPN1
SMARCC1	3.34E-05	0.149300355	0.453	0.208	1	Leuk	SMARCC1
LSM4	3.38E-05	0.195237241	0.436	0.192	1	Leuk	LSM4

MRPS7	3.38E-05	0.165097406	0.324	0.117	1	Leuk	MRPS7
PRDX2	3.44E-05	0.168589224	0.382	0.158	1	Leuk	PRDX2
RPL36A	3.45E-05	0.261348374	0.91	0.742	1	Leuk	RPL36A
RPS12	3.45E-05	0.239875827	0.988	0.975	1	Leuk	RPS12
CBX3	3.46E-05	0.220280441	0.568	0.292	1	Leuk	CBX3
PFDN2	3.48E-05	0.102177543	0.289	0.092	1	Leuk	PFDN2
PSMB3	3.50E-05	0.164315281	0.552	0.258	1	Leuk	PSMB3
PSMA2	3.50E-05	0.160542987	0.508	0.242	1	Leuk	PSMA2
NDUFS8	3.54E-05	0.186306664	0.463	0.217	1	Leuk	NDUFS8
RFC2	3.57E-05	0.151550728	0.157	0.017	1	Leuk	RFC2
NUDT21	3.69E-05	0.147688639	0.256	0.075	1	Leuk	NUDT21
RPL27A	3.71E-05	0.266659159	0.774	0.5	1	Leuk	RPL27A
PSME2	3.71E-05	0.234465212	0.621	0.342	1	Leuk	PSME2
HSP90B1	3.72E-05	0.323744375	0.484	0.258	1	Leuk	HSP90B1
RPS8	3.74E-05	0.230337706	0.993	0.975	1	Leuk	RPS8
AKR1C1	3.77E-05	0.156686352	0.27	0.083	1	Leuk	AKR1C1
SRSF3	3.81E-05	0.266460134	0.682	0.417	1	Leuk	SRSF3
HINT2	3.81E-05	0.141015845	0.268	0.083	1	Leuk	HINT2
ECHS1	3.85E-05	0.148202475	0.243	0.067	1	Leuk	ECHS1
NDUFB10	3.90E-05	0.158851066	0.491	0.225	1	Leuk	NDUFB10
SUMO3	3.97E-05	0.154278095	0.347	0.133	1	Leuk	SUMO3
SERF2	4.00E-05	0.199860952	0.961	0.9	1	Leuk	SERF2
ADRM1	4.07E-05	0.170256701	0.421	0.192	1	Leuk	ADRM1
FKBP3	4.10E-05	0.11760383	0.327	0.117	1	Leuk	FKBP3
RPS5	4.13E-05	0.254810902	0.96	0.925	1	Leuk	RPS5
TFDP2	4.21E-05	0.169059995	0.539	0.258	1	Leuk	TFDP2
YIF1B	4.33E-05	0.12839391	0.21	0.05	1	Leuk	YIF1B
MAD2L2	4.37E-05	0.190208343	0.312	0.117	1	Leuk	MAD2L2
TM7SF3	4.41E-05	0.132848136	0.254	0.075	1	Leuk	TM7SF3
HPRT1	4.52E-05	0.144373772	0.261	0.083	1	Leuk	HPRT1
DDAH2	4.52E-05	0.18490891	0.425	0.192	1	Leuk	DDAH2
UBE2S	4.53E-05	0.197595278	0.345	0.142	1	Leuk	UBE2S
PRCC	4.60E-05	0.157199124	0.212	0.05	1	Leuk	PRCC
TRA2B	4.61E-05	0.186757183	0.472	0.225	1	Leuk	TRA2B
QPRT	4.66E-05	0.154381376	0.151	0.017	1	Leuk	QPRT
CCDC85B	4.80E-05	0.151536469	0.391	0.167	1	Leuk	CCDC85B
CCDC47	4.96E-05	0.165411304	0.207	0.05	1	Leuk	CCDC47
GART	5.17E-05	0.119669088	0.169	0.025	1	Leuk	GART
ATG3	5.19E-05	0.109247426	0.244	0.067	1	Leuk	ATG3
TPO	5.24E-05	0.182965098	0.286	0.1	1	Leuk	TPO
VEGFB	5.27E-05	0.150609182	0.264	0.083	1	Leuk	VEGFB
DOCK8	5.31E-05	0.156074538	0.238	0.067	1	Leuk	DOCK8
EIF2A	5.38E-05	0.147264211	0.209	0.05	1	Leuk	EIF2A
RALBP1	5.40E-05	0.176923136	0.419	0.183	1	Leuk	RALBP1
YWHAE	5.41E-05	0.16047507	0.3	0.108	1	Leuk	YWHAE
OCIAD2	5.51E-05	0.183814028	0.508	0.25	1	Leuk	OCIAD2
HELLS	5.56E-05	0.147273219	0.182	0.033	1	Leuk	HELLS
DUT	5.60E-05	0.279601334	0.496	0.258	1	Leuk	DUT
NUDT3	5.72E-05	0.164151218	0.174	0.033	1	Leuk	NUDT3

YWHAH	5.76E-05	0.132908542	0.362	0.142	1	Leuk	YWHAH
POLD2	5.80E-05	0.135461464	0.196	0.042	1	Leuk	POLD2
URM1	5.88E-05	0.149132428	0.248	0.075	1	Leuk	URM1
MRPS34	5.88E-05	0.142261218	0.359	0.142	1	Leuk	MRPS34
SNU13	6.22E-05	0.178714846	0.516	0.258	1	Leuk	SNU13
ANAPC11	6.23E-05	0.217470466	0.591	0.333	1	Leuk	ANAPC11
USP10	6.34E-05	0.13965426	0.192	0.042	1	Leuk	USP10
SMDT1	6.47E-05	0.17000843	0.584	0.308	1	Leuk	SMDT1
PFDN6	6.53E-05	0.150063285	0.192	0.042	1	Leuk	PFDN6
SAP18	6.63E-05	0.19125383	0.623	0.325	1	Leuk	SAP18
PTTG1	6.75E-05	0.194224151	0.193	0.042	1	Leuk	PTTG1
ENY2	6.85E-05	0.14341513	0.319	0.125	1	Leuk	ENY2
SMC3	7.11E-05	0.136322993	0.398	0.175	1	Leuk	SMC3
PMVK	7.14E-05	0.123011638	0.134	0.008	1	Leuk	PMVK
DTYMK	7.18E-05	0.125637941	0.179	0.033	1	Leuk	DTYMK
RPL21	7.25E-05	0.248278092	0.941	0.842	1	Leuk	RPL21
C1orf35	7.33E-05	0.108043163	0.149	0.017	1	Leuk	C1orf35
AEBP1	7.33E-05	0.284317356	0.661	0.408	1	Leuk	AEBP1
MRPL12	7.46E-05	0.146621365	0.255	0.083	1	Leuk	MRPL12
MRPS15	7.47E-05	0.143648737	0.299	0.108	1	Leuk	MRPS15
HNRNPA3	7.60E-05	0.251795223	0.666	0.408	1	Leuk	HNRNPA3
TMEM106C	7.66E-05	0.112869751	0.193	0.042	1	Leuk	TMEM106C
SEC61G	7.80E-05	0.138270119	0.512	0.242	1	Leuk	SEC61G
MTCH2	7.89E-05	0.108729329	0.233	0.067	1	Leuk	MTCH2
EIF6	7.93E-05	0.1236804	0.367	0.15	1	Leuk	EIF6
CCT2	8.00E-05	0.167693465	0.548	0.275	1	Leuk	CCT2
SET	8.00E-05	0.202052397	0.709	0.433	1	Leuk	SET
CENPX	8.02E-05	0.169170303	0.252	0.083	1	Leuk	CENPX
SNHG8	8.04E-05	0.229445507	0.527	0.283	1	Leuk	SNHG8
PTPN6	8.07E-05	0.18915233	0.544	0.283	1	Leuk	PTPN6
RPS21	8.09E-05	0.260567438	0.945	0.842	1	Leuk	RPS21
STAT5A	8.20E-05	0.173849587	0.382	0.167	1	Leuk	STAT5A
TSPAN5	8.31E-05	0.141341596	0.132	0.008	1	Leuk	TSPAN5
MT-ND5	8.32E-05	0.234961459	0.898	0.767	1	Leuk	MT-ND5
ZWINT	8.39E-05	0.129200912	0.131	0.008	1	Leuk	ZWINT
TPI1	8.65E-05	0.332262699	0.616	0.4	1	Leuk	TPI1
ELOVL5	8.73E-05	0.13567442	0.437	0.208	1	Leuk	ELOVL5
NDUFB6	8.80E-05	0.11702482	0.39	0.167	1	Leuk	NDUFB6
VPS72	8.86E-05	0.107134849	0.177	0.033	1	Leuk	VPS72
RRP7A	8.90E-05	0.137478955	0.175	0.033	1	Leuk	RRP7A
MGST3	8.99E-05	0.149765711	0.214	0.058	1	Leuk	MGST3
MRPS16	9.15E-05	0.144051339	0.217	0.058	1	Leuk	MRPS16
GINS2	9.15E-05	0.141846658	0.144	0.017	1	Leuk	GINS2
TIMM13	9.44E-05	0.178328239	0.403	0.183	1	Leuk	TIMM13
C1orf43	9.46E-05	0.116326948	0.29	0.1	1	Leuk	C1orf43
HAX1	9.75E-05	0.109402466	0.23	0.067	1	Leuk	HAX1
POMP	9.85E-05	0.171363298	0.525	0.267	1	Leuk	POMP
GNB1	9.95E-05	0.17276608	0.381	0.167	1	Leuk	GNB1
STIP1	0.000100688	0.122688972	0.254	0.083	1	Leuk	STIP1

HNRNPA0	0.000102263	0.210247081	0.673	0.408	1	Leuk	HNRNPA0
GPATCH4	0.000102476	0.123808177	0.145	0.017	1	Leuk	GPATCH4
RPS20	0.000103177	0.222905213	0.705	0.442	1	Leuk	RPS20
NDUFV2	0.000103257	0.137793155	0.419	0.183	1	Leuk	NDUFV2
NOP58	0.000105081	0.159134832	0.339	0.142	1	Leuk	NOP58
HSPB11	0.000106179	0.147662601	0.28	0.1	1	Leuk	HSPB11
SOD2	0.000106826	0.108524932	0.16	0.025	1	Leuk	SOD2
PREB	0.000107985	0.111581443	0.129	0.008	1	Leuk	PREB
TCHP	0.000109312	0.143603255	0.141	0.017	1	Leuk	TCHP
EIF2S1	0.000111073	0.136328601	0.2	0.05	1	Leuk	EIF2S1
ATP5MC2	0.000112305	0.267032729	0.891	0.75	1	Leuk	ATP5MC2
HEBP2	0.000113236	0.13117652	0.324	0.133	1	Leuk	HEBP2
HADH	0.000113745	0.120459627	0.199	0.05	1	Leuk	HADH
EWSR1	0.000114222	0.178450034	0.603	0.325	1	Leuk	EWSR1
EXOSC9	0.000116019	0.112454608	0.173	0.033	1	Leuk	EXOSC9
ILF2	0.000117026	0.172303422	0.524	0.267	1	Leuk	ILF2
MZT1	0.000117174	0.133966951	0.156	0.025	1	Leuk	MZT1
SSNA1	0.000117548	0.152119229	0.367	0.158	1	Leuk	SSNA1
PTGES3	0.000117948	0.241768581	0.654	0.417	1	Leuk	PTGES3
CDCA7L	0.000118637	0.164267211	0.274	0.1	1	Leuk	CDCA7L
EEF1E1	0.000119045	0.101115342	0.173	0.033	1	Leuk	EEF1E1
METTL5	0.000119047	0.124429212	0.212	0.058	1	Leuk	METTL5
MPP1	0.000120841	0.147893958	0.271	0.1	1	Leuk	MPP1
PHIP	0.000121065	0.171324448	0.324	0.133	1	Leuk	PHIP
CCDC88A	0.000121288	0.145016196	0.291	0.108	1	Leuk	CCDC88A
POLR2L	0.000121337	0.147071111	0.513	0.267	1	Leuk	POLR2L
PSMA5	0.000124779	0.12721304	0.468	0.217	1	Leuk	PSMA5
CIAO2B	0.000125032	0.139077659	0.409	0.183	1	Leuk	CIAO2B
FEN1	0.000125774	0.104063111	0.155	0.025	1	Leuk	FEN1
MRPS26	0.000128306	0.122790726	0.214	0.058	1	Leuk	MRPS26
NDST3	0.00012897	0.163866156	0.168	0.033	1	Leuk	NDST3
ATP5MD	0.000130073	0.177013427	0.534	0.283	1	Leuk	ATP5MD
CCDC167	0.000132269	0.140797333	0.247	0.083	1	Leuk	CCDC167
NDUFB2	0.000135264	0.186081262	0.566	0.308	1	Leuk	NDUFB2
MTHFD1	0.000137097	0.120784094	0.155	0.025	1	Leuk	MTHFD1
PSME3	0.000137148	0.111898125	0.141	0.017	1	Leuk	PSME3
UHRF1	0.0001372	0.131682709	0.124	0.008	1	Leuk	UHRF1
HLTF	0.000140141	0.14245964	0.181	0.042	1	Leuk	HLTF
MAPKAPK3	0.000140288	0.129181524	0.263	0.092	1	Leuk	MAPKAPK3
ETHE1	0.000140525	0.142981036	0.223	0.067	1	Leuk	ETHE1
YTHDF2	0.000140831	0.120376698	0.3	0.117	1	Leuk	YTHDF2
UROS	0.000141113	0.10979392	0.185	0.042	1	Leuk	UROS
UQCRC1	0.000141838	0.103590772	0.349	0.142	1	Leuk	UQCRC1
NAP1L1	0.000142567	0.264644538	0.684	0.425	1	Leuk	NAP1L1
ST13	0.000143123	0.155356667	0.542	0.267	1	Leuk	ST13
DANCR	0.000143152	0.101134963	0.237	0.075	1	Leuk	DANCR
TTC3	0.000144159	0.138139675	0.531	0.275	1	Leuk	TTC3
FARSA	0.000146527	0.124782879	0.277	0.1	1	Leuk	FARSA
RPL4	0.000146907	0.2692684	0.919	0.775	1	Leuk	RPL4

TMEM230	0.000148616	0.131935091	0.392	0.175	1	Leuk	TMEM230
NDUFA8	0.0001501	0.124463872	0.239	0.075	1	Leuk	NDUFA8
BCL2	0.000150226	0.157623271	0.208	0.058	1	Leuk	BCL2
TXN2	0.00015173	0.129068949	0.261	0.092	1	Leuk	TXN2
UBE2V2	0.000152735	0.133288596	0.246	0.083	1	Leuk	UBE2V2
SPINT2	0.000155931	0.140781621	0.382	0.167	1	Leuk	SPINT2
MRPL57	0.000163137	0.150503124	0.523	0.267	1	Leuk	MRPL57
CENPK	0.000173	0.129866305	0.194	0.05	1	Leuk	CENPK
METTL26	0.000173107	0.132287156	0.415	0.192	1	Leuk	METTL26
CTPS1	0.000173313	0.115625888	0.122	0.008	1	Leuk	CTPS1
MAT2A	0.000174625	0.111698519	0.302	0.117	1	Leuk	MAT2A
YBX1	0.000175494	0.293087789	0.847	0.717	1	Leuk	YBX1
DUS1L	0.000176206	0.126641551	0.31	0.125	1	Leuk	DUS1L
TUBGCP2	0.000178292	0.158142207	0.176	0.042	1	Leuk	TUBGCP2
TMEM39B	0.00018044	0.119947943	0.106	0	1	Leuk	TMEM39B
CYC1	0.000181515	0.15792322	0.347	0.15	1	Leuk	CYC1
MICOS13	0.000184308	0.110899671	0.346	0.142	1	Leuk	MICOS13
CENPM	0.000186578	0.126560628	0.105	0	1	Leuk	CENPM
SF3B5	0.000190508	0.197582021	0.567	0.317	1	Leuk	SF3B5
BID	0.000194497	0.117447803	0.259	0.092	1	Leuk	BID
GNPDA1	0.000201586	0.129665848	0.118	0.008	1	Leuk	GNPDA1
PTPN11	0.000202372	0.147003382	0.176	0.042	1	Leuk	PTPN11
POLR2G	0.000203504	0.136688632	0.409	0.192	1	Leuk	POLR2G
CUX1	0.000204324	0.145504708	0.393	0.183	1	Leuk	CUX1
POLE3	0.00020481	0.111045958	0.235	0.075	1	Leuk	POLE3
AK2	0.000204891	0.112709608	0.219	0.067	1	Leuk	AK2
ACTL6A	0.000205041	0.118039617	0.164	0.033	1	Leuk	ACTL6A
TKT	0.000206036	0.197275609	0.592	0.333	1	Leuk	TKT
RPL39L	0.000207343	0.129717454	0.191	0.05	1	Leuk	RPL39L
TUBA1C	0.000207954	0.149875621	0.231	0.075	1	Leuk	TUBA1C
NCL	0.000210926	0.199736021	0.766	0.55	1	Leuk	NCL
SF3B6	0.000213613	0.139999454	0.476	0.242	1	Leuk	SF3B6
SQLE	0.000213991	0.120958074	0.135	0.017	1	Leuk	SQLE
PHGDH	0.0002161	0.119739325	0.178	0.042	1	Leuk	PHGDH
SNW1	0.000216512	0.147253559	0.227	0.075	1	Leuk	SNW1
FLYWCH2	0.000218602	0.10628998	0.164	0.033	1	Leuk	FLYWCH2
SARNP	0.000221252	0.134758552	0.279	0.108	1	Leuk	SARNP
ANP32E	0.00022167	0.155197407	0.301	0.125	1	Leuk	ANP32E
ABCE1	0.000224846	0.136101851	0.257	0.092	1	Leuk	ABCE1
RAB8A	0.000225304	0.126330414	0.219	0.067	1	Leuk	RAB8A
SRSF1	0.000232187	0.153277103	0.363	0.167	1	Leuk	SRSF1
CYB5R3	0.000239658	0.126925082	0.254	0.092	1	Leuk	CYB5R3
SPTSSA	0.00024367	0.101694604	0.102	0	1	Leuk	SPTSSA
IFI27L2	0.000245136	0.126805575	0.293	0.117	1	Leuk	IFI27L2
RAB7A	0.000246873	0.133463434	0.386	0.175	1	Leuk	RAB7A
STOML2	0.000247041	0.116995238	0.321	0.133	1	Leuk	STOML2
TMEM205	0.000248379	0.122354088	0.175	0.042	1	Leuk	TMEM205
LAPTM4B	0.000248897	0.124983506	0.145	0.025	1	Leuk	LAPTM4B
SMC2	0.000249241	0.109717891	0.177	0.042	1	Leuk	SMC2

ANAPC15	0.000250757	0.112138364	0.226	0.075	1	Leuk	ANAPC15
NDUFV1	0.000251753	0.135751285	0.332	0.142	1	Leuk	NDUFV1
GLRX5	0.000253751	0.126879237	0.381	0.175	1	Leuk	GLRX5
MYH10	0.000253944	0.110251154	0.133	0.017	1	Leuk	MYH10
MYDGF	0.000254307	0.115152597	0.318	0.133	1	Leuk	MYDGF
RRM1	0.000257333	0.13900276	0.157	0.033	1	Leuk	RRM1
C1QBP	0.000257387	0.118267147	0.362	0.158	1	Leuk	C1QBP
DDT	0.00026079	0.123820988	0.266	0.1	1	Leuk	DDT
ZNF22	0.00026636	0.143438386	0.453	0.233	1	Leuk	ZNF22
DNAJC8	0.000267035	0.115804268	0.409	0.183	1	Leuk	DNAJC8
PSMD11	0.000268686	0.11422519	0.227	0.075	1	Leuk	PSMD11
VAT1	0.000279727	0.135982141	0.391	0.192	1	Leuk	VAT1
UFD1	0.000280156	0.113414442	0.267	0.1	1	Leuk	UFD1
XRCC6	0.00028277	0.18292554	0.531	0.283	1	Leuk	XRCC6
TMEM18	0.000284711	0.108012147	0.201	0.058	1	Leuk	TMEM18
RPL18A	0.000288011	0.21983096	0.985	0.942	1	Leuk	RPL18A
NDUFC2	0.000290014	0.142887522	0.552	0.317	1	Leuk	NDUFC2
CDCA4	0.000292198	0.113741334	0.115	0.008	1	Leuk	CDCA4
HIST1H4C	0.000293006	0.600753164	0.516	0.292	1	Leuk	HIST1H4C
BTF3	0.000293298	0.201886071	0.914	0.775	1	Leuk	BTF3
KIF5B	0.000296087	0.166502885	0.385	0.183	1	Leuk	KIF5B
RPL23A	0.000299598	0.221441481	0.94	0.875	1	Leuk	RPL23A
USP5	0.000303736	0.120063847	0.115	0.008	1	Leuk	USP5
ATP5PB	0.000304268	0.140968494	0.554	0.3	1	Leuk	ATP5PB
RUNX1	0.000306155	0.17943791	0.512	0.283	1	Leuk	RUNX1
H2AFV	0.000306682	0.226332154	0.567	0.317	1	Leuk	H2AFV
PAM16	0.000307224	0.122050205	0.13	0.017	1	Leuk	PAM16
TRIM28	0.000311476	0.144812018	0.468	0.242	1	Leuk	TRIM28
RAB18	0.000312	0.13079363	0.17	0.042	1	Leuk	RAB18
ERCC1	0.00031218	0.126929644	0.214	0.067	1	Leuk	ERCC1
GPX4	0.000316292	0.135329338	0.559	0.3	1	Leuk	GPX4
TMEM147	0.000317809	0.119835104	0.252	0.092	1	Leuk	TMEM147
RPL23	0.000329549	0.149170002	0.636	0.367	1	Leuk	RPL23
DCPS	0.000331582	0.116929591	0.143	0.025	1	Leuk	DCPS
NDUFB3	0.000332324	0.129880696	0.33	0.142	1	Leuk	NDUFB3
KXD1	0.000333277	0.107075795	0.267	0.1	1	Leuk	KXD1
SNX3	0.000343302	0.146360712	0.404	0.192	1	Leuk	SNX3
MCTS1	0.000345518	0.114999465	0.227	0.075	1	Leuk	MCTS1
BCL2L1	0.000346905	0.107208056	0.28	0.108	1	Leuk	BCL2L1
CYTOR	0.000347258	0.129568552	0.168	0.042	1	Leuk	CYTOR
NDUFAB1	0.000354199	0.131019247	0.366	0.167	1	Leuk	NDUFAB1
MZT2B	0.000355961	0.186294395	0.661	0.408	1	Leuk	MZT2B
ACTR10	0.000362637	0.1211623	0.142	0.025	1	Leuk	ACTR10
TMEM109	0.000363397	0.109751513	0.224	0.075	1	Leuk	TMEM109
IFRD2	0.000371501	0.102164665	0.112	0.008	1	Leuk	IFRD2
SF3A3	0.000374412	0.118338736	0.234	0.083	1	Leuk	SF3A3
TSPAN3	0.000375813	0.108994409	0.236	0.083	1	Leuk	TSPAN3
PARK7	0.000376544	0.109446964	0.541	0.283	1	Leuk	PARK7
CANX	0.000376889	0.140315679	0.453	0.233	1	Leuk	CANX

ISOC2	0.000378476	0.12856273	0.153	0.033	1	Leuk	ISOC2
HMGA1	0.000379293	0.19829308	0.461	0.25	1	Leuk	HMGA1
NTMT1	0.000386382	0.111357055	0.11	0.008	1	Leuk	NTMT1
SAC3D1	0.000400311	0.115283031	0.167	0.042	1	Leuk	SAC3D1
CBX5	0.000404601	0.122872291	0.22	0.075	1	Leuk	CBX5
BEX1	0.000406299	0.207405352	0.316	0.142	1	Leuk	BEX1
CLTA	0.000414199	0.137253784	0.358	0.167	1	Leuk	CLTA
PCNA	0.000417206	0.157367739	0.217	0.075	1	Leuk	PCNA
RPS14	0.000418495	0.207520573	0.981	0.925	1	Leuk	RPS14
MIER1	0.000437935	0.163922998	0.265	0.108	1	Leuk	MIER1
CKS1B	0.000451035	0.128426124	0.154	0.033	1	Leuk	CKS1B
LAGE3	0.00046563	0.104367095	0.3	0.125	1	Leuk	LAGE3
MRFAP1	0.000468409	0.105056321	0.236	0.083	1	Leuk	MRFAP1
ADA	0.00047383	0.154246643	0.33	0.15	1	Leuk	ADA
TBCB	0.000480433	0.111037179	0.444	0.217	1	Leuk	TBCB
CRNDE	0.000487472	0.118690846	0.314	0.133	1	Leuk	CRNDE
SELENOF	0.000488941	0.130345356	0.473	0.242	1	Leuk	SELENOF
MORF4L2	0.000492254	0.105334557	0.193	0.058	1	Leuk	MORF4L2
LRRC47	0.000507203	0.121859679	0.136	0.025	1	Leuk	LRRC47
EIF4A3	0.000508812	0.12484945	0.267	0.108	1	Leuk	EIF4A3
PDCD10	0.000513825	0.113633612	0.246	0.092	1	Leuk	PDCD10
PPIF	0.000517332	0.113429978	0.134	0.025	1	Leuk	PPIF
MZB1	0.000525151	0.178607935	0.721	0.475	1	Leuk	MZB1
RPL41	0.000525816	0.16326505	0.992	0.992	1	Leuk	RPL41
CHORDC1	0.000534731	0.100947096	0.181	0.05	1	Leuk	CHORDC1
ALYREF	0.000543119	0.213691257	0.464	0.267	1	Leuk	ALYREF
SNRPE	0.000548599	0.152001379	0.443	0.233	1	Leuk	SNRPE
MKI67	0.000552229	0.270100754	0.146	0.033	1	Leuk	MKI67
SLTM	0.000562352	0.144727031	0.453	0.233	1	Leuk	SLTM
OAZ1	0.00056292	0.198276482	0.814	0.617	1	Leuk	OAZ1
SMC1A	0.000580813	0.135099869	0.265	0.108	1	Leuk	SMC1A
NDUFA2	0.000590474	0.10552065	0.432	0.208	1	Leuk	NDUFA2
CCND3	0.000593028	0.2634266	0.764	0.533	1	Leuk	CCND3
TMEM160	0.000593571	0.107497367	0.364	0.175	1	Leuk	TMEM160
CSRP1	0.000604204	0.107379436	0.121	0.017	1	Leuk	CSRP1
GADD45GIP1	0.000606381	0.115051961	0.391	0.183	1	Leuk	GADD45GIP1
UBE2G1	0.0006143	0.110589567	0.136	0.025	1	Leuk	UBE2G1
EIF4A1	0.000620904	0.149915416	0.61	0.367	1	Leuk	EIF4A1
EXOSC5	0.000623374	0.111896698	0.189	0.058	1	Leuk	EXOSC5
DNAJB11	0.000623656	0.128551892	0.16	0.042	1	Leuk	DNAJB11
SNHG3	0.000626088	0.137504313	0.238	0.092	1	Leuk	SNHG3
CNBP	0.000627887	0.17323396	0.687	0.442	1	Leuk	CNBP
APEX1	0.000628603	0.152899908	0.558	0.325	1	Leuk	APEX1
CLSPN	0.000648778	0.11336051	0.118	0.017	1	Leuk	CLSPN
RSRC1	0.000663091	0.100444419	0.204	0.067	1	Leuk	RSRC1
SRP9	0.000675442	0.150156061	0.587	0.342	1	Leuk	SRP9
EIF2S2	0.000695561	0.122483274	0.312	0.142	1	Leuk	EIF2S2
PPP6R1	0.000703329	0.110782534	0.287	0.125	1	Leuk	PPP6R1
TMX2	0.000705691	0.100098095	0.163	0.042	1	Leuk	TMX2

PYURF	0.000720695	0.109706655	0.385	0.183	1	Leuk	PYURF
KIFC3	0.000731303	0.100443587	0.148	0.033	1	Leuk	KIFC3
UBE2D2	0.000744663	0.125315878	0.648	0.375	1	Leuk	UBE2D2
RPL6	0.000745223	0.176123097	0.984	0.958	1	Leuk	RPL6
NDUFA6	0.000761817	0.145954583	0.42	0.225	1	Leuk	NDUFA6
TRMT112	0.00076263	0.118466069	0.609	0.35	1	Leuk	TRMT112
MYC	0.000781608	0.113700287	0.158	0.042	1	Leuk	MYC
GGCT	0.000787621	0.111999645	0.206	0.075	1	Leuk	GGCT
SNF8	0.000797247	0.130851851	0.36	0.175	1	Leuk	SNF8
PSMB7	0.000803916	0.108247669	0.383	0.183	1	Leuk	PSMB7
ANKIB1	0.000805491	0.116338803	0.102	0.008	1	Leuk	ANKIB1
UGT3A2	0.000809641	0.108580691	0.115	0.017	1	Leuk	UGT3A2
SMIM24	0.000815003	0.161517518	0.584	0.342	1	Leuk	SMIM24
PGK1	0.000815969	0.181136857	0.664	0.425	1	Leuk	PGK1
CENPU	0.000829525	0.100016071	0.145	0.033	1	Leuk	CENPU
THRAP3	0.000842395	0.162314845	0.492	0.275	1	Leuk	THRAP3
CHCHD2	0.000850755	0.263153793	0.829	0.675	1	Leuk	CHCHD2
RPL37A	0.000851109	0.206094515	0.933	0.817	1	Leuk	RPL37A
PGAM1	0.0008646	0.12745306	0.485	0.258	1	Leuk	PGAM1
PRPF31	0.000864943	0.102773877	0.187	0.058	1	Leuk	PRPF31
GTF2F2	0.000879059	0.107146329	0.13	0.025	1	Leuk	GTF2F2
RPL18	0.000879756	0.157241418	0.979	0.975	1	Leuk	RPL18
MRPL37	0.000892892	0.111270934	0.238	0.092	1	Leuk	MRPL37
SAMM50	0.000893527	0.104574443	0.144	0.033	1	Leuk	SAMM50
CENPF	0.000916798	0.278322303	0.14	0.033	1	Leuk	CENPF
RSL1D1	0.000918114	0.170900092	0.494	0.283	1	Leuk	RSL1D1
MSL2	0.000942019	0.101038467	0.13	0.025	1	Leuk	MSL2
SNHG29	0.00098236	0.210749267	0.833	0.625	1	Leuk	SNHG29
ERGIC3	0.001008902	0.125750818	0.374	0.183	1	Leuk	ERGIC3
SNX2	0.001015608	0.110513999	0.153	0.042	1	Leuk	SNX2
PRDX3	0.001022892	0.109916844	0.349	0.167	1	Leuk	PRDX3
LSM2	0.001027344	0.100568411	0.429	0.217	1	Leuk	LSM2
SEC61B	0.001028428	0.23661484	0.679	0.45	1	Leuk	SEC61B
GGA1	0.001030654	0.11116509	0.114	0.017	1	Leuk	GGA1
MAP2K3	0.001039036	0.104571268	0.142	0.033	1	Leuk	MAP2K3
UBE2R2	0.001049224	0.101542296	0.345	0.167	1	Leuk	UBE2R2
RPS15	0.001051976	0.177502288	0.983	0.933	1	Leuk	RPS15
PSMD7	0.001054306	0.101446564	0.331	0.158	1	Leuk	PSMD7
SH2D2A	0.001098636	0.108078065	0.233	0.092	1	Leuk	SH2D2A
ATF6	0.001103414	0.102153717	0.128	0.025	1	Leuk	ATF6
CDCA7	0.001124594	0.10488462	0.372	0.183	1	Leuk	CDCA7
RPS9	0.00112982	0.186684899	0.973	0.925	1	Leuk	RPS9
NDUFB11	0.001135812	0.184139259	0.645	0.425	1	Leuk	NDUFB11
EIF3A	0.001158465	0.117229363	0.529	0.292	1	Leuk	EIF3A
CHI3L2	0.001161724	0.446109072	0.694	0.567	1	Leuk	CHI3L2
RBM6	0.00116238	0.12679769	0.256	0.108	1	Leuk	RBM6
SSB	0.001171296	0.141661592	0.452	0.258	1	Leuk	SSB
EEF1A1	0.001175312	0.17622938	0.998	0.992	1	Leuk	EEF1A1
DBI	0.001193367	0.130257936	0.514	0.283	1	Leuk	DBI

SEC61A1	0.001214798	0.102824585	0.168	0.05	1	Leuk	SEC61A1
AP2M1	0.001216116	0.104380257	0.498	0.267	1	Leuk	AP2M1
RAB4A	0.001228417	0.114853314	0.23	0.092	1	Leuk	RAB4A
WNK1	0.001229842	0.129591026	0.306	0.142	1	Leuk	WNK1
RPL12	0.001255584	0.218075173	0.961	0.883	1	Leuk	RPL12
GSPT1	0.001266062	0.102486135	0.351	0.175	1	Leuk	GSPT1
RPL36	0.001298763	0.204352306	0.952	0.867	1	Leuk	RPL36
HOPX	0.001300867	0.189934972	0.456	0.275	1	Leuk	HOPX
NDUFB7	0.001307096	0.120248738	0.459	0.242	1	Leuk	NDUFB7
LAMTOR5	0.001310558	0.154316414	0.485	0.275	1	Leuk	LAMTOR5
SYPL1	0.00131299	0.146418965	0.43	0.233	1	Leuk	SYPL1
XRN2	0.001335711	0.107846276	0.367	0.183	1	Leuk	XRN2
CTSB	0.001344801	0.119783899	0.136	0.033	1	Leuk	CTSB
PLIN2	0.001345663	0.131970303	0.297	0.142	1	Leuk	PLIN2
DNAJC9	0.001356118	0.113854434	0.316	0.15	1	Leuk	DNAJC9
COA8	0.001371401	0.103280758	0.123	0.025	1	Leuk	COA8
COPB2	0.001383183	0.100058761	0.19	0.067	1	Leuk	COPB2
CKLF	0.001408237	0.112579104	0.565	0.325	1	Leuk	CKLF
NDUFAF8	0.001412795	0.103824316	0.306	0.142	1	Leuk	NDUFAF8
SRSF2	0.001416637	0.124805542	0.681	0.425	1	Leuk	SRSF2
CCDC124	0.001432944	0.10219314	0.306	0.142	1	Leuk	CCDC124
CHST12	0.001475758	0.107167897	0.254	0.108	1	Leuk	CHST12
ANP32B	0.001487377	0.195378165	0.716	0.475	1	Leuk	ANP32B
EEF1G	0.001516564	0.185567427	0.9	0.792	1	Leuk	EEF1G
GABARAP	0.001523552	0.161960577	0.516	0.292	1	Leuk	GABARAP
DCAF7	0.001541507	0.119852304	0.406	0.208	1	Leuk	DCAF7
PPIL3	0.001552139	0.10593749	0.121	0.025	1	Leuk	PPIL3
UBA52	0.001564138	0.169793366	0.93	0.725	1	Leuk	UBA52
MFHAS1	0.00156627	0.10761923	0.191	0.067	1	Leuk	MFHAS1
FDFT1	0.001727817	0.134899701	0.362	0.183	1	Leuk	FDFT1
PABPN1	0.001738169	0.161665414	0.653	0.425	1	Leuk	PABPN1
SLC39A3	0.001765661	0.100177396	0.17	0.058	1	Leuk	SLC39A3
COX7C	0.00179975	0.155956846	0.858	0.658	1	Leuk	COX7C
MYL12B	0.001806075	0.145044663	0.769	0.517	1	Leuk	MYL12B
ANAPC5	0.001820677	0.119827765	0.348	0.175	1	Leuk	ANAPC5
EIF3D	0.001837394	0.109931851	0.493	0.283	1	Leuk	EIF3D
RPS4X	0.001838136	0.166087075	0.977	0.958	1	Leuk	RPS4X
LSM7	0.00183966	0.144877838	0.553	0.333	1	Leuk	LSM7
SERPINB1	0.001846517	0.118088466	0.337	0.167	1	Leuk	SERPINB1
CCT7	0.001853037	0.11566388	0.481	0.275	1	Leuk	CCT7
NDUFA7	0.001909814	0.100766981	0.348	0.175	1	Leuk	NDUFA7
ILKAP	0.001935711	0.110796222	0.173	0.058	1	Leuk	ILKAP
ZNF32	0.001967351	0.100117523	0.187	0.067	1	Leuk	ZNF32
LIMS2	0.001988806	0.105958432	0.211	0.083	1	Leuk	LIMS2
TALDO1	0.00208933	0.133390853	0.576	0.342	1	Leuk	TALDO1
ITGA4	0.00216942	0.158425446	0.621	0.375	1	Leuk	ITGA4
MAP1A	0.002172566	0.128107963	0.285	0.133	1	Leuk	MAP1A
ATAD2	0.002251496	0.10068319	0.143	0.042	1	Leuk	ATAD2
PSMA7	0.002291966	0.161298575	0.649	0.408	1	Leuk	PSMA7

RPL38	0.002324558	0.15428121	0.845	0.658	1	Leuk	RPL38
SPON2	0.002335493	0.151254122	0.14	0.042	1	Leuk	SPON2
AIF1	0.002384069	0.138279915	0.58	0.35	1	Leuk	AIF1
VAMP5	0.002548508	0.102877809	0.279	0.133	1	Leuk	VAMP5
H3F3A	0.002552165	0.189986001	0.979	0.95	1	Leuk	H3F3A
ATP5MF	0.002595977	0.150983887	0.597	0.367	1	Leuk	ATP5MF
LGALS9	0.002712418	0.102643381	0.313	0.158	1	Leuk	LGALS9
TUBB2A	0.002735883	0.110189316	0.25	0.117	1	Leuk	TUBB2A
MARCKSL1	0.002772607	0.108306119	0.701	0.425	1	Leuk	MARCKSL1
NDUFA13	0.002905675	0.139699978	0.753	0.517	1	Leuk	NDUFA13
PCNX4	0.002967919	0.109260593	0.267	0.125	1	Leuk	PCNX4
NOP10	0.002996255	0.125090118	0.533	0.317	1	Leuk	NOP10
RPLP2	0.003052868	0.167747734	0.965	0.917	1	Leuk	RPLP2
WDR43	0.003081926	0.107545605	0.201	0.083	1	Leuk	WDR43
NUSAP1	0.00313604	0.160424245	0.139	0.042	1	Leuk	NUSAP1
GPI	0.003137245	0.103461022	0.346	0.183	1	Leuk	GPI
RHOA	0.003152665	0.135085061	0.803	0.558	1	Leuk	RHOA
COX7B	0.00315677	0.143175673	0.535	0.325	1	Leuk	COX7B
SAFB	0.003244132	0.104299594	0.253	0.117	1	Leuk	SAFB
CDKN2A	0.003508425	0.172866591	0.627	0.425	1	Leuk	CDKN2A
RBM8A	0.003555073	0.10124948	0.497	0.283	1	Leuk	RBM8A
ATP5F1D	0.003641758	0.114672842	0.594	0.358	1	Leuk	ATP5F1D
RPS26	0.003755182	0.185823428	0.842	0.625	1	Leuk	RPS26
SUB1	0.003831356	0.171758711	0.806	0.575	1	Leuk	SUB1
HNRNPF	0.004001252	0.144987323	0.595	0.367	1	Leuk	HNRNPF
SELENOH	0.004044691	0.133273498	0.711	0.483	1	Leuk	SELENOH
HSPA8	0.004094092	0.144575115	0.813	0.6	1	Leuk	HSPA8
TOP2A	0.004173134	0.199865076	0.107	0.025	1	Leuk	TOP2A
SPINK2	0.004235343	0.131015333	0.544	0.342	1	Leuk	SPINK2
ADAM10	0.00426289	0.1165219	0.522	0.308	1	Leuk	ADAM10
CWC25	0.004351457	0.108001611	0.121	0.033	1	Leuk	CWC25
MSI2	0.004355418	0.128547814	0.512	0.308	1	Leuk	MSI2
C4orf48	0.004409426	0.102134124	0.106	0.025	1	Leuk	C4orf48
TIA1	0.004549735	0.118097556	0.291	0.15	1	Leuk	TIA1
RING1	0.004627964	0.111189605	0.158	0.058	1	Leuk	RING1
CALM2	0.004829256	0.15707215	0.754	0.517	1	Leuk	CALM2
RPL26	0.00486234	0.183662433	0.979	0.942	1	Leuk	RPL26
PPP3R1	0.004981958	0.115868661	0.143	0.05	1	Leuk	PPP3R1
COX6C	0.005046843	0.176279198	0.779	0.542	1	Leuk	COX6C
P4HB	0.00546347	0.114759111	0.524	0.325	1	Leuk	P4HB
H2AFX	0.005592281	0.102887636	0.276	0.142	1	Leuk	H2AFX
UXT	0.005647093	0.123985729	0.606	0.375	1	Leuk	UXT
RPS19BP1	0.005840173	0.100520104	0.3	0.158	1	Leuk	RPS19BP1
HSP90AA1	0.006028379	0.218875467	0.86	0.783	1	Leuk	HSP90AA1
TUFM	0.006230132	0.12139594	0.446	0.267	1	Leuk	TUFM
GNAS	0.00627238	0.136087065	0.883	0.733	1	Leuk	GNAS
MTDH	0.006356884	0.103676795	0.601	0.367	1	Leuk	MTDH
SEPTIN6	0.00650403	0.191923897	0.845	0.65	1	Leuk	SEPTIN6
SSR4	0.006735585	0.130185281	0.71	0.483	1	Leuk	SSR4

MYL6	0.008667289	0.165430078	0.96	0.858	1	Leuk	MYL6
DNTT	0.00884751	0.101018457	0.236	0.117	1	Leuk	DNTT
HPGD	0.00939138	0.14224485	0.605	0.425	1	Leuk	HPGD
