# Supplementary material for: High CD44 expression and enhanced E-selectin binding identified as biomarkers of chemoresistant leukemic cells in human T-ALL
Source: Leukemia. 2024 Nov 24;39(2):323–36. doi: 10.1038/s41375-024-02473-7 (PMC11794132; doi:10.1038/s41375-024-02473-7)
Supplement: Supplementary file 9 — Supplemental Table 8 [file 41375_2024_2473_MOESM9_ESM.pdf]

upregulated genes in Ki67neg CD44high leukemic cells from Library 4 (M144 & M172) (Supplementary Figure 11e)

	p_val	avg_log2FC	pct.1	pct.2	p_val_adj	cluster	gene
CD44	6.11E-192	1.619397796	1	0.131	2.23E-187	CD44 > 1 & MKI67 < 1 Leuk	CD44
MALAT1	2.18E-12	0.586654733	1	1	7.99E-08	CD44 > 1 & MKI67 < 1 Leuk	MALAT1
TXNIP	1.34E-10	0.800324016	0.735	0.52	4.91E-06	CD44 > 1 & MKI67 < 1 Leuk	TXNIP
JUN	2.42E-10	0.739753197	0.872	0.866	8.85E-06	CD44 > 1 & MKI67 < 1 Leuk	JUN
TSC22D3	6.21E-08	0.567817419	0.803	0.72	0.002274137	CD44 > 1 & MKI67 < 1 Leuk	TSC22D3
DUSP1	1.38E-07	0.744457491	0.692	0.618	0.005065567	CD44 > 1 & MKI67 < 1 Leuk	DUSP1
EEF1A1	6.59E-07	0.353155235	1	0.997	0.024114673	CD44 > 1 & MKI67 < 1 Leuk	EEF1A1
TPT1	9.16E-07	0.359223391	0.983	0.994	0.033515794	CD44 > 1 & MKI67 < 1 Leuk	TPT1
ZFP36L2	3.35E-06	0.592762933	0.744	0.714	0.122574883	CD44 > 1 & MKI67 < 1 Leuk	ZFP36L2
NFKB1	5.48E-06	0.282785063	0.179	0.073	0.200699522	CD44 > 1 & MKI67 < 1 Leuk	NFKB1
CD69	6.49E-06	0.46651752	0.761	0.716	0.237609866	CD44 > 1 & MKI67 < 1 Leuk	CD69
KLF6	7.92E-06	0.433461887	0.821	0.776	0.28981369	CD44 > 1 & MKI67 < 1 Leuk	KLF6
FOS	9.28E-06	0.715669522	0.752	0.693	0.339514778	CD44 > 1 & MKI67 < 1 Leuk	FOS
RPL34	1.25E-05	0.263797357	0.957	0.985	0.458870535	CD44 > 1 & MKI67 < 1 Leuk	RPL34
RPL11	1.31E-05	0.247083897	0.983	0.995	0.478822067	CD44 > 1 & MKI67 < 1 Leuk	RPL11
TIMP1	1.45E-05	0.368816031	0.316	0.18	0.530516817	CD44 > 1 & MKI67 < 1 Leuk	TIMP1
IL7R	1.46E-05	0.606354662	0.598	0.459	0.532573134	CD44 > 1 & MKI67 < 1 Leuk	IL7R
RGS1	4.45E-05	0.47079664	0.299	0.176	1	CD44 > 1 & MKI67 < 1 Leuk	RGS1
KLF2	4.77E-05	0.327783122	0.179	0.08	1	CD44 > 1 & MKI67 < 1 Leuk	KLF2
H3F3B	9.91E-05	0.388700138	0.974	0.991	1	CD44 > 1 & MKI67 < 1 Leuk	H3F3B
TP53INP1	0.000113961	0.419671467	0.282	0.172	1	CD44 > 1 & MKI67 < 1 Leuk	TP53INP1
HSH2D	0.000129135	0.326130213	0.342	0.222	1	CD44 > 1 & MKI67 < 1 Leuk	HSH2D
RPL9	0.000193069	0.234376794	0.915	0.966	1	CD44 > 1 & MKI67 < 1 Leuk	RPL9
NFKBIA	0.000200326	0.582171016	0.487	0.393	1	CD44 > 1 & MKI67 < 1 Leuk	NFKBIA
KHDRBS3	0.000222332	0.374050094	0.368	0.257	1	CD44 > 1 & MKI67 < 1 Leuk	KHDRBS3
RPS28	0.00028184	0.209858975	0.915	0.983	1	CD44 > 1 & MKI67 < 1 Leuk	RPS28
SMURF1	0.000294465	0.194176055	0.103	0.039	1	CD44 > 1 & MKI67 < 1 Leuk	SMURF1
CLEC12A	0.000303503	0.217597927	0.154	0.071	1	CD44 > 1 & MKI67 < 1 Leuk	CLEC12A
AHR	0.000433098	0.401344536	0.274	0.172	1	CD44 > 1 & MKI67 < 1 Leuk	AHR
LDB1	0.000600703	0.216085545	0.145	0.068	1	CD44 > 1 & MKI67 < 1 Leuk	LDB1
ENG	0.000774009	0.250334786	0.162	0.082	1	CD44 > 1 & MKI67 < 1 Leuk	ENG
BTG1	0.001066828	0.290839594	0.726	0.719	1	CD44 > 1 & MKI67 < 1 Leuk	BTG1
RPL35A	0.001245487	0.212388408	0.94	0.981	1	CD44 > 1 & MKI67 < 1 Leuk	RPL35A
SSR2	0.001284202	0.299851973	0.632	0.637	1	CD44 > 1 & MKI67 < 1 Leuk	SSR2
SMAP2	0.001334976	0.364259058	0.436	0.36	1	CD44 > 1 & MKI67 < 1 Leuk	SMAP2
PNRC1	0.001420823	0.280428295	0.709	0.718	1	CD44 > 1 & MKI67 < 1 Leuk	PNRC1
PIK3IP1	0.001435801	0.171484197	0.145	0.072	1	CD44 > 1 & MKI67 < 1 Leuk	PIK3IP1
NR3C1	0.001596522	0.358138631	0.41	0.328	1	CD44 > 1 & MKI67 < 1 Leuk	NR3C1
CD38	0.001832228	0.331026494	0.427	0.335	1	CD44 > 1 & MKI67 < 1 Leuk	CD38
RNF169	0.002600281	0.353112655	0.222	0.143	1	CD44 > 1 & MKI67 < 1 Leuk	RNF169
CD7	0.002618092	0.320218669	0.624	0.588	1	CD44 > 1 & MKI67 < 1 Leuk	CD7
PDE3B	0.002668026	0.369880846	0.35	0.264	1	CD44 > 1 & MKI67 < 1 Leuk	PDE3B
FTL	0.002867867	0.25642448	0.957	0.967	1	CD44 > 1 & MKI67 < 1 Leuk	FTL
TENT5A	0.002904057	0.290085282	0.197	0.117	1	CD44 > 1 & MKI67 < 1 Leuk	TENT5A
MYLIP	0.003187509	0.291167974	0.308	0.219	1	CD44 > 1 & MKI67 < 1 Leuk	MYLIP
PDZD8	0.003425498	0.231338794	0.205	0.128	1	CD44 > 1 & MKI67 < 1 Leuk	PDZD8
RPS3A	0.003585202	0.164504162	0.966	0.991	1	CD44 > 1 & MKI67 < 1 Leuk	RPS3A
JUND	0.004903589	0.390298093	0.88	0.919	1	CD44 > 1 & MKI67 < 1 Leuk	JUND
MGLL	0.005200064	0.19846216	0.145	0.08	1	CD44 > 1 & MKI67 < 1 Leuk	MGLL
LINC01578	0.00545586	0.423373428	0.615	0.657	1	CD44 > 1 & MKI67 < 1 Leuk	LINC01578
RPS23	0.005669962	0.172382657	0.957	0.991	1	CD44 > 1 & MKI67 < 1 Leuk	RPS23

CRADD	0.005698737	0.140782733	0.12	0.061	1	CD44 > 1 & MKI67 < 1 Leuk	CRADD
GIHCG	0.005842215	0.330322302	0.692	0.798	1	CD44 > 1 & MKI67 < 1 Leuk	GIHCG
ETV6	0.005918573	0.355294657	0.291	0.222	1	CD44 > 1 & MKI67 < 1 Leuk	ETV6
GLS	0.006049759	0.351882564	0.427	0.364	1	CD44 > 1 & MKI67 < 1 Leuk	GLS
ANKRD28	0.00606567	0.362345639	0.402	0.337	1	CD44 > 1 & MKI67 < 1 Leuk	ANKRD28
RPL39	0.007573812	0.157078699	0.949	0.978	1	CD44 > 1 & MKI67 < 1 Leuk	RPL39
TRGV10	0.007991237	0.227000505	0.171	0.102	1	CD44 > 1 & MKI67 < 1 Leuk	TRGV10
JUNB	0.00888735	0.389376978	0.47	0.405	1	CD44 > 1 & MKI67 < 1 Leuk	JUNB
LINC00426	0.009128205	0.29905601	0.188	0.121	1	CD44 > 1 & MKI67 < 1 Leuk	LINC00426
DAGLB	0.009307117	0.234648265	0.145	0.086	1	CD44 > 1 & MKI67 < 1 Leuk	DAGLB
BAZ2B	0.009857704	0.2895431	0.256	0.19	1	CD44 > 1 & MKI67 < 1 Leuk	BAZ2B
GAPDH	1.28E-21	0.882837989	0.983	0.906	4.68E-17	Leuk	GAPDH
STMN1	2.12E-16	0.88705454	0.868	0.598	7.77E-12	Leuk	STMN1
TUBA1B	2.87E-15	1.243196071	0.749	0.419	1.05E-10	Leuk	TUBA1B
H3F3A	9.67E-15	0.738957247	0.959	0.863	3.54E-10	Leuk	H3F3A
FXYD2	4.01E-14	0.87774686	0.487	0.111	1.47E-09	Leuk	FXYD2
CHI3L2	8.16E-13	1.360696156	0.474	0.128	2.99E-08	Leuk	CHI3L2
COX6C	2.30E-12	0.516654012	0.7	0.333	8.41E-08	Leuk	COX6C
HMGB2	6.74E-12	0.943645884	0.48	0.137	2.47E-07	Leuk	HMGB2
ACTB	7.47E-12	0.59892118	0.972	0.94	2.73E-07	Leuk	ACTB
TRBC2	1.28E-11	0.507737896	0.577	0.214	4.70E-07	Leuk	TRBC2
CHCHD2	1.30E-11	0.52963624	0.846	0.564	4.76E-07	Leuk	CHCHD2
COX8A	2.26E-11	0.54921954	0.682	0.308	8.28E-07	Leuk	COX8A
TRBV11-2	3.10E-11	1.270961178	0.476	0.145	1.13E-06	Leuk	TRBV11-2
RANBP1	3.10E-11	0.456577263	0.401	0.077	1.14E-06	Leuk	RANBP1
DEK	4.70E-11	0.442849213	0.389	0.068	1.72E-06	Leuk	DEK
TYMS	7.35E-11	0.637034314	0.404	0.094	2.69E-06	Leuk	TYMS
AC002454.1	9.84E-11	0.468953708	0.394	0.077	3.60E-06	Leuk	AC002454.1
SIVA1	1.28E-10	0.492464691	0.549	0.205	4.67E-06	Leuk	SIVA1
COX6B1	1.42E-10	0.423778633	0.684	0.316	5.18E-06	Leuk	COX6B1
ALYREF	7.78E-10	0.428802007	0.43	0.12	2.85E-05	Leuk	ALYREF
NDUFB6	1.07E-09	0.351763468	0.324	0.043	3.91E-05	Leuk	NDUFB6
EZR	2.00E-09	0.438131865	0.39	0.103	7.33E-05	Leuk	EZR
TRAV29DV5	2.23E-09	0.445267737	0.327	0.051	8.18E-05	Leuk	TRAV29DV5
RPS26	2.58E-09	0.530864806	0.897	0.692	9.43E-05	Leuk	RPS26
GSTP1	3.13E-09	0.481832679	0.887	0.692	0.000114565	Leuk	GSTP1
IDH2	4.61E-09	0.409367262	0.594	0.274	0.000168588	Leuk	IDH2
C1QBP	5.49E-09	0.346285352	0.409	0.111	0.000200906	Leuk	C1QBP
H2AFZ	6.17E-09	0.632651187	0.713	0.444	0.000225752	Leuk	H2AFZ
FABP5	7.58E-09	0.410881441	0.32	0.06	0.000277484	Leuk	FABP5
HIST1H4C	9.63E-09	1.040354188	0.556	0.274	0.000352568	Leuk	HIST1H4C
H2AFY	1.73E-08	0.43983396	0.737	0.427	0.000632345	Leuk	H2AFY
CDT1	1.74E-08	0.307302636	0.25	0.017	0.000637848	Leuk	CDT1
PCNA	2.34E-08	0.419375701	0.269	0.034	0.00085713	Leuk	PCNA
JPT1	3.08E-08	0.490779949	0.494	0.214	0.00112601	Leuk	JPT1
ATP5F1B	3.79E-08	0.39492245	0.691	0.376	0.00138759	Leuk	ATP5F1B
TXN	3.85E-08	0.438146366	0.679	0.342	0.001408282	Leuk	TXN
CFL1	4.09E-08	0.403441548	0.914	0.786	0.001497536	Leuk	CFL1
NDUFB10	4.11E-08	0.292668245	0.397	0.12	0.001505727	Leuk	NDUFB10
COX6A1	4.38E-08	0.41739312	0.747	0.427	0.001604175	Leuk	COX6A1
TUBB	4.40E-08	0.781075649	0.795	0.598	0.001609454	Leuk	TUBB
COX7B	5.05E-08	0.339819757	0.474	0.188	0.001847019	Leuk	COX7B
DUT	5.44E-08	0.487381168	0.51	0.231	0.001989948	Leuk	DUT
CKS2	6.84E-08	0.365545341	0.26	0.034	0.00250307	Leuk	CKS2

RAN	8.18E-08	0.36514237	0.579	0.274	0.002993451	Leuk	RAN
HMGB1	8.42E-08	0.485985461	0.957	0.897	0.003083614	Leuk	HMGB1
HMGN2	8.51E-08	0.704572187	0.769	0.564	0.003113867	Leuk	HMGN2
AKR1B1	8.89E-08	0.37553947	0.26	0.034	0.003253843	Leuk	AKR1B1
CKS1B	9.12E-08	0.345199515	0.243	0.026	0.003339334	Leuk	CKS1B
LDHA	1.01E-07	0.405945287	0.585	0.291	0.00370443	Leuk	LDHA
DNAJC9	1.04E-07	0.351508184	0.364	0.103	0.003807387	Leuk	DNAJC9
SNRPG	1.06E-07	0.296942927	0.332	0.085	0.003871116	Leuk	SNRPG
RBM3	1.10E-07	0.415953157	0.704	0.41	0.004032728	Leuk	RBM3
MCM7	1.25E-07	0.350587445	0.353	0.103	0.004574213	Leuk	MCM7
POMP	1.39E-07	0.302811267	0.515	0.214	0.005085729	Leuk	POMP
NAP1L1	1.66E-07	0.375758027	0.614	0.325	0.006059581	Leuk	NAP1L1
SIX6	1.76E-07	0.406032164	0.278	0.051	0.006459974	Leuk	SIX6
ERH	1.85E-07	0.37065715	0.452	0.188	0.006785929	Leuk	ERH
PPIA	1.90E-07	0.381374254	0.953	0.855	0.00693597	Leuk	PPIA
RALY	1.96E-07	0.321516797	0.529	0.231	0.007156493	Leuk	RALY
NPM1	2.00E-07	0.40350386	0.854	0.581	0.007308987	Leuk	NPM1
TMSB4X	2.19E-07	0.425424005	0.991	0.957	0.008022222	Leuk	TMSB4X
YBX1	2.34E-07	0.375415781	0.835	0.632	0.008554031	Leuk	YBX1
PSMA7	2.49E-07	0.367544437	0.624	0.342	0.009104977	Leuk	PSMA7
PSME1	2.51E-07	0.311559861	0.696	0.385	0.009184641	Leuk	PSME1
PTMA	2.58E-07	0.370580196	0.995	1	0.009452188	Leuk	PTMA
SNRPE	2.58E-07	0.307346901	0.439	0.154	0.009457067	Leuk	SNRPE
ATP5MF	2.63E-07	0.317315175	0.564	0.274	0.009611581	Leuk	ATP5MF
HSP90AA1	2.69E-07	0.360393985	0.882	0.701	0.009841332	Leuk	HSP90AA1
DNMT1	2.71E-07	0.298502884	0.284	0.06	0.00993433	Leuk	DNMT1
GYPC	2.74E-07	0.337771855	0.328	0.085	0.010030077	Leuk	GYPC
PSMB7	2.79E-07	0.305807144	0.398	0.137	0.010228068	Leuk	PSMB7
NME1	2.85E-07	0.264801279	0.329	0.085	0.010417889	Leuk	NME1
NASP	3.08E-07	0.390755518	0.491	0.222	0.011290149	Leuk	NASP
OAZ1	3.62E-07	0.331519748	0.71	0.385	0.013264356	Leuk	OAZ1
PSMA6	3.69E-07	0.334646093	0.722	0.41	0.013520565	Leuk	PSMA6
PCLAF	3.88E-07	0.359549131	0.321	0.085	0.014201561	Leuk	PCLAF
CALR	4.06E-07	0.367805969	0.682	0.376	0.014843051	Leuk	CALR
EIF4A1	4.38E-07	0.37540382	0.746	0.462	0.016020869	Leuk	EIF4A1
PFN1	4.46E-07	0.391158828	0.933	0.855	0.016327835	Leuk	PFN1
SUZ12	4.55E-07	0.275427164	0.34	0.094	0.016635566	Leuk	SUZ12
TECR	5.93E-07	0.303778703	0.413	0.154	0.021701339	Leuk	TECR
NME2	6.20E-07	0.293824072	0.611	0.299	0.02270039	Leuk	NME2
LSM4	6.98E-07	0.336721388	0.449	0.197	0.025555544	Leuk	LSM4
CDK2AP2	7.24E-07	0.37836938	0.304	0.085	0.026507805	Leuk	CDK2AP2
TXNL4A	7.64E-07	0.262033707	0.355	0.111	0.027965129	Leuk	TXNL4A
BANF1	7.89E-07	0.209713448	0.487	0.197	0.0288911	Leuk	BANF1
NDUFB7	7.94E-07	0.267044923	0.422	0.154	0.029063076	Leuk	NDUFB7
ARHGDIB	8.09E-07	0.409573898	0.913	0.769	0.029616813	Leuk	ARHGDIB
SDHC	8.59E-07	0.237115099	0.248	0.043	0.031439096	Leuk	SDHC
MRPL51	8.91E-07	0.266483343	0.375	0.128	0.032627572	Leuk	MRPL51
SNRPF	9.36E-07	0.267081757	0.408	0.154	0.034261005	Leuk	SNRPF
LEF1	9.54E-07	0.265439773	0.337	0.103	0.034912855	Leuk	LEF1
EWSR1	1.01E-06	0.24810853	0.533	0.239	0.036960146	Leuk	EWSR1
BEX3	1.04E-06	0.284818712	0.45	0.188	0.038203741	Leuk	BEX3
GTF3C5	1.10E-06	0.29735582	0.352	0.111	0.040421187	Leuk	GTF3C5
SNRPC	1.16E-06	0.284476668	0.456	0.179	0.042407779	Leuk	SNRPC
MYL6	1.25E-06	0.379293006	0.901	0.709	0.045602744	Leuk	MYL6

NDUFA6	1.49E-06	0.305481436	0.41	0.162	0.054476934	Leuk	NDUFA6
TOMM6	1.49E-06	0.257482491	0.436	0.179	0.054587988	Leuk	TOMM6
ILF2	1.52E-06	0.324146552	0.55	0.274	0.055667949	Leuk	ILF2
MLLT11	1.53E-06	0.234571135	0.34	0.103	0.055973405	Leuk	MLLT11
HNRNPAB	1.66E-06	0.305989468	0.433	0.179	0.060717087	Leuk	HNRNPAB
HNRNPC	1.69E-06	0.284266768	0.72	0.376	0.061707042	Leuk	HNRNPC
INSIG1	1.76E-06	0.283198953	0.296	0.085	0.064366775	Leuk	INSIG1
TMBIM6	1.88E-06	0.29948911	0.484	0.205	0.068835791	Leuk	TMBIM6
MICOS10	1.94E-06	0.235755624	0.278	0.068	0.070859992	Leuk	MICOS10
ACTG1	1.99E-06	0.42397309	0.839	0.701	0.072750935	Leuk	ACTG1
SF3B2	2.08E-06	0.258446037	0.525	0.248	0.076130496	Leuk	SF3B2
MIF	2.15E-06	0.363640922	0.888	0.667	0.07868766	Leuk	MIF
CCT2	2.15E-06	0.213148591	0.444	0.179	0.078849716	Leuk	CCT2
NUSAP1	2.16E-06	0.269510008	0.18	0.009	0.078983496	Leuk	NUSAP1
UHRF1	2.26E-06	0.199513842	0.233	0.043	0.082884205	Leuk	UHRF1
CCDC124	2.54E-06	0.209230024	0.264	0.06	0.092959348	Leuk	CCDC124
MARCKSL1	2.63E-06	0.416070616	0.646	0.393	0.096218856	Leuk	MARCKSL1
FDPS	2.65E-06	0.355458822	0.392	0.154	0.097058314	Leuk	FDPS
CALM3	2.94E-06	0.299344554	0.459	0.205	0.107657003	Leuk	CALM3
LIME1	2.99E-06	0.292463664	0.376	0.137	0.109584469	Leuk	LIME1
MYL12B	3.05E-06	0.277274125	0.601	0.316	0.111574561	Leuk	MYL12B
H2AFX	3.06E-06	0.30672014	0.267	0.068	0.111957994	Leuk	H2AFX
GNA15	3.13E-06	0.224414998	0.677	0.385	0.114549625	Leuk	GNA15
RPS17	3.40E-06	0.309928426	0.736	0.444	0.124343267	Leuk	RPS17
SRI	3.55E-06	0.225639096	0.232	0.043	0.130083417	Leuk	SRI
RAB11B	3.59E-06	0.252097515	0.368	0.137	0.1312354	Leuk	RAB11B
DNAJA1	3.68E-06	0.191670981	0.272	0.068	0.134513789	Leuk	DNAJA1
MAD2L2	3.75E-06	0.273786659	0.386	0.145	0.137104595	Leuk	MAD2L2
RBX1	3.80E-06	0.235865278	0.384	0.145	0.138941181	Leuk	RBX1
SUB1	3.81E-06	0.358263959	0.761	0.538	0.139626984	Leuk	SUB1
PPP1CA	3.87E-06	0.246167311	0.515	0.248	0.141753978	Leuk	PPP1CA
YIF1B	3.93E-06	0.212266454	0.231	0.043	0.14379838	Leuk	YIF1B
ACSF3	4.06E-06	0.294633711	0.308	0.094	0.148442203	Leuk	ACSF3
TMPO	4.12E-06	0.293561499	0.38	0.145	0.150967451	Leuk	TMPO
SNRPD2	4.96E-06	0.300509052	0.744	0.444	0.181611713	Leuk	SNRPD2
UQCR11	5.18E-06	0.265939444	0.535	0.265	0.18955373	Leuk	UQCR11
LMNB1	5.65E-06	0.224497343	0.244	0.06	0.206654588	Leuk	LMNB1
MT-ND5	5.85E-06	0.346124331	0.765	0.487	0.214202814	Leuk	MT-ND5
STAT5A	6.10E-06	0.242404312	0.236	0.051	0.223443364	Leuk	STAT5A
NDUFB11	6.36E-06	0.281689812	0.62	0.316	0.232761983	Leuk	NDUFB11
HIST1H3D	6.37E-06	0.354249554	0.242	0.06	0.23309144	Leuk	HIST1H3D
POLR2L	7.03E-06	0.200642096	0.387	0.145	0.257235578	Leuk	POLR2L
VAMP5	7.53E-06	0.256422112	0.232	0.051	0.275506134	Leuk	VAMP5
HPS4	8.00E-06	0.216951932	0.178	0.017	0.292728764	Leuk	HPS4
MFHAS1	8.24E-06	0.222314944	0.194	0.026	0.301631034	Leuk	MFHAS1
CYCS	8.33E-06	0.225103398	0.381	0.145	0.304743306	Leuk	CYCS
LARS	8.48E-06	0.207954933	0.266	0.068	0.310311139	Leuk	LARS
TUBB4B	8.71E-06	0.265108079	0.261	0.068	0.318828215	Leuk	TUBB4B
MEA1	9.00E-06	0.197293087	0.326	0.111	0.329520409	Leuk	MEA1
SELENOH	9.13E-06	0.278512634	0.631	0.368	0.334292099	Leuk	SELENOH
CRNDE	9.24E-06	0.258639041	0.393	0.162	0.338289357	Leuk	CRNDE
KIF22	9.99E-06	0.218683319	0.267	0.077	0.365544421	Leuk	KIF22
WDR34	1.06E-05	0.207668565	0.187	0.026	0.389609556	Leuk	WDR34
AZIN1	1.08E-05	0.224294284	0.26	0.068	0.394855427	Leuk	AZIN1

PSMA4	1.10E-05	0.18322561	0.374	0.145	0.401016248	Leuk	PSMA4
CLSPN	1.12E-05	0.213942667	0.17	0.017	0.409850521	Leuk	CLSPN
MT-ND6	1.14E-05	0.228649012	0.331	0.12	0.416871938	Leuk	MT-ND6
WDR1	1.17E-05	0.18619411	0.273	0.077	0.429451396	Leuk	WDR1
UBE2E3	1.20E-05	0.195477111	0.175	0.017	0.439463306	Leuk	UBE2E3
PRKDC	1.21E-05	0.242404027	0.325	0.12	0.441784382	Leuk	PRKDC
NDUFS6	1.24E-05	0.227371153	0.444	0.197	0.454713689	Leuk	NDUFS6
ATP5PO	1.26E-05	0.290631778	0.639	0.385	0.460774853	Leuk	ATP5PO
STAM	1.30E-05	0.216505039	0.32	0.111	0.47411157	Leuk	STAM
GINS2	1.32E-05	0.214770405	0.17	0.017	0.48486048	Leuk	GINS2
SLC25A3	1.38E-05	0.187193988	0.641	0.368	0.506500228	Leuk	SLC25A3
SNRPB	1.39E-05	0.271144738	0.609	0.342	0.510521192	Leuk	SNRPB
HEBP2	1.53E-05	0.220168136	0.225	0.051	0.559316613	Leuk	HEBP2
PSMD14	1.55E-05	0.184530908	0.201	0.034	0.567710815	Leuk	PSMD14
PCBP1	1.59E-05	0.231461435	0.523	0.256	0.580629699	Leuk	PCBP1
CCT8	1.65E-05	0.198822236	0.403	0.171	0.602578191	Leuk	CCT8
RPA3	1.66E-05	0.169680481	0.307	0.103	0.608890513	Leuk	RPA3
FEN1	1.69E-05	0.180207287	0.184	0.026	0.617565447	Leuk	FEN1
ATP5IF1	1.71E-05	0.228855236	0.512	0.248	0.624611628	Leuk	ATP5IF1
ATP5MC1	1.77E-05	0.215508383	0.314	0.111	0.648284904	Leuk	ATP5MC1
TRBV20-1	1.88E-05	0.279965283	0.261	0.077	0.686439195	Leuk	TRBV20-1
MZT2B	1.90E-05	0.231303774	0.591	0.299	0.69377197	Leuk	MZT2B
C11orf58	1.90E-05	0.199563949	0.487	0.231	0.694853199	Leuk	C11orf58
MCM5	1.93E-05	0.185446242	0.251	0.068	0.707572748	Leuk	MCM5
CCT7	1.99E-05	0.210416584	0.403	0.179	0.727139818	Leuk	CCT7
CENPU	2.07E-05	0.194849901	0.373	0.145	0.758996054	Leuk	CENPU
CACYBP	2.08E-05	0.203097554	0.29	0.094	0.762939774	Leuk	CACYBP
CCS	2.16E-05	0.192102362	0.282	0.085	0.789416027	Leuk	CCS
NDUFB2	2.18E-05	0.216880227	0.452	0.214	0.796489078	Leuk	NDUFB2
TMSB15A	2.41E-05	0.233641257	0.194	0.034	0.883009474	Leuk	TMSB15A
ROCK1	2.43E-05	0.169751125	0.34	0.128	0.89063771	Leuk	ROCK1
TPGS2	2.50E-05	0.159799861	0.339	0.128	0.914803772	Leuk	TPGS2
HSBP1	2.61E-05	0.168378555	0.303	0.103	0.956429539	Leuk	HSBP1
PRDX1	2.62E-05	0.297681922	0.467	0.239	0.957638455	Leuk	PRDX1
RAB1B	2.66E-05	0.173720292	0.222	0.051	0.97239664	Leuk	RAB1B
TRA2B	2.87E-05	0.222135798	0.48	0.239	1	Leuk	TRA2B
BLOC1S1	2.88E-05	0.21490849	0.429	0.205	1	Leuk	BLOC1S1
CENPF	2.96E-05	0.311178351	0.173	0.026	1	Leuk	CENPF
MAL	3.00E-05	0.365402818	0.517	0.291	1	Leuk	MAL
PXMP2	3.05E-05	0.190535746	0.205	0.043	1	Leuk	PXMP2
SMC4	3.09E-05	0.248542608	0.334	0.128	1	Leuk	SMC4
NELFE	3.15E-05	0.158236507	0.177	0.026	1	Leuk	NELFE
DTYMK	3.16E-05	0.200449719	0.198	0.043	1	Leuk	DTYMK
UBE2I	3.17E-05	0.190457405	0.426	0.197	1	Leuk	UBE2I
IL2RG	3.20E-05	0.235831937	0.656	0.393	1	Leuk	IL2RG
SRSF3	3.35E-05	0.258581189	0.638	0.359	1	Leuk	SRSF3
PTPN6	3.42E-05	0.223012739	0.241	0.068	1	Leuk	PTPN6
ALDH1A2	3.44E-05	0.215899574	0.175	0.026	1	Leuk	ALDH1A2
INO80E	3.45E-05	0.17034608	0.216	0.051	1	Leuk	INO80E
DHFR	3.51E-05	0.201411067	0.172	0.026	1	Leuk	DHFR
PRDX6	3.55E-05	0.250468589	0.526	0.274	1	Leuk	PRDX6
SRSF9	3.60E-05	0.250847335	0.539	0.282	1	Leuk	SRSF9
PTGES3	3.62E-05	0.260527238	0.593	0.316	1	Leuk	PTGES3
AKR1A1	3.64E-05	0.207522194	0.301	0.111	1	Leuk	AKR1A1

PAICS	3.64E-05	0.168871244	0.206	0.043	1	Leuk	PAICS
EPHB6	3.70E-05	0.22189094	0.201	0.043	1	Leuk	EPHB6
PHGDH	3.90E-05	0.204652039	0.174	0.026	1	Leuk	PHGDH
DNAJB6	3.93E-05	0.215732833	0.284	0.094	1	Leuk	DNAJB6
ADRM1	4.04E-05	0.16828386	0.238	0.068	1	Leuk	ADRM1
HDAC2	4.07E-05	0.152327538	0.287	0.094	1	Leuk	HDAC2
PSMA3	4.10E-05	0.185122399	0.229	0.06	1	Leuk	PSMA3
TERF2IP	4.15E-05	0.183839628	0.428	0.188	1	Leuk	TERF2IP
ITM2A	4.19E-05	0.454407773	0.939	0.778	1	Leuk	ITM2A
CENPM	4.22E-05	0.182560267	0.153	0.017	1	Leuk	CENPM
PAFAH1B3	4.38E-05	0.183705811	0.388	0.162	1	Leuk	PAFAH1B3
CORO1A	4.51E-05	0.291525575	0.572	0.333	1	Leuk	CORO1A
BEX4	4.83E-05	0.197552636	0.273	0.094	1	Leuk	BEX4
ELP5	4.94E-05	0.150371529	0.185	0.034	1	Leuk	ELP5
CDK2AP1	4.95E-05	0.153618797	0.518	0.265	1	Leuk	CDK2AP1
SLC25A5	5.02E-05	0.267112384	0.778	0.504	1	Leuk	SLC25A5
CLTA	5.21E-05	0.125631441	0.256	0.077	1	Leuk	CLTA
PRDX2	5.33E-05	0.222317089	0.391	0.171	1	Leuk	PRDX2
EZH2	5.34E-05	0.165349229	0.185	0.034	1	Leuk	EZH2
CD3D	5.40E-05	0.253280267	0.877	0.65	1	Leuk	CD3D
MZT1	5.55E-05	0.165551718	0.153	0.017	1	Leuk	MZT1
MCM3	5.76E-05	0.198221657	0.224	0.06	1	Leuk	MCM3
DDX39A	5.78E-05	0.185492156	0.312	0.111	1	Leuk	DDX39A
SEPTIN1	5.82E-05	0.224023731	0.367	0.162	1	Leuk	SEPTIN1
UBE2L6	5.84E-05	0.174221327	0.364	0.154	1	Leuk	UBE2L6
HPGD	5.84E-05	0.35161382	0.386	0.188	1	Leuk	HPGD
GMNN	6.07E-05	0.150511456	0.155	0.017	1	Leuk	GMNN
NSD2	6.19E-05	0.175064945	0.209	0.051	1	Leuk	NSD2
NDUFS7	6.31E-05	0.204188566	0.34	0.137	1	Leuk	NDUFS7
SRP72	6.34E-05	0.158988032	0.348	0.137	1	Leuk	SRP72
SELENOW	6.36E-05	0.376491105	0.572	0.35	1	Leuk	SELENOW
USP1	6.43E-05	0.185614673	0.245	0.077	1	Leuk	USP1
PSMB6	6.49E-05	0.184041605	0.544	0.299	1	Leuk	PSMB6
TMEM160	6.51E-05	0.169662111	0.323	0.128	1	Leuk	TMEM160
RALBP1	6.51E-05	0.143106023	0.289	0.103	1	Leuk	RALBP1
POLR2G	6.59E-05	0.188256844	0.388	0.171	1	Leuk	POLR2G
BSG	6.59E-05	0.176795617	0.475	0.231	1	Leuk	BSG
DYNLL1	6.64E-05	0.2018429	0.452	0.222	1	Leuk	DYNLL1
PHPT1	6.66E-05	0.163622431	0.29	0.103	1	Leuk	PHPT1
COPS9	6.81E-05	0.178571942	0.292	0.103	1	Leuk	COPS9
HADH	6.83E-05	0.149739097	0.197	0.043	1	Leuk	HADH
AATF	6.87E-05	0.178587405	0.221	0.06	1	Leuk	AATF
LDHB	7.07E-05	0.230353726	0.869	0.641	1	Leuk	LDHB
YEATS4	7.27E-05	0.155398929	0.167	0.026	1	Leuk	YEATS4
FADS2	7.46E-05	0.171681206	0.181	0.034	1	Leuk	FADS2
HNRNPF	7.49E-05	0.229915927	0.385	0.179	1	Leuk	HNRNPF
HNRNPD	7.51E-05	0.273606654	0.623	0.368	1	Leuk	HNRNPD
VRK1	7.55E-05	0.166865817	0.163	0.026	1	Leuk	VRK1
MYBL2	7.73E-05	0.163123519	0.135	0.009	1	Leuk	MYBL2
S100A10	7.85E-05	0.194816693	0.209	0.051	1	Leuk	S100A10
RNPS1	7.88E-05	0.157543768	0.39	0.171	1	Leuk	RNPS1
ZWINT	7.89E-05	0.157052653	0.163	0.026	1	Leuk	ZWINT
PNRC2	8.02E-05	0.180404627	0.248	0.077	1	Leuk	PNRC2
KRT1	8.05E-05	0.152787202	0.166	0.026	1	Leuk	KRT1

SMARCB1	8.23E-05	0.200434501	0.33	0.137	1	Leuk	SMARCB1
PCNP	8.41E-05	0.133576842	0.25	0.077	1	Leuk	PCNP
NEDD8	8.46E-05	0.179806787	0.497	0.239	1	Leuk	NEDD8
TIMM13	8.49E-05	0.165040467	0.251	0.077	1	Leuk	TIMM13
PPA1	8.59E-05	0.16935892	0.271	0.094	1	Leuk	PPA1
AC000065.1	8.62E-05	0.188730628	0.193	0.043	1	Leuk	AC000065.1
MX1	8.68E-05	0.19401788	0.162	0.026	1	Leuk	MX1
XRCC6	8.90E-05	0.190184982	0.512	0.265	1	Leuk	XRCC6
RPL7	8.91E-05	0.287802843	0.899	0.667	1	Leuk	RPL7
CYB5R3	8.93E-05	0.155971156	0.223	0.06	1	Leuk	CYB5R3
FADS1	9.01E-05	0.190279188	0.23	0.068	1	Leuk	FADS1
STRAP	9.08E-05	0.179080668	0.386	0.171	1	Leuk	STRAP
UBL5	9.10E-05	0.239778962	0.607	0.333	1	Leuk	UBL5
TRAPPC5	9.19E-05	0.178069018	0.351	0.145	1	Leuk	TRAPPC5
CALM2	9.28E-05	0.399966819	0.672	0.47	1	Leuk	CALM2
PRMT1	9.32E-05	0.164363769	0.376	0.162	1	Leuk	PRMT1
LSM7	9.35E-05	0.277783776	0.652	0.393	1	Leuk	LSM7
TRIM28	9.41E-05	0.185196924	0.418	0.197	1	Leuk	TRIM28
KRT10	9.47E-05	0.220557449	0.457	0.231	1	Leuk	KRT10
HIST1H3B	9.83E-05	0.280583283	0.173	0.034	1	Leuk	HIST1H3B
ZNHIT1	9.87E-05	0.172094932	0.314	0.12	1	Leuk	ZNHIT1
PGAM1	0.000101445	0.135471326	0.3	0.111	1	Leuk	PGAM1
DCTN2	0.000102842	0.174870783	0.266	0.094	1	Leuk	DCTN2
SSRP1	0.000104602	0.190204262	0.277	0.103	1	Leuk	SSRP1
ARPC1B	0.000104955	0.17545066	0.393	0.188	1	Leuk	ARPC1B
PSMB10	0.000106003	0.137677431	0.32	0.128	1	Leuk	PSMB10
HSPE1	0.000112	0.1795945	0.332	0.137	1	Leuk	HSPE1
NUBP2	0.000112207	0.178473322	0.21	0.06	1	Leuk	NUBP2
POLR2E	0.000112542	0.178412969	0.364	0.162	1	Leuk	POLR2E
SKA2	0.000113447	0.135276394	0.161	0.026	1	Leuk	SKA2
PKM	0.000117464	0.180471143	0.407	0.188	1	Leuk	PKM
EIF5B	0.000118094	0.162053428	0.241	0.077	1	Leuk	EIF5B
NDUFB3	0.000119341	0.133702392	0.199	0.051	1	Leuk	NDUFB3
RPL37A	0.000122858	0.228256514	0.939	0.778	1	Leuk	RPL37A
PSMB9	0.000122927	0.157402744	0.297	0.12	1	Leuk	PSMB9
NRDC	0.000123863	0.147402173	0.291	0.111	1	Leuk	NRDC
FKBP3	0.00012492	0.152967631	0.302	0.111	1	Leuk	FKBP3
MRPL12	0.000128342	0.140123692	0.144	0.017	1	Leuk	MRPL12
SRP9	0.000128649	0.221103201	0.451	0.231	1	Leuk	SRP9
MAGED1	0.000130481	0.145463626	0.174	0.034	1	Leuk	MAGED1
HIST1H1B	0.00013148	0.344822179	0.194	0.051	1	Leuk	HIST1H1B
TOP2A	0.000131681	0.214527541	0.127	0.009	1	Leuk	TOP2A
NDUFAB1	0.00013422	0.167922467	0.28	0.103	1	Leuk	NDUFAB1
RRM2	0.000134568	0.205859084	0.14	0.017	1	Leuk	RRM2
HMGB3	0.000135906	0.157829004	0.212	0.06	1	Leuk	HMGB3
NUTF2	0.000136009	0.103675247	0.338	0.137	1	Leuk	NUTF2
EIF4A3	0.000137172	0.158068022	0.186	0.043	1	Leuk	EIF4A3
USP39	0.000138485	0.16329322	0.168	0.034	1	Leuk	USP39
RSL24D1	0.000141064	0.171983767	0.463	0.231	1	Leuk	RSL24D1
TMEM179B	0.000142176	0.133879367	0.143	0.017	1	Leuk	TMEM179B
YWHAB	0.000143207	0.248794287	0.552	0.308	1	Leuk	YWHAB
MAD2L1	0.000145784	0.157915594	0.168	0.034	1	Leuk	MAD2L1
EMC6	0.00014715	0.171321442	0.208	0.06	1	Leuk	EMC6
TPR	0.000147413	0.189124863	0.404	0.188	1	Leuk	TPR

FDFT1	0.000148677	0.194984598	0.36	0.162	1	Leuk	FDFT1
TAF15	0.00015048	0.130949291	0.32	0.128	1	Leuk	TAF15
HIGD2A	0.000150948	0.193117742	0.411	0.205	1	Leuk	HIGD2A
PDCD7	0.000153197	0.142242397	0.157	0.026	1	Leuk	PDCD7
CD320	0.000153413	0.141910834	0.141	0.017	1	Leuk	CD320
PFKP	0.000157062	0.146797286	0.156	0.026	1	Leuk	PFKP
BEX2	0.000157848	0.180824491	0.168	0.034	1	Leuk	BEX2
TBCB	0.000158115	0.165966102	0.288	0.111	1	Leuk	TBCB
CCDC88A	0.000158488	0.161708192	0.296	0.12	1	Leuk	CCDC88A
RBMX	0.000159702	0.157591147	0.518	0.274	1	Leuk	RBMX
AKR1C3	0.000160931	0.163955084	0.278	0.103	1	Leuk	AKR1C3
ARL6IP4	0.000160977	0.13801514	0.552	0.291	1	Leuk	ARL6IP4
BRCA1	0.000161143	0.137695577	0.126	0.009	1	Leuk	BRCA1
SRSF10	0.000161203	0.20777054	0.426	0.214	1	Leuk	SRSF10
RAB10	0.000162391	0.151098175	0.199	0.051	1	Leuk	RAB10
SUMO3	0.000164583	0.19603482	0.436	0.214	1	Leuk	SUMO3
CCDC167	0.000164691	0.155060823	0.168	0.034	1	Leuk	CCDC167
SYCP2	0.000166403	0.159803608	0.14	0.017	1	Leuk	SYCP2
EXOSC8	0.000168775	0.161136748	0.167	0.034	1	Leuk	EXOSC8
RPL35	0.000170625	0.234461484	0.957	0.821	1	Leuk	RPL35
ANP32B	0.00017198	0.302279513	0.682	0.47	1	Leuk	ANP32B
NUCKS1	0.000175758	0.283904446	0.505	0.282	1	Leuk	NUCKS1
CDCA7	0.000188924	0.158255243	0.248	0.085	1	Leuk	CDCA7
SLIRP	0.000191222	0.103356893	0.24	0.077	1	Leuk	SLIRP
NGDN	0.000192016	0.146522167	0.165	0.034	1	Leuk	NGDN
HPRT1	0.000192481	0.141556275	0.196	0.051	1	Leuk	HPRT1
TCEAL9	0.000195668	0.14389575	0.138	0.017	1	Leuk	TCEAL9
ATP6V1D	0.000201036	0.150403809	0.122	0.009	1	Leuk	ATP6V1D
PDIA6	0.000201544	0.198046191	0.312	0.137	1	Leuk	PDIA6
FKBP1A	0.000201896	0.227973752	0.4	0.197	1	Leuk	FKBP1A
HERPUD1	0.000204153	0.303923678	0.522	0.291	1	Leuk	HERPUD1
TMEM208	0.000210584	0.153285147	0.151	0.026	1	Leuk	TMEM208
MKI67	0.00021322	0.237363659	0.146	0.026	1	Leuk	MKI67
DNAJC8	0.000213465	0.167185965	0.351	0.154	1	Leuk	DNAJC8
PRMT7	0.000214864	0.181410579	0.296	0.12	1	Leuk	PRMT7
SMC2	0.000217157	0.106567427	0.232	0.077	1	Leuk	SMC2
CTBP1	0.000217745	0.151030295	0.235	0.077	1	Leuk	CTBP1
MAZ	0.000228448	0.125758967	0.511	0.265	1	Leuk	MAZ
BAG1	0.000230499	0.13022467	0.222	0.068	1	Leuk	BAG1
DGUOK	0.000233987	0.156892834	0.256	0.094	1	Leuk	DGUOK
PKMYT1	0.000246964	0.140317319	0.117	0.009	1	Leuk	PKMYT1
NDUFA11	0.000247507	0.221784032	0.656	0.393	1	Leuk	NDUFA11
PPM1G	0.000248233	0.167048791	0.351	0.154	1	Leuk	PPM1G
NOP58	0.000248815	0.165420792	0.333	0.145	1	Leuk	NOP58
BTG3	0.000251916	0.104867881	0.195	0.051	1	Leuk	BTG3
DPY30	0.000252997	0.137291893	0.119	0.009	1	Leuk	DPY30
VCP	0.00025417	0.143843308	0.298	0.12	1	Leuk	VCP
SPINK2	0.000255145	0.173438718	0.288	0.12	1	Leuk	SPINK2
NDUFA8	0.000261993	0.149411516	0.161	0.034	1	Leuk	NDUFA8
NDUFAF3	0.00026208	0.142138651	0.22	0.068	1	Leuk	NDUFAF3
LCK	0.000263769	0.231418478	0.488	0.265	1	Leuk	LCK
SLC3A2	0.00026669	0.183500513	0.361	0.171	1	Leuk	SLC3A2
RPS27L	0.000271345	0.129268993	0.343	0.154	1	Leuk	RPS27L
UBR7	0.000275498	0.124315018	0.102	0	1	Leuk	UBR7

HIRIP3	0.000277633	0.112763703	0.119	0.009	1	Leuk	HIRIP3
SPCS2	0.000278889	0.138992717	0.314	0.128	1	Leuk	SPCS2
CENPN	0.000284618	0.129008392	0.102	0	1	Leuk	CENPN
EIF6	0.000284673	0.156292196	0.242	0.085	1	Leuk	EIF6
BRD7	0.0002874	0.163972128	0.354	0.162	1	Leuk	BRD7
PEBP1	0.00028798	0.138599257	0.447	0.231	1	Leuk	PEBP1
PPDPF	0.000288958	0.251661513	0.872	0.709	1	Leuk	PPDPF
GUK1	0.000291097	0.21464021	0.593	0.35	1	Leuk	GUK1
HSPB11	0.000293749	0.148987554	0.294	0.12	1	Leuk	HSPB11
MYO7B	0.000296708	0.185534313	0.295	0.12	1	Leuk	MYO7B
NR4A1	0.000296794	0.200065431	0.116	0.009	1	Leuk	NR4A1
THOC3	0.000299818	0.131646425	0.162	0.034	1	Leuk	THOC3
IGFBP2	0.000308779	0.154360267	0.146	0.026	1	Leuk	IGFBP2
TSN	0.000310097	0.159139904	0.171	0.043	1	Leuk	TSN
MCM2	0.00031337	0.144297462	0.144	0.026	1	Leuk	MCM2
SPON2	0.000315344	0.185036474	0.228	0.077	1	Leuk	SPON2
DDAH2	0.000316522	0.184569829	0.533	0.299	1	Leuk	DDAH2
ARL4C	0.000319725	0.163030478	0.381	0.188	1	Leuk	ARL4C
IAH1	0.000319883	0.118014999	0.159	0.034	1	Leuk	IAH1
PHB2	0.000320695	0.127193082	0.481	0.239	1	Leuk	PHB2
AHCY	0.000335193	0.144036309	0.199	0.06	1	Leuk	AHCY
HINT2	0.000336917	0.111669314	0.233	0.077	1	Leuk	HINT2
CD34	0.000342533	0.153596689	0.115	0.009	1	Leuk	CD34
CENPW	0.000346907	0.13752676	0.174	0.043	1	Leuk	CENPW
CCT3	0.000348135	0.128408194	0.357	0.162	1	Leuk	CCT3
CBX1	0.000349427	0.132807355	0.227	0.077	1	Leuk	CBX1
LSM5	0.000349548	0.151487665	0.324	0.145	1	Leuk	LSM5
TMCO1	0.000356573	0.139456495	0.13	0.017	1	Leuk	TMCO1
TMX1	0.000360178	0.132455657	0.145	0.026	1	Leuk	TMX1
TIMM17B	0.000361085	0.148518459	0.252	0.094	1	Leuk	TIMM17B
YWHAE	0.000364181	0.180488898	0.42	0.222	1	Leuk	YWHAE
SUPT16H	0.000369238	0.114434778	0.271	0.103	1	Leuk	SUPT16H
ACO2	0.000371259	0.115027074	0.158	0.034	1	Leuk	ACO2
UQCRQ	0.000381063	0.199902578	0.501	0.282	1	Leuk	UQCRQ
C4orf3	0.000383473	0.145796721	0.319	0.137	1	Leuk	C4orf3
GOLGA7	0.000384757	0.145288237	0.144	0.026	1	Leuk	GOLGA7
GABARAP	0.00038831	0.219772739	0.641	0.385	1	Leuk	GABARAP
MCM4	0.000390134	0.149260791	0.156	0.034	1	Leuk	MCM4
NAA38	0.000390385	0.135333409	0.282	0.111	1	Leuk	NAA38
NET1	0.000398457	0.158412691	0.17	0.043	1	Leuk	NET1
ZNRF1	0.000398726	0.127139609	0.381	0.179	1	Leuk	ZNRF1
TSPO	0.0004023	0.14039366	0.226	0.077	1	Leuk	TSPO
CMC2	0.000406666	0.109514721	0.228	0.077	1	Leuk	CMC2
ENY2	0.00041098	0.16331528	0.326	0.145	1	Leuk	ENY2
XRCC5	0.000417322	0.151494577	0.493	0.265	1	Leuk	XRCC5
MRPS21	0.000418604	0.124557279	0.268	0.111	1	Leuk	MRPS21
MARS	0.000422184	0.127756112	0.129	0.017	1	Leuk	MARS
HTATSF1	0.000423577	0.129862689	0.257	0.094	1	Leuk	HTATSF1
PDCD6	0.000427465	0.177959811	0.283	0.12	1	Leuk	PDCD6
DDB1	0.000430308	0.146979248	0.183	0.051	1	Leuk	DDB1
PRPF4B	0.000439887	0.111717353	0.349	0.162	1	Leuk	PRPF4B
CDKN2D	0.000444744	0.151330092	0.276	0.111	1	Leuk	CDKN2D
CDC45	0.000449503	0.105249947	0.112	0.009	1	Leuk	CDC45
RAD23A	0.000452903	0.130996392	0.327	0.145	1	Leuk	RAD23A

SMU1	0.000454496	0.132163576	0.154	0.034	1	Leuk	SMU1
SH2D1A	0.000464059	0.129405354	0.449	0.239	1	Leuk	SH2D1A
CDK4	0.000465138	0.162912518	0.267	0.111	1	Leuk	CDK4
SAC3D1	0.000467601	0.129371719	0.126	0.017	1	Leuk	SAC3D1
EPN1	0.000491322	0.121081441	0.127	0.017	1	Leuk	EPN1
HMGN1	0.00049166	0.173458267	0.705	0.444	1	Leuk	HMGN1
CCT4	0.000494198	0.160386601	0.423	0.214	1	Leuk	CCT4
TMEM147	0.000494679	0.142076679	0.233	0.085	1	Leuk	TMEM147
CHURC1	0.000500075	0.137027268	0.298	0.128	1	Leuk	CHURC1
C9orf16	0.000507767	0.224602648	0.686	0.419	1	Leuk	C9orf16
CLIP3	0.000510046	0.13860553	0.125	0.017	1	Leuk	CLIP3
RPS20	0.000518194	0.17272934	0.734	0.479	1	Leuk	RPS20
SRSF2	0.00052387	0.224921591	0.685	0.453	1	Leuk	SRSF2
NDUFS8	0.000528466	0.149109549	0.411	0.205	1	Leuk	NDUFS8
DHX15	0.000531001	0.138135275	0.181	0.051	1	Leuk	DHX15
ACAT2	0.000533499	0.170509251	0.191	0.06	1	Leuk	ACAT2
SLBP	0.000536045	0.140638721	0.192	0.06	1	Leuk	SLBP
MT-ATP6	0.000547639	0.221299089	0.961	0.855	1	Leuk	MT-ATP6
PSMC3	0.000551478	0.156542339	0.304	0.137	1	Leuk	PSMC3
PDXP	0.000552755	0.110089564	0.141	0.026	1	Leuk	PDXP
HNRNPDL	0.000560894	0.240368612	0.735	0.479	1	Leuk	HNRNPDL
PKN2	0.000570308	0.106577316	0.196	0.06	1	Leuk	PKN2
SMS	0.000581354	0.144306389	0.206	0.068	1	Leuk	SMS
FAM177A1	0.000585897	0.151308622	0.151	0.034	1	Leuk	FAM177A1
VAT1	0.000586608	0.227405839	0.414	0.222	1	Leuk	VAT1
CD5	0.000589051	0.126039807	0.108	0.009	1	Leuk	CD5
UBE2C	0.000590326	0.181303157	0.106	0.009	1	Leuk	UBE2C
MCM6	0.000591756	0.141102173	0.18	0.051	1	Leuk	MCM6
HM13	0.000600708	0.158325327	0.35	0.162	1	Leuk	HM13
COPS3	0.000605505	0.119701112	0.258	0.103	1	Leuk	COPS3
LSM6	0.00060974	0.133188954	0.137	0.026	1	Leuk	LSM6
SINHCAF	0.000619184	0.141740084	0.254	0.103	1	Leuk	SINHCAF
SRSF7	0.000623541	0.183212932	0.516	0.282	1	Leuk	SRSF7
DANCR	0.000624538	0.153256929	0.135	0.026	1	Leuk	DANCR
ATAD5	0.000627957	0.13395016	0.137	0.026	1	Leuk	ATAD5
ANAPC11	0.000628105	0.205280027	0.488	0.274	1	Leuk	ANAPC11
CSNK1G2	0.000635875	0.133256628	0.165	0.043	1	Leuk	CSNK1G2
AHSA1	0.000639598	0.11826737	0.138	0.026	1	Leuk	AHSA1
ELOC	0.00064187	0.114902246	0.273	0.111	1	Leuk	ELOC
G3BP2	0.000650316	0.100838005	0.247	0.094	1	Leuk	G3BP2
CPSF6	0.000652014	0.143085788	0.256	0.103	1	Leuk	CPSF6
TMEM106C	0.000653474	0.141620507	0.187	0.06	1	Leuk	TMEM106C
TAGLN2	0.000679999	0.215342445	0.68	0.453	1	Leuk	TAGLN2
BIRC5	0.000684409	0.139166442	0.105	0.009	1	Leuk	BIRC5
MTDH	0.000695671	0.167413392	0.508	0.291	1	Leuk	MTDH
ARPC2	0.000734261	0.193272794	0.646	0.419	1	Leuk	ARPC2
PSMC4	0.000736963	0.114418079	0.177	0.051	1	Leuk	PSMC4
CENPV	0.00073794	0.123693857	0.203	0.068	1	Leuk	CENPV
LRRC59	0.000740772	0.106091102	0.164	0.043	1	Leuk	LRRC59
RTN4	0.000752928	0.166575107	0.571	0.333	1	Leuk	RTN4
HCFC1	0.000754432	0.106276836	0.151	0.034	1	Leuk	HCFC1
WDR83OS	0.000758619	0.127214144	0.425	0.222	1	Leuk	WDR83OS
MRPS16	0.0007607	0.125823592	0.187	0.06	1	Leuk	MRPS16
NFYC	0.000760996	0.118901558	0.12	0.017	1	Leuk	NFYC

NFIC	0.000762297	0.108721449	0.136	0.026	1	Leuk	NFIC
LAGE3	0.000765433	0.14753963	0.26	0.111	1	Leuk	LAGE3
TMEM109	0.000775247	0.14416113	0.186	0.06	1	Leuk	TMEM109
MRPL16	0.000781304	0.102334838	0.253	0.103	1	Leuk	MRPL16
NOL7	0.000782891	0.110259894	0.301	0.128	1	Leuk	NOL7
HAUS1	0.000797268	0.118930823	0.133	0.026	1	Leuk	HAUS1
AURKAIP1	0.000799599	0.137110936	0.316	0.145	1	Leuk	AURKAIP1
TPX2	0.000799719	0.176244576	0.156	0.043	1	Leuk	TPX2
HIST1H1C	0.000801504	0.178177337	0.464	0.256	1	Leuk	HIST1H1C
UBE2D2	0.000807899	0.197178435	0.454	0.256	1	Leuk	UBE2D2
PPIB	0.000819332	0.239783202	0.754	0.538	1	Leuk	PPIB
RPL18A	0.000839009	0.227624083	0.986	0.915	1	Leuk	RPL18A
NUDT21	0.000843349	0.113266648	0.229	0.085	1	Leuk	NUDT21
PMS2	0.000873126	0.130321439	0.118	0.017	1	Leuk	PMS2
FAM111A	0.000876725	0.124519676	0.201	0.068	1	Leuk	FAM111A
PSMA2	0.000881265	0.151289584	0.521	0.291	1	Leuk	PSMA2
SRRM1	0.00088421	0.11864641	0.552	0.333	1	Leuk	SRRM1
DDX24	0.000885869	0.158761422	0.334	0.162	1	Leuk	DDX24
NUP93	0.000903721	0.108483214	0.118	0.017	1	Leuk	NUP93
LAT	0.000916553	0.146850565	0.474	0.274	1	Leuk	LAT
RNASEH2A	0.000917071	0.11493429	0.146	0.034	1	Leuk	RNASEH2A
SLC43A3	0.000930672	0.103113905	0.103	0.009	1	Leuk	SLC43A3
MYL6B	0.00093175	0.143184259	0.372	0.188	1	Leuk	MYL6B
ANAPC15	0.000935531	0.13778976	0.223	0.085	1	Leuk	ANAPC15
MLF2	0.000947425	0.128299733	0.44	0.239	1	Leuk	MLF2
MFSD14C	0.000951018	0.111673392	0.118	0.017	1	Leuk	MFSD14C
UQCRC1	0.000958479	0.114689228	0.344	0.162	1	Leuk	UQCRC1
PSMB3	0.000965052	0.165273583	0.511	0.299	1	Leuk	PSMB3
WASF2	0.000967962	0.120180513	0.456	0.239	1	Leuk	WASF2
LGALS9	0.00098337	0.112996443	0.278	0.12	1	Leuk	LGALS9
ZCRB1	0.000990702	0.134246656	0.26	0.111	1	Leuk	ZCRB1
TK1	0.000998495	0.12346104	0.116	0.017	1	Leuk	TK1
NINJ2	0.000999854	0.14270997	0.143	0.034	1	Leuk	NINJ2
IK	0.001000134	0.14708329	0.293	0.137	1	Leuk	IK
HNRNPU	0.00100052	0.184701952	0.642	0.393	1	Leuk	HNRNPU
DENND2D	0.001011906	0.127045352	0.159	0.043	1	Leuk	DENND2D
ESD	0.001012212	0.11501085	0.284	0.12	1	Leuk	ESD
PALM2-AKAP2	0.001015823	0.175535438	0.547	0.325	1	Leuk	PALM2-AKAP2
ARHGDIA	0.001051706	0.145692266	0.349	0.171	1	Leuk	ARHGDIA
IGFBP5	0.001061956	0.181270769	0.209	0.077	1	Leuk	IGFBP5
BUD31	0.001068904	0.109445407	0.28	0.12	1	Leuk	BUD31
BID	0.001081788	0.103833484	0.159	0.043	1	Leuk	BID
PA2G4	0.001091111	0.161555221	0.43	0.231	1	Leuk	PA2G4
PSMD1	0.00109829	0.137396077	0.181	0.06	1	Leuk	PSMD1
CKAP2	0.001116028	0.123410436	0.13	0.026	1	Leuk	CKAP2
ATAD2	0.001119248	0.105093142	0.172	0.051	1	Leuk	ATAD2
C19orf48	0.001148956	0.128194075	0.245	0.103	1	Leuk	C19orf48
MSRB2	0.001156463	0.111643449	0.129	0.026	1	Leuk	MSRB2
ENOSF1	0.001158555	0.159320268	0.204	0.077	1	Leuk	ENOSF1
HSPA9	0.001185149	0.102645512	0.303	0.137	1	Leuk	HSPA9
SIGIRR	0.001198299	0.160524038	0.329	0.162	1	Leuk	SIGIRR
TCF3	0.001200332	0.135381602	0.205	0.077	1	Leuk	TCF3
NAA20	0.001200597	0.104583024	0.129	0.026	1	Leuk	NAA20
GLO1	0.00121804	0.119743909	0.221	0.085	1	Leuk	GLO1

SSNA1	0.001241048	0.11887063	0.299	0.137	1	Leuk	SSNA1
UBE2S	0.001248152	0.234163185	0.424	0.248	1	Leuk	UBE2S
COX7C	0.001250399	0.211901793	0.797	0.556	1	Leuk	COX7C
ATP5F1C	0.001258088	0.112707826	0.5	0.282	1	Leuk	ATP5F1C
NSMCE1	0.001266313	0.116332092	0.259	0.111	1	Leuk	NSMCE1
CAPZB	0.001269816	0.101263426	0.461	0.256	1	Leuk	CAPZB
SMARCA4	0.001279767	0.104606769	0.282	0.128	1	Leuk	SMARCA4
G3BP1	0.001285191	0.132479588	0.22	0.085	1	Leuk	G3BP1
POLR2K	0.00128928	0.119523843	0.154	0.043	1	Leuk	POLR2K
PRKAB1	0.001300624	0.143115832	0.139	0.034	1	Leuk	PRKAB1
EIF4EBP1	0.001303425	0.120188361	0.182	0.06	1	Leuk	EIF4EBP1
RPSA	0.001312484	0.205309984	0.972	0.88	1	Leuk	RPSA
TTF2	0.00132515	0.100129512	0.113	0.017	1	Leuk	TTF2
CENPH	0.001326137	0.103800677	0.112	0.017	1	Leuk	CENPH
ACAA2	0.001326211	0.111698096	0.141	0.034	1	Leuk	ACAA2
HELLS	0.001343339	0.10526441	0.14	0.034	1	Leuk	HELLS
RHOA	0.001346276	0.196164324	0.777	0.538	1	Leuk	RHOA
BRD2	0.001360812	0.120965224	0.337	0.162	1	Leuk	BRD2
BAZ1B	0.001372348	0.106790435	0.236	0.094	1	Leuk	BAZ1B
TXNDC17	0.001383964	0.111328395	0.169	0.051	1	Leuk	TXNDC17
AC090152.1	0.001387414	0.113808126	0.269	0.12	1	Leuk	AC090152.1
GNB1	0.001398223	0.146958253	0.34	0.171	1	Leuk	GNB1
SNX1	0.00140241	0.104412674	0.127	0.026	1	Leuk	SNX1
ARFGAP3	0.001403061	0.108330299	0.196	0.068	1	Leuk	ARFGAP3
HSPBP1	0.001418135	0.106527445	0.127	0.026	1	Leuk	HSPBP1
POLR2D	0.001428517	0.123737617	0.139	0.034	1	Leuk	POLR2D
MT2A	0.001437792	0.191735192	0.267	0.128	1	Leuk	MT2A
EDF1	0.00145086	0.11773346	0.65	0.402	1	Leuk	EDF1
COTL1	0.001477256	0.13864154	0.23	0.094	1	Leuk	COTL1
ORMDL1	0.001487872	0.109434442	0.169	0.051	1	Leuk	ORMDL1
ACD	0.001522591	0.10293262	0.126	0.026	1	Leuk	ACD
GTF2A2	0.001526718	0.138783299	0.33	0.162	1	Leuk	GTF2A2
HSPA4	0.001530378	0.112401093	0.153	0.043	1	Leuk	HSPA4
PPP1R35	0.001531372	0.123587177	0.205	0.077	1	Leuk	PPP1R35
CCDC34	0.001532711	0.108374517	0.111	0.017	1	Leuk	CCDC34
BCL11A	0.001541713	0.120942167	0.152	0.043	1	Leuk	BCL11A
RTL6	0.001561865	0.100134579	0.126	0.026	1	Leuk	RTL6
COMMD7	0.001587021	0.100375434	0.254	0.111	1	Leuk	COMMD7
SMIM26	0.001641247	0.13019735	0.292	0.137	1	Leuk	SMIM26
IP6K2	0.001697851	0.114419526	0.23	0.094	1	Leuk	IP6K2
PRKAR1B	0.001712839	0.117947849	0.217	0.085	1	Leuk	PRKAR1B
MAF1	0.001717412	0.108945967	0.254	0.111	1	Leuk	MAF1
CYSTM1	0.001724131	0.100300149	0.109	0.017	1	Leuk	CYSTM1
MTHFD2L	0.001730932	0.139196144	0.175	0.06	1	Leuk	MTHFD2L
VIPR2	0.001745725	0.108291748	0.11	0.017	1	Leuk	VIPR2
SIT1	0.001756033	0.120075872	0.108	0.017	1	Leuk	SIT1
PPIH	0.001764512	0.102253791	0.205	0.077	1	Leuk	PPIH
KPNA2	0.001768826	0.108854751	0.215	0.085	1	Leuk	KPNA2
MAP4K4	0.001797994	0.10800264	0.225	0.094	1	Leuk	MAP4K4
COX5A	0.001825878	0.143097863	0.579	0.359	1	Leuk	COX5A
ADH5	0.001829518	0.106591977	0.242	0.103	1	Leuk	ADH5
PHC2	0.001842847	0.104937283	0.137	0.034	1	Leuk	PHC2
VIM	0.001846061	0.249736569	0.702	0.496	1	Leuk	VIM
PGK1	0.001862651	0.193532436	0.583	0.359	1	Leuk	PGK1

ADK	0.001899604	0.104745756	0.148	0.043	1	Leuk	ADK
CUL3	0.001899875	0.130418563	0.189	0.068	1	Leuk	CUL3
PFDN2	0.001912409	0.119125704	0.315	0.154	1	Leuk	PFDN2
MTHFD2	0.001914177	0.108883069	0.123	0.026	1	Leuk	MTHFD2
BRWD1	0.001972057	0.109031712	0.203	0.077	1	Leuk	BRWD1
TAF9	0.001979216	0.125657125	0.224	0.094	1	Leuk	TAF9
ZNF423	0.001993417	0.109727284	0.107	0.017	1	Leuk	ZNF423
TMEM126A	0.00199405	0.110218878	0.106	0.017	1	Leuk	TMEM126A
PSMD4	0.002012678	0.137437671	0.348	0.179	1	Leuk	PSMD4
MZT2A	0.002031465	0.106774156	0.214	0.085	1	Leuk	MZT2A
VDAC1	0.002040381	0.123162902	0.283	0.137	1	Leuk	VDAC1
NUCB2	0.002051732	0.115641897	0.899	0.752	1	Leuk	NUCB2
FUS	0.002081012	0.223256612	0.712	0.513	1	Leuk	FUS
TMEM141	0.002192199	0.124529558	0.13	0.034	1	Leuk	TMEM141
MRPL2	0.002209971	0.122667544	0.131	0.034	1	Leuk	MRPL2
UBALD2	0.002214817	0.103159037	0.415	0.231	1	Leuk	UBALD2
HARS	0.002332671	0.112274436	0.118	0.026	1	Leuk	HARS
SDCBP	0.002395601	0.119125039	0.197	0.077	1	Leuk	SDCBP
SYNC	0.002401684	0.101968523	0.226	0.094	1	Leuk	SYNC
UBA1	0.002427449	0.118968508	0.17	0.06	1	Leuk	UBA1
PSMB2	0.002445488	0.129520236	0.378	0.205	1	Leuk	PSMB2
RFC1	0.002459251	0.104804432	0.232	0.103	1	Leuk	RFC1
CCND3	0.002461672	0.176627872	0.76	0.53	1	Leuk	CCND3
C1orf122	0.00250552	0.113293387	0.158	0.051	1	Leuk	C1orf122
CYC1	0.002519304	0.114845276	0.271	0.128	1	Leuk	CYC1
HNRNPUL2	0.002519702	0.113260067	0.261	0.12	1	Leuk	HNRNPUL2
MCUR1	0.002564785	0.103898299	0.171	0.06	1	Leuk	MCUR1
MRPS15	0.002622446	0.12755343	0.257	0.12	1	Leuk	MRPS15
PLPP5	0.002630276	0.108081501	0.144	0.043	1	Leuk	PLPP5
HAX1	0.002652383	0.11595791	0.195	0.077	1	Leuk	HAX1
HP1BP3	0.002664354	0.137984204	0.608	0.368	1	Leuk	HP1BP3
ARL2	0.002678078	0.103735004	0.144	0.043	1	Leuk	ARL2
CRIP1	0.002706248	0.24154472	0.377	0.222	1	Leuk	CRIP1
TBCA	0.002728555	0.111378065	0.427	0.239	1	Leuk	TBCA
AGPAT2	0.002761297	0.113489354	0.117	0.026	1	Leuk	AGPAT2
SAP18	0.002776082	0.115652968	0.597	0.376	1	Leuk	SAP18
OST4	0.002822686	0.159356964	0.631	0.402	1	Leuk	OST4
SRM	0.002841912	0.135896134	0.254	0.12	1	Leuk	SRM
GABARAPL2	0.002844902	0.114186698	0.299	0.145	1	Leuk	GABARAPL2
TTC32	0.002846943	0.100547566	0.102	0.017	1	Leuk	TTC32
PGD	0.002892576	0.130719133	0.331	0.171	1	Leuk	PGD
WNK1	0.002920444	0.110925723	0.316	0.162	1	Leuk	WNK1
BRMS1	0.002934931	0.100249043	0.13	0.034	1	Leuk	BRMS1
SELENOF	0.002945729	0.11356903	0.336	0.179	1	Leuk	SELENOF
NDUFA12	0.002977381	0.11404496	0.301	0.154	1	Leuk	NDUFA12
GPX4	0.003051421	0.165545371	0.616	0.393	1	Leuk	GPX4
COPE	0.00305394	0.166199804	0.518	0.325	1	Leuk	COPE
RBCK1	0.003104915	0.106026768	0.235	0.103	1	Leuk	RBCK1
B3GNTL1	0.003143619	0.105875803	0.129	0.034	1	Leuk	B3GNTL1
KTN1	0.00314424	0.130906514	0.451	0.265	1	Leuk	KTN1
GNAI2	0.003152849	0.128020656	0.495	0.308	1	Leuk	GNAI2
H2AFV	0.003169403	0.116544182	0.654	0.436	1	Leuk	H2AFV
SERBP1	0.003180385	0.111627201	0.544	0.325	1	Leuk	SERBP1
HDAC1	0.003188	0.109810461	0.282	0.137	1	Leuk	HDAC1

RSF1	0.003271973	0.132904777	0.292	0.145	1	Leuk	RSF1
GNG5	0.003294174	0.130439396	0.544	0.333	1	Leuk	GNG5
ILF3	0.003428529	0.113255158	0.432	0.248	1	Leuk	ILF3
POLR2I	0.003444414	0.116668888	0.255	0.12	1	Leuk	POLR2I
HACD1	0.003465636	0.120752174	0.362	0.205	1	Leuk	HACD1
MIEN1	0.003484791	0.103598592	0.258	0.12	1	Leuk	MIEN1
ANP32E	0.003522377	0.114475001	0.282	0.137	1	Leuk	ANP32E
HIST1H1D	0.003524625	0.226447305	0.648	0.47	1	Leuk	HIST1H1D
ABHD17A	0.003527687	0.11639149	0.226	0.103	1	Leuk	ABHD17A
SUMO2	0.003622619	0.211069055	0.802	0.624	1	Leuk	SUMO2
SVIP	0.003670606	0.104198582	0.138	0.043	1	Leuk	SVIP
SNRPN	0.003707988	0.107017642	0.126	0.034	1	Leuk	SNRPN
DYNLL2	0.003713028	0.11057963	0.139	0.043	1	Leuk	DYNLL2
TPM3	0.003722383	0.233308532	0.733	0.547	1	Leuk	TPM3
URM1	0.003730823	0.119743719	0.211	0.094	1	Leuk	URM1
LSM2	0.003745715	0.1035536	0.422	0.239	1	Leuk	LSM2
YWHAQ	0.003745747	0.191044834	0.575	0.368	1	Leuk	YWHAQ
DDX18	0.003754805	0.109103692	0.215	0.094	1	Leuk	DDX18
TMEM167A	0.003765281	0.103914085	0.111	0.026	1	Leuk	TMEM167A
RNF168	0.003860292	0.134870617	0.29	0.145	1	Leuk	RNF168
RNASEH2B	0.003897656	0.14549093	0.517	0.308	1	Leuk	RNASEH2B
ATP5MC3	0.003950068	0.201114117	0.707	0.504	1	Leuk	ATP5MC3
RBM38	0.00400762	0.100655559	0.126	0.034	1	Leuk	RBM38
TCERG1	0.004159652	0.120139676	0.235	0.111	1	Leuk	TCERG1
DMTF1	0.004173507	0.119832731	0.124	0.034	1	Leuk	DMTF1
RHBDD2	0.004227169	0.123751511	0.135	0.043	1	Leuk	RHBDD2
FIS1	0.004228894	0.135922433	0.314	0.171	1	Leuk	FIS1
TOPORS	0.004232599	0.102858149	0.151	0.051	1	Leuk	TOPORS
LIMD2	0.004390989	0.159929954	0.533	0.333	1	Leuk	LIMD2
PIN1	0.004411362	0.116301065	0.387	0.214	1	Leuk	PIN1
RPL21	0.004512151	0.17514868	0.963	0.88	1	Leuk	RPL21
RAP1B	0.004513821	0.133342159	0.382	0.214	1	Leuk	RAP1B
RPS16	0.004746354	0.153048944	0.95	0.863	1	Leuk	RPS16
CD3G	0.004970723	0.109958848	0.555	0.342	1	Leuk	CD3G
RPL31	0.00498694	0.202290376	0.837	0.692	1	Leuk	RPL31
HMGA1	0.005182122	0.140635357	0.426	0.248	1	Leuk	HMGA1
ZNRD1	0.005337424	0.112290454	0.247	0.12	1	Leuk	ZNRD1
CUL4A	0.005434602	0.10476398	0.147	0.051	1	Leuk	CUL4A
SF3B6	0.005583447	0.118282708	0.43	0.248	1	Leuk	SF3B6
GNAS	0.005608464	0.177059915	0.886	0.709	1	Leuk	GNAS
RFC4	0.005729392	0.100001421	0.119	0.034	1	Leuk	RFC4
BCL7B	0.005884504	0.101340147	0.132	0.043	1	Leuk	BCL7B
CD82	0.005944378	0.15989369	0.454	0.274	1	Leuk	CD82
TRMT112	0.005970608	0.161954166	0.483	0.308	1	Leuk	TRMT112
HMG20B	0.006048961	0.109729504	0.217	0.103	1	Leuk	HMG20B
HSP90AB1	0.006145719	0.168564509	0.851	0.675	1	Leuk	HSP90AB1
EIF2AK2	0.006204445	0.10613197	0.207	0.094	1	Leuk	EIF2AK2
WDR82	0.006278493	0.110358739	0.143	0.051	1	Leuk	WDR82
YARS	0.00631315	0.10279032	0.181	0.077	1	Leuk	YARS
HSD17B12	0.00642889	0.100250842	0.104	0.026	1	Leuk	HSD17B12
NDUFA13	0.006582494	0.148746927	0.76	0.547	1	Leuk	NDUFA13
UBE2L3	0.006589921	0.10585918	0.349	0.197	1	Leuk	UBE2L3
STT3B	0.00662739	0.13335939	0.594	0.393	1	Leuk	STT3B
NDUFS5	0.006941163	0.209505819	0.592	0.393	1	Leuk	NDUFS5

SNRPD1	0.00713616	0.126717794	0.408	0.239	1	Leuk	SNRPD1
RFXANK	0.007184302	0.10004757	0.204	0.094	1	Leuk	RFXANK
METTL26	0.007244529	0.100004334	0.306	0.171	1	Leuk	METTL26
UQCRH	0.007308396	0.126669497	0.611	0.41	1	Leuk	UQCRH
HNRNPA3	0.007372332	0.11515924	0.64	0.427	1	Leuk	HNRNPA3
LAPTM5	0.007575631	0.228817409	0.606	0.444	1	Leuk	LAPTM5
PSMC5	0.007887061	0.109978923	0.307	0.171	1	Leuk	PSMC5
SURF1	0.008107163	0.121608133	0.162	0.068	1	Leuk	SURF1
CLIC1	0.008327118	0.191506636	0.848	0.684	1	Leuk	CLIC1
UFM1	0.008839614	0.10598659	0.213	0.103	1	Leuk	UFM1
COX5B	0.008971017	0.121738176	0.605	0.376	1	Leuk	COX5B
UCP2	0.009080419	0.11388633	0.292	0.162	1	Leuk	UCP2
ISY1	0.009293941	0.100310792	0.162	0.068	1	Leuk	ISY1
HNRNPK	0.00931322	0.138657644	0.675	0.47	1	Leuk	HNRNPK
NCOR2	0.009941664	0.105225233	0.137	0.051	1	Leuk	NCOR2
