# Supplementary material for: High CD44 expression and enhanced E-selectin binding identified as biomarkers of chemoresistant leukemic cells in human T-ALL
Source: Leukemia. 2024 Nov 24;39(2):323–36. doi: 10.1038/s41375-024-02473-7 (PMC11794132; doi:10.1038/s41375-024-02473-7)
Supplement: Supplementary file 10 — Supplemental Table 9 [file 41375_2024_2473_MOESM10_ESM.pdf]

Common upregulated genes in Ki67neg CD44high leukemic cells in libraries (Figure 4g)

Common	gene
5 common elements in "Lib 1" and "Lib 2":	EIF1
	FTH1
	ARL4C
	SF1
	ST3GAL1
1 common element in "Lib 3" and "Lib 4":	TXNIP
2 common elements in "Lib 2" and "Lib 4":	TSC22D3
	BTG1
2 common elements in "Lib 1", "Lib 2" and "Lib 4":	JUND
	KLF2
16 common elements in "Lib 1" and "Lib 4":	KHDRBS3
	TPT1
	NFKB1
	KLF6
	H3F3B
	PNRC1
	FTL
	LINC01578
	JUNB
	CD69
	CD7
	GLS
	HSH2D
	SMAP2
	FOS
	NR3C1
1 common element in "Lib 1", "Lib 2", "Lib 3" and "Lib 4":	MALAT1
8 common elements in "Lib 1" and "Lib 3":	HLA-B
	LIMD2
	B2M
	HLA-E
	SH3BP5
	HLA-A
	LTB
	FCGRT
3 common elements in "Lib 1", "Lib 2" and "Lib 3":	EMP3
	HLA-C
	SH3BGRL3
