# Supplementary material for: High CD44 expression and enhanced E-selectin binding identified as biomarkers of chemoresistant leukemic cells in human T-ALL
Source: Leukemia. 2024 Nov 24;39(2):323–36. doi: 10.1038/s41375-024-02473-7 (PMC11794132; doi:10.1038/s41375-024-02473-7)
Supplement: Supplementary file 11 — Supplemental Table 10 [file 41375_2024_2473_MOESM11_ESM.pdf]

Common upregulated genes between Cluster 4 & Common 38 genes from 4 libraries scRNAseq (Figure 4h)

GENENAME
LTB
KLF2
EMP3
SH3BGRL3
TXNIP
SH3BP5
KLF6
MALAT1
ARL4C
FOS
HLA-E
B2M
PNRC1
LIMD2
HLA-B
TSC22D3
HLA-A
JUND
SF1
HLA-C
BTG1
EIF1
ST3GAL1
FCGRT
