# Supplementary material for: High CD44 expression and enhanced E-selectin binding identified as biomarkers of chemoresistant leukemic cells in human T-ALL
Source: Leukemia. 2024 Nov 24;39(2):323–36. doi: 10.1038/s41375-024-02473-7 (PMC11794132; doi:10.1038/s41375-024-02473-7)
Supplement: Supplementary file 12 — Supplemental Table 11 [file 41375_2024_2473_MOESM12_ESM.pdf]

upregulated genes in Ki67neg CD44high normal cells from Library 1 (Supplementary Figure 12a)

	p_val	avg_log2FC	pct.1	pct.2	p_val_adj	cluster	gene
RPL30	3.58E-19	0.554057204	1	1	1.31E-14	NO SIGNATURE NON LEUK	RPL30
RPL34	2.42E-18	0.507153978	1	1	8.86E-14	NO SIGNATURE NON LEUK	RPL34
RPS3	1.66E-17	0.596115452	1	0.996	6.07E-13	NO SIGNATURE NON LEUK	RPS3
RPS3A	4.13E-17	0.560476783	1	1	1.51E-12	NO SIGNATURE NON LEUK	RPS3A
RPS26	4.46E-17	0.547849551	1	1	1.63E-12	NO SIGNATURE NON LEUK	RPS26
RPS27A	2.71E-16	0.562893441	1	1	9.92E-12	NO SIGNATURE NON LEUK	RPS27A
RPS18	3.45E-16	0.526146287	1	1	1.26E-11	NO SIGNATURE NON LEUK	RPS18
RPS12	3.49E-16	0.512286313	1	1	1.28E-11	NO SIGNATURE NON LEUK	RPS12
RPS6	7.69E-16	0.534782051	1	1	2.82E-11	NO SIGNATURE NON LEUK	RPS6
RPSA	9.34E-16	0.593953672	1	0.965	3.42E-11	NO SIGNATURE NON LEUK	RPSA
RPL41	2.02E-15	0.435365627	1	1	7.39E-11	NO SIGNATURE NON LEUK	RPL41
RPS15A	2.48E-15	0.460335383	1	1	9.08E-11	NO SIGNATURE NON LEUK	RPS15A
RPL10	3.48E-15	0.383211961	1	1	1.27E-10	NO SIGNATURE NON LEUK	RPL10
RPS27	1.29E-14	0.538493809	1	1	4.73E-10	NO SIGNATURE NON LEUK	RPS27
RPL32	2.69E-14	0.463356768	0.99	1	9.85E-10	NO SIGNATURE NON LEUK	RPL32
RPL3	2.81E-14	0.52297543	0.995	0.996	1.03E-09	NO SIGNATURE NON LEUK	RPL3
RPS21	3.31E-14	0.541842453	0.969	0.992	1.21E-09	NO SIGNATURE NON LEUK	RPS21
RPS5	3.91E-14	0.554005349	0.984	0.984	1.43E-09	NO SIGNATURE NON LEUK	RPS5
RPL13	4.35E-14	0.429475822	1	1	1.59E-09	NO SIGNATURE NON LEUK	RPL13
RPL18	4.54E-14	0.470496601	1	0.996	1.66E-09	NO SIGNATURE NON LEUK	RPL18
EEF1A1	2.46E-13	0.320519956	1	1	9.01E-09	NO SIGNATURE NON LEUK	EEF1A1
RPL37	2.95E-13	0.394684161	1	1	1.08E-08	NO SIGNATURE NON LEUK	RPL37
RPL11	3.18E-13	0.39859761	1	1	1.16E-08	NO SIGNATURE NON LEUK	RPL11
PTMA	4.18E-13	0.489707796	0.984	0.984	1.53E-08	NO SIGNATURE NON LEUK	PTMA
RPS15	7.17E-13	0.394957529	0.995	0.996	2.62E-08	NO SIGNATURE NON LEUK	RPS15
RPL14	1.79E-12	0.447458602	0.995	0.992	6.55E-08	NO SIGNATURE NON LEUK	RPL14
RPL12	1.86E-12	0.411770559	1	1	6.81E-08	NO SIGNATURE NON LEUK	RPL12
RPS14	1.92E-12	0.418065439	1	1	7.04E-08	NO SIGNATURE NON LEUK	RPS14
RPL10A	3.64E-12	0.448551349	0.984	0.988	1.33E-07	NO SIGNATURE NON LEUK	RPL10A
RPL36	6.45E-12	0.507366142	0.984	0.996	2.36E-07	NO SIGNATURE NON LEUK	RPL36
CXCR4	7.83E-12	0.68525169	0.78	0.514	2.87E-07	NO SIGNATURE NON LEUK	CXCR4
RPL18A	8.79E-12	0.408163264	1	1	3.22E-07	NO SIGNATURE NON LEUK	RPL18A
RPL19	1.01E-11	0.373733855	1	1	3.68E-07	NO SIGNATURE NON LEUK	RPL19
RPS28	1.50E-11	0.406922292	1	1	5.48E-07	NO SIGNATURE NON LEUK	RPS28
RPL23A	1.76E-11	0.461851811	0.995	0.98	6.45E-07	NO SIGNATURE NON LEUK	RPL23A
RPS8	1.94E-11	0.400599737	1	1	7.09E-07	NO SIGNATURE NON LEUK	RPS8
RPS4X	1.95E-11	0.410280179	1	1	7.14E-07	NO SIGNATURE NON LEUK	RPS4X
RPL17	3.42E-11	0.445287039	0.995	0.996	1.25E-06	NO SIGNATURE NON LEUK	RPL17
RPS19	6.61E-11	0.455617683	1	1	2.42E-06	NO SIGNATURE NON LEUK	RPS19
RPL39	1.30E-10	0.402720455	1	1	4.77E-06	NO SIGNATURE NON LEUK	RPL39
RPL35	1.73E-10	0.405395619	0.974	0.988	6.33E-06	NO SIGNATURE NON LEUK	RPL35
NPM1	3.25E-10	0.621703594	0.775	0.631	1.19E-05	NO SIGNATURE NON LEUK	NPM1
RPS23	3.67E-10	0.370798587	1	1	1.34E-05	NO SIGNATURE NON LEUK	RPS23
RPL21	7.11E-10	0.363594509	0.984	0.988	2.60E-05	NO SIGNATURE NON LEUK	RPL21
SARAF	9.24E-10	0.590402964	0.801	0.588	3.38E-05	NO SIGNATURE NON LEUK	SARAF
EEF1B2	1.21E-09	0.471837297	0.958	0.949	4.44E-05	NO SIGNATURE NON LEUK	EEF1B2
KLF2	1.68E-09	0.810736234	0.508	0.255	6.16E-05	NO SIGNATURE NON LEUK	KLF2
RPS25	2.02E-09	0.422120244	1	0.992	7.39E-05	NO SIGNATURE NON LEUK	RPS25
RPL5	2.03E-09	0.431340819	0.995	0.992	7.45E-05	NO SIGNATURE NON LEUK	RPL5
RPS7	3.78E-09	0.336383578	1	0.996	1.38E-04	NO SIGNATURE NON LEUK	RPS7
LTB	5.47E-09	0.47230148	0.508	0.243	2.00E-04	NO SIGNATURE NON LEUK	LTB

RPLP1	6.04E-09	0.300323021	1	1	2.21E-04	NO SIGNATURE NON LEUK	RPLP1
RPS29	6.25E-09	0.430345472	0.937	0.945	2.29E-04	NO SIGNATURE NON LEUK	RPS29
RPLP2	7.05E-09	0.358588976	1	0.992	2.58E-04	NO SIGNATURE NON LEUK	RPLP2
RPL7A	9.70E-09	0.372529283	1	0.988	3.55E-04	NO SIGNATURE NON LEUK	RPL7A
RPL4	1.67E-08	0.38813088	0.948	0.941	6.11E-04	NO SIGNATURE NON LEUK	RPL4
FAU	2.23E-08	0.344067109	0.995	1	8.15E-04	NO SIGNATURE NON LEUK	FAU
RPL29	2.47E-08	0.321497818	0.99	1	9.03E-04	NO SIGNATURE NON LEUK	RPL29
LEPROTL1	3.56E-08	0.533642487	0.555	0.31	1.30E-03	NO SIGNATURE NON LEUK	LEPROTL1
RPS2	3.74E-08	0.284735685	1	1	1.37E-03	NO SIGNATURE NON LEUK	RPS2
EEF1D	5.10E-08	0.423724217	0.953	0.914	1.87E-03	NO SIGNATURE NON LEUK	EEF1D
RPL26	1.75E-07	0.284179614	1	1	6.41E-03	NO SIGNATURE NON LEUK	RPL26
RBM38	1.89E-07	0.462999406	0.455	0.22	6.93E-03	NO SIGNATURE NON LEUK	RBM38
RPS16	2.70E-07	0.352202835	0.99	0.992	9.87E-03	NO SIGNATURE NON LEUK	RPS16
EEF1G	2.78E-07	0.389322234	0.927	0.949	1.02E-02	NO SIGNATURE NON LEUK	EEF1G
RPL13A	3.15E-07	0.322983068	0.974	0.988	1.15E-02	NO SIGNATURE NON LEUK	RPL13A
ZFP36L2	3.63E-07	0.64754954	0.665	0.451	1.33E-02	NO SIGNATURE NON LEUK	ZFP36L2
RPL27	3.84E-07	0.345980981	0.974	0.973	1.41E-02	NO SIGNATURE NON LEUK	RPL27
JUND	4.65E-07	0.516875861	0.702	0.533	1.70E-02	NO SIGNATURE NON LEUK	JUND
GAS5	8.84E-07	0.482123927	0.681	0.588	3.23E-02	NO SIGNATURE NON LEUK	GAS5
RPLP0	9.06E-07	0.355259467	1	1	3.31E-02	NO SIGNATURE NON LEUK	RPLP0
CD69	1.06E-06	0.478440574	0.366	0.169	3.90E-02	NO SIGNATURE NON LEUK	CD69
TSC22D3	1.24E-06	0.830041279	0.45	0.259	4.55E-02	NO SIGNATURE NON LEUK	TSC22D3
RPL36A	1.37E-06	0.347785349	0.969	0.969	5.02E-02	NO SIGNATURE NON LEUK	RPL36A
HSP90AB1	1.47E-06	0.514276824	0.728	0.655	5.37E-02	NO SIGNATURE NON LEUK	HSP90AB1
RPL22	1.51E-06	0.346910621	0.953	0.965	5.52E-02	NO SIGNATURE NON LEUK	RPL22
RPL8	1.88E-06	0.301488676	1	0.996	6.87E-02	NO SIGNATURE NON LEUK	RPL8
NOP53	2.26E-06	0.403235066	0.775	0.765	8.25E-02	NO SIGNATURE NON LEUK	NOP53
RPL9	2.37E-06	0.353399681	0.979	0.988	8.68E-02	NO SIGNATURE NON LEUK	RPL9
RPL24	3.10E-06	0.299019241	0.979	0.992	1.14E-01	NO SIGNATURE NON LEUK	RPL24
HINT1	3.38E-06	0.458673666	0.743	0.682	1.24E-01	NO SIGNATURE NON LEUK	HINT1
TPT1	4.28E-06	0.228023344	1	1	1.57E-01	NO SIGNATURE NON LEUK	TPT1
MT-CYB	5.58E-06	0.307444511	0.99	0.988	2.04E-01	NO SIGNATURE NON LEUK	MT-CYB
RPL7	6.09E-06	0.327649393	0.921	0.906	2.23E-01	NO SIGNATURE NON LEUK	RPL7
NACA	6.51E-06	0.287175124	0.974	0.992	2.38E-01	NO SIGNATURE NON LEUK	NACA
CD7	7.55E-06	0.509089478	0.55	0.341	2.76E-01	NO SIGNATURE NON LEUK	CD7
TOMM7	8.45E-06	0.451843929	0.759	0.725	3.09E-01	NO SIGNATURE NON LEUK	TOMM7
CEBPZ	1.00E-05	0.298093229	0.267	0.106	3.67E-01	NO SIGNATURE NON LEUK	CEBPZ
CIRBP	1.12E-05	0.430948413	0.686	0.58	4.12E-01	NO SIGNATURE NON LEUK	CIRBP
ISCU	1.20E-05	0.400005763	0.319	0.157	4.39E-01	NO SIGNATURE NON LEUK	ISCU
LDHB	1.29E-05	0.40844258	0.597	0.463	4.72E-01	NO SIGNATURE NON LEUK	LDHB
CTSW	1.46E-05	0.397123239	0.398	0.204	5.35E-01	NO SIGNATURE NON LEUK	CTSW
SPOCK2	1.76E-05	0.391735077	0.445	0.239	6.45E-01	NO SIGNATURE NON LEUK	SPOCK2
RPL28	1.80E-05	0.198274708	1	1	6.57E-01	NO SIGNATURE NON LEUK	RPL28
SMAP2	1.80E-05	0.479622952	0.482	0.329	6.57E-01	NO SIGNATURE NON LEUK	SMAP2
HSP90AA1	1.98E-05	0.333214938	0.707	0.537	7.24E-01	NO SIGNATURE NON LEUK	HSP90AA1
FLT3LG	2.77E-05	0.469595567	0.471	0.286	1.00E+00	NO SIGNATURE NON LEUK	FLT3LG
LIMD2	2.78E-05	0.496257541	0.618	0.533	1.00E+00	NO SIGNATURE NON LEUK	LIMD2
IL32	3.10E-05	0.320423857	0.277	0.118	1.00E+00	NO SIGNATURE NON LEUK	IL32
DNAJB1	3.41E-05	0.426888754	0.382	0.216	1.00E+00	NO SIGNATURE NON LEUK	DNAJB1
RPL35A	3.44E-05	0.247785561	1	0.992	1.00E+00	NO SIGNATURE NON LEUK	RPL35A
RPL31	3.66E-05	0.272055099	0.932	0.957	1.00E+00	NO SIGNATURE NON LEUK	RPL31
G3BP2	4.21E-05	0.43672114	0.346	0.188	1.00E+00	NO SIGNATURE NON LEUK	G3BP2
IFITM1	5.17E-05	0.384444449	0.696	0.486	1.00E+00	NO SIGNATURE NON LEUK	IFITM1
PIK3IP1	5.18E-05	0.329478486	0.215	0.082	1.00E+00	NO SIGNATURE NON LEUK	PIK3IP1

RPL27A	5.30E-05	0.339052698	0.895	0.937	1.00E+00	NO SIGNATURE NON LEUK	RPL27A
GNAS	5.62E-05	0.379235147	0.754	0.765	1.00E+00	NO SIGNATURE NON LEUK	GNAS
HSPA8	6.02E-05	0.426131326	0.482	0.318	1.00E+00	NO SIGNATURE NON LEUK	HSPA8
ZFP36	6.71E-05	0.589285461	0.696	0.635	1.00E+00	NO SIGNATURE NON LEUK	ZFP36
PDCD4	7.12E-05	0.395622076	0.346	0.196	1.00E+00	NO SIGNATURE NON LEUK	PDCD4
CD3E	7.96E-05	0.383496516	0.471	0.306	1.00E+00	NO SIGNATURE NON LEUK	CD3E
HNRNPA1	1.04E-04	0.344049351	0.89	0.882	1.00E+00	NO SIGNATURE NON LEUK	HNRNPA1
NPDC1	1.29E-04	0.234463978	0.131	0.035	1.00E+00	NO SIGNATURE NON LEUK	NPDC1
SRRM1	1.30E-04	0.338750836	0.482	0.333	1.00E+00	NO SIGNATURE NON LEUK	SRRM1
IMP3	1.35E-04	0.326906587	0.267	0.133	1.00E+00	NO SIGNATURE NON LEUK	IMP3
GPBP1	1.37E-04	0.363592883	0.22	0.102	1.00E+00	NO SIGNATURE NON LEUK	GPBP1
RPL38	1.48E-04	0.322555433	0.906	0.918	1.00E+00	NO SIGNATURE NON LEUK	RPL38
CIB1	1.68E-04	0.387603767	0.513	0.408	1.00E+00	NO SIGNATURE NON LEUK	CIB1
SNHG7	1.96E-04	0.261529436	0.188	0.075	1.00E+00	NO SIGNATURE NON LEUK	SNHG7
CRTC3	2.03E-04	0.240751763	0.152	0.051	1.00E+00	NO SIGNATURE NON LEUK	CRTC3
RPS20	2.14E-04	0.310807803	0.853	0.867	1.00E+00	NO SIGNATURE NON LEUK	RPS20
LSR	2.15E-04	0.226309811	0.147	0.047	1.00E+00	NO SIGNATURE NON LEUK	LSR
GZMM	2.19E-04	0.28250601	0.298	0.149	1.00E+00	NO SIGNATURE NON LEUK	GZMM
RNPS1	2.41E-04	0.393402061	0.408	0.29	1.00E+00	NO SIGNATURE NON LEUK	RNPS1
IL7R	2.97E-04	0.458026604	0.319	0.188	1.00E+00	NO SIGNATURE NON LEUK	IL7R
TXNIP	3.08E-04	0.667924696	0.518	0.404	1.00E+00	NO SIGNATURE NON LEUK	TXNIP
HMGB1	3.12E-04	0.407973752	0.691	0.62	1.00E+00	NO SIGNATURE NON LEUK	HMGB1
SNHG8	4.08E-04	0.412711999	0.518	0.424	1.00E+00	NO SIGNATURE NON LEUK	SNHG8
TLE5	4.59E-04	0.424539177	0.503	0.404	1.00E+00	NO SIGNATURE NON LEUK	TLE5
DUSP1	5.06E-04	0.118213443	0.325	0.533	1.00E+00	NO SIGNATURE NON LEUK	DUSP1
SELENOK	6.93E-04	0.336122362	0.534	0.443	1.00E+00	NO SIGNATURE NON LEUK	SELENOK
PIM2	7.01E-04	0.208011757	0.194	0.082	1.00E+00	NO SIGNATURE NON LEUK	PIM2
MT-ATP8	7.21E-04	0.282651406	0.88	0.886	1.00E+00	NO SIGNATURE NON LEUK	MT-ATP8
CST7	7.35E-04	0.382556256	0.215	0.102	1.00E+00	NO SIGNATURE NON LEUK	CST7
RNF125	7.37E-04	0.423708309	0.309	0.184	1.00E+00	NO SIGNATURE NON LEUK	RNF125
SET	8.22E-04	0.319442037	0.346	0.227	1.00E+00	NO SIGNATURE NON LEUK	SET
MAL	8.23E-04	0.369795605	0.304	0.184	1.00E+00	NO SIGNATURE NON LEUK	MAL
AP3M2	8.24E-04	0.180184877	0.131	0.043	1.00E+00	NO SIGNATURE NON LEUK	AP3M2
SBDS	8.27E-04	0.307165838	0.461	0.333	1.00E+00	NO SIGNATURE NON LEUK	SBDS
ARL6IP5	8.97E-04	0.360359102	0.225	0.122	1.00E+00	NO SIGNATURE NON LEUK	ARL6IP5
CD8A	9.59E-04	0.420005994	0.33	0.204	1.00E+00	NO SIGNATURE NON LEUK	CD8A
NOL7	1.01E-03	0.304685493	0.314	0.2	1.00E+00	NO SIGNATURE NON LEUK	NOL7
GCC2	1.02E-03	0.322816282	0.204	0.102	1.00E+00	NO SIGNATURE NON LEUK	GCC2
HIST1H4C	1.04E-03	0.26867157	0.419	0.278	1.00E+00	NO SIGNATURE NON LEUK	HIST1H4C
TRBC1	1.21E-03	0.190336467	0.12	0.039	1.00E+00	NO SIGNATURE NON LEUK	TRBC1
DCXR	1.34E-03	0.288796046	0.147	0.059	1.00E+00	NO SIGNATURE NON LEUK	DCXR
OCIAD2	1.34E-03	0.260786517	0.314	0.184	1.00E+00	NO SIGNATURE NON LEUK	OCIAD2
PCM1	1.35E-03	0.243702646	0.178	0.082	1.00E+00	NO SIGNATURE NON LEUK	PCM1
SNRPA1	1.35E-03	0.313894606	0.293	0.184	1.00E+00	NO SIGNATURE NON LEUK	SNRPA1
CAMK4	1.49E-03	0.226623592	0.11	0.035	1.00E+00	NO SIGNATURE NON LEUK	CAMK4
KLRB1	1.70E-03	0.365151812	0.141	0.055	1.00E+00	NO SIGNATURE NON LEUK	KLRB1
IL21R	2.03E-03	0.152218722	0.141	0.055	1.00E+00	NO SIGNATURE NON LEUK	IL21R
CCDC85B	2.09E-03	0.300189528	0.246	0.145	1.00E+00	NO SIGNATURE NON LEUK	CCDC85B
LINC00667	2.13E-03	0.182192953	0.126	0.047	1.00E+00	NO SIGNATURE NON LEUK	LINC00667
RHOH	2.39E-03	0.266814523	0.262	0.157	1.00E+00	NO SIGNATURE NON LEUK	RHOH
ARID4B	2.53E-03	0.305800747	0.335	0.231	1.00E+00	NO SIGNATURE NON LEUK	ARID4B
ITM2C	2.62E-03	0.36844201	0.246	0.145	1.00E+00	NO SIGNATURE NON LEUK	ITM2C
CALM2	2.80E-03	0.324484103	0.487	0.384	1.00E+00	NO SIGNATURE NON LEUK	CALM2
PDE3B	3.62E-03	0.182140846	0.215	0.114	1.00E+00	NO SIGNATURE NON LEUK	PDE3B

EZR	3.77E-03	0.414568558	0.398	0.294	1.00E+00	NO SIGNATURE NON LEUK	EZR
SOD1	3.92E-03	0.341154843	0.372	0.267	1.00E+00	NO SIGNATURE NON LEUK	SOD1
ACIN1	4.08E-03	0.201890047	0.141	0.063	1.00E+00	NO SIGNATURE NON LEUK	ACIN1
UBALD2	4.13E-03	0.363929192	0.455	0.38	1.00E+00	NO SIGNATURE NON LEUK	UBALD2
GOLPH3	4.13E-03	0.192407168	0.136	0.059	1.00E+00	NO SIGNATURE NON LEUK	GOLPH3
TMC6	4.85E-03	0.176905697	0.105	0.039	1.00E+00	NO SIGNATURE NON LEUK	TMC6
HIST1H1C	4.94E-03	0.285552637	0.361	0.259	1.00E+00	NO SIGNATURE NON LEUK	HIST1H1C
CDKN1B	5.02E-03	0.232360519	0.183	0.094	1.00E+00	NO SIGNATURE NON LEUK	CDKN1B
DUSP11	5.07E-03	0.192444025	0.11	0.043	1.00E+00	NO SIGNATURE NON LEUK	DUSP11
HNRNPA0	5.55E-03	0.405959959	0.508	0.463	1.00E+00	NO SIGNATURE NON LEUK	HNRNPA0
MORF4L1	5.61E-03	0.329622135	0.492	0.439	1.00E+00	NO SIGNATURE NON LEUK	MORF4L1
RNMT	5.74E-03	0.270883805	0.319	0.224	1.00E+00	NO SIGNATURE NON LEUK	RNMT
CSNK1D	5.76E-03	0.28176425	0.215	0.125	1.00E+00	NO SIGNATURE NON LEUK	CSNK1D
RACK1	6.09E-03	0.186190145	0.963	0.988	1.00E+00	NO SIGNATURE NON LEUK	RACK1
CYFIP2	6.20E-03	0.189019232	0.152	0.075	1.00E+00	NO SIGNATURE NON LEUK	CYFIP2
RPS13	6.29E-03	0.187674713	1	0.996	1.00E+00	NO SIGNATURE NON LEUK	RPS13
TERF2IP	6.57E-03	0.206327298	0.23	0.141	1.00E+00	NO SIGNATURE NON LEUK	TERF2IP
STK17A	6.72E-03	0.235549595	0.461	0.361	1.00E+00	NO SIGNATURE NON LEUK	STK17A
CD247	6.91E-03	0.264490663	0.293	0.188	1.00E+00	NO SIGNATURE NON LEUK	CD247
CLTB	6.98E-03	0.289446822	0.225	0.137	1.00E+00	NO SIGNATURE NON LEUK	CLTB
DDX24	7.13E-03	0.265912001	0.476	0.404	1.00E+00	NO SIGNATURE NON LEUK	DDX24
RNF126	7.28E-03	0.242491207	0.22	0.129	1.00E+00	NO SIGNATURE NON LEUK	RNF126
BCCIP	7.58E-03	0.15985929	0.126	0.055	1.00E+00	NO SIGNATURE NON LEUK	BCCIP
SLBP	7.96E-03	0.140558465	0.162	0.078	1.00E+00	NO SIGNATURE NON LEUK	SLBP
DDX6	8.07E-03	0.185407809	0.188	0.106	1.00E+00	NO SIGNATURE NON LEUK	DDX6
STK17B	8.11E-03	0.337909354	0.56	0.518	1.00E+00	NO SIGNATURE NON LEUK	STK17B
DDX46	8.28E-03	0.229990804	0.141	0.071	1.00E+00	NO SIGNATURE NON LEUK	DDX46
PEBP1	8.38E-03	0.234101352	0.346	0.247	1.00E+00	NO SIGNATURE NON LEUK	PEBP1
CD6	8.56E-03	0.247481171	0.194	0.106	1.00E+00	NO SIGNATURE NON LEUK	CD6
PLSCR3	8.73E-03	0.222673453	0.136	0.067	1.00E+00	NO SIGNATURE NON LEUK	PLSCR3
SOCS1	9.14E-03	0.310999519	0.319	0.231	1.00E+00	NO SIGNATURE NON LEUK	SOCS1
DDX17	9.77E-03	0.20919925	0.157	0.082	1.00E+00	NO SIGNATURE NON LEUK	DDX17
CD44	5.90E-75	1.812365765	1	0.309	2.16E-70	CD44 > 1 & MKI67 < 1 NON LEUK	CD44
IL1B	1.55E-24	2.46125195	0.612	0.131	5.66E-20	CD44 > 1 & MKI67 < 1 NON LEUK	IL1B
PLAUR	3.49E-24	2.035446568	0.612	0.152	1.28E-19	CD44 > 1 & MKI67 < 1 NON LEUK	PLAUR
IL1RN	1.53E-23	1.925511475	0.584	0.11	5.61E-19	CD44 > 1 & MKI67 < 1 NON LEUK	IL1RN
BCL2A1	1.53E-23	1.380084723	0.635	0.168	5.61E-19	CD44 > 1 & MKI67 < 1 NON LEUK	BCL2A1
VIM	2.28E-23	1.54590183	0.961	0.916	8.36E-19	CD44 > 1 & MKI67 < 1 NON LEUK	VIM
THBS1	2.32E-23	1.732785379	0.592	0.12	8.48E-19	CD44 > 1 & MKI67 < 1 NON LEUK	THBS1
CXCL8	2.50E-23	2.092070968	0.624	0.168	9.15E-19	CD44 > 1 & MKI67 < 1 NON LEUK	CXCL8
FTH1	3.72E-23	1.08692267	1	0.995	1.36E-18	CD44 > 1 & MKI67 < 1 NON LEUK	FTH1
FCN1	3.95E-23	1.575071571	0.596	0.126	1.45E-18	CD44 > 1 & MKI67 < 1 NON LEUK	FCN1
ATP2B1-AS1	7.96E-23	1.550074028	0.58	0.141	2.91E-18	CD44 > 1 & MKI67 < 1 NON LEUK	ATP2B1-AS1
CCL3	1.62E-22	2.578365145	0.596	0.141	5.91E-18	CD44 > 1 & MKI67 < 1 NON LEUK	CCL3
SPHK1	2.31E-22	1.048326603	0.569	0.105	8.44E-18	CD44 > 1 & MKI67 < 1 NON LEUK	SPHK1
CXCL2	1.05E-21	1.320063731	0.545	0.099	3.85E-17	CD44 > 1 & MKI67 < 1 NON LEUK	CXCL2
SERPINB2	1.16E-21	2.097900691	0.569	0.131	4.26E-17	CD44 > 1 & MKI67 < 1 NON LEUK	SERPINB2
C15orf48	1.27E-21	1.558636755	0.573	0.115	4.65E-17	CD44 > 1 & MKI67 < 1 NON LEUK	C15orf48
CST3	1.86E-21	1.575778611	0.627	0.162	6.79E-17	CD44 > 1 & MKI67 < 1 NON LEUK	CST3
CCL3L1	2.01E-21	2.295600543	0.529	0.094	7.34E-17	CD44 > 1 & MKI67 < 1 NON LEUK	CCL3L1
IER3	2.08E-21	1.086494641	0.651	0.199	7.62E-17	CD44 > 1 & MKI67 < 1 NON LEUK	IER3
MAFB	3.09E-21	0.956309844	0.573	0.12	1.13E-16	CD44 > 1 & MKI67 < 1 NON LEUK	MAFB
CTSL	3.70E-21	1.300294688	0.561	0.115	1.35E-16	CD44 > 1 & MKI67 < 1 NON LEUK	CTSL
AC015912.3	6.46E-21	0.988233932	0.478	0.068	2.36E-16	CD44 > 1 & MKI67 < 1 NON LEUK	AC015912.3

LYZ	7.30E-21	1.426193444	0.573	0.12	2.67E-16	CD44 > 1 & MKI67 < 1 NON LEUK	LYZ
SOD2	1.33E-20	2.250565121	0.737	0.414	4.87E-16	CD44 > 1 & MKI67 < 1 NON LEUK	SOD2
LGALS3	2.29E-20	1.451685418	0.643	0.241	8.39E-16	CD44 > 1 & MKI67 < 1 NON LEUK	LGALS3
BASP1	2.45E-20	0.965605869	0.565	0.131	8.95E-16	CD44 > 1 & MKI67 < 1 NON LEUK	BASP1
CD68	2.52E-20	1.122268529	0.576	0.141	9.21E-16	CD44 > 1 & MKI67 < 1 NON LEUK	CD68
DRAM1	2.62E-20	0.883342204	0.51	0.089	9.58E-16	CD44 > 1 & MKI67 < 1 NON LEUK	DRAM1
IFNGR2	2.85E-20	1.092557144	0.616	0.194	1.04E-15	CD44 > 1 & MKI67 < 1 NON LEUK	IFNGR2
S100A10	3.16E-20	1.227658328	0.812	0.466	1.16E-15	CD44 > 1 & MKI67 < 1 NON LEUK	S100A10
ICAM1	3.39E-20	0.782028537	0.541	0.11	1.24E-15	CD44 > 1 & MKI67 < 1 NON LEUK	ICAM1
CSTB	3.72E-20	0.990429177	0.651	0.199	1.36E-15	CD44 > 1 & MKI67 < 1 NON LEUK	CSTB
CCL4L2	4.43E-20	1.776430743	0.482	0.079	1.62E-15	CD44 > 1 & MKI67 < 1 NON LEUK	CCL4L2
S100A9	9.14E-20	1.939975081	0.604	0.178	3.35E-15	CD44 > 1 & MKI67 < 1 NON LEUK	S100A9
DNAAF1	1.15E-19	0.922781593	0.482	0.079	4.21E-15	CD44 > 1 & MKI67 < 1 NON LEUK	DNAAF1
SERPINA1	1.18E-19	0.987584592	0.557	0.12	4.33E-15	CD44 > 1 & MKI67 < 1 NON LEUK	SERPINA1
PTGS2	1.25E-19	0.884861736	0.435	0.047	4.59E-15	CD44 > 1 & MKI67 < 1 NON LEUK	PTGS2
ANXA5	1.51E-19	0.844049995	0.635	0.178	5.53E-15	CD44 > 1 & MKI67 < 1 NON LEUK	ANXA5
S100A8	1.59E-19	1.315365105	0.584	0.136	5.82E-15	CD44 > 1 & MKI67 < 1 NON LEUK	S100A8
PHLDA1	1.83E-19	0.868480773	0.569	0.141	6.71E-15	CD44 > 1 & MKI67 < 1 NON LEUK	PHLDA1
IFI30	2.73E-19	1.122016931	0.588	0.126	1.00E-14	CD44 > 1 & MKI67 < 1 NON LEUK	IFI30
TYROBP	4.12E-19	1.191399084	0.604	0.157	1.51E-14	CD44 > 1 & MKI67 < 1 NON LEUK	TYROBP
S100A6	5.22E-19	1.313905648	0.8	0.508	1.91E-14	CD44 > 1 & MKI67 < 1 NON LEUK	S100A6
FCER1G	7.15E-19	0.900270573	0.576	0.131	2.62E-14	CD44 > 1 & MKI67 < 1 NON LEUK	FCER1G
CCL4	7.66E-19	2.014596482	0.537	0.136	2.80E-14	CD44 > 1 & MKI67 < 1 NON LEUK	CCL4
CD63	7.73E-19	1.046202403	0.62	0.23	2.83E-14	CD44 > 1 & MKI67 < 1 NON LEUK	CD63
MAP3K8	1.34E-18	0.968902492	0.678	0.267	4.91E-14	CD44 > 1 & MKI67 < 1 NON LEUK	MAP3K8
MSC	2.03E-18	0.839602962	0.494	0.099	7.42E-14	CD44 > 1 & MKI67 < 1 NON LEUK	MSC
G0S2	3.34E-18	1.112571794	0.49	0.094	1.22E-13	CD44 > 1 & MKI67 < 1 NON LEUK	G0S2
PPP1R15A	3.45E-18	1.30401731	0.694	0.34	1.26E-13	CD44 > 1 & MKI67 < 1 NON LEUK	PPP1R15A
ITGB8	4.37E-18	0.764167513	0.471	0.084	1.60E-13	CD44 > 1 & MKI67 < 1 NON LEUK	ITGB8
S100A12	4.98E-18	0.993168376	0.514	0.094	1.82E-13	CD44 > 1 & MKI67 < 1 NON LEUK	S100A12
HLA-DRA	5.69E-18	1.245731817	0.647	0.22	2.08E-13	CD44 > 1 & MKI67 < 1 NON LEUK	HLA-DRA
AIF1	7.09E-18	0.914276194	0.643	0.236	2.60E-13	CD44 > 1 & MKI67 < 1 NON LEUK	AIF1
TXN	9.68E-18	0.866249102	0.643	0.22	3.54E-13	CD44 > 1 & MKI67 < 1 NON LEUK	TXN
LGALS1	1.07E-17	1.026084895	0.608	0.209	3.90E-13	CD44 > 1 & MKI67 < 1 NON LEUK	LGALS1
CD83	1.18E-17	0.790058556	0.569	0.168	4.32E-13	CD44 > 1 & MKI67 < 1 NON LEUK	CD83
S100A4	1.95E-17	1.076850577	0.808	0.487	7.16E-13	CD44 > 1 & MKI67 < 1 NON LEUK	S100A4
NRP2	2.43E-17	0.635358732	0.435	0.068	8.89E-13	CD44 > 1 & MKI67 < 1 NON LEUK	NRP2
CSTA	3.75E-17	0.658180224	0.498	0.105	1.37E-12	CD44 > 1 & MKI67 < 1 NON LEUK	CSTA
HLA-DRB1	3.94E-17	1.374456697	0.631	0.257	1.44E-12	CD44 > 1 & MKI67 < 1 NON LEUK	HLA-DRB1
EHD1	4.20E-17	0.852293967	0.573	0.173	1.54E-12	CD44 > 1 & MKI67 < 1 NON LEUK	EHD1
ATP6V1F	4.33E-17	0.822586709	0.6	0.215	1.59E-12	CD44 > 1 & MKI67 < 1 NON LEUK	ATP6V1F
TIMP1	4.62E-17	1.228964948	0.647	0.277	1.69E-12	CD44 > 1 & MKI67 < 1 NON LEUK	TIMP1
AQP9	4.84E-17	0.58249503	0.427	0.063	1.77E-12	CD44 > 1 & MKI67 < 1 NON LEUK	AQP9
UBE2D1	5.45E-17	0.655056869	0.494	0.11	2.00E-12	CD44 > 1 & MKI67 < 1 NON LEUK	UBE2D1
NFKBIA	8.82E-17	1.148624414	0.835	0.592	3.23E-12	CD44 > 1 & MKI67 < 1 NON LEUK	NFKBIA
FLT1	1.06E-16	0.840850124	0.506	0.126	3.90E-12	CD44 > 1 & MKI67 < 1 NON LEUK	FLT1
RNF144B	1.08E-16	0.720286893	0.514	0.131	3.95E-12	CD44 > 1 & MKI67 < 1 NON LEUK	RNF144B
ZEB2	1.18E-16	0.736421579	0.576	0.173	4.33E-12	CD44 > 1 & MKI67 < 1 NON LEUK	ZEB2
CYTOR	1.27E-16	0.730799922	0.604	0.215	4.63E-12	CD44 > 1 & MKI67 < 1 NON LEUK	CYTOR
ITGAX	1.86E-16	0.652179815	0.475	0.099	6.81E-12	CD44 > 1 & MKI67 < 1 NON LEUK	ITGAX
CLEC7A	1.94E-16	0.710418456	0.475	0.099	7.10E-12	CD44 > 1 & MKI67 < 1 NON LEUK	CLEC7A
CTSS	2.19E-16	0.702034192	0.565	0.178	8.01E-12	CD44 > 1 & MKI67 < 1 NON LEUK	CTSS
FTL	2.85E-16	1.431418512	0.984	0.942	1.04E-11	CD44 > 1 & MKI67 < 1 NON LEUK	FTL
S100A11	3.29E-16	1.224172474	0.722	0.414	1.20E-11	CD44 > 1 & MKI67 < 1 NON LEUK	S100A11

CPVL	3.31E-16	0.605959228	0.404	0.058	1.21E-11	CD44 > 1 & MKI67 < 1 NON LEUK	CPVL
BID	3.64E-16	0.71233529	0.545	0.173	1.33E-11	CD44 > 1 & MKI67 < 1 NON LEUK	BID
EREG	4.71E-16	0.826720967	0.463	0.105	1.72E-11	CD44 > 1 & MKI67 < 1 NON LEUK	EREG
GRN	5.49E-16	0.780217302	0.533	0.152	2.01E-11	CD44 > 1 & MKI67 < 1 NON LEUK	GRN
CXCL16	7.25E-16	0.554986764	0.451	0.089	2.65E-11	CD44 > 1 & MKI67 < 1 NON LEUK	CXCL16
PLEK	7.55E-16	0.71905209	0.533	0.147	2.76E-11	CD44 > 1 & MKI67 < 1 NON LEUK	PLEK
JARID2	9.69E-16	0.578807162	0.478	0.11	3.55E-11	CD44 > 1 & MKI67 < 1 NON LEUK	JARID2
NINJ1	1.39E-15	1.180605027	0.718	0.435	5.09E-11	CD44 > 1 & MKI67 < 1 NON LEUK	NINJ1
SAT1	1.43E-15	1.054585821	0.741	0.44	5.25E-11	CD44 > 1 & MKI67 < 1 NON LEUK	SAT1
PSAP	2.38E-15	0.839247675	0.678	0.33	8.70E-11	CD44 > 1 & MKI67 < 1 NON LEUK	PSAP
NAMPT	2.71E-15	0.633452954	0.576	0.183	9.93E-11	CD44 > 1 & MKI67 < 1 NON LEUK	NAMPT
PKM	3.76E-15	0.743230337	0.612	0.236	1.38E-10	CD44 > 1 & MKI67 < 1 NON LEUK	PKM
APLP2	4.57E-15	0.582490149	0.478	0.131	1.67E-10	CD44 > 1 & MKI67 < 1 NON LEUK	APLP2
ETS2	5.40E-15	0.724512757	0.588	0.225	1.98E-10	CD44 > 1 & MKI67 < 1 NON LEUK	ETS2
CFP	7.88E-15	0.578440569	0.478	0.115	2.88E-10	CD44 > 1 & MKI67 < 1 NON LEUK	CFP
CD74	8.29E-15	0.707397435	0.808	0.518	3.03E-10	CD44 > 1 & MKI67 < 1 NON LEUK	CD74
DENND5A	1.35E-14	0.507461519	0.431	0.094	4.95E-10	CD44 > 1 & MKI67 < 1 NON LEUK	DENND5A
PALM2-AKAP2	1.61E-14	0.604714332	0.4	0.084	5.88E-10	CD44 > 1 & MKI67 < 1 NON LEUK	PALM2-AKAP2
GAPDH	1.83E-14	0.837197001	0.945	0.843	6.71E-10	CD44 > 1 & MKI67 < 1 NON LEUK	GAPDH
CEBPD	2.00E-14	0.553142787	0.478	0.115	7.32E-10	CD44 > 1 & MKI67 < 1 NON LEUK	CEBPD
SPI1	2.84E-14	0.603060792	0.486	0.126	1.04E-09	CD44 > 1 & MKI67 < 1 NON LEUK	SPI1
SH3BGRL3	3.00E-14	0.737602863	0.945	0.822	1.10E-09	CD44 > 1 & MKI67 < 1 NON LEUK	SH3BGRL3
F3	3.01E-14	0.815780504	0.38	0.063	1.10E-09	CD44 > 1 & MKI67 < 1 NON LEUK	F3
LUCAT1	3.80E-14	0.473130815	0.365	0.052	1.39E-09	CD44 > 1 & MKI67 < 1 NON LEUK	LUCAT1
SMS	4.08E-14	0.598594661	0.494	0.147	1.49E-09	CD44 > 1 & MKI67 < 1 NON LEUK	SMS
NLRP3	4.20E-14	0.448854506	0.369	0.052	1.54E-09	CD44 > 1 & MKI67 < 1 NON LEUK	NLRP3
HLA-DQB1	5.11E-14	0.969022764	0.616	0.251	1.87E-09	CD44 > 1 & MKI67 < 1 NON LEUK	HLA-DQB1
USP12	5.25E-14	0.658287367	0.537	0.188	1.92E-09	CD44 > 1 & MKI67 < 1 NON LEUK	USP12
IL6	6.60E-14	0.903675534	0.357	0.058	2.42E-09	CD44 > 1 & MKI67 < 1 NON LEUK	IL6
MIR3945HG	7.53E-14	0.473315026	0.294	0.021	2.76E-09	CD44 > 1 & MKI67 < 1 NON LEUK	MIR3945HG
TSPO	7.92E-14	0.616422218	0.616	0.246	2.90E-09	CD44 > 1 & MKI67 < 1 NON LEUK	TSPO
INSIG1	8.64E-14	0.692916096	0.529	0.178	3.16E-09	CD44 > 1 & MKI67 < 1 NON LEUK	INSIG1
CFD	9.67E-14	0.467792495	0.408	0.079	3.54E-09	CD44 > 1 & MKI67 < 1 NON LEUK	CFD
PDE4DIP	9.87E-14	0.557311172	0.486	0.131	3.61E-09	CD44 > 1 & MKI67 < 1 NON LEUK	PDE4DIP
RIN3	1.02E-13	0.444720251	0.447	0.11	3.73E-09	CD44 > 1 & MKI67 < 1 NON LEUK	RIN3
GABARAP	1.11E-13	0.713870466	0.655	0.319	4.08E-09	CD44 > 1 & MKI67 < 1 NON LEUK	GABARAP
OAZ1	1.42E-13	0.878045059	0.725	0.445	5.20E-09	CD44 > 1 & MKI67 < 1 NON LEUK	OAZ1
PID1	2.09E-13	0.591744944	0.369	0.068	7.66E-09	CD44 > 1 & MKI67 < 1 NON LEUK	PID1
CTSD	2.93E-13	0.764434856	0.565	0.22	1.07E-08	CD44 > 1 & MKI67 < 1 NON LEUK	CTSD
INHBA	3.04E-13	0.554663161	0.353	0.058	1.11E-08	CD44 > 1 & MKI67 < 1 NON LEUK	INHBA
DUSP6	3.74E-13	0.528623916	0.514	0.157	1.37E-08	CD44 > 1 & MKI67 < 1 NON LEUK	DUSP6
NFKBIZ	4.02E-13	0.515281354	0.545	0.183	1.47E-08	CD44 > 1 & MKI67 < 1 NON LEUK	NFKBIZ
SRGN	4.96E-13	0.704893827	0.906	0.749	1.82E-08	CD44 > 1 & MKI67 < 1 NON LEUK	SRGN
RGCC	5.05E-13	0.629249312	0.62	0.251	1.85E-08	CD44 > 1 & MKI67 < 1 NON LEUK	RGCC
APOBEC3A	5.93E-13	0.729639412	0.373	0.068	2.17E-08	CD44 > 1 & MKI67 < 1 NON LEUK	APOBEC3A
CYBA	6.79E-13	0.669037389	0.753	0.445	2.49E-08	CD44 > 1 & MKI67 < 1 NON LEUK	CYBA
IL1R1	6.83E-13	0.450635546	0.349	0.058	2.50E-08	CD44 > 1 & MKI67 < 1 NON LEUK	IL1R1
GRINA	6.88E-13	0.640624997	0.569	0.225	2.52E-08	CD44 > 1 & MKI67 < 1 NON LEUK	GRINA
MGLL	1.27E-12	0.414264512	0.337	0.058	4.65E-08	CD44 > 1 & MKI67 < 1 NON LEUK	MGLL
SMOX	1.46E-12	0.560918087	0.427	0.11	5.34E-08	CD44 > 1 & MKI67 < 1 NON LEUK	SMOX
NPC2	1.66E-12	0.734711778	0.584	0.267	6.08E-08	CD44 > 1 & MKI67 < 1 NON LEUK	NPC2
OGFRL1	2.41E-12	0.535078513	0.396	0.094	8.81E-08	CD44 > 1 & MKI67 < 1 NON LEUK	OGFRL1
ATP2B1	3.08E-12	0.60930678	0.525	0.188	1.13E-07	CD44 > 1 & MKI67 < 1 NON LEUK	ATP2B1
NCF2	4.98E-12	0.398645719	0.341	0.063	1.82E-07	CD44 > 1 & MKI67 < 1 NON LEUK	NCF2

PDLIM5	5.83E-12	0.37936563	0.373	0.079	2.14E-07	CD44 > 1 & MKI67 < 1 NON LEUK	PDLIM5
CD300C	7.64E-12	0.402404039	0.361	0.079	2.80E-07	CD44 > 1 & MKI67 < 1 NON LEUK	CD300C
MTSS1	7.65E-12	0.481882659	0.412	0.105	2.80E-07	CD44 > 1 & MKI67 < 1 NON LEUK	MTSS1
BZW1	8.85E-12	0.664968175	0.765	0.476	3.24E-07	CD44 > 1 & MKI67 < 1 NON LEUK	BZW1
TMSB10	9.32E-12	0.479126927	0.988	0.974	3.41E-07	CD44 > 1 & MKI67 < 1 NON LEUK	TMSB10
PTPRJ	1.00E-11	0.449799042	0.388	0.094	3.66E-07	CD44 > 1 & MKI67 < 1 NON LEUK	PTPRJ
BACH1	1.66E-11	0.422977011	0.459	0.141	6.09E-07	CD44 > 1 & MKI67 < 1 NON LEUK	BACH1
PNP	1.84E-11	0.444262922	0.38	0.094	6.73E-07	CD44 > 1 & MKI67 < 1 NON LEUK	PNP
RIN2	2.02E-11	0.366825501	0.306	0.047	7.38E-07	CD44 > 1 & MKI67 < 1 NON LEUK	RIN2
CXCL3	2.14E-11	0.811501719	0.357	0.084	7.84E-07	CD44 > 1 & MKI67 < 1 NON LEUK	CXCL3
SDC2	2.56E-11	0.304909636	0.267	0.026	9.36E-07	CD44 > 1 & MKI67 < 1 NON LEUK	SDC2
ADM	2.61E-11	0.375053479	0.282	0.037	9.54E-07	CD44 > 1 & MKI67 < 1 NON LEUK	ADM
CCL7	2.82E-11	0.779467595	0.251	0.021	1.03E-06	CD44 > 1 & MKI67 < 1 NON LEUK	CCL7
ALDOA	2.92E-11	0.586896937	0.592	0.283	1.07E-06	CD44 > 1 & MKI67 < 1 NON LEUK	ALDOA
CSF3R	3.05E-11	0.412712243	0.392	0.105	1.12E-06	CD44 > 1 & MKI67 < 1 NON LEUK	CSF3R
TREM1	3.07E-11	0.392502991	0.353	0.079	1.12E-06	CD44 > 1 & MKI67 < 1 NON LEUK	TREM1
MET	3.27E-11	0.377026292	0.306	0.047	1.20E-06	CD44 > 1 & MKI67 < 1 NON LEUK	MET
CTSB	3.49E-11	0.44345813	0.404	0.115	1.28E-06	CD44 > 1 & MKI67 < 1 NON LEUK	CTSB
UBE2J1	3.80E-11	0.544608748	0.529	0.215	1.39E-06	CD44 > 1 & MKI67 < 1 NON LEUK	UBE2J1
MPP1	5.43E-11	0.395096861	0.38	0.099	1.99E-06	CD44 > 1 & MKI67 < 1 NON LEUK	MPP1
FCGRT	5.51E-11	0.480731952	0.502	0.194	2.02E-06	CD44 > 1 & MKI67 < 1 NON LEUK	FCGRT
TNFRSF1B	5.75E-11	0.502356361	0.482	0.168	2.10E-06	CD44 > 1 & MKI67 < 1 NON LEUK	TNFRSF1B
PMAIP1	5.90E-11	0.614713227	0.529	0.225	2.16E-06	CD44 > 1 & MKI67 < 1 NON LEUK	PMAIP1
SLC25A37	7.37E-11	0.396804903	0.333	0.068	2.70E-06	CD44 > 1 & MKI67 < 1 NON LEUK	SLC25A37
MSANTD3	1.03E-10	0.228338243	0.231	0.016	3.78E-06	CD44 > 1 & MKI67 < 1 NON LEUK	MSANTD3
SLC16A6	1.17E-10	0.284183172	0.251	0.026	4.27E-06	CD44 > 1 & MKI67 < 1 NON LEUK	SLC16A6
METRNL	1.19E-10	0.486109817	0.604	0.267	4.34E-06	CD44 > 1 & MKI67 < 1 NON LEUK	METRNL
RASGEF1B	1.28E-10	0.363547661	0.345	0.079	4.69E-06	CD44 > 1 & MKI67 < 1 NON LEUK	RASGEF1B
MARCKS	1.42E-10	0.380161528	0.298	0.052	5.19E-06	CD44 > 1 & MKI67 < 1 NON LEUK	MARCKS
PTX3	1.50E-10	0.487235596	0.224	0.016	5.50E-06	CD44 > 1 & MKI67 < 1 NON LEUK	PTX3
KYNU	1.83E-10	0.288576397	0.278	0.042	6.70E-06	CD44 > 1 & MKI67 < 1 NON LEUK	KYNU
KLF10	2.05E-10	0.382506048	0.408	0.126	7.50E-06	CD44 > 1 & MKI67 < 1 NON LEUK	KLF10
GNG5	2.07E-10	0.449943471	0.588	0.267	7.56E-06	CD44 > 1 & MKI67 < 1 NON LEUK	GNG5
DSE	2.19E-10	0.403606578	0.369	0.105	8.00E-06	CD44 > 1 & MKI67 < 1 NON LEUK	DSE
SERPINB9	2.20E-10	0.456780846	0.451	0.152	8.06E-06	CD44 > 1 & MKI67 < 1 NON LEUK	SERPINB9
TNIP1	2.31E-10	0.488355261	0.537	0.22	8.47E-06	CD44 > 1 & MKI67 < 1 NON LEUK	TNIP1
TMSB4X	2.35E-10	0.431737837	1	1	8.60E-06	CD44 > 1 & MKI67 < 1 NON LEUK	TMSB4X
ATP13A3	2.37E-10	0.366186591	0.322	0.068	8.66E-06	CD44 > 1 & MKI67 < 1 NON LEUK	ATP13A3
FCGR2A	2.48E-10	0.319707456	0.302	0.058	9.06E-06	CD44 > 1 & MKI67 < 1 NON LEUK	FCGR2A
ANXA1	2.74E-10	0.559144217	0.671	0.366	1.00E-05	CD44 > 1 & MKI67 < 1 NON LEUK	ANXA1
ANXA2	2.81E-10	0.557594953	0.631	0.33	1.03E-05	CD44 > 1 & MKI67 < 1 NON LEUK	ANXA2
C19orf38	2.93E-10	0.338848573	0.298	0.058	1.07E-05	CD44 > 1 & MKI67 < 1 NON LEUK	C19orf38
MS4A7	3.68E-10	0.349530163	0.325	0.073	1.35E-05	CD44 > 1 & MKI67 < 1 NON LEUK	MS4A7
TUBB6	3.73E-10	0.318952291	0.337	0.084	1.37E-05	CD44 > 1 & MKI67 < 1 NON LEUK	TUBB6
PGAM1	3.78E-10	0.533336285	0.529	0.236	1.38E-05	CD44 > 1 & MKI67 < 1 NON LEUK	PGAM1
SLC7A7	3.99E-10	0.341238725	0.333	0.079	1.46E-05	CD44 > 1 & MKI67 < 1 NON LEUK	SLC7A7
BRI3	4.29E-10	0.375805458	0.518	0.194	1.57E-05	CD44 > 1 & MKI67 < 1 NON LEUK	BRI3
ASPH	4.60E-10	0.367872584	0.333	0.079	1.68E-05	CD44 > 1 & MKI67 < 1 NON LEUK	ASPH
PDXK	4.61E-10	0.364251374	0.388	0.11	1.69E-05	CD44 > 1 & MKI67 < 1 NON LEUK	PDXK
CARD16	6.13E-10	0.292375968	0.306	0.058	2.24E-05	CD44 > 1 & MKI67 < 1 NON LEUK	CARD16
CDC42EP3	6.43E-10	0.433548035	0.518	0.215	2.35E-05	CD44 > 1 & MKI67 < 1 NON LEUK	CDC42EP3
DMXL2	6.80E-10	0.289279951	0.235	0.026	2.49E-05	CD44 > 1 & MKI67 < 1 NON LEUK	DMXL2
WTAP	7.49E-10	0.60130299	0.671	0.424	2.74E-05	CD44 > 1 & MKI67 < 1 NON LEUK	WTAP
PTPRE	7.88E-10	0.368155248	0.373	0.11	2.89E-05	CD44 > 1 & MKI67 < 1 NON LEUK	PTPRE

PPIF	8.50E-10	0.408396258	0.365	0.105	3.11E-05	CD44 > 1 & MKI67 < 1 NON LEUK	PPIF
PELI1	9.76E-10	0.65174326	0.537	0.251	3.57E-05	CD44 > 1 & MKI67 < 1 NON LEUK	PELI1
TYMP	1.04E-09	0.295569548	0.318	0.068	3.79E-05	CD44 > 1 & MKI67 < 1 NON LEUK	TYMP
CREG1	1.13E-09	0.335721666	0.227	0.026	4.15E-05	CD44 > 1 & MKI67 < 1 NON LEUK	CREG1
HLA-DRB5	1.29E-09	0.560299537	0.537	0.236	4.70E-05	CD44 > 1 & MKI67 < 1 NON LEUK	HLA-DRB5
FPR1	1.37E-09	0.386135175	0.314	0.079	5.03E-05	CD44 > 1 & MKI67 < 1 NON LEUK	FPR1
SLC43A2	1.65E-09	0.289436475	0.341	0.089	6.02E-05	CD44 > 1 & MKI67 < 1 NON LEUK	SLC43A2
GSTO1	1.76E-09	0.474937861	0.478	0.204	6.45E-05	CD44 > 1 & MKI67 < 1 NON LEUK	GSTO1
SNX18	1.82E-09	0.248117927	0.22	0.021	6.66E-05	CD44 > 1 & MKI67 < 1 NON LEUK	SNX18
CHMP4B	1.91E-09	0.291853165	0.329	0.079	7.01E-05	CD44 > 1 & MKI67 < 1 NON LEUK	CHMP4B
PSMA6	1.97E-09	0.46184993	0.549	0.251	7.22E-05	CD44 > 1 & MKI67 < 1 NON LEUK	PSMA6
EMILIN2	1.99E-09	0.267174381	0.255	0.042	7.30E-05	CD44 > 1 & MKI67 < 1 NON LEUK	EMILIN2
MYO9B	2.03E-09	0.310071379	0.42	0.136	7.43E-05	CD44 > 1 & MKI67 < 1 NON LEUK	MYO9B
CCRL2	2.12E-09	0.360678564	0.286	0.063	7.76E-05	CD44 > 1 & MKI67 < 1 NON LEUK	CCRL2
SPRY2	2.19E-09	0.293705929	0.227	0.026	8.03E-05	CD44 > 1 & MKI67 < 1 NON LEUK	SPRY2
SLC16A3	2.24E-09	0.262566772	0.204	0.016	8.20E-05	CD44 > 1 & MKI67 < 1 NON LEUK	SLC16A3
AZIN1-AS1	2.36E-09	0.245036269	0.235	0.031	8.63E-05	CD44 > 1 & MKI67 < 1 NON LEUK	AZIN1-AS1
PRKAG2	2.36E-09	0.322593237	0.29	0.063	8.64E-05	CD44 > 1 & MKI67 < 1 NON LEUK	PRKAG2
ZFAND5	3.37E-09	0.431075461	0.514	0.22	1.23E-04	CD44 > 1 & MKI67 < 1 NON LEUK	ZFAND5
NRGN	3.62E-09	0.22845748	0.263	0.047	1.33E-04	CD44 > 1 & MKI67 < 1 NON LEUK	NRGN
P4HB	3.95E-09	0.416970578	0.522	0.225	1.44E-04	CD44 > 1 & MKI67 < 1 NON LEUK	P4HB
CCDC71L	4.05E-09	0.3294859	0.329	0.089	1.48E-04	CD44 > 1 & MKI67 < 1 NON LEUK	CCDC71L
CDKN2B	4.66E-09	0.255693171	0.188	0.01	1.71E-04	CD44 > 1 & MKI67 < 1 NON LEUK	CDKN2B
CD300E	4.91E-09	0.37969401	0.275	0.058	1.80E-04	CD44 > 1 & MKI67 < 1 NON LEUK	CD300E
TJP2	6.32E-09	0.267406716	0.239	0.037	2.31E-04	CD44 > 1 & MKI67 < 1 NON LEUK	TJP2
IL3RA	7.08E-09	0.314244898	0.224	0.031	2.59E-04	CD44 > 1 & MKI67 < 1 NON LEUK	IL3RA
LST1	7.31E-09	0.226588266	0.486	0.204	2.67E-04	CD44 > 1 & MKI67 < 1 NON LEUK	LST1
ACSL1	7.32E-09	0.288114016	0.314	0.079	2.68E-04	CD44 > 1 & MKI67 < 1 NON LEUK	ACSL1
ODF3B	7.85E-09	0.37614731	0.396	0.136	2.87E-04	CD44 > 1 & MKI67 < 1 NON LEUK	ODF3B
ID2	1.02E-08	0.423860375	0.58	0.277	3.74E-04	CD44 > 1 & MKI67 < 1 NON LEUK	ID2
AHR	1.05E-08	0.310793376	0.345	0.099	3.83E-04	CD44 > 1 & MKI67 < 1 NON LEUK	AHR
TPI1	1.08E-08	0.395672342	0.537	0.241	3.94E-04	CD44 > 1 & MKI67 < 1 NON LEUK	TPI1
BNIP3L	1.16E-08	0.447094211	0.443	0.188	4.24E-04	CD44 > 1 & MKI67 < 1 NON LEUK	BNIP3L
LCP1	1.20E-08	0.465706265	0.553	0.246	4.38E-04	CD44 > 1 & MKI67 < 1 NON LEUK	LCP1
AC007032.1	1.24E-08	0.278122772	0.169	0.005	4.56E-04	CD44 > 1 & MKI67 < 1 NON LEUK	AC007032.1
PIM3	1.41E-08	0.397444023	0.51	0.225	5.15E-04	CD44 > 1 & MKI67 < 1 NON LEUK	PIM3
RNF145	1.42E-08	0.352043706	0.58	0.277	5.19E-04	CD44 > 1 & MKI67 < 1 NON LEUK	RNF145
SERPINB1	1.60E-08	0.360540718	0.471	0.199	5.84E-04	CD44 > 1 & MKI67 < 1 NON LEUK	SERPINB1
PRDM1	1.61E-08	0.241507335	0.325	0.089	5.89E-04	CD44 > 1 & MKI67 < 1 NON LEUK	PRDM1
SLC11A1	1.74E-08	0.290245528	0.231	0.037	6.36E-04	CD44 > 1 & MKI67 < 1 NON LEUK	SLC11A1
CASP1	1.97E-08	0.231115852	0.212	0.026	7.20E-04	CD44 > 1 & MKI67 < 1 NON LEUK	CASP1
LGALS2	2.01E-08	0.252491868	0.208	0.026	7.37E-04	CD44 > 1 & MKI67 < 1 NON LEUK	LGALS2
STX11	2.06E-08	0.290031497	0.4	0.136	7.54E-04	CD44 > 1 & MKI67 < 1 NON LEUK	STX11
ECE1	2.11E-08	0.321987096	0.345	0.11	7.72E-04	CD44 > 1 & MKI67 < 1 NON LEUK	ECE1
MYC	2.32E-08	0.328320788	0.408	0.152	8.50E-04	CD44 > 1 & MKI67 < 1 NON LEUK	MYC
HOTAIRM1	2.62E-08	0.29259503	0.251	0.052	9.58E-04	CD44 > 1 & MKI67 < 1 NON LEUK	HOTAIRM1
MIR22HG	2.63E-08	0.274523172	0.271	0.063	9.61E-04	CD44 > 1 & MKI67 < 1 NON LEUK	MIR22HG
CTNNB1	2.69E-08	0.341955983	0.333	0.105	9.84E-04	CD44 > 1 & MKI67 < 1 NON LEUK	CTNNB1
GCH1	2.79E-08	0.375399902	0.322	0.094	1.02E-03	CD44 > 1 & MKI67 < 1 NON LEUK	GCH1
PRKAG2-AS1	2.88E-08	0.220845822	0.208	0.026	1.05E-03	CD44 > 1 & MKI67 < 1 NON LEUK	PRKAG2-AS1
SLC15A3	2.97E-08	0.251193928	0.224	0.037	1.09E-03	CD44 > 1 & MKI67 < 1 NON LEUK	SLC15A3
TKT	3.03E-08	0.417507686	0.557	0.257	1.11E-03	CD44 > 1 & MKI67 < 1 NON LEUK	TKT
ETV3	3.18E-08	0.264740203	0.259	0.058	1.17E-03	CD44 > 1 & MKI67 < 1 NON LEUK	ETV3
SCPEP1	3.32E-08	0.219653129	0.255	0.052	1.21E-03	CD44 > 1 & MKI67 < 1 NON LEUK	SCPEP1

NEDD4L	3.34E-08	0.265082516	0.255	0.052	1.22E-03	CD44 > 1 & MKI67 < 1 NON LEUK	NEDD4L
CFL1	3.34E-08	0.392439983	0.851	0.691	1.22E-03	CD44 > 1 & MKI67 < 1 NON LEUK	CFL1
PILRA	3.80E-08	0.267765314	0.255	0.052	1.39E-03	CD44 > 1 & MKI67 < 1 NON LEUK	PILRA
FNIP2	3.92E-08	0.282861817	0.247	0.052	1.43E-03	CD44 > 1 & MKI67 < 1 NON LEUK	FNIP2
SIRPA	4.02E-08	0.255562363	0.239	0.047	1.47E-03	CD44 > 1 & MKI67 < 1 NON LEUK	SIRPA
TLR2	4.31E-08	0.213634985	0.18	0.016	1.58E-03	CD44 > 1 & MKI67 < 1 NON LEUK	TLR2
LHFPL2	4.43E-08	0.225291702	0.235	0.042	1.62E-03	CD44 > 1 & MKI67 < 1 NON LEUK	LHFPL2
MFSD2A	4.65E-08	0.235535064	0.22	0.037	1.70E-03	CD44 > 1 & MKI67 < 1 NON LEUK	MFSD2A
NEAT1	4.79E-08	0.451083237	0.545	0.251	1.75E-03	CD44 > 1 & MKI67 < 1 NON LEUK	NEAT1
HIF1A	4.80E-08	0.408560264	0.573	0.277	1.76E-03	CD44 > 1 & MKI67 < 1 NON LEUK	HIF1A
LYN	5.59E-08	0.287578723	0.322	0.094	2.05E-03	CD44 > 1 & MKI67 < 1 NON LEUK	LYN
ARFGAP3	5.77E-08	0.268081085	0.369	0.126	2.11E-03	CD44 > 1 & MKI67 < 1 NON LEUK	ARFGAP3
HLA-DPA1	6.13E-08	0.593546846	0.502	0.241	2.24E-03	CD44 > 1 & MKI67 < 1 NON LEUK	HLA-DPA1
IL6R	6.14E-08	0.236674546	0.239	0.047	2.25E-03	CD44 > 1 & MKI67 < 1 NON LEUK	IL6R
HLA-DPB1	6.30E-08	0.443425681	0.471	0.204	2.31E-03	CD44 > 1 & MKI67 < 1 NON LEUK	HLA-DPB1
LILRB4	6.52E-08	0.218996084	0.216	0.037	2.38E-03	CD44 > 1 & MKI67 < 1 NON LEUK	LILRB4
LILRA5	6.74E-08	0.215664665	0.208	0.031	2.47E-03	CD44 > 1 & MKI67 < 1 NON LEUK	LILRA5
SERTAD2	6.79E-08	0.224918571	0.247	0.052	2.48E-03	CD44 > 1 & MKI67 < 1 NON LEUK	SERTAD2
MAP1LC3B	6.82E-08	0.34634978	0.506	0.215	2.50E-03	CD44 > 1 & MKI67 < 1 NON LEUK	MAP1LC3B
CDA	6.85E-08	0.31988468	0.255	0.063	2.51E-03	CD44 > 1 & MKI67 < 1 NON LEUK	CDA
HLX	6.87E-08	0.221623094	0.196	0.026	2.52E-03	CD44 > 1 & MKI67 < 1 NON LEUK	HLX
ARL8B	7.51E-08	0.296968647	0.408	0.157	2.75E-03	CD44 > 1 & MKI67 < 1 NON LEUK	ARL8B
LILRB2	7.55E-08	0.303422452	0.267	0.068	2.77E-03	CD44 > 1 & MKI67 < 1 NON LEUK	LILRB2
AC004130.2	7.80E-08	0.209543815	0.184	0.021	2.85E-03	CD44 > 1 & MKI67 < 1 NON LEUK	AC004130.2
SEMA6B	7.82E-08	0.294978832	0.231	0.047	2.86E-03	CD44 > 1 & MKI67 < 1 NON LEUK	SEMA6B
CES1	7.91E-08	0.238150576	0.173	0.016	2.90E-03	CD44 > 1 & MKI67 < 1 NON LEUK	CES1
ARPC5	8.99E-08	0.296640107	0.408	0.152	3.29E-03	CD44 > 1 & MKI67 < 1 NON LEUK	ARPC5
CHST2	9.05E-08	0.193358716	0.251	0.058	3.31E-03	CD44 > 1 & MKI67 < 1 NON LEUK	CHST2
MYL6	1.06E-07	0.410432531	0.851	0.639	3.87E-03	CD44 > 1 & MKI67 < 1 NON LEUK	MYL6
MAP2K3	1.09E-07	0.303518516	0.376	0.136	3.98E-03	CD44 > 1 & MKI67 < 1 NON LEUK	MAP2K3
TPM4	1.12E-07	0.419360235	0.514	0.251	4.11E-03	CD44 > 1 & MKI67 < 1 NON LEUK	TPM4
ITGB2	1.13E-07	0.347418952	0.573	0.293	4.12E-03	CD44 > 1 & MKI67 < 1 NON LEUK	ITGB2
VEGFA	1.15E-07	0.184860649	0.2	0.031	4.21E-03	CD44 > 1 & MKI67 < 1 NON LEUK	VEGFA
CASP4	1.23E-07	0.254245298	0.318	0.094	4.52E-03	CD44 > 1 & MKI67 < 1 NON LEUK	CASP4
FOXO3	1.32E-07	0.190026958	0.267	0.063	4.82E-03	CD44 > 1 & MKI67 < 1 NON LEUK	FOXO3
SLC7A5	1.34E-07	0.285923875	0.58	0.272	4.91E-03	CD44 > 1 & MKI67 < 1 NON LEUK	SLC7A5
B4GALT5	1.47E-07	0.204160413	0.243	0.052	5.37E-03	CD44 > 1 & MKI67 < 1 NON LEUK	B4GALT5
SLC2A6	1.48E-07	0.253987882	0.239	0.052	5.41E-03	CD44 > 1 & MKI67 < 1 NON LEUK	SLC2A6
IFIT3	1.48E-07	0.533181318	0.361	0.131	5.43E-03	CD44 > 1 & MKI67 < 1 NON LEUK	IFIT3
SLC25A6	1.52E-07	0.381345981	0.949	0.806	5.57E-03	CD44 > 1 & MKI67 < 1 NON LEUK	SLC25A6
SDCBP	1.62E-07	0.340982771	0.502	0.23	5.94E-03	CD44 > 1 & MKI67 < 1 NON LEUK	SDCBP
OASL	1.68E-07	0.416139936	0.725	0.45	6.16E-03	CD44 > 1 & MKI67 < 1 NON LEUK	OASL
AGTRAP	1.69E-07	0.185680533	0.224	0.042	6.19E-03	CD44 > 1 & MKI67 < 1 NON LEUK	AGTRAP
AL118516.1	1.82E-07	0.255956079	0.31	0.094	6.67E-03	CD44 > 1 & MKI67 < 1 NON LEUK	AL118516.1
TLR4	1.84E-07	0.181120039	0.145	0.005	6.74E-03	CD44 > 1 & MKI67 < 1 NON LEUK	TLR4
EIF1B	1.90E-07	0.512594945	0.533	0.288	6.94E-03	CD44 > 1 & MKI67 < 1 NON LEUK	EIF1B
PIK3AP1	1.92E-07	0.209222281	0.255	0.063	7.01E-03	CD44 > 1 & MKI67 < 1 NON LEUK	PIK3AP1
EPB41L3	2.15E-07	0.188609038	0.176	0.021	7.87E-03	CD44 > 1 & MKI67 < 1 NON LEUK	EPB41L3
EIF4A1	2.20E-07	0.414398261	0.855	0.592	8.04E-03	CD44 > 1 & MKI67 < 1 NON LEUK	EIF4A1
SAMSN1	2.22E-07	0.289956728	0.408	0.162	8.11E-03	CD44 > 1 & MKI67 < 1 NON LEUK	SAMSN1
TNFAIP8	2.26E-07	0.333557545	0.443	0.194	8.25E-03	CD44 > 1 & MKI67 < 1 NON LEUK	TNFAIP8
IQGAP1	2.33E-07	0.286532864	0.306	0.094	8.53E-03	CD44 > 1 & MKI67 < 1 NON LEUK	IQGAP1
GADD45A	2.55E-07	0.39303975	0.455	0.209	9.33E-03	CD44 > 1 & MKI67 < 1 NON LEUK	GADD45A
MXD1	2.59E-07	0.292544726	0.263	0.073	9.48E-03	CD44 > 1 & MKI67 < 1 NON LEUK	MXD1

PLBD1	2.68E-07	0.200767534	0.188	0.026	9.83E-03	CD44 > 1 & MKI67 < 1 NON LEUK	PLBD1
MED13L	2.71E-07	0.278898103	0.29	0.089	9.91E-03	CD44 > 1 & MKI67 < 1 NON LEUK	MED13L
PLD3	2.75E-07	0.316412401	0.333	0.12	1.01E-02	CD44 > 1 & MKI67 < 1 NON LEUK	PLD3
SLCO3A1	3.06E-07	0.224535712	0.29	0.084	1.12E-02	CD44 > 1 & MKI67 < 1 NON LEUK	SLCO3A1
IRAK2	3.23E-07	0.228134189	0.224	0.052	1.18E-02	CD44 > 1 & MKI67 < 1 NON LEUK	IRAK2
CNPY3	3.34E-07	0.205148978	0.322	0.11	1.22E-02	CD44 > 1 & MKI67 < 1 NON LEUK	CNPY3
PRELID1	3.35E-07	0.328628373	0.443	0.199	1.23E-02	CD44 > 1 & MKI67 < 1 NON LEUK	PRELID1
PIK3R5	3.55E-07	0.245095868	0.247	0.063	1.30E-02	CD44 > 1 & MKI67 < 1 NON LEUK	PIK3R5
TBC1D12	4.81E-07	0.192126395	0.149	0.01	1.76E-02	CD44 > 1 & MKI67 < 1 NON LEUK	TBC1D12
ADAMTS2	5.06E-07	0.210279246	0.208	0.042	1.85E-02	CD44 > 1 & MKI67 < 1 NON LEUK	ADAMTS2
AP1S2	5.12E-07	0.19823424	0.22	0.047	1.87E-02	CD44 > 1 & MKI67 < 1 NON LEUK	AP1S2
CCL20	5.17E-07	0.414718544	0.18	0.026	1.89E-02	CD44 > 1 & MKI67 < 1 NON LEUK	CCL20
NFKB1	5.36E-07	0.384974206	0.667	0.398	1.96E-02	CD44 > 1 & MKI67 < 1 NON LEUK	NFKB1
IRAK1	5.47E-07	0.265565382	0.29	0.094	2.00E-02	CD44 > 1 & MKI67 < 1 NON LEUK	IRAK1
ADORA2A	5.68E-07	0.229013806	0.235	0.058	2.08E-02	CD44 > 1 & MKI67 < 1 NON LEUK	ADORA2A
QKI	6.18E-07	0.213488778	0.318	0.11	2.26E-02	CD44 > 1 & MKI67 < 1 NON LEUK	QKI
MIR4435-2HG	6.82E-07	0.260730819	0.345	0.126	2.50E-02	CD44 > 1 & MKI67 < 1 NON LEUK	MIR4435-2HG
TPST1	7.05E-07	0.236777664	0.216	0.047	2.58E-02	CD44 > 1 & MKI67 < 1 NON LEUK	TPST1
TIMP2	7.06E-07	0.24197186	0.29	0.094	2.59E-02	CD44 > 1 & MKI67 < 1 NON LEUK	TIMP2
C5AR1	7.70E-07	0.257179529	0.208	0.042	2.82E-02	CD44 > 1 & MKI67 < 1 NON LEUK	C5AR1
ARRB2	7.76E-07	0.213370857	0.38	0.147	2.84E-02	CD44 > 1 & MKI67 < 1 NON LEUK	ARRB2
MT-ND1	8.20E-07	0.27741347	0.984	0.958	3.00E-02	CD44 > 1 & MKI67 < 1 NON LEUK	MT-ND1
WARS	8.44E-07	0.239215084	0.271	0.079	3.09E-02	CD44 > 1 & MKI67 < 1 NON LEUK	WARS
PHLDA2	8.62E-07	0.196623599	0.165	0.021	3.15E-02	CD44 > 1 & MKI67 < 1 NON LEUK	PHLDA2
LMNA	8.80E-07	0.332246091	0.451	0.204	3.22E-02	CD44 > 1 & MKI67 < 1 NON LEUK	LMNA
CD14	9.11E-07	0.263233992	0.231	0.058	3.33E-02	CD44 > 1 & MKI67 < 1 NON LEUK	CD14
MAP4K4	9.15E-07	0.338786325	0.388	0.168	3.35E-02	CD44 > 1 & MKI67 < 1 NON LEUK	MAP4K4
WDR1	9.23E-07	0.274012423	0.506	0.251	3.38E-02	CD44 > 1 & MKI67 < 1 NON LEUK	WDR1
HS3ST3B1	9.31E-07	0.212327705	0.196	0.037	3.41E-02	CD44 > 1 & MKI67 < 1 NON LEUK	HS3ST3B1
FLOT1	9.66E-07	0.308145735	0.361	0.152	3.54E-02	CD44 > 1 & MKI67 < 1 NON LEUK	FLOT1
LRRFIP2	9.81E-07	0.198414424	0.298	0.094	3.59E-02	CD44 > 1 & MKI67 < 1 NON LEUK	LRRFIP2
FOSL2	9.82E-07	0.286828254	0.424	0.178	3.59E-02	CD44 > 1 & MKI67 < 1 NON LEUK	FOSL2
SEC11A	9.95E-07	0.255836048	0.42	0.178	3.64E-02	CD44 > 1 & MKI67 < 1 NON LEUK	SEC11A
ST3GAL1	1.01E-06	0.249090778	0.286	0.094	3.70E-02	CD44 > 1 & MKI67 < 1 NON LEUK	ST3GAL1
TPRA1	1.10E-06	0.282347811	0.247	0.073	4.01E-02	CD44 > 1 & MKI67 < 1 NON LEUK	TPRA1
LIMS1	1.15E-06	0.22329732	0.302	0.105	4.23E-02	CD44 > 1 & MKI67 < 1 NON LEUK	LIMS1
TRAF1	1.16E-06	0.267270328	0.255	0.073	4.25E-02	CD44 > 1 & MKI67 < 1 NON LEUK	TRAF1
RNF130	1.19E-06	0.273481509	0.322	0.12	4.37E-02	CD44 > 1 & MKI67 < 1 NON LEUK	RNF130
SNX8	1.20E-06	0.172314502	0.243	0.063	4.39E-02	CD44 > 1 & MKI67 < 1 NON LEUK	SNX8
FNDC3B	1.23E-06	0.204402026	0.22	0.052	4.50E-02	CD44 > 1 & MKI67 < 1 NON LEUK	FNDC3B
ATP6V0B	1.23E-06	0.269385552	0.49	0.225	4.52E-02	CD44 > 1 & MKI67 < 1 NON LEUK	ATP6V0B
PLSCR1	1.24E-06	0.29768826	0.361	0.147	4.55E-02	CD44 > 1 & MKI67 < 1 NON LEUK	PLSCR1
EHD4	1.26E-06	0.296647855	0.329	0.126	4.60E-02	CD44 > 1 & MKI67 < 1 NON LEUK	EHD4
PITPNA	1.34E-06	0.165229608	0.153	0.016	4.90E-02	CD44 > 1 & MKI67 < 1 NON LEUK	PITPNA
ETHE1	1.35E-06	0.215214719	0.29	0.094	4.95E-02	CD44 > 1 & MKI67 < 1 NON LEUK	ETHE1
RNF19B	1.40E-06	0.244298366	0.208	0.047	5.12E-02	CD44 > 1 & MKI67 < 1 NON LEUK	RNF19B
DAPK1	1.41E-06	0.179720986	0.161	0.021	5.15E-02	CD44 > 1 & MKI67 < 1 NON LEUK	DAPK1
MGAT1	1.47E-06	0.256640714	0.337	0.126	5.37E-02	CD44 > 1 & MKI67 < 1 NON LEUK	MGAT1
RHOA	1.48E-06	0.35625359	0.651	0.398	5.42E-02	CD44 > 1 & MKI67 < 1 NON LEUK	RHOA
ABHD17C	1.51E-06	0.155465701	0.137	0.01	5.53E-02	CD44 > 1 & MKI67 < 1 NON LEUK	ABHD17C
FLNA	1.52E-06	0.250670634	0.404	0.173	5.57E-02	CD44 > 1 & MKI67 < 1 NON LEUK	FLNA
VIM-AS1	1.57E-06	0.199799906	0.192	0.037	5.76E-02	CD44 > 1 & MKI67 < 1 NON LEUK	VIM-AS1
ZNF385A	1.59E-06	0.168244539	0.239	0.063	5.82E-02	CD44 > 1 & MKI67 < 1 NON LEUK	ZNF385A
SOCS3	1.62E-06	0.245436936	0.302	0.105	5.94E-02	CD44 > 1 & MKI67 < 1 NON LEUK	SOCS3

LY86	1.65E-06	0.1818243	0.227	0.058	6.02E-02	CD44 > 1 & MKI67 < 1 NON LEUK	LY86
DUSP5	1.66E-06	0.199640504	0.29	0.089	6.06E-02	CD44 > 1 & MKI67 < 1 NON LEUK	DUSP5
GPX4	1.67E-06	0.385707927	0.643	0.398	6.11E-02	CD44 > 1 & MKI67 < 1 NON LEUK	GPX4
HLA-DMA	1.70E-06	0.346909356	0.369	0.157	6.21E-02	CD44 > 1 & MKI67 < 1 NON LEUK	HLA-DMA
ELL2	1.73E-06	0.34941482	0.447	0.22	6.32E-02	CD44 > 1 & MKI67 < 1 NON LEUK	ELL2
COTL1	1.85E-06	0.250110384	0.624	0.361	6.77E-02	CD44 > 1 & MKI67 < 1 NON LEUK	COTL1
SPAG9	1.94E-06	0.176060465	0.263	0.079	7.10E-02	CD44 > 1 & MKI67 < 1 NON LEUK	SPAG9
MARCKSL1	1.97E-06	0.344752481	0.416	0.194	7.21E-02	CD44 > 1 & MKI67 < 1 NON LEUK	MARCKSL1
RAB32	2.29E-06	0.195645726	0.255	0.079	8.39E-02	CD44 > 1 & MKI67 < 1 NON LEUK	RAB32
B3GNT5	2.36E-06	0.193844891	0.204	0.047	8.65E-02	CD44 > 1 & MKI67 < 1 NON LEUK	B3GNT5
TMED5	2.40E-06	0.207455401	0.259	0.084	8.79E-02	CD44 > 1 & MKI67 < 1 NON LEUK	TMED5
CHST7	2.43E-06	0.190924013	0.145	0.016	8.89E-02	CD44 > 1 & MKI67 < 1 NON LEUK	CHST7
CD302	2.56E-06	0.190342491	0.192	0.042	9.36E-02	CD44 > 1 & MKI67 < 1 NON LEUK	CD302
NPC1	2.58E-06	0.158721339	0.133	0.01	9.44E-02	CD44 > 1 & MKI67 < 1 NON LEUK	NPC1
RAB31	2.61E-06	0.12893212	0.169	0.026	9.55E-02	CD44 > 1 & MKI67 < 1 NON LEUK	RAB31
ISG15	2.70E-06	0.847949146	0.463	0.246	9.87E-02	CD44 > 1 & MKI67 < 1 NON LEUK	ISG15
LILRA2	2.84E-06	0.180352218	0.204	0.047	1.04E-01	CD44 > 1 & MKI67 < 1 NON LEUK	LILRA2
ZMIZ1	2.97E-06	0.242668005	0.271	0.094	1.09E-01	CD44 > 1 & MKI67 < 1 NON LEUK	ZMIZ1
MT-CO1	3.43E-06	0.253697991	1	1	1.25E-01	CD44 > 1 & MKI67 < 1 NON LEUK	MT-CO1
CCL2	3.45E-06	0.437752402	0.153	0.021	1.26E-01	CD44 > 1 & MKI67 < 1 NON LEUK	CCL2
CD109	3.48E-06	0.183366536	0.22	0.058	1.27E-01	CD44 > 1 & MKI67 < 1 NON LEUK	CD109
KLF4	3.51E-06	0.214730072	0.18	0.037	1.29E-01	CD44 > 1 & MKI67 < 1 NON LEUK	KLF4
UFM1	3.62E-06	0.231790582	0.361	0.152	1.33E-01	CD44 > 1 & MKI67 < 1 NON LEUK	UFM1
SLC7A11	3.75E-06	0.198578014	0.141	0.016	1.37E-01	CD44 > 1 & MKI67 < 1 NON LEUK	SLC7A11
GBP2	3.84E-06	0.227715604	0.255	0.079	1.41E-01	CD44 > 1 & MKI67 < 1 NON LEUK	GBP2
HMGA1	3.94E-06	0.390693926	0.576	0.314	1.44E-01	CD44 > 1 & MKI67 < 1 NON LEUK	HMGA1
KCNJ2-AS1	4.04E-06	0.14609127	0.118	0.005	1.48E-01	CD44 > 1 & MKI67 < 1 NON LEUK	KCNJ2-AS1
HS3ST1	4.21E-06	0.206716418	0.161	0.026	1.54E-01	CD44 > 1 & MKI67 < 1 NON LEUK	HS3ST1
AK4	4.23E-06	0.188106635	0.224	0.063	1.55E-01	CD44 > 1 & MKI67 < 1 NON LEUK	AK4
MAN2B1	4.29E-06	0.222673728	0.31	0.12	1.57E-01	CD44 > 1 & MKI67 < 1 NON LEUK	MAN2B1
ADA	4.29E-06	0.209315093	0.271	0.094	1.57E-01	CD44 > 1 & MKI67 < 1 NON LEUK	ADA
ZNFX1	4.40E-06	0.178780964	0.298	0.105	1.61E-01	CD44 > 1 & MKI67 < 1 NON LEUK	ZNFX1
GPRC5A	4.49E-06	0.129769207	0.118	0.005	1.64E-01	CD44 > 1 & MKI67 < 1 NON LEUK	GPRC5A
TNFAIP2	4.56E-06	0.173088764	0.161	0.026	1.67E-01	CD44 > 1 & MKI67 < 1 NON LEUK	TNFAIP2
PICALM	4.63E-06	0.187656756	0.196	0.047	1.69E-01	CD44 > 1 & MKI67 < 1 NON LEUK	PICALM
CREB5	4.91E-06	0.167352502	0.125	0.01	1.80E-01	CD44 > 1 & MKI67 < 1 NON LEUK	CREB5
OTUD1	4.93E-06	0.189853606	0.2	0.047	1.80E-01	CD44 > 1 & MKI67 < 1 NON LEUK	OTUD1
GRAMD2B	5.29E-06	0.154492067	0.161	0.026	1.94E-01	CD44 > 1 & MKI67 < 1 NON LEUK	GRAMD2B
SLC43A3	5.70E-06	0.170889867	0.165	0.031	2.09E-01	CD44 > 1 & MKI67 < 1 NON LEUK	SLC43A3
TENT5A	5.84E-06	0.17453619	0.239	0.073	2.14E-01	CD44 > 1 & MKI67 < 1 NON LEUK	TENT5A
CFLAR	6.01E-06	0.248810185	0.408	0.188	2.20E-01	CD44 > 1 & MKI67 < 1 NON LEUK	CFLAR
LINC-PINT	6.02E-06	0.184154247	0.188	0.042	2.20E-01	CD44 > 1 & MKI67 < 1 NON LEUK	LINC-PINT
RGS10	6.06E-06	0.213492521	0.408	0.183	2.22E-01	CD44 > 1 & MKI67 < 1 NON LEUK	RGS10
IFI27L2	6.54E-06	0.160932944	0.176	0.037	2.39E-01	CD44 > 1 & MKI67 < 1 NON LEUK	IFI27L2
TP53INP2	6.74E-06	0.135773667	0.137	0.016	2.47E-01	CD44 > 1 & MKI67 < 1 NON LEUK	TP53INP2
SNX9	6.85E-06	0.36952664	0.573	0.356	2.51E-01	CD44 > 1 & MKI67 < 1 NON LEUK	SNX9
TGIF1	7.36E-06	0.176681136	0.282	0.094	2.69E-01	CD44 > 1 & MKI67 < 1 NON LEUK	TGIF1
ATP6V1B2	7.42E-06	0.180733633	0.204	0.052	2.71E-01	CD44 > 1 & MKI67 < 1 NON LEUK	ATP6V1B2
MT2A	7.44E-06	0.600096327	0.643	0.414	2.72E-01	CD44 > 1 & MKI67 < 1 NON LEUK	MT2A
XBP1	7.48E-06	0.264580866	0.557	0.309	2.74E-01	CD44 > 1 & MKI67 < 1 NON LEUK	XBP1
KLF6	7.54E-06	0.261743579	0.808	0.56	2.76E-01	CD44 > 1 & MKI67 < 1 NON LEUK	KLF6
C15orf39	7.68E-06	0.161416975	0.204	0.052	2.81E-01	CD44 > 1 & MKI67 < 1 NON LEUK	C15orf39
C1orf122	7.70E-06	0.224231392	0.325	0.131	2.82E-01	CD44 > 1 & MKI67 < 1 NON LEUK	C1orf122
LAIR1	8.01E-06	0.237303847	0.278	0.099	2.93E-01	CD44 > 1 & MKI67 < 1 NON LEUK	LAIR1

HCK	8.68E-06	0.120907463	0.188	0.042	3.18E-01	CD44 > 1 & MKI67 < 1 NON LEUK	HCK
ATOX1	8.72E-06	0.181157692	0.18	0.042	3.19E-01	CD44 > 1 & MKI67 < 1 NON LEUK	ATOX1
ATF5	8.89E-06	0.174696773	0.18	0.042	3.25E-01	CD44 > 1 & MKI67 < 1 NON LEUK	ATF5
SMPDL3A	9.28E-06	0.141099387	0.122	0.01	0.339562061	CD44 > 1 & MKI67 < 1 NON LEUK	SMPDL3A
MAFG	9.39E-06	0.152720458	0.153	0.026	0.343637917	CD44 > 1 & MKI67 < 1 NON LEUK	MAFG
RAPGEF1	9.77E-06	0.187774567	0.22	0.063	0.357709052	CD44 > 1 & MKI67 < 1 NON LEUK	RAPGEF1
CCR1	9.84E-06	0.20382256	0.153	0.026	0.360006466	CD44 > 1 & MKI67 < 1 NON LEUK	CCR1
CRTAP	1.04E-05	0.196047699	0.349	0.152	0.381244901	CD44 > 1 & MKI67 < 1 NON LEUK	CRTAP
LRP1	1.07E-05	0.150028905	0.161	0.031	0.392048176	CD44 > 1 & MKI67 < 1 NON LEUK	LRP1
FKBP1A	1.14E-05	0.193612854	0.325	0.131	0.41611528	CD44 > 1 & MKI67 < 1 NON LEUK	FKBP1A
WDR33	1.14E-05	0.215674844	0.443	0.209	0.416745184	CD44 > 1 & MKI67 < 1 NON LEUK	WDR33
GNA13	1.16E-05	0.208831368	0.388	0.178	0.425940211	CD44 > 1 & MKI67 < 1 NON LEUK	GNA13
ENO1	1.18E-05	0.383096781	0.659	0.45	0.433445369	CD44 > 1 & MKI67 < 1 NON LEUK	ENO1
SGK1	1.19E-05	0.263661222	0.365	0.162	0.434600766	CD44 > 1 & MKI67 < 1 NON LEUK	SGK1
TCN2	1.20E-05	0.123917153	0.141	0.021	0.438975949	CD44 > 1 & MKI67 < 1 NON LEUK	TCN2
PNPLA8	1.23E-05	0.159985814	0.231	0.068	0.449925519	CD44 > 1 & MKI67 < 1 NON LEUK	PNPLA8
MYO10	1.36E-05	0.158196992	0.129	0.016	0.497255083	CD44 > 1 & MKI67 < 1 NON LEUK	MYO10
ANPEP	1.37E-05	0.149000342	0.137	0.021	0.500989799	CD44 > 1 & MKI67 < 1 NON LEUK	ANPEP
VCAN	1.37E-05	0.111176319	0.161	0.031	0.502935755	CD44 > 1 & MKI67 < 1 NON LEUK	VCAN
AOAH	1.38E-05	0.149020351	0.161	0.031	0.506140466	CD44 > 1 & MKI67 < 1 NON LEUK	AOAH
QSOX1	1.39E-05	0.155056228	0.157	0.031	0.508456231	CD44 > 1 & MKI67 < 1 NON LEUK	QSOX1
MREG	1.45E-05	0.172256817	0.184	0.047	0.530964117	CD44 > 1 & MKI67 < 1 NON LEUK	MREG
MDM2	1.54E-05	0.16221954	0.137	0.021	0.563241929	CD44 > 1 & MKI67 < 1 NON LEUK	MDM2
CAMTA2	1.54E-05	0.151729874	0.106	0.005	0.563914932	CD44 > 1 & MKI67 < 1 NON LEUK	CAMTA2
FCAR	1.57E-05	0.20525503	0.208	0.063	0.573747989	CD44 > 1 & MKI67 < 1 NON LEUK	FCAR
IRF7	1.58E-05	0.271051979	0.333	0.147	0.577077747	CD44 > 1 & MKI67 < 1 NON LEUK	IRF7
EMP3	1.72E-05	0.371181084	0.8	0.597	0.62939104	CD44 > 1 & MKI67 < 1 NON LEUK	EMP3
HIVEP2	1.77E-05	0.156460984	0.169	0.037	0.646448154	CD44 > 1 & MKI67 < 1 NON LEUK	HIVEP2
CYP1B1	1.77E-05	0.162738864	0.157	0.031	0.647764304	CD44 > 1 & MKI67 < 1 NON LEUK	CYP1B1
LCP2	1.81E-05	0.262118761	0.522	0.283	0.663261386	CD44 > 1 & MKI67 < 1 NON LEUK	LCP2
BCAT1	1.84E-05	0.17542075	0.184	0.047	0.674376921	CD44 > 1 & MKI67 < 1 NON LEUK	BCAT1
AC007952.4	1.85E-05	0.14619304	0.157	0.031	0.677110471	CD44 > 1 & MKI67 < 1 NON LEUK	AC007952.4
CYBB	1.87E-05	0.138567339	0.204	0.058	0.684509934	CD44 > 1 & MKI67 < 1 NON LEUK	CYBB
PLD1	1.92E-05	0.139412183	0.137	0.021	0.700952982	CD44 > 1 & MKI67 < 1 NON LEUK	PLD1
MARCO	2.09E-05	0.165937941	0.114	0.01	0.763503205	CD44 > 1 & MKI67 < 1 NON LEUK	MARCO
VASP	2.11E-05	0.170278062	0.298	0.115	0.774020931	CD44 > 1 & MKI67 < 1 NON LEUK	VASP
RAP1B	2.15E-05	0.241328896	0.6	0.33	0.786646203	CD44 > 1 & MKI67 < 1 NON LEUK	RAP1B
AC006449.6	2.17E-05	0.158599008	0.145	0.026	0.795845822	CD44 > 1 & MKI67 < 1 NON LEUK	AC006449.6
CTSZ	2.19E-05	0.186738825	0.259	0.094	0.802853053	CD44 > 1 & MKI67 < 1 NON LEUK	CTSZ
IGSF6	2.26E-05	0.14841562	0.157	0.031	0.828155308	CD44 > 1 & MKI67 < 1 NON LEUK	IGSF6
VOPP1	2.27E-05	0.189384174	0.216	0.068	0.829252891	CD44 > 1 & MKI67 < 1 NON LEUK	VOPP1
AZIN1	2.43E-05	0.186665256	0.227	0.073	0.887611459	CD44 > 1 & MKI67 < 1 NON LEUK	AZIN1
ARPC1B	2.45E-05	0.23160624	0.514	0.272	0.897315572	CD44 > 1 & MKI67 < 1 NON LEUK	ARPC1B
AMPD3	2.48E-05	0.142994995	0.157	0.031	0.909523808	CD44 > 1 & MKI67 < 1 NON LEUK	AMPD3
GNA15	2.50E-05	0.261948853	0.514	0.272	0.914032792	CD44 > 1 & MKI67 < 1 NON LEUK	GNA15
STAB1	2.52E-05	0.101165418	0.114	0.01	0.920801593	CD44 > 1 & MKI67 < 1 NON LEUK	STAB1
CSF3	2.59E-05	0.255782413	0.133	0.021	0.948771222	CD44 > 1 & MKI67 < 1 NON LEUK	CSF3
RILPL2	2.60E-05	0.266681274	0.443	0.23	0.953254304	CD44 > 1 & MKI67 < 1 NON LEUK	RILPL2
PRDM8	2.69E-05	0.219990067	0.227	0.079	0.98483504	CD44 > 1 & MKI67 < 1 NON LEUK	PRDM8
YWHAE	2.75E-05	0.206037759	0.282	0.115	1	CD44 > 1 & MKI67 < 1 NON LEUK	YWHAE
TNFRSF21	2.76E-05	0.172837425	0.18	0.047	1	CD44 > 1 & MKI67 < 1 NON LEUK	TNFRSF21
EIF5	2.79E-05	0.272729417	0.647	0.414	1	CD44 > 1 & MKI67 < 1 NON LEUK	EIF5
TNF	2.88E-05	0.275635577	0.18	0.047	1	CD44 > 1 & MKI67 < 1 NON LEUK	TNF
RNASET2	2.94E-05	0.359323221	0.561	0.356	1	CD44 > 1 & MKI67 < 1 NON LEUK	RNASET2

MANBA	2.94E-05	0.17744698	0.255	0.094	1	CD44 > 1 & MKI67 < 1 NON LEUK	MANBA
PHACTR1	3.11E-05	0.178627853	0.204	0.063	1	CD44 > 1 & MKI67 < 1 NON LEUK	PHACTR1
ALDH2	3.16E-05	0.176231153	0.176	0.047	1	CD44 > 1 & MKI67 < 1 NON LEUK	ALDH2
TLE1	3.21E-05	0.134303804	0.145	0.026	1	CD44 > 1 & MKI67 < 1 NON LEUK	TLE1
SNX10	3.28E-05	0.148568767	0.188	0.052	1	CD44 > 1 & MKI67 < 1 NON LEUK	SNX10
P2RX4	3.60E-05	0.161687333	0.169	0.042	1	CD44 > 1 & MKI67 < 1 NON LEUK	P2RX4
SLC6A6	3.60E-05	0.165417395	0.188	0.052	1	CD44 > 1 & MKI67 < 1 NON LEUK	SLC6A6
ANKLE2	3.78E-05	0.182361945	0.325	0.141	1	CD44 > 1 & MKI67 < 1 NON LEUK	ANKLE2
ABL2	3.82E-05	0.142926354	0.161	0.037	1	CD44 > 1 & MKI67 < 1 NON LEUK	ABL2
STON2	3.96E-05	0.124747219	0.129	0.021	1	CD44 > 1 & MKI67 < 1 NON LEUK	STON2
IL1A	4.12E-05	0.156083788	0.129	0.021	1	CD44 > 1 & MKI67 < 1 NON LEUK	IL1A
MPZL1	4.33E-05	0.10528366	0.129	0.021	1	CD44 > 1 & MKI67 < 1 NON LEUK	MPZL1
EIF4E	4.95E-05	0.23031982	0.255	0.099	1	CD44 > 1 & MKI67 < 1 NON LEUK	EIF4E
NOTCH1	4.95E-05	0.112244347	0.106	0.01	1	CD44 > 1 & MKI67 < 1 NON LEUK	NOTCH1
ASAH1	4.96E-05	0.163785372	0.298	0.12	1	CD44 > 1 & MKI67 < 1 NON LEUK	ASAH1
PTPN12	5.01E-05	0.145510196	0.192	0.058	1	CD44 > 1 & MKI67 < 1 NON LEUK	PTPN12
TPD52L2	5.01E-05	0.15554888	0.22	0.073	1	CD44 > 1 & MKI67 < 1 NON LEUK	TPD52L2
ALG2	5.17E-05	0.171860281	0.216	0.073	1	CD44 > 1 & MKI67 < 1 NON LEUK	ALG2
ACTB	5.22E-05	0.219524585	0.969	0.948	1	CD44 > 1 & MKI67 < 1 NON LEUK	ACTB
CAPG	5.23E-05	0.21274455	0.235	0.089	1	CD44 > 1 & MKI67 < 1 NON LEUK	CAPG
CSF2	5.33E-05	0.305610952	0.106	0.01	1	CD44 > 1 & MKI67 < 1 NON LEUK	CSF2
FAM49A	5.38E-05	0.17025937	0.165	0.042	1	CD44 > 1 & MKI67 < 1 NON LEUK	FAM49A
H3F3A	5.44E-05	0.278171467	0.898	0.791	1	CD44 > 1 & MKI67 < 1 NON LEUK	H3F3A
CD53	5.49E-05	0.162467344	0.369	0.168	1	CD44 > 1 & MKI67 < 1 NON LEUK	CD53
LINC00884	5.56E-05	0.119181733	0.118	0.016	1	CD44 > 1 & MKI67 < 1 NON LEUK	LINC00884
PPP3CC	5.62E-05	0.127287457	0.247	0.089	1	CD44 > 1 & MKI67 < 1 NON LEUK	PPP3CC
OXSR1	5.66E-05	0.166541458	0.259	0.105	1	CD44 > 1 & MKI67 < 1 NON LEUK	OXSR1
NRIP3	5.68E-05	0.148042077	0.125	0.021	1	CD44 > 1 & MKI67 < 1 NON LEUK	NRIP3
RABGEF1	5.73E-05	0.155081812	0.2	0.063	1	CD44 > 1 & MKI67 < 1 NON LEUK	RABGEF1
ARPC2	5.99E-05	0.304610251	0.725	0.471	1	CD44 > 1 & MKI67 < 1 NON LEUK	ARPC2
ALOX5AP	5.99E-05	0.207235515	0.278	0.11	1	CD44 > 1 & MKI67 < 1 NON LEUK	ALOX5AP
TSC22D2	6.31E-05	0.1299384	0.184	0.052	1	CD44 > 1 & MKI67 < 1 NON LEUK	TSC22D2
MS4A6A	6.53E-05	0.131772583	0.125	0.021	1	CD44 > 1 & MKI67 < 1 NON LEUK	MS4A6A
MAPK6	6.58E-05	0.186721939	0.259	0.105	1	CD44 > 1 & MKI67 < 1 NON LEUK	MAPK6
ZADH2	6.72E-05	0.125756521	0.137	0.026	1	CD44 > 1 & MKI67 < 1 NON LEUK	ZADH2
CD82	7.36E-05	0.230396078	0.365	0.183	1	CD44 > 1 & MKI67 < 1 NON LEUK	CD82
TNFRSF10B	7.38E-05	0.112987344	0.184	0.052	1	CD44 > 1 & MKI67 < 1 NON LEUK	TNFRSF10B
CAPNS1	7.44E-05	0.193851012	0.314	0.141	1	CD44 > 1 & MKI67 < 1 NON LEUK	CAPNS1
KDM7A-DT	7.73E-05	0.12218924	0.114	0.016	1	CD44 > 1 & MKI67 < 1 NON LEUK	KDM7A-DT
DLL1	7.95E-05	0.11472233	0.102	0.01	1	CD44 > 1 & MKI67 < 1 NON LEUK	DLL1
FAM126A	7.96E-05	0.132612219	0.133	0.026	1	CD44 > 1 & MKI67 < 1 NON LEUK	FAM126A
RUNX1	8.27E-05	0.151126006	0.29	0.131	1	CD44 > 1 & MKI67 < 1 NON LEUK	RUNX1
MYOF	8.31E-05	0.111694546	0.102	0.01	1	CD44 > 1 & MKI67 < 1 NON LEUK	MYOF
FH	8.42E-05	0.110481527	0.133	0.026	1	CD44 > 1 & MKI67 < 1 NON LEUK	FH
AC009951.6	8.44E-05	0.107330121	0.102	0.01	1	CD44 > 1 & MKI67 < 1 NON LEUK	AC009951.6
ARPC3	8.97E-05	0.243184043	0.612	0.387	1	CD44 > 1 & MKI67 < 1 NON LEUK	ARPC3
PARVB	9.08E-05	0.110847483	0.133	0.026	1	CD44 > 1 & MKI67 < 1 NON LEUK	PARVB
MT-ND4	9.39E-05	0.268902178	0.89	0.869	1	CD44 > 1 & MKI67 < 1 NON LEUK	MT-ND4
FNIP1	9.59E-05	0.134114842	0.169	0.047	1	CD44 > 1 & MKI67 < 1 NON LEUK	FNIP1
RAPGEF2	9.99E-05	0.133001928	0.141	0.031	1	CD44 > 1 & MKI67 < 1 NON LEUK	RAPGEF2
PARP14	1.01E-04	0.206468053	0.227	0.089	1	CD44 > 1 & MKI67 < 1 NON LEUK	PARP14
CTSH	1.02E-04	0.139909662	0.247	0.094	1	CD44 > 1 & MKI67 < 1 NON LEUK	CTSH
RAB5A	1.03E-04	0.171130463	0.314	0.141	1	CD44 > 1 & MKI67 < 1 NON LEUK	RAB5A
LONRF1	1.04E-04	0.107582382	0.133	0.026	1	CD44 > 1 & MKI67 < 1 NON LEUK	LONRF1

AHNAK	1.04E-04	0.244567159	0.357	0.183	1	CD44 > 1 & MKI67 < 1 NON LEUK	AHNAK
TMEM158	1.04E-04	0.213116477	0.157	0.042	1	CD44 > 1 & MKI67 < 1 NON LEUK	TMEM158
SLA	1.08E-04	0.132006034	0.169	0.047	1	CD44 > 1 & MKI67 < 1 NON LEUK	SLA
WDFY1	1.09E-04	0.169859956	0.184	0.058	1	CD44 > 1 & MKI67 < 1 NON LEUK	WDFY1
IL10	1.12E-04	0.127550728	0.122	0.021	1	CD44 > 1 & MKI67 < 1 NON LEUK	IL10
HES4	1.14E-04	0.331084779	0.388	0.22	1	CD44 > 1 & MKI67 < 1 NON LEUK	HES4
BCAP31	1.15E-04	0.163150123	0.314	0.147	1	CD44 > 1 & MKI67 < 1 NON LEUK	BCAP31
NFKB2	1.18E-04	0.195122471	0.341	0.162	1	CD44 > 1 & MKI67 < 1 NON LEUK	NFKB2
SLC7A1	1.24E-04	0.110561908	0.176	0.052	1	CD44 > 1 & MKI67 < 1 NON LEUK	SLC7A1
LRRC25	1.26E-04	0.102270724	0.141	0.031	1	CD44 > 1 & MKI67 < 1 NON LEUK	LRRC25
LINC01588	1.28E-04	0.103165162	0.11	0.016	1	CD44 > 1 & MKI67 < 1 NON LEUK	LINC01588
CITED2	1.28E-04	0.194294523	0.345	0.168	1	CD44 > 1 & MKI67 < 1 NON LEUK	CITED2
CD93	1.31E-04	0.148568869	0.145	0.037	1	CD44 > 1 & MKI67 < 1 NON LEUK	CD93
TRAPPC5	1.32E-04	0.168529075	0.255	0.105	1	CD44 > 1 & MKI67 < 1 NON LEUK	TRAPPC5
CDKN2A	1.36E-04	0.125423893	0.11	0.016	1	CD44 > 1 & MKI67 < 1 NON LEUK	CDKN2A
AKIRIN2	1.38E-04	0.175194167	0.314	0.147	1	CD44 > 1 & MKI67 < 1 NON LEUK	AKIRIN2
NOTCH2NLC	1.41E-04	0.109120872	0.157	0.042	1	CD44 > 1 & MKI67 < 1 NON LEUK	NOTCH2NLC
ATF4	1.48E-04	0.258492979	0.518	0.304	1	CD44 > 1 & MKI67 < 1 NON LEUK	ATF4
PLEKHM2	1.52E-04	0.145738118	0.2	0.068	1	CD44 > 1 & MKI67 < 1 NON LEUK	PLEKHM2
NFAT5	1.54E-04	0.146591401	0.231	0.089	1	CD44 > 1 & MKI67 < 1 NON LEUK	NFAT5
IER3-AS1	1.56E-04	0.122738615	0.106	0.016	1	CD44 > 1 & MKI67 < 1 NON LEUK	IER3-AS1
EAF1	1.62E-04	0.127114372	0.125	0.026	1	CD44 > 1 & MKI67 < 1 NON LEUK	EAF1
HNRNPC	1.66E-04	0.258793705	0.737	0.545	1	CD44 > 1 & MKI67 < 1 NON LEUK	HNRNPC
CD151	1.68E-04	0.116163185	0.153	0.042	1	CD44 > 1 & MKI67 < 1 NON LEUK	CD151
CD58	1.68E-04	0.116999035	0.251	0.099	1	CD44 > 1 & MKI67 < 1 NON LEUK	CD58
GPR183	1.70E-04	0.243703187	0.322	0.152	1	CD44 > 1 & MKI67 < 1 NON LEUK	GPR183
LAPTM5	1.71E-04	0.148586759	0.902	0.749	1	CD44 > 1 & MKI67 < 1 NON LEUK	LAPTM5
STXBP2	1.77E-04	0.112695634	0.275	0.115	1	CD44 > 1 & MKI67 < 1 NON LEUK	STXBP2
OAS3	1.77E-04	0.159634843	0.192	0.068	1	CD44 > 1 & MKI67 < 1 NON LEUK	OAS3
CKB	1.78E-04	0.225091155	0.161	0.047	1	CD44 > 1 & MKI67 < 1 NON LEUK	CKB
SMIM3	1.79E-04	0.165738053	0.176	0.058	1	CD44 > 1 & MKI67 < 1 NON LEUK	SMIM3
HLA-DQA1	1.83E-04	0.11174057	0.208	0.073	1	CD44 > 1 & MKI67 < 1 NON LEUK	HLA-DQA1
TPP1	1.84E-04	0.109094255	0.157	0.042	1	CD44 > 1 & MKI67 < 1 NON LEUK	TPP1
TRIM25	1.85E-04	0.147877017	0.255	0.11	1	CD44 > 1 & MKI67 < 1 NON LEUK	TRIM25
RYBP	1.88E-04	0.156468366	0.184	0.063	1	CD44 > 1 & MKI67 < 1 NON LEUK	RYBP
GNA12	1.96E-04	0.106472789	0.169	0.052	1	CD44 > 1 & MKI67 < 1 NON LEUK	GNA12
MCRIP2	1.98E-04	0.125056486	0.216	0.079	1	CD44 > 1 & MKI67 < 1 NON LEUK	MCRIP2
GRB2	1.98E-04	0.183786862	0.494	0.288	1	CD44 > 1 & MKI67 < 1 NON LEUK	GRB2
DOT1L	2.01E-04	0.142322324	0.169	0.052	1	CD44 > 1 & MKI67 < 1 NON LEUK	DOT1L
KBTBD2	2.06E-04	0.143026803	0.216	0.079	1	CD44 > 1 & MKI67 < 1 NON LEUK	KBTBD2
CLIC1	2.08E-04	0.259477089	0.635	0.398	1	CD44 > 1 & MKI67 < 1 NON LEUK	CLIC1
SAMD9	2.10E-04	0.188883373	0.259	0.11	1	CD44 > 1 & MKI67 < 1 NON LEUK	SAMD9
CAST	2.16E-04	0.171855015	0.349	0.178	1	CD44 > 1 & MKI67 < 1 NON LEUK	CAST
SERPINB9P1	2.17E-04	0.130259323	0.114	0.021	1	CD44 > 1 & MKI67 < 1 NON LEUK	SERPINB9P1
ANO6	2.19E-04	0.117560176	0.133	0.031	1	CD44 > 1 & MKI67 < 1 NON LEUK	ANO6
SLC2A3	2.22E-04	0.244400547	0.537	0.314	1	CD44 > 1 & MKI67 < 1 NON LEUK	SLC2A3
FGFR1	2.23E-04	0.123267066	0.133	0.031	1	CD44 > 1 & MKI67 < 1 NON LEUK	FGFR1
CEBPB	2.23E-04	0.209308279	0.424	0.23	1	CD44 > 1 & MKI67 < 1 NON LEUK	CEBPB
OLR1	2.27E-04	0.142270261	0.122	0.026	1	CD44 > 1 & MKI67 < 1 NON LEUK	OLR1
MCTP1	2.28E-04	0.111934772	0.114	0.021	1	CD44 > 1 & MKI67 < 1 NON LEUK	MCTP1
CRIP1	2.33E-04	0.343958725	0.722	0.508	1	CD44 > 1 & MKI67 < 1 NON LEUK	CRIP1
AKAP13	2.34E-04	0.16091749	0.365	0.188	1	CD44 > 1 & MKI67 < 1 NON LEUK	AKAP13
MICOS10	2.37E-04	0.242370505	0.341	0.183	1	CD44 > 1 & MKI67 < 1 NON LEUK	MICOS10
DDX3X	2.43E-04	0.13548643	0.447	0.236	1	CD44 > 1 & MKI67 < 1 NON LEUK	DDX3X

ADAM15	2.49E-04	0.134714577	0.122	0.026	1	CD44 > 1 & MKI67 < 1 NON LEUK	ADAM15
SERF2	2.52E-04	0.276367592	0.831	0.691	1	CD44 > 1 & MKI67 < 1 NON LEUK	SERF2
PGD	2.54E-04	0.147787187	0.216	0.084	1	CD44 > 1 & MKI67 < 1 NON LEUK	PGD
PPP3CA	2.59E-04	0.118215954	0.169	0.052	1	CD44 > 1 & MKI67 < 1 NON LEUK	PPP3CA
ZYX	2.63E-04	0.20502531	0.435	0.246	1	CD44 > 1 & MKI67 < 1 NON LEUK	ZYX
RARA	2.64E-04	0.154318573	0.255	0.11	1	CD44 > 1 & MKI67 < 1 NON LEUK	RARA
GTPBP4	2.68E-04	0.141011391	0.345	0.168	1	CD44 > 1 & MKI67 < 1 NON LEUK	GTPBP4
GLUL	2.71E-04	0.23307378	0.341	0.183	1	CD44 > 1 & MKI67 < 1 NON LEUK	GLUL
IFIT2	2.73E-04	0.40392367	0.2	0.079	1	CD44 > 1 & MKI67 < 1 NON LEUK	IFIT2
ARHGAP21	2.77E-04	0.101208523	0.102	0.016	1	CD44 > 1 & MKI67 < 1 NON LEUK	ARHGAP21
UBE2R2	2.82E-04	0.159376064	0.235	0.099	1	CD44 > 1 & MKI67 < 1 NON LEUK	UBE2R2
MAD1L1	2.83E-04	0.215728311	0.239	0.099	1	CD44 > 1 & MKI67 < 1 NON LEUK	MAD1L1
NOCT	2.86E-04	0.132027723	0.129	0.031	1	CD44 > 1 & MKI67 < 1 NON LEUK	NOCT
PIK3CB	2.93E-04	0.102266762	0.176	0.058	1	CD44 > 1 & MKI67 < 1 NON LEUK	PIK3CB
ARHGEF2	3.00E-04	0.118016398	0.149	0.042	1	CD44 > 1 & MKI67 < 1 NON LEUK	ARHGEF2
PTEN	3.01E-04	0.133705955	0.216	0.089	1	CD44 > 1 & MKI67 < 1 NON LEUK	PTEN
TGFBI	3.10E-04	0.114725935	0.11	0.021	1	CD44 > 1 & MKI67 < 1 NON LEUK	TGFBI
MAML2	3.13E-04	0.112223923	0.169	0.052	1	CD44 > 1 & MKI67 < 1 NON LEUK	MAML2
PABPC4	3.17E-04	0.107886992	0.255	0.11	1	CD44 > 1 & MKI67 < 1 NON LEUK	PABPC4
IFITM3	3.24E-04	0.21356622	0.153	0.047	1	CD44 > 1 & MKI67 < 1 NON LEUK	IFITM3
NRIP1	3.30E-04	0.134467959	0.235	0.099	1	CD44 > 1 & MKI67 < 1 NON LEUK	NRIP1
UPP1	0.000336272	0.285800539	0.745	0.55	1	CD44 > 1 & MKI67 < 1 NON LEUK	UPP1
HK2	0.000339094	0.107038894	0.11	0.021	1	CD44 > 1 & MKI67 < 1 NON LEUK	HK2
CARD19	0.000339987	0.149797293	0.18	0.063	1	CD44 > 1 & MKI67 < 1 NON LEUK	CARD19
PLEKHO1	0.000341985	0.192634404	0.478	0.272	1	CD44 > 1 & MKI67 < 1 NON LEUK	PLEKHO1
TRIP10	0.000352659	0.148754059	0.153	0.047	1	CD44 > 1 & MKI67 < 1 NON LEUK	TRIP10
ST8SIA4	0.000365903	0.154476347	0.153	0.047	1	CD44 > 1 & MKI67 < 1 NON LEUK	ST8SIA4
SLC31A2	0.000377941	0.12258913	0.133	0.037	1	CD44 > 1 & MKI67 < 1 NON LEUK	SLC31A2
SEC61B	0.000392062	0.212350184	0.584	0.387	1	CD44 > 1 & MKI67 < 1 NON LEUK	SEC61B
MTF1	0.000399818	0.1044997	0.18	0.063	1	CD44 > 1 & MKI67 < 1 NON LEUK	MTF1
RHEB	0.000411182	0.203439448	0.416	0.23	1	CD44 > 1 & MKI67 < 1 NON LEUK	RHEB
MFSD12	0.000419718	0.10277648	0.125	0.031	1	CD44 > 1 & MKI67 < 1 NON LEUK	MFSD12
MYD88	0.000455081	0.112230686	0.271	0.12	1	CD44 > 1 & MKI67 < 1 NON LEUK	MYD88
H2AFY	0.000455429	0.193417074	0.431	0.251	1	CD44 > 1 & MKI67 < 1 NON LEUK	H2AFY
GADD45B	0.000457209	0.284733935	0.647	0.45	1	CD44 > 1 & MKI67 < 1 NON LEUK	GADD45B
LYPD3	0.000458787	0.247015526	0.251	0.115	1	CD44 > 1 & MKI67 < 1 NON LEUK	LYPD3
ACTR3	0.000465099	0.151143676	0.329	0.162	1	CD44 > 1 & MKI67 < 1 NON LEUK	ACTR3
NDST2	0.000477811	0.129987225	0.145	0.042	1	CD44 > 1 & MKI67 < 1 NON LEUK	NDST2
PDLIM7	0.000490504	0.132488815	0.106	0.021	1	CD44 > 1 & MKI67 < 1 NON LEUK	PDLIM7
ACSL3	0.000503801	0.144337199	0.212	0.084	1	CD44 > 1 & MKI67 < 1 NON LEUK	ACSL3
NR4A3	0.000511923	0.154744194	0.275	0.131	1	CD44 > 1 & MKI67 < 1 NON LEUK	NR4A3
PFN1	0.000513404	0.205192778	0.835	0.654	1	CD44 > 1 & MKI67 < 1 NON LEUK	PFN1
KMO	0.000515794	0.111861959	0.106	0.021	1	CD44 > 1 & MKI67 < 1 NON LEUK	KMO
SCO2	0.000524494	0.105430028	0.188	0.068	1	CD44 > 1 & MKI67 < 1 NON LEUK	SCO2
CDKN1A	0.000539715	0.140921469	0.4	0.215	1	CD44 > 1 & MKI67 < 1 NON LEUK	CDKN1A
MIR155HG	0.000544558	0.131882139	0.114	0.026	1	CD44 > 1 & MKI67 < 1 NON LEUK	MIR155HG
B4GALT1	0.000552131	0.101260616	0.212	0.084	1	CD44 > 1 & MKI67 < 1 NON LEUK	B4GALT1
PTGER2	0.000559583	0.134494008	0.318	0.157	1	CD44 > 1 & MKI67 < 1 NON LEUK	PTGER2
IL10RA	0.00056803	0.104632918	0.38	0.199	1	CD44 > 1 & MKI67 < 1 NON LEUK	IL10RA
B3GNT2	0.000628977	0.14950742	0.227	0.099	1	CD44 > 1 & MKI67 < 1 NON LEUK	B3GNT2
NCOR2	0.00064159	0.151996166	0.216	0.094	1	CD44 > 1 & MKI67 < 1 NON LEUK	NCOR2
LACTB	0.000647539	0.140071633	0.22	0.094	1	CD44 > 1 & MKI67 < 1 NON LEUK	LACTB
ITGA5	0.000650261	0.124413747	0.165	0.058	1	CD44 > 1 & MKI67 < 1 NON LEUK	ITGA5
SOX4	0.000651941	0.22597389	0.271	0.131	1	CD44 > 1 & MKI67 < 1 NON LEUK	SOX4

ABCA1	0.000669573	0.161447528	0.259	0.12	1	CD44 > 1 & MKI67 < 1 NON LEUK	ABCA1
ATP6V1H	0.000673574	0.123033439	0.243	0.11	1	CD44 > 1 & MKI67 < 1 NON LEUK	ATP6V1H
BRK1	0.000675251	0.117818885	0.361	0.188	1	CD44 > 1 & MKI67 < 1 NON LEUK	BRK1
RTL8C	0.000699344	0.104894328	0.102	0.021	1	CD44 > 1 & MKI67 < 1 NON LEUK	RTL8C
BLVRB	7.05E-04	0.12863953	0.161	0.058	1.00E+00	CD44 > 1 & MKI67 < 1 NON LEUK	BLVRB
SDF2L1	7.07E-04	0.165216642	0.224	0.094	1.00E+00	CD44 > 1 & MKI67 < 1 NON LEUK	SDF2L1
ARHGAP31	7.24E-04	0.107168751	0.114	0.026	1.00E+00	CD44 > 1 & MKI67 < 1 NON LEUK	ARHGAP31
TAGAP	7.35E-04	0.160652519	0.278	0.136	1.00E+00	CD44 > 1 & MKI67 < 1 NON LEUK	TAGAP
MAPKAPK2	7.49E-04	0.104897596	0.329	0.168	1.00E+00	CD44 > 1 & MKI67 < 1 NON LEUK	MAPKAPK2
MID1IP1	7.84E-04	0.135133767	0.129	0.037	1.00E+00	CD44 > 1 & MKI67 < 1 NON LEUK	MID1IP1
HCST	8.16E-04	0.188031323	0.608	0.393	1.00E+00	CD44 > 1 & MKI67 < 1 NON LEUK	HCST
HMGA2	8.41E-04	0.117076088	0.11	0.026	1.00E+00	CD44 > 1 & MKI67 < 1 NON LEUK	HMGA2
SRC	8.46E-04	0.116367796	0.137	0.042	1.00E+00	CD44 > 1 & MKI67 < 1 NON LEUK	SRC
NANS	8.59E-04	0.119952599	0.204	0.084	1.00E+00	CD44 > 1 & MKI67 < 1 NON LEUK	NANS
IFI6	8.91E-04	0.143278121	0.192	0.079	1.00E+00	CD44 > 1 & MKI67 < 1 NON LEUK	IFI6
KDM6B	8.96E-04	0.140314596	0.298	0.152	1.00E+00	CD44 > 1 & MKI67 < 1 NON LEUK	KDM6B
ANAPC15	9.13E-04	0.103527286	0.196	0.079	1.00E+00	CD44 > 1 & MKI67 < 1 NON LEUK	ANAPC15
CLEC4E	9.18E-04	0.153254676	0.149	0.052	1.00E+00	CD44 > 1 & MKI67 < 1 NON LEUK	CLEC4E
AP2S1	9.27E-04	0.123357496	0.29	0.147	1.00E+00	CD44 > 1 & MKI67 < 1 NON LEUK	AP2S1
LPXN	9.92E-04	0.107325842	0.145	0.047	1.00E+00	CD44 > 1 & MKI67 < 1 NON LEUK	LPXN
SPSB1	1.02E-03	0.141485765	0.118	0.031	1.00E+00	CD44 > 1 & MKI67 < 1 NON LEUK	SPSB1
ERO1A	1.07E-03	0.142653871	0.216	0.094	1.00E+00	CD44 > 1 & MKI67 < 1 NON LEUK	ERO1A
IRF2BP2	1.07E-03	0.157804862	0.329	0.178	1.00E+00	CD44 > 1 & MKI67 < 1 NON LEUK	IRF2BP2
RIPK2	1.08E-03	0.11552	0.2	0.084	1.00E+00	CD44 > 1 & MKI67 < 1 NON LEUK	RIPK2
GPR84	1.13E-03	0.135575787	0.133	0.042	1.00E+00	CD44 > 1 & MKI67 < 1 NON LEUK	GPR84
LGALS9	1.17E-03	0.110121647	0.224	0.099	1.00E+00	CD44 > 1 & MKI67 < 1 NON LEUK	LGALS9
C11orf96	1.18E-03	0.185039004	0.114	0.031	1.00E+00	CD44 > 1 & MKI67 < 1 NON LEUK	C11orf96
GBP1	1.20E-03	0.142981205	0.106	0.026	1.00E+00	CD44 > 1 & MKI67 < 1 NON LEUK	GBP1
STK40	1.22E-03	0.122878136	0.188	0.079	1.00E+00	CD44 > 1 & MKI67 < 1 NON LEUK	STK40
BAALC	1.22E-03	0.133546481	0.114	0.031	1.00E+00	CD44 > 1 & MKI67 < 1 NON LEUK	BAALC
ADGRE2	1.24E-03	0.123940867	0.106	0.026	1.00E+00	CD44 > 1 & MKI67 < 1 NON LEUK	ADGRE2
CAMKK2	1.26E-03	0.107596371	0.106	0.026	1.00E+00	CD44 > 1 & MKI67 < 1 NON LEUK	CAMKK2
BTG1	1.28E-03	0.243454906	0.973	0.921	1.00E+00	CD44 > 1 & MKI67 < 1 NON LEUK	BTG1
ETV6	1.29E-03	0.139843683	0.231	0.11	1.00E+00	CD44 > 1 & MKI67 < 1 NON LEUK	ETV6
REL	1.30E-03	0.185472006	0.49	0.298	1.00E+00	CD44 > 1 & MKI67 < 1 NON LEUK	REL
CDC37	1.31E-03	0.17419745	0.459	0.272	1.00E+00	CD44 > 1 & MKI67 < 1 NON LEUK	CDC37
PTGER4	1.34E-03	0.132054446	0.294	0.152	1.00E+00	CD44 > 1 & MKI67 < 1 NON LEUK	PTGER4
TFDP1	1.34E-03	0.136406559	0.165	0.063	1.00E+00	CD44 > 1 & MKI67 < 1 NON LEUK	TFDP1
IL4I1	1.36E-03	0.103674801	0.106	0.026	1.00E+00	CD44 > 1 & MKI67 < 1 NON LEUK	IL4I1
SPAG7	1.42E-03	0.120057559	0.165	0.063	1.00E+00	CD44 > 1 & MKI67 < 1 NON LEUK	SPAG7
FOSL1	1.46E-03	0.126024567	0.137	0.047	1.00E+00	CD44 > 1 & MKI67 < 1 NON LEUK	FOSL1
ZC3H12A	1.51E-03	0.131538452	0.224	0.105	1.00E+00	CD44 > 1 & MKI67 < 1 NON LEUK	ZC3H12A
ANTXR2	1.59E-03	0.10597607	0.129	0.042	1.00E+00	CD44 > 1 & MKI67 < 1 NON LEUK	ANTXR2
TWISTNB	1.69E-03	0.241913866	0.259	0.136	1.00E+00	CD44 > 1 & MKI67 < 1 NON LEUK	TWISTNB
RAC1	1.71E-03	0.193559423	0.624	0.424	1.00E+00	CD44 > 1 & MKI67 < 1 NON LEUK	RAC1
MAP1LC3A	1.73E-03	0.104771885	0.114	0.031	1.00E+00	CD44 > 1 & MKI67 < 1 NON LEUK	MAP1LC3A
ZCCHC2	1.74E-03	0.166837438	0.243	0.12	1.00E+00	CD44 > 1 & MKI67 < 1 NON LEUK	ZCCHC2
RB1CC1	1.77E-03	0.131712393	0.376	0.22	1.00E+00	CD44 > 1 & MKI67 < 1 NON LEUK	RB1CC1
TAGLN2	1.83E-03	0.19096667	0.663	0.445	1.00E+00	CD44 > 1 & MKI67 < 1 NON LEUK	TAGLN2
TLNRD1	1.85E-03	0.103042726	0.208	0.094	1.00E+00	CD44 > 1 & MKI67 < 1 NON LEUK	TLNRD1
ZBTB43	2.09E-03	0.152977893	0.239	0.12	1.00E+00	CD44 > 1 & MKI67 < 1 NON LEUK	ZBTB43
SLC39A8	2.11E-03	0.135573343	0.161	0.063	1.00E+00	CD44 > 1 & MKI67 < 1 NON LEUK	SLC39A8
TMEM219	2.16E-03	0.125680354	0.353	0.199	1.00E+00	CD44 > 1 & MKI67 < 1 NON LEUK	TMEM219
FMNL1	2.17E-03	0.113170028	0.22	0.105	1.00E+00	CD44 > 1 & MKI67 < 1 NON LEUK	FMNL1

DBI	2.27E-03	0.178699915	0.341	0.194	1.00E+00	CD44 > 1 & MKI67 < 1 NON LEUK	DBI
RBM23	2.34E-03	0.124149229	0.188	0.084	1.00E+00	CD44 > 1 & MKI67 < 1 NON LEUK	RBM23
MRPS24	2.35E-03	0.125921161	0.22	0.105	1.00E+00	CD44 > 1 & MKI67 < 1 NON LEUK	MRPS24
GLS	2.37E-03	0.11929939	0.267	0.136	1.00E+00	CD44 > 1 & MKI67 < 1 NON LEUK	GLS
CANX	2.44E-03	0.142740653	0.235	0.12	1.00E+00	CD44 > 1 & MKI67 < 1 NON LEUK	CANX
SQLE	2.44E-03	0.107215562	0.125	0.042	1.00E+00	CD44 > 1 & MKI67 < 1 NON LEUK	SQLE
PTTG1IP	2.53E-03	0.103367984	0.176	0.073	1.00E+00	CD44 > 1 & MKI67 < 1 NON LEUK	PTTG1IP
LRRC8C	2.72E-03	0.116269927	0.133	0.047	1.00E+00	CD44 > 1 & MKI67 < 1 NON LEUK	LRRC8C
ERCC1	2.77E-03	0.10307944	0.149	0.058	1.00E+00	CD44 > 1 & MKI67 < 1 NON LEUK	ERCC1
GLRX	2.79E-03	0.129383033	0.153	0.063	1.00E+00	CD44 > 1 & MKI67 < 1 NON LEUK	GLRX
ZNF655	2.86E-03	0.109261422	0.149	0.058	1.00E+00	CD44 > 1 & MKI67 < 1 NON LEUK	ZNF655
SLC12A7	2.99E-03	0.107602772	0.157	0.063	1.00E+00	CD44 > 1 & MKI67 < 1 NON LEUK	SLC12A7
YBX1	2.99E-03	0.165223267	0.855	0.702	1.00E+00	CD44 > 1 & MKI67 < 1 NON LEUK	YBX1
RFX2	3.03E-03	0.117047551	0.114	0.037	1.00E+00	CD44 > 1 & MKI67 < 1 NON LEUK	RFX2
IRAK3	3.10E-03	0.115601123	0.137	0.052	1.00E+00	CD44 > 1 & MKI67 < 1 NON LEUK	IRAK3
SRA1	3.10E-03	0.106810349	0.341	0.194	1.00E+00	CD44 > 1 & MKI67 < 1 NON LEUK	SRA1
MSN	3.16E-03	0.129423612	0.439	0.277	1.00E+00	CD44 > 1 & MKI67 < 1 NON LEUK	MSN
CD55	3.34E-03	0.10176185	0.365	0.209	1.00E+00	CD44 > 1 & MKI67 < 1 NON LEUK	CD55
PLIN2	3.50E-03	0.169327236	0.282	0.157	1.00E+00	CD44 > 1 & MKI67 < 1 NON LEUK	PLIN2
PSMG3	3.52E-03	0.104193799	0.106	0.031	1.00E+00	CD44 > 1 & MKI67 < 1 NON LEUK	PSMG3
LGALS8	3.58E-03	0.127920608	0.153	0.063	1.00E+00	CD44 > 1 & MKI67 < 1 NON LEUK	LGALS8
DUSP4	3.67E-03	0.185832048	0.392	0.246	1.00E+00	CD44 > 1 & MKI67 < 1 NON LEUK	DUSP4
MAFF	3.68E-03	0.139879524	0.212	0.105	1.00E+00	CD44 > 1 & MKI67 < 1 NON LEUK	MAFF
H1F0	3.69E-03	0.165535427	0.192	0.094	1.00E+00	CD44 > 1 & MKI67 < 1 NON LEUK	H1F0
GSTP1	3.76E-03	0.182673618	0.537	0.346	1.00E+00	CD44 > 1 & MKI67 < 1 NON LEUK	GSTP1
ERP29	3.83E-03	0.137902007	0.514	0.335	1.00E+00	CD44 > 1 & MKI67 < 1 NON LEUK	ERP29
BTG3	3.96E-03	0.100019104	0.184	0.084	1.00E+00	CD44 > 1 & MKI67 < 1 NON LEUK	BTG3
TNFSF14	4.38E-03	0.111910503	0.118	0.042	1.00E+00	CD44 > 1 & MKI67 < 1 NON LEUK	TNFSF14
ITPRID2	4.58E-03	0.105556942	0.208	0.105	1.00E+00	CD44 > 1 & MKI67 < 1 NON LEUK	ITPRID2
RELT	4.64E-03	0.109508424	0.125	0.047	1.00E+00	CD44 > 1 & MKI67 < 1 NON LEUK	RELT
VTI1B	4.73E-03	0.102352932	0.102	0.031	1.00E+00	CD44 > 1 & MKI67 < 1 NON LEUK	VTI1B
ARF5	4.78E-03	0.132183799	0.416	0.262	1.00E+00	CD44 > 1 & MKI67 < 1 NON LEUK	ARF5
SSR3	4.99E-03	0.110561191	0.231	0.12	1.00E+00	CD44 > 1 & MKI67 < 1 NON LEUK	SSR3
LRRFIP1	5.12E-03	0.150584071	0.604	0.408	1.00E+00	CD44 > 1 & MKI67 < 1 NON LEUK	LRRFIP1
RSAD2	5.17E-03	0.165215915	0.153	0.068	1.00E+00	CD44 > 1 & MKI67 < 1 NON LEUK	RSAD2
RBM3	5.46E-03	0.149767127	0.867	0.702	1.00E+00	CD44 > 1 & MKI67 < 1 NON LEUK	RBM3
KDM5B	5.58E-03	0.106263921	0.227	0.12	1.00E+00	CD44 > 1 & MKI67 < 1 NON LEUK	KDM5B
STK10	5.71E-03	0.108560289	0.173	0.079	1.00E+00	CD44 > 1 & MKI67 < 1 NON LEUK	STK10
NFATC1	5.76E-03	0.113404581	0.157	0.068	1.00E+00	CD44 > 1 & MKI67 < 1 NON LEUK	NFATC1
LDHA	5.93E-03	0.241645719	0.812	0.691	1.00E+00	CD44 > 1 & MKI67 < 1 NON LEUK	LDHA
ERVK3-1	6.50E-03	0.10363045	0.161	0.073	1.00E+00	CD44 > 1 & MKI67 < 1 NON LEUK	ERVK3-1
NBDY	6.73E-03	0.110638818	0.204	0.105	1.00E+00	CD44 > 1 & MKI67 < 1 NON LEUK	NBDY
CHIC2	7.16E-03	0.114375724	0.259	0.141	1.00E+00	CD44 > 1 & MKI67 < 1 NON LEUK	CHIC2
ARHGDIA	7.25E-03	0.16405371	0.58	0.408	1.00E+00	CD44 > 1 & MKI67 < 1 NON LEUK	ARHGDIA
TNFSF8	7.26E-03	0.157756552	0.235	0.126	1.00E+00	CD44 > 1 & MKI67 < 1 NON LEUK	TNFSF8
MCUB	7.35E-03	0.119557763	0.306	0.183	1.00E+00	CD44 > 1 & MKI67 < 1 NON LEUK	MCUB
NR3C1	8.52E-03	0.154749986	0.431	0.288	1.00E+00	CD44 > 1 & MKI67 < 1 NON LEUK	NR3C1
