# Supplementary material for: High CD44 expression and enhanced E-selectin binding identified as biomarkers of chemoresistant leukemic cells in human T-ALL
Source: Leukemia. 2024 Nov 24;39(2):323–36. doi: 10.1038/s41375-024-02473-7 (PMC11794132; doi:10.1038/s41375-024-02473-7)
Supplement: Supplementary file 13 — Supplemental Table 12 [file 41375_2024_2473_MOESM13_ESM.pdf]

upregulated genes in Ki67neg CD44high normal cells from Library 2 (Supplementary Figure 12b)

	p_val	avg_log2FC	pct.1	pct.2	p_val_adj	cluster	gene
CD44	1.41E-46	1.503811635	1	0.254	5.15E-42	CD44 > 1 & MKI67 < 1 NON Leuk	CD44
TOMM7	0.000392994	0.40435845	0.855	0.762	1	CD44 > 1 & MKI67 < 1 NON Leuk	TOMM7
RPS27A	0.00057243	0.256416785	1	0.997	1	CD44 > 1 & MKI67 < 1 NON Leuk	RPS27A
DYRK2	0.000710763	0.251911786	0.129	0.031	1	CD44 > 1 & MKI67 < 1 NON Leuk	DYRK2
ISG20	0.001581412	0.518623989	0.5	0.351	1	CD44 > 1 & MKI67 < 1 NON Leuk	ISG20
RPL38	0.001984643	0.292468212	0.855	0.846	1	CD44 > 1 & MKI67 < 1 NON Leuk	RPL38
UBA52	0.002320885	0.273899707	0.952	0.94	1	CD44 > 1 & MKI67 < 1 NON Leuk	UBA52
FAU	0.002397216	0.246760364	1	0.987	1	CD44 > 1 & MKI67 < 1 NON Leuk	FAU
RPS15A	0.002772374	0.187109539	1	0.997	1	CD44 > 1 & MKI67 < 1 NON Leuk	RPS15A
SLC35D2	0.005668012	0.236697262	0.145	0.053	1	CD44 > 1 & MKI67 < 1 NON Leuk	SLC35D2
KLF2	0.005940039	0.452965631	0.839	0.68	1	CD44 > 1 & MKI67 < 1 NON Leuk	KLF2
NDUFV2-AS1	0.00645467	0.201755983	0.113	0.034	1	CD44 > 1 & MKI67 < 1 NON Leuk	NDUFV2-AS1
SNTB2	0.006624127	0.230505954	0.113	0.034	1	CD44 > 1 & MKI67 < 1 NON Leuk	SNTB2
RPS26	0.007143637	0.215322096	1	1	1	CD44 > 1 & MKI67 < 1 NON Leuk	RPS26
GAS5	0.00744179	0.305278476	0.742	0.649	1	CD44 > 1 & MKI67 < 1 NON Leuk	GAS5
ETS1	0.007639869	0.488305316	0.452	0.335	1	CD44 > 1 & MKI67 < 1 NON Leuk	ETS1
RPL11	0.007891504	0.213297945	1	0.991	1	CD44 > 1 & MKI67 < 1 NON Leuk	RPL11
THEMIS	0.008869495	0.260829889	0.161	0.066	1	CD44 > 1 & MKI67 < 1 NON Leuk	THEMIS
RPS25	0.009086401	0.22368601	1	0.987	1	CD44 > 1 & MKI67 < 1 NON Leuk	RPS25
TNFAIP3	0.009259259	0.343943111	0.629	0.451	1	CD44 > 1 & MKI67 < 1 NON Leuk	TNFAIP3
TXNIP	0.009791077	0.478185759	0.887	0.824	1	CD44 > 1 & MKI67 < 1 NON Leuk	TXNIP
DNAJB6	1.47E-05	0.48884899	0.342	0.065	0.53676997	NON Leuk	DNAJB6
EIF3I	4.74E-05	0.442835433	0.301	0.048	1	NON Leuk	EIF3I
NDUFA1	0.000265531	0.329712104	0.392	0.129	1	NON Leuk	NDUFA1
SOX4	0.000512301	0.488283626	0.276	0.065	1	NON Leuk	SOX4
PSMA1	0.000749818	0.223312145	0.329	0.097	1	NON Leuk	PSMA1
LGALS9	0.000884208	0.274271101	0.21	0.032	1	NON Leuk	LGALS9
H3F3A	0.000976624	0.487052628	0.765	0.565	1	NON Leuk	H3F3A
NME1	0.001005256	0.214185526	0.182	0.016	1	NON Leuk	NME1
JPT1	0.001017737	0.269457862	0.21	0.032	1	NON Leuk	JPT1
HM13	0.001046338	0.230783411	0.213	0.032	1	NON Leuk	HM13
NEAT1	0.001113692	0.395310894	0.285	0.081	1	NON Leuk	NEAT1
TALDO1	0.001139404	0.34091929	0.279	0.081	1	NON Leuk	TALDO1
XRCC5	0.001165353	0.273935516	0.326	0.097	1	NON Leuk	XRCC5
LUC7L3	0.001328968	0.242971949	0.226	0.048	1	NON Leuk	LUC7L3
MYCBP2	0.001438429	0.293066315	0.201	0.032	1	NON Leuk	MYCBP2
STMN1	0.001461947	0.399173968	0.273	0.081	1	NON Leuk	STMN1
ACBD6	0.001467785	0.22476961	0.172	0.016	1	NON Leuk	ACBD6
HIST1H2AC	0.001499999	0.26978479	0.144	0	1	NON Leuk	HIST1H2AC
CPNE3	0.001500002	0.193294189	0.144	0	1	NON Leuk	CPNE3
ZNHIT1	0.001612735	0.260298391	0.273	0.081	1	NON Leuk	ZNHIT1
SYNGR2	0.001632454	0.337914357	0.254	0.065	1	NON Leuk	SYNGR2
MCM7	0.001714558	0.198336928	0.141	0	1	NON Leuk	MCM7
NKTR	0.001765865	0.300738774	0.245	0.065	1	NON Leuk	NKTR
RNF130	0.001980992	0.310278409	0.27	0.081	1	NON Leuk	RNF130
PARP1	0.002111605	0.16550726	0.201	0.032	1	NON Leuk	PARP1
ZMAT2	0.002207214	0.222208018	0.194	0.032	1	NON Leuk	ZMAT2
PTPN11	0.00223703	0.16788781	0.135	0	1	NON Leuk	PTPN11
TIMM13	0.002276429	0.20850005	0.219	0.048	1	NON Leuk	TIMM13

MRPL28	0.002371552	0.163577003	0.166	0.016	1	NON Leuk	MRPL28
EIF4EBP1	0.00255353	0.146708916	0.132	0	1	NON Leuk	EIF4EBP1
TFDP1	0.00255353	0.134779197	0.132	0	1	NON Leuk	TFDP1
OAZ1	0.0026823	0.387992888	0.643	0.355	1	NON Leuk	OAZ1
SMC1A	0.002777697	0.154358226	0.163	0.016	1	NON Leuk	SMC1A
SRSF3	0.002830154	0.318971428	0.42	0.194	1	NON Leuk	SRSF3
SLC7A5	0.002913552	0.223414157	0.129	0	1	NON Leuk	SLC7A5
SPTLC1	0.002913552	0.155245841	0.129	0	1	NON Leuk	SPTLC1
PSENEN	0.003032785	0.206400047	0.157	0.016	1	NON Leuk	PSENEN
SNX6	0.003062205	0.283888826	0.204	0.048	1	NON Leuk	SNX6
STAT1	0.003117815	0.212026179	0.157	0.016	1	NON Leuk	STAT1
SUGT1	0.003227153	0.168098391	0.157	0.016	1	NON Leuk	SUGT1
RETREG2	0.003322941	0.152240105	0.125	0	1	NON Leuk	RETREG2
HLA-DRA	0.003327516	0.534445712	0.241	0.065	1	NON Leuk	HLA-DRA
TMEM183A	0.003381685	0.179422202	0.154	0.016	1	NON Leuk	TMEM183A
RASSF1	0.003404379	0.184356849	0.238	0.065	1	NON Leuk	RASSF1
SNX2	0.003500437	0.217238264	0.154	0.016	1	NON Leuk	SNX2
SMC4	0.00350398	0.174451981	0.157	0.016	1	NON Leuk	SMC4
PSMD1	0.003788316	0.148082152	0.122	0	1	NON Leuk	PSMD1
SH3BGRL	0.00381348	0.194086398	0.282	0.097	1	NON Leuk	SH3BGRL
DDX42	0.003848134	0.17164754	0.15	0.016	1	NON Leuk	DDX42
UBE2S	0.004126317	0.24539905	0.26	0.081	1	NON Leuk	UBE2S
TUBA1C	0.004153097	0.110575118	0.154	0.016	1	NON Leuk	TUBA1C
NUDT1	0.004236199	0.153985397	0.15	0.016	1	NON Leuk	NUDT1
CENPK	0.004317171	0.154343993	0.119	0	1	NON Leuk	CENPK
METTL17	0.004317171	0.147648464	0.119	0	1	NON Leuk	METTL17
BLVRA	0.004317171	0.144275764	0.119	0	1	NON Leuk	BLVRA
CDCA7L	0.004317171	0.129234207	0.119	0	1	NON Leuk	CDCA7L
HSPH1	0.004443247	0.199391067	0.15	0.016	1	NON Leuk	HSPH1
TFDP2	0.004691066	0.147998016	0.15	0.016	1	NON Leuk	TFDP2
LAPTM4A	0.004697098	0.206483572	0.229	0.065	1	NON Leuk	LAPTM4A
PPP1R14B	0.004719409	0.277685723	0.276	0.097	1	NON Leuk	PPP1R14B
HSPB1	0.004731635	0.298164214	0.307	0.129	1	NON Leuk	HSPB1
ATP5F1C	0.004802969	0.252589643	0.292	0.113	1	NON Leuk	ATP5F1C
GBP4	0.004917994	0.207603586	0.116	0	1	NON Leuk	GBP4
JPX	0.004917994	0.15271096	0.116	0	1	NON Leuk	JPX
OSGEP	0.004917994	0.146099178	0.116	0	1	NON Leuk	OSGEP
HLA-DQB1	0.00501261	0.478510867	0.245	0.081	1	NON Leuk	HLA-DQB1
TNRC6B	0.005067873	0.221115755	0.197	0.048	1	NON Leuk	TNRC6B
CFLAR	0.00525097	0.281789378	0.197	0.048	1	NON Leuk	CFLAR
HSP90AA1	0.005253018	0.29555756	0.708	0.532	1	NON Leuk	HSP90AA1
BZW2	0.005332415	0.119699395	0.147	0.016	1	NON Leuk	BZW2
NDUFB8	0.005343441	0.225624532	0.364	0.161	1	NON Leuk	NDUFB8
CDK6	0.005399389	0.227520717	0.229	0.065	1	NON Leuk	CDK6
UROS	0.005512163	0.156844067	0.144	0.016	1	NON Leuk	UROS
MLF2	0.005522575	0.177713366	0.223	0.065	1	NON Leuk	MLF2
SUMF2	0.005600398	0.150903723	0.113	0	1	NON Leuk	SUMF2
NFE2	0.005600398	0.129676158	0.113	0	1	NON Leuk	NFE2
MPST	0.005663612	0.140670211	0.144	0.016	1	NON Leuk	MPST
SHOC2	0.005779644	0.193663039	0.144	0.016	1	NON Leuk	SHOC2
MYDGF	0.005864974	0.171750038	0.229	0.065	1	NON Leuk	MYDGF
SRSF9	0.006132079	0.242074113	0.376	0.177	1	NON Leuk	SRSF9

U2AF2	0.006375274	0.150403385	0.11	0	1	NON Leuk	U2AF2
SH3KBP1	0.006429044	0.228862807	0.194	0.048	1	NON Leuk	SH3KBP1
HNRNPH1	0.006444213	0.18748092	0.226	0.065	1	NON Leuk	HNRNPH1
BSG	0.00660611	0.350177809	0.332	0.161	1	NON Leuk	BSG
ATP5MD	0.006627029	0.214316689	0.295	0.113	1	NON Leuk	ATP5MD
NDUFB3	0.006691522	0.129970535	0.138	0.016	1	NON Leuk	NDUFB3
FOXP1	0.006756448	0.201330524	0.401	0.177	1	NON Leuk	FOXP1
DOCK8	0.006802647	0.238802606	0.166	0.032	1	NON Leuk	DOCK8
ABI1	0.006835126	0.180076705	0.141	0.016	1	NON Leuk	ABI1
TM7SF3	0.006835126	0.115116532	0.141	0.016	1	NON Leuk	TM7SF3
NUDT21	0.006974659	0.160978241	0.169	0.032	1	NON Leuk	NUDT21
MRPS34	0.007001023	0.176774516	0.223	0.065	1	NON Leuk	MRPS34
IFITM3	0.007016108	0.280483324	0.138	0.016	1	NON Leuk	IFITM3
HAGH	0.007016118	0.15832352	0.138	0.016	1	NON Leuk	HAGH
MAP1LC3B	0.007099316	0.26569754	0.254	0.097	1	NON Leuk	MAP1LC3B
RNF10	0.007147324	0.196389379	0.135	0.016	1	NON Leuk	RNF10
HNRNPUL1	0.007152769	0.206264375	0.26	0.097	1	NON Leuk	HNRNPUL1
PECAM1	0.007196109	0.183875104	0.135	0.016	1	NON Leuk	PECAM1
SS18L2	0.007231655	0.191526487	0.163	0.032	1	NON Leuk	SS18L2
ATP6V1F	0.00723195	0.104808274	0.339	0.145	1	NON Leuk	ATP6V1F
CARD8	0.007254963	0.16921239	0.107	0	1	NON Leuk	CARD8
MOB2	0.007254963	0.145391281	0.107	0	1	NON Leuk	MOB2
MRPL2	0.007254963	0.142865716	0.107	0	1	NON Leuk	MRPL2
AKR1C3	0.007254963	0.142271678	0.107	0	1	NON Leuk	AKR1C3
RIOK1	0.007254963	0.1343712	0.107	0	1	NON Leuk	RIOK1
IARS	0.007254963	0.115827465	0.107	0	1	NON Leuk	IARS
AHSA1	0.007254963	0.108596457	0.107	0	1	NON Leuk	AHSA1
RAB11A	0.007312384	0.160677834	0.223	0.065	1	NON Leuk	RAB11A
ACAA2	0.007415437	0.105325714	0.169	0.032	1	NON Leuk	ACAA2
PARVG	0.007449268	0.195480902	0.16	0.032	1	NON Leuk	PARVG
COMMD3	0.007552444	0.101271762	0.169	0.032	1	NON Leuk	COMMD3
TXN	0.007739885	0.219856313	0.263	0.097	1	NON Leuk	TXN
TMOD3	0.007743535	0.164176914	0.166	0.032	1	NON Leuk	TMOD3
CAP1	0.007958151	0.218072668	0.292	0.113	1	NON Leuk	CAP1
BCAP31	0.008024181	0.260022723	0.342	0.161	1	NON Leuk	BCAP31
PSMD6	0.008033567	0.163472372	0.163	0.032	1	NON Leuk	PSMD6
PDCD2	0.008082736	0.160181524	0.191	0.048	1	NON Leuk	PDCD2
GSTP1	0.008166281	0.441880951	0.48	0.258	1	NON Leuk	GSTP1
IVNS1ABP	0.008253452	0.165945038	0.103	0	1	NON Leuk	IVNS1ABP
REPS1	0.008253452	0.16251617	0.103	0	1	NON Leuk	REPS1
VHL	0.008253452	0.140873624	0.103	0	1	NON Leuk	VHL
PIH1D1	0.008253452	0.106974271	0.103	0	1	NON Leuk	PIH1D1
MRPL51	0.008674189	0.172752202	0.207	0.065	1	NON Leuk	MRPL51
EIF1AX	0.008712572	0.108460997	0.194	0.048	1	NON Leuk	EIF1AX
DYNLL1	0.008713162	0.214109134	0.216	0.065	1	NON Leuk	DYNLL1
GTF3C6	0.008791415	0.144966449	0.188	0.048	1	NON Leuk	GTF3C6
CDK4	0.008797446	0.166912757	0.157	0.032	1	NON Leuk	CDK4
RPL35	0.008854265	0.204970035	0.972	0.984	1	NON Leuk	RPL35
SLU7	0.009030614	0.209907025	0.182	0.048	1	NON Leuk	SLU7
UXT	0.009064896	0.299244168	0.539	0.306	1	NON Leuk	UXT
TAPBP	0.009116316	0.238649647	0.348	0.161	1	NON Leuk	TAPBP
HNRNPF	0.009380473	0.182706499	0.26	0.097	1	NON Leuk	HNRNPF

CENPF	0.009386603	0.162258515	0.1	0	1	NON Leuk	CENPF
ATXN10	0.009386603	0.117384292	0.1	0	1	NON Leuk	ATXN10
MALSU1	0.009386603	0.116609035	0.1	0	1	NON Leuk	MALSU1
TAF6	0.009386603	0.101167365	0.1	0	1	NON Leuk	TAF6
VDAC2	0.009428974	0.142554072	0.31	0.129	1	NON Leuk	VDAC2
MICOS10	0.009585567	0.186796963	0.263	0.097	1	NON Leuk	MICOS10
ITSN2	0.009628512	0.144927134	0.188	0.048	1	NON Leuk	ITSN2
ATP5ME	0.00964176	0.195529152	0.257	0.097	1	NON Leuk	ATP5ME
SENP6	0.009807694	0.150229965	0.238	0.081	1	NON Leuk	SENP6
BLVRB	0.009923536	0.527645923	0.15	0.032	1	NON Leuk	BLVRB
