# Supplementary material for: High CD44 expression and enhanced E-selectin binding identified as biomarkers of chemoresistant leukemic cells in human T-ALL
Source: Leukemia. 2024 Nov 24;39(2):323–36. doi: 10.1038/s41375-024-02473-7 (PMC11794132; doi:10.1038/s41375-024-02473-7)
Supplement: Supplementary file 14 — Supplemental Table 13 [file 41375_2024_2473_MOESM14_ESM.pdf]

upregulated genes in Ki67neg CD44high normal cells from Library 3 (Supplementary Figure 12c)

	p_val	avg_log2FC	pct.1	pct.2	p_val_adj	cluster	gene
CD44	2.28E-52	1.395106026	1	0.234	8.35E-48	CD44 > 1 & MKI67 < 1 NON Leuk	CD44
KCTD2	4.73E-07	0.157612512	0.111	0.012	0.017297389	CD44 > 1 & MKI67 < 1 NON Leuk	KCTD2
MIR4435-2HG	5.54E-07	0.289441316	0.222	0.05	0.020283289	CD44 > 1 & MKI67 < 1 NON Leuk	MIR4435-2HG
S100A8	6.73E-07	1.109125085	0.241	0.059	0.024639029	CD44 > 1 & MKI67 < 1 NON Leuk	S100A8
CSF3R	8.94E-07	0.287423734	0.167	0.03	0.032703933	CD44 > 1 & MKI67 < 1 NON Leuk	CSF3R
RXRA	9.31E-07	0.209724278	0.167	0.03	0.034071245	CD44 > 1 & MKI67 < 1 NON Leuk	RXRA
HRH2	9.98E-07	0.227204653	0.185	0.036	0.036523412	CD44 > 1 & MKI67 < 1 NON Leuk	HRH2
VCAN	1.31E-06	0.47013236	0.185	0.038	0.048015105	CD44 > 1 & MKI67 < 1 NON Leuk	VCAN
RNF130	1.72E-06	0.290991588	0.296	0.086	0.06281974	CD44 > 1 & MKI67 < 1 NON Leuk	RNF130
CFD	2.54E-06	0.693671168	0.222	0.058	0.093110138	CD44 > 1 & MKI67 < 1 NON Leuk	CFD
FCN1	3.79E-06	1.095402423	0.241	0.068	0.138879395	CD44 > 1 & MKI67 < 1 NON Leuk	FCN1
S100A10	4.32E-06	0.795447144	0.667	0.419	0.158067352	CD44 > 1 & MKI67 < 1 NON Leuk	S100A10
CD36	4.49E-06	0.132777559	0.111	0.015	0.164424507	CD44 > 1 & MKI67 < 1 NON Leuk	CD36
TGFBI	4.73E-06	0.122830341	0.148	0.027	0.173158798	CD44 > 1 & MKI67 < 1 NON Leuk	TGFBI
LYZ	7.51E-06	1.097425258	0.241	0.068	0.274718751	CD44 > 1 & MKI67 < 1 NON Leuk	LYZ
NR4A1	7.65E-06	0.23688181	0.185	0.042	0.28009061	CD44 > 1 & MKI67 < 1 NON Leuk	NR4A1
C3AR1	9.26E-06	0.20442184	0.148	0.029	0.338951286	CD44 > 1 & MKI67 < 1 NON Leuk	C3AR1
S100A9	1.20E-05	1.453291692	0.241	0.071	0.437406178	CD44 > 1 & MKI67 < 1 NON Leuk	S100A9
CLEC12A	1.30E-05	0.344276337	0.185	0.046	0.474478733	CD44 > 1 & MKI67 < 1 NON Leuk	CLEC12A
LGALS2	1.31E-05	0.469878622	0.167	0.038	0.480623399	CD44 > 1 & MKI67 < 1 NON Leuk	LGALS2
SERPINA1	1.35E-05	0.646833474	0.241	0.07	0.495730816	CD44 > 1 & MKI67 < 1 NON Leuk	SERPINA1
CD14	1.78E-05	0.583876865	0.167	0.038	0.651311306	CD44 > 1 & MKI67 < 1 NON Leuk	CD14
CFP	2.15E-05	0.469532778	0.204	0.056	0.786399166	CD44 > 1 & MKI67 < 1 NON Leuk	CFP
MNDA	2.37E-05	0.588086807	0.222	0.065	0.866947695	CD44 > 1 & MKI67 < 1 NON Leuk	MNDA
TYROBP	2.48E-05	1.046114444	0.481	0.253	0.906916092	CD44 > 1 & MKI67 < 1 NON Leuk	TYROBP
SLC31A2	2.65E-05	0.2169967	0.148	0.032	0.968833321	CD44 > 1 & MKI67 < 1 NON Leuk	SLC31A2
JAML	3.05E-05	0.429923041	0.222	0.068	1	CD44 > 1 & MKI67 < 1 NON Leuk	JAML
ITGA5	3.27E-05	0.182841395	0.148	0.032	1	CD44 > 1 & MKI67 < 1 NON Leuk	ITGA5
TMEM176B	3.96E-05	0.496527716	0.222	0.065	1	CD44 > 1 & MKI67 < 1 NON Leuk	TMEM176B
GCLM	4.79E-05	0.149696837	0.13	0.026	1	CD44 > 1 & MKI67 < 1 NON Leuk	GCLM
TIMP1	4.87E-05	0.686847235	0.315	0.123	1	CD44 > 1 & MKI67 < 1 NON Leuk	TIMP1
CSF1R	5.60E-05	0.256606811	0.204	0.058	1	CD44 > 1 & MKI67 < 1 NON Leuk	CSF1R
AIF1	5.61E-05	0.787777206	0.315	0.124	1	CD44 > 1 & MKI67 < 1 NON Leuk	AIF1
CPVL	5.79E-05	0.250610317	0.204	0.058	1	CD44 > 1 & MKI67 < 1 NON Leuk	CPVL
SUSD6	6.13E-05	0.162866517	0.148	0.033	1	CD44 > 1 & MKI67 < 1 NON Leuk	SUSD6
ARRDC1	6.82E-05	0.340194415	0.333	0.138	1	CD44 > 1 & MKI67 < 1 NON Leuk	ARRDC1
GABARAP	0.000102313	0.476564681	0.815	0.666	1	CD44 > 1 & MKI67 < 1 NON Leuk	GABARAP
ADA2	0.000103741	0.35307822	0.296	0.117	1	CD44 > 1 & MKI67 < 1 NON Leuk	ADA2
AHNAK	0.000136769	0.520886803	0.5	0.294	1	CD44 > 1 & MKI67 < 1 NON Leuk	AHNAK
SRGN	0.000139048	0.518648706	0.667	0.401	1	CD44 > 1 & MKI67 < 1 NON Leuk	SRGN
NINJ1	0.000145073	0.356529242	0.259	0.096	1	CD44 > 1 & MKI67 < 1 NON Leuk	NINJ1
RTN3	0.000153509	0.285985072	0.333	0.147	1	CD44 > 1 & MKI67 < 1 NON Leuk	RTN3
LILRB1	0.000164243	0.279173047	0.204	0.065	1	CD44 > 1 & MKI67 < 1 NON Leuk	LILRB1
CD300LF	0.000197203	0.132748607	0.111	0.023	1	CD44 > 1 & MKI67 < 1 NON Leuk	CD300LF
BAHD1	0.000200901	0.177657579	0.111	0.023	1	CD44 > 1 & MKI67 < 1 NON Leuk	BAHD1
LRP1	0.00020596	0.21744675	0.13	0.03	1	CD44 > 1 & MKI67 < 1 NON Leuk	LRP1
CD63	0.000207469	0.471875108	0.5	0.285	1	CD44 > 1 & MKI67 < 1 NON Leuk	CD63
MS4A6A	0.000213542	0.28811949	0.167	0.047	1	CD44 > 1 & MKI67 < 1 NON Leuk	MS4A6A
CSTA	0.000236378	0.185485039	0.167	0.047	1	CD44 > 1 & MKI67 < 1 NON Leuk	CSTA
TNFSF10	0.000242288	0.425263853	0.333	0.153	1	CD44 > 1 & MKI67 < 1 NON Leuk	TNFSF10

NFIL3	0.000244502	0.132158461	0.13	0.03	1	CD44 > 1 & MKI67 < 1 NON Leuk	NFIL3
CTSH	0.000249898	0.438972289	0.333	0.152	1	CD44 > 1 & MKI67 < 1 NON Leuk	CTSH
NBR1	0.000272571	0.248463725	0.222	0.077	1	CD44 > 1 & MKI67 < 1 NON Leuk	NBR1
CAMK2G	0.000287324	0.179757403	0.148	0.039	1	CD44 > 1 & MKI67 < 1 NON Leuk	CAMK2G
FCER1G	0.000345745	0.697960108	0.426	0.226	1	CD44 > 1 & MKI67 < 1 NON Leuk	FCER1G
TMEM176A	0.00037685	0.189735726	0.185	0.056	1	CD44 > 1 & MKI67 < 1 NON Leuk	TMEM176A
KIF3B	0.000390809	0.133428996	0.13	0.032	1	CD44 > 1 & MKI67 < 1 NON Leuk	KIF3B
ADGRE5	0.000456999	0.424006287	0.426	0.229	1	CD44 > 1 & MKI67 < 1 NON Leuk	ADGRE5
SMIM25	0.000482338	0.379922267	0.167	0.05	1	CD44 > 1 & MKI67 < 1 NON Leuk	SMIM25
GBP1	0.000484405	0.35853576	0.259	0.106	1	CD44 > 1 & MKI67 < 1 NON Leuk	GBP1
COX8A	0.000486029	0.376148084	0.63	0.442	1	CD44 > 1 & MKI67 < 1 NON Leuk	COX8A
TNFSF13B	0.000502342	0.304561986	0.167	0.052	1	CD44 > 1 & MKI67 < 1 NON Leuk	TNFSF13B
TNFAIP3	0.000649607	0.338132917	0.204	0.074	1	CD44 > 1 & MKI67 < 1 NON Leuk	TNFAIP3
PLBD1	0.000656418	0.159688748	0.111	0.026	1	CD44 > 1 & MKI67 < 1 NON Leuk	PLBD1
LGALS3	0.000657398	0.374407604	0.278	0.12	1	CD44 > 1 & MKI67 < 1 NON Leuk	LGALS3
S100A11	0.000661704	0.76192092	0.519	0.349	1	CD44 > 1 & MKI67 < 1 NON Leuk	S100A11
NCOA6	0.000724636	0.189193921	0.148	0.044	1	CD44 > 1 & MKI67 < 1 NON Leuk	NCOA6
GPBAR1	0.00075279	0.251795458	0.13	0.035	1	CD44 > 1 & MKI67 < 1 NON Leuk	GPBAR1
GLIPR2	0.000766994	0.321731364	0.278	0.123	1	CD44 > 1 & MKI67 < 1 NON Leuk	GLIPR2
SH3BGRL3	0.000821156	0.380529869	0.926	0.888	1	CD44 > 1 & MKI67 < 1 NON Leuk	SH3BGRL3
NEAT1	0.000821328	0.58781629	0.481	0.293	1	CD44 > 1 & MKI67 < 1 NON Leuk	NEAT1
SLC7A7	0.000840921	0.18209936	0.148	0.044	1	CD44 > 1 & MKI67 < 1 NON Leuk	SLC7A7
BAG1	0.000940101	0.202267308	0.315	0.143	1	CD44 > 1 & MKI67 < 1 NON Leuk	BAG1
CST3	0.000954776	1.306664116	0.259	0.115	1	CD44 > 1 & MKI67 < 1 NON Leuk	CST3
S100A6	0.000958166	0.929611581	0.611	0.473	1	CD44 > 1 & MKI67 < 1 NON Leuk	S100A6
RNF213	0.001032237	0.438930927	0.537	0.351	1	CD44 > 1 & MKI67 < 1 NON Leuk	RNF213
CORO1C	0.001086655	0.167033823	0.148	0.046	1	CD44 > 1 & MKI67 < 1 NON Leuk	CORO1C
KPNA1	0.001092116	0.251197335	0.167	0.056	1	CD44 > 1 & MKI67 < 1 NON Leuk	KPNA1
PIGT	0.001129065	0.138421843	0.241	0.094	1	CD44 > 1 & MKI67 < 1 NON Leuk	PIGT
PSMB4	0.001164047	0.410984585	0.333	0.171	1	CD44 > 1 & MKI67 < 1 NON Leuk	PSMB4
PSAP	0.001183346	0.993033783	0.519	0.373	1	CD44 > 1 & MKI67 < 1 NON Leuk	PSAP
LILRB4	0.001253288	0.114922906	0.148	0.046	1	CD44 > 1 & MKI67 < 1 NON Leuk	LILRB4
NCF2	0.001288028	0.180874961	0.204	0.076	1	CD44 > 1 & MKI67 < 1 NON Leuk	NCF2
S100A4	0.001321576	0.695093598	0.667	0.513	1	CD44 > 1 & MKI67 < 1 NON Leuk	S100A4
DUSP6	0.001333285	0.252191387	0.204	0.076	1	CD44 > 1 & MKI67 < 1 NON Leuk	DUSP6
NPEPL1	0.001408617	0.167127334	0.111	0.029	1	CD44 > 1 & MKI67 < 1 NON Leuk	NPEPL1
TNFAIP2	0.001419716	0.396890342	0.13	0.038	1	CD44 > 1 & MKI67 < 1 NON Leuk	TNFAIP2
SH2B3	0.001472082	0.184538931	0.148	0.047	1	CD44 > 1 & MKI67 < 1 NON Leuk	SH2B3
PYCARD	0.001514888	0.366884811	0.481	0.297	1	CD44 > 1 & MKI67 < 1 NON Leuk	PYCARD
PLCB2	0.001530582	0.13770888	0.167	0.056	1	CD44 > 1 & MKI67 < 1 NON Leuk	PLCB2
KLF10	0.001540009	0.249893196	0.222	0.09	1	CD44 > 1 & MKI67 < 1 NON Leuk	KLF10
SEC11A	0.001588748	0.273329986	0.444	0.255	1	CD44 > 1 & MKI67 < 1 NON Leuk	SEC11A
MVP	0.001707538	0.232138445	0.278	0.127	1	CD44 > 1 & MKI67 < 1 NON Leuk	MVP
IGSF6	0.001714066	0.487453553	0.185	0.07	1	CD44 > 1 & MKI67 < 1 NON Leuk	IGSF6
FCGRT	0.001718559	0.461501036	0.278	0.134	1	CD44 > 1 & MKI67 < 1 NON Leuk	FCGRT
VSIR	0.001759119	0.42800964	0.389	0.228	1	CD44 > 1 & MKI67 < 1 NON Leuk	VSIR
CHCHD1	0.001825789	0.274067061	0.259	0.12	1	CD44 > 1 & MKI67 < 1 NON Leuk	CHCHD1
ELF1	0.0020043	0.291166244	0.537	0.317	1	CD44 > 1 & MKI67 < 1 NON Leuk	ELF1
LDHA	0.002031943	0.22747079	0.611	0.36	1	CD44 > 1 & MKI67 < 1 NON Leuk	LDHA
CEBPD	0.002049511	0.44418244	0.222	0.094	1	CD44 > 1 & MKI67 < 1 NON Leuk	CEBPD
CDKN1C	0.002086849	0.175100213	0.13	0.038	1	CD44 > 1 & MKI67 < 1 NON Leuk	CDKN1C
PPP1R15A	0.00209273	0.336165295	0.315	0.153	1	CD44 > 1 & MKI67 < 1 NON Leuk	PPP1R15A
CD4	0.002103864	0.162961287	0.148	0.049	1	CD44 > 1 & MKI67 < 1 NON Leuk	CD4

CD151	0.002152977	0.188010689	0.167	0.061	1	CD44 > 1 & MKI67 < 1 NON Leuk	CD151
CD93	0.002158611	0.132826387	0.111	0.03	1	CD44 > 1 & MKI67 < 1 NON Leuk	CD93
RAC1	0.002283652	0.439858647	0.574	0.422	1	CD44 > 1 & MKI67 < 1 NON Leuk	RAC1
CD300E	0.002312041	0.196821789	0.13	0.039	1	CD44 > 1 & MKI67 < 1 NON Leuk	CD300E
TCIRG1	0.002335046	0.416712341	0.352	0.196	1	CD44 > 1 & MKI67 < 1 NON Leuk	TCIRG1
FGL2	0.002464407	0.300854688	0.185	0.073	1	CD44 > 1 & MKI67 < 1 NON Leuk	FGL2
EMC10	0.002474364	0.238852075	0.241	0.108	1	CD44 > 1 & MKI67 < 1 NON Leuk	EMC10
HDAC8	0.002618106	0.225399787	0.167	0.062	1	CD44 > 1 & MKI67 < 1 NON Leuk	HDAC8
CLEC2B	0.002807483	0.318079291	0.463	0.272	1	CD44 > 1 & MKI67 < 1 NON Leuk	CLEC2B
HCST	0.003019417	0.384444064	0.667	0.464	1	CD44 > 1 & MKI67 < 1 NON Leuk	HCST
SMCO4	0.003048805	0.173515206	0.167	0.062	1	CD44 > 1 & MKI67 < 1 NON Leuk	SMCO4
SLC39A13	0.003285297	0.133049508	0.13	0.041	1	CD44 > 1 & MKI67 < 1 NON Leuk	SLC39A13
IFITM3	0.003379557	0.99397682	0.667	0.504	1	CD44 > 1 & MKI67 < 1 NON Leuk	IFITM3
NUDT16	0.003412865	0.159833801	0.148	0.052	1	CD44 > 1 & MKI67 < 1 NON Leuk	NUDT16
FBP1	0.003444466	0.12151835	0.111	0.032	1	CD44 > 1 & MKI67 < 1 NON Leuk	FBP1
TMED4	0.003521688	0.283368557	0.315	0.17	1	CD44 > 1 & MKI67 < 1 NON Leuk	TMED4
MS4A4A	0.003537603	0.144563825	0.111	0.032	1	CD44 > 1 & MKI67 < 1 NON Leuk	MS4A4A
GLE1	0.003537603	0.102186985	0.111	0.032	1	CD44 > 1 & MKI67 < 1 NON Leuk	GLE1
MYD88	0.003664454	0.210040437	0.278	0.134	1	CD44 > 1 & MKI67 < 1 NON Leuk	MYD88
CEBPB	0.003666639	0.391395449	0.278	0.144	1	CD44 > 1 & MKI67 < 1 NON Leuk	CEBPB
CASP1	0.003674555	0.326918299	0.278	0.141	1	CD44 > 1 & MKI67 < 1 NON Leuk	CASP1
LCP2	0.003805636	0.350335572	0.37	0.212	1	CD44 > 1 & MKI67 < 1 NON Leuk	LCP2
CLEC4A	0.003883705	0.163986809	0.148	0.052	1	CD44 > 1 & MKI67 < 1 NON Leuk	CLEC4A
MAP3K5	0.004020872	0.158310841	0.13	0.042	1	CD44 > 1 & MKI67 < 1 NON Leuk	MAP3K5
MS4A7	0.004160874	0.330127006	0.167	0.064	1	CD44 > 1 & MKI67 < 1 NON Leuk	MS4A7
SLAIN2	0.004368592	0.177026413	0.222	0.097	1	CD44 > 1 & MKI67 < 1 NON Leuk	SLAIN2
UBE2D1	0.004523882	0.193662872	0.222	0.1	1	CD44 > 1 & MKI67 < 1 NON Leuk	UBE2D1
COTL1	0.004638736	0.503716194	0.593	0.419	1	CD44 > 1 & MKI67 < 1 NON Leuk	COTL1
POM121C	0.004840223	0.217749826	0.13	0.044	1	CD44 > 1 & MKI67 < 1 NON Leuk	POM121C
BRI3	0.005011881	0.566490483	0.352	0.217	1	CD44 > 1 & MKI67 < 1 NON Leuk	BRI3
ITM2B	0.005039588	0.393468443	0.833	0.706	1	CD44 > 1 & MKI67 < 1 NON Leuk	ITM2B
ANXA1	0.005048067	0.409097135	0.444	0.266	1	CD44 > 1 & MKI67 < 1 NON Leuk	ANXA1
ITGB2	0.005056826	0.395836573	0.63	0.47	1	CD44 > 1 & MKI67 < 1 NON Leuk	ITGB2
MRPL38	0.005122718	0.256978055	0.278	0.146	1	CD44 > 1 & MKI67 < 1 NON Leuk	MRPL38
FTH1	0.0051539	0.904401285	1	0.941	1	CD44 > 1 & MKI67 < 1 NON Leuk	FTH1
LILRA2	0.005445238	0.128964461	0.13	0.044	1	CD44 > 1 & MKI67 < 1 NON Leuk	LILRA2
FBXL5	0.005479895	0.206616173	0.222	0.103	1	CD44 > 1 & MKI67 < 1 NON Leuk	FBXL5
CRYZL1	0.005480874	0.174525625	0.148	0.055	1	CD44 > 1 & MKI67 < 1 NON Leuk	CRYZL1
ZFP36L2	0.005584951	0.392484176	0.667	0.531	1	CD44 > 1 & MKI67 < 1 NON Leuk	ZFP36L2
ASAH1	0.005777757	0.246443987	0.315	0.173	1	CD44 > 1 & MKI67 < 1 NON Leuk	ASAH1
LINC00909	0.005801791	0.237659743	0.13	0.046	1	CD44 > 1 & MKI67 < 1 NON Leuk	LINC00909
CTSC	0.005832025	0.302441318	0.481	0.288	1	CD44 > 1 & MKI67 < 1 NON Leuk	CTSC
TBXAS1	0.005849566	0.213309958	0.185	0.077	1	CD44 > 1 & MKI67 < 1 NON Leuk	TBXAS1
SQSTM1	0.005884672	0.242685249	0.444	0.273	1	CD44 > 1 & MKI67 < 1 NON Leuk	SQSTM1
MAIP1	0.005922183	0.123914933	0.111	0.035	1	CD44 > 1 & MKI67 < 1 NON Leuk	MAIP1
GIMAP2	0.006086665	0.150741693	0.204	0.088	1	CD44 > 1 & MKI67 < 1 NON Leuk	GIMAP2
AATF	0.006196343	0.204366318	0.278	0.143	1	CD44 > 1 & MKI67 < 1 NON Leuk	AATF
RAB5IF	0.006223509	0.203650557	0.426	0.255	1	CD44 > 1 & MKI67 < 1 NON Leuk	RAB5IF
RAP1B	0.006332974	0.353916891	0.574	0.414	1	CD44 > 1 & MKI67 < 1 NON Leuk	RAP1B
TSC22D4	0.0064118	0.298660911	0.37	0.217	1	CD44 > 1 & MKI67 < 1 NON Leuk	TSC22D4
CLIC4	0.006442024	0.241295096	0.167	0.07	1	CD44 > 1 & MKI67 < 1 NON Leuk	CLIC4
LINC00861	0.006500076	0.284869862	0.296	0.159	1	CD44 > 1 & MKI67 < 1 NON Leuk	LINC00861
LST1	0.006515028	0.701868679	0.278	0.152	1	CD44 > 1 & MKI67 < 1 NON Leuk	LST1

ARHGEF1	0.006525193	0.251599174	0.5	0.334	1	CD44 > 1 & MKI67 < 1 NON Leuk	ARHGEF1
WDR33	0.006531341	0.271512379	0.278	0.147	1	CD44 > 1 & MKI67 < 1 NON Leuk	WDR33
MEGF9	0.006569333	0.10583051	0.111	0.035	1	CD44 > 1 & MKI67 < 1 NON Leuk	MEGF9
FYB1	0.006908387	0.454551329	0.5	0.338	1	CD44 > 1 & MKI67 < 1 NON Leuk	FYB1
PLEC	0.006999766	0.143857414	0.204	0.088	1	CD44 > 1 & MKI67 < 1 NON Leuk	PLEC
VAMP7	0.007086923	0.160758008	0.148	0.058	1	CD44 > 1 & MKI67 < 1 NON Leuk	VAMP7
YIPF3	0.007132108	0.29748965	0.315	0.182	1	CD44 > 1 & MKI67 < 1 NON Leuk	YIPF3
STIMATE	0.007231436	0.150364921	0.111	0.036	1	CD44 > 1 & MKI67 < 1 NON Leuk	STIMATE
LGALS1	0.007440564	0.658577909	0.407	0.264	1	CD44 > 1 & MKI67 < 1 NON Leuk	LGALS1
USP36	0.007458936	0.181166558	0.13	0.047	1	CD44 > 1 & MKI67 < 1 NON Leuk	USP36
RGL2	0.007747606	0.189490773	0.148	0.058	1	CD44 > 1 & MKI67 < 1 NON Leuk	RGL2
RARA	0.007755507	0.122613268	0.167	0.067	1	CD44 > 1 & MKI67 < 1 NON Leuk	RARA
DENND5A	0.007885182	0.139639141	0.148	0.056	1	CD44 > 1 & MKI67 < 1 NON Leuk	DENND5A
SDCBP	0.007894937	0.21361553	0.278	0.146	1	CD44 > 1 & MKI67 < 1 NON Leuk	SDCBP
ZYX	0.008022158	0.396581564	0.333	0.209	1	CD44 > 1 & MKI67 < 1 NON Leuk	ZYX
OS9	0.00806274	0.315626218	0.333	0.203	1	CD44 > 1 & MKI67 < 1 NON Leuk	OS9
N4BP1	0.008197036	0.181328429	0.204	0.093	1	CD44 > 1 & MKI67 < 1 NON Leuk	N4BP1
IFIT5	0.008271671	0.123266038	0.13	0.046	1	CD44 > 1 & MKI67 < 1 NON Leuk	IFIT5
PTPRE	0.008335558	0.203592552	0.222	0.103	1	CD44 > 1 & MKI67 < 1 NON Leuk	PTPRE
EMP3	0.008347456	0.330507567	0.778	0.659	1	CD44 > 1 & MKI67 < 1 NON Leuk	EMP3
CLEC7A	0.008403549	0.111611673	0.13	0.046	1	CD44 > 1 & MKI67 < 1 NON Leuk	CLEC7A
LILRB2	0.008516946	0.160462642	0.148	0.056	1	CD44 > 1 & MKI67 < 1 NON Leuk	LILRB2
CARD16	0.008589151	0.284090238	0.463	0.305	1	CD44 > 1 & MKI67 < 1 NON Leuk	CARD16
DENND6A	0.008721787	0.123378323	0.111	0.036	1	CD44 > 1 & MKI67 < 1 NON Leuk	DENND6A
LUZP1	0.008769867	0.125812816	0.13	0.047	1	CD44 > 1 & MKI67 < 1 NON Leuk	LUZP1
UBA1	0.008834774	0.211818916	0.278	0.147	1	CD44 > 1 & MKI67 < 1 NON Leuk	UBA1
TATDN1	0.008977209	0.231109123	0.148	0.061	1	CD44 > 1 & MKI67 < 1 NON Leuk	TATDN1
DRG2	0.00908468	0.197299802	0.185	0.083	1	CD44 > 1 & MKI67 < 1 NON Leuk	DRG2
ARAP1	0.009292673	0.225412575	0.167	0.07	1	CD44 > 1 & MKI67 < 1 NON Leuk	ARAP1
METTL5	0.009332267	0.297059341	0.259	0.143	1	CD44 > 1 & MKI67 < 1 NON Leuk	METTL5
IMMT	0.009338307	0.195274332	0.222	0.108	1	CD44 > 1 & MKI67 < 1 NON Leuk	IMMT
ATG3	0.009649739	0.283260395	0.315	0.176	1	CD44 > 1 & MKI67 < 1 NON Leuk	ATG3
PTMA	1.29E-05	0.54302419	0.982	0.963	0.472745331	NON Leuk	PTMA
CD79A	2.28E-05	1.018384295	0.534	0.259	0.834834964	NON Leuk	CD79A
TCL1A	3.04E-05	1.2482924	0.439	0.148	1	NON Leuk	TCL1A
NIBAN3	5.91E-05	0.689427817	0.343	0.074	1	NON Leuk	NIBAN3
SEPTIN7	9.90E-05	0.583631597	0.61	0.37	1	NON Leuk	SEPTIN7
MS4A1	0.000118271	0.865466624	0.451	0.167	1	NON Leuk	MS4A1
CYB561A3	0.000154988	0.564897561	0.319	0.074	1	NON Leuk	CYB561A3
CD79B	0.000185945	0.936547	0.545	0.296	1	NON Leuk	CD79B
AFF3	0.000321886	0.550726219	0.323	0.093	1	NON Leuk	AFF3
VPREB3	0.000397097	0.820180564	0.343	0.111	1	NON Leuk	VPREB3
EEF1A1	0.000526156	0.351974665	0.998	1	1	NON Leuk	EEF1A1
RPL10	0.000539082	0.335440757	0.995	1	1	NON Leuk	RPL10
SPIB	0.000578498	0.737662158	0.361	0.13	1	NON Leuk	SPIB
RPS5	0.000643511	0.425504805	0.959	0.926	1	NON Leuk	RPS5
IGHM	0.000684553	0.797199524	0.466	0.222	1	NON Leuk	IGHM
FCRLA	0.000706956	0.585807007	0.284	0.074	1	NON Leuk	FCRLA
RPL6	0.000877102	0.339532145	0.979	0.963	1	NON Leuk	RPL6
CD37	0.0009051	0.573543834	0.866	0.815	1	NON Leuk	CD37
RPS6	0.001207028	0.384975261	0.977	0.981	1	NON Leuk	RPS6
SNRPB	0.001400256	0.331400718	0.378	0.148	1	NON Leuk	SNRPB
NCL	0.001647025	0.43443416	0.646	0.444	1	NON Leuk	NCL

EEF1G	0.001774107	0.434047318	0.856	0.778	1	NON Leuk	EEF1G
CORO1A	0.001839222	0.305485219	0.862	0.741	1	NON Leuk	CORO1A
SLC25A5	0.001952783	0.443110064	0.581	0.407	1	NON Leuk	SLC25A5
RPSA	0.002017151	0.400635812	0.941	0.944	1	NON Leuk	RPSA
RPL5	0.002190501	0.325625551	0.954	0.981	1	NON Leuk	RPL5
PABPN1	0.002259853	0.375583581	0.466	0.241	1	NON Leuk	PABPN1
NCF1	0.002567326	0.556172695	0.496	0.296	1	NON Leuk	NCF1
CD24	0.003292596	0.418297041	0.208	0.037	1	NON Leuk	CD24
RPL23A	0.003845478	0.318051846	0.968	0.889	1	NON Leuk	RPL23A
IGLC2	0.004260458	0.685791858	0.193	0.037	1	NON Leuk	IGLC2
RPL27A	0.004559376	0.36192126	0.731	0.593	1	NON Leuk	RPL27A
RPL7A	0.004689725	0.292227424	0.992	0.981	1	NON Leuk	RPL7A
BACH2	0.004863774	0.377404801	0.226	0.056	1	NON Leuk	BACH2
TMUB1	0.004927244	0.225718195	0.191	0.037	1	NON Leuk	TMUB1
RPS18	0.005415459	0.308444625	0.986	0.981	1	NON Leuk	RPS18
RALGPS2	0.005458223	0.335355182	0.361	0.148	1	NON Leuk	RALGPS2
GNAS	0.005465417	0.347020797	0.687	0.5	1	NON Leuk	GNAS
IGLC1	0.005769033	0.771104292	0.184	0.037	1	NON Leuk	IGLC1
HLA-DRA	0.006202627	0.55433901	0.716	0.593	1	NON Leuk	HLA-DRA
RPL18A	0.006595225	0.341021536	0.992	0.981	1	NON Leuk	RPL18A
P2RX5	0.006625734	0.320534916	0.297	0.111	1	NON Leuk	P2RX5
ATIC	0.006760832	0.189157619	0.121	0	1	NON Leuk	ATIC
CBX3	0.007283704	0.273464077	0.325	0.148	1	NON Leuk	CBX3
SENP6	0.007653489	0.24301904	0.211	0.056	1	NON Leuk	SENP6
PLD4	0.008028315	0.391085002	0.226	0.074	1	NON Leuk	PLD4
RPS8	0.008207078	0.344459842	0.994	0.981	1	NON Leuk	RPS8
BLK	0.008368371	0.257574418	0.15	0.019	1	NON Leuk	BLK
RPL22	0.008478792	0.274206337	0.924	0.889	1	NON Leuk	RPL22
CCT2	0.008502231	0.302445024	0.291	0.13	1	NON Leuk	CCT2
BTG1	0.008609931	0.444168559	0.856	0.741	1	NON Leuk	BTG1
RPL18	0.009689053	0.281726268	0.994	0.981	1	NON Leuk	RPL18
BRD7	0.009709722	0.245705182	0.228	0.074	1	NON Leuk	BRD7
STK24	0.009797315	0.269056109	0.193	0.056	1	NON Leuk	STK24
HMCES	0.009839412	0.230427722	0.175	0.037	1	NON Leuk	HMCES
LTB	0.009957377	0.465648299	0.653	0.519	1	NON Leuk	LTB
