# Supplementary material for: High CD44 expression and enhanced E-selectin binding identified as biomarkers of chemoresistant leukemic cells in human T-ALL
Source: Leukemia. 2024 Nov 24;39(2):323–36. doi: 10.1038/s41375-024-02473-7 (PMC11794132; doi:10.1038/s41375-024-02473-7)
Supplement: Supplementary file 15 — Supplemental Table 14 [file 41375_2024_2473_MOESM15_ESM.pdf]

upregulated genes in Ki67neg CD44high normal cells from Library 4 (Supplementary Figure 12d)

	p_val	avg_log2FC	pct.1	pct.2	p_val_adj	cluster	gene
CD44	1.38E-38	1.501592907	1	0.268	5.05E-34	CD44 > 1 & MKI67 < 1 NON Leuk	CD44
RPL27A	1.35E-06	0.782853112	0.86	0.621	0.049553109	CD44 > 1 & MKI67 < 1 NON Leuk	RPL27A
RPL30	4.15E-06	0.686070494	0.977	0.928	0.152026703	CD44 > 1 & MKI67 < 1 NON Leuk	RPL30
TRBV4-2	8.48E-06	0.548753402	0.116	0.013	0.310352113	CD44 > 1 & MKI67 < 1 NON Leuk	TRBV4-2
MAP3K1	1.51E-05	0.229056207	0.14	0.021	0.551587233	CD44 > 1 & MKI67 < 1 NON Leuk	MAP3K1
RPL19	1.72E-05	0.547829877	1	0.862	0.630286849	CD44 > 1 & MKI67 < 1 NON Leuk	RPL19
HLA-A	1.76E-05	0.621884491	0.953	0.813	0.643461605	CD44 > 1 & MKI67 < 1 NON Leuk	HLA-A
B2M	1.99E-05	0.579898904	1	0.894	0.727652679	CD44 > 1 & MKI67 < 1 NON Leuk	B2M
RPS27	2.80E-05	0.65424113	0.953	0.855	1	CD44 > 1 & MKI67 < 1 NON Leuk	RPS27
RPS15A	3.50E-05	0.556476869	1	0.913	1	CD44 > 1 & MKI67 < 1 NON Leuk	RPS15A
RPL28	3.54E-05	0.49157367	1	0.898	1	CD44 > 1 & MKI67 < 1 NON Leuk	RPL28
RPL18	4.34E-05	0.564388407	1	0.84	1	CD44 > 1 & MKI67 < 1 NON Leuk	RPL18
RPL34	4.71E-05	0.567552741	0.977	0.819	1	CD44 > 1 & MKI67 < 1 NON Leuk	RPL34
RPL37A	4.95E-05	0.431023625	0.907	0.766	1	CD44 > 1 & MKI67 < 1 NON Leuk	RPL37A
RPL11	0.000107696	0.493317394	0.977	0.898	1	CD44 > 1 & MKI67 < 1 NON Leuk	RPL11
HLA-B	0.000145078	0.6343127	0.977	0.76	1	CD44 > 1 & MKI67 < 1 NON Leuk	HLA-B
ZYX	0.000180687	0.498976834	0.419	0.213	1	CD44 > 1 & MKI67 < 1 NON Leuk	ZYX
RPL36	0.000193799	0.526620271	0.907	0.762	1	CD44 > 1 & MKI67 < 1 NON Leuk	RPL36
RPS14	0.000236005	0.491312138	0.977	0.877	1	CD44 > 1 & MKI67 < 1 NON Leuk	RPS14
RPS23	0.000243843	0.510124411	0.977	0.853	1	CD44 > 1 & MKI67 < 1 NON Leuk	RPS23
TMSB10	0.000249966	0.542765652	0.93	0.777	1	CD44 > 1 & MKI67 < 1 NON Leuk	TMSB10
EIF1	0.000334627	0.55994638	0.953	0.883	1	CD44 > 1 & MKI67 < 1 NON Leuk	EIF1
ZFP36	0.00035411	0.847660944	0.767	0.581	1	CD44 > 1 & MKI67 < 1 NON Leuk	ZFP36
ITK	0.000388846	0.361997297	0.256	0.089	1	CD44 > 1 & MKI67 < 1 NON Leuk	ITK
EEF1A1	0.000399712	0.476875184	0.977	0.913	1	CD44 > 1 & MKI67 < 1 NON Leuk	EEF1A1
RPL27	0.00046817	0.4694185	0.907	0.764	1	CD44 > 1 & MKI67 < 1 NON Leuk	RPL27
RPL32	0.000569509	0.451663715	0.977	0.911	1	CD44 > 1 & MKI67 < 1 NON Leuk	RPL32
NOP53	0.000573473	0.643744126	0.605	0.436	1	CD44 > 1 & MKI67 < 1 NON Leuk	NOP53
BTG1	0.0005998	0.536205187	0.837	0.677	1	CD44 > 1 & MKI67 < 1 NON Leuk	BTG1
RPL41	0.000615882	0.446260963	1	0.945	1	CD44 > 1 & MKI67 < 1 NON Leuk	RPL41
RPS6	0.000649943	0.429110878	1	0.845	1	CD44 > 1 & MKI67 < 1 NON Leuk	RPS6
RPL35A	0.000658572	0.474277545	0.977	0.864	1	CD44 > 1 & MKI67 < 1 NON Leuk	RPL35A
CD52	0.000715222	0.714330187	0.767	0.557	1	CD44 > 1 & MKI67 < 1 NON Leuk	CD52
RPL22	0.000764362	0.342805636	0.93	0.743	1	CD44 > 1 & MKI67 < 1 NON Leuk	RPL22
RPL24	0.000796234	0.472178404	0.953	0.847	1	CD44 > 1 & MKI67 < 1 NON Leuk	RPL24
RPL21	0.000815995	0.444424609	0.93	0.881	1	CD44 > 1 & MKI67 < 1 NON Leuk	RPL21
RPS27A	0.000949825	0.486929025	0.977	0.866	1	CD44 > 1 & MKI67 < 1 NON Leuk	RPS27A
RPS28	0.000951868	0.505630889	0.977	0.86	1	CD44 > 1 & MKI67 < 1 NON Leuk	RPS28
ZNF675	0.001170577	0.232736944	0.116	0.026	1	CD44 > 1 & MKI67 < 1 NON Leuk	ZNF675
ARID5A	0.001181186	0.391875085	0.233	0.089	1	CD44 > 1 & MKI67 < 1 NON Leuk	ARID5A
TPT1	0.001186708	0.485460218	0.93	0.934	1	CD44 > 1 & MKI67 < 1 NON Leuk	TPT1
RPL17	0.001253191	0.416564738	0.93	0.809	1	CD44 > 1 & MKI67 < 1 NON Leuk	RPL17
RPL37	0.001286455	0.455962763	0.977	0.834	1	CD44 > 1 & MKI67 < 1 NON Leuk	RPL37
RPLP1	0.001331548	0.413341381	1	0.938	1	CD44 > 1 & MKI67 < 1 NON Leuk	RPLP1
CD48	0.001356542	0.450388333	0.512	0.317	1	CD44 > 1 & MKI67 < 1 NON Leuk	CD48
EIF4A2	0.001395132	0.532803387	0.512	0.362	1	CD44 > 1 & MKI67 < 1 NON Leuk	EIF4A2
SOCS3	0.001420281	0.229406012	0.116	0.026	1	CD44 > 1 & MKI67 < 1 NON Leuk	SOCS3
TCF7	0.001589227	0.406262877	0.349	0.172	1	CD44 > 1 & MKI67 < 1 NON Leuk	TCF7
RPL29	0.001688965	0.439726023	0.977	0.843	1	CD44 > 1 & MKI67 < 1 NON Leuk	RPL29

RPL26	0.001829722	0.450130281	0.953	0.849	1	CD44 > 1 & MKI67 < 1 NON Leuk	RPL26
RRAS	0.001955422	0.255808548	0.116	0.028	1	CD44 > 1 & MKI67 < 1 NON Leuk	RRAS
KLRC4	0.001963386	0.279675167	0.163	0.051	1	CD44 > 1 & MKI67 < 1 NON Leuk	KLRC4
RPS13	0.001979223	0.552117809	0.977	0.862	1	CD44 > 1 & MKI67 < 1 NON Leuk	RPS13
FNBP1	0.002225836	0.490159866	0.419	0.249	1	CD44 > 1 & MKI67 < 1 NON Leuk	FNBP1
RPS3A	0.002414233	0.420326354	0.953	0.877	1	CD44 > 1 & MKI67 < 1 NON Leuk	RPS3A
HLA-C	0.002513149	0.508027415	0.907	0.766	1	CD44 > 1 & MKI67 < 1 NON Leuk	HLA-C
LTB	0.002752752	0.458540822	0.419	0.24	1	CD44 > 1 & MKI67 < 1 NON Leuk	LTB
RPS4X	0.003063633	0.345333444	0.953	0.815	1	CD44 > 1 & MKI67 < 1 NON Leuk	RPS4X
VPS13D	0.00309476	0.222074648	0.116	0.03	1	CD44 > 1 & MKI67 < 1 NON Leuk	VPS13D
IER2	0.003143502	0.658496258	0.628	0.479	1	CD44 > 1 & MKI67 < 1 NON Leuk	IER2
FBXW4	0.003333647	0.201298907	0.116	0.03	1	CD44 > 1 & MKI67 < 1 NON Leuk	FBXW4
RPLP2	0.003584376	0.404775622	0.93	0.851	1	CD44 > 1 & MKI67 < 1 NON Leuk	RPLP2
PNISR	0.003777613	0.373838965	0.558	0.419	1	CD44 > 1 & MKI67 < 1 NON Leuk	PNISR
NACA	0.003984334	0.354374566	0.884	0.809	1	CD44 > 1 & MKI67 < 1 NON Leuk	NACA
PABPC1	0.004498969	0.459399912	0.744	0.721	1	CD44 > 1 & MKI67 < 1 NON Leuk	PABPC1
C12orf57	0.00464787	0.488736632	0.605	0.5	1	CD44 > 1 & MKI67 < 1 NON Leuk	C12orf57
RPS25	0.004689019	0.391969546	0.907	0.834	1	CD44 > 1 & MKI67 < 1 NON Leuk	RPS25
RPL7A	0.004806523	0.372572053	1	0.874	1	CD44 > 1 & MKI67 < 1 NON Leuk	RPL7A
CCDC18-AS1	0.004857756	0.371719005	0.209	0.089	1	CD44 > 1 & MKI67 < 1 NON Leuk	CCDC18-AS1
RPL14	0.004899437	0.348236371	0.93	0.851	1	CD44 > 1 & MKI67 < 1 NON Leuk	RPL14
UBC	0.00516272	0.333024091	0.953	0.838	1	CD44 > 1 & MKI67 < 1 NON Leuk	UBC
NELL2	0.005620655	0.248513209	0.116	0.032	1	CD44 > 1 & MKI67 < 1 NON Leuk	NELL2
RPL31	0.005734803	0.555674869	0.814	0.691	1	CD44 > 1 & MKI67 < 1 NON Leuk	RPL31
ZFP36L2	0.006427023	0.317212829	0.767	0.609	1	CD44 > 1 & MKI67 < 1 NON Leuk	ZFP36L2
SELPLG	0.006808487	0.313046183	0.395	0.236	1	CD44 > 1 & MKI67 < 1 NON Leuk	SELPLG
TMSB4X	0.00706517	0.410843346	0.977	0.751	1	CD44 > 1 & MKI67 < 1 NON Leuk	TMSB4X
MALAT1	0.007233109	0.450997622	1	0.987	1	CD44 > 1 & MKI67 < 1 NON Leuk	MALAT1
H3F3B	0.00750442	0.495818991	0.907	0.891	1	CD44 > 1 & MKI67 < 1 NON Leuk	H3F3B
RPS15	0.007577245	0.292502736	0.953	0.902	1	CD44 > 1 & MKI67 < 1 NON Leuk	RPS15
FAU	0.007967552	0.34132883	0.93	0.872	1	CD44 > 1 & MKI67 < 1 NON Leuk	FAU
RPL12	0.008103466	0.342684001	0.93	0.879	1	CD44 > 1 & MKI67 < 1 NON Leuk	RPL12
LEPROTL1	0.008282386	0.466592833	0.535	0.402	1	CD44 > 1 & MKI67 < 1 NON Leuk	LEPROTL1
MT-CO1	0.008363982	0.264069911	1	0.991	1	CD44 > 1 & MKI67 < 1 NON Leuk	MT-CO1
RPS16	0.008393869	0.481714932	0.907	0.794	1	CD44 > 1 & MKI67 < 1 NON Leuk	RPS16
ZDHHC5	0.008579869	0.114850776	0.116	0.034	1	CD44 > 1 & MKI67 < 1 NON Leuk	ZDHHC5
VCPKMT	0.008684789	0.289400572	0.186	0.079	1	CD44 > 1 & MKI67 < 1 NON Leuk	VCPKMT
RPL18A	0.008796387	0.338806159	0.953	0.874	1	CD44 > 1 & MKI67 < 1 NON Leuk	RPL18A
ITGB2	0.008977503	0.270173641	0.605	0.415	1	CD44 > 1 & MKI67 < 1 NON Leuk	ITGB2
RPL23	0.009392047	0.41298508	0.558	0.445	1	CD44 > 1 & MKI67 < 1 NON Leuk	RPL23
FBRSL1	0.009630086	0.281475871	0.116	0.036	1	CD44 > 1 & MKI67 < 1 NON Leuk	FBRSL1
SPOCK2	0.009805213	0.506169907	0.349	0.211	1	CD44 > 1 & MKI67 < 1 NON Leuk	SPOCK2
UBB	1.73E-05	0.902116987	0.832	0.581	0.631910488	NON Leuk	UBB
CAT	0.000116417	0.494425285	0.315	0.023	1	NON Leuk	CAT
ALAS2	0.000129963	1.34990753	0.309	0.023	1	NON Leuk	ALAS2
PSMD4	0.000146976	0.319099391	0.345	0.047	1	NON Leuk	PSMD4
SLC25A39	0.000149916	0.81261463	0.44	0.14	1	NON Leuk	SLC25A39
PA2G4	0.000180851	0.348069529	0.336	0.047	1	NON Leuk	PA2G4
HIST1H4C	0.000201152	1.140665954	0.432	0.14	1	NON Leuk	HIST1H4C
HBA1	0.000246809	1.728782668	0.315	0.047	1	NON Leuk	HBA1
GHITM	0.000258364	0.342896265	0.355	0.07	1	NON Leuk	GHITM
BCL2L1	0.000273524	0.456511426	0.245	0	1	NON Leuk	BCL2L1

AHSP	0.000274256	1.747864834	0.319	0.047	1	NON Leuk	AHSP
BLVRB	0.000311856	1.189414857	0.343	0.07	1	NON Leuk	BLVRB
RAD23A	0.000318744	0.603627474	0.511	0.209	1	NON Leuk	RAD23A
SLC25A37	0.000318953	0.98212643	0.34	0.07	1	NON Leuk	SLC25A37
BRK1	0.000359748	0.339891549	0.4	0.093	1	NON Leuk	BRK1
SNCA	0.000421226	0.842806021	0.304	0.047	1	NON Leuk	SNCA
CA1	0.000435002	1.8979385	0.309	0.047	1	NON Leuk	CA1
EIF1AY	0.000471561	0.41131099	0.266	0.023	1	NON Leuk	EIF1AY
MXI1	0.000623446	0.368373384	0.221	0	1	NON Leuk	MXI1
HBA2	0.000643104	1.922390701	0.313	0.07	1	NON Leuk	HBA2
DPM2	0.00072196	0.245000442	0.217	0	1	NON Leuk	DPM2
SELENBP1	0.000746496	0.572234488	0.253	0.023	1	NON Leuk	SELENBP1
MKRN1	0.000815093	0.459401433	0.251	0.023	1	NON Leuk	MKRN1
HBD	0.000829066	1.576569372	0.326	0.07	1	NON Leuk	HBD
CAPZA2	0.000898129	0.234353434	0.211	0	1	NON Leuk	CAPZA2
FECH	0.000915599	0.364338364	0.255	0.023	1	NON Leuk	FECH
HAGH	0.000922822	0.390392946	0.249	0.023	1	NON Leuk	HAGH
STRADB	0.000965498	0.327699246	0.209	0	1	NON Leuk	STRADB
TSTA3	0.000965498	0.249513576	0.209	0	1	NON Leuk	TSTA3
SLC4A1	0.001043087	0.829657401	0.281	0.047	1	NON Leuk	SLC4A1
EPB42	0.001083631	0.450293877	0.249	0.023	1	NON Leuk	EPB42
NCOA4	0.001108838	0.440848329	0.345	0.093	1	NON Leuk	NCOA4
MAD2L2	0.001197873	0.221967179	0.202	0	1	NON Leuk	MAD2L2
GSPT1	0.001206219	0.34609946	0.381	0.116	1	NON Leuk	GSPT1
FAM210B	0.00124791	0.395252052	0.243	0.023	1	NON Leuk	FAM210B
GYPC	0.001282254	0.535634018	0.704	0.419	1	NON Leuk	GYPC
TFDP1	0.001384222	0.302846073	0.272	0.047	1	NON Leuk	TFDP1
TUBA1B	0.001439215	1.152280717	0.481	0.279	1	NON Leuk	TUBA1B
GSTO1	0.001440929	0.214294793	0.279	0.047	1	NON Leuk	GSTO1
GLRX5	0.00145568	0.563727	0.457	0.186	1	NON Leuk	GLRX5
MTCH2	0.001483266	0.183890275	0.196	0	1	NON Leuk	MTCH2
MCM5	0.001592098	0.247609533	0.194	0	1	NON Leuk	MCM5
HMBS	0.00166174	0.685023522	0.294	0.07	1	NON Leuk	HMBS
LGALS3	0.00167675	0.451783346	0.309	0.07	1	NON Leuk	LGALS3
DCAF12	0.001708554	0.428209832	0.191	0	1	NON Leuk	DCAF12
AMD1	0.001811988	0.247472411	0.234	0.023	1	NON Leuk	AMD1
SPTA1	0.001833146	0.390504195	0.189	0	1	NON Leuk	SPTA1
MPP1	0.001885011	0.400733887	0.26	0.047	1	NON Leuk	MPP1
SMC4	0.001889332	0.271170101	0.268	0.047	1	NON Leuk	SMC4
AP2M1	0.001922683	0.296689368	0.368	0.116	1	NON Leuk	AP2M1
CA2	0.001962308	1.185154801	0.226	0.023	1	NON Leuk	CA2
PDZK1IP1	0.001966418	0.349372973	0.187	0	1	NON Leuk	PDZK1IP1
PCNA	0.001966418	0.25095808	0.187	0	1	NON Leuk	PCNA
TIMM8B	0.001966418	0.188932617	0.187	0	1	NON Leuk	TIMM8B
MPC2	0.001981628	0.302407488	0.336	0.093	1	NON Leuk	MPC2
TPGS2	0.002012977	0.242033639	0.226	0.023	1	NON Leuk	TPGS2
KLF1	0.002108945	0.358882785	0.185	0	1	NON Leuk	KLF1
GMPS	0.002261344	0.19323411	0.183	0	1	NON Leuk	GMPS
SMC2	0.002261344	0.189555542	0.183	0	1	NON Leuk	SMC2
HBM	0.00245532	1.034460921	0.298	0.07	1	NON Leuk	HBM
TUBB2A	0.0024785	0.365314555	0.257	0.047	1	NON Leuk	TUBB2A
PYURF	0.002497337	0.217858208	0.223	0.023	1	NON Leuk	PYURF

UROD	0.002550512	0.452587956	0.317	0.093	1	NON Leuk	UROD
TMEM14C	0.002556496	0.371792766	0.221	0.023	1	NON Leuk	TMEM14C
GATA1	0.002598411	0.290759879	0.179	0	1	NON Leuk	GATA1
ANP32B	0.002748469	0.441559438	0.549	0.256	1	NON Leuk	ANP32B
PRDX2	0.00294979	0.860866275	0.532	0.279	1	NON Leuk	PRDX2
COX7B	0.002972264	0.134148642	0.485	0.186	1	NON Leuk	COX7B
TYMS	0.002980555	0.268625169	0.219	0.023	1	NON Leuk	TYMS
FRYL	0.002983363	0.244292942	0.174	0	1	NON Leuk	FRYL
UFD1	0.003080888	0.209683532	0.255	0.047	1	NON Leuk	UFD1
GUK1	0.003134291	0.393840016	0.666	0.372	1	NON Leuk	GUK1
PPIH	0.003195797	0.159608269	0.172	0	1	NON Leuk	PPIH
CENPF	0.003324911	0.391179514	0.209	0.023	1	NON Leuk	CENPF
H1F0	0.003378842	0.623115861	0.243	0.047	1	NON Leuk	H1F0
JAZF1	0.003422702	0.256945174	0.17	0	1	NON Leuk	JAZF1
CD47	0.003459779	0.161435907	0.332	0.093	1	NON Leuk	CD47
RRM2	0.003665023	0.331835922	0.168	0	1	NON Leuk	RRM2
GYPB	0.003834486	0.480923924	0.206	0.023	1	NON Leuk	GYPB
PMAIP1	0.003888709	0.347274441	0.285	0.07	1	NON Leuk	PMAIP1
MKI67	0.004019996	0.346330555	0.24	0.047	1	NON Leuk	MKI67
SUGT1	0.004106025	0.162974977	0.211	0.023	1	NON Leuk	SUGT1
ZNF326	0.004200006	0.176708783	0.164	0	1	NON Leuk	ZNF326
BIRC5	0.004200008	0.18853118	0.164	0	1	NON Leuk	BIRC5
RRM1	0.004200008	0.154377887	0.164	0	1	NON Leuk	RRM1
H2AFX	0.004221966	0.218657081	0.209	0.023	1	NON Leuk	H2AFX
DEK	0.004246889	0.314001511	0.398	0.14	1	NON Leuk	DEK
DNMT1	0.00432704	0.188756564	0.209	0.023	1	NON Leuk	DNMT1
HEMGN	0.004455081	0.615518297	0.236	0.047	1	NON Leuk	HEMGN
TMEM167A	0.004494873	0.180387685	0.162	0	1	NON Leuk	TMEM167A
SQSTM1	0.004515621	0.201862316	0.249	0.047	1	NON Leuk	SQSTM1
ATP5IF1	0.004619433	0.443684859	0.5	0.256	1	NON Leuk	ATP5IF1
ENY2	0.00471445	0.161858439	0.287	0.07	1	NON Leuk	ENY2
CCDC124	0.004718297	0.14319441	0.206	0.023	1	NON Leuk	CCDC124
NME1	0.004758225	0.221942971	0.2	0.023	1	NON Leuk	NME1
WNK1	0.004800891	0.19246366	0.289	0.07	1	NON Leuk	WNK1
NFIX	0.004809572	0.266660868	0.16	0	1	NON Leuk	NFIX
UBE2T	0.004809572	0.181790819	0.16	0	1	NON Leuk	UBE2T
INTS11	0.004809572	0.163182611	0.16	0	1	NON Leuk	INTS11
POP7	0.004809572	0.146030029	0.16	0	1	NON Leuk	POP7
NUSAP1	0.005035783	0.358200492	0.198	0.023	1	NON Leuk	NUSAP1
SUCLG1	0.005187132	0.194254689	0.198	0.023	1	NON Leuk	SUCLG1
RBX1	0.005235189	0.307872416	0.462	0.209	1	NON Leuk	RBX1
SMAP2	0.005262526	0.302029287	0.313	0.093	1	NON Leuk	SMAP2
POLR2G	0.005351535	0.228675576	0.272	0.07	1	NON Leuk	POLR2G
UBE2G1	0.005355043	0.223012867	0.196	0.023	1	NON Leuk	UBE2G1
MIEN1	0.005484563	0.219304827	0.234	0.047	1	NON Leuk	MIEN1
TUBG1	0.005503689	0.197356953	0.155	0	1	NON Leuk	TUBG1
ZWINT	0.005503689	0.148049013	0.155	0	1	NON Leuk	ZWINT
AK2	0.005503689	0.143416724	0.155	0	1	NON Leuk	AK2
RBMX	0.005527838	0.196782994	0.281	0.07	1	NON Leuk	RBMX
NDUFB3	0.005531269	0.206266282	0.236	0.047	1	NON Leuk	NDUFB3
PRPF40A	0.00572863	0.186986626	0.277	0.07	1	NON Leuk	PRPF40A
HIST1H3B	0.005885917	0.244622497	0.153	0	1	NON Leuk	HIST1H3B

HMGB3	0.00588592	0.129284685	0.153	0	1	NON Leuk	HMGB3
CMAS	0.005907684	0.212497069	0.196	0.023	1	NON Leuk	CMAS
MRPL51	0.005933935	0.201591506	0.272	0.07	1	NON Leuk	MRPL51
CYC1	0.005984956	0.224717099	0.272	0.07	1	NON Leuk	CYC1
CKS2	0.006088187	0.273867754	0.272	0.07	1	NON Leuk	CKS2
CYB5R3	0.006263444	0.19446773	0.196	0.023	1	NON Leuk	CYB5R3
FEN1	0.006293624	0.140606568	0.151	0	1	NON Leuk	FEN1
YWHAH	0.00638521	0.245242066	0.226	0.047	1	NON Leuk	YWHAH
BSG	0.006420199	0.583212355	0.577	0.349	1	NON Leuk	BSG
TXNL1	0.006516455	0.168012104	0.234	0.047	1	NON Leuk	TXNL1
MINPP1	0.006728438	0.289397757	0.149	0	1	NON Leuk	MINPP1
CCS	0.006867339	0.182620075	0.236	0.047	1	NON Leuk	CCS
CBX5	0.006987691	0.112842139	0.194	0.023	1	NON Leuk	CBX5
TK1	0.007192102	0.169214641	0.147	0	1	NON Leuk	TK1
ATAD2	0.007192102	0.167012181	0.147	0	1	NON Leuk	ATAD2
BLVRA	0.007192102	0.144365105	0.147	0	1	NON Leuk	BLVRA
ACAT1	0.007192102	0.125746073	0.147	0	1	NON Leuk	ACAT1
HAUS4	0.007192102	0.124094042	0.147	0	1	NON Leuk	HAUS4
CANX	0.007210296	0.257594731	0.266	0.07	1	NON Leuk	CANX
MAPRE1	0.007320083	0.178478151	0.187	0.023	1	NON Leuk	MAPRE1
GLUL	0.007507988	0.235520572	0.304	0.093	1	NON Leuk	GLUL
YBX3	0.007550626	0.347196959	0.362	0.14	1	NON Leuk	YBX3
ST6GALNAC4	0.007686463	0.205728875	0.145	0	1	NON Leuk	ST6GALNAC4
MYBL2	0.007686467	0.153009532	0.145	0	1	NON Leuk	MYBL2
TMEM106C	0.007686467	0.142371002	0.145	0	1	NON Leuk	TMEM106C
MCM6	0.007686467	0.127840437	0.145	0	1	NON Leuk	MCM6
CENPU	0.007967855	0.175707468	0.185	0.023	1	NON Leuk	CENPU
COX6C	0.008021938	0.303790252	0.56	0.326	1	NON Leuk	COX6C
NAXE	0.008081383	0.114640428	0.189	0.023	1	NON Leuk	NAXE
TOP2A	0.008213499	0.202090721	0.143	0	1	NON Leuk	TOP2A
CZIB	0.008213499	0.148471104	0.143	0	1	NON Leuk	CZIB
CDT1	0.008213499	0.125180797	0.143	0	1	NON Leuk	CDT1
HBB	0.008227018	2.642443774	0.394	0.233	1	NON Leuk	HBB
PCM1	0.008858264	0.214675167	0.296	0.093	1	NON Leuk	PCM1
PSMD13	0.008993187	0.115610482	0.223	0.047	1	NON Leuk	PSMD13
RAB10	0.009163265	0.171293805	0.181	0.023	1	NON Leuk	RAB10
GADD45A	0.009183071	0.171637789	0.226	0.047	1	NON Leuk	GADD45A
CBX3	0.009259289	0.214770522	0.362	0.14	1	NON Leuk	CBX3
PSME4	0.009374054	0.190409209	0.138	0	1	NON Leuk	PSME4
MAN2A1	0.009374054	0.180325892	0.138	0	1	NON Leuk	MAN2A1
VPS35	0.009374054	0.147234754	0.138	0	1	NON Leuk	VPS35
NSL1	0.009374054	0.138357112	0.138	0	1	NON Leuk	NSL1
WBP2	0.009423453	0.160958733	0.26	0.07	1	NON Leuk	WBP2
NDUFAF3	0.009482485	0.151424992	0.223	0.047	1	NON Leuk	NDUFAF3
PNP	0.009514241	0.252680769	0.296	0.093	1	NON Leuk	PNP
CAPRIN1	0.009589955	0.155078256	0.179	0.023	1	NON Leuk	CAPRIN1
ATP5MPL	0.009627394	0.176060665	0.402	0.163	1	NON Leuk	ATP5MPL
SLC2A1	0.009640284	0.293961847	0.211	0.047	1	NON Leuk	SLC2A1
CKAP2	0.009790557	0.197487529	0.221	0.047	1	NON Leuk	CKAP2
PGAM1	0.009907686	0.275087282	0.281	0.093	1	NON Leuk	PGAM1
SRRT	0.009942593	0.170216574	0.181	0.023	1	NON Leuk	SRRT
