# Supplementary material for: High CD44 expression and enhanced E-selectin binding identified as biomarkers of chemoresistant leukemic cells in human T-ALL
Source: Leukemia. 2024 Nov 24;39(2):323–36. doi: 10.1038/s41375-024-02473-7 (PMC11794132; doi:10.1038/s41375-024-02473-7)
Supplement: Supplementary file 16 — Supplemental Table 15 [file 41375_2024_2473_MOESM16_ESM.pdf]

Common genes of Ki67neg CD44high nomal cells from all libraries (Supplementary Figure 12e)

IL1B
PLAUR
IL1RN
BCL2A1
VIM
THBS1
CXCL8
FTH1
FCN1
ATP2B1-AS1
CCL3
SPHK1
CXCL2
SERPINB2
C15orf48
CST3
CCL3L1
IER3
MAFB
CTSL
AC015912.3
LYZ
SOD2
LGALS3
BASP1
CD68
DRAM1
IFNGR2
S100A10
ICAM1
CSTB
CCL4L2
S100A9
DNAAF1
SERPINA1
PTGS2
ANXA5
S100A8
PHLDA1
IFI30
TYROBP
S100A6
FCER1G
CCL4
CD63

MAP3K8
MSC
G0S2
PPP1R15A
ITGB8
S100A12
HLA-DRA
AIF1
TXN
LGALS1
CD83
S100A4
NRP2
CSTA
HLA-DRB1
EHD1
ATP6V1F
TIMP1
AQP9
UBE2D1
NFKBIA
FLT1
RNF144B
ZEB2
CYTOR
ITGAX
CLEC7A
CTSS
FTL
S100A11
CPVL
BID
EREG
GRN
CXCL16
PLEK
JARID2
NINJ1
SAT1
PSAP
NAMPT
PKM
APLP2
ETS2
CFP
CD74
DENND5A

PALM2-AKAP2
GAPDH
CEBPD
SPI1
SH3BGRL3
F3
LUCAT1
SMS
NLRP3
HLA-DQB1
USP12
IL6
MIR3945HG
TSPO
INSIG1
CFD
PDE4DIP
RIN3
GABARAP
OAZ1
PID1
CTSD
INHBA
DUSP6
NFKBIZ
SRGN
RGCC
APOBEC3A
CYBA
IL1R1
GRINA
MGLL
SMOX
NPC2
OGFRL1
ATP2B1
NCF2
PDLIM5
CD300C
MTSS1
BZW1
TMSB10
PTPRJ
BACH1
PNP
RIN2
CXCL3

SDC2
ADM
CCL7
ALDOA
CSF3R
TREM1
MET
CTSB
UBE2J1
MPP1
FCGRT
TNFRSF1B
PMAIP1
SLC25A37
MSANTD3
SLC16A6
METRNL
RASGEF1B
MARCKS
PTX3
KYNU
KLF10
GNG5
DSE
SERPINB9
TNIP1
TMSB4X
ATP13A3
FCGR2A
ANXA1
ANXA2
C19orf38
MS4A7
TUBB6
PGAM1
SLC7A7
BRI3
ASPH
PDXK
CARD16
CDC42EP3
DMXL2
WTAP
PTPRE
PPIF
PELI1
TYMP

CREG1
HLA-DRB5
FPR1
SLC43A2
GSTO1
SNX18
CHMP4B
PSMA6
EMILIN2
MYO9B
CCRL2
SPRY2
SLC16A3
AZIN1-AS1
PRKAG2
ZFAND5
NRGN
P4HB
CCDC71L
CDKN2B
CD300E
TJP2
IL3RA
LST1
ACSL1
ODF3B
ID2
AHR
TPI1
BNIP3L
LCP1
AC007032.1
PIM3
RNF145
SERPINB1
PRDM1
SLC11A1
CASP1
LGALS2
STX11
ECE1
MYC
HOTAIRM1
MIR22HG
CTNNB1
GCH1
PRKAG2-AS1

SLC15A3
TKT
ETV3
SCPEP1
NEDD4L
CFL1
PILRA
FNIP2
SIRPA
TLR2
LHFPL2
MFSD2A
NEAT1
HIF1A
LYN
ARFGAP3
HLA-DPA1
IL6R
HLA-DPB1
LILRB4
LILRA5
SERTAD2
MAP1LC3B
CDA
HLX
ARL8B
LILRB2
AC004130.2
SEMA6B
CES1
ARPC5
CHST2
MYL6
MAP2K3
TPM4
ITGB2
VEGFA
CASP4
FOXO3
SLC7A5
B4GALT5
SLC2A6
IFIT3
SLC25A6
SDCBP
OASL
AGTRAP

AL118516.1
TLR4
EIF1B
PIK3AP1
EPB41L3
EIF4A1
SAMSN1
TNFAIP8
IQGAP1
GADD45A
MXD1
PLBD1
MED13L
PLD3
SLCO3A1
IRAK2
CNPY3
PRELID1
PIK3R5
TBC1D12
ADAMTS2
AP1S2
CCL20
NFKB1
IRAK1
ADORA2A
QKI
MIR4435-2HG
TPST1
TIMP2
C5AR1
ARRB2
MT-ND1
WARS
PHLDA2
LMNA
CD14
MAP4K4
WDR1
HS3ST3B1
FLOT1
LRRFIP2
FOSL2
SEC11A
ST3GAL1
TPRA1
LIMS1

TRAF1
RNF130
SNX8
FNDC3B
ATP6V0B
PLSCR1
EHD4
PITPNA
ETHE1
RNF19B
DAPK1
MGAT1
RHOA
ABHD17C
FLNA
VIM-AS1
ZNF385A
SOCS3
LY86
DUSP5
GPX4
HLA-DMA
ELL2
COTL1
SPAG9
MARCKSL1
RAB32
B3GNT5
TMED5
CHST7
CD302
NPC1
RAB31
ISG15
LILRA2
ZMIZ1
MT-CO1
CCL2
CD109
KLF4
UFM1
SLC7A11
GBP2
HMGA1
KCNJ2-AS1
HS3ST1
AK4

MAN2B1
ADA
ZNFX1
GPRC5A
TNFAIP2
PICALM
CREB5
OTUD1
GRAMD2B
SLC43A3
TENT5A
CFLAR
LINC-PINT
RGS10
IFI27L2
TP53INP2
SNX9
TGIF1
ATP6V1B2
MT2A
XBP1
KLF6
C15orf39
C1orf122
LAIR1
HCK
ATOX1
ATF5
SMPDL3A
MAFG
RAPGEF1
CCR1
CRTAP
LRP1
FKBP1A
WDR33
GNA13
ENO1
SGK1
TCN2
PNPLA8
MYO10
ANPEP
VCAN
AOAH
QSOX1
MREG

MDM2
CAMTA2
FCAR
IRF7
EMP3
HIVEP2
CYP1B1
LCP2
BCAT1
AC007952.4
CYBB
PLD1
MARCO
VASP
RAP1B
AC006449.6
CTSZ
IGSF6
VOPP1
AZIN1
ARPC1B
AMPD3
GNA15
STAB1
CSF3
RILPL2
PRDM8
YWHAE
TNFRSF21
EIF5
TNF
RNASET2
MANBA
PHACTR1
ALDH2
TLE1
SNX10
P2RX4
SLC6A6
ANKLE2
ABL2
STON2
IL1A
MPZL1
EIF4E
NOTCH1
ASAH1

PTPN12
TPD52L2
ALG2
ACTB
CAPG
CSF2
FAM49A
H3F3A
CD53
LINC00884
PPP3CC
OXSR1
NRIP3
RABGEF1
ARPC2
ALOX5AP
TSC22D2
MS4A6A
MAPK6
ZADH2
CD82
TNFRSF10B
CAPNS1
KDM7A-DT
DLL1
FAM126A
RUNX1
MYOF
FH
AC009951.6
ARPC3
PARVB
MT-ND4
FNIP1
RAPGEF2
PARP14
CTSH
RAB5A
LONRF1
AHNAK
TMEM158
SLA
WDFY1
IL10
HES4
BCAP31
NFKB2

SLC7A1
LRRC25
LINC01588
CITED2
CD93
TRAPPC5
CDKN2A
AKIRIN2
NOTCH2NLC
ATF4
PLEKHM2
NFAT5
IER3-AS1
EAF1
HNRNPC
CD151
CD58
GPR183
LAPTM5
STXBP2
OAS3
CKB
SMIM3
HLA-DQA1
TPP1
TRIM25
RYBP
GNA12
MCRIP2
GRB2
DOT1L
KBTBD2
CLIC1
SAMD9
CAST
SERPINB9P1
ANO6
SLC2A3
FGFR1
CEBPB
OLR1
MCTP1
CRIP1
AKAP13
MICOS10
DDX3X
ADAM15

SERF2
PGD
PPP3CA
ZYX
RARA
GTPBP4
GLUL
IFIT2
ARHGAP21
UBE2R2
MAD1L1
NOCT
PIK3CB
ARHGEF2
PTEN
TGFBI
MAML2
PABPC4
IFITM3
NRIP1
UPP1
HK2
CARD19
PLEKHO1
TRIP10
ST8SIA4
SLC31A2
SEC61B
MTF1
RHEB
MFSD12
MYD88
H2AFY
GADD45B
LYPD3
ACTR3
NDST2
PDLIM7
ACSL3
NR4A3
PFN1
KMO
SCO2
CDKN1A
MIR155HG
B4GALT1
PTGER2

IL10RA
B3GNT2
NCOR2
LACTB
ITGA5
SOX4
ABCA1
ATP6V1H
BRK1
RTL8C
BLVRB
SDF2L1
ARHGAP31
TAGAP
MAPKAPK2
MID1IP1
HCST
HMGA2
SRC
NANS
IFI6
KDM6B
ANAPC15
CLEC4E
AP2S1
LPXN
SPSB1
ERO1A
IRF2BP2
RIPK2
GPR84
LGALS9
C11orf96
GBP1
STK40
BAALC
ADGRE2
CAMKK2
BTG1
ETV6
REL
CDC37
PTGER4
TFDP1
IL4I1
SPAG7
FOSL1

ZC3H12A
ANTXR2
TWISTNB
RAC1
MAP1LC3A
ZCCHC2
RB1CC1
TAGLN2
TLNRD1
ZBTB43
SLC39A8
TMEM219
FMNL1
DBI
RBM23
MRPS24
GLS
CANX
SQLE
PTTG1IP
LRRC8C
ERCC1
GLRX
ZNF655
SLC12A7
YBX1
RFX2
IRAK3
SRA1
MSN
CD55
PLIN2
PSMG3
LGALS8
DUSP4
MAFF
H1F0
GSTP1
ERP29
BTG3
TNFSF14
ITPRID2
RELT
VTI1B
ARF5
SSR3
LRRFIP1

RSAD2
RBM3
KDM5B
STK10
NFATC1
LDHA
ERVK3-1
NBDY
CHIC2
ARHGDIA
TNFSF8
MCUB
NR3C1
TOMM7
RPS27A
DYRK2
ISG20
RPL38
UBA52
FAU
RPS15A
SLC35D2
KLF2
NDUFV2-AS1
SNTB2
RPS26
GAS5
ETS1
RPL11
THEMIS
RPS25
TNFAIP3
TXNIP
KCTD2
MIR4435-2HG
S100A8
CSF3R
RXRA
HRH2
VCAN
RNF130
CFD
FCN1
S100A10
CD36
TGFBI
LYZ

NR4A1
C3AR1
S100A9
CLEC12A
LGALS2
SERPINA1
CD14
CFP
MNDA
TYROBP
SLC31A2
RPL27A
RPL30
TRBV4-2
MAP3K1
RPL19
HLA-A
B2M
