# Supplementary material for: High CD44 expression and enhanced E-selectin binding identified as biomarkers of chemoresistant leukemic cells in human T-ALL
Source: Leukemia. 2024 Nov 24;39(2):323–36. doi: 10.1038/s41375-024-02473-7 (PMC11794132; doi:10.1038/s41375-024-02473-7)
Supplement: Supplementary file 17 — Supplemental Table 16 [file 41375_2024_2473_MOESM17_ESM.pdf]

Exclusif genes of Ki67neg CD44high leukemic cells compared to Ki67neg CD44high normal cells (Supplementary Figure 12f)

LTB
SH3BP5
MALAT1
ARL4C
FOS
HLA-E
PNRC1
LIMD2
HLA-B
TSC22D3
JUND
SF1
HLA-C
EIF1
