# Supplementary material for: High CD44 expression and enhanced E-selectin binding identified as biomarkers of chemoresistant leukemic cells in human T-ALL
Source: Leukemia. 2024 Nov 24;39(2):323–36. doi: 10.1038/s41375-024-02473-7 (PMC11794132; doi:10.1038/s41375-024-02473-7)
Supplement: Supplementary file 18 — Supplemental Table 17 [file 41375_2024_2473_MOESM18_ESM.pdf]

Antibody name	Clone	Fluorochrome	Reference	Brand	Application
**CD1a**	HI149	eFluor450	48-0019-42	ThermoFisher Scientific	Flow Cytometry
**CD3**	SK7	PerCP-Cy5.5	344808	Biolegend	
**CD4**	OKT4	FITC	317408	Biolegend	
**CD5**	L17F12	PerCP-Cy5.5	364006	Biolegend	
**CD7**	CD7-6B7	APC-Vio770	130-117-677	Miltenyi Biotech	
	CD7-6B7	PE-Cy7	343114	Biolegend	
	CD7-6B7	FITC	343104	Biolegend	
**CD8**	SK1	APC	344722	Biolegend	
**CD34**	581	PE-Cy7	343516	Biolegend	
	581	PerCP-Cy5.5	343522	Biolegend	
**CD44**	IM7	APC-eFluor780	47-0441-82	ThermoFisher Scientific	
**CD44V6**	2F10	PE	FAB3660P	R&D System	
**CD45**	HI30	BV421	304032	Biolegend	
	HI30	PE	304039	Biolegend	
**CD62E**	HCD62E	PE	322606	Biolegend	
**CD162**	KPL-1	PE	328806	Biolegend	
**CD184/CXCR4**	12G5	APC	555976	BD Pharmingen	
**TCRa/b**	IP26	PE	306708	Biolegend	
**CLA**	HECA-452	PE	130-123-707	Miltenyi Biotech	
**Anti-Fc human IgG1**		PE	12-4998-82	ThermoFisher Scientific	
**Ki67 & Isotype Control**		PE	556027	BD Bioscience	
		FITC	556026	BD Bioscience	
**Isotype IgG2a,k**	MOPC-173	PE	981910	Biolegend	
**Human CD44s pan specific**	2C5	Unconjugated	BBA10	Bio-Techne	Western Blot
**b-actin**	AC-74	Unconjugated	A5441	Sigma-Aldrich	
**goat anti-Mouse IgG H&L**		IRDye® 680RD	ab216776	Abcam	
**goat anti-Rabbit IgG H&L**		IRDye® 800CW	ab216773	Abcam	
