# Supplementary material for: High CD44 expression and enhanced E-selectin binding identified as biomarkers of chemoresistant leukemic cells in human T-ALL
Source: Leukemia. 2024 Nov 24;39(2):323–36. doi: 10.1038/s41375-024-02473-7 (PMC11794132; doi:10.1038/s41375-024-02473-7)
Supplement: Supplementary file 19 — Supplemental Table 18 [file 41375_2024_2473_MOESM19_ESM.pdf]

	Forward	Reverse	
**GAPDH**	5'-GGAGGAGTGGGTGTCGCTGTT-3'	5'-GGGAAACTGTGGCGTGAT-3'	
**CD44**	5'-AGCAGCACTTCAGGAGGTTACA-3'	5'-GGTTGTGTTTGCTCCACCTTCT-3'	
**B2m**	5'-CACAGCCCAAGATAGTTAAGT-3'	5'-CCAGCCCTCCTAGAGC-3'	
**CXCR4**	5'-CAGTAGCCACCGCATCTG-3'	5'-GCCCACAATGCCAGTTAAGAA-3'	
**pTa**	5'-GTGTCCAGCCCTACCCAC-3'	5'-ATCCACCAGCAGCATGATTG-3'	
**Hes1**	5'-CAACACGACACCGGATAAACC-3'	5'-CCAGAATGTCCGCCTTC-3'	
**Deltex1**	5'-TTCTGACTTCAGGAGCGAAAG-3'	5'-TGCCCACTCCCAACGA-3'	
**CD44 C16-C17**	5'-GTCCCATACCACTCATGGATCTGA	5'-GGTTGTGTTTGCTCCACCTTCT	All transcripts
**CD44 C5-C17**	5'-AGCAGCACTTCAGGAGGTTACA	5'-GGTTGTGTTTGCTCCACCTTCT	Transcripts containing only constant exons
**CD44 V4-V5**	5'-CAGTGGAACCCAAGCCATTCAA	5'-TGATGCTCATGGTGAATGAGGG	Transcripts including variable exons V4 and V5
**CD44 V5-V6**	5'-GAAACTGGAACCCAGAAGCACA	5'-GTCTTCTTTGGGTGTTTGGCGA	Transcripts including variable exons V5 and V6
**CD44 V6-V7**	5'-CAGAAGGAACAGTGGTTTGGCA	5'-GGGTGTGAGATTGGGTTGAAGA	Transcripts including variable exons V6 and V7
**CD44 V7-V8**	5'-TGCAAGGAAGGACAACACCAAG	5'-AAGAGGTCCTGTCCTGTCCAAA	Transcripts including variable exons V7 and V8
**CD44 V8-V9**	5'-ACGCTTCAGCCTACTGCAAA	5'-GTCAGAGTAGAAGTTGTTGGATGGTC	Transcripts including variable exons V8 and V9
**CD44 V9-V10**	5'-GAGCTTCTCTACATCACATGAAGGC	5'-TAGCTGAGGTCACTGGGATGAA	Transcripts including variable exons V9 and V10
