# Supplementary material for: High CD44 expression and enhanced E-selectin binding identified as biomarkers of chemoresistant leukemic cells in human T-ALL
Source: Leukemia. 2024 Nov 24;39(2):323–36. doi: 10.1038/s41375-024-02473-7 (PMC11794132; doi:10.1038/s41375-024-02473-7)
Supplement: Supplementary file 2 — Supplemental Table 1 [file 41375_2024_2473_MOESM2_ESM.pdf]

	Age	Gender	White Blood Cell Count (G/L)	ALL	CNS involvement	Blood or Bone Marrow	Karyotype	Oncogene	Group	EGIL Grade	Relapse/Refractory	ALIVE	% E selectin binding	Figures	Supplementary Figures
M74	4	M		T		Blood		ND	ND	ND	YES	NO	78.3	5F, 5G	14E
M191	4	M		T/M		Bone Marrow	46,XY,add(15)(q22~23) or (q23~q24)[8]/46,XY[17]	ND	ND	T-II	NO	YES	32.5	2E, 2F, 5F, 5G	3, 5C, 6B, 14E
M82	11	F	194.8	T			46, XX, add or del(11)(q?14-21),<br>del(17)(p11)/46, sl, del(5)(q13q?33),<br>del(12)(p1?3)[2]/46, sdl1, del(9)(q22q34)[9]/46, XX [3]	ND	ND	T-IV	YES	NO	37.6	5F, 5G	14E
M150	7	M	60	T				TLX3	TLX3	T-III	NO	YES	25.5	5F, 5G	14E
M112	3	F	150.1	T			50, XX, +11, +12, +14, + X [4]	TLX3	TLX3	T-III	YES		25.4	5F, 5G	14E
M141	70	M		T/B/M		Blood		ND	ND	ND			24.6	5F, 5G	14E
M158	9	M		T/M		Blood	50, XY, del(1)(p21-22q32-33), add(2) or del(2)(q3?5), del(16)(q11-12), +17, +18, +19, +21 [15]/<br>46, XY [4]	ND	ND	T-II	YES	NO	13.5	5F, 5G	14E
M216	6	F		T		Blood	46,XX,t(8;14)(q24;q11)[22]	MYC<br>SIL-TAL1^{del}	TAL1	T-IV	YES	NO	12.7	5F, 5G	14D, 14E
M107	9	F		T				ND	ND	ND			12.5	5G	14E
M138	42	M	37.7	T			Normal	ND	ND	ND			11	5G	14E
M163	9	F		T		Bone Marrow	46, XX, del(12)(p11p13), del13 (q12q14) [5]/ 46, sl, del(6)(q21q24), del (11) (q14q24-25) [3]	SET-CAN	ND	T-I	YES	YES	11.5	5F, 5G	14E
M166	12	M		T		Blood	46,XY,der(X)t(X;5)(q21;q12),der(5)(5pter-> 5q12::Xq?28->Xqter),inv(14)(q11q32)[26].nuc.	TLX3	TLX3	T-I	YES	YES	10.4	5F, 5G	14E
M99	3	M	517.6	T				ND	ND		NO	YES	9.88	5F, 5G	14E
M101	14	M	233.3	T			46, XY, del(6)(q1?5q2?6), add(7)(p13) or<br>del(7)(p11p?21or?15),+/-*?10p,<br>add(14)(q11)[2?]/46, idem, -16, +mar<br>[?der(16)(?) or ?19][16]/46, idem, -16,<br>add(17)(q.12), +mar [2]	ND	ND	T-I	YES	NO	10.7	5F, 5G	14E
M176	14	M		T			47,XY,+8[13]/46,XY[10]	TLX3	TLX3	T-IV	YES	YES	7.03	5F, 5G	14E
M154								ND	ND	T-IV	NO	NO	3.55	5F, 5G	14E
M201	9	M		T		Bone Marrow	47,XY,dup(6)(q16q21),t(11;14)(p13;q3?1),+20[1]/46,XY[21]	ND	ND	T-IV	YES	NO	2.96	5F	14E
M42	9	M	177.5	T			46, XY, [24]	ND	ND	T-IV	NO	YES	2.97	5F, 5G	14E
M164	11	M	225.4	T		Blood		TLX3	TLX3	T-III	YES	YES	2.94	5F, 5G	14E
M184	7	M	96.14	T		Blood	46,XY[20].nuc ish(ABL1,MLL,BCR)x2[200]	ND	ND	T-III	NO	YES	2.32	5F, 5G	14E
M161		F		T		Blood		ND	ND	ND			2.07	5G	14E
M169	1	M	240	T			46,XX,add(16)(q2?3)[1] /46,sl,ins(6;6)(q1?6;q2?2;q2?4)[19]/<br>46,XX[2]; t(6;7) Rearrangement locus TCRb(7q34) Addition sur le bras court du chromosome 12	ND	ND	T-III	NO	YES	1.66	5F, 5G	14E
M205	9	M		T/M		Bone Marrow	45,XY,del(6)(q1?6q22),del(11)(p1?2p13)[9]/46,sl,t(3;3)(q2?5;q2?7)[7]/46,XY[6]	ND	ND	T-IV	NO	YES	1.39	5C, 5E, 5F, 5G	14E
M193R	11	M	180	T			SIL-TAL1, del CDK2A/B, del CASP8AP2 et LEF, del insterstitiel 6q16-24	SIL-TAL1^{del}	TAL1	T-IV	YES	NO	1.18	2E, 2F, 5F, 5G	3, 5C, 6B, 6D, 6E, 6F, 6G, 14E
M173	2	F	33.43	T		Blood	46, XX[20]	ND	ND	T-III	NO	YES	0.83	5F, 5G	14E
M165	12	F		T		Blood	46, XX, inv7(p15q34) add21(q22)[16]/ 46, XX [4]	ND	ND	T-II	NO	YES	0.81	5F	14E
M182	5	M		T		Bone Marrow	47,XY,+8[2]/46,XY[28]	TLX3	TLX3	T-III	NO	YES	0.69	5F, 5G	14E
M172	13	M		T		Bone Marrow	45, XY, -21 [3]/ 46, XY [23]	ND	ND	T-II	YES	YES	0.64	5F, 5G	11A, 11B, 11C, 11D, 11E, 12D, 12E, 12F, 14E
M155	9	M		T			46,XY,del(9)(q12q31),add(9)(q34)[6]/46,XY[10]	ND	ND	T-IV	NO	YES	0.46	5F, 5G	14D, 14E
M144	3	M	220	T		Blood	46, XY, [21]	SIL-TAL1^{del}	TAL1	T-IV	YES	NO	0.38	5F, 5G	11A, 11B, 11C, 11D, 11E, 12D, 12E, 12F, 14E
M183	4	M		T		Blood	47,XY,del(9)(p24p1?3),add(14)(q32),+mar[18]/46,XY[3]	TLX3	TLX3	T-III	NO	YES	0.36	5F	14E
M174	2	M	129.99	T		Bone Marrow	45,XY,der(5)t(5;9)(p14;q11) OR dic(5;9)(p14;p1?2),del(6)(q1?5;q2?4)[15]/46,XY[6]	SIL-TAL1^{del}	TAL1	T-IV	NO	YES	0.31	5F	14E
M180	6	F		T				ND	ND	ND			0.3	2E, 2F	5A, 6B, 6D, 6E, 6F, 6G, 14E
M181				T											14B, 14C
M159	2	M	199.8	T	NO		46,XY,del(6)(q22,q26)	ND	ND	T-II	YES	NO	0.26	5F, 5G	14E
M204	5	M		T		Blood	46,XY[19]	TLX3	TLX3	T-III	NO	YES	0.25	5F, 5G	14E
M143	15	M		T		Bone Marrow		ND	ND	T-II	YES	NO	0.22	5F, 5G	10A, 10B, 10C, 10D, 10E, 12B, 12E, 12F, 14E
M118	8	M	460	T		Blood	46, XY, [23]	SIL-TAL1^{del}	TAL1	T-III	YES	NO	0.2	5F, 5G	14E
M185	15	M		T			46,XY,t(10;14)(q24;q11),del(11)(q23)[3]/46,XY[10]	TLX1	TLX1	T-III	NO	YES	0.2	2E, 2F	3, 5C, 6B, 6D, 14E
M197	11	M	45.19	T			46, XY, del(6)(q?16q2?5),del(9)(p2?4p1?3)<br>CGH: del SYNCRYP et SNHG5 (SNORD50A et SNORD50B) del(CDKN2A/CDKN2B)	SIL-TAL1^{del}	TAL1	T-III > T-IV	NO	YES	0.18	2E, 2F, 3G, 5B, 5C,5E, 5F	3, 5C, 6B, 6D, 6E, 6F, 6G, 14E
M89			165.7	T			t(1;14)	ND	TAL1	ND			0.17	5G	14E
M126	17	M	54	T		Bone Marrow	46, XY, (5;8)(q12-13;p12-21),<br>9?del(13)(q1?q3)? Der(14)t(5;14)(q35;q32)<br>[9]/46 idem +der(5) +7 [3]/46, XY [8]	TLX3	TLX3	T-IV	NO	YES	0.15	5F, 5G	14E
M119	9	M	130	T		Blood	46, XY, [24]	ND	ND	T-IV	NO	YES	0.12	5F, 5G	14E
M117	13	M	600	T			46, XY, add(10)(q2?4) [2]/46, XY, [14]	TLX1	TLX1	T-III	NO	YES	0.11	5F, 5G	14E
M160	12	F	302.8	T		Blood	46,XX[10].nuc ish(TLX3x2)[150],(TLX1x2)[200], (ABL1x2,BCRx1)[18/200]	ND	ND	T-III	NO	YES	0.067	5F	14E
M113	5	F	162.1	T		Blood	46, XX, t(8;14) and t(6;7) cMyc/TCRab	TLX3	TLX3	T-IV	NO	YES	0.055	5F, 5G	14E
M136	5	M		T				ND	ND				0.012		14E
M103	8	M	48	T	NO		46, XY [22]	ND	ND	T-III	NO	YES		2E, 2F, 3B	3, 5C, 6B, 6D, 6E, 6F, 6G, 9B, 9C, 9D, 9E
M105	12	M	129	T	NO	Blood	t(11;14)(p13;q11)[18]/46, XY [2]	ND	TAL1/LMO2	T-III				1I, 2E, 2F	6B, 6D, 6E, 6F, 6G
M106	6	F	743	T	YES		46, XX, t(11;14)(11p13;q11)	SIL-TAL1^{del}	TAL1	T-IV	NO	NO		1H, 1I, 2E, 2F, 2I, 2J, 3B, 3C, 3D, 3E, 3F, 3G, 3H, 5B, 5C, 5D, 5E	3, 5B, 5C, 6B, 6D, 6E, 6F, 6G, 9A, 9B, 9C, 9D, 9E, 14B, 14C, 15A, 15B, 15C
M108	1	M	708	T	YES	Blood	46, XY, t(6;7) (q23;q34) [18]/46, XY [2]	ND	ND	T-III				2E, 2F	3, 5C, 6B, 6D
M114	16	M	362	T	NO		46, XY, t(11;14)(p13;q11) [20]	ND	ND	T-III				2E, 2F	3, 5C, 6B, 6D
M18	6	M	115	T			46, XY del(9)(p21)[10]	SIL-TAL1^{del}	TAL1	T-IV	?	NO		1B, 1C, 1D, 1E, 1F, 1G, 2A, 2B, 2C, 2D, 2E, 2F, 2G, 2H, 3B, 3C, 3D, 3E, 3G, 5A, 5B, 5E	1, 2, 3, 4, 5C, 6A, 6B, 6C, 6D, 6E, 6F, 6G, 8A, 8B, 9B, 9C, 9D,9E, 14A
M192	8	M	187.63	T		Blood	47,XY,dup(6)(q16q21),t(11;14)(p13;q3?1),+20[21]/47,sl,del(12)(p11p13)[3]	ND	ND	T-IV	YES	NO		2E, 2F	3, 5C, 6B, 6D
M69	12	F	239	T	NO	Blood	46, XX, del(6)(q14;q24) [9]/46, XX [1]	SIL-TAL1^{del}	TAL1	ND				1I, 2E, 2F, 3B, 3F, 3G, 3H, 3I, 5C	3, 5C, 6B, 6D, 6E, 6F, 6G, 9B, 9C, 9D,9E
MAP693				T				ND	ND	ND				2E, 2F	6B
MAP740				T				ND	ND	ND				2E, 2F	3, 5C, 6B, 6D, 6E, 6F, 6G
M104				T		Blood		TLX3	TLX3	ND				4A, 4B, 4C, 4D, 4E, 4F, 4G	12A, 12E, 12F
M104R				T		Blood		TLX3	TLX3	ND				4A, 4B, 4C, 4D, 4E, 4F, 4G	12A, 12E, 12F
M143R				T		Bone Marrow		ND	ND	ND					10A, 10B,10C, 10D, 10E, 12B, 12E, 12F
M187				T		Bone Marrow		SIL-TAL1^{del}	TAL1	ND					10F, 10G, 10H, 10I, 10J, 12C, 12E, 12F
M187R				T		Blood		SIL-TAL1^{del}	TAL1	ND					10F, 10G, 10H, 10I, 10J, 12C, 12E, 12F
M220	12	F		T		Bone Marrow		ND	ND	ND				2F, 5B	
M221	2	M		T		Bone Marrow		ND	ND	ND				2F	
M222	5	M		T		Blood		SIL-TAL1^{del}	TAL1	ND				2F, 5B	14B, 14C
M223				T											14B, 14C
M225	14	F	138.72	T		Blood									14B, 14C, 14F
PPO	19			B				IGH-CEBPE ZEB2^{mut}							7A, 7B
RIG	19			B				NUP214-ABL1							7B
UEL	18			B				PAX5 fusion							7A, 7B
